# Supplementary material for: Stereodivergent synthesis of chiral amines bearing vicinal stereocenters via hydroamination of trisubstituted alkenes
Source: Nat Commun. 2026 Mar 4;17:3431. doi: 10.1038/s41467-026-70294-6 (PMC13076855; doi:10.1038/s41467-026-70294-6)
Supplement: Supplementary file 1 — Supplementary Information [file 41467_2026_70294_MOESM1_ESM.pdf]

Supplementary Information

Stereodivergent Synthesis of Chiral Amines bearing Vicinal Stereocenters via Hydroamination of Acyclic Trisubstituted Alkenes

Haohao Bai,[1] Mingchao Li,[2] Xiuping Wang,[1] Tao Jiang,[1] Lintao Zeng,*[2] Lanlan Zhang,[1] and Chao Wang*[1]

[1]Tianjin Key Laboratory of Structure and Performance for Functional Molecules; College of Chemistry, Tianjin Normal University, Tianjin 300387 (P. R. China).

[2]School of Light Industry and Food Engineering, Guangxi University, Nanning 530004, (P. R. China).

E-mail: chwang@tjnu.edu.cn; zlt1981@126.com.

Table of Contents

1. General remarks S3

2. Conditional optimization S5

3. Preparation of substrates S9

4. Procedure for hydroamination of acyclic trisubstituted alkenes. S21

5. Unsuccessful substrates S66

6. Stereodivergent synthesis S67

7. Late-stage decoration of drug-like molecules S70

8. Gram-scale reaction, product derivatizations and synthetic potentials S76

9. Mechanism experiment S83

10. X-ray crystallographic data S94

11. NMR spectra S98

12. Supplementary references S201

1. General remarks

All the manipulations were performed in an argon-filled glovebox, unless mentioned otherwise. Anhydrous solvent was purchased from commercial sources and transferred under argon atmosphere. Alkene substrates and amine electrophiles were prepared according to previously reported procedures. $NiBr_2$•DME (CAS 28923-39-9) was purchased from Heowns. Other reagents were purchased from Meryer (Shanghai) Biochemical Technology Co., Ltd. Adamas-beta®, Energy Chemicals, Bidepharm and used directly without further purification unless otherwise specified. ^{1}H NMR, ^{13}C NMR spectra were recorded using Bruker 400/600 MHz NMR spectrometer and Zhongke-Niejin 400 MHz NMR spectrometer. ^{1}H NMR and ^{13}C NMR spectra were referenced to resonances of the residual protons in the deuterated solvents. Multiplicities are recorded as: s = singlet, d = doublet, t = triplet and m = multiplet. GC-MS analysis was performed on Shimadzu GC-2010 gas chromatography coupled to a Shimadzu QP2010 mass selective detector. Analytical HPLC/MS was performed with an Agilent 6520 Series HPLC; X-Ray Diffraction (XRD) was carried out on an Bruker D8 Venture Metaljet Photon II. Reactions carried out at elevated temperature were heated using oil bath.

Medium-sized screw-cap test tubes (4 mL) were used for all 0.20 mmol scale reactions:15 x 45 mm tubes.

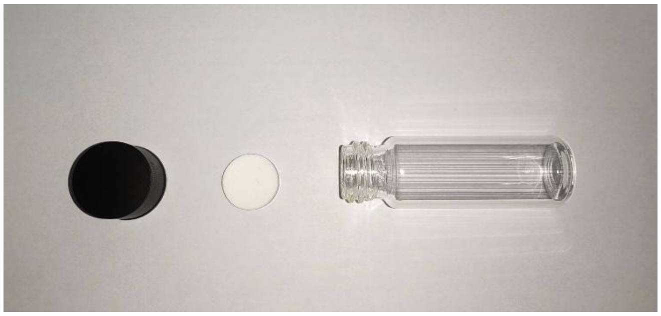

Supplementary Fig. 1. Reaction Tube (4 mL)

100 mL round bottom pressure vessel (Synthware) was used for 5.0 mmol-scale reaction.

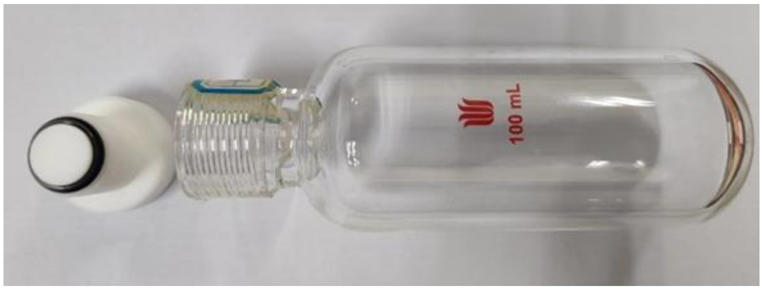

Supplementary Fig. 2. Reaction Tube (100 mL)

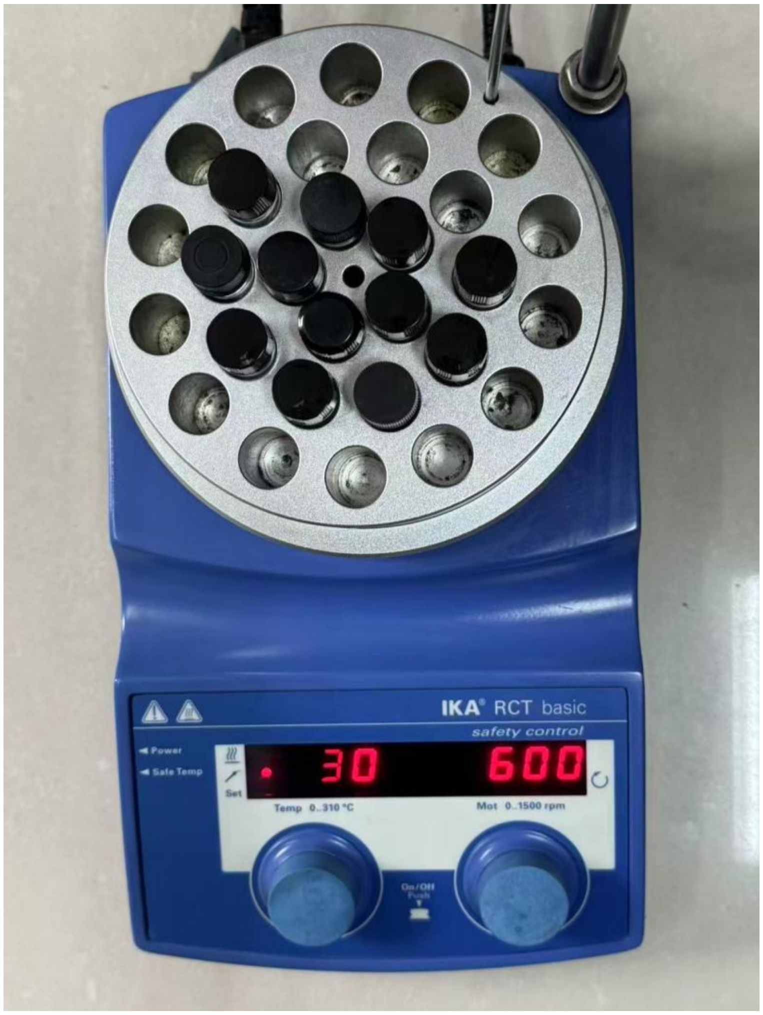

Supplementary Fig. 3. Reaction on Process

2. Conditional optimization

Supplementary Table 1. Screening of ligands.

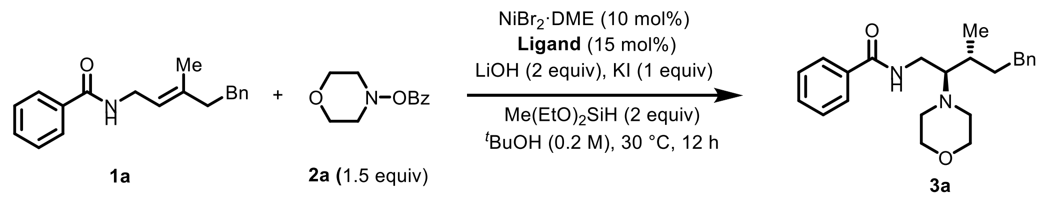

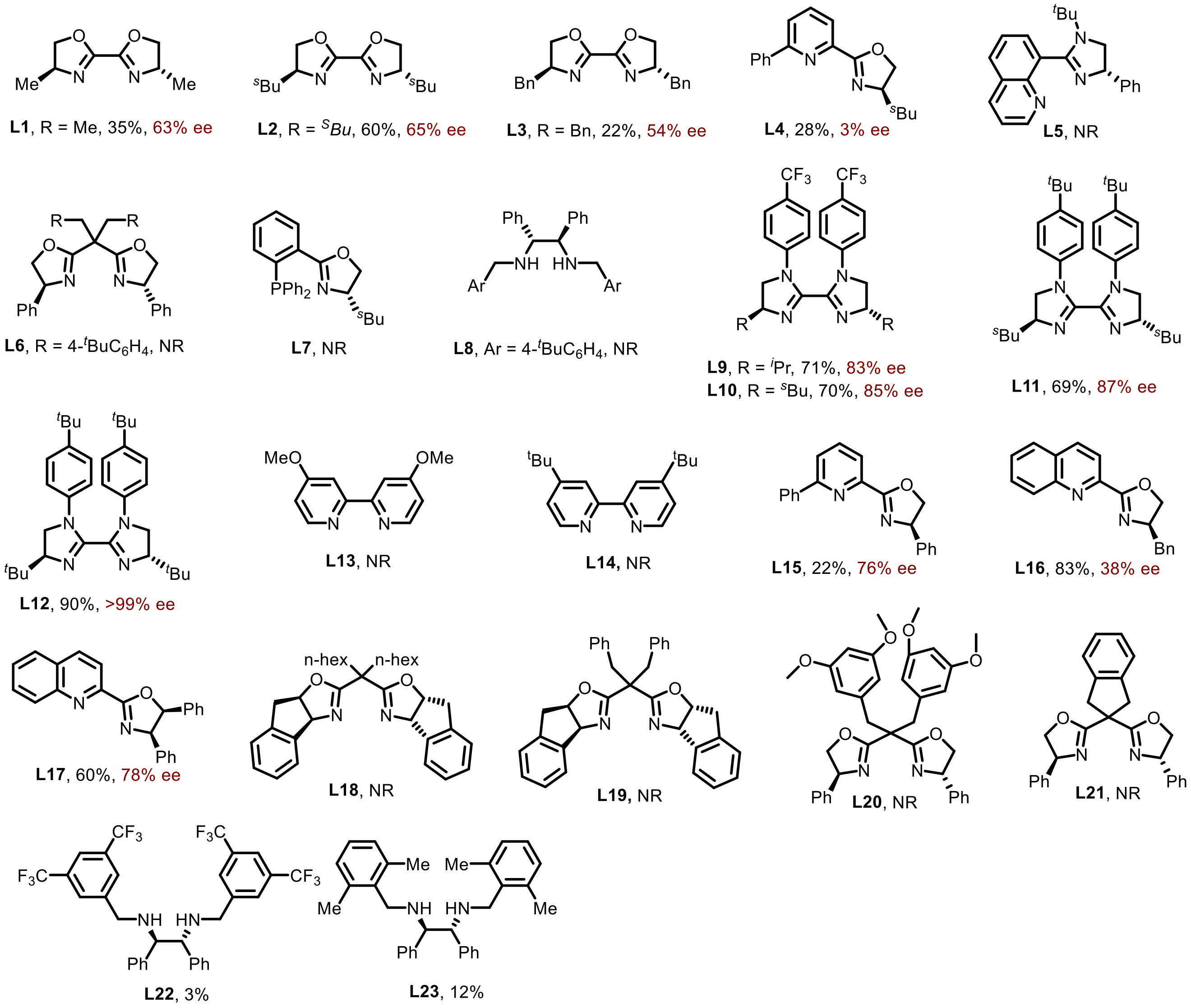

Supplementary Table 2. Screening of solvents.

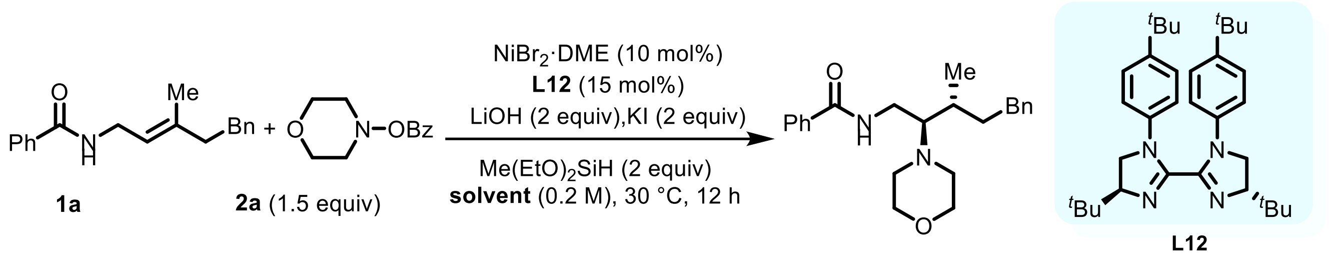

entry	**solvent**	yield (%)	ee (%)	dr
1	^{i}PrOH	42	95	>20:1
2	2-Methyl-2-butanol	77	96	>20:1
3	^{t}BuOH	98	>99	>20:1
4	Toluene	N.D.	/	/
5	DMF	N.D.	/	/
6	1,4-Dioxane	N.D.	/	/
7	DMSO	N.D.	/	/
8	CH_3CN	N.D.	/	/
9	THF	N.D.	/	/

Supplementary Table 3. Screening of bases.

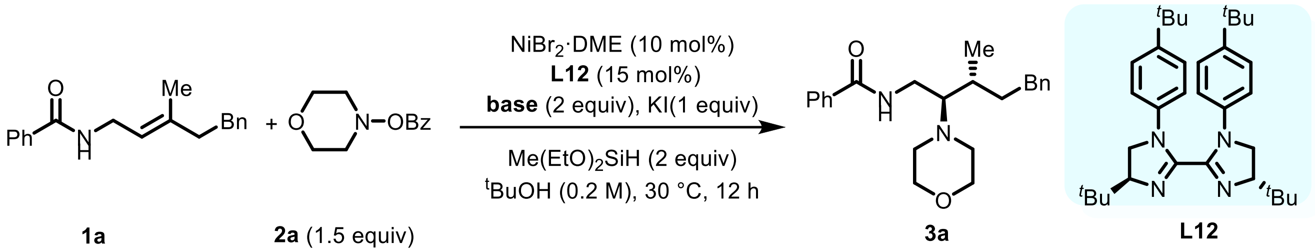

entry	**base**	yield (%)	ee (%)	dr
1	Na_2CO_3	6	96	>20:1
2	K_2CO_3	20	94	>20:1
3	$K_3PO_4 \cdot H_2O$	33	94	>20:1
4	LiOH	98	>99	>20:1
5	Li_3PO_4	N.D.	/	/
6	MeONa	N.D.	/	/
7	^{t}BuOLi	N.D.	/	/
8	CsF	N.D.	/	/
9	KOAC	N.D.	/	/

Supplementary Table 4. Screening of catalysts.

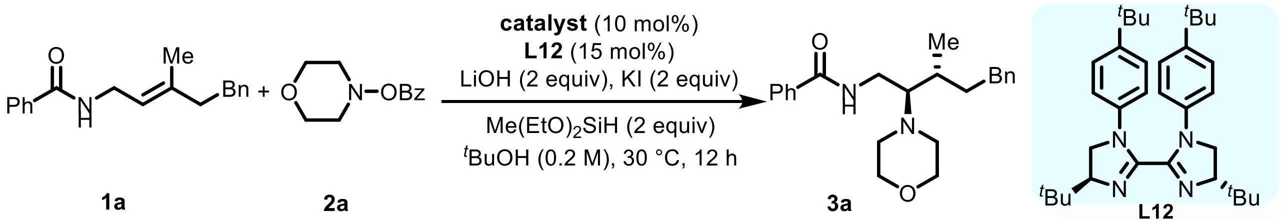

entry	**catalyst**	**yield** (%)	**ee** (%)	**dr**
1	$NiCl_2$	41	97	>20:1
2	$NiBr_2$	39	97	>20:1
3	$Ni(acac)_2$	62	96	>20:1
4	$Ni(OTf)_2$	79	97	>20:1
5	$NiCl_2 \cdot 6H_2O$	86	99	>20:1
6	$NiCl_2 \cdot DME$	79	98	>20:1
7	$NiBr_2 \cdot DME$	98	>99	>20:1
8	$CoBr_2$	N.D.	/	/
9	$FeCl_2$	N.D.	/	/

Supplementary Table 5. Screening of additive.

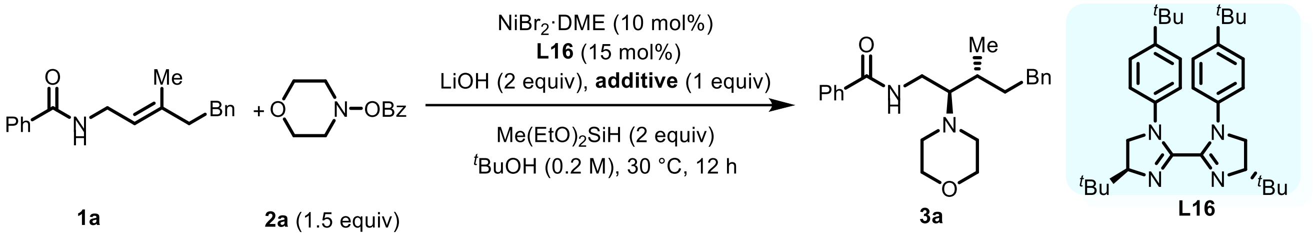

entry	**additive**	yield (%)	ee (%)	dr
1	LiI	67	97	>20:1
2	NaI	64	98	>20:1
3	CoPc	49	98	>20:1
4	^{t}BuOLi	51	98	>20:1
5	w/o KI	43	99	>20:1
6	KI	98	>99	>20:1
7	$ZnCl_2$	5	/	/

3. Preparation of substrates

Synthesis of alkene substrate

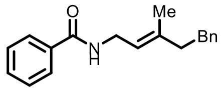

1a

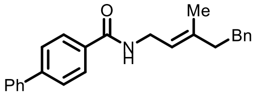

1b

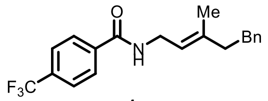

1c

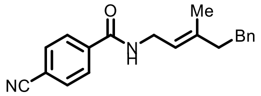

1d

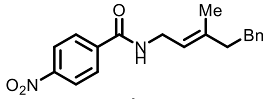

1e

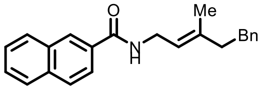

1f

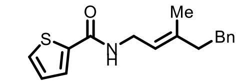

1g

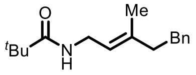

1h

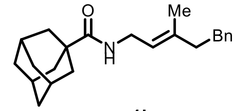

1i

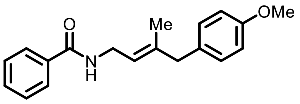

1j

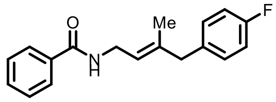

1k

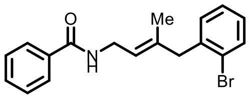

1l

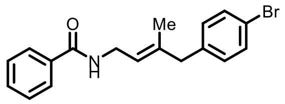

1m

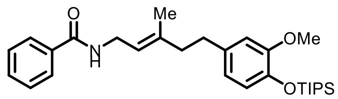

1n

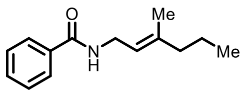

1o

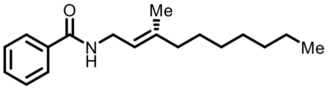

1p

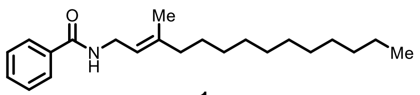

1q

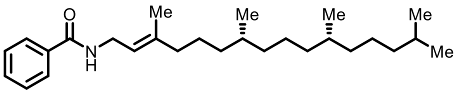

1r

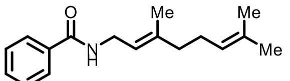

1s

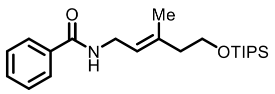

1t

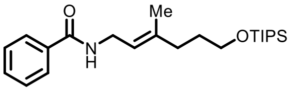

1u

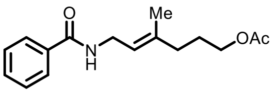

1v

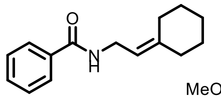

1w

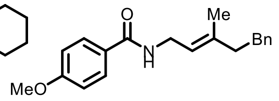

1x

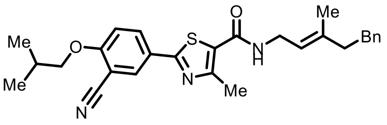

1y

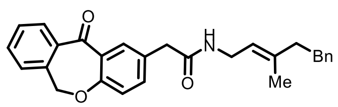

1z

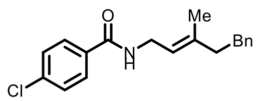

1aa

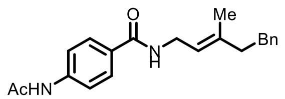

1ab

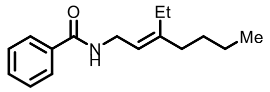

1ac

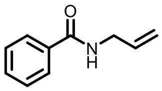

8a

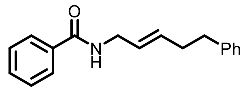

8b

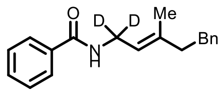

1a-*d*

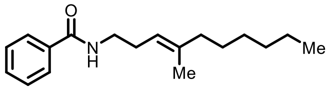

1ad

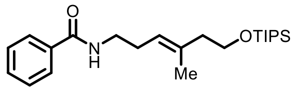

1ae

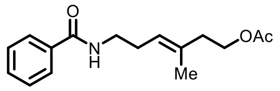

1af

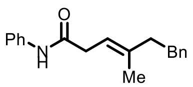

1ag

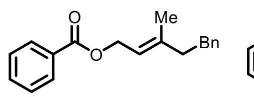

1ah

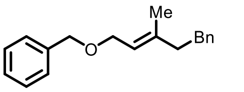

1ai

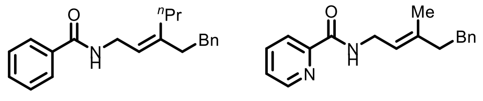

1aj **1ak**

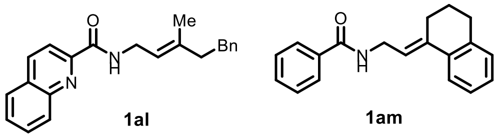

1al **1am**

Compounds **1a-1am** were synthesized according to the literature.[1]

General procedure 1

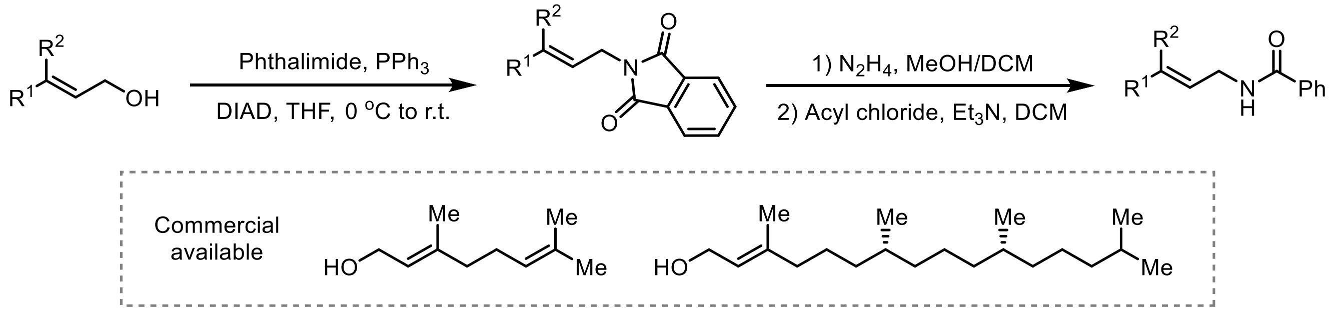

A flame-dried Schlenk flask was charged with phthalimide (22 mmol, 1.1 equiv) and PPh_3 (22 mmol, equiv) under an argon atmosphere. Anhydrous THF (0.20 M) was added, followed by dropwise addition of the allylic alcohol (20 mmol, 1.0 equiv) at room temperature. The mixture was cooled to 0 °C, and diisopropyl azodicarboxylate (DIAD) (22 mmol, 1.1 equiv) was added dropwise via syringe. The reaction mixture was allowed to warm to room temperature and stirred for 16 h. Upon completion, the solvent was removed *in vacuo*. The crude residue was purified by flash column chromatography (PE/EtOAc gradient elution) to afford the imide.

To a flask charged with imide in MeOH/DCM (1/1, v/v, 0.20 M) was added hydrazine hydrate (2.0 equiv). The mixture was stirred at room temperature until complete conversion as indicated by TLC. The mixture was filtered through Celite and the solvent was evaporated. The crude allylamine was dissolved in DCM (0.20 M) and Et_3N and acyl chloride were added at 0 ° C. The mixture was then allowed to warm to r.t. and stirred for 2 h. The reaction was quenched with saturated NH_4Cl (20 mL) and the mixture was extracted with DCM (3 × 20 mL). The organic phase was combined and dried with $MgSO_4$. The solvent was evaporated, and the residue was purified by silica gel column chromatography with PE/EtOAc as eluent to give the allylamide (**1r-1s, 8a-8b**).

General procedure 2

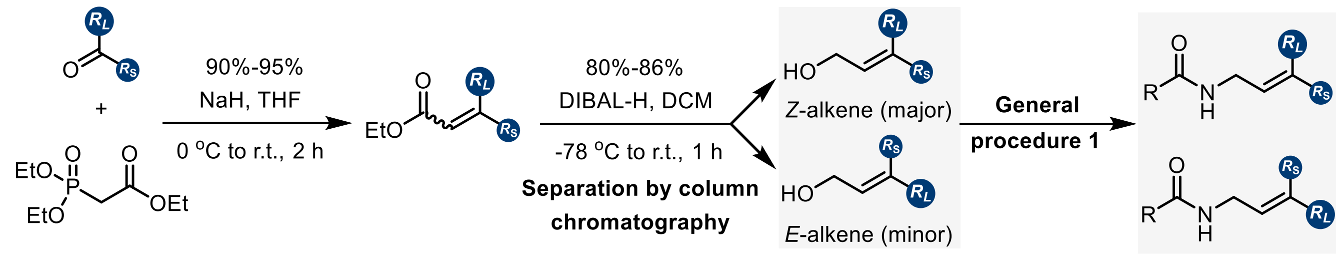

To a solution of THF (0.40 M) and NaH (60% dispersion in mineral oil, 1.1 equiv), triethyl phosphonoacetate (1.1 equiv.) was added dropwise at 0 ° C. The reaction was stirred for 30 min. T A solution of ketone (20 mmol, 1.0 equiv) in anhydrous THF (10 mL) was then added via cannula over 15 min, maintaining internal temperature <5 °C. The reaction was stirred until complete conversion as indicated by TLC. The reaction mixture was quenched with saturated NH_4Cl (20 mL). The aqueous layer was extracted with EtOAc (3 × 30 mL), and the combined organic extracts were dried over anhydrous $MgSO_4$, filtered, and concentrated *in vacuo*. Purification by flash column chromatography (PE/EtOAc = 20:1) afforded a,b-unsaturated ester.

To a solution of a,b-unsaturated ester (20 mmol, 1.0 equiv) in dry DCM (0.50 M) was added DIBAL-H (2.3 equiv, 1.5 M in toluene) dropwise at -78 ° C. The cooling bath was removed, and the reaction mixture was allowed to warm slowly to room temperature. After stirring for 2 h at rt, the mixture was cooled to 0 °C and quenched by dropwise addition of 15% aqueous NaOH (2 M). The mixture was stirred for another 15 min and filtered through Celite. The solvent was evaporated and the residue was purified by silica gel column chromatography with EtOAc/PE as eluent to give the allylic alcohol. Subsequent synthesis steps referring to general procedure 1 afforded trisubstituted olefins **1a-1q**, **1w-1ab**,

(3*r*,5*r*,7*r*)-*N*-((*E*)-3-methyl-5-phenylpent-2-en-1-yl)adamantane-1-carboxamide (1i)

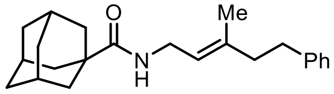

^{1}H NMR (400 MHz, Chloroform-*d*) δ 7.39–7.33 (m, 2H), 7.26 (t, *J* = 8.9 Hz, 3H), 5.42 (s, 1H), 5.22 (t, *J* = 6.9 Hz, 1H), 3.88 (t, *J* = 6.0 Hz, 2H), 2.88–2.72 (m, 2H), 2.40 (t, *J* = 7.9 Hz, 2H), 2.13 (s, 3H), 1.93–1.89 (m, 6H), 1.84–1.77 (m, 9H). **^{13}C NMR (**101 MHz, Chloroform-*d*) δ 177.9, 142.0, 139.3, 128.6, 128.4, 125.9, 121.1, 41.4, 40.7, 39.4, 37.4, 36.7, 34.4, 28.3, 16.5. **HRMS (ESI)** m/z calculated for $C_{23}H_{32}NO$ $[M+H]^+$ 338.2478, found 338.2474.

(*E*)-*N*-(3-methyl-5-phenylpent-2-en-1-yl)pivalamide (1h)

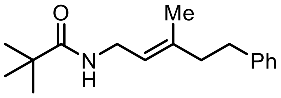

^{1}H NMR (400 MHz, Chloroform-*d*) δ 7.30–7.24 (m, 2H), 7.21–7.13 (m, 3H), 5.37 (s, 1H), 5.19–5.09 (m, 1H), 3.84–3.77 (m, 2H), 2.77–2.69 (m, 2H), 2.35–2.28 (m, 2H), 1.72 (s, 3H), 1.18 (s, 9H). **^{13}C NMR** (101 MHz, Chloroform-*d*) δ 178.3, 142.0, 139.3, 128.6, 128.4, 126.0, 121.0, 38.7, 37.7, 34.4, 27.8, 16.5. **HRMS (ESI)** m/z calculated for $C_{17}H_{26}NO$ $[M+H]^+$ 260.2009, found 260.2010.

(*E*)-*N*-(4-(4-methoxyphenyl)-3-methylbut-2-en-1-yl)benzamide (1j)

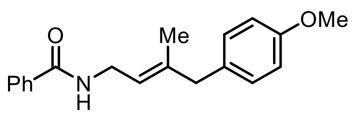

^{1}H NMR (400 MHz, Chloroform-*d*) δ 7.76 (d, *J* = 7.2 Hz, 2H), 7.50 (t, *J* = 7.3 Hz, 1H), 7.43 (t, *J* = 7.5 Hz, 2H), 7.09 (d, *J* = 8.2 Hz, 2H), 6.84 (d, *J* = 8.2 Hz, 2H), 6.03 (s, 1H), 5.35 (t, *J* = 7.1 Hz, 1H), 4.09 (t, *J* = 6.2 Hz, 2H), 3.79 (s, 3H), 3.27 (s, 2H), 1.67 (s, 3H). **^{13}C NMR** (101 MHz, Chloroform-*d*) δ 166.9, 162.2, 142.0, 139.6, 128.8, 128.6, 128.4, 127.1, 126.0, 120.9, 113.8, 55.6, 41.5, 38.0, 34.4, 16.5. **HRMS (ESI)** m/z calculated for $C_{19}H_{22}NO_2$ $[M+H]^+$ 296.1645, found 296.1648.

(*E*)-*N*-(4-(4-fluorophenyl)-3-methylbut-2-en-1-yl)benzamide (1k)

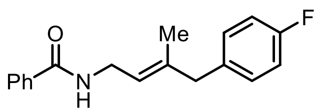

^{1}H NMR (400 MHz, Chloroform-*d*) δ 7.77 (d, *J* = 7.6 Hz, 2H), 7.49 (t, *J* = 7.7 Hz, 1H), 7.41 (t, *J* = 7.5 Hz, 2H), 7.15–7.07 (m, 2H), 6.96 (t, *J* = 8.5 Hz, 2H), 6.23 (s, 1H), 5.35 (t, *J* = 7.1 Hz, 1H), 4.08 (t, *J* = 6.2 Hz, 2H), 3.28 (s, 2H), 1.65 (s, 3H). **^{13}C NMR (**101 MHz, Chloroform-*d*) δ 167.5, 161.6 (d, *J* = 244.0 Hz), 139.3, 135.0 (d, *J* = 3.3 Hz), 134.7, 131.5, 130.4 (d, *J* = 7.8 Hz), 128.7, 127.0, 122.1, 115.2 (d, *J* = 21.2 Hz), 45.2, 38.1, 16.3. **HRMS (ESI)** m/z calculated for $C_{18}H_{18}FNNaO$ $[M+Na]^+$ 306.1265, found 306.1270.

(*E*)-*N*-(4-(2-bromophenyl)-3-methylbut-2-en-1-yl)benzamide (1l)

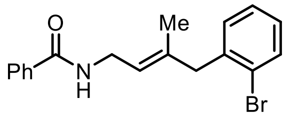

^{1}H NMR (400 MHz, Chloroform-*d*) δ 7.78 (d, *J* = 8.0 Hz, 2H), 7.57 (d, *J* = 8.0 Hz, 1H), 7.52 (t, *J* = 7.8 Hz, 1H), 7.45 (t, *J* = 7.7 Hz, 2H), 7.31–7.27 (m, 1H), 7.23 (d, *J* = 7.1 Hz, 1H), 7.11 (t, *J* = 7.5 Hz, 1H), 6.08 (s, 1H), 5.25 (t, *J* = 7.0 Hz, 1H), 4.11 (t, *J* = 6.1 Hz, 2H), 3.50 (s, 2H), 1.77 (s, 3H). **^{13}C NMR** (101 MHz, Chloroform-*d*) δ 167.4, 138.7, 138.2, 134.7, 133.0, 131.5, 131.2, 128.7, 128.1, 127.5, 127.0, 125.3, 122.3, 45.3, 38.1, 16.8. **HRMS (ESI)** m/z calculated for $C_{18}H_{19}BrNO$ $[M+H]^+$ 344.0645, found 344.0642.

(*E*)-*N*-(5-(3-methoxy-4-((triisopropylsilyl)oxy)phenyl)-3-methylpent-2-en-1-yl)benzamide (1n)

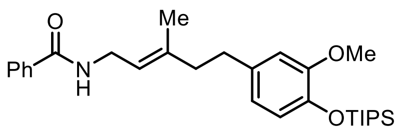

^{1}H NMR (400 MHz, Chloroform-*d*) δ 7.79–7.72 (m, 2H), 7.51–7.43 (m, 1H), 7.44–7.36 (m, 2H), 6.77 (d, *J* = 8.0 Hz, 1H), 6.64 (d, *J* = 2.1 Hz, 1H), 6.58 (dd, *J* = 8.0, 2.1 Hz, 1H), 6.15 (s, 1H), 5.26 (td, *J* = 7.1, 1.4 Hz, 1H), 4.05–3.98 (m, 2H), 3.76 (s, 3H), 2.70–2.61 (m, 2H), 2.29 (t, *J* = 7.9 Hz, 2H), 1.74 (s, 3H), 1.28–1.18 (m, 3H), 1.08 (d, *J* = 7.3 Hz, 18H). **^{13}C NMR (**101 MHz, Chloroform-*d*) δ 150.7, 143.6, 139.8, 135.1, 134.7, 131.4, 128.6, 127.0, 120.4, 112.5, 55.6, 41.6, 38.1, 34.1, 18.0, 16.5, 12.9. **HRMS (ESI)** m/z calculated for $C_{29}H_{43}NNaO_3Si$ $[M+Na]^+$ 504.2904, found 504.2908.

(*E*)-*N*-(3-methyl-5-((triisopropylsilyl)oxy)pent-2-en-1-yl)benzamide (1t)

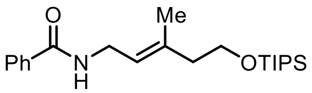

^{1}H NMR (400 MHz, Chloroform-*d*) δ 7.77–7.73 (m, 2H), 7.49–7.44 (m, 1H), 7.43–7.36 (m, 2H), 6.13 (s, 1H), 5.40–5.30 (m, 1H), 4.07–4.01 (m, 2H), 3.77 (t, *J* = 6.8 Hz, 2H), 2.26 (t, *J* = 6.8 Hz, 2H), 1.74 (s, 3H), 1.07–1.02 (m, 21H). **^{13}C NMR** (101 MHz, Chloroform-*d*) δ 167.4, 137.9, 134.8, 131.4, 128.6, 127.0, 121.7, 62.2, 43.0, 38.1, 18.1, 16.8, 12.1. **HRMS (ESI)** m/z calculated for $C_{22}H_{38}NO_2Si$ $[M+H]^+$ 376.2666, found 376.2663.

General procedure 3

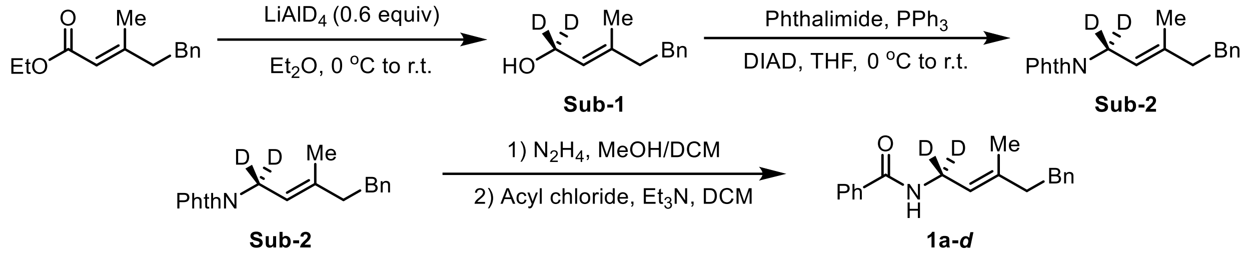

Compounds **1a-*d*** were synthesized according to literature procedures 3.[2] To a suspension of $LiAlD_4$ (492.0 mg, 12.0 mmol) in anhydrous Et_2O (20 mL) at 0 °C, a solution of ethyl *(E)*-3-methyl-5-phenylpent-2-enoate (10.0 mmol, 1.0 equiv) in anhydrous Et_2O (10 mL) was added dropwise. The mixture was warmed to room temperature and stirred for 4 h. The reaction was carefully quenched at 0 °C by sequential addition of saturated aqueous Rochelle's salt (potassium sodium tartrate, 20 mL). The mixture was extracted with EtOAc (2 × 15 mL), and the combined organic layers were washed with brine (30 mL), dried over anhydrous Na_2SO_4, filtered, and concentrated under reduced pressure. Purification by flash chromatography (PE:EtOAc = 15:1) afforded (*E*)-3-methyl-5-phenylpent-2-en-1,1-*d*$_2$-1-ol **1a-*d*** (1.42 g, 80% yield) as a colorless oil (>98% of deuterium atom content).

(*E*)-3-methyl-5-phenylpent-2-en-1,1-*d*$_2$-1-ol (Sub-1)

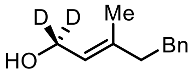

^{1}H NMR (400 MHz, Chloroform-*d*) δ 7.2–7.2 (m, 2H), 7.1–7.1 (m, 3H), 5.3 (s, 1H), 2.7–2.6 (m, 2H), 2.3–2.2 (m, 2H), 1.7 (d, *J* = 1.4 Hz, 3H). **HRMS (ESI)** m/z calculated for $C_{12}H_{15}D_2O$ $[M+H]^+$ 179.1339, found 179.1338.

(*E*)-*N*-(3-methyl-5-phenylpent-2-en-1-yl-1,1-*d*$_2$)benzamide (1a-*d*)

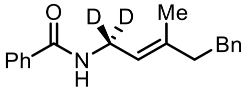

^{1}H NMR (400 MHz, Chloroform-*d*) δ 7.7–7.6 (m, 2H), 7.4–7.4 (m, 1H), 7.3–7.3 (m, 2H), 7.2–7.1 (m, 2H), 7.1–7.0 (m, 3H), 6.0 (s, 1H), 5.2 (d, *J* = 2.1 Hz, 1H), 2.7–2.6 (m, 2H), 2.3–2.2 (m, 2H), 1.7 (d, *J* = 1.4 Hz, 3H). **HRMS (ESI)** m/z calculated for $C_{19}H_{20}D_2NO$ $[M+H]^+$ 282.1821, found 282.1819.

Synthesis of amine electrophiles

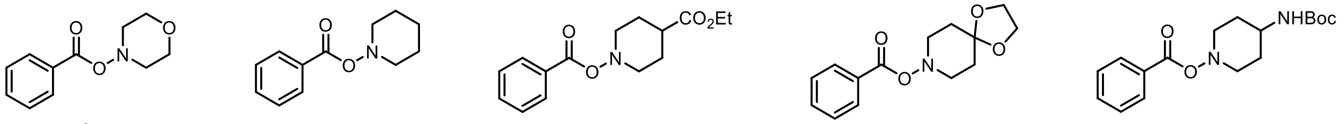

2a **2b** **2c** **2d** **2e**

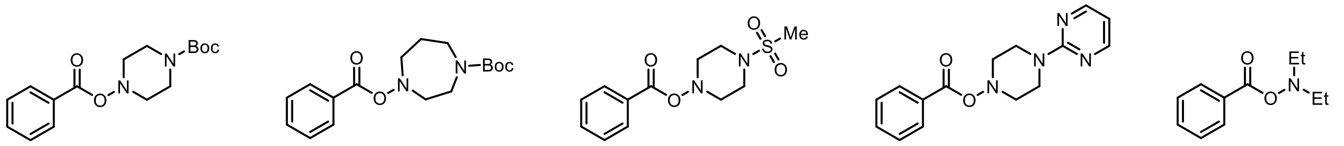

2f **2g** **2h** **2i** **2j**

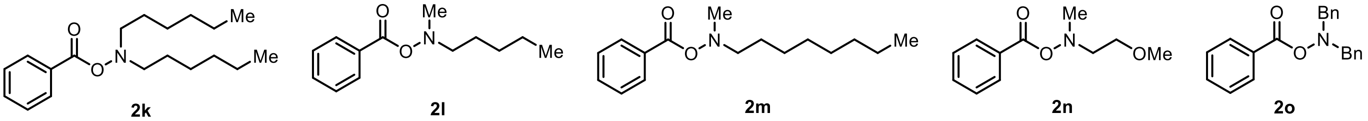

2k **2l** **2m** **2n** **2o**

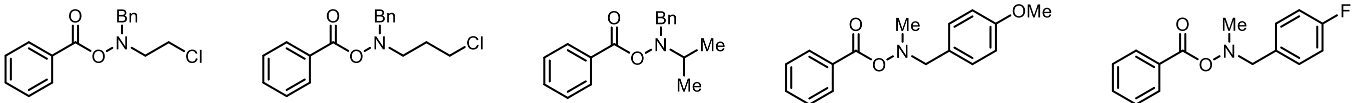

2p **2q** **2r** **2s** **2t**

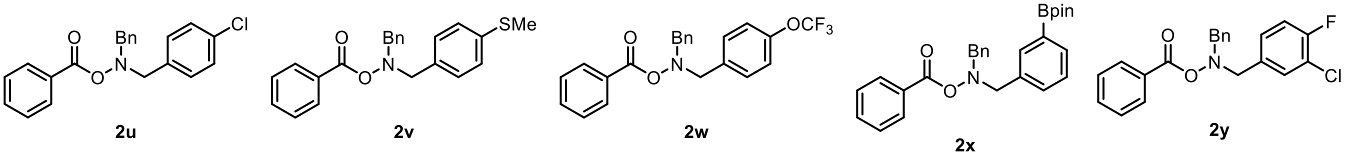

2u **2v** **2w** **2x** **2y**

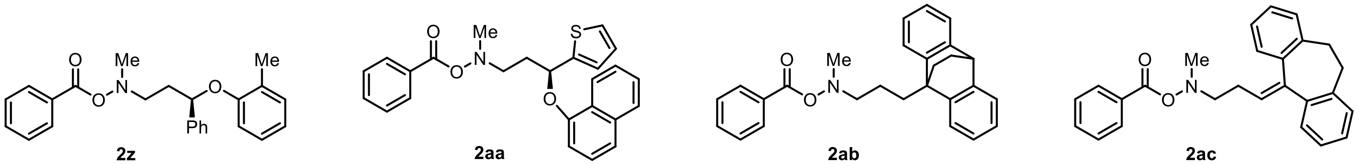

2z **2aa** **2ab** **2ac**

The structures of compounds **2a-2d, 2o, 2s**, **2t** and **2w** were determined based on the reports in the literature through NMR comparison. The remaining compounds were synthesized according to the methods introduced below and the product structures were confirmed by NMR.

General procedure 4

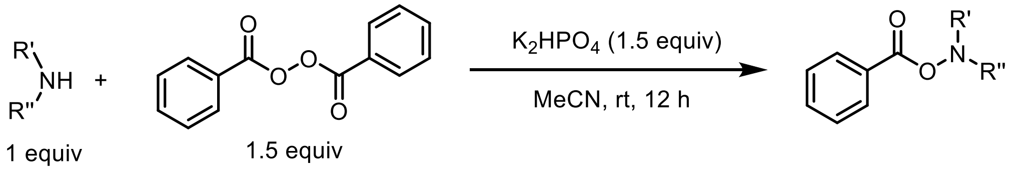

Compound **2a-2o**, **2r-2t**, and **2z-2ac** were synthesized according to literature procedures 4.[3] Prepare a round-bottom flask, add benzoic peroxyanhydride (1.5 equiv), K_2HPO_4 (1.5 equiv) and MeCN (0.2M)

into the flask. The reaction mixture is slowly added to the secondary amine (1.0 equiv) under stirring conditions at room temperature for 12 hours. The reaction mixture was monitored by TLC. After the disappearance of the starting material, filter the reaction system to remove solid impurities. After removal of the solvent, the residue was purified by flash chromatography on silica gel (ethyl acetate:hexane) to yield the corresponding amine electrophiles.

General procedure 5

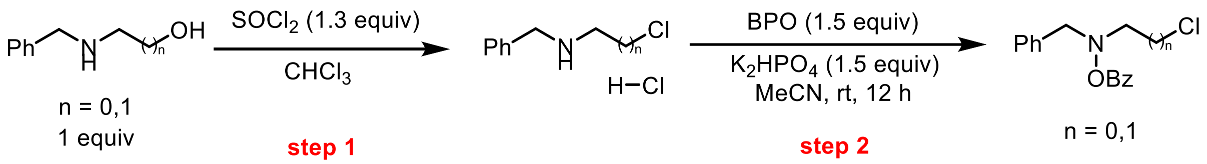

Compound **2p** and **2q** were synthesized according to literature procedures 5.[4]

Step1: To a stirred solution of 2-(benzylamino)ethan-1-ol or 3-(benzylamino)propan-1-ol (132 mmol) in chloroform (200 ml.) at 0 °C was added thionyl chloride (172 mmol) in chloroform (100 mL) dropwise and stirred at rt for 30 min and at reflux for 2 h. Cooled the reaction mixture to RT and solid formed was filtered, washed with ether and dried to get compound as a white solid. The white solid product can obtain the target product secondary by adjusting the PH value to alkaline amine

Step2: Prepare a round-bottom flask, add benzoic peroxyanhydride (1.5 equiv), K_2HPO_4 (1.5 equiv) and MeCN (0.2 M) into the flask. The reaction mixture is slowly added to the secondary amine (1.0 equiv) under stirring conditions at room temperature for 12 hours. The reaction mixture was monitored by TLC. After the disappearance of the starting material, filter the reaction system to remove solid impurities. After removal of the solvent, the residue was purified by flash chromatography on silica gel (ethyl acetate:hexane) to yield the corresponding amine electrophiles.

General procedure 6

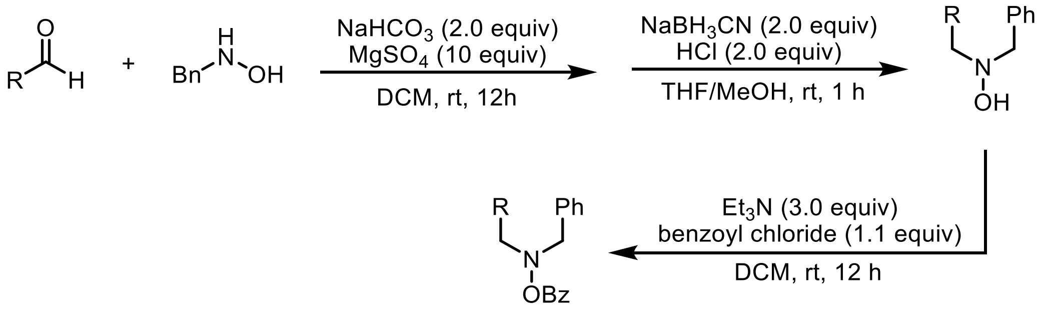

Compound **2u-2y** was synthesized according to literature procedures 6.[5] To a round bottom flask were added the aldehyde (20 mmol, 1.0 equiv.), N benzylhydroxylamine·HCl salt (20 mmol, 1.0 equiv.), $NaHCO_3$ (40 mmol, 2.0 equiv.), $MgSO_4$ (200 mmol, 10 equiv.) and CH_2Cl_2 (50 mL, 0.40 M). The reaction mixture was stirred at room temperature for 12 h, filtered through a sintered glass funnel eluting with CH_2Cl_2, and then concentrated in vacuo with the aid of a rotary evaporator. THF (20 mL, 1.0 M) was added to the residual mixture. A solution of $NaBH_3CN$ (40 mmol, 2.0 equiv.) in MeOH (20 mL) and HCl in ether (40 mL, 1.0 M, 40 mmol) were added simultaneously via syringe over the course of 10 min. The reaction mixture was allowed to stir at room temperature for an additional 4 h and then quenched through the addition of sat. $NaHCO_3$ (aq.). The mixture was extracted with ethyl acetate (three times), and the combined organic layers were concentrated with the aid of rotary evaporator and then purified by column chromatography to yield the N, N-dialkylhydroxylamine (50-90% yield over two steps). To a suspension of N, N-dialkylhydroxylamine (1.0 equiv.), N, N-dimethylpyridin-4 amine (DMAP, 10 mol%) and triethylamine (1.0 equiv.) in CH_2Cl_2 (0.50 M) was added benzoyl chloride (1.0 equiv.) at 0 °C. Then the reaction mixture was allowed to stir at room temperature for 8–12 h until the reaction went to full completion as indicated by TLC analysis. The reaction was quenched with HCl (1 M) and extracted with CH2Cl2 (3 × 50 mL), the combined organics were dried over Na_2SO_4 and concentrated under reduced pressure. The residue was purified by flash chromatography on silica gel to give the product.

4-((*tert*-Butoxycarbonyl)amino)piperidin-1-yl benzoate (2e)

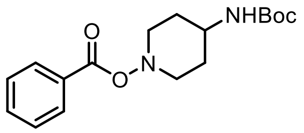

^{1}H NMR (600 MHz, $CDCl_3$) δ 7.98–7.95 (m, 2H), 7.53 (t, J = 7.4 Hz, 1H), 7.40 (t, J = 7.7 Hz, 2H), 4.75–4.53 (m, 1H), 3.53 (t, J = 14.8 Hz, 2H), 3.36–3.17 (m, 1H), 2.92–2.58 (m, 2H), 2.10–1.93 (m, 2H), 1.88–1.69 (m, 2H), 1.42 (s, 9H). **^{13}C NMR** (151 MHz, $CDCl_3$) δ 164.8, 155.2, 133.1, 129.4, 129.2, 128.4, 79.5, 55.8, 47.0, 31.6, 28.4. **HRMS (ESI)** m/z calculated for $C_{17}H_{24}N_2O_4$ $[M+H]^+$ 321.1809, found 321.1809.

***tert*-Butyl 4-(benzoyloxy)piperazine-1-carboxylate (2f)**

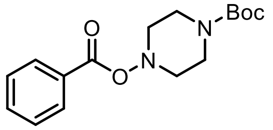

^{1}H NMR (400 MHz, $CDCl_3$) δ 7.99 (d, J = 7.7 Hz, 2H), 7.57 (t, J = 7.4 Hz, 1H), 7.44 (t, J = 7.6 Hz, 2H), 4.15–3.90 (m, 2H), 3.50–3.39 (m, 2H), 3.31 (s, 2H), 3.00–2.86 (m, 2H), 1.47 (s, 9H). **^{13}C NMR** (101 MHz, $CDCl_3$) δ 164.6, 154.5, 133.3, 129.5, 129.1, 128.5, 80.3, 55.9, 28.4. **HRMS (ESI)** m/z calculated for $C_{16}H_{22}N_2O_4$ $[M+H]^+$ 307.1653, found 307.1653.

tert-Butyl 4-(benzoyloxy)-1,4-diazepane-1-carboxylate (2g)

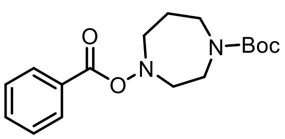

^{1}H NMR (600 MHz, $CDCl_3$) δ 8.00–7.92 (m, 2H), 7.56–7.50 (m, 1H), 7.43–7.37 (m, 2H), 3.71–3.61 (m, 1H), 3.58–3.50 (m, 2H), 3.48 (t, J = 6.5 Hz, 1H), 3.34–3.26 (m, 4H), 2.06–1.96 (m, 2H), 1.44 (d, J = 2.5 Hz, 9H). **^{13}C NMR** (151 MHz, $CDCl_3$) δ 164.5, 155.5, 155.3, 133.1, 129.4, 129.2, 128.5, 79.8 (d, J = 8.4 Hz), 59.5 (d, J = 15.6 Hz), 57.7 (d, J = 39.9 Hz), 45.0 (d, J = 172.0 Hz), 42.3 (d, J = 110.5 Hz), 28.5, 23.78 (d, J = 26.3 Hz). **HRMS (ESI)** m/z calculated for $C_{17}H_{24}N_2O_4$ $[M+H]^+$ 321.1809, found 321.1812.

4-(Methylsulfonyl)piperazin-1-yl benzoate (2h)

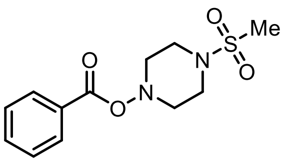

^{1}H NMR (600 MHz, $CDCl_3$) δ 8.00–7.96 (m, 2H), 7.60–7.54 (m, 1H), 7.44 (t, J = 7.8 Hz, 2H), 3.80–3.66 (m, 2H), 3.59–3.46 (m, 2H), 3.25 (t, J = 11.0 Hz, 2H), 3.11 (t, J = 10.6 Hz, 2H), 2.82 (s, 3H). **^{13}C NMR** (151 MHz, $CDCl_3$) δ 164.5, 133.5, 129.5, 128.7, 128.6, 55.2, 44.2, 34.9. **HRMS (ESI)** m/z calculated for $C_{12}H_{16}N_2O_4S$ $[M+H]^+$ 285.0904, found 285.0905.

4-(Pyrimidin-2-yl)piperazin-1-yl benzoate (2i)

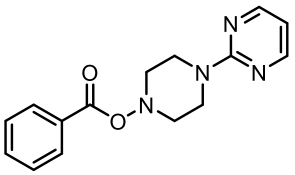

^{1}H NMR (400 MHz, $CDCl_3$) δ 8.35–8.30 (m, 2H), 8.04–7.98 (m, 2H), 7.60–7.52 (m, 1H), 7.43 (t, J = 7.6 Hz, 2H), 6.53 (t, J = 4.8 Hz, 1H), 4.64 (d, J = 13.2 Hz, 2H), 3.55 (t, J = 11.5 Hz, 4H), 3.02 (d, J = 11.0 Hz, 2H). **^{13}C NMR** (101 MHz, $CDCl_3$) δ 164.7, 161.4, 157.8, 133.2, 129.5, 129.2, 128.5, 110.4, 55.9, 42.2. **HRMS (ESI)** m/z calculated for $C_{15}H_{16}N_4O_2$ $[M+H]^+$ 285.1346, found 285.1346.

***O*-benzoyl-*N*,*N*-diethylhydroxylamine (2j)**

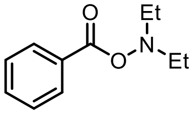

^{1}H NMR (400 MHz, $CDCl_3$) δ 8.05–7.97 (m, 2H), 7.56–7.50 (m, 1H), 7.41 (t, J = 7.7 Hz, 2H), 3.01 (q, J = 7.1 Hz, 4H), 1.15 (t, J = 7.1 Hz, 6H). **^{13}C NMR** (101 MHz, $CDCl_3$) δ 165.9, 133.0, 129.5, 129.2, 128.4, 53.5, 11.9. **HRMS (ESI)** m/z calculated for $C_{11}H_{15}NO_2$ $[M+H]^+$ 194.1176, found 194.1178.

***O*-benzoyl-*N*,*N*-dihexylhydroxylamine (2k)**

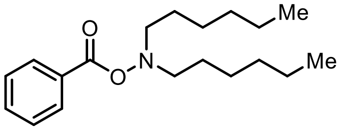

^{1}H NMR (400 MHz, $CDCl_3$) δ 8.06–7.98 (m, 2H), 7.55 (t, J = 7.4 Hz, 1H), 7.42 (t, J = 7.6 Hz, 2H), 2.99–2.90 (m, 4H), 1.63–1.51 (m, 4H), 1.38–1.18 (m, 12H), 0.84 (t, J = 6.7 Hz, 6H). **^{13}C NMR** (101 MHz, $CDCl_3$) δ 165.7,

133.0, 129.5, 129.4, 128.4, 59.9, 31.7, 27.0, 26.8, 22.6, 14.0. **HRMS (ESI)** m/z calculated for $C_{19}H_{31}NO_2$ $[M+H]^+$ 306.2428, found 306.2428.

***O*-benzoyl-*N*-methyl-*N*-pentylhydroxylamine (2l)**

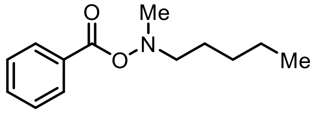

^{1}H NMR (400 MHz, $CDCl_3$) δ 8.00 (d, *J* = 7.7 Hz, 2H), 7.54 (t, *J* = 7.4 Hz, 1H), 7.42 (t, *J* = 7.6 Hz, 2H), 2.98–2.90 (m, 2H), 2.87 (s, 3H), 1.65–1.51 (m, 2H), 1.39–1.21 (m, 4H), 0.86 (t, *J* = 6.8 Hz, 3H). **^{13}C NMR** (101 MHz, $CDCl_3$) δ 165.2, 133.0, 129.4, 129.4, 128.4, 61.3, 47.1, 29.4, 26.7, 22.5, 14.0. **HRMS (ESI)** m/z calculated for $C_{19}H_{31}NO_2$ $[M+H]^+$ 222.1489, found 222.1490.

***O*-benzoyl-*N*-methyl-*N*-octylhydroxylamine (2m)**

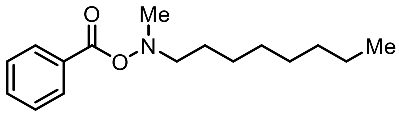

^{1}H NMR (400 MHz, $CDCl_3$) δ 8.03–7.96 (m, 2H), 7.58–7.50 (m, 1H), 7.41 (t, *J* = 7.7 Hz, 2H), 3.02–2.89 (m, 2H), 2.86 (s, 3H), 1.64–1.52 (m, 2H), 1.38–1.30 (m, 2H), 1.29–1.19 (m, 8H), 0.83 (t, *J* = 6.6 Hz, 3H). **^{13}C NMR** (101 MHz, $CDCl_3$) δ 165.2, 133.0, 129.5, 129.4, 128.4, 61.4, 47.1, 31.8, 29.4, 29.2, 27.2, 27.0, 22.7, 14.1. **HRMS (ESI)** m/z calculated for $C_{16}H_{25}NO_2$ $[M+H]^+$ 264.1958, found 264.1958.

***O*-benzoyl-*N*-(2-methoxyethyl)-*N*-methylhydroxylamine (2n)**

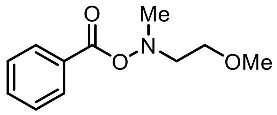

^{1}H NMR (600 MHz, $CDCl_3$) δ 7.95–7.90 (m, 2H), 7.50–7.44 (m, 1H), 7.39–7.30 (m, 2H), 3.52 (t, *J* = 5.8 Hz, 2H), 3.22 (s, 3H), 3.10 (t, *J* = 5.7 Hz, 2H), 2.85 (s, 3H). **^{13}C NMR** (151 MHz, $CDCl_3$) δ 165.0, 133.0, 129.4, 129.2, 128.4, 69.7, 60.3, 58.8, 47.4. **HRMS (ESI)** m/z calculated for $C_{11}H_{15}NO_3$ $[M+H]^+$ 210.1125, found 210.1126.

***O*-benzoyl-*N*-benzyl-*N*-(2-chloroethyl)hydroxylamine (2p)**

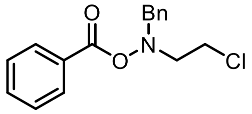

^{1}H NMR (600 MHz, $CDCl_3$) δ 7.84–7.78 (m, 2H), 7.41–7.37 (m, 1H), 7.30–7.22 (m, 4H), 7.21–7.15 (m, 2H), 7.14–7.10 (m, 1H), 4.10 (s, 2H), 3.56–3.51 (m, 2H), 3.23–3.16 (m, 2H). **^{13}C NMR** (151 MHz, $CDCl_3$) δ 165.1, 134.9, 133.3, 129.8, 129.7, 129.5, 129.0, 128.5, 128.5, 128.1, 63.7, 59.4, 40.4. **HRMS (ESI)** m/z calculated for $C_{16}H_{16}ClNO_2$ $[M+H]^+$ 290.0943, found 190.0948.

***O*-benzoyl-*N*-benzyl-*N*-(3-chloropropyl)hydroxylamine (2q)**

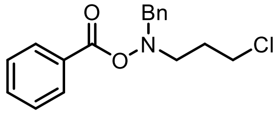

^{1}H NMR (600 MHz, $CDCl_3$) δ 7.85–7.81 (m, 2H), 7.45–7.42 (m, 1H), 7.34–7.28 (m, 4H), 7.23–7.18 (m, 2H), 7.18–7.13 (m, 1H), 4.11 (s, 2H), 3.61 (t, *J* = 6.3 Hz,

2H), 3.07 (t, J = 6.7 Hz, 2H), 1.97–1.89 (m, 2H). **^{13}C NMR** (151 MHz, $CDCl_3$) δ 165.3, 133.2, 130.2, 129.6, 129.4, 128.5, 128.5, 128.4, 127.9, 63.9, 55.2, 42.8, 30.1. **HRMS (ESI)** m/z calculated for $C_{17}H_{18}ClNO_2$ $[M+H]^+$ 304.1099, found 304.1103.

***O*-benzoyl-*N*-benzyl-*N*-isopropylhydroxylamine (2r)**

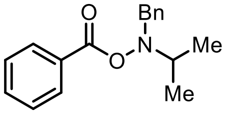

^{1}H NMR (400 MHz, $CDCl_3$) δ 7.98–7.91 (m, 2H), 7.60–7.51 (m, 1H), 7.51–7.39 (m, 4H), 7.35–7.21 (m, 3H), 4.21 (s, 2H), 3.45–3.31 (m, 1H), 1.30 (d, J = 6.5 Hz, 6H). **^{13}C NMR** (101 MHz, $CDCl_3$) δ 165.3, 136.4, 132.8, 129.5, 129.5, 129.4, 128.4, 128.3, 127.5, 59.3, 56.8, 18.8. **HRMS (ESI)** m/z calculated for $C_{17}H_{19}NO_2$ $[M+H]^+$ 270.1489, found 270.1489.

***O*-benzoyl-*N*-benzyl-*N*-(4-chlorobenzyl)hydroxylamine (2u)**

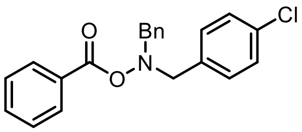

^{1}H NMR (400 MHz, $CDCl_3$) δ 7.90–7.83 (m, 2H), 7.59–7.52 (m, 1H), 7.49 (d, J = 7.4 Hz, 2H), 7.44–7.28 (m, 9H), 4.25 (s, 2H), 4.19 (s, 2H). **^{13}C NMR** (101 MHz, $CDCl_3$) δ 164.9, 135.6, 134.6, 133.5, 133.0, 130.6, 129.5, 129.3, 129.1, 128.6, 128.5, 128.4, 127.8, 62.4, 61.2. **HRMS (ESI)** m/z calculated for $C_{21}H_{18}ClNO_2$ $[M+H]^+$ 352.1099, found 352.1099.

***O*-benzoyl-*N*-benzyl-*N*-(4-(methylthio)benzyl)hydroxylamine (2v)**

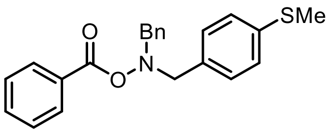

^{1}H NMR (600 MHz, $CDCl_3$) δ 7.77–7.73 (m, 2H), 7.44–7.38 (m, 1H), 7.37–7.32 (m, 2H), 7.30–7.25 (m, 4H), 7.25–7.19 (m, 2H), 7.18–7.14 (m, 1H), 7.13–7.08 (m, 2H), 4.10 (s, 2H), 4.07 (s, 2H), 2.35 (s, 3H). **^{13}C NMR** (151 MHz, $CDCl_3$) δ 164., 137.7, 135.9, 132.9, 132.7, 129.9, 129.4, 129.3, 129.3, 128.4, 128.4, 127.7, 126.4, 62.1, 61.6, 15.8. **HRMS (ESI)** m/z calculated for $C_{22}H_{21}NO_2S$ $[M+H]^+$ 364.1366, found 364.1368.

***O*-benzoyl-*N*-benzyl-*N*-(3-(4,4,5,5-tetramethyl-1,3,2-dioxaborolan-2-yl)benzyl)hydroxylamine (2x)**

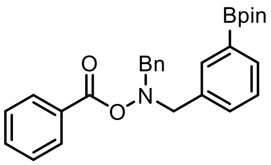

^{1}H NMR (400 MHz, $CDCl_3$) δ 7.93–7.86 (m, 2H), 7.83 (s, 1H), 7.79–7.72 (m, 1H), 7.69 (dt, J = 7.7, 1.6 Hz, 1H), 7.58–7.51 (m, 1H), 7.49–7.44 (m, 2H), 7.43–7.24 (m, 6H), 4.27 (s, 2H), 4.23 (s, 2H), 1.37 (s, 12H). **^{13}C NMR** (101 MHz, $CDCl_3$) δ 164.9, 135.9, 135., 134.2, 132.8, 132.7, 129.5, 129.4, 128.4, 128.3, 128.0, 127.7, 83.8, 62.1, 62.0, 24.9. **HRMS (ESI)** m/z calculated for $C_{27}H_{30}BNO_4$ $[M+H]^+$ 444.2341, found 444.2341.

***O*-benzoyl-*N*-benzyl-*N*-(3-chloro-4-fluorobenzyl)hydroxylamine (2y)**

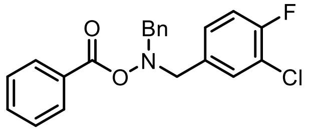

^{1}H NMR (400 MHz, $CDCl_3$) δ 8.23–8.18 (m, 1H), 7.91–7.84 (m, 2H), 7.59–7.48 (m, 4H), 7.44–7.32 (m, 5H), 7.09 (t, *J* = 8.7 Hz, 1H), 4.26 (s, 2H), 4.15 (s, 2H). **^{13}C NMR** (101 MHz, $CDCl_3$) δ 164.8, 162.4, 157.6 (d, *J* = 248.7 Hz), 135.4, 134.6, 133.3 (d, *J* = 3.9 Hz), 133.1, 131.3, 130.6, 129.5, 129.3, 129.0, 128.9, 128.5, 128.5, 127.9, 120.8 (d, *J* = 17.8 Hz), 116.5 (d, *J* = 21.1 Hz), 62.5, 60.7. **HRMS (ESI)** m/z calculated for $C_{21}H_{17}ClFNO_2$ $[M+H]^+$ 370.1005, found 370.1005.

(*R*)-*O*-benzoyl-*N*-methyl-*N*-(3-phenyl-3-(o-tolyloxy)propyl)hydroxylamine (2z)

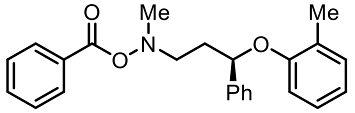

^{1}H NMR (600 MHz, $CDCl_3$) δ 7.94–7.88 (m, 2H), 7.50–7.44 (m, 1H), 7.37–7.31 (m, 2H), 7.26–7.23 (m, 2H), 7.22–7.19 (m, 2H), 7.16–7.11 (m, 1H), 7.04–7.00 (m, 1H), 6.88–6.82 (m, 1H), 6.70–6.65 (m, 1H), 6.56–6.52 (m, 1H), 5.30 (d, *J* = 7.2 Hz, 1H), 3.15–3.04 (m, 2H), 2.81 (s, 3H), 2.28–2.19 (m, 4H), 2.10–2.02 (m, 1H). **^{13}C NMR** (151 MHz, $CDCl_3$) δ 165.3, 155.9, 141.7, 133.2, 130.6, 129.5, 129.2, 128.7, 128.5, 127.6, 126.9, 126.7, 125.8, 120.3, 112.9, 57.6, 47.4, 36.4, 16.6. **HRMS (ESI)** m/z calculated for $C_{24}H_{25}NO_3$ $[M+H]^+$ 376.1907, found 376.1908.

(*S*)-*O*-benzoyl-*N*-methyl-*N*-(3-(naphthalen-1-yloxy)-3-(thiophen-2-yl)propyl)hydroxylamine (2aa)

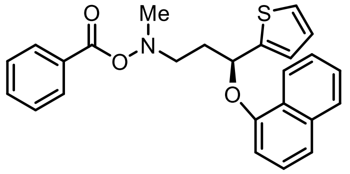

^{1}H NMR (600 MHz, $CDCl_3$) δ 8.28–8.21 (m, 1H), 7.91–7.87 (m, 2H), 7.68–7.61 (m, 1H), 7.44–7.40 (m, 1H), 7.38–7.33 (m, 2H), 7.31–7.26 (m, 2H), 7.25 (d, *J* = 8.2 Hz, 1H), 7.15–7.09 (m, 1H), 7.07–7.02 (m, 1H), 6.97–6.91 (m, 1H), 6.82–6.75 (m, 2H), 5.84 (t, *J* = 6.1 Hz, 1H), 3.17–3.05 (m, 2H), 2.76 (s, 3H), 2.49–2.40 (m, 1H), 2.26–2.17 (m, 1H). **^{13}C NMR** (151 MHz, $CDCl_3$) δ 165.7, 153.3, 144.9, 134.6, 133.3, 129.6, 129.2, 128.6, 127.6, 126.7, 126.4, 126.2, 125.9, 125.3, 124.9, 124.9, 122.2, 120.7, 107.3, 74.1, 57.4, 47.5, 36.5. **HRMS (ESI)** m/z calculated for $C_{25}H_{23}NO_3S$ $[M+H]^+$ 418.1472, found 418.1472.

***N*-(3-(9,10-ethanoanthracen-9(10H)-yl)propyl)-*O*-benzoyl-*N*-methylhydroxylamine (2ab)**

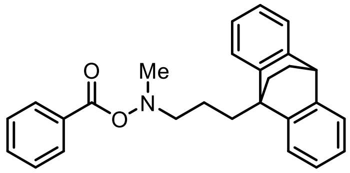

^{1}H NMR (600 MHz, $CDCl_3$) δ 8.01–7.95 (m, 2H), 7.52–7.46 (m, 1H), 7.41–7.34 (m, 2H), 7.16–7.12 (m, 4H), 6.98–6.94 (m, 2H), 6.93–6.89 (m, 2H), 4.16 (t, *J* = 2.8 Hz, 1H), 3.22 (s, 2H), 2.94 (s, 3H), 2.53–2.43 (m, 2H), 2.04 (t, *J* = 8.4 Hz, 2H), 1.73–1.67 (m, 2H), 1.49–1.43 (m, 2H). **^{13}C NMR** (151 MHz, $CDCl_3$) δ 165.3, 145.2, 144.9, 133.12, 129.5, 129.3, 128.5, 125.3, 125.2, 123.3, 121.3, 62.1, 47.4,

44.7, 44.5, 29.6, 28.7, 27.6, 22.6. **HRMS (ESI)** m/z calculated for $C_{27}H_{27}NO_2$ $[M+H]^+$ 398.2115, found 398.2116.

***O*-benzoyl-*N*-(3-(10,11-dihydro-5H-dibenzo[a,d][7]annulen-5-ylidene)propyl)-*N*-methylhydroxylamine (2ac)**

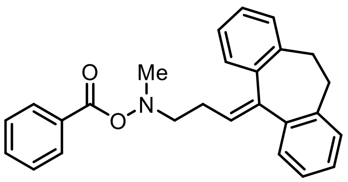

^{1}H NMR (600 MHz, $CDCl_3$) δ 7.82–7.77 (m, 2H), 7.49–7.43 (m, 1H), 7.33–7.27 (m, 2H), 7.19–7.14 (m, 1H), 7.07–7.01 (m, 4H), 7.01–6.97 (m, 2H), 6.95–6.91 (m, 1H), 5.84 (t, *J* = 7.5 Hz, 1H), 3.32–3.23 (m, 1H), 3.21–3.12 (m, 1H), 3.03–2.93 (m, 2H), 2.85 (d, *J* = 14.7 Hz, 1H), 2.78 (s, 3H), 2.67–2.59 (m, 1H), 2.44–2.36 (m, 2H). **^{13}C NMR** (151 MHz, $CDCl_3$) δ 165.2, 144.4, 141.0, 139.8, 139.3, 137.1, 133.0, 130.0, 129.4, 129.2, 128.6, 128.4, 128.1, 128.0, 128.0, 127.4, 127.1, 126.0, 125.8, 60.8, 46.9, 33.8, 32.0, 27.3. **HRMS (ESI)** m/z calculated for $C_{26}H_{25}NO_2$ $[M+H]^+$ 384.1958, found 384.1958.

4. Procedure for hydroamination of acyclic trisubstituted alkenes.

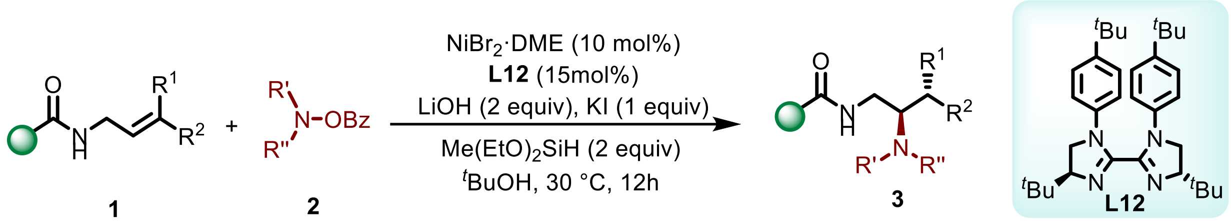

<u>**General procedure A**</u> **for NiH-catalyzed asymmetric Hydroamination of Acyclic Trisubstituted Alkenes.** In a nitrogen-filled glove box, to an oven-dried 4 mL screw-cap vial equipped with a magnetic stir bar was added $NiBr_2$·DME (6.2 mg, 10 mol%), **(*S*,*S*)-L12** (15.4 mg, 15 mol%), LiOH (9.6 mg, 0.40 mmol, 2.0 equiv), KI (33.2 mg, 0.20 mmol, 1.0 equiv), Olefin feedstock (0.20 mmol, 1.0 equiv), hydroxylamine ester (0.30 mmol, 1.5 equiv) and Anhydrous *tert*-butyl alcohol (1.0 mL, 0.20 M). The mixture was stirred for 10 min at room temperature, at which time $Me(EtO)_2SiH$ (80 µL, 0.50 mmol, 2.5 equiv) were added to the resulting mixture. The tube was sealed with a teflon-lined screw cap, removed from the glove box and the reaction was stirred at 30 °C water bath for up to 12 h (the mixture was stirred at 800 rpm). After the reaction was complete, the reaction was quenched upon the addition of H_2O, and the mixture was extracted with Et_2O. The product was purified by flash column chromatography (petroleum ether/EtOAc) for each substrate. The yields reported are the average of at least two

experiments, unless otherwise indicated. The enantiomeric excesses (% ee) were determined by HPLC analysis using chiral stationary phases.

□ <> 结果视图 - 峰表

峰表 化合物 组 校准曲线

峰号	保留时间	面积	高度	标记	浓度	浓度单位	化合物ID号	化合物名	面积%
	Retention	Area	Height	Mark	Concentration				peak area %

***N*-((2*R*,3*R*)-3-methyl-2-morpholino-5-phenylpentyl)benzamide (3a)**

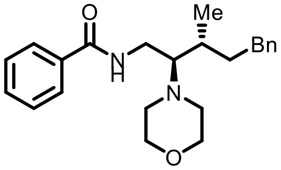

From (*E*)-*N*-(3-methyl-5-phenylpent-2-en-1-yl)benzamide (**1a**) (55.8 mg, 0.20 mmol, 1.0 equiv) and morpholino benzoate (**2a**) (62.1 mg, 0.30 mmol, 1.5 equiv), the title compound was prepared following the general procedure **A** using $NiBr_2$•DME (6.2 mg, 10 mol%), **(*S*,*S*)-L12** (15.4 mg, 15 mol%), LiOH (9.6 mg, 0.40 mmol, 2.0 equiv), KI (33.2 mg, 0.20 mmol, 1.0 equiv), $Me(EtO)_2SiH$ (80 µL, 0.50 mmol, 2.5 equiv), Anhydrous *tert*-butyl alcohol (1.0 mL, 0.20 M). The reaction mixture was stirred for 12 h at 30 °C. The crude material was purified by flash column chromatography (petroleum ether/EtOAc = 3:1) to provide the title compound as a yellow oil in 90% yield (65.9 mg).

^{1}H NMR (400 MHz, $CDCl_3$) δ 7.73–7.63 (m, 2H), 7.46–7.40 (m, 1H), 7.39–7.33 (m, 2H), 7.24–7.17 (m, 2H), 7.14–7.07 (m, 3H), 6.83 (s, 1H), 3.68-3.53 (m, 5H), 3.28-3.16(m, 1H), 2.72-2.58 (m, 3H), 2.56–2.42 (m, 4H), 1.90–1.80 (m, 1H), 1.77–1.66 (m, 1H), 1.58–1.46 (m, 1H), 0.95 (d, J = 6.8 Hz, 3H); **^{13}C NMR** (101 MHz, $CDCl_3$) δ 167.1, 142.1, 134.8, 131.4, 128.7, 128.4, 128.4, 126.8, 125.9, 67.9, 67.1, 49.6, 37.4, 36.9, 33.4, 31.1, 16.5. **HRMS** (ESI) m/z calculated for $C_{23}H_{30}N_2O_2$ $[M+H]^+$ 365.2588, found: 365.2589. **Optical rotation**: $[\alpha]^{20}_D$ = -51.8 (c = 1.0 g/L, $CHCl_3$). The absolute configuration was assigned by analogy to that of **4b**. **HPLC condition**: Chiral column AD-H, n-hexane/i-PrOH = 95:5, flow rate = 1 mL/min, wavelength = 254 nm, tR = 41.42 min for major isomer, tR = 36.83 min for minor isomer.

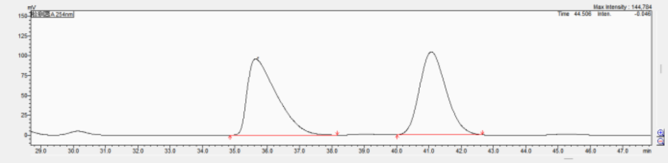

峰号	保留时间	面积	高度	标记	浓度	浓度单位	化合物ID号	化合物名	面积%
1	35.640	6045308	96260	M	50.358				50.358
2	41.076	5959338	104530	M	49.642				49.642
总计		12004646	200790		100.000				100.000

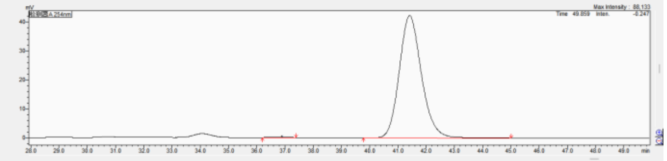

峰号	保留时间	面积	高度	标记	浓度	浓度单位	化合物ID号	化合物名	面积%
1	36.826	7854	198	M	0.339				0.339
2	41.418	2309995	42327	M	99.661				99.661
总计		2317849	42525		100.000				100.000

***N*-((2*R*,3*R*)-3-methyl-2-morpholino-5-phenylpentyl)benzamide (3b)**

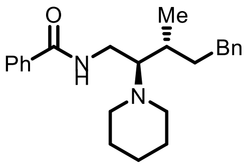

From (*E*)-*N*-(3-methyl-5-phenylpent-2-en-1-yl)benzamide (**1a**) (55.8 mg, 0.20 mmol, 1.0 equiv) and morpholino benzoate (**2b**) (62.1 mg, 0.30 mmol, 1.5 equiv), the title compound was prepared following the general procedure **A** using $NiBr_2$·DME (6.2 mg, 10 mol%), ***(S,S)*-L12** (15.4 mg, 15 mol%), LiOH (9.6 mg, 0.40 mmol, 2.0 equiv), KI (33.2 mg, 0.20 mmol, 1.0 equiv), $Me(EtO)_2SiH$ (80 μL, 0.50 mmol, 2.5 equiv), Anhydrous *tert*-butyl alcohol (1.0 mL, 0.20 M). The reaction mixture was stirred for 12 h at 30 °C. The crude material was purified by flash column chromatography (petroleum ether/EtOAc = 3:1) to provide the title compound as a yellow oil in 88% yield (64.5 mg).

^{1}H NMR (400 MHz, $CDCl_3$) δ 7.85 (d, J = 7.4 Hz, 2H), 7.56–7.48 (m, 3H), 7.37–7.30 (m, 3H), 7.27–7.23 (m, 3H), 3.82–3.71 (m, 1H), 3.28–3.18 (m, 1H), 2.83–2.73 (m, 3H), 2.69–2.61 (m, 2H), 2.59–2.53 (m, 2H), 2.01–1.96 (m, 1H), 1.87–1.81 (m, 1H), 1.71–1.57 (m, 5H), 1.07 (d, J = 6.4 Hz, 3H); **^{13}C NMR** (101 MHz, $CDCl_3$) δ 167.0, 142.4, 134.9, 131.3, 128.6, 128.4, 126.9, 125.8, 67.4, 50.2, 37.7, 37.1, 33.3, 31.3, 27.3, 24.9, 16.7. **HRMS** (ESI) m/z calculated for $C_{24}H_{32}N_2O$ $[M+H]^+$ 365.2588, found: 365.2589. **Optical rotation**: $[\alpha]^{20}_D$ = -95.3 (c = 1.0 g/L, $CHCl_3$). The absolute configuration was assigned by analogy to that of **4b**. **HPLC condition**: Chiral column AD-H, n-hexane/i-PrOH = 97:3, flow rate = 0.8 mL/min, wavelength = 254 nm, tR = 38.2 min for major isomer, tR = 36.2 min for minor isomer.

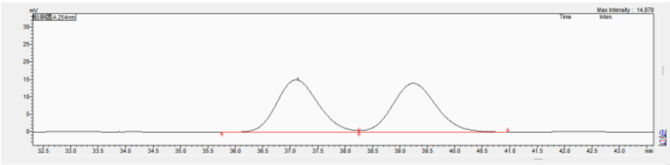

峰号	保留时间	面积	高度	标记	浓度	浓度单位	化合物ID号	化合物名	面积%
1	37.103	781920	15015	M	50.380				50.380
2	39.240	770140	13999	V M	49.620				49.620
总计		1552061	29013		100.000				100.000

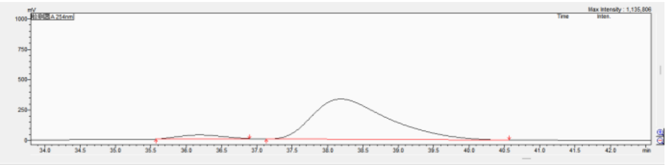

峰号	保留时间	面积	高度	标记	浓度	浓度单位	化合物ID号	化合物名	面积%
1	36.190	1436191	33038	M	5.434				5.434
2	38.188	24995865	332494	M	94.566				94.566
总计		26432055	365532		100.000				100.000

Ethyl 1-((2*R*,3*R*)-1-benzamido-3-methyl-5-phenylpentan-2-yl)piperidine-4-carboxylate (3c)

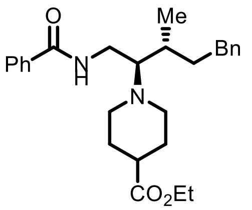

From (*E*)-*N*-(3-methyl-5-phenylpent-2-en-1-yl)benzamide (**1a**) (55.8 mg, 0.20 mmol, 1.0 equiv) and Ethyl 1-(benzoyloxy)piperidine-4-carboxylate (**2c**) (83.1 mg, 0.30 mmol, 1.5 equiv), the title compound was prepared following the general procedure **A** using $NiBr_2$·DME (6.2 mg, 10 mol%), ***(S,S)*-L12** (15.4 mg, 15 mol%), LiOH (9.6 mg, 0.40 mmol, 2.0 equiv), KI (33.2 mg, 0.20 mmol, 1.0 equiv), $Me(EtO)_2SiH$ (80 μL, 0.50 mmol, 2.5 equiv), Anhydrous tert-butyl alcohol (1.0 mL, 0.20 M). The reaction mixture was stirred for 12 h at 30 °C. The crude material was purified by flash column chromatography (petroleum ether/EtOAc = 5:1) to provide the title compound as a yellow oil in 64% yield (55.8 mg).

^{1}H NMR (400 MHz, $CDCl_3$) δ 7.80 (d, *J* = 6.7 Hz, 2H), 7.54–7.46 (m, 3H), 7.31 (d, *J* = 7.5 Hz, 2H), 7.24–7.20 (m, 3H), 6.99 (s, 1H), 4.23–4.13 (m, 2H), 3.81–3.73 (m, 1H), 3.29–3.21 (m, 1H), 2.97 (d, *J* = 10.4 Hz, 1H), 2.83–2.78 (m, 1H), 2.75–2.69 (m, 1H), 2.67–2.57 (m, 3H), 2.42–2.30 (m, 2H), 1.97–1.92 (m, 2H), 1.86–1.74 (m, 3H), 1.68–1.57 (m, 2H), 1.29 (t, *J* = 5.8 Hz, 3H), 1.05 (d, *J* = 6.3 Hz, 3H); **^{13}C NMR** (101 MHz, $CDCl_3$) δ 175.1, 167.1, 142.2, 134.8, 131.4, 128.7, 128.4, 128.4, 126.8, 125.9, 67.2, 60.4, 51.1, 46.3, 41.6, 37.6, 37.1, 33.3, 31.2, 16.7, 14.3. **HRMS** (ESI) m/z calculated for $C_{27}H_{36}N_2O_3$ $[M+H]^+$ 437.2799, found: 437.2793. **Optical rotation**: $[\alpha]^{20}_{D}$ = -10.1 (c = 1.0 g/L, $CHCl_3$). The absolute configuration was assigned by analogy to that of **3ab**. **HPLC condition**: Chiral column AD-H, n-hexane/i-PrOH = 95:5, flow rate = 1 mL/min, wavelength = 254 nm, tR = 37.33 min for major isomer, tR = 34.01 min for minor isomer.

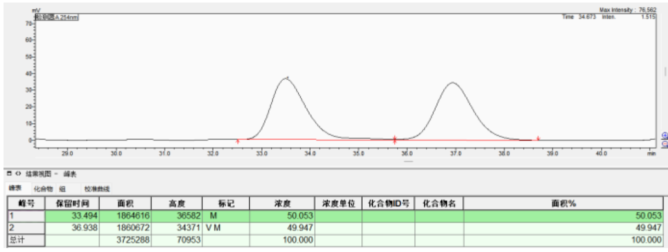

峰号	保留时间	面积	高度	标记	浓度	浓度单位	化合物ID号	化合物名	面积%
1	33.494	1864616	36582	M	50.053				50.053
2	36.938	1860672	34371	V M	49.947				49.947
总计		3725288	70953		100.000				100.000

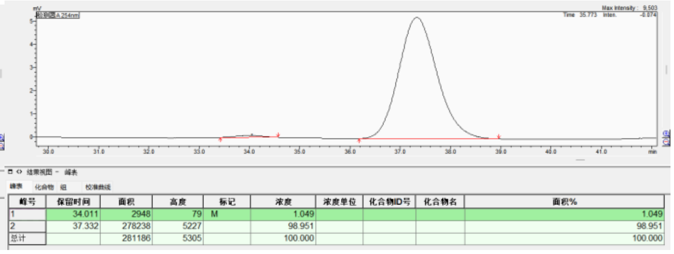

峰号	保留时间	面积	高度	标记	浓度	浓度单位	化合物ID号	化合物名	面积%
1	34.011	2948	79	M	1.049				1.049
2	37.332	278238	5227		98.951				98.951
总计		281186	5305		100.000				100.000

N-((2*R*,3*R*)-3-methyl-5-phenyl-2-(1,4-dioxa-8-azaspiro[4.5]decan-8-yl)pentyl)benzamide (3d)

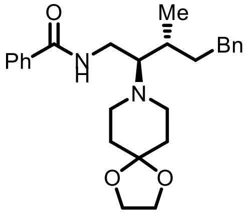

From (*E*)-*N*-(3-methyl-5-phenylpent-2-en-1-yl)benzamide (**1a**) (55.8 mg, 0.20 mmol, 1.0 equiv) and 1,4-dioxa-8-azaspiro[4.5]decan-8-yl benzoate (**2d**) (84.2 mg, 0.30 mmol, 1.5 equiv), the title compound was prepared following the general procedure **A** using $NiBr_2 \cdot DME$ (6.2 mg, 10 mol%), **(*S*,*S*)-L12** (15.4 mg, 15 mol%), LiOH (9.6 mg, 0.40 mmol, 2.0 equiv), KI (33.2 mg, 0.20 mmol, 1.0 equiv), $Me(EtO)_2SiH$ (80 μL, 0.50 mmol, 2.5 equiv), Anhydrous *tert*-butyl alcohol (1.0 mL, 0.20 M). The reaction mixture was stirred for 12 h at 30 °C. The crude material was purified by flash column chromatography (petroleum ether/EtOAc = 5:1) to provide the title compound as a yellow oil in 77% yield (65.0 mg).

^{1}H NMR (400 MHz, $CDCl_3$) δ 7.75 (d, *J* = 7.1 Hz, 2H), 7.51–7.47 (m, 1H), 7.46–7.41 (m, 2H), 7.30–7.26 (m, 2H), 7.20–7.16 (m, 3H), 6.97 (s, 1H), 3.96 (s, 4H), 3.76–3.68 (m, 1H), 3.27–3.18 (m, 1H), 2.89–2.82 (m, 2H), 2.70–2.56 (m, 5H), 1.97–1.90 (m, 1H), 1.78–1.69 (m, 5H), 1.61–1.53 (m, 1H), 1.02 (d, *J* = 6.9 Hz, 3H); **^{13}C NMR** (101 MHz, $CDCl_3$) δ 167.1, 142.3, 134.8, 131.3, 128.6, 128.4, 128.4, 126.8, 125.8, 107.3, 66.6, 64.3, 46.8, 37.7, 37.0, 36.1, 33.4, 31.2, 16.7. **HRMS** (ESI) m/z calculated for $C_{26}H_{34}N_2O_3$ $[M+H]^+$ 423.2642, found: 432.2644. **Optical rotation**: $[\alpha]^{20}_{D}$ = -91.8 (c = 1.0 g/L, $CHCl_3$). The absolute

configuration was assigned by analogy to that of **4b**. **HPLC condition**: Chiral column AD-H, n-hexane/i-PrOH = 95:5, flow rate = 1 mL/min, wavelength = 254 nm, tR = 35.78 min for major isomer, tR = 31.75 min for minor isomer.

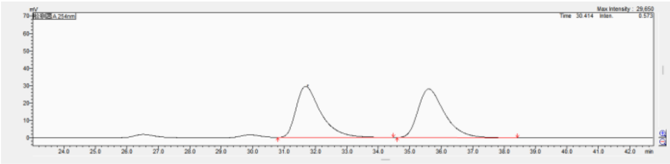

峰号	保留时间	面积	高度	标记	浓度	浓度单位	化合物ID号	化合物名	面积%
1	31.689	1612666	29499	M	49.609				49.609
2	35.601	1638064	28009	M	50.391				50.391
总计		3250730	57508		100.000				100.000

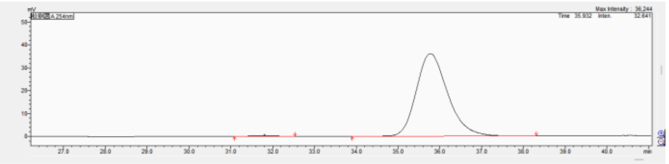

峰号	保留时间	面积	高度	标记	浓度	浓度单位	化合物ID号	化合物名	面积%
1	31.752	10103	239	M	0.535				0.535
2	35.775	1879874	36109	M	99.465				99.465
总计		1889977	36348		100.000				100.000

***tert*-Butyl (1-((2*R*,3*R*)-1-benzamido-3-methyl-5-phenylpentan-2-yl)piperidin-4-yl)carbamate (3e)**

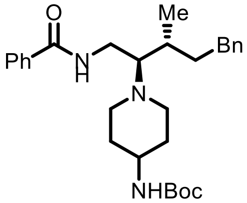

From (*E*)-N-(3-methyl-5-phenylpent-2-en-1-yl)benzamide (**1a**) (55.8 mg, 0.20 mmol, 1.0 equiv) and 4-((*tert*-butoxycarbonyl)amino)piperidin-1-yl benzoate (**2e**) (96.1 mg, 0.30 mmol, 1.5 equiv), the title compound was prepared following the general procedure **A** using $NiBr_2$·DME (6.2 mg, 10 mol%), **(*S*,*S*)-L12** (15.4 mg, 15 mol%), LiOH (9.6 mg, 0.40 mmol, 2.0 equiv), KI (33.2 mg, 0.20 mmol, 1.0 equiv), $Me(EtO)_2SiH$ (80 μL, 0.50 mmol, 2.5 equiv), Anhydrous *tert*-butyl alcohol (1.0 mL, 0.20 M). The reaction mixture was stirred for 12 h at 30 °C. The crude material was purified by flash column chromatography (petroleum ether/EtOAc = 1:1) to provide the title compound as a yellow oil in 76% yield (72.9 mg).

^{1}H NMR (400 MHz, $CDCl_3$) δ 7.75 (d, *J* = 7.6 Hz, 2H), 7.53–7.48 (m, 1H), 7.47–7.42 (m, 2H), 7.30–7.26 (m, 2H), 7.20–7.16 (m, 3H), 6.89 (s, 1H), 4.46 (s, 1H), 3.75–3.64 (m, 1H), 3.53–3.37 (m, 1H), 3.27–3.13 (m, 1H), 2.86 (d, *J* = 11.4 Hz, 1H), 2.75–2.65 (m, 3H), 2.62–2.51 (m, 2H), 2.47–2.32 (m, 1H), 1.98–1.91 (m, 2H), 1.91–1.80 (m, 2H), 1.79–1.72 (m, 1H), 1.61–1.52 (m, 1H), 1.44 (s, 10H), 1.00 (d, *J* = 6.8 Hz, 3H); **^{13}C NMR** (101 MHz, $CDCl_3$) δ 167.1, 155.2, 142.2, 134.8, 131.4, 128.7, 128.5, 128.5, 128.4, 126.8, 125.9, 79.4, 66.9, 50.2, 45.7, 37.6, 37.3, 33.3, 31.3, 29.7, 28.5, 16.7. **HRMS** (ESI) m/z calculated for $C_{29}H_{41}N_3O_3$ $[M+Na]^+$ 502.3040, found: 502.3040. **Optical rotation**: $[\alpha]^{20}_D$ = -114 (c = 1.0 g/L, $CHCl_3$). The absolute configuration was assigned by analogy to that of **4b**. **HPLC condition**: Chiral column OD-H, n-hexane/i-PrOH = 95:5, flow rate = 1 mL/min, wavelength = 254 nm, tR = 41.33 min for major isomer, tR = 37.44 min for minor isomer.

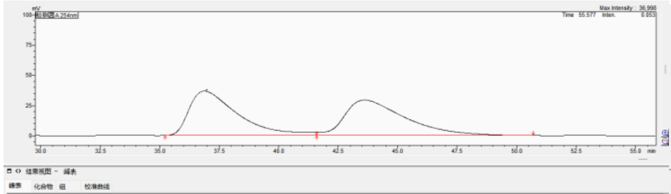

峰号	保留时间	面积	高度	标记	浓度	浓度单位	化合物ID号	化合物名	面积%
1	36.878	5236514	36727	M	50.125				50.125
2	43.611	5210491	29298	V M	49.875				49.875
总计		10447005	66025		100.000				100.000

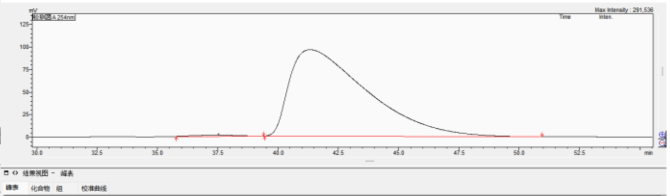

峰号	保留时间	面积	高度	标记	浓度	浓度单位	化合物ID号	化合物名	面积%
1	37.436	148531	1290	M	0.689				0.689
2	41.330	21405989	95877	M	99.311				99.311
总计		21554521	97166		100.000				100.000

tert-Butyl 4-((2*R*,3*R*)-1-benzamido-3-methyl-5-phenylpentan-2-yl)piperazine-1-carboxylate (3f)

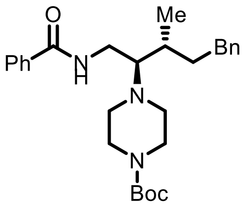

From (*E*)-*N*-(3-methyl-5-phenylpent-2-en-1-yl)benzamide (**1a**) (55.8 mg, 0.20 mmol, 1.0 equiv) and *tert*-butyl 4-(benzoyloxy)piperazine-1-carboxylate (**2f**) (91.8 mg, 0.30 mmol, 1.5 equiv), the title compound was prepared following the general procedure **A** using $NiBr_2$·DME (6.2 mg, 10 mol%), **(*S*,*S*)-L12** (15.4 mg, 15 mol%), LiOH (9.6 mg, 0.40 mmol, 2.0 equiv), KI (33.2 mg, 0.20 mmol, 1.0 equiv), $Me(EtO)_2SiH$ (80 µL, 0.50 mmol, 2.5 equiv), Anhydrous *tert*-butyl alcohol (1.0 mL, 0.20 M). The reaction mixture was stirred for 12 h at 30 °C. The crude material was purified by flash column chromatography (petroleum ether/EtOAc = 2:1) to provide the title compound as a yellow oil in 70% yield (65.1 mg).

^{1}H NMR (400 MHz, $CDCl_3$) δ 7.76–7.73 (m, 2H), 7.51–7.48 (m, 1H), 7.46–7.42 (m, 2H), 7.30–7.26 (m, 2H), 7.21–7.16 (m, 3H), 6.88–6.83 (m, 1H), 3.74–3.67 (m, 1H), 3.45–3.38 (m, 3H), 3.31–3.24 (m, 1H), 2.73–2.65 (m, 3H), 2.65–2.57 (m, 2H), 2.54–2.48 (m, 2H), 1.92–1.85 (m, 1H), 1.83–1.73 (m, 2H), 1.61–1.54 (m, 1H), 1.46 (s, 9H), 1.02 (d, *J* = 6.8 Hz, 3H); **^{13}C NMR** (101 MHz, $CDCl_3$) δ 167.1, 154.8, 142.1, 134.7, 131.4, 128.7, 128.5, 128.4, 126.8, 125.9, 79.9, 67.1, 48.9, 37.4, 37.0, 33.3, 31.2, 29.3, 28.5, 16.6. **HRMS** (ESI) m/z calculated for $C_{28}H_{39}N_3O_3$ $[M+Na]^+$ 488.2883, found: 488.2884. **Optical rotation**: $[\alpha]^{20}_D$ = -127 (c = 1.0 g/L, $CHCl_3$). The absolute configuration was assigned by analogy to that of **4b**. **HPLC condition**: Chiral column AD-H, n-hexane/i-PrOH = 90:10, flow rate = 1 mL/min, wavelength = 254 nm, tR = 34.58 min for major isomer, tR = 18.70 min for minor isomer.

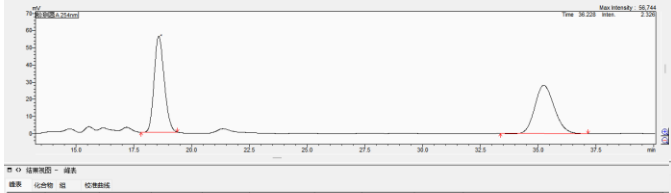

峰号	保留时间	面积	高度	标记	浓度	浓度单位	化合物ID号	化合物名	面积%
1	18.584	1697922	55881	M	50.802				50.802
2	35.245	1644299	28138	M	49.198				49.198
总计		3342221	84019		100.000				100.000

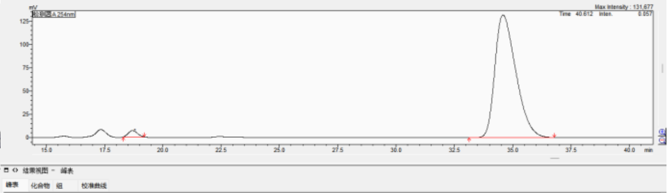

峰号	保留时间	面积	高度	标记	浓度	浓度单位	化合物ID号	化合物名	面积%
1	18.699	180318	6677	M	2.144				2.144
2	34.584	8228270	131575	M	97.856				97.856
总计		8408588	138252		100.000				100.000

tert-Butyl 4-((2_R_,3_R_)-1-benzamido-3-methyl-5-phenylpentan-2-yl)-1,4-diazepane-1-carboxylate (3g)

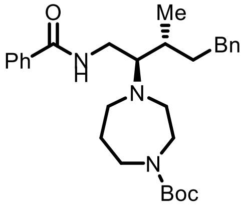

From (_E_)-_N_-(3-methyl-5-phenylpent-2-en-1-yl)benzamide (**1a**) (55.8 mg, 0.20 mmol, 1.0 equiv) and _tert_-butyl 4-(benzoyloxy)-1,4-diazepane-1-carboxylate (**2g**) (96.1 mg, 0.30 mmol, 1.5 equiv), the title compound was prepared following the general procedure **A** using $NiBr_2$·DME (6.2 mg, 10 mol%), **(_S_,_S_)-L12** (15.4 mg, 15 mol%), LiOH (9.6 mg, 0.40 mmol, 2.0 equiv), KI (33.2 mg, 0.20 mmol, 1.0 equiv), $Me(EtO)_2SiH$ (80 µL, 0.50 mmol, 2.5 equiv), Anhydrous _tert_-butyl alcohol (1.0 mL, 0.20 M). The reaction mixture was stirred for 12 h at 30 °C. The crude material was purified by flash column chromatography (petroleum ether/EtOAc = 2:1) to provide the title compound as a yellow oil in 62% yield (59.4 mg).

^{1}H NMR (400 MHz, $CDCl_3$) δ 7.83–7.78 (m, 2H), 7.53–7.46 (m, 3H), 7.34–7.29 (m, 2H), 7.24–7.20 (m, 3H), 6.87 (s, 1H), 3.84 (d, _J_ = 6.5 Hz, 1H), 3.48–3.39 (m, 3H), 3.17–3.04 (m, 1H), 2.98–2.88 (m, 2H), 2.80–2.72 (m, 2H), 2.72–2.65 (m, 2H), 2.60–2.52 (m, 1H), 1.93–1.82 (m, 2H), 1.81–1.67 (m, 3H), 1.57–1.47 (m, 2H), 1.44 (d, _J_ = 12.7 Hz, 9H), 1.06 (d, _J_ = 6.5 Hz, 3H). **^{13}C NMR** (101 MHz, $CDCl_3$) δ 167.0, 155.5, 142.3, 134.5, 131.4, 128.7, 128.4, 128.4, 126.8, 125.9, 79.4, 69.9, 52.5, 51.1, 49.3, 48.8, 45.6, 39.1, 36.8, 33.3, 30.3, 28.5, 16.7. **HRMS** (ESI) m/z calculated for $C_{29}H_{41}N_3O_3$ $[M+H]^+$ 480.3221, found: 480.3221. **Optical rotation**: $[\alpha]^{20}_D$ = -254 (c = 1.0 g/L, $CHCl_3$). The absolute configuration was assigned by analogy to that of **4b**. **HPLC condition**: Chiral column AD-H, n-hexane/i-PrOH = 90:10, flow rate = 1 mL/min, wavelength = 254 nm, tR = 22.04 min for major isomer, tR = 15.38 min for minor isomer.

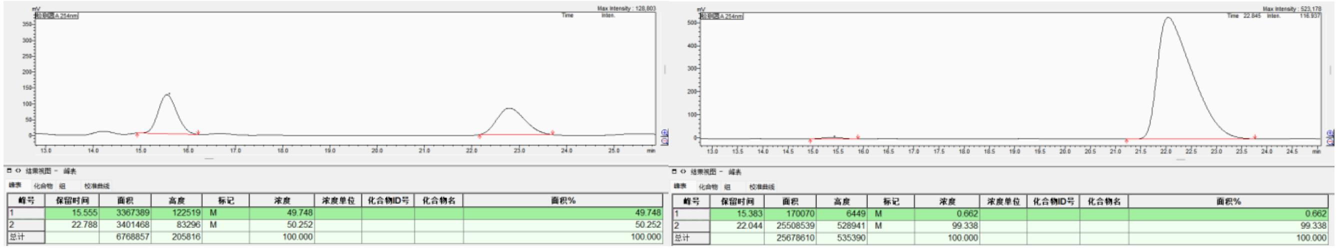

峰号	保留时间	面积	高度	标记	浓度	浓度单位	化合物ID号	化合物名	面积%
1	15.555	3367389	122519	M	49.748				49.748
2	22.788	3401468	83296	M	50.252				50.252
总计		6768857	205816		100.000				100.000

峰号	保留时间	面积	高度	标记	浓度	浓度单位	化合物ID号	化合物名	面积%
1	15.383	170070	6449	M	0.662				0.662
2	22.044	25508539	528941	M	99.338				99.338
总计		25678610	535390		100.000				100.000

_N_-((2_R_,3_R_)-3-methyl-2-(4-(methylsulfonyl)piperazin-1-yl)-5-phenylpentyl)benzamide (3h)

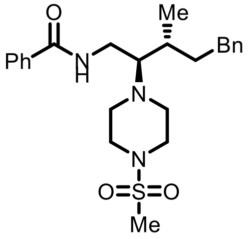

From (_E_)-_N_-(3-methyl-5-phenylpent-2-en-1-yl)benzamide (**1a**) (55.8 mg, 0.20 mmol, 1.0 equiv) and 4-(methylsulfonyl)piperazin-1-yl benzoate (**2h**) (85.2 mg, 0.30 mmol, 1.5 equiv), the title compound was prepared following the general procedure **A** using $NiBr_2$·DME (6.2 mg, 10 mol%), **(_S_,_S_)-L12** (15.4 mg, 15 mol%), LiOH (9.6 mg, 0.40 mmol, 2.0 equiv), KI (33.2 mg, 0.20 mmol, 1.0 equiv), $Me(EtO)_2SiH$ (80

μL, 0.50 mmol, 2.5 equiv), Anhydrous *tert*-butyl alcohol (1.0 mL, 0.20 M). The reaction mixture was stirred for 12 h at 30 °C. The crude material was purified by flash column chromatography (petroleum ether/EtOAc = 3:1) to provide the title compound as a yellow oil in 73% yield (64.9 mg).

^{1}H NMR (400 MHz, $CDCl_3$) δ 7.76–7.69 (m, 2H), 7.55–7.48 (m, 1H), 7.47–7.42 (m, 2H), 7.30–7.26 (m, 2H), 7.22–7.15 (m, 3H), 6.58 (s, 1H), 3.74–3.64 (m, 1H), 3.38–3.30 (m, 1H), 3.23–3.12 (m, 4H), 2.85–2.79 (m, 2H), 2.77 (s, 3H), 2.72–2.63 (m, 4H), 2.61–2.52 (m, 1H), 1.86–1.77 (m, 2H), 1.63–1.52 (m, 1H), 1.04 (d, *J* = 6.6 Hz, 3H); **^{13}C NMR** (101 MHz, $CDCl_3$) δ 167.3, 142.1, 134.7, 131.6, 128.7, 128.5, 128.4, 126.8, 126.0, 67.3, 48.6, 46.7, 37.3, 37.1, 34.7, 33.3, 31.4, 16.6. **HRMS** (ESI) m/z calculated for $C_{24}H_{33}N_3O_3S$ $[M+H]^+$ 444.2316, found: 444.2312. **Optical rotation**: $[\alpha]^{20}_D$ = -13.1 (c = 1.0 g/L, $CHCl_3$). The absolute configuration was assigned by analogy to that of **4b**. **HPLC condition**: Chiral column OD-H, n-hexane/i-PrOH = 85:15, flow rate = 1 mL/min, wavelength = 254 nm, tR = 67.41 min for major isomer, tR = 58.34 min for minor isomer.

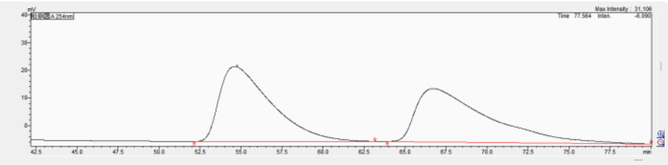

峰号	保留时间	面积	高度	标记	浓度	浓度单位	化合物ID号	化合物名	面积%
1	54.606	5956614	27202		50.314				50.314
2	66.708	5882326	19300		49.686				49.686
总计		11838940	46503		100.000				100.000

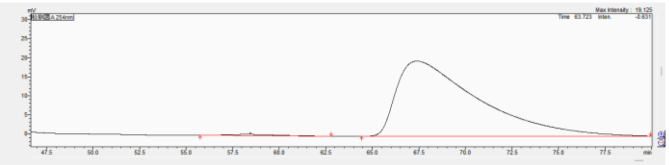

峰号	保留时间	面积	高度	标记	浓度	浓度单位	化合物ID号	化合物名	面积%
1	58.336	66111	345	M	1.082				1.082
2	67.407	6042598	19751	M	98.918				98.918
总计		6108709	20096		100.000				100.000

***N*-((2*R*,3*R*)-3-methyl-5-phenyl-2-(4-(pyrimidin-2-yl)piperazin-1-yl)pentyl)benzamide (3i)**

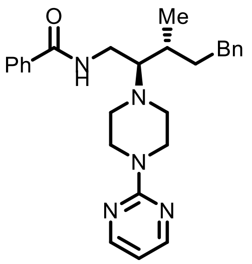

From (*E*)-*N*-(3-methyl-5-phenylpent-2-en-1-yl)benzamide (**1a**) (55.8 mg, 0.20 mmol, 1.0 equiv) and 4-(pyrimidin-2-yl)piperazin-1-yl benzoate (**2i**) (85.2 mg, 0.30 mmol, 1.5 equiv), the title compound was prepared following the general procedure **A** using $NiBr_2$·DME (6.2 mg, 10 mol%), ***(S,S)*-L12** (15.4 mg, 15 mol%), LiOH (9.6 mg, 0.40 mmol, 2.0 equiv), KI (33.2 mg, 0.20 mmol, 1.0 equiv), $Me(EtO)_2SiH$ (80 μL, 0.50 mmol, 2.5 equiv), Anhydrous tert-butyl alcohol (1.0 mL, 0.20 M). The reaction mixture was stirred for 12 h at 30 °C. The crude material was purified by flash column chromatography (petroleum ether/EtOAc = 3:1) to provide the title compound as a yellow oil in 65% yield (57.6 mg).

^{1}H NMR (400 MHz, $CDCl_3$) δ 8.39 (d, *J* = 4.7 Hz, 2H), 7.85 (d, *J* = 7.4 Hz, 2H), 7.59–7.54 (m, 1H), 7.53–7.49 (m, 2H), 7.36–7.32 (m, 2H), 7.27–7.22 (m, 3H), 7.10 (s, 1H), 6.61–6.54 (m, 1H), 3.95–3.80 (m, 5H), 3.44–3.36 (m, 1H), 2.96–2.87 (m, 2H), 2.81–2.62 (m, 5H), 2.05–1.96 (m, 1H), 1.94–1.83 (m, 1H),

1.74–1.63 (m, 1H), 1.12 (d, J = 6.8 Hz, 3H); **^{13}C NMR** (101 MHz, $CDCl_3$) δ 167.2, 161.7, 157.8, 142.1, 134.7, 131.4, 128.7, 128.4, 128.4, 126.8, 125.9, 110.1, 67.1, 49.0, 44.7, 37.5, 37.0, 33.3, 31.1, 16.6. **HRMS** (ESI) m/z calculated for $C_{27}H_{33}N_5O$ $[M+H]^+$ 444.2758, found: 444.2758. **Optical rotation**: $[\alpha]^{20}_D$ = -21.7 (c = 1.0 g/L, $CHCl_3$). The absolute configuration was assigned by analogy to that of **4b**. **HPLC condition**: Chiral column OD-H, n-hexane/i-PrOH = 90:10, flow rate = 1 mL/min, wavelength = 254 nm, tR = 33.86 min for major isomer, tR = 29.92 min for minor isomer.

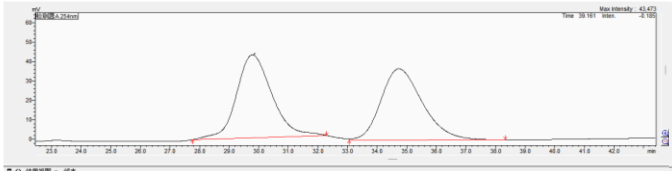

峰号	保留时间	面积	高度	标记	浓度	浓度单位	化合物ID号	化合物名	面积%
1	29.784	3546912	42678	M	49.727				49.727
2	34.734	3585831	36718	M	50.273				50.273
总计		7132743	79396		100.000				100.000

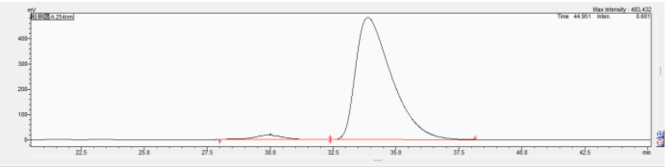

峰号	保留时间	面积	高度	标记	浓度	浓度单位	化合物ID号	化合物名	面积%
1	29.923	1436534	17131	M	2.919				2.919
2	33.883	47768989	481490	M	97.081				97.081
总计		49205524	498621		100.000				100.000

N-((2*R*,3*R*)-2-(diethylamino)-3-methyl-5-phenylpentyl)benzamide (3j)

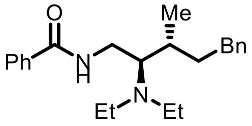

From (*E*)-*N*-(3-methyl-5-phenylpent-2-en-1-yl)benzamide (**1a**) (55.8 mg, 0.20 mmol, 1.0 equiv) and *O*-benzoyl-*N*,*N*-diethylhydroxylamine (**2j**) (57.9 mg, 0.30 mmol, 1.5 equiv), the title compound was prepared following the general procedure **A** using $NiBr_2$·DME (6.2 mg, 10 mol%), **(*S*,*S*)-L12** (15.4 mg, 15 mol%), LiOH (9.6 mg, 0.40 mmol, 2.0 equiv), KI (33.2 mg, 0.20 mmol, 1.0 equiv), $Me(EtO)_2SiH$ (80 μL, 0.50 mmol, 2.5 equiv), Anhydrous *tert*-butyl alcohol (1.0 mL, 0.20 M). The reaction mixture was stirred for 12 h at 30 °C. The crude material was purified by flash column chromatography (petroleum ether/EtOAc = 3:1) to provide the title compound as a yellow oil in 77% yield (54.2 mg).

^{1}H NMR (400 MHz, $CDCl_3$) δ 7.85 (d, J = 7.1 Hz, 2H), 7.54 (d, J = 7.0 Hz, 1H), 7.52–7.48 (m, 2H), 7.38–7.31 (m, 3H), 7.26–7.23 (m, 2H), 7.19 (s, 1H), 3.89–3.82 (m, 1H), 3.18–3.01 (m, 1H), 2.84–2.74 (m, 4H), 2.65–2.58 (m, 3H), 1.93–1.86 (m, 2H), 1.61–1.53 (m, 1H), 1.14–1.08 (m, 9H); **^{13}C NMR** (101 MHz, $CDCl_3$) δ 166.9, 142.4, 134.9, 131.2, 128.6, 128.4, 128.4, 126.8, 125.8, 63.6, 44.4, 38.3, 37.3, 33.5, 32.9, 16.6, 15.9. **HRMS** (ESI) m/z calculated for $C_{23}H_{32}N_2O$ $[M+H]^+$ 353.2588, found: 353.2587. **Optical rotation**: $[\alpha]^{20}_D$ = -252 (c = 1.0 g/L, $CHCl_3$). The absolute configuration was assigned by analogy to that of **4b**. **HPLC condition**: Chiral column AD-H, n-hexane/i-PrOH = 95:5, flow rate = 1 mL/min, wavelength = 254 nm, tR = 15.78 min for major isomer, tR = 14.61 min for minor isomer.

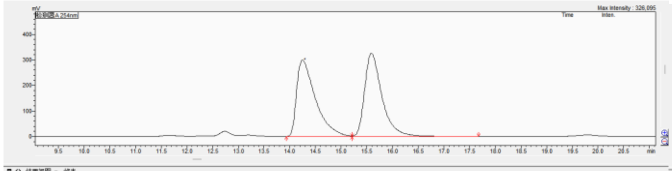

峰号	保留时间	面积	高度	标记	浓度	浓度单位	化合物ID号	化合物名	面积%
1	14.249	7598092	298637	M	49.809				49.809
2	15.591	7656384	325495	V M	50.191				50.191
总计		15254476	624131		100.000				100.000

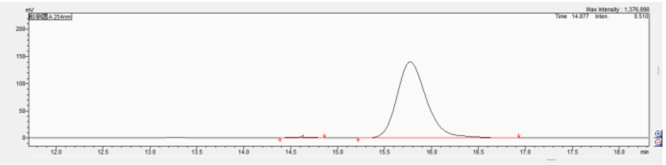

峰号	保留时间	面积	高度	标记	浓度	浓度单位	化合物ID号	化合物名	面积%
1	14.608	20692	1327	M	0.673				0.673
2	15.775	3053045	140014	M	99.327				99.327
总计		3073737	141341		100.000				100.000

N-((2*R*,3*R*)-2-(dihexylamino)-3-methyl-5-phenylpentyl)benzamide (3k)

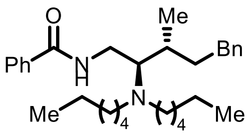

From (*E*)-*N*-(3-methyl-5-phenylpent-2-en-1-yl)benzamide (**1a**) (55.8 mg, 0.20 mmol, 1.0 equiv) and *O*-benzoyl-*N*,*N*-dihexylhydroxylamine (**2k**) (91.6 mg, 0.30 mmol, 1.5 equiv), the title compound was prepared following the general procedure **A** using $NiBr_2$·DME (6.2 mg, 10 mol%), **(*S*,*S*)-L12** (15.4 mg, 15 mol%), LiOH (9.6 mg, 0.40 mmol, 2.0 equiv), KI (33.2 mg, 0.20 mmol, 1.0 equiv), $Me(EtO)_2SiH$ (80 μL, 0.50 mmol, 2.5 equiv), Anhydrous *tert*-butyl alcohol (1.0 mL, 0.20 M). The reaction mixture was stirred for 12 h at 30 °C. The crude material was purified by flash column chromatography (petroleum ether/EtOAc = 3:1) to provide the title compound as a yellow oil in 82% yield (76.2 mg).

^{1}H NMR (400 MHz, $CDCl_3$) δ 7.80 (d, *J* = 7.4 Hz, 2H), 7.54–7.50 (m, 1H), 7.48–7.44 (m, 2H), 7.36–7.31 (m, 2H), 7.24–7.21 (m, 3H), 7.12 (d, *J* = 6.2 Hz, 1H), 3.88–3.78 (m, 1H), 3.11–2.99 (m, 1H), 2.81–2.73 (m, 1H), 2.70–2.59 (m, 4H), 2.53–2.45 (m, 2H), 1.93–1.82 (m, 2H), 1.58–1.53 (m, 1H), 1.42–1.34 (m, 3H), 1.30–1.24 (m, 13H), 1.06 (d, *J* = 6.5 Hz, 3H), 0.89–0.86 (m, 6H); **^{13}C NMR** (101 MHz, $CDCl_3$) δ 167.0, 142.4, 134.9, 131.2, 128.5, 128.4, 128.4, 126.8, 125.8, 63.9, 51.0, 38.3, 37.5, 33.5, 32.3, 31.8, 30.3, 27.2, 22.7, 16.7, 14.1. **HRMS** (ESI) m/z calculated for $C_{31}H_{48}N_2O$ $[M+Na]^+$ 487.3659, found: 487.3659. **Optical rotation**: $[\alpha]^{20}_D$ = -51.9 (c = 1.0 g/L, $CHCl_3$). The absolute configuration was assigned by analogy to that of **4b**. **HPLC condition**: Chiral column IF, n-hexane/i-PrOH = 98:2, flow rate = 0.5 mL/min, wavelength = 254 nm, tR = 55.59 min for major isomer, tR = 60.55 min for minor isomer.

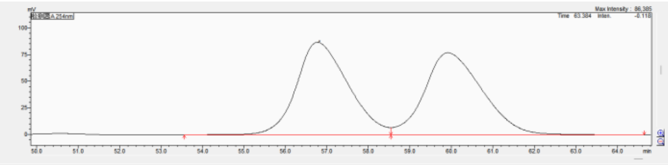

峰号	保留时间	面积	高度	标记	浓度	浓度单位	化合物ID号	化合物名	面积%
1	56.773	7528612	86734		50.263				50.263
2	59.920	7449944	76898	V	49.737				49.737
总计		14978555	163633		100.000				100.000

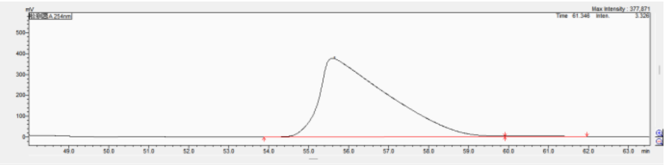

峰号	保留时间	面积	高度	标记	浓度	浓度单位	化合物ID号	化合物名	面积%
1	55.588	44898373	377419	M	99.391				99.391
2	60.551	275088	3090	V M	0.609				0.609
总计		45173461	380510		100.000				100.000

_N_-((2_R_,3_R_)-3-methyl-2-(methyl(pentyl)amino)-5-phenylpentyl)benzamide (3l)

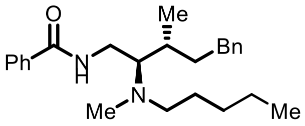

From (_E_)-_N_-(3-methyl-5-phenylpent-2-en-1-yl)benzamide (**1a**) (55.8 mg, 0.20 mmol, 1.0 equiv) and _O_-benzoyl-_N_-methyl-_N_-pentylhydroxylamine (**2l**) (66.3 mg, 0.30 mmol, 1.5 equiv), the title compound was prepared following the general procedure **A** using $NiBr_2$·DME (6.2 mg, 10 mol%), **(_S_,_S_)-L12** (15.4 mg, 15 mol%), LiOH (9.6 mg, 0.40 mmol, 2.0 equiv), KI (33.2 mg, 0.20 mmol, 1.0 equiv), $Me(EtO)_2SiH$ (80 μL, 0.50 mmol, 2.5 equiv), Anhydrous _tert_-butyl alcohol (1.0 mL, 0.20 M). The reaction mixture was stirred for 12 h at 30 °C. The crude material was purified by flash column chromatography (petroleum ether/EtOAc = 3:1) to provide the title compound as a yellow oil in 77% yield (58.6 mg).

^{1}H NMR (400 MHz, $CDCl_3$) δ 7.84 (d, _J_ = 5.8 Hz, 2H), 7.56–7.52 (m, 1H), 7.51–7.47 (m, 2H), 7.37–7.30 (m, 3H), 7.26–7.24 (m, 2H), 7.11 (s, 1H), 3.88–3.77 (m, 1H), 3.23–3.13 (m, 1H), 2.81–2.75 (m, 1H), 2.72–2.61 (m, 3H), 2.59–2.52 (m, 1H), 2.36 (s, 3H), 1.97–1.91 (m, 1H), 1.87–1.80 (m, 1H), 1.55–1.49 (m, 2H), 1.39–1.30 (m, 6H), 1.08 (d, _J_ = 6.6 Hz, 3H), 0.91 (t, _J_ = 6.4 Hz, 3H); **^{13}C NMR** (101 MHz, $CDCl_3$) δ 167.2, 163.7, 134.9, 131.2, 128.5, 128.4, 126.9, 125.8, 66.5, 54.7, 37.8, 36.5, 33.4, 31.4, 29.7, 29.6, 22.7, 16.6, 14.1. **HRMS** (ESI) m/z calculated for $C_{25}H_{36}N_2O$ $[M+H]^+$ 381.2910, found: 381.2910. **Optical rotation**: $[\alpha]^{20}_D$ = 8.23 (c = 1.0 g/L, $CHCl_3$). The absolute configuration was assigned by analogy to that of **4b**. **HPLC condition**: Chiral column AD-H, n-hexane/i-PrOH = 95:5, flow rate = 1 mL/min, wavelength = 254 nm, tR = 11.14 min for major isomer, tR = 9.99 min for minor isomer.

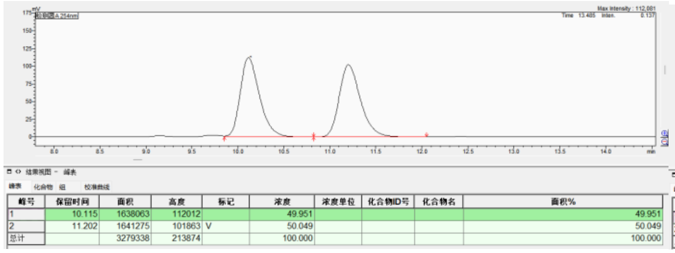

峰号	保留时间	面积	高度	标记	浓度	浓度单位	化合物ID号	化合物名	面积%
1	10.115	1638063	112012		49.951				49.951
2	11.202	1641275	101863	V	50.049				50.049
总计		3279338	213874		100.000				100.000

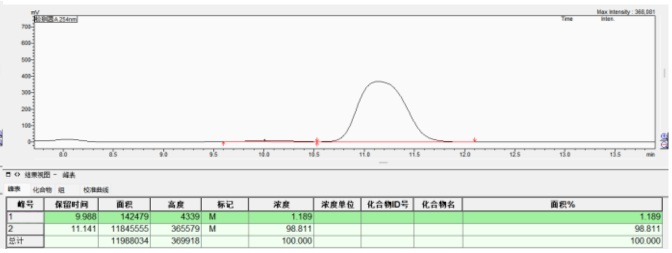

峰号	保留时间	面积	高度	标记	浓度	浓度单位	化合物ID号	化合物名	面积%
1	9.988	142479	4339	M	1.189				1.189
2	11.141	11845555	365579	M	98.811				98.811
总计		11988034	369918		100.000				100.000

_N_-((2_R_,3_R_)-3-methyl-2-(methyl(octyl)amino)-5-phenylpentyl)benzamide (3m)

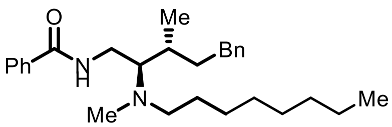

From (_E_)-_N_-(3-methyl-5-phenylpent-2-en-1-yl)benzamide (**1a**) (55.8 mg, 0.20 mmol, 1.0 equiv) and _O_-benzoyl-_N_-methyl-_N_-octylhydroxylamine (**2m**) (79.0 mg, 0.30 mmol, 1.5 equiv), the title compound was prepared following the general procedure **A** using $NiBr_2$·DME (6.2 mg, 10 mol%), **(_S_,_S_)-L12** (15.4 mg, 15 mol%), LiOH (9.6 mg, 0.40 mmol, 2.0 equiv), KI (33.2 mg, 0.20 mmol, 1.0 equiv), $Me(EtO)_2SiH$ (80 μL, 0.50

mmol, 2.5 equiv), Anhydrous *tert*-butyl alcohol (1.0 mL, 0.20 M). The reaction mixture was stirred for 12 h at 30 °C. The crude material was purified by flash column chromatography (petroleum ether/EtOAc = 3:1) to provide the title compound as a yellow oil in 56% yield (47.3 mg).

^{1}H NMR (400 MHz, $CDCl_3$) δ 7.84 (d, *J* = 7.3 Hz, 2H), 7.56–7.52 (m, 1H), 7.50–7.47 (m, 2H), 7.36–7.32 (m, 2H), 7.26–7.23 (m, 3H), 7.14 (s, 1H), 3.86–3.78 (m, 1H), 3.24–3.13 (m, 1H), 2.81–2.75 (m, 1H), 2.72–2.62 (m, 3H), 2.58–2.52 (m, 1H), 2.36 (s, 3H), 1.96–1.91 (m, 1H), 1.87–1.82 (m, 1H), 1.66–1.58 (m, 1H), 1.54–1.48 (m, 2H), 1.40–1.29 (m, 10H), 1.08 (d, *J* = 6.7 Hz, 3H), 0.92 (t, *J* = 6.8 Hz, 3H); **^{13}C NMR** (101 MHz, $CDCl_3$) δ 167.2, 142.3, 134.9, 131.2, 128.5, 128.4, 126.9, 125.8, 66.5, 54.7, 37.8, 37.5, 36.5, 33.4, 31.9, 29.7, 29.4, 27.5, 22.7, 16.7, 14.1. **HRMS** (ESI) m/z calculated for $C_{28}H_{42}N_2O$ $[M+H]^+$ 423.3370, found: 423.3362. **Optical rotation**: $[\alpha]^{20}_D$ = -3.67 (c = 1.0 g/L, $CHCl_3$). The absolute configuration was assigned by analogy to that of **4b**. **HPLC condition**: Chiral column OD-H, n-hexane/i-PrOH = 95:5, flow rate = 1 mL/min, wavelength = 254 nm, tR = 13.17 min for major isomer, tR = 10.79 min for minor isomer.

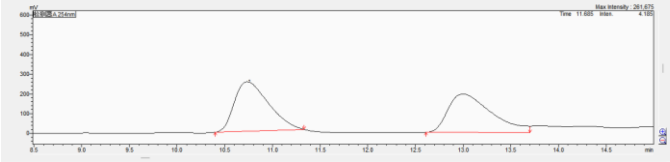

峰号	保留时间	面积	高度	标记	浓度	浓度单位	化合物ID号	化合物名	面积%
1	10.734	6257363	251109	M	50.383				50.383
2	12.997	6162329	194916	M	49.617				49.617
总计		12419692	446025		100.000				100.000

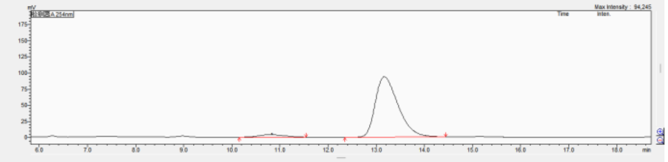

峰号	保留时间	面积	高度	标记	浓度	浓度单位	化合物ID号	化合物名	面积%
1	10.787	141081	3793	M	4.374				4.374
2	13.168	3084676	93447	M	95.626				95.626
总计		3225756	97240		100.000				100.000

N-((2*R*,3*R*)-2-((2-methoxyethyl)(methyl)amino)-3-methyl-5-phenylpentyl)benzamide (3n)

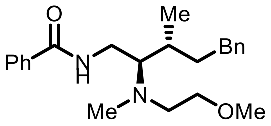

From (*E*)-*N*-(3-methyl-5-phenylpent-2-en-1-yl)benzamide (**1a**) (55.8 mg, 0.20 mmol, 1.0 equiv) and *O*-benzoyl-*N*-(2-methoxyethyl)-*N*-methylhydroxylamine (**2n**) (79.0 mg, 0.30 mmol, 1.5 equiv), the title compound was prepared following the general procedure **A** using $NiBr_2$·DME (6.2 mg, 10 mol%), **(*S*,*S*)-L12** (15.4 mg, 15 mol%), LiOH (9.6 mg, 0.40 mmol, 2.0 equiv), KI (33.2 mg, 0.20 mmol, 1.0 equiv), $Me(EtO)_2SiH$ (80 µL, 0.50 mmol, 2.5 equiv), Anhydrous *tert*-butyl alcohol (1.0 mL, 0.20 M). The reaction mixture was stirred for 12 h at 30 °C. The crude material was purified by flash column chromatography (petroleum ether/EtOAc = 3:1) to provide the title compound as a yellow oil in 64% yield (47.1 mg).

^{1}H NMR (400 MHz, $CDCl_3$) δ 7.86 (d, *J* = 7.4 Hz, 2H), 7.49–7.44 (m, 1H), 7.43–7.39 (m, 2H), 7.30–7.26 (m, 2H), 7.22–7.15 (m, 4H), 3.92–3.83 (m, 1H), 3.50–3.39 (m, 2H), 3.23 (s, 3H), 3.07–2.93 (m, 2H),

2.82–2.70 (m, 2H), 2.65–2.51 (m, 2H), 2.37 (s, 3H), 1.90–1.73 (m, 3H), 1.02 (d, *J* = 6.7 Hz, 3H); **^{13}C NMR** (101 MHz, $CDCl_3$) δ 167.5, 142.4, 131.0, 128.4, 128.4, 128.3, 127.3, 126.9, 125.9, 70.4, 67.1, 58.7, 55.1, 39.0, 36.9, 35.9, 33.3, 22.0, 16.4. **HRMS** (ESI) m/z calculated for $C_{23}H_{32}N_2O_2$ $[M+H]^+$ 369.2537, found: 369.2537. **Optical rotation**: $[\alpha]^{20}_D$ = -253 (c = 1.0 g/L, $CHCl_3$). The absolute configuration was assigned by analogy to that of **4b**. **HPLC condition**: Chiral column AD-H, n-hexane/i-PrOH = 95:5, flow rate = 1 mL/min, wavelength = 254 nm, tR = 19.68 min for major isomer, tR = 16.52 min for minor isomer.

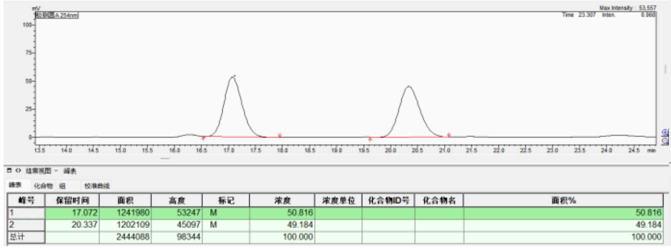

峰号	保留时间	面积	高度	标记	浓度	浓度单位	化合物ID号	化合物名	面积%
1	17.072	1241980	53247	M	50.816				50.816
2	20.337	1202109	45097	M	49.184				49.184
总计		2444088	98344		100.000				100.000

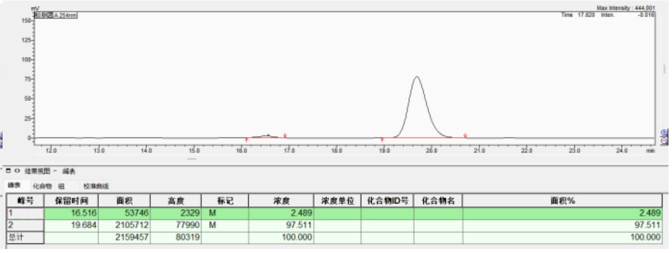

峰号	保留时间	面积	高度	标记	浓度	浓度单位	化合物ID号	化合物名	面积%
1	16.516	53746	2329	M	2.489				2.489
2	19.684	2105712	77990	M	97.511				97.511
总计		2159457	80319		100.000				100.000

***N*-((2*R*,3*R*)-2-(dibenzylamino)-3-methyl-5-phenylpentyl)benzamide (3o)**

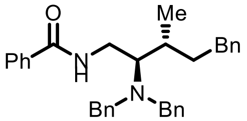

From (*E*)-*N*-(3-methyl-5-phenylpent-2-en-1-yl)benzamide (**1a**) (55.8 mg, 0.20 mmol, 1.0 equiv) and *O*-benzoyl-*N*,*N*-dibenzylhydroxylamine (**2o**) (95.1 mg, 0.30 mmol, 1.5 equiv), the title compound was prepared following the general procedure **A** using $NiBr_2$·DME (6.2 mg, 10 mol%), **(*S*,*S*)-L12** (15.4 mg, 15 mol%), LiOH (9.6 mg, 0.40 mmol, 2.0 equiv), KI (33.2 mg, 0.20 mmol, 1.0 equiv), $Me(EtO)_2SiH$ (80 μL, 0.50 mmol, 2.5 equiv), Anhydrous *tert*-butyl alcohol (1.0 mL, 0.20 M). The reaction mixture was stirred for 12 h at 30 °C. The crude material was purified by flash column chromatography (petroleum ether/EtOAc = 3:1) to provide the title compound as a yellow oil in 82% yield (78.1 mg).

^{1}H NMR (400 MHz, $CDCl_3$) δ 7.65 (d, *J* = 7.7 Hz, 2H), 7.59–7.53 (m, 1H), 7.49–7.45 (m, 2H), 7.40–7.35 (m, 2H), 7.29–7.25 (m, 9H), 7.22–7.19 (m, 4H), 6.56 (d, *J* = 4.7 Hz, 1H), 3.94–3.88 (m, 2H), 3.71–3.62 (m, 1H), 3.57–3.52 (m, 2H), 3.46–3.37 (m, 1H), 2.84–2.73 (m, 2H), 2.68–2.61 (m, 1H), 2.20–2.10 (m, 1H), 1.90–1.82 (m, 1H), 1.70–1.60 (m, 1H), 1.16 (d, *J* = 6.8 Hz, 3H); **^{13}C NMR** (101 MHz, $CDCl_3$) δ 167.3, 142.2, 139.9, 134.8, 131.3, 129.2, 128.5, 128.5, 128.5, 128.5, 127.3, 126.9, 126.0, 61.3, 53.9, 37.9, 37.6, 33.5, 30.7, 16.5. **HRMS** (ESI) m/z calculated for $C_{33}H_{36}N_2O$ $[M+H]^+$ 477.2901, found: 477.2901. **Optical rotation**: $[\alpha]^{20}_D$ = 156 (c = 1.0 g/L, $CHCl_3$). The absolute configuration was assigned by analogy to that of **4b**. **HPLC condition**: Chiral column AD-H, n-hexane/i-PrOH = 95:5, flow rate = 1 mL/min, wavelength = 254 nm, tR = 35.50 min for major isomer, tR = 32.66 min for minor isomer.

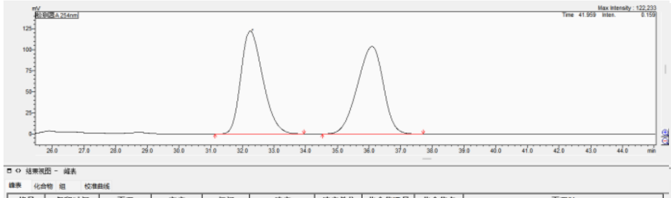

峰号	保留时间	面积	高度	标记	浓度	浓度单位	化合物ID号	化合物名	面积%
1	32.246	5869001	121945		49.962				49.962
2	36.093	5877876	103432		50.038				50.038
总计		11746878	225377		100.000				100.000

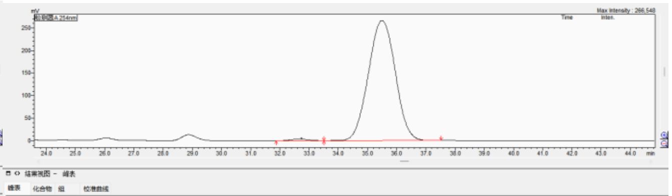

峰号	保留时间	面积	高度	标记	浓度	浓度单位	化合物ID号	化合物名	面积%
1	32.659	156346	3551	M	0.909				0.909
2	35.496	17049832	266057	V M	99.091				99.091
总计		17206178	269607		100.000				100.000

N-((2*R*,3*R*)-2-(benzyl(2-chloroethyl)amino)-3-methyl-5-phenylpentyl)benzamide (3p)

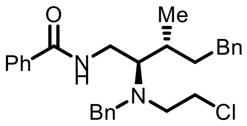

From (*E*)-*N*-(3-methyl-5-phenylpent-2-en-1-yl)benzamide (**1a**) (55.8 mg, 0.20 mmol, 1.0 equiv) and *O*-benzoyl-*N*-benzyl-*N*-(2-chloroethyl)hydroxylamine (**2p**) (86.7 mg, 0.30 mmol, 1.5 equiv), the title compound was prepared following the general procedure **A** using $NiBr_2$·DME (6.2 mg, 10 mol%), **(*S*,*S*)-L12** (15.4 mg, 15 mol%), LiOH (9.6 mg, 0.40 mmol, 2.0 equiv), KI (33.2 mg, 0.20 mmol, 1.0 equiv), $Me(EtO)_2SiH$ (80 µL, 0.50 mmol, 2.5 equiv), Anhydrous *tert*-butyl alcohol (1.0 mL, 0.20 M). The reaction mixture was stirred for 12 h at 30 °C. The crude material was purified by flash column chromatography (petroleum ether/EtOAc = 3:1) to provide the title compound as a yellow oil in 46% yield (41.2 mg).

^{1}H NMR (400 MHz, $CDCl_3$) δ 7.73 (d, *J* = 7.5 Hz, 2H), 7.51–7.48 (m, 1H), 7.45–7.42 (m, 2H), 7.34–7.27 (m, 3H), 7.23–7.18 (m, 7H), 7.04–6.99 (m, 1H), 3.87–3.78 (m, 2H), 3.60–3.54 (m, 1H), 3.49–3.38 (m, 1H), 3.36–3.30 (m, 1H), 3.16–3.05 (m, 2H), 3.01–2.88 (m, 1H), 2.82–2.75 (m, 1H), 2.74–2.67 (m, 1H), 2.61–2.52 (m, 1H), 1.95–1.89 (m, 1H), 1.86–1.76 (m, 1H), 1.61–1.53 (m, 1H), 1.08 (d, *J* = 6.7 Hz, 3H); **^{13}C NMR** (101 MHz, $CDCl_3$) δ 167.2, 142.1, 139.6, 134.7, 131.3, 128.9, 128.6, 128.5, 128.4, 127.5, 127.0, 126.0, 64.5, 55.4, 53.1, 44.1, 38.4, 37.4, 33.4, 32.3, 16.7. **HRMS** (ESI) m/z calculated for $C_{28}H_{33}ClN_2O$ $[M+Na]^+$ 471.2173, found: 471.2175. **Optical rotation**: $[\alpha]^{20}_D$ = -86.0 (c = 1.0 g/L, $CHCl_3$). The absolute configuration was assigned by analogy to that of **4b**. **HPLC condition**: Chiral column OD-H, n-hexane/i-PrOH = 95:5, flow rate = 1 mL/min, wavelength = 254 nm, tR = 32.21 min for major isomer, tR = 41.56 min for minor isomer.

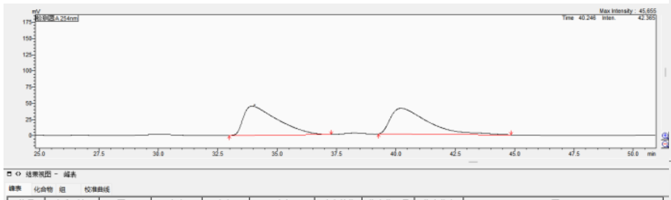

峰号	保留时间	面积	高度	标记	浓度	浓度单位	化合物ID号	化合物名	面积%
1	33.949	4480768	44829	M	50.757				50.757
2	40.220	4347198	39761	M	49.243				49.243
总计		8827966	84591		100.000				100.000

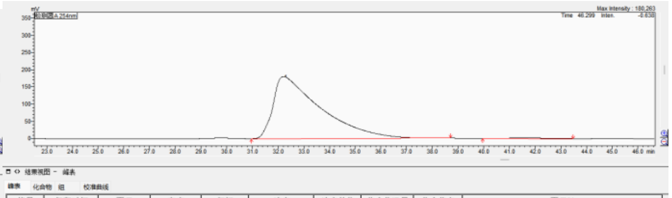

峰号	保留时间	面积	高度	标记	浓度	浓度单位	化合物ID号	化合物名	面积%
1	32.210	23079055	180473	M	99.081				99.081
2	41.555	214159	2242	M	0.919				0.919
总计		23293214	182714		100.000				100.000

N-((2R,3R)-2-(benzyl(3-chloropropyl)amino)-3-methyl-5-phenylpentyl)benzamide (3q)

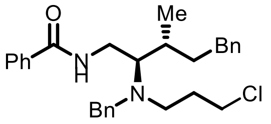

From (*E*)-*N*-(3-methyl-5-phenylpent-2-en-1-yl)benzamide (**1a**) (55.8 mg, 0.20 mmol, 1.0 equiv) and *O*-benzoyl-*N*-benzyl-*N*-(3-chloropropyl)hydroxylamine (**2q**) (90.9 mg, 0.30 mmol, 1.5 equiv), the title compound was prepared following the general procedure **A** using $NiBr_2$·DME (6.2 mg, 10 mol%), **(*S*,*S*)-L12** (15.4 mg, 15 mol%), LiOH (9.6 mg, 0.40 mmol, 2.0 equiv), KI (33.2 mg, 0.20 mmol, 1.0 equiv), $Me(EtO)_2SiH$ (80 µL, 0.50 mmol, 2.5 equiv), Anhydrous *tert*-butyl alcohol (1.0 mL, 0.20 M). The reaction mixture was stirred for 12 h at 30 °C. The crude material was purified by flash column chromatography (petroleum ether/EtOAc = 3:1) to provide the title compound as a yellow oil in 68% yield (62.9 mg).

^{1}H NMR (400 MHz, $CDCl_3$) δ 7.68–7.61 (m, 2H), 7.53–7.48 (m, 1H), 7.46–7.40 (m, 2H), 7.34–7.29 (m, 2H), 7.24–7.18 (m, 8H), 6.61–6.52 (m, 1H), 3.79 (d, *J* = 13.7 Hz, 1H), 3.74–3.65 (m, 1H), 3.55 (d, *J* = 13.7 Hz, 1H), 3.46 (t, *J* = 6.3 Hz, 2H), 3.30–3.22 (m, 1H), 2.96–2.86 (m, 1H), 2.80–2.71 (m, 1H), 2.71–2.63 (m, 2H), 2.62–2.53 (m, 1H), 2.01–1.92 (m, 1H), 1.90–1.78 (m, 3H), 1.63–1.51 (m, 1H), 1.08 (d, *J* = 6.7 Hz, 3H); **^{13}C NMR** (101 MHz, $CDCl_3$) δ 167.2, 142.2, 140.1, 134.7, 131.3, 128.8, 128.6, 128.6, 128.5, 128.5, 127.3, 126.8, 125.9, 63.5, 55.3, 47.2, 42.9, 38.0, 37.6, 33.4, 32.1, 31.5, 16.6. **HRMS** (ESI) m/z calculated for $C_{29}H_{35}ClN_2O$ $[M+Na]^+$ 485.2330, found: 485.2330. **Optical rotation**: $[\alpha]^{20}_D$ = 7.17 (c = 1.0 g/L, $CHCl_3$). The absolute configuration was assigned by analogy to that of **4b**. **HPLC condition**: Chiral column AD-H, n-hexane/i-PrOH = 95:5, flow rate = 1 mL/min, wavelength = 254 nm, tR = 25.43 min for major isomer, tR = 28.98 min for minor isomer.

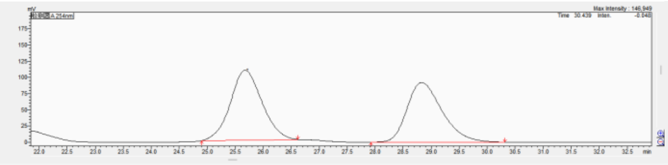

峰号	保留时间	面积	高度	标记	浓度	浓度单位	化合物ID号	化合物名	面积%
1	25.676	4234102	107792	M	51.377				51.377
2	28.831	4007182	92169	M	48.623				48.623
总计		8241285	199961		100.000				100.000

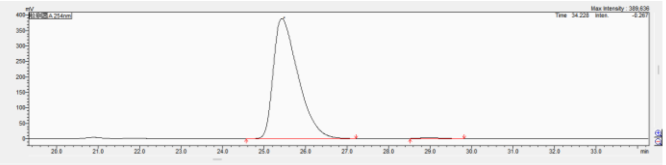

峰号	保留时间	面积	高度	标记	浓度	浓度单位	化合物ID号	化合物名	面积%
1	25.427	16171719	389357	M	99.352				99.352
2	28.977	105544	2782	M	0.648				0.648
总计		16277263	392139		100.000				100.000

N-((2R,3R)-2-(benzyl(isopropyl)amino)-3-methyl-5-phenylpentyl)benzamide (3r)

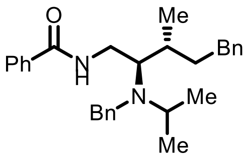

From (*E*)-*N*-(3-methyl-5-phenylpent-2-en-1-yl)benzamide (**1a**) (55.8 mg, 0.20 mmol, 1.0 equiv) and *O*-benzoyl-*N*-benzyl-*N*-isopropylhydroxylamine (**2r**) (80.7 mg, 0.30 mmol, 1.5 equiv), the title compound was prepared following the general procedure **A** using $NiBr_2$·DME (6.2 mg, 10 mol%), **(*S*,*S*)-L12** (15.4 mg, 15 mol%), LiOH (9.6 mg, 0.40 mmol, 2.0 equiv), KI (33.2 mg, 0.20 mmol, 1.0 equiv), $Me(EtO)_2SiH$ (80 μL, 0.50 mmol, 2.5 equiv), Anhydrous *tert*-butyl alcohol (1.0 mL, 0.20 M). The reaction mixture was stirred for 12 h at 30 °C. The crude material was purified by flash column chromatography (petroleum ether/EtOAc = 3:1) to provide the title compound as a yellow oil in 80% yield (68.5 mg).

^{1}H NMR (400 MHz, $CDCl_3$) δ 7.63 (d, *J* = 7.4 Hz, 2H), 7.53–7.49 (m, 1H), 7.46–7.42 (m, 2H), 7.35–7.29 (m, 2H), 7.28–7.25 (m, 4H), 7.24–7.20 (m, 4H), 6.51–6.45 (m, 1H), 3.88 (d, *J* = 14.0 Hz, 1H), 3.63–3.51 (m, 2H), 3.37–3.26 (m, 1H), 3.26–3.15 (m, 1H), 2.81–2.68 (m, 2H), 2.63–2.54 (m, 1H), 2.07–1.96 (m, 1H), 1.81–1.74 (m, 1H), 1.63–1.51 (m, 1H), 1.14 (d, *J* = 6.5 Hz, 3H), 1.11–1.07 (m, 6H); **^{13}C NMR** (101 MHz, $CDCl_3$) δ 167.3, 142.3, 141.4, 134.9, 131.2, 128.8, 128.6, 128.4, 127.0, 126.9, 125.9, 60.6, 49.4, 46.6, 38.2, 37.8, 33.8, 32.5, 23.4, 19.7, 16.1. **HRMS** (ESI) m/z calculated for $C_{29}H_{36}N_2O$ $[M+H]^+$ 429.2901, found: 429.2901. **Optical rotation**: $[\alpha]^{20}_D$ = 6.40 (c = 1.0 g/L, $CHCl_3$). The absolute configuration was assigned by analogy to that of **4b**. **HPLC condition**: Chiral column OD-H, n-hexane/i-PrOH = 95:5, flow rate = 1 mL/min, wavelength = 254 nm, tR = 15.47 min for major isomer, tR = 18.63 min for minor isomer.

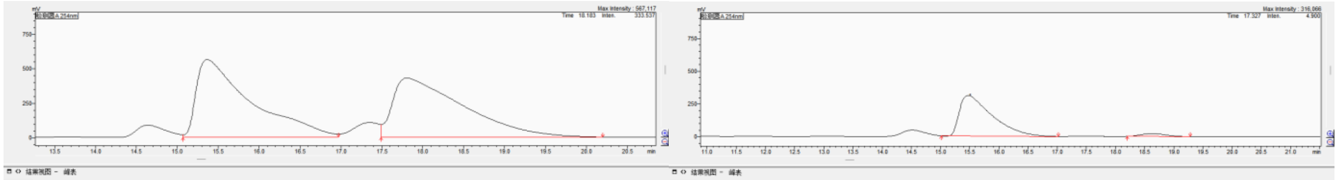

峰号	保留时间	面积	高度	标记	浓度	浓度单位	化合物ID号	化合物名	面积%
1	15.365	26093552	562813	M	50.386				50.386
2	17.807	25693983	429326	M	49.614				49.614
总计		51787535	992140		100.000				100.000

峰号	保留时间	面积	高度	标记	浓度	浓度单位	化合物ID号	化合物名	面积%
1	15.473	11963446	309329	M	94.796				94.796
2	18.626	656792	22285	M	5.204				5.204
总计		12620237	331613		100.000				100.000

N-((2*R*,3*R*)-2-((4-methoxybenzyl)(methyl)amino)-3-methyl-5-phenylpentyl)benzamide (3s)

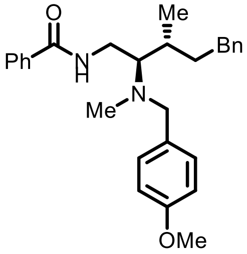

From (*E*)-*N*-(3-methyl-5-phenylpent-2-en-1-yl)benzamide (**1a**) (55.8 mg, 0.20 mmol, 1.0 equiv) and *O*-benzoyl-*N*-(4-methoxybenzyl)-*N*-methylhydroxylamine (**2s**) (81.3 mg, 0.30 mmol, 1.5 equiv), the title compound was prepared following the general procedure **A** using $NiBr_2$·DME (6.2 mg, 10 mol%), **(*S*,*S*)-L12** (15.4 mg, 15 mol%), LiOH (9.6 mg, 0.40 mmol, 2.0 equiv), KI (33.2 mg, 0.20 mmol, 1.0 equiv),

Me(EtO)$_2$SiH (80 μL, 0.50 mmol, 2.5 equiv), Anhydrous *tert*-butyl alcohol (1.0 mL, 0.20 M). The reaction mixture was stirred for 12 h at 30 °C. The crude material was purified by flash column chromatography (petroleum ether/EtOAc = 3:1) to provide the title compound as a yellow oil in 48% yield (41.3 mg).

^{1}H NMR (400 MHz, CDCl$_3$) δ 7.75 (d, J = 7.6 Hz, 2H), 7.57–7.52 (m, 1H), 7.49–7.44 (m, 2H), 7.38–7.33 (m, 2H), 7.28–7.24 (m, 3H), 7.19 (d, J = 8.1 Hz, 2H), 6.94 (d, J = 6.8 Hz, 1H), 6.85 (d, J = 8.1 Hz, 2H), 3.83 (s, 3H), 3.81–3.72 (m, 2H), 3.62 (d, J = 13.1 Hz, 1H), 3.32–3.19 (m, 1H), 2.84–2.71 (m, 2H), 2.69–2.61 (m, 1H), 2.32 (s, 3H), 2.06–1.96 (m, 1H), 1.90–1.82 (m, 1H), 1.70–1.59 (m, 1H), 1.11 (d, J = 6.7 Hz, 3H); **^{13}C NMR** (101 MHz, CDCl$_3$) δ 167.2, 158.8, 142.3, 134.9, 131.9, 131.3, 129.9, 128.5, 128.5, 127.0, 125.9, 113.9, 65.3, 58.8, 55.3, 37.8, 37.5, 36.4, 33.4, 31.3, 16.6. **HRMS** (ESI) m/z calculated for $C_{28}H_{34}N_2O_2$ $[M+H]^+$ 431.2693, found: 431.2693. **Optical rotation**: $[\alpha]^{20}_D$ = 12.8 (c = 1.0 g/L, CHCl$_3$). The absolute configuration was assigned by analogy to that of **4b**. **HPLC condition**: Chiral column AD-H, n-hexane/i-PrOH = 95:5, flow rate = 1 mL/min, wavelength = 254 nm, tR = 31.16 min for major isomer, tR = 29.46 min for minor isomer.

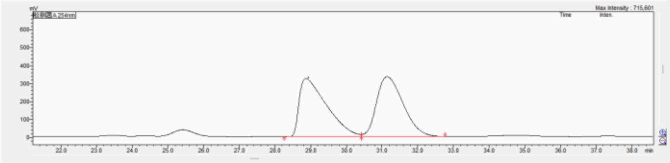

结果视图 - 峰表

峰号	保留时间	面积	高度	标记	浓度	浓度单位	化合物ID号	化合物名	面积%
1	28.866	17284985	324169	M	50.009				50.009
2	31.143	17278703	334549	V M	49.991				49.991
总计		34563687	658718		100.000				100.000

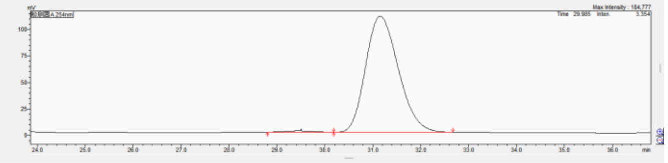

结果视图 - 峰表

峰号	保留时间	面积	高度	标记	浓度	浓度单位	化合物ID号	化合物名	面积%
1	29.457	54950	1275	M	1.049				1.049
2	31.156	5185544	109411	M	98.951				98.951
总计		5240494	110686		100.000				100.000

N-((2*R*,3*R*)-2-((4-fluorobenzyl)(methyl)amino)-3-methyl-5-phenylpentyl)benzamide (3t)

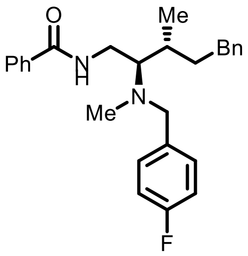

From (*E*)-*N*-(3-methyl-5-phenylpent-2-en-1-yl)benzamide (**1a**) (55.8 mg, 0.20 mmol, 1.0 equiv) and *O*-benzoyl-*N*-(4-fluorobenzyl)-*N*-methylhydroxylamine (**2t**) (77.7 mg, 0.30 mmol, 1.5 equiv), the title compound was prepared following the general procedure **A** using NiBr$_2$·DME (6.2 mg, 10 mol%), **(*S*,*S*)-L12** (15.4 mg, 15 mol%), LiOH (9.6 mg, 0.40 mmol, 2.0 equiv), KI (33.2 mg, 0.20 mmol, 1.0 equiv), Me(EtO)$_2$SiH (80 μL, 0.50 mmol, 2.5 equiv), Anhydrous *tert*-butyl alcohol (1.0 mL, 0.20 M). The reaction mixture was stirred for 12 h at 30 °C. The crude material was purified by flash column chromatography (petroleum ether/EtOAc = 3:1) to provide the title compound as a yellow oil in 64% yield (53.5 mg).

^{1}H NMR (400 MHz, CDCl$_3$) δ 7.66–7.60 (m, 2H), 7.46–7.41 (m, 1H), 7.37–7.33 (m, 2H), 7.25–7.20 (m, 2H), 7.14–7.08 (m, 5H), 6.90–6.82 (m, 2H), 6.71 (d, J = 6.6 Hz, 1H), 3.69–3.60 (m, 2H), 3.51 (d, J = 13.4

Hz, 1H), 3.23–3.13 (m, 1H), 2.71–2.59 (m, 2H), 2.55–2.47 (m, 1H), 2.19 (s, 3H), 1.91–1.83 (m, 1H), 1.76–1.70 (m, 1H), 1.55–1.47 (m, 1H), 0.99 (d, *J* = 6.8 Hz, 3H); **^{13}C NMR** (101 MHz, $CDCl_3$) δ 167.2, 162.0 (d, *J* = 245.4 Hz), 142.3, 135.2 (d, *J* = 79.2 Hz), 131.4, 130.1 (d, *J* = 8.0 Hz), 128.6, 128.5, 126.9, 125.9, 115.4, 115.2, 65.9, 58.7, 37.8, 37.4, 36.4, 33.3, 31.4, 16.6; **19F NMR** (376 MHz, $CDCl_3$) δ -117.13. **HRMS** (ESI) m/z calculated for $C_{27}H_{31}FN_2O$ $[M+H]^+$ 419.2493, found: 419.2493. **Optical rotation**: $[\alpha]^{20}_D$ = -46.3 (c = 1.0 g/L, $CHCl_3$). The absolute configuration was assigned by analogy to that of **4b**. **HPLC condition**: Chiral column OD-H, n-hexane/i-PrOH = 95:5, flow rate = 1 mL/min, wavelength = 254 nm, tR = 31.98 min for major isomer, tR = 42.66 min for minor isomer.

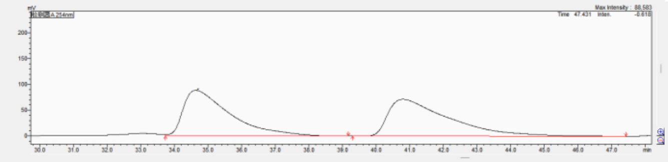

峰号	保留时间	面积	高度	标记	浓度	浓度单位	化合物ID号	化合物名	面积%
1	34.637	8659241	88946		49.159				49.159
2	40.791	8955414	71577		50.841				50.841
总计		17614655	160523		100.000				100.000

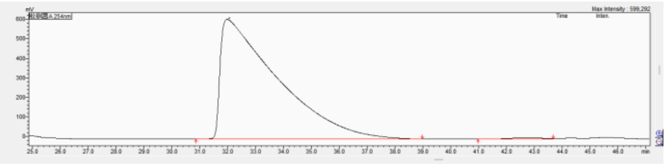

峰号	保留时间	面积	高度	标记	浓度	浓度单位	化合物ID号	化合物名	面积%
1	31.979	87011067	611981	M	99.303				99.303
2	42.660	610328	6197	M	0.697				0.697
总计		87621395	618178		100.000				100.000

_N_-((2_R_,3_R_)-2-(benzyl(4-chlorobenzyl)amino)-3-methyl-5-phenylpentyl)benzamide (3u)

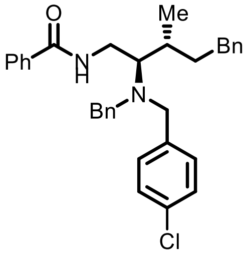

From (*E*)-*N*-(3-methyl-5-phenylpent-2-en-1-yl)benzamide (**1a**) (55.8 mg, 0.20 mmol, 1.0 equiv) and *O*-benzoyl-*N*-benzyl-*N*-(4-chlorobenzyl)hydroxylamine (**2u**) (105.3 mg, 0.30 mmol, 1.5 equiv), the title compound was prepared following the general procedure **A** using $NiBr_2$·DME (6.2 mg, 10 mol%), **(*S*,*S*)-L12** (15.4 mg, 15 mol%), LiOH (9.6 mg, 0.40 mmol, 2.0 equiv), KI (33.2 mg, 0.20 mmol, 1.0 equiv), $Me(EtO)_2SiH$ (80 μL, 0.50 mmol, 2.5 equiv), Anhydrous *tert*-butyl alcohol (1.0 mL, 0.20 M). The reaction mixture was stirred for 12 h at 30 °C. The crude material was purified by flash column chromatography (petroleum ether/EtOAc = 3:1) to provide the title compound as a yellow oil in 53% yield (54.1 mg).

^{1}H NMR (400 MHz, $CDCl_3$) δ 7.57 (d, *J* = 7.4 Hz, 2H), 7.54–7.50 (m, 1H), 7.46–7.42 (m, 2H), 7.35–7.30 (m, 2H), 7.24–7.19 (m, 6H), 7.18–7.13 (m, 4H), 7.05 (d, *J* = 8.2 Hz, 2H), 6.36 (s, 1H), 3.81 (d, *J* = 13.3 Hz, 2H), 3.63–3.56 (m, 1H), 3.52–3.45 (m, 2H), 3.44–3.36 (m, 1H), 2.79–2.73 (m, 1H), 2.68–2.57 (m, 2H), 2.10–2.03 (m, 1H), 1.84–1.77 (m, 1H), 1.63–1.59 (m, 1H), 1.11 (d, *J* = 6.7 Hz, 3H); **^{13}C NMR** (101 MHz, $CDCl_3$) δ 167.3, 142.1, 139.7, 138.5, 134.7, 132.9, 131.4, 130.4, 129.1, 128.6, 128.6, 128.5, 128.5, 127.4, 126.8, 126.0, 61.6, 53.9, 53.3, 37.8, 37.5, 33.4, 30.8, 16.5. **HRMS** (ESI) m/z calculated for $C_{33}H_{35}ClN_2O$ $[M+H]^+$ 511.2511, found: 511.2511. **Optical rotation**: $[\alpha]^{20}_D$ = 10.233 (c = 1.0 g/L, $CHCl_3$).

The absolute configuration was assigned by analogy to that of **4b**. **HPLC condition**: Chiral column OD-H, n-hexane/i-PrOH = 95:5, flow rate = 1 mL/min, wavelength = 254 nm, tR = 26.49 min for major isomer, tR = 37.90 min for minor isomer.

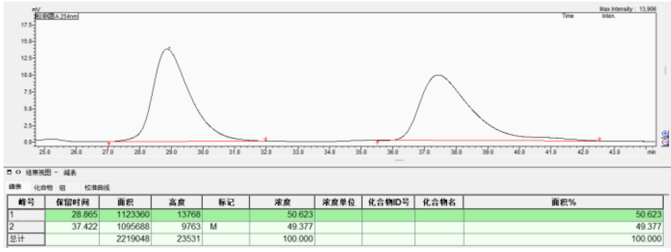

峰号	保留时间	面积	高度	标记	浓度	浓度单位	化合物ID号	化合物名	面积%
1	28.865	1123360	13768		50.623				50.623
2	37.422	1095688	9763	M	49.377				49.377
总计		2219048	23531		100.000				100.000

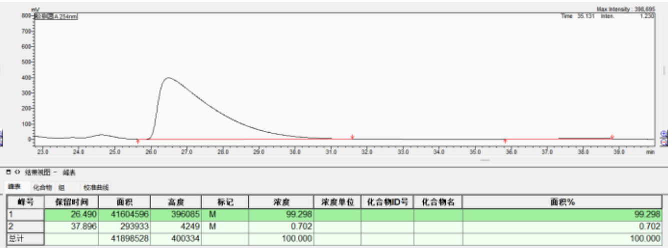

峰号	保留时间	面积	高度	标记	浓度	浓度单位	化合物ID号	化合物名	面积%
1	26.490	41604596	396085	M	99.298				99.298
2	37.896	293933	4249	M	0.702				0.702
总计		41898528	400334		100.000				100.000

N-((2R,3R)-2-(benzyl(4-(methylthio)benzyl)amino)-3-methyl-5-phenylpentyl)benzamide (3v)

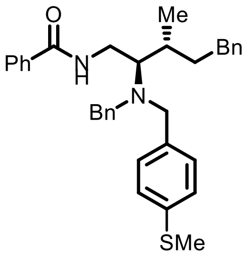

From (*E*)-*N*-(3-methyl-5-phenylpent-2-en-1-yl)benzamide (**1a**) (55.8 mg, 0.20 mmol, 1.0 equiv) and *O*-benzoyl-*N*-benzyl-*N*-(4-(methylthio)benzyl)hydroxylamine (**2v**) (108.9 mg, 0.30 mmol, 1.5 equiv), the title compound was prepared following the general procedure **A** using $NiBr_2$·DME (6.2 mg, 10 mol%), **(*S*,*S*)-L12** (15.4 mg, 15 mol%), LiOH (9.6 mg, 0.40 mmol, 2.0 equiv), KI (33.2 mg, 0.20 mmol, 1.0 equiv), $Me(EtO)_2SiH$ (80 µL, 0.50 mmol, 2.5 equiv), Anhydrous tert-butyl alcohol (1.0 mL, 0.20 M). The reaction mixture was stirred for 12 h at 30 °C. The crude material was purified by flash column chromatography (petroleum ether/EtOAc = 3:1) to provide the title compound as a yellow oil in 75% yield (78.3 mg).

^{1}H NMR (400 MHz, $CDCl_3$) δ 7.63 (d, *J* = 7.5 Hz, 2H), 7.58–7.52 (m, 1H), 7.51–7.44 (m, 2H), 7.40–7.33 (m, 2H), 7.29–7.23 (m, 6H), 7.19 (d, *J* = 6.9 Hz, 2H), 7.16–7.06 (m, 4H), 6.51 (d, *J* = 6.1 Hz, 1H), 3.92–3.79 (m, 2H), 3.68–3.59 (m, 1H), 3.56–3.45 (m, 2H), 3.45–3.36 (m, 1H), 2.86–2.76 (m, 1H), 2.75–2.69 (m, 1H), 2.67–2.57 (m, 1H), 2.45 (s, 3H), 2.18–2.07 (m, 1H), 1.87–1.78 (m, 1H), 1.69–1.58 (m, 1H), 1.14 (d, *J* = 6.8 Hz, 3H); **^{13}C NMR** (101 MHz, $CDCl_3$) δ 167.3, 142.2, 139.9, 137.2, 136.8, 134.7, 131.3, 129.6, 129.2, 128.6, 128.5, 127.3, 126.9, 126.8, 126.0, 61.4, 53.8, 53.5, 37.9, 37.6, 33.5, 30.7, 16.5, 15.9. **HRMS** (ESI) m/z calculated for $C_{34}H_{38}N_2OS$ $[M+H]^+$ 523.2778, found: 523.2778. **Optical rotation**: $[\alpha]^{20}_D$ = 28.4 (c = 1.0 g/L, $CHCl_3$). The absolute configuration was assigned by analogy to that of **4b**. **HPLC condition**: Chiral column OD-H, n-hexane/i-PrOH = 95:5, flow rate = 1 mL/min, wavelength = 254 nm, tR = 32.02 min for major isomer, tR = 44.35 min for minor isomer.

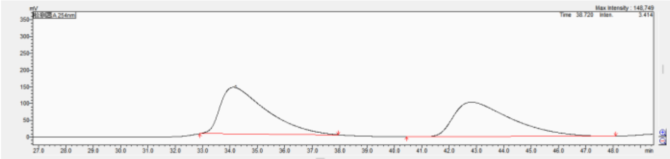

峰号	保留时间	面积	高度	标记	浓度	浓度单位	化合物ID号	化合物名	面积%
1	34.119	16225957	138921	M	51.925				51.925
2	42.823	15022616	102746	M	48.075				48.075
总计		31248573	241667		100.000				100.000

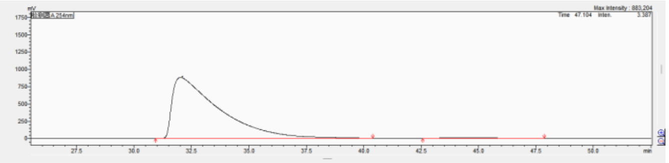

峰号	保留时间	面积	高度	标记	浓度	浓度单位	化合物ID号	化合物名	面积%
1	32.020	125240683	881190	M	98.898				98.898
2	44.347	1394999	9553	M	1.102				1.102
总计		126635682	890743		100.000				100.000

N-((2R,3R)-2-(benzyl(4-(trifluoromethoxy)benzyl)amino)-3-methyl-5-phenylpentyl)benzamide (3w)

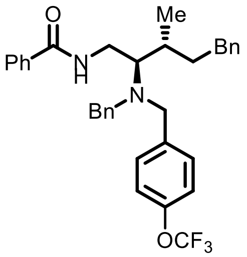

From (*E*)-*N*-(3-methyl-5-phenylpent-2-en-1-yl)benzamide (**1a**) (55.8 mg, 0.20 mmol, 1.0 equiv) and *O*-benzoyl-*N*-benzyl-*N*-(4-(trifluoromethoxy)benzyl)hydroxylamine (**2w**) (108.9 mg, 0.30 mmol, 1.5 equiv), the title compound was prepared following the general procedure **A** using $NiBr_2$·DME (6.2 mg, 10 mol%), **(*S*,*S*)-L12** (15.4 mg, 15 mol%), LiOH (9.6 mg, 0.40 mmol, 2.0 equiv), KI (33.2 mg, 0.20 mmol, 1.0 equiv), $Me(EtO)_2SiH$ (80 μL, 0.50 mmol, 2.5 equiv), Anhydrous *tert*-butyl alcohol (1.0 mL, 0.20 M). The reaction mixture was stirred for 12 h at 30 °C. The crude material was purified by flash column chromatography (petroleum ether/EtOAc = 3:1) to provide the title compound as a yellow oil in 85% yield (95.2 mg).

^{1}H NMR (400 MHz, $CDCl_3$) δ 7.63 (d, *J* = 7.6 Hz, 2H), 7.59–7.54 (m, 1H), 7.50–7.44 (m, 2H), 7.39–7.33 (m, 2H), 7.29–7.23 (m, 6H), 7.21–7.15 (m, 4H), 7.11–7.05 (m, 2H), 6.42 (d, *J* = 6.1 Hz, 1H), 3.92–3.80 (m, 2H), 3.67–3.59 (m, 1H), 3.55 (d, *J* = 13.3 Hz, 2H), 3.51–3.42 (m, 1H), 2.86–2.76 (m, 1H), 2.75–2.68 (m, 1H), 2.66–2.57 (m, 1H), 2.17–2.06 (m, 1H), 1.90–1.79 (m, 1H), 1.68–1.58 (m, 1H), 1.15 (d, *J* = 6.7 Hz, 3H); **^{13}C NMR** (101 MHz, $CDCl_3$) δ 167.3, 148.3, 142.1, 139.7, 138.7, 134.7, 131.4, 130.3, 129.1, 128.6, 128.5, 128.5, 127.4, 126.8, 126.0, 120.9, 120.4 (q, *J* = 257.0 Hz), 61.8, 54.1, 53.3, 37.7, 37.6, 33.4, 30.9, 16.5; **^{19}F NMR** (376 MHz, $CDCl_3$) δ -57.85. **HRMS** (ESI) m/z calculated for $C_{34}H_{35}F_3N_2O_2$ $[M+Na]^+$ 583.2543, found: 583.2537. **Optical rotation**: $[\alpha]^{20}_D$ = 10.6 (c = 1.0 g/L, $CHCl_3$). The absolute configuration was assigned by analogy to that of **4b**. **HPLC condition**: Chiral column OD-H, n-hexane/i-PrOH = 95:5, flow rate = 1 mL/min, wavelength = 254 nm, tR = 31.8 min for major isomer, tR = 24.5 min for minor isomer.

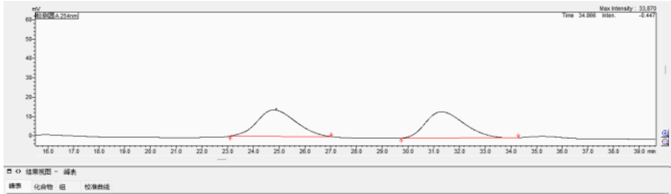

峰号	保留时间	面积	高度	标记	浓度	浓度单位	化合物ID号	化合物名	面积%
1	24.785	1422621	13662	M	50.265				50.265
2	31.311	1407627	13588	M	49.735				49.735
总计		2830249	27250		100.000				100.000

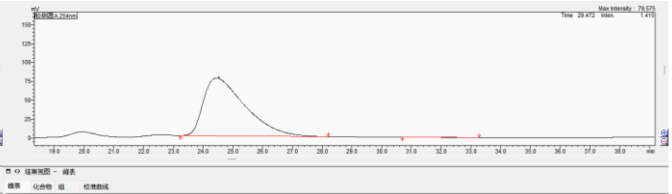

峰号	保留时间	面积	高度	标记	浓度	浓度单位	化合物ID号	化合物名	面积%
1	24.450	7460882	76587	M	99.449				99.449
2	31.780	41371	530	M	0.551				0.551
总计		7502253	77117		100.000				100.000

N-((2*R*,3*R*)-2-(benzyl(3-(4,4,5,5-tetramethyl-1,3,2-dioxaborolan-2-yl)benzyl)amino)-3-methyl-5-phenylpentyl)benzamide (3x)

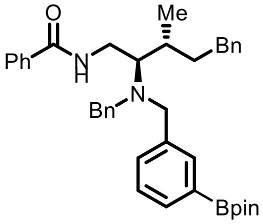

From (*E*)-*N*-(3-methyl-5-phenylpent-2-en-1-yl)benzamide (**1a**) (55.8 mg, 0.20 mmol, 1.0 equiv) and *O*-benzoyl-*N*-benzyl-*N*-(3-(4,4,5,5-tetramethyl-1,3,2-dioxaborolan-2-yl)benzyl)hydroxylamine (**2x**) (133.0 mg, 0.30 mmol, 1.5 equiv), the title compound was prepared following the general procedure **A** using $NiBr_2$·DME (6.2 mg, 10 mol%), **(*S*,*S*)-L12** (15.4 mg, 15 mol%), LiOH (9.6 mg, 0.40 mmol, 2.0 equiv), KI (33.2 mg, 0.20 mmol, 1.0 equiv), $Me(EtO)_2SiH$ (80 µL, 0.50 mmol, 2.5 equiv), Anhydrous *tert*-butyl alcohol (1.0 mL, 0.20 M). The reaction mixture was stirred for 12 h at 30 °C. The crude material was purified by flash column chromatography (petroleum ether/EtOAc = 3:1) to provide the title compound as a yellow oil in 92% yield (110.8 mg).

^{1}H NMR (400 MHz, $CDCl_3$) δ 7.65–7.56 (m, 2H), 7.51 (d, *J* = 7.6 Hz, 2H), 7.44–7.39 (m, 1H), 7.37–7.30 (m, 2H), 7.27–7.22 (m, 2H), 7.21–7.08 (m, 8H), 7.07–7.01 (m, 2H), 6.48 (d, *J* = 6.4 Hz, 1H), 3.89–3.71 (m, 2H), 3.60–3.50 (m, 1H), 3.49–3.35 (m, 2H), 3.34–3.21 (m, 1H), 2.75–2.59 (m, 2H), 2.57–2.47 (m, 1H), 2.08–1.97 (m, 1H), 1.75–1.68 (m, 1H), 1.58–1.46 (m, 1H), 1.21 (s, 12H), 1.03 (d, *J* = 6.7 Hz, 3H). **^{13}C NMR** (101 MHz, $CDCl_3$) δ 167.3, 142.2, 139.9, 139.2, 135.7, 134.7, 133.8, 132.0, 131.2, 129.2, 128.5, 128.5, 128.1, 127.2, 127.0, 125.9, 83.8, 61.2, 54.0, 53.7, 38.0, 37.5, 33.5, 30.4, 24.9, 24.9, 16.5. **HRMS** (ESI) m/z calculated for $C_{39}H_{47}BN_2O_3$ $[M+Na]^+$ 625.3572, found: 625.3588. **Optical rotation**: $[\alpha]^{20}_D$ = 9.93 (c = 1.0 g/L, $CHCl_3$). The absolute configuration was assigned by analogy to that of **4b**. **HPLC condition**: Chiral column OD-H, n-hexane/i-PrOH = 95:5, flow rate = 1 mL/min, wavelength = 254 nm, tR = 15.86 min for major isomer, tR = 22.08 min for minor isomer.

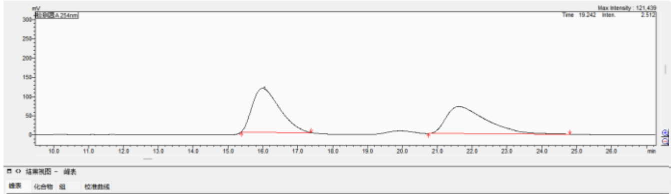

峰号	保留时间	面积	高度	标记	浓度	浓度单位	化合物ID号	化合物名	面积%
1	15.990	6202728	113918	M	52.358				52.358
2	21.624	5644100	70437	M	47.642				47.642
总计		11846827	184356		100.000				100.000

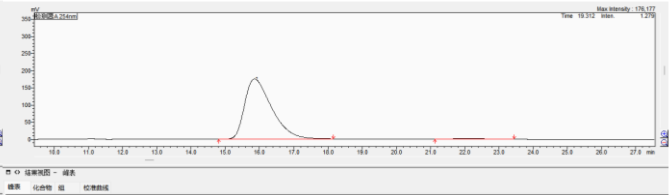

峰号	保留时间	面积	高度	标记	浓度	浓度单位	化合物ID号	化合物名	面积%
1	15.857	9554412	174761	M	98.753				98.753
2	22.082	120642	1770	M	1.247				1.247
总计		9675054	176530		100.000				100.000

N-((2*R*,3*R*)-2-(benzyl(3-chloro-4-fluorobenzyl)amino)-3-methyl-5-phenylpentyl)benzamide (3y)

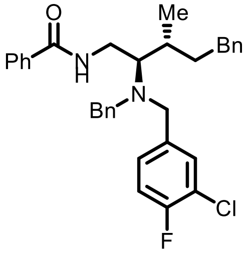

From (*E*)-*N*-(3-methyl-5-phenylpent-2-en-1-yl)benzamide (**1a**) (55.8 mg, 0.20 mmol, 1.0 equiv) and *O*-benzoyl-*N*-benzyl-*N*-(3-chloro-4-fluorobenzyl)hydroxylamine (**2y**) (110.7 mg, 0.30 mmol, 1.5 equiv), the title compound was prepared following the general procedure **A** using $NiBr_2$·DME (6.2 mg, 10 mol%), **(*S*,*S*)-L12** (15.4 mg, 15 mol%), LiOH (9.6 mg, 0.40 mmol, 2.0 equiv), KI (33.2 mg, 0.20 mmol, 1.0 equiv), $Me(EtO)_2SiH$ (80 μL, 0.50 mmol, 2.5 equiv), Anhydrous *tert*-butyl alcohol (1.0 mL, 0.20 M). The reaction mixture was stirred for 12 h at 30 °C. The crude material was purified by flash column chromatography (petroleum ether/EtOAc = 3:1) to provide the title compound as a yellow oil in 88% yield (93.0 mg).

^{1}H NMR (400 MHz, $CDCl_3$) δ 7.69–7.65 (m, 2H), 7.57–7.52 (m, 1H), 7.51–7.46 (m, 2H), 7.39–7.34 (m, 2H), 7.28–7.21 (m, 7H), 7.20–7.14 (m, 2H), 7.03–6.94 (m, 2H), 6.48–6.40 (m, 1H), 3.86–3.79 (m, 2H), 3.66–3.59 (m, 1H), 3.56–3.43 (m, 3H), 2.88–2.76 (m, 1H), 2.73–2.66 (m, 1H), 2.65–2.55 (m, 1H), 2.13–2.05 (m, 1H), 1.88–1.80 (m, 1H), 1.66–1.56 (m, 1H), 1.14 (d, *J* = 6.8 Hz, 3H); **^{13}C NMR** (101 MHz, $CDCl_3$) δ 167.3, 158.4, 156.0, 142.1, 139.5, 137.2 (d, *J* = 3.9 Hz), 134.5, 131.5, 131.0, 129.1, 128.7, 128.5, 128.5, 127.5, 126.8, 126.0, 121.0 (d, *J* = 17.8 Hz), 116.5 (d, *J* = 21.0 Hz), 61.9, 54.1, 53.0, 37.7, 37.5, 33.4, 30.8, 16.5; **^{19}F NMR** (376 MHz, $CDCl_3$) δ -117.56. **HRMS** (ESI) m/z calculated for $C_{33}H_{34}ClFN_2O$ $[M+H]^+$ 529.2417, found: 529.2417. **Optical rotation**: $[\alpha]^{20}_D$ = 9.50 (c = 1.0 g/L, $CHCl_3$). The absolute configuration was assigned by analogy to that of **4b**. **HPLC condition**: Chiral column OD-H, n-hexane/i-PrOH = 95:5, flow rate = 1 mL/min, wavelength = 254 nm, tR = 36.57 min for major isomer, tR = 52.92 min for minor isomer.

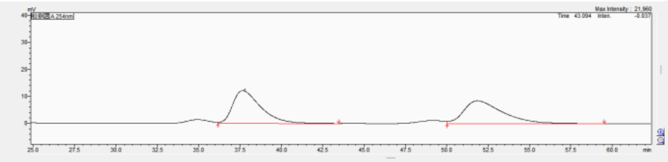

峰号	保留时间	面积	高度	标记	浓度	浓度单位	化合物ID号	化合物名	面积%
1	37.663	1433274	12149	M	50.659				50.659
2	51.858	1395997	8445	M	49.341				49.341
总计		2829271	20594		100.000				100.000

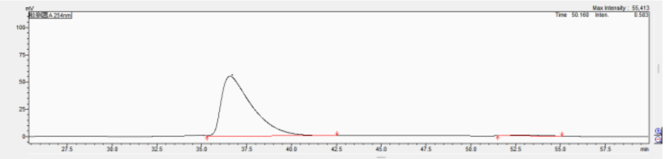

峰号	保留时间	面积	高度	标记	浓度	浓度单位	化合物ID号	化合物名	面积%
1	36.569	6555254	55002		98.595				98.595
2	52.924	93404	717	M	1.405				1.405
总计		6648657	55719		100.000				100.000

4-Methoxy-*N*-((2*R*,3*R*)-3-methyl-2-morpholino-5-phenylpentyl)benzamide (4a)

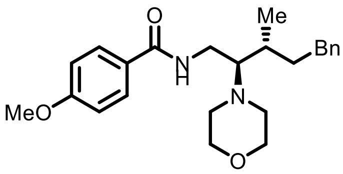

From (*E*)-4-methoxy-*N*-(3-methyl-5-phenylpent-2-en-1-yl)benzamide (**1x**) (61.8 mg, 0.20 mmol, 1.0 equiv) and morpholino benzoate (**2a**) (62.1 mg, 0.30 mmol, 1.5 equiv), the title compound was prepared following the general procedure **A** using NiBr$_2$·DME (6.2 mg, 10 mol%), **(*S*,*S*)-L12** (15.4 mg, 15 mol%), LiOH (9.6 mg, 0.40 mmol, 2.0 equiv), KI (33.2 mg, 0.20 mmol, 1.0 equiv), Me(EtO)$_2$SiH (80 μL, 0.50 mmol, 2.5 equiv), Anhydrous *tert*-butyl alcohol (1.0 mL, 0.20 M). The reaction mixture was stirred for 12 h at 30 °C. The crude material was purified by flash column chromatography (petroleum ether/EtOAc = 3:1) to provide the title compound as a yellow oil in 81% yield (64.2 mg).

^{1}H NMR (400 MHz, CDCl$_3$) δ 7.80 (d, *J* = 8.3 Hz, 2H), 7.38–7.31 (m, 2H), 7.29-7.22 (m, 3H), 7.00 (d, *J* = 8.3 Hz, 2H), 6.91 (s, 1H), 3.92 (s, 3H), 3.82–3.66 (m, 5H), 3.40-3.29 (m, 1H), 2.88–2.72 (m, 3H), 2.69–2.56 (m, 4H), 2.04–1.93 (m, 1H), 1.91-1.79 (m, 1H), 1.72–1.60 (m, 1H), 1.09 (d, *J* = 6.7 Hz, 3H); **^{13}C NMR** (101 MHz, CDCl$_3$) δ 166.7, 162.1, 142.2, 128.6, 128.4, 128.4, 127.0, 125.9, 113.9, 67.9, 67.1, 55.5, 49.6, 37.4, 36.8, 33.4, 31.1, 16.5. **HRMS** (ESI) m/z calculated for $C_{24}H_{32}N_2O_3$ $[M+Na]^+$ 419.2305, found: 419.2304. **Optical rotation**: $[\alpha]^{20}_D$ = -93.6 (c = 1.0 g/L, CHCl$_3$). The absolute configuration was assigned by analogy to that of **4b**. **HPLC condition**: Chiral column AD-H, n-hexane/i-PrOH = 95:5, flow rate = 1 mL/min, wavelength = 254 nm, tR = 67.97 min for major isomer, tR = 75.74 min for minor isomer.

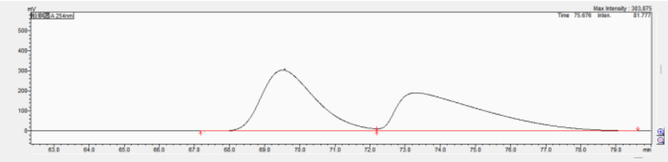

峰号	保留时间	面积	高度	标记	浓度	浓度单位	化合物ID号	化合物名	面积%
1	69.514	32439010	303704	M	49.797				49.797
2	73.306	32703808	190367	V M	50.203				50.203
总计		65142818	494071		100.000				100.000

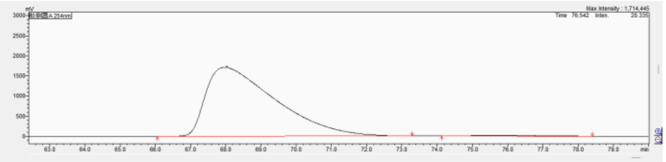

峰号	保留时间	面积	高度	标记	浓度	浓度单位	化合物ID号	化合物名	面积%
1	67.969	235067545	1712734	M	98.753				98.753
2	75.735	2968341	24532	M	1.247				1.247
总计		238035886	1737266		100.000				100.000

N-((2*R*,3*R*)-3-methyl-2-morpholino-5-phenylpentyl)-[1,1'-biphenyl]-4-carboxamide (4b)

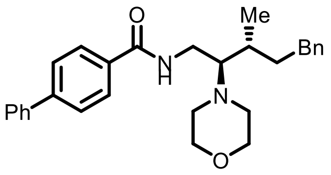

From (*E*)-*N*-(3-methyl-5-phenylpent-2-en-1-yl)-[1,1'-biphenyl]-4-carboxamide (**1b**) (71.0 mg, 0.20 mmol, 1.0 equiv) and morpholino benzoate (**2a**) (62.1 mg, 0.30 mmol, 1.5 equiv), the title compound was prepared following the general procedure **A** using $NiBr_2$·DME (6.2 mg, 10 mol%), **(*S*,*S*)-L12** (15.4 mg, 15 mol%), LiOH (9.6 mg, 0.40 mmol, 2.0 equiv), KI (33.2 mg, 0.20 mmol, 1.0 equiv), $Me(EtO)_2SiH$ (80 µL, 0.50 mmol, 2.5 equiv), Anhydrous *tert*-butyl alcohol (1.0 mL, 0.20 M). The reaction mixture was stirred for 12 h at 30 °C. The crude material was purified by flash column chromatography (petroleum ether/EtOAc = 3:1) to provide the title compound as a white solid in 75% yield (66.3 mg).

^{1}H NMR (400 MHz, $CDCl_3$) δ 7.85 (d, *J* = 8.1 Hz, 2H), 7.71–7.66 (m, 2H), 7.65–7.60 (m, 2H), 7.51–7.44 (m, 2H), 7.42–7.36 (m, 1H), 7.33–7.27 (m, 2H), 7.23-7.17 (m, 3H), 7.00–6.93 (m, 1H), 3.78-3.62 (m, 5H), 3.38-3.26 (m, 1H), 2.80-2.68 (m, 3H), 2.65–2.52 (m, 4H), 1.98–1.88 (m, 1H), 1.87–1.76 (m, 1H), 1.68-1.55 (m, 1H), 1.05 (d, *J* = 6.9 Hz, 3H); **^{13}C NMR** (101 MHz, $CDCl_3$) δ 166.8, 144.2, 142.1, 140.1, 133.4, 129.0, 128.5, 128.4, 128.0, 127.4, 127.2, 125.9, 67.9, 67.1, 49.6, 37.4, 36.9, 33.4, 31.2, 16.5. **HRMS** (ESI) m/z calculated for $C_{29}H_{34}N_2O_2$ $[M+Na]^+$ 465.2512, found: 465.2512. **Optical rotation**: $[\alpha]^{20}_D$ = -41.9 (c = 1.0 g/L, $CHCl_3$). The absolute configuration was assigned by analogy to that of **4b**. **HPLC condition**: Chiral column OD-H, n-hexane/i-PrOH = 90:10, flow rate = 1 mL/min, wavelength = 254 nm, tR = 29.14 min for major isomer, tR = 34.01 min for minor isomer.

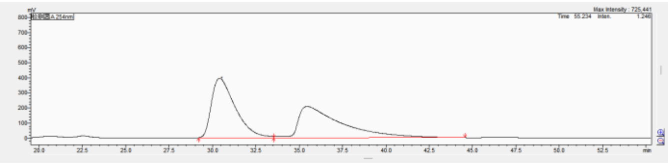

结果视图 - 峰表

峰号	保留时间	面积	高度	标记	浓度	浓度单位	化合物ID号	化合物名	面积%
1	30.402	37336976	393668	M	50.975				50.975
2	35.452	35909354	207014	V M	49.025				49.025
总计		73246329	600682		100.000				100.000

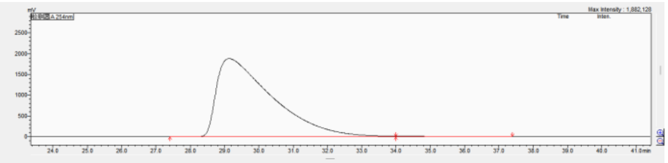

结果视图 - 峰表

峰号	保留时间	面积	高度	标记	浓度	浓度单位	化合物ID号	化合物名	面积%
1	29.135	211861434	1881238	M	99.585				99.585
2	34.013	882268	12079	V M	0.415				0.415
总计		212743702	1893316		100.000				100.000

***N*-((2*R*,3*R*)-3-methyl-2-morpholino-5-phenylpentyl)-4-(trifluoromethyl)benzamide (4c)**

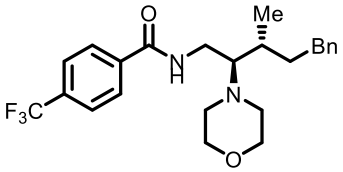

From (*E*)-*N*-(3-methyl-5-phenylpent-2-en-1-yl)-4-(trifluoromethyl)benzamide (**1c**) (69.4 mg, 0.20 mmol, 1.0 equiv) and morpholino benzoate (**2a**) (62.1 mg, 0.30 mmol, 1.5 equiv), the title compound was prepared following the general procedure **A** using $NiBr_2$·DME (6.2 mg, 10 mol%), **(*S*,*S*)-L12** (15.4 mg, 15 mol%), LiOH (9.6 mg, 0.40 mmol, 2.0 equiv), KI (33.2 mg, 0.20 mmol, 1.0 equiv), $Me(EtO)_2SiH$ (80 µL, 0.50 mmol, 2.5 equiv), Anhydrous *tert*-butyl

alcohol (1.0 mL, 0.20 M). The reaction mixture was stirred for 12 h at 30 °C. The crude material was purified by flash column chromatography (petroleum ether/EtOAc = 3:1) to provide the title compound as a yellow oil in 84% yield (72.9 mg).

^{1}H NMR (400 MHz, $CDCl_3$) δ 7.93 (d, *J* = 8.1 Hz, 2H), 7.78 (d, *J* = 8.1 Hz, 2H), 7.40–7.31 (m, 2H), 7.29–7.20 (m, 3H), 7.07 (s, 1H), 3.82–3.66 (m, 5H), 3.42–3.31 (m, 1H), 2.86–2.73 (m, 3H), 2.72–2.57 (m, 4H), 2.06–1.93 (m, 1H), 1.92–1.79 (m, 1H), 1.73–1.60 (m, 1H), 1.09 (d, *J* = 6.8 Hz, 3H); **^{13}C NMR** (101 MHz, $CDCl_3$) δ 165.8, 142.0, 138.0, 133.2 (q, *J* = 32.8 Hz), 128.5, 128.4, 127.3, 126.0, 125.8 (q, *J* = 3.3 Hz), 123.7 (q, *J* = 272.5 Hz), 67.9, 67.0, 49.6, 37.5, 37.0, 33.4, 31.0, 16.5; **^{19}F NMR** (376 MHz, $CDCl_3$) δ -62.90. **HRMS** (ESI) m/z calculated for $C_{24}H_{29}F_3N_2O_2$ $[M+H]^+$ 435.2254, found: 435.2254. **Optical rotation**: $[\alpha]^{20}_D$ = -123 (c = 1.0 g/L, $CHCl_3$). The absolute configuration was assigned by analogy to that of **4b**. **HPLC condition**: Chiral column AD-H, n-hexane/i-PrOH = 95:5, flow rate = 1 mL/min, wavelength = 254 nm, tR = 24.91 min for major isomer, tR = 34.29 min for minor isomer.

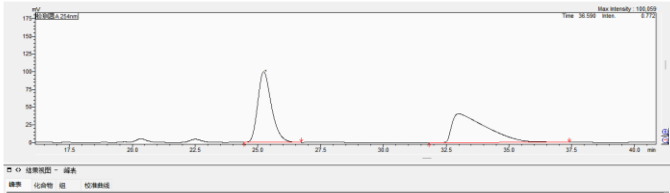

峰号	保留时间	面积	高度	标记	浓度	浓度单位	化合物ID号	化合物名	面积%
1	25.232	3808031	99819	M	50.336				50.336
2	32.994	3757197	40479	M	49.664				49.664
总计		7565228	140298		100.000				100.000

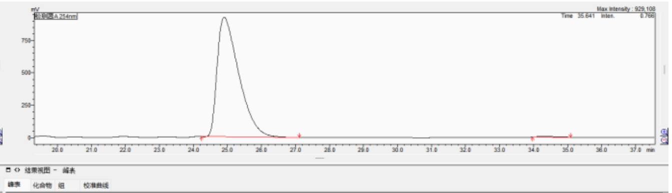

峰号	保留时间	面积	高度	标记	浓度	浓度单位	化合物ID号	化合物名	面积%
1	24.906	40864121	921876	M	99.528				99.528
2	34.287	193788	4824	M	0.472				0.472
总计		41057909	926701		100.000				100.000

4-Cyano-*N*-((2*R*,3*R*)-3-methyl-2-morpholino-5-phenylpentyl)benzamide (4d)

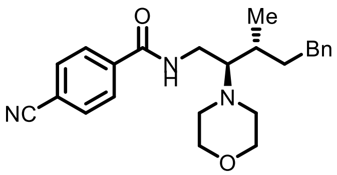

From (*E*)-4-cyano-*N*-(3-methyl-5-phenylpent-2-en-1-yl)benzamide (**1d**) (60.8 mg, 0.20 mmol, 1.0 equiv) and morpholino benzoate (**2a**) (62.1 mg, 0.30 mmol, 1.5 equiv), the title compound was prepared following the general procedure **A** using $NiBr_2 \cdot DME$ (6.2 mg, 10 mol%), **(*S*,*S*)-L12** (15.4 mg, 15 mol%), LiOH (9.6 mg, 0.40 mmol, 2.0 equiv), KI (33.2 mg, 0.20 mmol, 1.0 equiv), $Me(EtO)_2SiH$ (80 µL, 0.50 mmol, 2.5 equiv), Anhydrous *tert*-butyl alcohol (1.0 mL, 0.20 M). The reaction mixture was stirred for 12 h at 30 °C. The crude material was purified by flash column chromatography (petroleum ether/EtOAc = 3:1) to provide the title compound as a yellow oil in 72% yield (56.3 mg).

^{1}H NMR (400 MHz, $CDCl_3$) δ 7.89 (d, *J* = 8.0 Hz, 2H), 7.82–7.75 (m, 2H), 7.36–7.27 (m, 2H), 7.27–7.16 (m, 3H), 7.04 (s, 1H), 3.78–3.64 (m, 5H), 3.38–3.27 (m, 1H), 2.82–2.69 (m, 3H), 2.66–2.54 (m, 4H), 2.04–1.90 (m, 1H), 1.88–1.75 (m, 1H), 1.70–1.57 (m, 1H), 1.06 (d, *J* = 6.9 Hz, 3H); **^{13}C NMR** (101 MHz,

$CDCl_3$) δ 165.2, 141.9, 138.6, 132.6, 128.5, 128.4, 127.5, 126.0, 118.1, 115.0, 67.8, 49.5, 37.5, 37.0, 33.3, 30.9, 29.3, 16.5. **HRMS** (ESI) m/z calculated for $C_{24}H_{29}N_3O_2$ $[M+H]^+$ 392.2333, found: 392.2333. **Optical rotation**: $[\alpha]^{20}_D$ = -50.8 (c = 1.0 g/L, $CHCl_3$). The absolute configuration was assigned by analogy to that of **4b**. **HPLC condition**: Chiral column OD-H, n-hexane/i-PrOH = 90:10, flow rate = 1 mL/min, wavelength = 254 nm, tR = 49.08 min for major isomer, tR = 42.66 min for minor isomer.

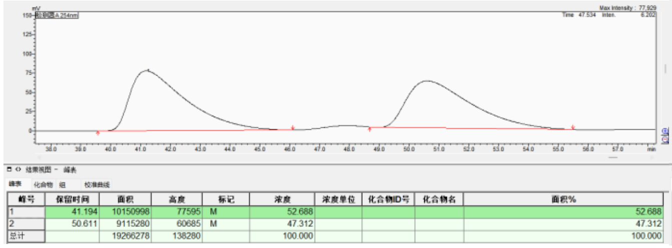

峰号	保留时间	面积	高度	标记	浓度	浓度单位	化合物ID号	化合物名	面积%
1	41.194	10150998	77595	M	52.688				52.688
2	50.611	9115280	60685	M	47.312				47.312
总计		19266278	138280		100.000				100.000

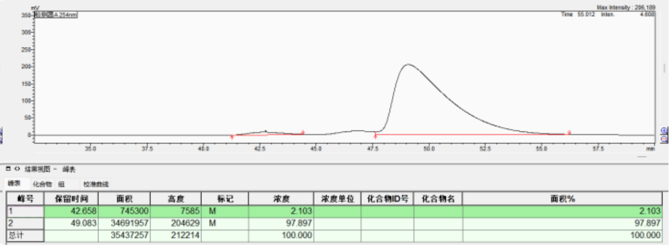

峰号	保留时间	面积	高度	标记	浓度	浓度单位	化合物ID号	化合物名	面积%
1	42.658	745300	7585	M	2.103				2.103
2	49.083	34691957	204629	M	97.897				97.897
总计		35437257	212214		100.000				100.000

N-((2*R*,3*R*)-3-methyl-2-morpholino-5-phenylpentyl)-4-nitrobenzamide (4e)

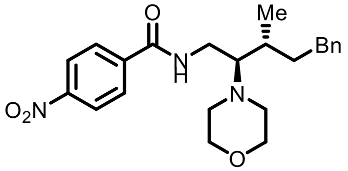

From (*E*)-*N*-(3-methyl-5-phenylpent-2-en-1-yl)-4-nitrobenzamide (**1e**) (64.8 mg, 0.20 mmol, 1.0 equiv) and morpholino benzoate (**2a**) (62.1 mg, 0.30 mmol, 1.5 equiv), the title compound was prepared following the general procedure **A** using $NiBr_2$·DME (6.2 mg, 10 mol%), **(*S*,*S*)-L12** (15.4 mg, 15 mol%), LiOH (9.6 mg, 0.40 mmol, 2.0 equiv), KI (33.2 mg, 0.20 mmol, 1.0 equiv), $Me(EtO)_2SiH$ (80 µL, 0.50 mmol, 2.5 equiv), Anhydrous *tert*-butyl alcohol (1.0 mL, 0.20 M). The reaction mixture was stirred for 12 h at 30 °C. The crude material was purified by flash column chromatography (petroleum ether/EtOAc = 3:1) to provide the title compound as a yellow oil in 67% yield (55.1 mg).

^{1}H NMR (400 MHz, $CDCl_3$) δ 8.34–8.25 (m, 2H), 7.91 (d, *J* = 8.5 Hz, 2H), 7.33–7.24 (m, 2H), 7.23–7.14 (m, 3H), 7.03 (d, *J* = 6.6 Hz, 1H), 3.76–3.61 (m, 5H), 3.35–3.24 (m, 1H), 2.78–2.68 (m, 3H), 2.65–2.51 (m, 4H), 2.02–1.88 (m, 1H), 1.85–1.72 (m, 1H), 1.67–1.53 (m, 1H), 1.03 (d, *J* = 6.9 Hz, 3H); **^{13}C NMR** (101 MHz, $CDCl_3$) δ 165.0, 149.6, 141.9, 140.3, 128.5, 128.4, 128.0, 126.0, 124.0, 67.9, 66.9, 49.5, 37.5, 37.1, 33.4, 30.9, 16.5. **HRMS** (ESI) m/z calculated for $C_{23}H_{29}N_3O_4$ $[M+H]^+$ 412.2231, found: 412.2232. **Optical rotation**: $[\alpha]^{20}_D$ = -30.0 (c = 1.0 g/L, $CHCl_3$). The absolute configuration was assigned by analogy to that of **4b**. **HPLC condition**: Chiral column AD-H, n-hexane/i-PrOH = 90:10, flow rate = 1 mL/min, wavelength = 254 nm, tR = 21.26 min for major isomer, tR = 33.66 min for minor isomer.

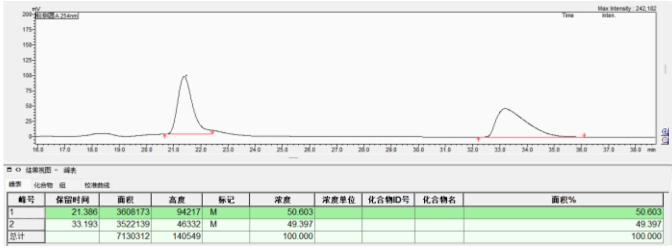

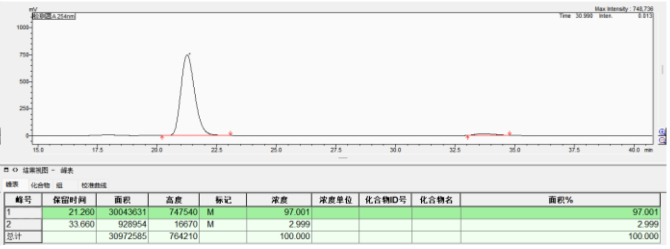

N-((2R,3R)-3-methyl-2-morpholino-5-phenylpentyl)-2-naphthamide (4f)

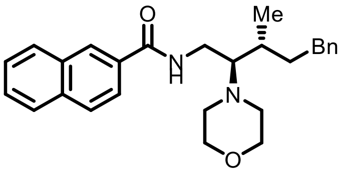

From (E)-N-(3-methyl-5-phenylpent-2-en-1-yl)-2-naphthamide (**1f**) (65.8 mg, 0.20 mmol, 1.0 equiv) and morpholino benzoate (**2a**) (62.1 mg, 0.30 mmol, 1.5 equiv), the title compound was prepared following the general procedure **A** using $NiBr_2$·DME (6.2 mg, 10 mol%), **(*S*,*S*)-L12** (15.4 mg, 15 mol%), LiOH (9.6 mg, 0.40 mmol, 2.0 equiv), KI (33.2 mg, 0.20 mmol, 1.0 equiv), $Me(EtO)_2SiH$ (80 μL, 0.50 mmol, 2.5 equiv), Anhydrous *tert*-butyl alcohol (1.0 mL, 0.20 M). The reaction mixture was stirred for 12 h at 30 °C. The crude material was purified by flash column chromatography (petroleum ether/EtOAc = 3:1) to provide the title compound as a yellow oil in 78% yield (64.9 mg).

^{1}H NMR (400 MHz, $CDCl_3$) δ 8.34 (s, 1H), 8.00–7.90 (m, 3H), 7.89–7.83 (m, 1H), 7.65–7.55 (m, 2H), 7.37–7.28 (m, 2H), 7.27–7.20 (m, 3H), 7.12 (s, 1H), 3.85–3.68 (m, 5H), 3.45–3.30 (m, 1H), 2.86–2.72 (m, 3H), 2.70–2.58 (m, 4H), 2.04–1.92 (m, 1H), 1.90–1.80 (m, 1H), 1.70–1.60 (m, 1H), 1.09 (d, J = 6.8 Hz, 3H); **^{13}C NMR** (101 MHz, $CDCl_3$) δ 167.1, 142.1, 134.7, 132.7, 131.9, 129.0, 128.6, 128.5, 128.4, 127.8, 127.7, 127.4, 126.8, 125.9, 123.4, 67.9, 67.1, 49.6, 37.5, 37.0, 33.4, 31.2, 16.5. **HRMS** (ESI) m/z calculated for $C_{27}H_{32}N_2O_2$ $[M+Na]^+$ 439.2354, found: 439.2352. **Optical rotation**: $[\alpha]^{20}_D$ = -36.9 (c = 1.0 g/L, $CHCl_3$). The absolute configuration was assigned by analogy to that of **4b**. **HPLC condition**: Chiral column AD-H, n-hexane/i-PrOH = 90:10, flow rate = 1 mL/min, wavelength = 254 nm, tR = 37.81 min for major isomer, tR = 29.88 min for minor isomer.

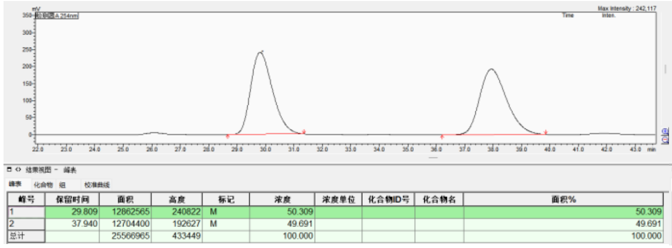

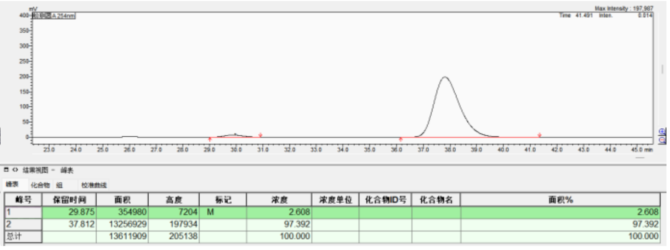

N-((2R,3R)-3-methyl-2-morpholino-5-phenylpentyl)thiophene-2-carboxamide (4g)

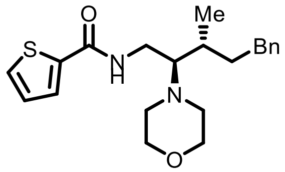

From (*E*)-*N*-(3-methyl-5-phenylpent-2-en-1-yl)thiophene-2-carboxamide (**1g**) (57.0 mg, 0.20 mmol, 1.0 equiv) and morpholino benzoate (**2a**) (62.1 mg, .30 mmol, 1.5 equiv), the title compound was prepared following the general procedure **A** using $NiBr_2$·DME (6.2 mg, 10 mol%), **(*S*,*S*)-L12** (15.4 mg, 15 mol%), LiOH (9.6 mg, 0.40 mmol, 2.0 equiv), KI (33.2 mg, 0.20 mmol, 1.0 equiv), $Me(EtO)_2SiH$ (80 µL, 0.50 mmol, 2.5 equiv), Anhydrous *tert*-butyl alcohol (1.0 mL, 0.20 M). The reaction mixture was stirred for 12 h at 30 °C. The crude material was purified by flash column chromatography (petroleum ether/EtOAc = 3:1) to provide the title compound as a yellow oil in 85% yield (63.3 mg).

^{1}H NMR (400 MHz, $CDCl_3$) δ 7.57 (d, J = 3.7 Hz, 1H), 7.53 (d, J = 5.0 Hz, 1H), 7.37–7.33 (m, 2H), 7.28–7.23 (m, 3H), 7.17–7.14 (m, 1H), 6.90 (s, 1H), 3.82–3.71 (m, 5H), 3.35–3.26 (m, 1H), 2.79 (s, 3H), 2.68–2.58 (m, 4H), 2.01–1.95 (m, 1H), 1.88–1.84 (m, 1H), 1.70–1.62 (m, 1H), 1.08 (d, J = 6.8 Hz, 3H); **^{13}C NMR** (101 MHz, $CDCl_3$) δ 161.6, 142.1, 139.1, 129.6, 128.5, 128.4, 128.1, 127.8, 125.9, 67.9, 67.0, 49.5, 37.4, 36.8, 33.3, 31.2, 16.5. **HRMS** (ESI) m/z calculated for $C_{21}H_{28}N_2O_2S$ $[M+H]^+$ 373.1944, found: 373.1940. **Optical rotation**: $[\alpha]^{20}_D$ = -44.5 (c = 1.0 g/L, $CHCl_3$). The absolute configuration was assigned by analogy to that of **4b**. **HPLC condition**: Chiral column IC, n-hexane/i-PrOH = 85:15, flow rate = 1 mL/min, wavelength = 254 nm, tR = 39.85 min for major isomer, tR = 34.41 min for minor isomer.

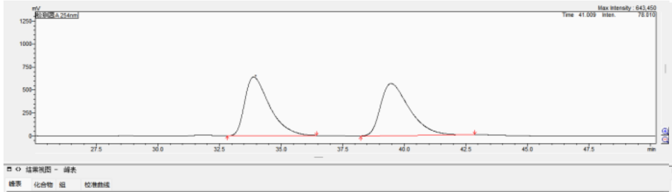

峰号	保留时间	面积	高度	标记	浓度	浓度单位	化合物ID号	化合物名	面积%
1	33.885	44914355	641004	M	49.348				49.348
2	39.470	46101180	567780	M	50.652				50.652
总计		91015534	1208785		100.000				100.000

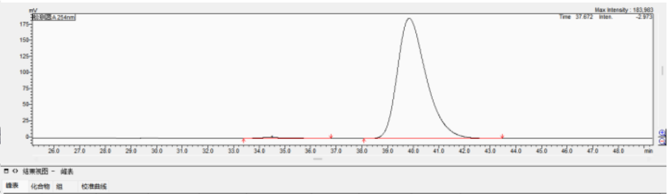

峰号	保留时间	面积	高度	标记	浓度	浓度单位	化合物ID号	化合物名	面积%
1	34.412	119920	1433		0.815				0.815
2	39.848	14590761	186888		99.185				99.185
总计		14710682	188321		100.000				100.000

N-((2R,3R)-3-methyl-2-morpholino-5-phenylpentyl)pivalamide (4h)

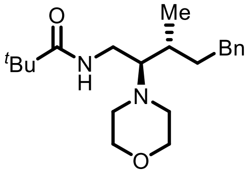

From (*E*)-*N*-(3-methyl-5-phenylpent-2-en-1-yl)pivalamide (**1h**) (51.8 mg, 0.20 mmol, 1.0 equiv) and morpholino benzoate (**2a**) (62.1 mg, 0.30 mmol, 1.5 equiv), the title compound was prepared following the general procedure **A** using $NiBr_2$·DME (6.2 mg, 10 mol%), **(*S*,*S*)-L12** (15.4 mg, 15 mol%), LiOH (9.6 mg, 0.40 mmol, 2.0 equiv), KI (33.2 mg, 0.20 mmol, 1.0 equiv), $Me(EtO)_2SiH$ (80 µL, 0.50 mmol, 2.5 equiv), Anhydrous *tert*-butyl alcohol (1.0 mL, 0.20 M). The reaction mixture was stirred for 12 h at 30 °C. The

crude material was purified by flash column chromatography (petroleum ether/EtOAc = 2:1) to provide the title compound as a yellow oil in 75% yield (51.9 mg).

^{1}H NMR (400 MHz, $CDCl_3$) δ 7.41–7.32 (m, 2H), 7.30–7.20 (m, 3H), 6.51 (s, 1H), 3.80–3.66 (m, 4H), 3.58–3.50 (m, 1H), 3.20–3.09 (m, 1H), 2.83–2.71 (m, 3H), 2.68–2.57 (m, 3H), 2.54–2.45 (m, 1H), 1.99–1.90 (m, 1H), 1.87–1.79 (m, 1H), 1.69–1.56 (m, 1H), 1.27 (s, 9H), 1.04 (d, J = 6.8 Hz, 3H). **^{13}C NMR** (101 MHz, $CDCl_3$) δ 178.2, 142.2, 128.4, 128.4, 125.9, 67.9, 66.9, 49.6, 37.3, 36.5, 33.3, 31.3, 27.6, 27.1, 16.4. **HRMS** (ESI) m/z calculated for $C_{21}H_{34}N_2O_2$ $[M+H]^+$ 347.2693, found: 347.2693. **Optical rotation**: $[\alpha]^{20}_D$ = -169 (c = 1.0 g/L, $CHCl_3$). The absolute configuration was assigned by analogy to that of **4b**. **HPLC condition**: Chiral column AD-H, n-hexane/i-PrOH = 97:3, flow rate = 1 mL/min, wavelength = 254 nm, tR = 13.44 min for major isomer, tR = 15.26 min for minor isomer.

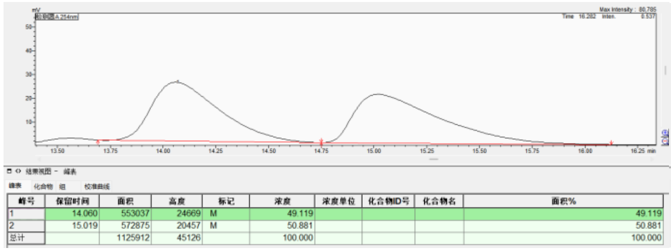

峰号	保留时间	面积	高度	标记	浓度	浓度单位	化合物ID号	化合物名	面积%
1	14.060	553037	24669	M	49.119				49.119
2	15.019	572875	20457	M	50.881				50.881
总计		1125912	45126		100.000				100.000

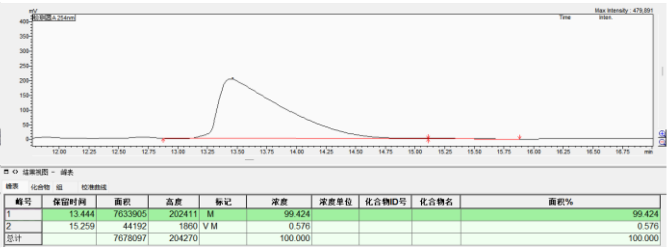

峰号	保留时间	面积	高度	标记	浓度	浓度单位	化合物ID号	化合物名	面积%
1	13.444	7633905	202411	M	99.424				99.424
2	15.259	44192	1860	V M	0.576				0.576
总计		7678097	204270		100.000				100.000

(3*R*,5*R*,7*R*)-*N*-((2*R*,3*R*)-3-methyl-2-morpholino-5-phenylpentyl)adamantane-1-carboxamide (4i)

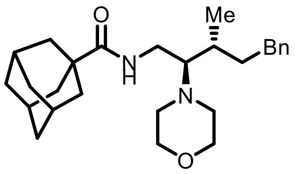

From (1*s*,3*s*)-*N*-((*E*)-3-methyl-5-phenylpent-2-en-1-yl)adamantane-1-carboxamide (**1i**) (67.4 mg, 0.20 mmol, 1.0 equiv) and morpholino benzoate (**2a**) (62.1 mg, 0.30 mmol, 1.5 equiv), the title compound was prepared following the general procedure **A** using $NiBr_2$·DME (6.2 mg, 10 mol%), **(*S*,*S*)-L12** (15.4 mg, 15 mol%), LiOH (9.6 mg, 0.40 mmol, 2.0 equiv), KI (33.2 mg, 0.20 mmol, 1.0 equiv), $Me(EtO)_2SiH$ (80 μL, 0.50 mmol, 2.5 equiv), Anhydrous *tert*-butyl alcohol (1.0 mL, 0.20 M). The reaction mixture was stirred for 12 h at 30 °C. The crude material was purified by flash column chromatography (petroleum ether/EtOAc = 2:1) to provide the title compound as a yellow oil in 64% yield (54.3 mg).

^{1}H NMR (400 MHz, $CDCl_3$) δ 7.27–7.17 (m, 2H), 7.16–7.07 (m, 3H), 6.29 (s, 1H), 3.68–3.54 (m, 4H), 3.46–3.35 (m, 1H), 3.08–2.97 (m, 1H), 2.69–2.57 (m, 3H), 2.55–2.44 (m, 3H), 2.41–2.32 (m, 1H), 2.02–1.96 (m, 3H), 1.81–1.76 (m, 6H), 1.73–1.60 (m, 8H), 1.54–1.42 (m, 1H), 0.91 (d, J = 6.7 Hz, 3H). **^{13}C NMR** (101 MHz, $CDCl_3$) δ 177.8, 142.2, 128.4, 128.4, 125.8, 67.9, 67.0, 49.6, 40.6, 39.4, 37.2, 36.6, 36.3, 33.3, 31.3, 28.2, 16.4. **HRMS** (ESI) m/z calculated for $C_{27}H_{40}N_2O_2$ $[M+H]^+$ 425.3163, found: 425.3164.

Optical rotation: $[\alpha]^{20}_D$ = -42.4 (c = 1.0 g/L, $CHCl_3$). The absolute configuration was assigned by analogy to that of **4b**. **HPLC condition**: Chiral column AD-H, n-hexane/i-PrOH = 95:5, flow rate = 1 mL/min, wavelength = 254 nm, tR = 17.93 min for major isomer, tR = 19.50 min for minor isomer.

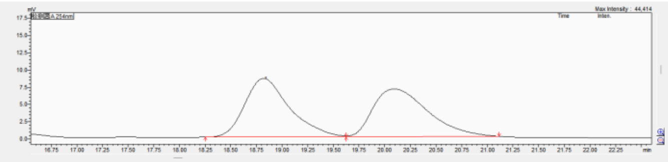

峰号	保留时间	面积	高度	标记	浓度	浓度单位	化合物ID号	化合物名	面积%
1	18.820	255937	8458	M	50.055				50.055
2	20.094	255378	6892	V M	49.945				49.945
总计		511315	15350		100.000				100.000

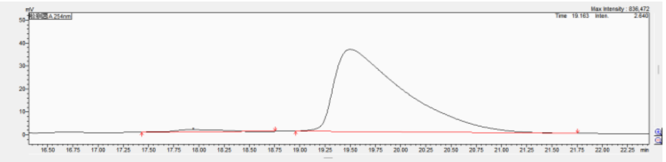

峰号	保留时间	面积	高度	标记	浓度	浓度单位	化合物ID号	化合物名	面积%
1	17.925	26647	953	M	1.535				1.535
2	19.501	1709361	35828	M	98.465				98.465
总计		1736008	36781		100.000				100.000

***N*-((2*R*,3*R*)-4-(4-methoxyphenyl)-3-methyl-2-morpholinobutyl)benzamide (4j)**

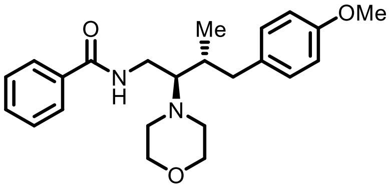

From (*E*)-*N*-(4-(4-methoxyphenyl)-3-methylbut-2-en-1-yl)benzamide (**1j**) (59.0 mg, 0.20 mmol, 1.0 equiv) and morpholino benzoate (**2a**) (62.1 mg, 0.30 mmol, 1.5 equiv), the title compound was prepared following the general procedure **A** using $NiBr_2$·DME (6.2 mg, 10 mol%), **(*S*,*S*)-L12** (15.4 mg, 15 mol%), LiOH (9.6 mg, 0.40 mmol, 2.0 equiv), KI (33.2 mg, 0.20 mmol, 1.0 equiv), $Me(EtO)_2SiH$ (80 µL, 0.50 mmol, 2.5 equiv), Anhydrous *tert*-butyl alcohol (1.0 mL, 0.20 M). The reaction mixture was stirred for 12 h at 30 °C. The crude material was purified by flash column chromatography (petroleum ether/EtOAc = 3:1) to provide the title compound as a yellow oil in 63% yield (48.2 mg).

^{1}H NMR (400 MHz, $CDCl_3$) δ 7.79 (d, *J* = 7.5 Hz, 2H), 7.62–7.54 (m, 1H), 7.51–7.39 (m, 2H), 7.09 (d, *J* = 8.3 Hz, 2H), 6.82 (d, *J* = 8.3 Hz, 2H), 3.78 (s, 3H), 3.75–3.66 (m, 5H), 3.46–3.32 (m, 1H), 2.82–2.73 (m, 2H), 2.71–2.61 (m, 2H), 2.57–2.44 (m, 3H), 2.27–2.14 (m, 1H), 0.99 (d, *J* = 6.8 Hz, 3H); **^{13}C NMR** (101 MHz, $CDCl_3$) δ 167.4, 158.0, 133.2, 131.5, 130.1, 128.7, 128.4, 126.9, 113.8, 67.3, 66.0, 55.3, 49.3, 41.2, 36.1, 33.2, 16.7. **HRMS** (ESI) m/z calculated for $C_{23}H_{30}N_2O_3$ $[M+H]^+$ 383.2329, found: 383.2329. **Optical rotation**: $[\alpha]^{20}_D$ = -124 (c = 1.0 g/L, $CHCl_3$). The absolute configuration was assigned by analogy to that of **4b**. **HPLC condition**: Chiral column OD-H, n-hexane/i-PrOH = 95:5, flow rate = 1 mL/min, wavelength = 254 nm, tR = 46.30 min for major isomer, tR = 56.38 min for minor isomer.

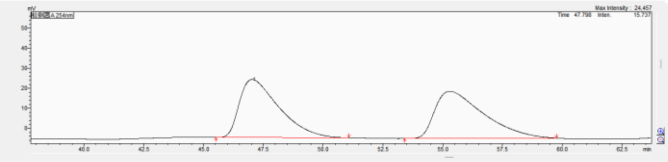

峰号	保留时间	面积	高度	标记	浓度	浓度单位	化合物ID号	化合物名	面积%
1	47.029	3345947	29038		50.080				50.080
2	55.304	3335236	23534	M	49.920				49.920
总计		6681183	52572		100.000				100.000

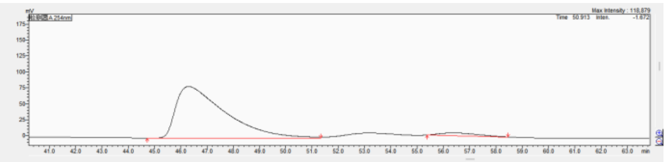

峰号	保留时间	面积	高度	标记	浓度	浓度单位	化合物ID号	化合物名	面积%
1	46.298	10539179	80754	M	95.984				95.984
2	56.376	441016	4376	M	4.016				4.016
总计		10980195	85131		100.000				100.000

***N*-((2*R*,3*R*)-4-(4-fluorophenyl)-3-methyl-2-morpholinobutyl)benzamide (4k)**

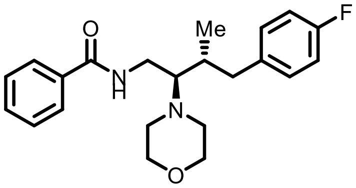

From (*E*)-*N*-(4-(4-fluorophenyl)-3-methylbut-2-en-1-yl)benzamide (**1k**) (56.6 mg, 0.20 mmol, 1.0 equiv) and morpholino benzoate (**2a**) (62.1 mg, 0.30 mmol, 1.5 equiv), the title compound was prepared following the general procedure **A** using $NiBr_2$·DME (6.2 mg, 10 mol%), **(*S*,*S*)-L12** (15.4 mg, 15 mol%), LiOH (9.6 mg, 0.40 mmol, 2.0 equiv), KI (33.2 mg, 0.20 mmol, 1.0 equiv), $Me(EtO)_2SiH$ (80 µL, 0.50 mmol, 2.5 equiv), Anhydrous *tert*-butyl alcohol (1.0 mL, 0.20 M). The reaction mixture was stirred for 12 h at 30 °C. The crude material was purified by flash column chromatography (petroleum ether/EtOAc = 3:1) to provide the title compound as a yellow oil in 45% yield (33.3 mg).

^{1}H NMR (400 MHz, $CDCl_3$) δ 7.77 (d, *J* = 7.4 Hz, 2H), 7.54–7.48 (m, 1H), 7.47–7.39 (m, 2H), 7.16–7.05 (m, 2H), 7.01–6.92 (m, 2H), 6.89 (s, 1H), 3.80–3.60 (m, 5H), 3.40–3.26 (m, 1H), 2.76–2.66 (m, 3H), 2.56–2.47 (m, 2H), 2.50–2.41 (m, 1H), 2.24–2.10 (m, 1H), 0.96 (d, *J* = 6.8 Hz, 3H); **^{13}C NMR** (101 MHz, $CDCl_3$) δ 167.2, 161.4 (d, *J* = 244.0 Hz), 136.1, 134.7, 131.5, 130.5 (d, *J* = 7.7 Hz), 128.7, 126.8, 115.2 (d, *J* = 21.1 Hz), 67.8, 65.7, 49.4, 41.3, 36.3, 33.4, 16.6; **^{19}F NMR** (376 MHz, $CDCl_3$) δ -62.92. **HRMS** (ESI) m/z calculated for $C_{22}H_{27}FN_2O_2$ $[M+H]^+$ 371.2130, found: 371.2131. **Optical rotation**: $[\alpha]^{20}_D$ = -19 (c = 1.0 g/L, $CHCl_3$). The absolute configuration was assigned by analogy to that of **4b**. **HPLC condition**: Chiral column OD-H, n-hexane/i-PrOH = 95:5, flow rate = 1 mL/min, wavelength = 254 nm, tR = 35.98 min for major isomer, tR = 43.04 min for minor isomer.

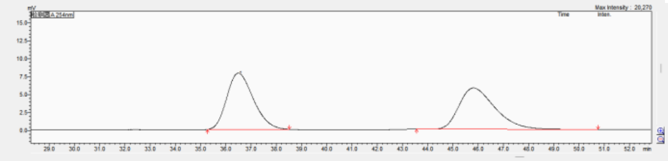

峰号	保留时间	面积	高度	标记	浓度	浓度单位	化合物ID号	化合物名	面积%
1	36.496	573245	7836	M	50.513				50.513
2	45.813	561596	5710	M	49.487				49.487
总计		1134840	13546		100.000				100.000

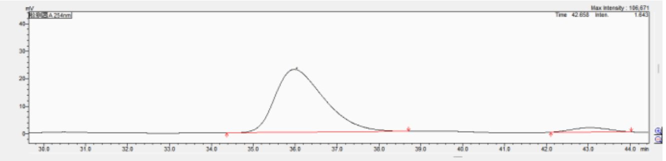

峰号	保留时间	面积	高度	标记	浓度	浓度单位	化合物ID号	化合物名	面积%
1	35.982	1865792	22920	M	95.021				95.021
2	43.036	97769	1606	M	4.979				4.979
总计		1963561	24526		100.000				100.000

N-((2R,3R)-4-(2-bromophenyl)-3-methyl-2-morpholinobutyl)benzamide (4l)

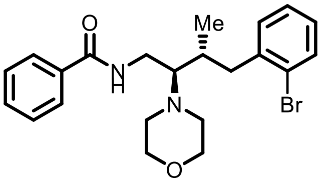

From (*E*)-*N*-(4-(2-bromophenyl)-3-methylbut-2-en-1-yl)benzamide (**1l**) (68.6 mg, 0.20 mmol, 1.0 equiv) and morpholino benzoate (**2a**) (62.1 mg, 0.30 mmol, 1.5 equiv), the title compound was prepared following the general procedure **A** using $NiBr_2$·DME (6.2 mg, 10 mol%), **(*S*,*S*)-L12** (15.4 mg, 15 mol%), LiOH (9.6 mg, 0.40 mmol, 2.0 equiv), KI (33.2 mg, 0.20 mmol, 1.0 equiv), $Me(EtO)_2SiH$ (80 μL, 0.50 mmol, 2.5 equiv), Anhydrous *tert*-butyl alcohol (1.0 mL, 0.20 M). The reaction mixture was stirred for 12 h at 30 °C. The crude material was purified by flash column chromatography (petroleum ether/EtOAc = 3:1) to provide the title compound as a yellow oil in 52% yield (44.7 mg).

^{1}H NMR (400 MHz, $CDCl_3$) δ 7.78 (d, *J* = 7.4 Hz, 2H), 7.56–7.49 (m, 2H), 7.47–7.41 (m, 2H), 7.25–7.19 (m, 1H), 7.18–7.14 (m, 1H), 7.11–7.02 (m, 1H), 6.94 (s, 1H), 3.83–3.73 (m, 1H), 3.72–3.60 (m, 4H), 3.43–3.32 (m, 1H), 2.97–2.87 (m, 1H), 2.79–2.66 (m, 2H), 2.63–2.52 (m, 2H), 2.50–2.42 (m, 2H), 2.41–2.31 (m, 1H), 0.95 (d, *J* = 6.8 Hz, 3H); **^{13}C NMR** (101 MHz, $CDCl_3$) δ 167.2, 139.9, 134.7, 133.0, 131.8, 131.5, 128.7, 128.0, 127.3, 126.8, 124.8, 67.8, 66.2, 49.4, 42.5, 36.4, 31.3, 16.4. **HRMS** (ESI) m/z calculated for $C_{22}H_{27}BrN_2O_2$ $[M+H]^+$ 431.1329 found: 371.1322. **Optical rotation**: $[\alpha]^{20}_D$ = -107 (c = 1.0 g/L, $CHCl_3$). The absolute configuration was assigned by analogy to that of **4b**. **HPLC condition**: Chiral column OD-H, n-hexane/i-PrOH = 95:5, flow rate = 1 mL/min, wavelength = 254 nm, tR = 48.54 min for major isomer, tR = 42.07 min for minor isomer.

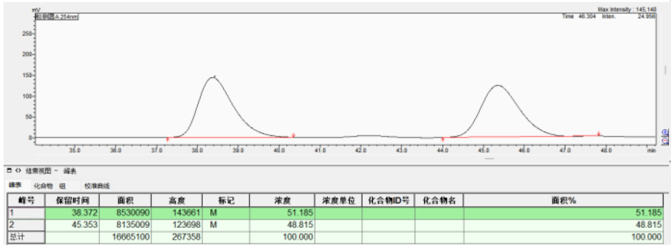

峰号	保留时间	面积	高度	标记	浓度	浓度单位	化合物ID号	化合物名	面积%
1	38.372	8530090	143661	M	51.185				51.185
2	45.353	8135009	123698	M	48.815				48.815
总计		16665100	267358		100.000				100.000

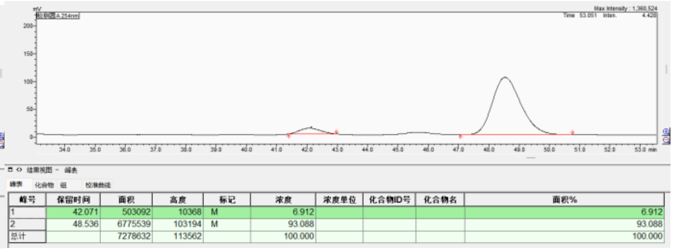

峰号	保留时间	面积	高度	标记	浓度	浓度单位	化合物ID号	化合物名	面积%
1	42.071	503092	10368	M	6.912				6.912
2	48.536	6775539	103194	M	93.088				93.088
总计		7278632	113562		100.000				100.000

N-((2R,3R)-4-(4-bromophenyl)-3-methyl-2-morpholinobutyl)benzamide (4m)

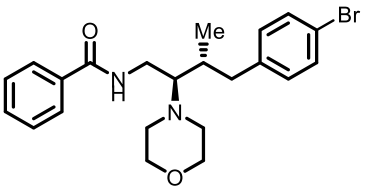

From (*E*)-*N*-(4-(4-bromophenyl)-3-methylbut-2-en-1-yl)benzamide (**1m**) (68.6 mg, 0.20 mmol, 1.0 equiv) and morpholino benzoate (**2a**) (62.1 mg, 0.30 mmol, 1.5 equiv), the title compound was prepared following the general procedure **A** using $NiBr_2$·DME (6.2 mg, 10 mol%), **(*S*,*S*)-L12** (15.4 mg, 15 mol%), LiOH (9.6 mg, 0.40 mmol, 2.0 equiv), KI (33.2 mg, 0.20 mmol, 1.0 equiv), $Me(EtO)_2SiH$

(80 μL, 0.50 mmol, 2.5 equiv), Anhydrous tert-butyl alcohol (1.0 mL, 0.20 M). The reaction mixture was stirred for 12 h at 30 °C. The crude material was purified by flash column chromatography (petroleum ether/EtOAc = 3:1) to provide the title compound as a yellow oil in 76% yield (65.4 mg).

^{1}H NMR (400 MHz, $CDCl_3$) δ 7.79 (d, J = 7.2 Hz, 2H), 7.57–7.49 (m, 2H), 7.48–7.42 (m, 2H), 7.24–7.15 (m, 2H), 7.11–7.02 (m, 1H), 6.91 (s, 1H), 3.84–3.75 (m, 1H), 3.74–3.60 (m, 4H), 3.43–3.33 (m, 1H), 2.98–2.86 (m, 1H), 2.81–2.67 (m, 2H), 2.65–2.54 (m, 1H), 2.51–2.43 (m, 2H), 2.41–2.33 (m, 1H), 1.83–1.71 (m, 1H), 0.96 (d, J = 6.8 Hz, 3H); **^{13}C NMR** (101 MHz, $CDCl_3$) δ 167.2, 133.0, 131.8, 131.5, 128.7, 128.0, 127.3, 126.8, 124.8, 67.8, 66.3, 49.4, 42.5, 36.3, 31.3, 16.4. **HRMS** (ESI) m/z calculated for $C_{22}H_{27}BrN_2O_2$ $[M+H]^+$ 431.1329 found: 371.1322. **Optical rotation**: $[\alpha]^{20}_D$ = -121 (c = 1.0 g/L, $CHCl_3$). The absolute configuration was assigned by analogy to that of **4b**. **HPLC condition**: Chiral column AD-H, n-hexane/i-PrOH = 95:5, flow rate = 1 mL/min, wavelength = 254 nm, tR = 45.36 min for major isomer, tR = 38.62 min for minor isomer.

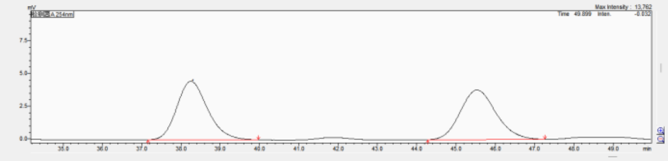

峰号	保留时间	面积	高度	标记	浓度	浓度单位	化合物ID号	化合物名	面积%
1	38.251	243853	4471		50.343				50.343
2	45.536	240526	3790		49.657				49.657
总计		484379	8260		100.000				100.000

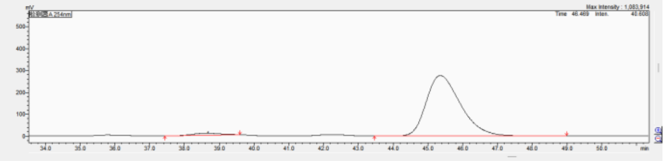

峰号	保留时间	面积	高度	标记	浓度	浓度单位	化合物ID号	化合物名	面积%
1	38.618	501594	9533	M	2.590				2.590
2	45.360	18863590	276557	M	97.410				97.410
总计		19365183	286090		100.000				100.000

***N*-((2*R*,3*R*)-5-(3-methoxy-4-((triisopropylsilyl)oxy)phenyl)-3-methyl-2-morpholinopentyl)benzamide (4n)**

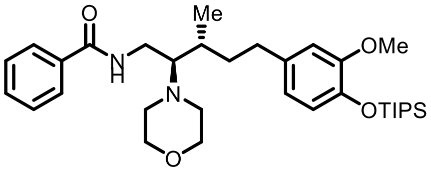

From (*E*)-*N*-(5-(3-methoxy-4-((triisopropylsilyl)oxy)phenyl)-3-methylpent-2-en-1-yl)benzamide (**1n**) (96.3 mg, 0.20 mmol, 1.0 equiv) and morpholino benzoate (**2a**) (62.1 mg, 0.30 mmol, 1.5 equiv), the title compound was prepared following the general procedure **A** using $NiBr_2 \cdot DME$ (6.2 mg, 10 mol%), **(*S*,*S*)-L12** (15.4 mg, 15 mol%), LiOH (9.6 mg, 0.40 mmol, 2.0 equiv), KI (33.2 mg, 0.20 mmol, 1.0 equiv), $Me(EtO)_2SiH$ (80 μL, 0.50 mmol, 2.5 equiv), Anhydrous *tert*-butyl alcohol (1.0 mL, 0.20 M). The reaction mixture was stirred for 12 h at 30 °C. The crude material was purified by flash column chromatography (petroleum ether/EtOAc = 5:1) to provide the title compound as a yellow oil in 86% yield (97.8 mg).

^{1}H NMR (400 MHz, $CDCl_3$) δ 7.76 (d, *J* = 7.5 Hz, 2H), 7.52–7.48 (m, 1H), 7.47–7.43 (m, 2H), 6.94 (s, 1H), 6.78 (d, *J* = 8.0 Hz, 1H), 6.65 (d, *J* = 2.0 Hz, 1H), 6.62–6.58 (m, 1H), 3.79 (s, 3H), 3.72–3.65 (m, 5H), 3.34–3.22 (m, 1H), 2.76–2.69 (m, 2H), 2.66–2.61 (m, 1H), 2.57–2.49 (m, 4H), 1.95–1.88 (m, 1H), 1.78–1.71 (m, 1H), 1.61–1.53 (m, 1H), 1.26–1.22 (m, 3H), 1.08 (d, *J* = 7.3 Hz, 18H), 1.01 (d, *J* = 6.8 Hz, 3H); **^{13}C NMR** (101 MHz, $CDCl_3$) δ 167.1, 150.7, 143.5, 135.1, 134.7, 131.4, 128.7, 126.8, 120.3, 120.2, 112.4, 67.9, 67.0, 55.6, 49.6, 37.5, 36.73, 33.0, 30.7, 18.0, 16.4, 12.9. **HRMS** (ESI) m/z calculated for $C_{33}H_{52}N_2O_4Si$ $[M+H]^+$ 569.3769 found: 569.3769. **Optical rotation**: $[\alpha]^{20}_D$ = -48.1 (c = 1.0 g/L, $CHCl_3$). The absolute configuration was assigned by analogy to that of **4b**. **HPLC condition**: Chiral column IF, n-hexane/i-PrOH = 95:5, flow rate = 1 mL/min, wavelength = 254 nm, tR = 31.50 min for major isomer, tR = 37.53 min for minor isomer.

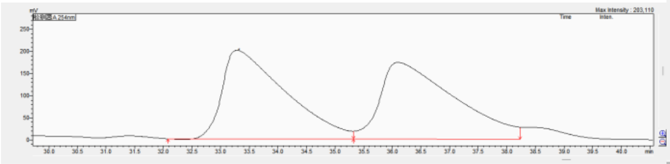

峰号	保留时间	面积	高度	标记	浓度	浓度单位	化合物ID号	化合物名	面积%
1	33.284	15396418	200898	M	49.294				49.294
2	36.099	15837331	173815	V M	50.706				50.706
总计		31233748	374712		100.000				100.000

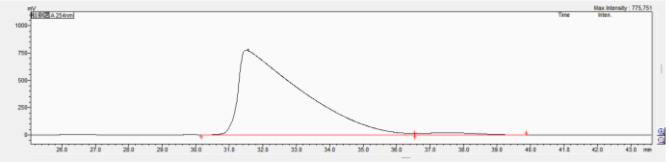

峰号	保留时间	面积	高度	标记	浓度	浓度单位	化合物ID号	化合物名	面积%
1	31.500	101468471	774596	M	98.034				98.034
2	37.531	2035252	18582	V M	1.966				1.966
总计		103503722	793179		100.000				100.000

***N*-((2*R*,3*R*)-3-methyl-2-morpholinohexyl)benzamide (4o)**

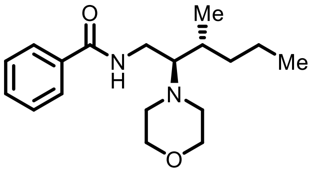

From (*E*)-*N*-(3-methylhex-2-en-1-yl)benzamide (**1o**) (43.4 mg, 0.20 mmol, 1.0 equiv) and morpholino benzoate (**2a**) (62.1 mg, 0.30 mmol, 1.5 equiv), the title compound was prepared following the general procedure **A** using $NiBr_2$·DME (6.2 mg, 10 mol%), **(*S*,*S*)-L12** (15.4 mg, 15 mol%), LiOH (9.6 mg, 0.40 mmol, 2.0 equiv), KI (33.2 mg, 0.20 mmol, 1.0 equiv), $Me(EtO)_2SiH$ (80 µL, 0.50 mmol, 2.5 equiv), Anhydrous *tert*-butyl alcohol (1.0 mL, 0.20 M). The reaction mixture was stirred for 12 h at 30 °C. The crude material was purified by flash column chromatography (petroleum ether/EtOAc = 5:1) to provide the title compound as a yellow oil in 62% yield (37.7 mg).

^{1}H NMR (400 MHz, $CDCl_3$) δ 7.77 (d, *J* = 7.4 Hz, 2H), 7.52–7.47 (m, 1H), 7.47–7.41 (m, 2H), 6.98 (s, 1H), 3.75–3.62 (m, 5H), 3.29–3.19 (m, 1H), 2.86–2.71 (m, 2H), 2.67–2.54 (m, 2H), 2.54–2.43 (m, 1H), 1.95–1.86 (m, 1H), 1.42–1.33 (m, 2H), 1.31–1.21 (m, 2H), 0.95–0.86 (m, 6H); **^{13}C NMR** (101 MHz, $CDCl_3$) δ 167.1, 134.8, 131.4, 128.7, 126.8, 67.9, 66.9, 49.5, 38.2, 36.6, 31.0, 20.3, 16.5, 14.3. **HRMS** (ESI) m/z calculated for $C_{18}H_{28}N_2O_2$ $[M+H]^+$ 305.2224 found: 305.2224. **Optical rotation**: $[\alpha]^{20}_D$ = -104

(c = 1.0 g/L, $CHCl_3$). The absolute configuration was assigned by analogy to that of **4b**. **HPLC condition**: Chiral column AD-H, n-hexane/i-PrOH = 95:5, flow rate = 1 mL/min, wavelength = 254 nm, tR = 21.57 min for major isomer, tR = 19.80 min for minor isomer.

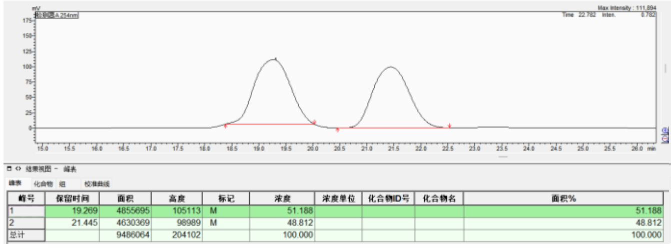

峰号	保留时间	面积	高度	标记	浓度	浓度单位	化合物ID号	化合物名	面积%
1	19.269	4855695	105113	M	51.188				51.188
2	21.445	4630369	98989	M	48.812				48.812
总计		9486064	204102		100.000				100.000

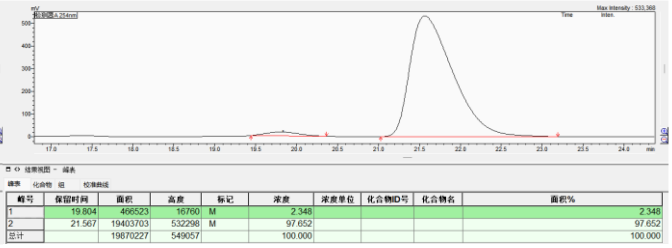

峰号	保留时间	面积	高度	标记	浓度	浓度单位	化合物ID号	化合物名	面积%
1	19.804	466523	16760	M	2.348				2.348
2	21.567	19403703	532298	M	97.652				97.652
总计		19870227	549057		100.000				100.000

***N*-((2*R*,3*R*)-3-methyl-2-morpholinodecyl)benzamide (4p)**

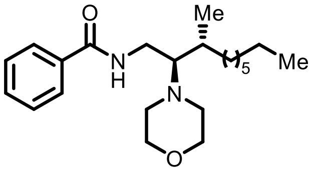

From (*E*)-*N*-(3-methyldec-2-en-1-yl)benzamide (**1p**) (54.6 mg, 0.20 mmol, 1.0 equiv) and morpholino benzoate (**2a**) (62.1 mg, 0.30 mmol, 1.5 equiv), the title compound was prepared following the general procedure **A** using $NiBr_2$·DME (6.2 mg, 10 mol%), **(*S*,*S*)-L12** (15.4 mg, 15 mol%), LiOH (9.6 mg, 0.40 mmol, 2.0 equiv), KI (33.2 mg, 0.20 mmol, 1.0 equiv), $Me(EtO)_2SiH$ (80 μL, 0.50 mmol, 2.5 equiv), Anhydrous *tert*-butyl alcohol (1.0 mL, 0.20 M). The reaction mixture was stirred for 12 h at 30 °C. The crude material was purified by flash column chromatography (petroleum ether/EtOAc = 5:1) to provide the title compound as a yellow oil in 79% yield (56.9 mg).

^{1}H NMR (400 MHz, $CDCl_3$) δ 7.77 (d, *J* = 7.2 Hz, 2H), 7.51–7.47 (m, 1H), 7.46–7.42 (m, 2H), 7.00 (s, 1H), 3.74–3.65 (m, 5H), 3.29–3.19 (m, 1H), 2.82–2.75 (m, 2H), 2.61–2.54 (m, 2H), 2.54–2.47 (m, 1H), 1.93–1.84 (m, 1H), 1.40–1.35 (m, 1H), 1.29–1.23 (m, 11H), 0.93 (d, *J* = 6.9 Hz, 3H), 0.88 (t, *J* = 6.4 Hz, 3H); **^{13}C NMR** (101 MHz, $CDCl_3$) δ 167.1, 134.8, 131.4, 128.7, 126.8, 67.9, 66.9, 49.5, 36.6, 35.9, 31.9, 31.3, 29.8, 29.4, 27.2, 22.7, 16.6, 14.2. **HRMS** (ESI) m/z calculated for $C_{22}H_{36}N_2O_2$ $[M+H]^+$ 361.2850 found: 361.2847. **Optical rotation**: $[\alpha]^{20}_D$ = -29.9 (c = 1.0 g/L, $CHCl_3$). The absolute configuration was assigned by analogy to that of **4b**. **HPLC condition**: Chiral column AD-H, n-hexane/i-PrOH = 95:5, flow rate = 1 mL/min, wavelength = 254 nm, tR = 15.24 min for major isomer, tR = 13.83 min for minor isomer.

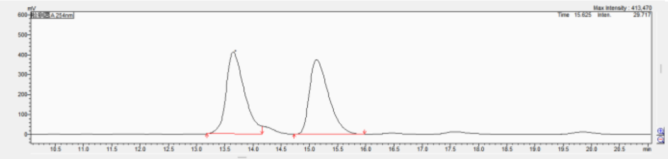

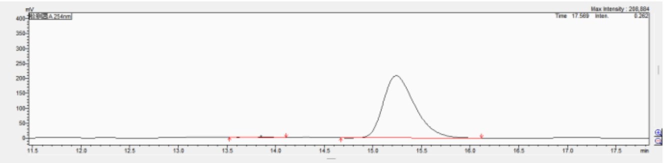

峰号	保留时间	面积	高度	标记	浓度	浓度单位	化合物ID号	化合物名	面积%
1	13.651	9231789	409123	M	51.228				51.228
2	15.129	8789240	373257	M	48.772				48.772
总计		18021028	782380		100.000				100.000

峰号	保留时间	面积	高度	标记	浓度	浓度单位	化合物ID号	化合物名	面积%
1	13.827	22292	1766	M	0.475				0.475
2	15.239	4669381	208028	M	99.525				99.525
总计		4691672	209794		100.000				100.000

N-((2*R*,3*R*)-3-methyl-2-morpholinotetradecyl)benzamide (4q)

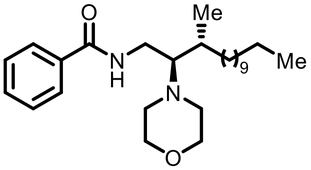

From (*E*)-*N*-(3-methyltetradec-2-en-1-yl)benzamide (**1q**) (65.9 mg, 0.20 mmol, 1.0 equiv) and morpholino benzoate (**2a**) (62.1 mg, 0.30 mmol, 1.5 equiv), the title compound was prepared following the general procedure **A** using $NiBr_2$·DME (6.2 mg, 10 mol%), **(*S*,*S*)-L12** (15.4 mg, 15 mol%), LiOH (9.6 mg, 0.40 mmol, 2.0 equiv), KI (33.2 mg, 0.20 mmol, 1.0 equiv), $Me(EtO)_2SiH$ (80 μL, 0.50 mmol, 2.5 equiv), Anhydrous *tert*-butyl alcohol (1.0 mL, 0.20 M). The reaction mixture was stirred for 12 h at 30 °C. The crude material was purified by flash column chromatography (petroleum ether/EtOAc = 5:1) to provide the title compound as a yellow oil in 58% yield (48.3 mg).

^{1}H NMR (400 MHz, $CDCl_3$) δ 7.79–7.74 (m, 2H), 7.51–7.47 (m, 1H), 7.46–7.42 (m, 2H), 7.04–6.96 (m, 1H), 3.74–3.64 (m, 5H), 3.27–3.19 (m, 1H), 2.81–2.73 (m, 2H), 2.61–2.54 (m, 2H), 2.53–2.47 (m, 1H), 1.93–1.83 (m, 1H), 1.32–1.30 (m, 1H), 1.27–1.23 (m, 19H), 0.93 (d, *J* = 6.9 Hz, 3H), 0.87 (t, *J* = 6.7 Hz, 3H); **^{13}C NMR** (101 MHz, $CDCl_3$) δ 167.0, 134.8, 131.4, 128.7, 126.8, 67.9, 66.8, 49.5, 36.6, 35.9, 32.0, 31.3, 29.9, 29.7, 29.4, 27.2, 22.7, 16.6, 14.2. **HRMS** (ESI) m/z calculated for $C_{26}H_{44}N_2O_2$ $[M+H]^+$ 417.3476 found: 417.3475. **Optical rotation**: $[\alpha]^{20}_D$ = -14.8 (c = 1.0 g/L, $CHCl_3$). The absolute configuration was assigned by analogy to that of **4b**. **HPLC condition**: Chiral column AD-H, n-hexane/i-PrOH = 95:5, flow rate = 1 mL/min, wavelength = 254 nm, tR = 12.18 min for major isomer, tR = 11.36 min for minor isomer.

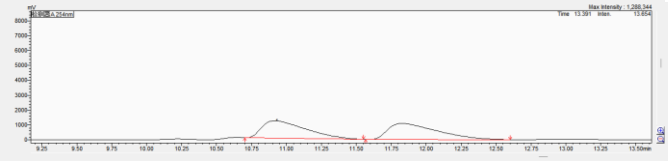

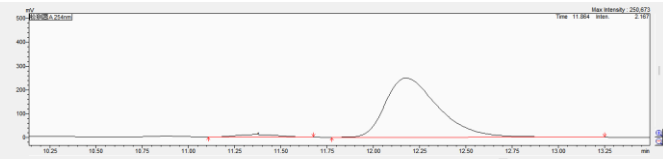

峰号	保留时间	面积	高度	标记	浓度	浓度单位	化合物ID号	化合物名	面积%
1	10.916	25166341	1164954	M	49.134				49.134
2	11.827	26053617	1072146	M	50.866				50.866
总计		51219957	2237099		100.000				100.000

峰号	保留时间	面积	高度	标记	浓度	浓度单位	化合物ID号	化合物名	面积%
1	11.365	165986	10609	M	3.250				3.250
2	12.177	4941543	249044	M	96.750				96.750
总计		5107529	259653		100.000				100.000

N-((2R,3R,7R,11R)-3,7,11,15-tetramethyl-2-morpholinohexadecyl)benzamide (4r)

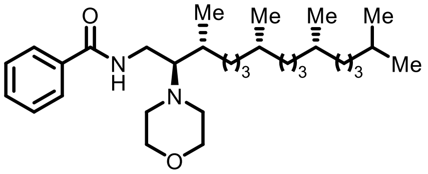

From *N*-((7*R*,11*R*,*E*)-3,7,11,15-tetramethylhexadec-2-en-1-yl)benzamide (**1r**) (79.9 mg, 0.20 mmol, 1.0 equiv) and morpholino benzoate (**2a**) (62.1 mg, 0.30 mmol, 1.5 equiv), the title compound was prepared following the general procedure **A** using $NiBr_2$·DME (6.2 mg, 10 mol%), **(*S*,*S*)-L12** (15.4 mg, 15 mol%), LiOH (9.6 mg, 0.40 mmol, 2.0 equiv), KI (33.2 mg, 0.20 mmol, 1.0 equiv), $Me(EtO)_2SiH$ (80 μL, 0.50 mmol, 2.5 equiv), Anhydrous *tert*-butyl alcohol (1.0 mL, 0.20 M). The reaction mixture was stirred for 12 h at 30 °C. The crude material was purified by flash column chromatography (petroleum ether/EtOAc = 5:1) to provide the title compound as a yellow oil in 67% yield (65.2 mg).

^{1}H NMR (400 MHz, $CDCl_3$) δ 7.80–7.75 (m, 2H), 7.52–7.48 (m, 1H), 7.47–7.43 (m, 2H), 6.99 (s, 1H), 3.77–3.65 (m, 5H), 3.30–3.19 (m, 1H), 2.79 (s, 2H), 2.59 (s, 2H), 2.52 (s, 1H), 1.95–1.86 (m, 1H), 1.77–1.71 (m, 1H), 1.68–1.45 (m, 2H), 1.39–1.32 (m, 5H), 1.30–1.27 (m, 4H), 1.22–1.19 (m, 2H), 1.16–1.11 (m, 3H), 1.08–1.01 (m, 4H), 0.95 (d, *J* = 6.8 Hz, 3H), 0.87–0.85 (m, 9H), 0.84 (d, *J* = 3.1 Hz, 3H). **^{13}C NMR** (101 MHz, $CDCl_3$) δ 167.1, 134.8, 131.4, 128.6, 126.8, 67.9, 66.8, 49.5, 39.4, 37.5, 37.5, 37.4, 37.3, 37.2, 37.1, 36.2, 32.8, 31.3, 28.0, 24.8, 24.6, 24.5, 22.7, 22.6, 19.8, 19.7. **HRMS** (ESI) m/z calculated for $C_{31}H_{54}N_2O_2$ $[M+H]^+$ 487.4258 found: 487.4249. **Optical rotation**: $[\alpha]^{20}_D$ = -23.8 (c = 1.0 g/L, $CHCl_3$). The absolute configuration was assigned by analogy to that of **4b**. **HPLC condition**: Chiral column IF, n-hexane/i-PrOH = 95:5, flow rate = 1 mL/min, wavelength = 254 nm, tR = 18.3 min for major isomer, tR = 20.1 min for minor isomer.

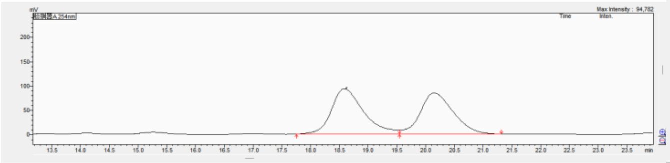

峰号	保留时间	面积	高度	标记	浓度	浓度单位	化合物ID号	化合物名	面积%
1	18.586	3628444	93268	M	51.920				51.920
2	20.147	3360135	84528	V M	48.080				48.080
总计		6988579	177797		100.000				100.000

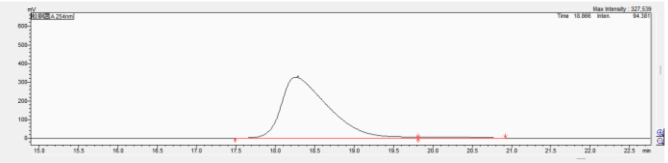

峰号	保留时间	面积	高度	标记	浓度	浓度单位	化合物ID号	化合物名	面积%
1	18.260	13359096	326278	M	98.193				98.193
2	20.125	245909	6118	V M	1.807				1.807
总计		13605005	332397		100.000				100.000

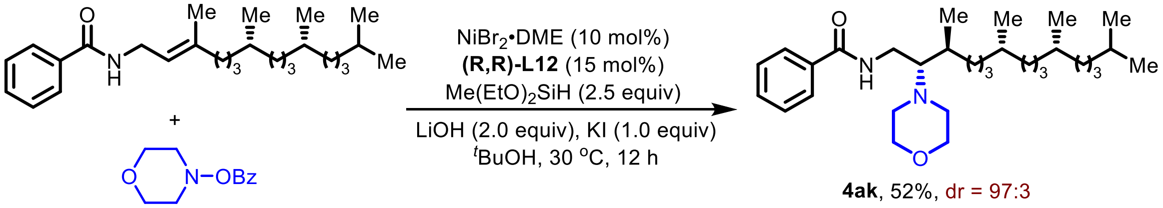

4ak, 52%, dr = 97:3

N-((2*S*,3*S*,7*R*,11*R*)-3,7,11,15-tetramethyl-2-morpholinohexadecyl)benzamide (4ak)

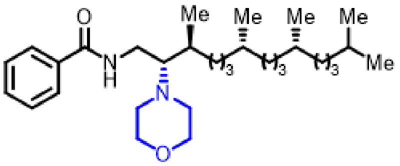

From *N*-((7*R*,11*R*,*E*)-3,7,11,15-tetramethylhexadec-2-en-1-yl)benzamide (**1r**) (79.9 mg, 0.20 mmol, 1.0 equiv) and morpholino benzoate (**2a**) (62.1 mg, 0.30 mmol, 1.5 equiv), the title compound was prepared following the general procedure **A** using $NiBr_2$·DME (6.2 mg, 10 mol%), **(*R*,*R*)-L12** (15.4 mg, 15 mol%), LiOH (9.6 mg, 0.40 mmol, 2.0 equiv), KI (33.2 mg, 0.20 mmol, 1.0 equiv), $Me(EtO)_2SiH$ (80 µL, 0.50 mmol, 2.5 equiv), Anhydrous *tert*-butyl alcohol (1.0 mL, 0.20 M). The reaction mixture was stirred for 12 h at 30 °C. The crude material was purified by flash column chromatography (petroleum ether/EtOAc = 5:1) to provide the title compound as a yellow oil in 52% yield (65.2 mg).

^{1}H NMR (400 MHz, $CDCl_3$) δ 7.77 (d, *J* = 7.4 Hz, 2H), 7.51–7.48 (m, 1H), 7.46–7.42 (m, 2H), 7.01 (s, 1H), 3.75–3.65 (m, 5H), 3.34–3.17 (m, 1H), 2.82–2.74 (m, 2H), 2.65–2.56 (m, 2H), 2.55–2.47 (m, 1H), 1.94–1.83 (m, 1H), 1.54–1.47 (m, 1H), 1.31–1.21 (m, 14H), 1.15–1.11 (m, 3H), 1.08–1.04 (m, 3H), 0.97–0.93 (m, 3H), 0.86–0.83 (m, 12H). **^{13}C NMR** (101 MHz, $CDCl_3$) δ 167.1, 134.8, 131.4, 128.7, 126.8, 67.9, 67.0, 49.5, 39.4, 37.5, 37.5, 37.3, 37.1, 36.2, 32.8, 32.8, 31.2, 28.0, 24.8, 24.6, 24.5, 22.8, 22.7, 19.8, 19.7, 16.5. **HRMS** (ESI) m/z calculated for $C_{31}H_{54}N_2O_2$ $[M+H]^+$ 487.4258 found: 487.4249. **Optical rotation**: $[\alpha]^{20}_D$ = 18.5 (c = 1.0 g/L, $CHCl_3$). The absolute configuration was assigned by analogy to that of **4b**. **HPLC condition**: Chiral column IF, n-hexane/i-PrOH = 95:5, flow rate = 1 mL/min, wavelength = 254 nm, tR = 21.1 min for major isomer, tR = 19.6 min for minor isomer.

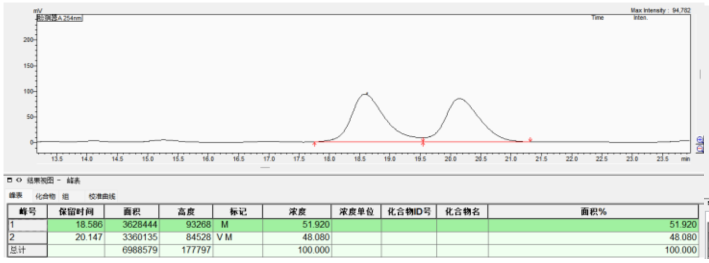

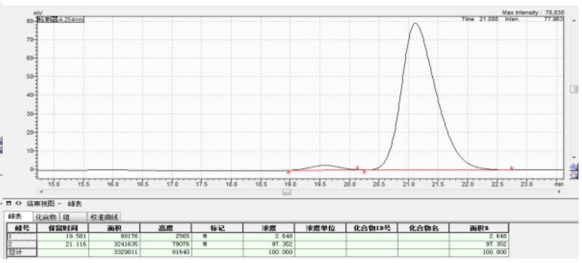

N-((2*R*,3*R*)-3,7-dimethyl-2-morpholinooct-6-en-1-yl)benzamide (4s)

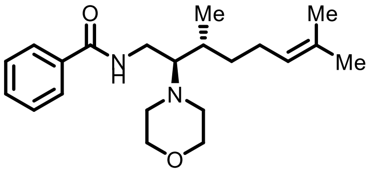

From (*E*)-*N*-(3,7-dimethylocta-2,6-dien-1-yl)benzamide (**1s**) (51.4 mg, 0.20 mmol, 1.0 equiv) and morpholino benzoate (**2a**) (62.1 mg, 0.30 mmol, 1.5 equiv), the title compound was prepared following the general

procedure **A** using NiBr$_2$·DME (6.2 mg, 10 mol%), ***(S,S)*-L12** (15.4 mg, 15 mol%), LiOH (9.6 mg, 0.40 mmol, 2.0 equiv), KI (33.2 mg, 0.20 mmol, 1.0 equiv), Me(EtO)$_2$SiH (80 μL, 0.50 mmol, 2.5 equiv), Anhydrous *tert*-butyl alcohol (1.0 mL, 0.20 M). The reaction mixture was stirred for 12 h at 30 °C. The crude material was purified by flash column chromatography (petroleum ether/EtOAc = 5:1) to provide the title compound as a yellow oil in 63% yield (43.4 mg).

^{1}H NMR (400 MHz, CDCl$_3$) δ 7.73–7.67 (m, 2H), 7.46–7.41 (m, 1H), 7.40–7.36 (m, 2H), 6.90 (s, 1H), 5.05–4.98 (m, 1H), 3.69–3.58 (m, 5H), 3.23–3.14 (m, 1H), 2.76–2.66 (m, 2H), 2.55–2.48 (m, 2H), 2.48–2.41 (m, 1H), 2.03–1.88 (m, 2H), 1.87–1.82 (m, 1H), 1.62 (s, 3H), 1.54 (s, 3H), 1.43–1.34 (m, 1H), 1.28–1.22 (m, 1H), 0.89 (d, J = 6.9 Hz, 3H); **^{13}C NMR** (101 MHz, CDCl$_3$) δ 167.1, 134.8, 131.9, 131.4, 128.7, 126.8, 124.1, 67.9, 66.9, 49.6, 36.7, 35.9, 30.9, 25.8, 25.6, 17.8, 16.5. **HRMS** (ESI) m/z calculated for $C_{21}H_{32}N_2O_2$ $[M+H]^+$ 345.2537 found: 345.2539. **Optical rotation**: $[\alpha]^{20}_D$ = -52.4 (c = 1.0 g/L, CHCl$_3$). The absolute configuration was assigned by analogy to that of **4b**. **HPLC condition**: Chiral column AD-H, n-hexane/i-PrOH = 95:5, flow rate = 1 mL/min, wavelength = 254 nm, tR = 19.83 min for major isomer, tR = 22.29 min for minor isomer.

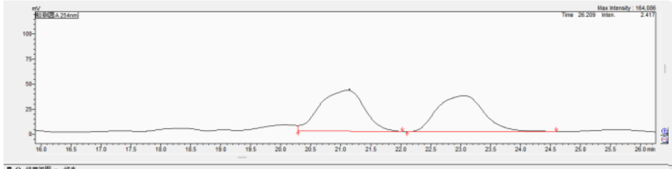

峰号	保留时间	面积	高度	标记	浓度	浓度单位	化合物ID号	化合物名	面积%
1	21.113	2096377	40942	M	51.940				51.940
2	23.058	1939760	36034	M	48.060				48.060
总计		4036137	76976		100.000				100.000

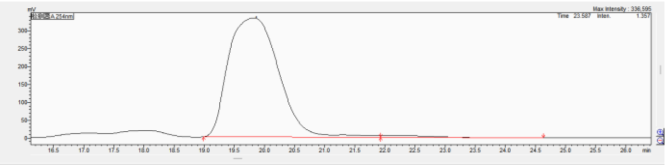

峰号	保留时间	面积	高度	标记	浓度	浓度单位	化合物ID号	化合物名	面积%
1	19.825	19249026	333299	M	98.815				98.815
2	22.290	230779	4571	V M	1.185				1.185
总计		19479805	337871		100.000				100.000

N-((2*R*,3*R*)-3-methyl-2-morpholino-5-((triisopropylsilyl)oxy)pentyl)benzamide (4t)

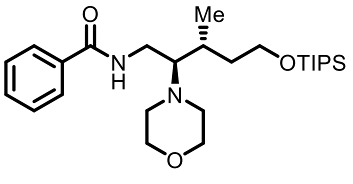

From (*E*)-*N*-(3-methyl-5-((triisopropylsilyl)oxy)pent-2-en-1-yl)benzamide (**1t**) (75.1 mg, 0.20 mmol, 1.0 equiv) and morpholino benzoate (**2a**) (62.1 mg, 0.30 mmol, 1.5 equiv), the title compound was prepared following the general procedure **A** using NiBr$_2$·DME (6.2 mg, 10 mol%), ***(S,S)*-L12** (15.4 mg, 15 mol%), LiOH (9.6 mg, 0.40 mmol, 2.0 equiv), KI (33.2 mg, 0.20 mmol, 1.0 equiv), Me(EtO)$_2$SiH (80 μL, 0.50 mmol, 2.5 equiv), Anhydrous *tert*-butyl alcohol (1.0 mL, 0.20 M). The reaction mixture was stirred for 12 h at 30 °C. The crude material was purified by flash column chromatography (petroleum ether/EtOAc = 5:1) to provide the title compound as a yellow oil in 58% yield (53.6 mg).

^{1}H NMR (400 MHz, $CDCl_3$) δ 7.77 (d, *J* = 7.3 Hz, 2H), 7.52–7.48 (m, 1H), 7.47–7.43 (m, 2H), 6.99 (s, 1H), 3.77–3.67 (m, 7H), 3.38–3.27 (m, 1H), 2.83–2.77 (m, 2H), 2.66–2.56 (m, 3H), 1.78–1.72 (m, 1H), 1.52–1.36 (m, 2H), 1.07–1.04 (m, 21H), 0.97 (d, *J* = 6.9 Hz, 3H); **^{13}C NMR** (101 MHz, $CDCl_3$) δ 167.2, 131.4, 128.7, 126.8, 67.8, 61.0, 49.6, 38.8, 36.7, 29.7, 27.7, 18.1, 16.3, 12.0. **HRMS** (ESI) m/z calculated for $C_{26}H_{46}N_2O_3Si$ $[M+H]^+$ 463.3351 found: 463.3349. **Optical rotation**: $[\alpha]^{20}_D$ = -3.77 (c = 1.0 g/L, $CHCl_3$). The absolute configuration was assigned by analogy to that of **4b**. **HPLC condition**: Chiral column IC, n-hexane/i-PrOH = 95:5, flow rate = 1 mL/min, wavelength = 254 nm, tR = 43.8 min for major isomer, tR = 39.1 min for minor isomer.

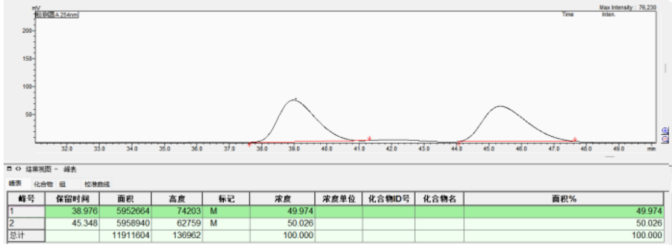

峰号	保留时间	面积	高度	标记	浓度	浓度单位	化合物ID号	化合物名	面积%
1	38.976	5952664	74203	M	49.974				49.974
2	45.348	5958940	62759	M	50.026				50.026
总计		11911604	136962		100.000				100.000

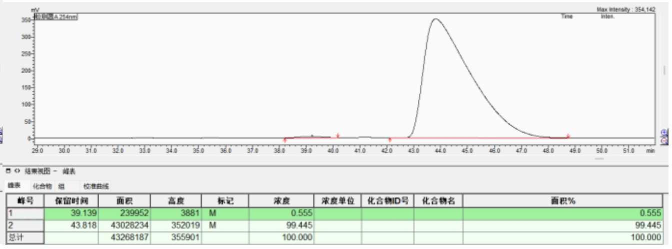

峰号	保留时间	面积	高度	标记	浓度	浓度单位	化合物ID号	化合物名	面积%
1	39.139	239952	3881	M	0.555				0.555
2	43.818	43028234	352019	M	99.445				99.445
总计		43268187	355901		100.000				100.000

N-((2R,3R)-3-methyl-2-morpholino-6-((triisopropylsilyl)oxy)hexyl)benzamide (4u)

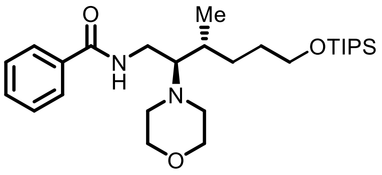

From (*E*)-*N*-(3-methyl-6-((triisopropylsilyl)oxy)hex-2-en-1-yl)benzamide (**1u**) (77.9 mg, 0.20 mmol, 1.0 equiv) and morpholino benzoate (**2a**) (62.1 mg, 0.30 mmol, 1.5 equiv), the title compound was prepared following the general procedure **A** using $NiBr_2$·DME (6.2 mg, 10 mol%), **(*S*,*S*)-L12** (15.4 mg, 15 mol%), LiOH (9.6 mg, 0.40 mmol, 2.0 equiv), KI (33.2 mg, 0.20 mmol, 1.0 equiv), $Me(EtO)_2SiH$ (80 μL, 0.50 mmol, 2.5 equiv), Anhydrous *tert*-butyl alcohol (1.0 mL, 0.20 M). The reaction mixture was stirred for 12 h at 30 °C. The crude material was purified by flash column chromatography (petroleum ether/EtOAc = 5:1) to provide the title compound as a yellow oil in 76% yield (72.4 mg).

^{1}H NMR (400 MHz, $CDCl_3$) δ 9.11 (s, 1H), 8.10 (d, *J* = 7.3 Hz, 2H), 7.47–7.40 (m, 3H), 4.57–4.40 (m, 2H), 4.08–3.90 (m, 3H), 3.74–3.64 (m, 3H), 3.55–3.46 (m, 1H), 3.37–3.27 (m, 2H), 3.26–3.16 (m, 1H), 3.05 (d, *J* = 11.1 Hz, 1H), 2.19–2.07 (m, 1H), 1.70–1.58 (m, 4H), 1.16 (d, *J* = 6.4 Hz, 3H), 1.05–1.02 (m, 21H); **^{13}C NMR** (101 MHz, $CDCl_3$) δ 167.7, 133.0, 131.9, 128.5, 127.8, 72.7, 63.6, 63.5, 63.0, 52.3, 47.8, 35.4, 32.3, 30.5, 30.5, 18.1, 15.8, 11.9. **HRMS** (ESI) m/z calculated for $C_{27}H_{48}N_2O_3Si$ $[M+Na]^+$ 499.3326 found: 499.3326. **Optical rotation**: $[\alpha]^{20}_D$ = -5.10 (c = 1.0 g/L, $CHCl_3$). The absolute configuration was

assigned by analogy to that of **4b**. **HPLC condition**: Chiral column OD-H, n-hexane/i-PrOH = 99:1, flow rate = 0.8 mL/min, wavelength = 254 nm, tR = 80.1 min for major isomer, tR = 92.9 min for minor isomer.

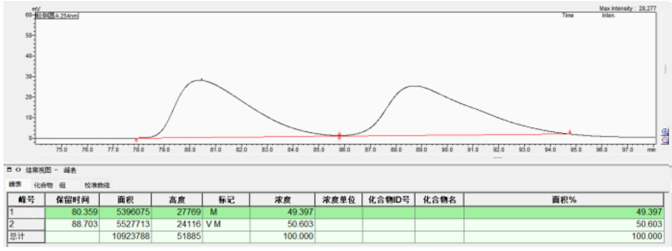

峰号	保留时间	面积	高度	标记	浓度	浓度单位	化合物ID号	化合物名	面积%
1	80.359	5396075	27769	M	49.397				49.397
2	88.703	5527713	24116	V M	50.603				50.603
总计		10923788	51885		100.000				100.000

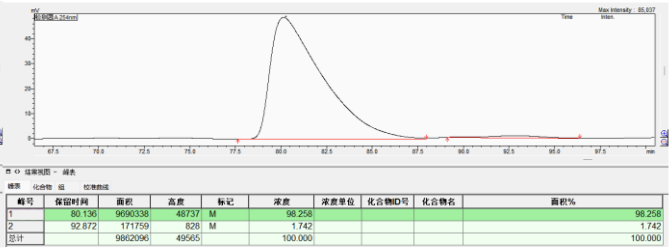

峰号	保留时间	面积	高度	标记	浓度	浓度单位	化合物ID号	化合物名	面积%
1	80.136	9690338	48737	M	98.258				98.258
2	92.872	171759	828	M	1.742				1.742
总计		9862096	49565		100.000				100.000

(4*R*,5*R*)-6-benzamido-4-methyl-5-morpholinohexyl acetate (4v)

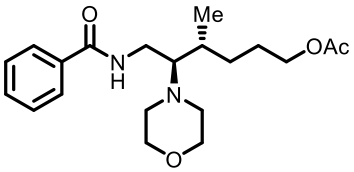

From (*E*)-6-benzamido-4-methylhex-4-en-1-yl acetate (**1v**) (55.0 mg, 0.20 mmol, 1.0 equiv) and morpholino benzoate (**2a**) (62.1 mg, 0.30 mmol, 1.5 equiv), the title compound was prepared following the general procedure **A** using $NiBr_2$·DME (6.2 mg, 10 mol%), **(*S*,*S*)-L12** (15.4 mg, 15 mol%), LiOH (9.6 mg, 0.40 mmol, 2.0 equiv), KI (33.2 mg, 0.20 mmol, 1.0 equiv), $Me(EtO)_2SiH$ (80 µL, 0.50 mmol, 2.5 equiv), Anhydrous *tert*-butyl alcohol (1.0 mL, 0.20 M). The reaction mixture was stirred for 12 h at 30 °C. The crude material was purified by flash column chromatography (petroleum ether/EtOAc = 5:1) to provide the title compound as a yellow oil in 62% yield (44.9 mg).

^{1}H NMR (400 MHz, $CDCl_3$) δ 7.77 (d, *J* = 7.5 Hz, 2H), 7.54–7.49 (m, 1H), 7.48–7.43 (m, 2H), 6.93 (s, 1H), 4.08–4.04 (m, 2H), 3.76–3.66 (m, 5H), 3.32–3.22 (m, 1H), 2.81–2.72 (m, 2H), 2.63–2.55 (m, 2H), 2.55–2.46 (m, 1H), 2.05 (s, 3H), 1.95–1.86 (m, 1H), 1.73–1.67 (m, 1H), 1.64–1.57 (m, 1H), 1.52–1.44 (m, 1H), 1.35–1.30 (m, 1H), 0.97 (d, *J* = 6.8 Hz, 3H); **^{13}C NMR** (101 MHz, $CDCl_3$) δ 171.2, 167.1, 131.5, 128.7, 126.8, 67.9, 66.9, 64.5, 49.6, 36.7, 31.9, 31.2, 26.3, 21.1, 16.5. **HRMS** (ESI) m/z calculated for $C_{20}H_{30}N_2O_4$ $[M+H]^+$ 363.2279 found: 363.2275. **Optical rotation**: $[\alpha]^{20}_D$ = 6.43 (c = 1.0 g/L, $CHCl_3$). The absolute configuration was assigned by analogy to that of **4b**. **HPLC condition**: Chiral column AD-H, n-hexane/i-PrOH = 95:5, flow rate = 1 mL/min, wavelength = 254 nm, tR = 74.8 min for major isomer, tR = 54.6 min for minor isomer.

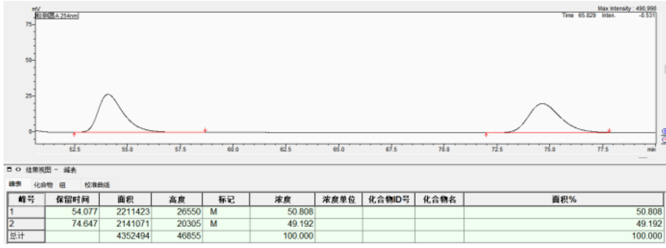

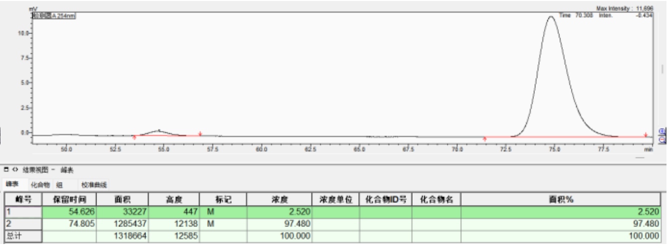

_N_-((2_R_,3_R_)-3-ethyl-2-morpholinoheptyl)benzamide (4w)

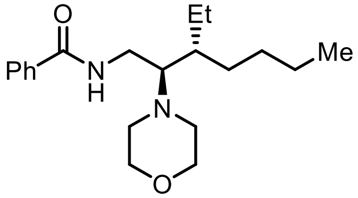

From (*E*)-*N*-(3-ethylhept-2-en-1-yl)benzamide (**1ac**) (49.0 mg, 0.20 mmol, 1.0 equiv) and morpholino benzoate (**2a**) (62.1 mg, 0.30 mmol, 1.5 equiv), the title compound was prepared following the general procedure **A** using $NiBr_2$·DME (6.2 mg, 10 mol%), **(*S*,*S*)-L2** (7.56 mg, 15 mol%), LiOH (9.6 mg, 0.40 mmol, 2.0 equiv), KI (33.2 mg, 0.20 mmol, 1.0 equiv), $Me(EtO)_2SiH$ (80 μL, 0.50 mmol, 2.5 equiv), Anhydrous *tert*-butyl alcohol (1.0 mL, 0.20 M). The reaction mixture was stirred for 12 h at 30 °C. The crude material was purified by flash column chromatography (petroleum ether/EtOAc = 3:1) to provide the title compound as a yellow oil in 42% yield (27.9 mg).

^{1}H NMR (400 MHz, $CDCl_3$) δ 7.71 (d, *J* = 7.1 Hz, 2H), 7.48–7.42 (m, 1H), 7.41–7.36 (m, 2H), 6.93 (s, 1H), 3.72–3.56 (m, 5H), 3.23–3.12 (m, 1H), 2.74–2.64 (m, 2H), 2.58–2.51 (m, 1H), 2.48 (d, *J* = 5.6 Hz, 2H), 1.53–1.43 (m, 2H), 1.39–1.33 (m, 1H), 1.29–1.21 (m, 4H), 1.13–1.01 (m, 2H), 0.86–0.81 (m, 6H). **^{13}C NMR** (101 MHz, $CDCl_3$) δ 166.0, 133.8, 130.3, 127.6, 125.8, 66.8, 64.2, 48.5, 36.9, 35.5, 30.0, 28.5, 22.0, 22.0, 13.1, 10.7. **HRMS** (ESI) m/z calculated for $C_{20}H_{32}N_2O_2$ $[M+H]^+$ 332.2537 found: 332.2537. **Optical rotation**: $[\alpha]^{20}_D$ = -115 (c = 1.0 g/L, $CHCl_3$). The absolute configuration was assigned by analogy to that of **4b**. **HPLC condition**: Chiral column AD-H, n-hexane/i-PrOH = 95:5, flow rate = 1 mL/min, wavelength = 254 nm, tR = 19.66 min for major isomer, tR = 16.06 min for minor isomer.

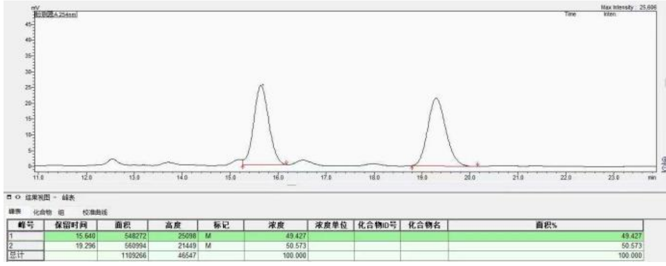

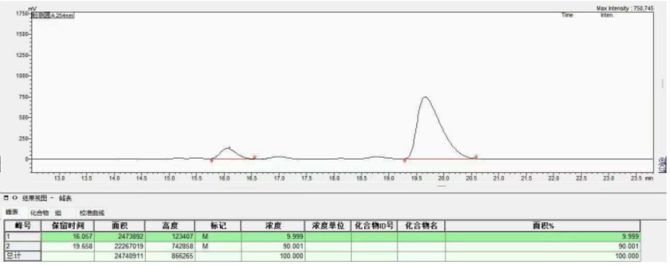

N-((2*R*,3*R*)-2-morpholino-3-phenethylhexyl)benzamide (4x)

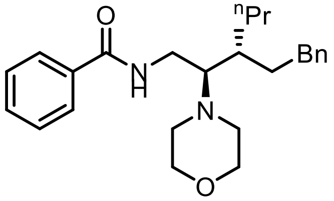

From (*E*)-*N*-(3-phenethylhex-2-en-1-yl)benzamide (**1aj**) (61.4 mg, 0.20 mmol, 1.0 equiv) and morpholino benzoate (**2a**) (62.1 mg, 0.30 mmol, 1.5 equiv), the title compound was prepared following the general procedure **A** using $NiBr_2$·DME (6.2 mg, 10 mol%), **(*S*,*S*)-L2** (7.56 mg, 15 mol%), LiOH (9.6 mg, 0.40 mmol, 2.0 equiv), KI (33.2 mg, 0.20 mmol, 1.0 equiv), $Me(EtO)_2SiH$ (80 µL, 0.50 mmol, 2.5 equiv), Anhydrous *tert*-butyl alcohol (1.0 mL, 0.20 M). The reaction mixture was stirred for 12 h at 30 °C. The crude material was purified by flash column chromatography (petroleum ether/EtOAc = 1:1) to provide the title compound as a yellow oil in 55% yield (43.4 mg).

^{1}H NMR (400 MHz, $CDCl_3$) δ 7.70 (s, 2H), 7.49–7.32 (m, 3H), 7.26–7.17 (m, 2H), 7.16–7.07 (m, 3H), 6.82 (s, 1H), 3.77–3.47 (m, 5H), 3.30–3.20 (m, 1H), 2.76–2.35 (m, 7H), 1.75–1.56 (m, 3H), 1.46–1.33 (m, 2H), 1.28–1.12 (m, 2H), 0.83 (t, *J* = 6.8 Hz, 3H). **^{13}C NMR** (101 MHz, $CDCl_3$) δ 167.2, 142.1, 131.5, 128.7, 128.5, 128.4, 126.9, 126.0, 67.8, 49.6, 36.8, 35.8, 34.1, 33.5, 33.5, 32.8, 20.5, 14.5. **HRMS** (ESI) m/z calculated for $C_{25}H_{34}N_2O_2$ $[M+H]^+$ 395.2693 found: 395.2695. **Optical rotation**: $[\alpha]^{20}_D$ = 7.13 (c = 1.0 g/L, $CHCl_3$). The absolute configuration was assigned by analogy to that of **4b**. **HPLC condition**: Chiral column OJ-H, n-hexane/i-PrOH = 95:5, flow rate = 1 mL/min, wavelength = 254 nm, tR = 40.35major isomer, tR = 35.99 minor isomer.

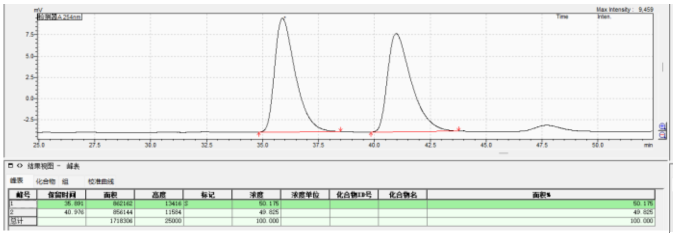

峰号	保留时间	面积	高度	标记	浓度	浓度单位	化合物ID号	化合物名	面积%
1	35.891	862162	13416	S	50.175				50.175
2	40.976	856144	11584		49.825				49.825
总计		1718306	25000		100.000				100.000

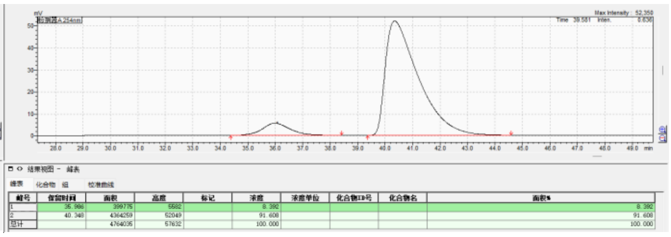

峰号	保留时间	面积	高度	标记	浓度	浓度单位	化合物ID号	化合物名	面积%
1	35.986	399775	5582		8.392				8.392
2	40.348	4364259	52049		91.608				91.608
总计		4764035	57632		100.000				100.000

(*R*)-*N*-(2-cyclohexyl-2-morpholinoethyl)benzamide (4y)

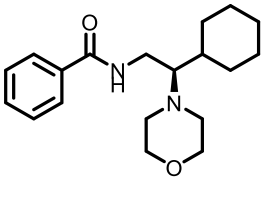

From *N*-(2-cyclohexylideneethyl)benzamide (**1x**) (45.8 mg, 0.20 mmol, 1.0 equiv) and morpholino benzoate (**2a**) (62.1 mg, 0.30 mmol, 1.5 equiv), the title compound was prepared following the general procedure **A** using $NiBr_2$·DME (6.2 mg, 10 mol%), **(*S*,*S*)-L12** (15.4 mg, 15 mol%), LiOH (9.6 mg, 0.40 mmol, 2.0 equiv), KI (33.2 mg, 0.20 mmol, 1.0 equiv), $Me(EtO)_2SiH$ (80 µL, 0.50 mmol, 2.5 equiv), Anhydrous *tert*-butyl alcohol (1.0 mL, 0.20 M). The reaction mixture was stirred for 12 h at 30 °C. The crude material was

purified by flash column chromatography (petroleum ether/EtOAc = 5:1) to provide the title compound as a yellow oil in 77% yield (48.7 mg).

^{1}H NMR (400 MHz, $CDCl_3$) δ 7.79–7.74 (m, 2H), 7.51–7.46 (m, 1H), 7.45–7.40 (m, 2H), 7.10–7.02 (m, 1H), 3.74–3.63 (m, 5H), 3.21–3.11 (m, 1H), 2.83–2.76 (m, 2H), 2.65–2.58 (m, 2H), 2.43–2.36 (m, 1H), 1.78–1.71 (m, 4H), 1.69–1.61 (m, 2H), 1.23–1.02 (m, 5H); **^{13}C NMR** (101 MHz, $CDCl_3$) δ 167.0, 134.8, 131.4, 128.6, 126.8, 68.3, 68.0, 49.5, 37.5, 37.4, 32.5, 29.9, 29.7, 26.5, 26.3. **HRMS** (ESI) m/z calculated for $C_{19}H_{28}N_2O_2$ $[M+H]^+$ 317.2224 found: 317.2225. **Optical rotation**: $[\alpha]^{20}_D$ = -41.6 (c = 1.0 g/L, $CHCl_3$). The absolute configuration was assigned by analogy to that of **4b**. **HPLC condition**: Chiral column AD-H, n-hexane/i-PrOH = 95:5, flow rate = 1 mL/min, wavelength = 254 nm, tR = 35.26 min for major isomer, tR = 32.25 min for minor isomer.

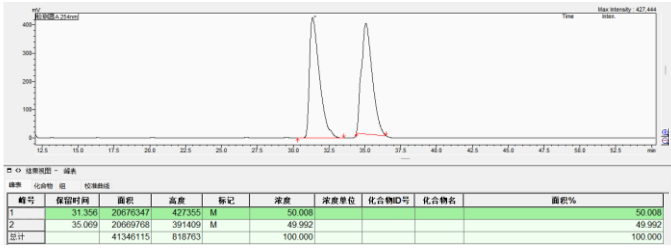

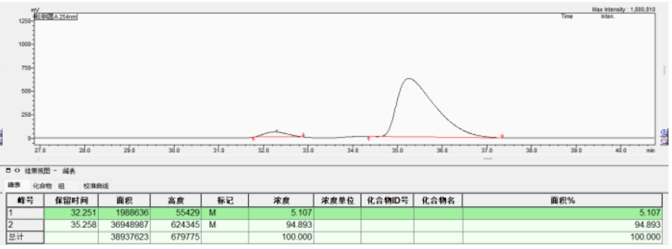

(*R*)-*N*-(2-morpholino-2-(1,4-dioxaspiro[4.5]decan-8-yl)ethyl)benzamide (4z)

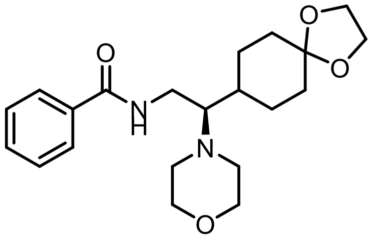

From *N*-(2-(1,4-dioxaspiro[4.5]decan-8-ylidene)ethyl)benzamide (**1x**) (57.4, 0.20 mmol, 1.0 equiv) and morpholino benzoate (**2a**) (62.1 mg, 0.30 mmol, 1.5 equiv), the title compound was prepared following the general procedure **A** using $NiBr_2$·DME (6.2 mg, 10 mol%), **(*S*,*S*)-L12** (15.4 mg, 15 mol%), LiOH (9.6 mg, 0.40 mmol, 2.0 equiv), KI (33.2 mg, 0.20 mmol, 1.0 equiv), $Me(EtO)_2SiH$ (80 μL, 0.50 mmol, 2.5 equiv), Anhydrous *tert*-butyl alcohol (1.0 mL, 0.20 M). The reaction mixture was stirred for 12 h at 30 °C. The crude material was purified by flash column chromatography (petroleum ether/EtOAc = 1:2) to provide the title compound as a yellow oil in 72 yield (53.8 mg).

^{1}H NMR (400 MHz, $CDCl_3$) δ 7.70 (d, *J* = 7.5 Hz, 2H), 7.47–7.41 (m, 1H), 7.40–7.35 (m, 2H), 7.00–6.89 (m, 1H), 3.89–3.83 (m, 4H), 3.74–3.54 (m, 5H), 3.10 (t, *J* = 11.4 Hz, 1H), 2.79–2.67 (m, 2H), 2.63–2.53 (m, 2H), 2.46–2.36 (m, 1H), 1.81–1.56 (m, 5H), 1.55–1.27 (m, 4H). **^{13}C NMR** (101 MHz, $CDCl_3$) δ 167. 134.7, 131.4 128.6, 126.8 108.3, 68.0, 67.4, 64.3, 64.3 49.4, 37.8 36.6, 34.6, 34.4, 29.4, 26.9. **HRMS** (ESI)

m/z calculated for $C_{21}H_{30}N_2O_4$ $[M+H]^+$ 375. 2279 found: 375. 2279. **Optical rotation**: $[\alpha]^{20}_D$ = -17.25 (c = 1.0 g/L, $CHCl_3$). The absolute configuration was assigned by analogy to that of **4b**. **HPLC condition**: Chiral column AD-H, n-hexane/i-PrOH = 90:10, flow rate = 1 mL/min, wavelength = 254 nm, tR = 19.92 for major isomer, tR = 16.20 for minor isomer.

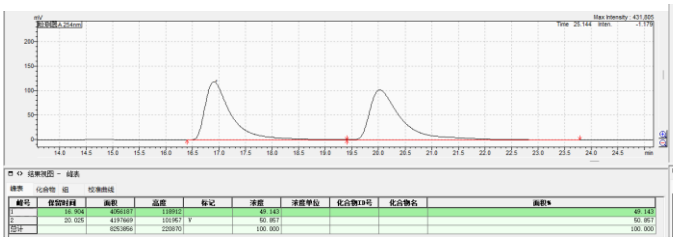

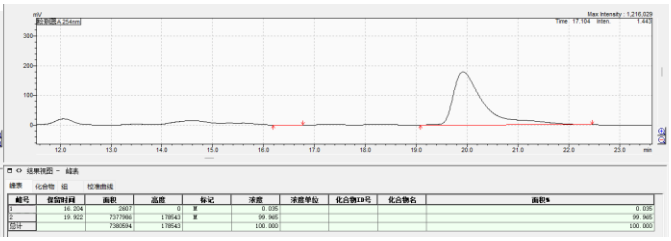

***N*-((2*R*)-2-morpholino-2-(1,2,3,4-tetrahydronaphthalen-1-yl)ethyl)benzamide (4aa)**

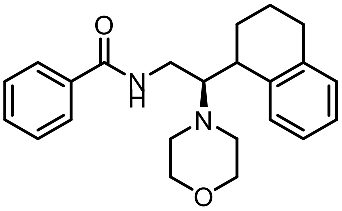

From (E)-N-(2-(3,4-dihydronaphthalen-1(2H)-ylidene)ethyl)benzamide (**1am**) (55.4, 0.20 mmol, 1.0 equiv) and morpholino benzoate (**2a**) (62.1 mg, 0.30 mmol, 1.5 equiv), the title compound was prepared following the general procedure **A** using $NiBr_2$·DME (6.2 mg, 10 mol%), **(*S*,*S*)-L2** (7.56 mg, 15 mol%), LiOH (9.6 mg, 0.40 mmol, 2.0 equiv), KI (33.2 mg, 0.20 mmol, 1.0 equiv), $Me(EtO)_2SiH$ (80 µL, 0.50 mmol, 2.5 equiv), Anhydrous *tert*-butyl alcohol (1.0 mL, 0.20 M). The reaction mixture was stirred for 12 h at 30 °C. The crude material was purified by flash column chromatography (petroleum ether/EtOAc = 1:2) to provide the title compound as a yellow oil in 26 yield (18.9 mg).

^{1}H NMR (400 MHz, $CDCl_3$) δ 7.59 (d, *J* = 7.3 Hz, 2H), 7.44–7.39 (m, 1H), 7.38–7.32 (m, 2H), 7.13 (d, *J* = 7.2 Hz, 1H), 7.10–6.97 (m, 3H), 6.62 (s, 1H), 3.66 (d, *J* = 16.3 Hz, 4H), 3.39 (t, *J* = 5.3 Hz, 2H), 3.12 (s, 1H), 2.77 (d, *J* = 10.3 Hz, 2H), 2.70–2.58 (m, 3H), 1.93–1.81 (m, 2H), 1.73 (d, *J* = 10.2 Hz, 1H), 1.65–1.52 (m, 2H), 1.28–1.13 (m, 2H). **^{13}C NMR** (101 MHz, $CDCl_3$) δ 170.5 138.7, 131.5 129.9, 129.6 128.6, 128.6 1280 127.1, 126.8 126.0, 68.4, 67.8 66.9, 60.5 50.1, 37.2 36.5 29.8 25.4 21.6 **HRMS** (ESI) m/z calculated for $C_{23}H_{28}N_2O_2$ $[M+H]^+$ 365.2224 found: 365.2224. **Optical rotation**: $[\alpha]^{20}_D$ = -50.4 (c = 1.0 g/L, $CHCl_3$). The absolute configuration was assigned by analogy to that of **4b**. **HPLC condition**: Chiral column AD-H, n-hexane/i-PrOH = 90:10, flow rate = 1 mL/min, wavelength = 254 nm, tR = 19.80 for major isomer, tR = 15.21 for minor isomer.

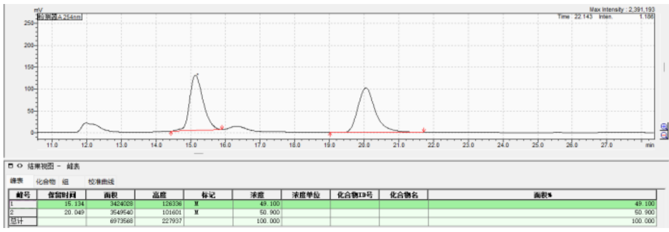

峰号	保留时间	面积	高度	标记	浓度	浓度单位	化合物ID号	化合物名	面积%
1	15.134	3424028	126336	M	49.100				49.100
2	20.049	3549540	101601	M	50.900				50.900
总计		6973568	227937		100.000				100.000

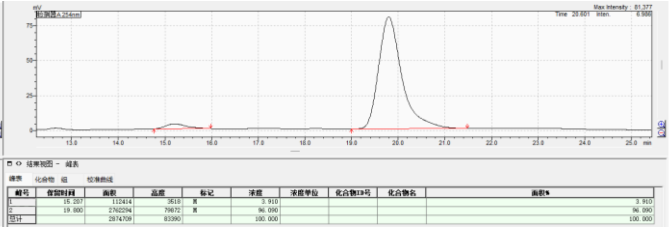

峰号	保留时间	面积	高度	标记	浓度	浓度单位	化合物ID号	化合物名	面积%
1	15.207	112414	3518	M	3.910				3.910
2	19.800	2762294	79872	M	96.090				96.090
总计		2874709	83390		100.000				100.000

5. Unsuccessful substrates

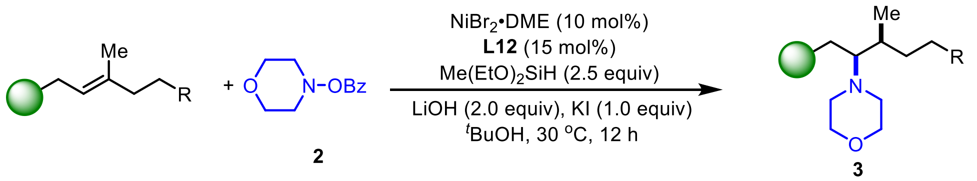

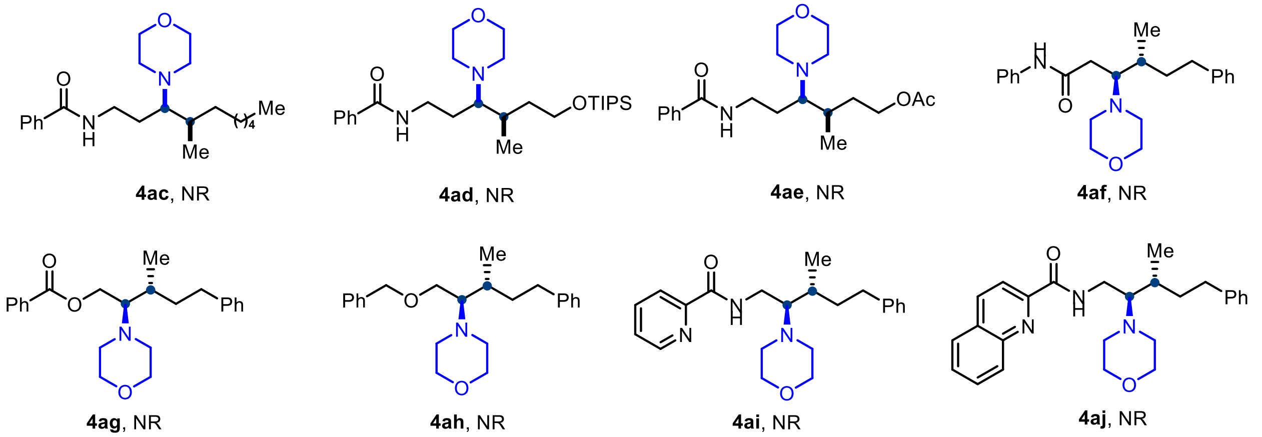

In an Ar-filled glovebox, a 4 mL reaction vials equipped with a magnetic stir bar was added NiBr2•DME (0.02 mmol, 10 mol%), L12 (0.030 mmol, 15 mol%), tri-substituted alkene substrate (0.2 mmol, 1.0 equiv.), LiOH (0.5 mmol, 2.5 equiv.), KI (0.2 mmol, 1.0 equiv), 2a (0.3 mmol, 1.5 equiv.), tBuOH (1.0 mL) and Me(OEt)2SiH (0.5 mmol, 2.5 equiv.). The reaction mixture was stirred at 30 °C for 12 h. After completion, The reaction mixture was periodically sampled and analyzed by gas chromatography-mass spectrometry (GC-MS) to track reaction progress and identify intermediates or by-products.

6. Stereodivergent Synthesis

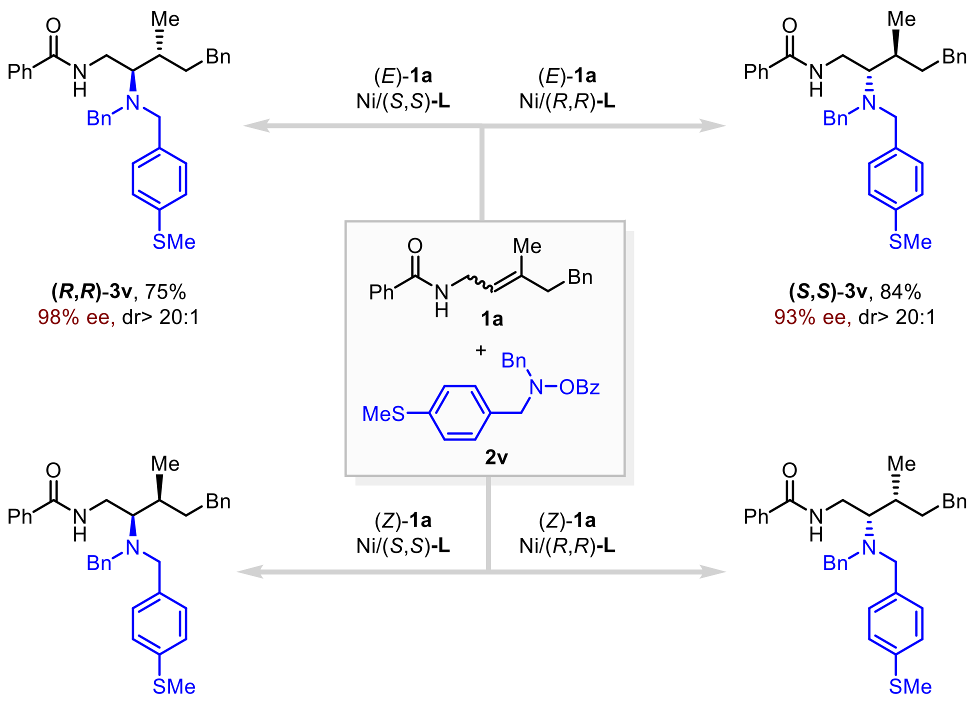

N-((2*S*,3*S*)-2-(benzyl(4-(methylthio)benzyl)amino)-3-methyl-5-phenylpentyl)benzamide ((*S*,*S*)-3v)

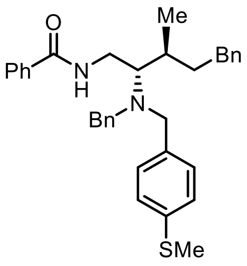

From (*E*)-N-(3-methyl-5-phenylpent-2-en-1-yl)benzamide (**1a**) (55.8 mg, 0.20 mmol, 1.0 equiv) and *O*-benzoyl-*N*-benzyl-*N*-(4-(methylthio)benzyl)hydroxylamine (**2v**) (108.9 mg, 0.30 mmol, 1.5 equiv), the title compound was prepared following the general procedure **A** using $NiBr_2$·DME (6.2 mg, 10 mol%), **(*R*,*R*)-L12** (15.4 mg, 15 mol%), LiOH (9.6 mg, 0.40 mmol, 2.0 equiv), KI (33.2 mg, 0.20 mmol, 1.0 equiv), $Me(EtO)_2SiH$ (80 µL, 0.50 mmol, 2.5 equiv), Anhydrous *tert*-butyl alcohol (1.0 mL, 0.20 M). The reaction mixture was stirred for 12 h at 30 °C. The crude material was purified by flash column chromatography (petroleum ether/EtOAc = 10:1) to provide the title compound as a yellow oil in 84% yield (87.7 mg).

^{1}H NMR (400 MHz, $CDCl_3$) δ 7.64–7.59 (m, 2H), 7.58–7.52 (m, 1H), 7.50–7.45 (m, 2H), 7.40–7.33 (m, 2H), 7.29–7.23 (m, 6H), 7.21–7.16 (m, 2H), 7.15–7.11 (m, 2H), 7.10–7.05 (m, 2H), 6.54–6.42 (m, 1H), 3.85 (t, *J* = 13.6 Hz, 2H), 3.69–3.58 (m, 1H), 3.50 (t, *J* = 13.0 Hz, 2H), 3.45–3.35 (m, 1H), 2.85–2.76 (m, 1H), 2.75–2.69 (m, 1H), 2.67–2.58 (m, 1H), 2.46 (s, 3H), 2.13 (s, 1H), 1.87–1.77 (m, 1H), 1.70–1.56 (m, 1H), 1.14 (d, *J* = 6.6 Hz, 2H). **^{13}C NMR** (101 MHz, $CDCl_3$) δ 167.3, 142.2, 139.9, 137.2, 136.8, 134.7,

131.3, 129.6, 129.2, 128.6, 128.5, 127.3, 126.9, 126.7, 126.0, 61.3, 53.8, 53.4, 37.9, 37.5, 33.5, 30.6, 16.5, 15.9. **HRMS** (ESI) m/z calculated for $C_{34}H_{38}N_2OS$ $[M+H]^+$ 523.2778 found: 523.2778. **Optical rotation**: $[\alpha]^{20}_D$ = -17.8 (c = 1.0 g/L, $CHCl_3$). The absolute configuration was assigned by analogy to that of **4b**. **HPLC condition**: Chiral column OD-H, n-hexane/i-PrOH = 95:5, flow rate = 1 mL/min, wavelength = 254 nm, tR = 39.8 min for major isomer, tR = 34.4 min for minor isomer.

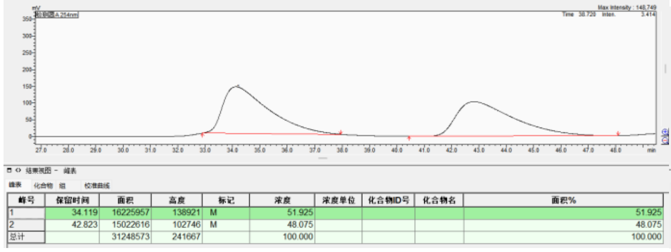

峰号	保留时间	面积	高度	标记	浓度	浓度单位	化合物ID号	化合物名	面积%
1	34.119	16225957	138921	M	51.925				51.925
2	42.823	15022616	102746	M	48.075				48.075
总计		31248573	241667		100.000				100.000

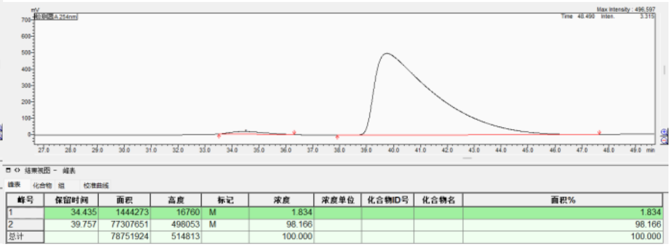

峰号	保留时间	面积	高度	标记	浓度	浓度单位	化合物ID号	化合物名	面积%
1	34.435	1444273	16760	M	1.834				1.834
2	39.757	77307651	498053	M	98.166				98.166
总计		78751924	514813		100.000				100.000

***N*-((2*R*,3*S*)-2-(benzyl(4-(methylthio)benzyl)amino)-3-methyl-5-phenylpentyl)benzamide ((*R*,*S*)-3v)**

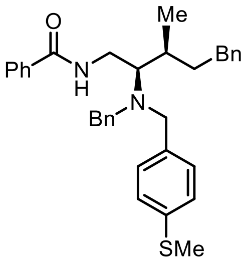

From (*Z*)-*N*-(3-methyl-5-phenylpent-2-en-1-yl)benzamide (**1a**) (55.8 mg, 0.20 mmol, 1.0 equiv) and *O*-benzoyl-*N*-benzyl-*N*-(4-(methylthio)benzyl)hydroxylamine (**2v**) (108.9 mg, 0.30 mmol, 1.5 equiv), the title compound was prepared following the general procedure **A** using $NiBr_2$·DME (6.2 mg, 10 mol%), **(*S*,*S*)-L12** (15.4 mg, 15 mol%), LiOH (9.6 mg, 0.40 mmol, 2.0 equiv), KI (33.2 mg, 0.20 mmol, 1.0 equiv), $Me(EtO)_2SiH$ (80 µL, 0.50 mmol, 2.5 equiv), Anhydrous *tert*-butyl alcohol (1.0 mL, 0.20 M). The reaction mixture was stirred for 12 h at 30 °C. The crude material was purified by flash column chromatography (petroleum ether/EtOAc = 10:1) to provide the title compound as a yellow oil in 70% yield (73.0 mg).

^{1}H NMR (400 MHz, $CDCl_3$) δ 7.65–7.61 (m, 2H), 7.60–7.55 (m, 1H), 7.50 (t, *J* = 7.5 Hz, 2H), 7.39–7.34 (m, 2H), 7.33–7.22 (m, 8H), 7.21–7.12 (m, 4H), 6.46 (d, *J* = 6.3 Hz, 1H), 3.91–3.80 (m, 2H), 3.78–3.69 (m, 1H), 3.63–3.50 (m, 2H), 3.43–3.32 (m, 1H), 2.92–2.80 (m, 1H), 2.79–2.71 (m, 1H), 2.68–2.56 (m, 1H), 2.49 (s, 3H), 2.14–2.04 (m, 1H), 2.02–1.92 (m, 1H), 1.65–1.48 (m, 1H), 1.25 (d, *J* = 6.8 Hz, 3H). **^{13}C NMR** (101 MHz, $CDCl_3$) δ 167.3, 142.3, 139.9, 137.2, 136.8, 134.7, 131.3, 129.7, 129.2, 128.6, 128.5, 128.4, 127.3, 126.9,126.8, 126.7, 126.0, 61.7, 53.9, 53.6, 37.6, 35.3, 33.5, 31.7, 18.9, 15.9. **HRMS** (ESI) m/z calculated for $C_{34}H_{38}N_2OS$ $[M+H]^+$ 523.2778 found: 523.2778. **Optical rotation**: $[\alpha]^{20}_D$ =71.2 (c = 1.0 g/L, $CHCl_3$). The absolute configuration was assigned by analogy to that of **4b**. **HPLC condition**: Chiral column OD-H, n-hexane/i-PrOH = 95:5, flow rate = 1 mL/min, wavelength = 254 nm, tR = 48.1 min for major isomer, tR = 33.2 min for minor isomer.

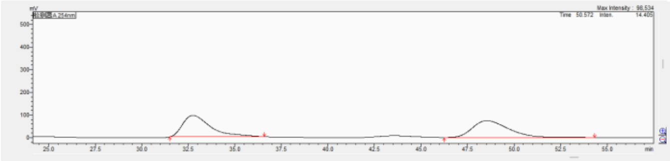

峰号	保留时间	面积	高度	标记	浓度	浓度单位	化合物ID号	化合物名	面积%
1	32.753	9971736	94880	M	50.192				50.192
2	48.542	9895540	74301	M	49.808				49.808
总计		19867276	169180		100.000				100.000

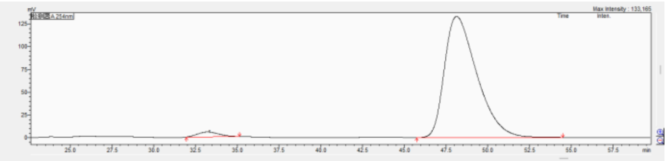

峰号	保留时间	面积	高度	标记	浓度	浓度单位	化合物ID号	化合物名	面积%
1	33.201	470686	5278	M	2.563				2.563
2	48.128	17895766	132797	S	97.437				97.437
总计		18366451	138075		100.000				100.000

***N*-((2*S*,3*R*)-2-(benzyl(4-(methylthio)benzyl)amino)-3-methyl-5-phenylpentyl)benzamide ((*S*,*R*)-3v)**

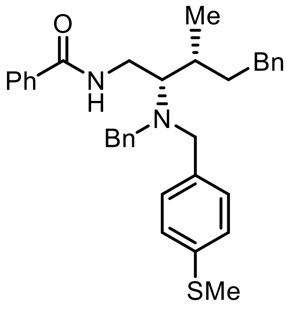

From (*Z*)-*N*-(3-methyl-5-phenylpent-2-en-1-yl)benzamide (**1a**) (55.8 mg, 0.20 mmol, 1.0 equiv) and *O*-benzoyl-*N*-benzyl-*N*-(4-(methylthio)benzyl)hydroxylamine (**2v**) (108.9 mg, 0.30 mmol, 1.5 equiv), the title compound was prepared following the general procedure **A** using $NiBr_2$·DME (6.2 mg, 10 mol%), **(R,R)-L12** (15.4 mg, 15 mol%), LiOH (9.6 mg, 0.40 mmol, 2.0 equiv), KI (33.2 mg, 0.20 mmol, 1.0 equiv), $Me(EtO)_2SiH$ (80 μL, 0.50 mmol, 2.5 equiv), Anhydrous *tert*-butyl alcohol (1.0 mL, 0.20 M). The reaction mixture was stirred for 12 h at 30 °C. The crude material was purified by flash column chromatography (petroleum ether/EtOAc = 10:1) to provide the title compound as a yellow oil in 72% yield (75.1 mg).

^{1}H NMR (400 MHz, $CDCl_3$) δ 7.61–7.57 (m, 2H), 7.56–7.52 (m, 1H), 7.49–7.43 (m, 2H), 7.37–7.31 (m, 2H), 7.30–7.18 (m, 8H), 7.17–7.08 (m, 4H), 6.47–6.39 (m, 1H), 3.82 (t, *J* = 13.7 Hz, 2H), 3.76–3.66 (m, 1H), 3.53 (t, *J* = 13.8 Hz, 2H), 3.40–3.29 (m, 1H), 2.87–2.77 (m, 1H), 2.76–2.67 (m, 1H), 2.66–2.53 (m, 1H), 2.46 (s, 3H), 2.10–2.01 (m, 1H), 1.99–1.88 (m, 1H), 1.58–1.49 (m, 1H), 1.22 (d, *J* = 6.8 Hz, 3H). **^{13}C NMR** (101 MHz, $CDCl_3$) δ 167.3, 142.2, 139.9, 137.2, 136.8, 134.7, 131.3, 129.7, 129.2, 128.6, 128.5, 128.4, 127.3, 126.9, 126.8, 126.0, 61.7, 53.9, 53.6, 37.6, 35.3, 33.5, 31.7, 18.94, 15.9. **HRMS** (ESI) m/z calculated for $C_{34}H_{38}N_2OS$ $[M+H]^+$ 523.2778 found: 523.2778. **Optical rotation**: $[\alpha]^{20}_D$ = -28.9 (c = 1.0 g/L, $CHCl_3$). The absolute configuration was assigned by analogy to that of **4b**. **HPLC condition**: Chiral column OD-H, n-hexane/i-PrOH = 95:5, flow rate = 1 mL/min, wavelength = 254 nm, tR = 31.7 min for major isomer, tR = 48.3 min for minor isomer.

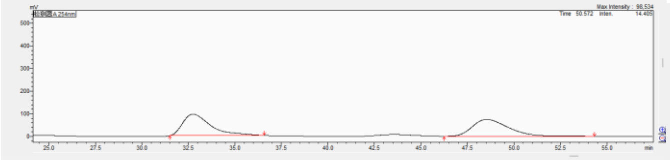

峰号	保留时间	面积	高度	标记	浓度	浓度单位	化合物ID号	化合物名	面积%
1	32.753	9971736	94880	M	50.192				50.192
2	48.542	9895540	74301	M	49.808				49.808
总计		19867276	169180		100.000				100.000

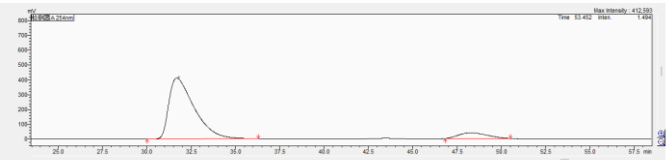

峰号	保留时间	面积	高度	标记	浓度	浓度单位	化合物ID号	化合物名	面积%
1	31.687	41098100	410973	M	90.505				90.505
2	48.281	4311842	38477	M	9.495				9.495
总计		45409942	449451		100.000				100.000

7. Late-stage decoration of drug-like molecules

2-(3-Cyano-4-isobutoxyphenyl)-4-methyl-*N*-((2*R*,3*R*)-3-methyl-2-morpholino-5-phenylpentyl)thiazole-5-carboxamide (5a)

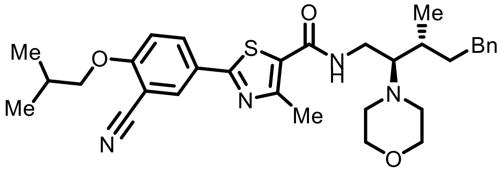

From (*E*)-2-(3-cyano-4-isobutoxyphenyl)-4-methyl-*N*-(3-methyl-5-phenylpent-2-en-1-yl)thiazole-5-carboxamide (**1y**) (94.6 mg, 0.20 mmol, 1.0 equiv) and morpholino benzoate (**2a**) (62.1 mg, 0.30 mmol, 1.5 equiv), the title compound was prepared following the general procedure **A** using $NiBr_2 \cdot DME$ (6.2 mg, 10 mol%), **(*S*,*S*)-L12** (15.4 mg, 15 mol%), LiOH (9.6 mg, 0.40 mmol, 2.0 equiv), KI (33.2 mg, 0.20 mmol, 1.0 equiv), $Me(EtO)_2SiH$ (80 µL, 0.50 mmol, 2.5 equiv), Anhydrous *tert*-butyl alcohol (1.0 mL, 0.20 M). The reaction mixture was stirred for 12 h at 30 °C. The crude material was purified by flash column chromatography (petroleum ether/EtOAc = 3:1) to provide the title compound as a yellow oil in 63% yield (70.6 mg).

^{1}H NMR (400 MHz, $CDCl_3$) δ 8.17 (d, *J* = 2.2 Hz, 1H), 8.11–8.06 (m, 1H), 7.33–7.30 (m, 2H), 7.24–7.20 (m, 3H), 7.03 (d, *J* = 8.9 Hz, 1H), 6.82–6.76 (m, 1H), 3.92 (d, *J* = 6.5 Hz, 2H), 3.77–3.69 (m, 5H), 3.32–3.22 (m, 1H), 2.80–2.76 (m, 5H), 2.75–2.71 (m, 1H), 2.67–2.62 (m, 1H), 2.61–2.56 (m, 3H), 2.27–2.21 (m, 1H), 2.01–1.95 (m, 1H), 1.84–1.76 (m, 1H), 1.68–1.59 (m, 1H), 1.12 (d, *J* = 6.7 Hz, 6H), 1.05 (d, *J* = 6.8 Hz, 3H); **^{13}C NMR** (101 MHz, $CDCl_3$) δ 188.9, 164.7, 162.3, 161.3, 155.1, 141.9, 132.5, 131.9, 128.5, 128.4, 126.0, 126.0, 115.5, 112.6, 102.9, 75.7, 67.8, 66.9, 49.5, 37.6, 36.9, 33.4, 30.8, 28.2, 19.1, 17.6, 16.5. **HRMS** (ESI) m/z calculated for $C_{32}H_{40}N_4O_3S$ $[M+Na]^+$ 583.2713 found: 583.2714. **Optical rotation**: $[\alpha]^{20}_D$ = -21.6 (c = 1.0 g/L, $CHCl_3$). The absolute configuration was assigned by analogy to that of **4b**. **HPLC condition**: Chiral column AD-H, n-hexane/i-PrOH = 95:5, flow rate = 1 mL/min, wavelength = 254 nm, tR = 59.2 min for major isomer, tR = 53.3 min for minor isomer.

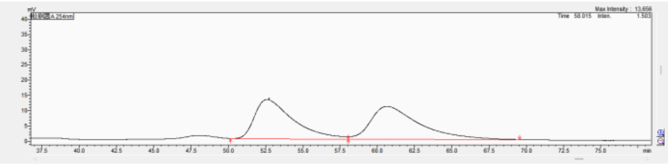

峰号	保留时间	面积	高度	标记	浓度	浓度单位	化合物ID号	化合物名	面积%
1	52.597	2301479	12787	M	48.778				48.778
2	60.660	2416764	10713	V M	51.222				51.222
总计		4718243	23500		100.000				100.000

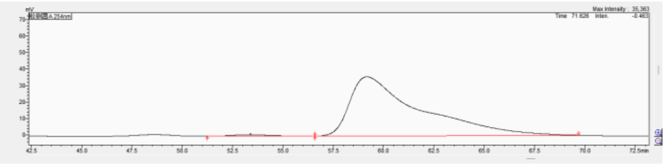

峰号	保留时间	面积	高度	标记	浓度	浓度单位	化合物ID号	化合物名	面积%
1	53.293	107010	806	M	1.223				1.223
2	59.172	8643524	35830	M	98.777				98.777
总计		8750534	36636		100.000				100.000

N-((2R,3R)-3-methyl-2-morpholino-5-phenylpentyl)-2-(11-oxo-6,11-dihydrodibenzo[b,e]oxepin-2-yl)acetamide (5b)

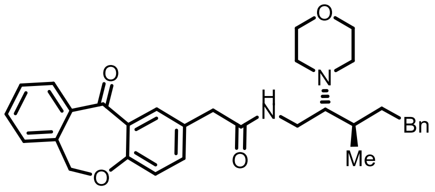

From (*E*)-*N*-(3-methyl-5-phenylpent-2-en-1-yl)-2-(11-oxo-6,11-dihydrodibenzo[b,e]oxepin-2-yl)acetamide (**1z**) (94.6 mg, 0.20 mmol, 1.0 equiv) and morpholino benzoate (**2a**) (85.0 mg, 0.30 mmol, 1.5 equiv), the title compound was prepared following the general procedure **A** using $NiBr_2$·DME (6.2 mg, 10 mol%), **(*S*,*S*)-L12** (15.4 mg, 15 mol%), LiOH (9.6 mg, 0.40 mmol, 2.0 equiv), KI (33.2 mg, 0.20 mmol, 1.0 equiv), $Me(EtO)_2SiH$ (80 μL, 0.50 mmol, 2.5 equiv), Anhydrous *tert*-butyl alcohol (1.0 mL, 0.20 M). The reaction mixture was stirred for 12 h at 30 °C. The crude material was purified by flash column chromatography (petroleum ether/EtOAc = 3:1) to provide the title compound as a yellow oil in 36% yield (36.9 mg).

^{1}H NMR (400 MHz, $CDCl_3$) δ 8.12 (d, J = 2.3 Hz, 1H), 7.89–7.86 (m, 1H), 7.60–7.55 (m, 1H), 7.50–7.46 (m, 1H), 7.42–7.37 (m, 2H), 7.28–7.26 (m, 1H), 7.25–7.23 (m, 1H), 7.18–7.13 (m, 3H), 7.09–7.05 (m, 1H), 6.19 (s, 1H), 5.19 (s, 2H), 3.66–3.54 (m, 3H), 3.51–3.45 (m, 1H), 3.40–3.23 (m, 4H), 3.04–2.93 (m, 1H), 2.66–2.61 (m, 1H), 2.55–2.45 (m, 3H), 2.33–2.28 (m, 2H), 1.82–1.75 (m, 1H), 1.70–1.63 (m, 1H), 1.54–1.45 (m, 1H), 0.89 (d, J = 6.7 Hz, 3H); **^{13}C NMR** (101 MHz, $CDCl_3$) δ 190.9, 160.6, 142.1, 140.4, 136.7, 135.4, 133.0, 132.6, 129.4, 128.9, 128.4, 128.3, 128.0, 125.9, 125.4, 121.6, 73.7, 67.4, 66.7, 49.3, 42.9, 37.4, 36.4, 33.3, 31.0, 16.3. **HRMS** (ESI) m/z calculated for $C_{32}H_{36}N_2O_4$ $[M+H]^+$ 513.2748 found: 513.2748. **Optical rotation**: $[\alpha]^{20}_D$ = -8.77 (c = 1.0 g/L, $CHCl_3$). The absolute configuration was assigned by analogy to that of **4b**. **HPLC condition**: Chiral column AD-H, n-hexane/i-PrOH = 85:15, flow rate = 1 mL/min, wavelength = 254 nm, tR = 25.288 min for major isomer, tR = 29.39 min for minor isomer.

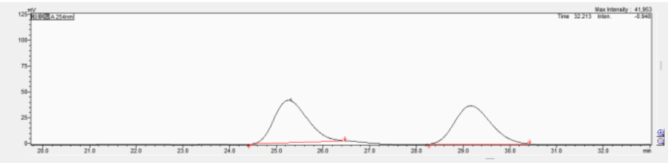

峰号	保留时间	面积	高度	标记	浓度	浓度单位	化合物ID号	化合物名	面积%
1	25.262	2024974	41107	M	50.583				50.583
2	29.164	1978313	36884	M	49.417				49.417
总计		4003287	77991		100.000				100.000

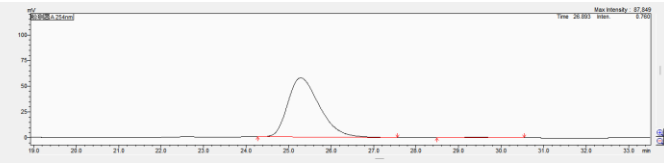

峰号	保留时间	面积	高度	标记	浓度	浓度单位	化合物ID号	化合物名	面积%
1	25.288	2979558	57486	M	98.882				98.882
2	29.390	33678	577	M	1.118				1.118
总计		3013237	58064		100.000				100.000

N-((2R,3R)-3-methyl-2-(methyl((R)-3-phenyl-3-(o-tolyloxy)propyl)amino)-5-phenylpentyl)benzamide (5c)

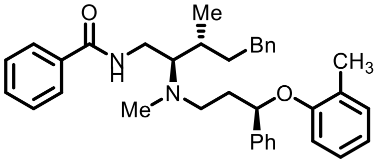

From (*E*)-*N*-(3-methyl-5-phenylpent-2-en-1-yl)benzamide (**1a**) (55.8 mg, 0.20 mmol, 1.0 equiv) and (*R*)-*O*-benzoyl-*N*-methyl-*N*-(3-phenyl-3-(o-tolyloxy)propyl)hydroxylamine (**2z**) (112.5 mg, 0.30 mmol, 1.5 equiv), the title compound was prepared following the general procedure **A** using $NiBr_2$·DME (6.2 mg, 10 mol%), **(*S*,*S*)-L12** (15.4 mg, 15 mol%), LiOH (9.6 mg, 0.40 mmol, 2.0 equiv), KI (33.2 mg, 0.20 mmol, 1.0 equiv), $Me(EtO)_2SiH$ (80 µL, 0.50 mmol, 2.5 equiv), Anhydrous *tert*-butyl alcohol (1.0 mL, 0.20 M). The reaction mixture was stirred for 12 h at 30 °C. The crude material was purified by flash column chromatography (petroleum ether/EtOAc = 3:1) to provide the title compound as a yellow oil in 46% yield (49.2 mg).

^{1}H NMR (400 MHz, $CDCl_3$) δ 7.73 (d, *J* = 7.6 Hz, 2H), 7.50–7.45 (m, 1H), 7.42–7.37 (m, 2H), 7.31–7.24 (m, 7H), 7.20–7.15 (m, 3H), 7.13–7.07 (m, 1H), 6.91–6.86 (m, 1H), 6.85–6.80 (m, 1H), 6.79–6.73 (m, 1H), 6.52–6.46 (m, 1H), 5.19–5.12 (m, 1H), 3.79–3.66 (m, 1H), 3.26–3.13 (m, 1H), 2.94–2.84 (m, 1H), 2.76–2.66 (m, 2H), 2.63–2.50 (m, 2H), 2.33 (s, 3H), 2.28 (s, 3H), 2.17–2.09 (m, 1H), 2.07–1.98 (m, 1H), 1.90–1.82 (m, 1H), 1.80–1.73 (m, 1H), 1.57–1.46 (m, 1H), 1.01 (d, *J* = 6.7 Hz, 3H). **^{13}C NMR** (101 MHz, $CDCl_3$) δ 167.2, 156.0, 142.3, 142.1, 135.0, 131.3, 130.7, 128.7, 128.6, 128.4, 127.6, 127.0, 126.9, 126.7, 125.9, 125.7, 120.3, 112.7, 77.8, 66.6, 51.4, 38.7, 37.9, 37.4, 37.0, 33.4, 31.7, 16.7, 16.6. **HRMS** (ESI) m/z calculated for $C_{36}H_{42}N_2O_2$ $[M+H]^+$ 535.3319 found: 535.3319. **Optical rotation**: $[\alpha]^{20}_D$ = -191.3 (c = 1.0 g/L, $CHCl_3$). The absolute configuration was assigned by analogy to that of **4b**. **HPLC condition**: Chiral column OD-H, n-hexane/i-PrOH = 95:5, flow rate = 1 mL/min, wavelength = 254 nm, tR = 40.52 min for major isomer, tR = 31.84 min for minor isomer.

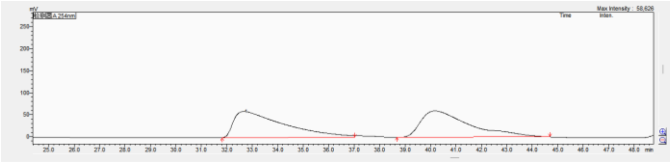

结果视图 - 峰表

峰号	保留时间	面积	高度	标记	浓度	浓度单位	化合物ID号	化合物名	面积%
1	32.664	8211703	59160	M	50.558				50.558
2	40.175	8030493	59777	M	49.442				49.442
总计		16242195	118938		100.000				100.000

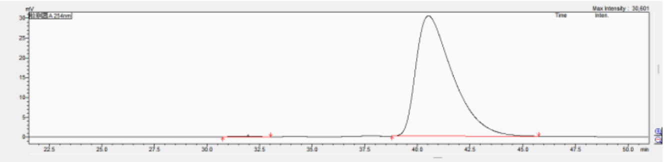

结果视图 - 峰表

峰号	保留时间	面积	高度	标记	浓度	浓度单位	化合物ID号	化合物名	面积%
1	31.843	9620	145	M	0.260				0.260
2	40.523	3686562	30359		99.740				99.740
总计		3696182	30505		100.000				100.000

N-((2*R*,3*R*)-3-methyl-2-(methyl((*S*)-3-(naphthalen-1-yloxy)-3-(thiophen-2-yl)propyl)amino)-5-phenylpentyl)benzamide (5d)

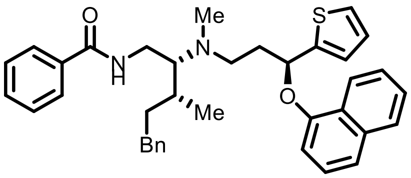

From (*E*)-*N*-(3-methyl-5-phenylpent-2-en-1-yl)benzamide (**1a**) (55.8 mg, 0.20 mmol, 1.0 equiv) and (*S*)-*O*-benzoyl-*N*-methyl-*N*-(3-(naphthalen-1-yloxy)-3-(thiophen-2-yl)propyl)hydroxylamine (**2aa**) (125.1 mg, 0.30 mmol, 1.5 equiv), the title compound was prepared following the general procedure **A** using $NiBr_2$·DME (6.2 mg, 10 mol%), **(*S*,*S*)-L12** (15.4 mg, 15 mol%), LiOH (9.6 mg, 0.40 mmol, 2.0 equiv), KI (33.2 mg, 0.20 mmol, 1.0 equiv), $Me(EtO)_2SiH$ (80 µL, 0.50 mmol, 2.5 equiv), Anhydrous *tert*-butyl alcohol (1.0 mL, 0.20 M). The reaction mixture was stirred for 12 h at 30 °C. The crude material was purified by flash column chromatography (petroleum ether/EtOAc = 3:1) to provide the title compound as a yellow oil in 43% yield (49.6 mg).

^{1}H NMR (400 MHz, $CDCl_3$) δ 8.46–8.37 (m, 1H), 7.90–7.82 (m, 3H), 7.62–7.56 (m, 3H), 7.52–7.47 (m, 3H), 7.37–7.33 (m, 2H), 7.30–7.24 (m, 5H), 7.07–7.04 (m, 1H), 7.02–6.99 (m, 1H), 6.89–6.78 (m, 2H), 5.85–5.71 (m, 1H), 3.89–3.79 (m, 1H), 3.31–3.18 (m, 1H), 3.07–2.97 (m, 1H), 2.94–2.85 (m, 1H), 2.82–2.69 (m, 2H), 2.67–2.60 (m, 1H), 2.58–2.50 (m, 1H), 2.44 (s, 3H), 2.35–2.25 (m, 1H), 1.97–1.86 (m, 2H), 1.63–1.53 (m, 1H), 1.09 (d, *J* = 6.6 Hz, 3H); **^{13}C NMR** (101 MHz, $CDCl_3$) δ 167.1, 153.3, 145.2, 142.3, 134.9, 134.6, 131.3, 128.6, 128.4, 127.6, 126.9, 126.7, 126.4, 126.1, 125.8, 125.7, 125.4, 124.8, 124.6, 122.0, 120.8, 107.0, 74.4, 66.8, 51.2, 38.5, 37.8, 37.3, 36.8, 33.3, 31.5, 16.7. **HRMS** (ESI) m/z calculated for $C_{37}H_{40}N_2O_2S$ $[M+H]^+$ 577.2883 found: 577.2883. **Optical rotation**: $[\alpha]^{20}_D$ = 81.1 (c = 1.0 g/L, $CHCl_3$). The absolute configuration was assigned by analogy to that of **4b**. **HPLC condition**: Chiral column IC, n-hexane/i-PrOH = 97:3, flow rate = 0.8 mL/min, wavelength = 254 nm, tR = 123.93 min for major isomer, tR = 132.52 min for minor isomer.

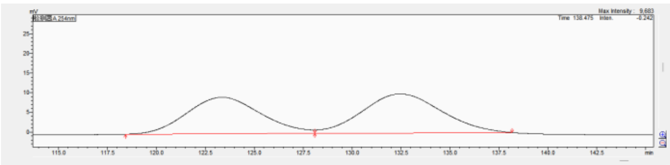

峰号	保留时间	面积	高度	标记	浓度	浓度单位	化合物ID号	化合物名	面积%
1	123.353	2448918	9294	M	46.599				46.599
2	132.447	2806357	9939	V M	53.401				53.401
总计		5255275	19233		100.000				100.000

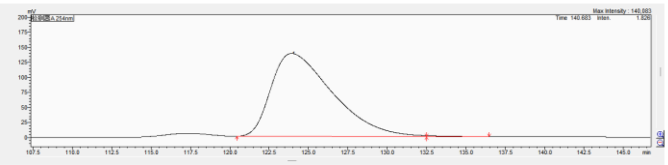

峰号	保留时间	面积	高度	标记	浓度	浓度单位	化合物ID号	化合物名	面积%
1	123.927	37404153	138081	M	99.619				99.619
2	132.521	142895	1677	V M	0.381				0.381
总计		37547049	139758		100.000				100.000

N-((2R,3R)-2-((3-(9,10-ethanoanthracen-9(10H)-yl)propyl)(methyl)amino)-3-methyl-5-phenylpentyl)benzamide (5e)

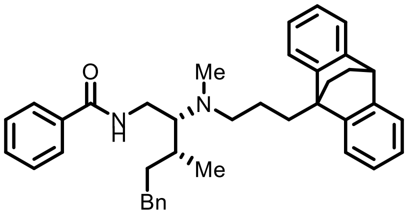

From (*E*)-*N*-(3-methyl-5-phenylpent-2-en-1-yl)benzamide (**1a**) (55.8 mg, 0.20 mmol, 1.0 equiv) and N-(3-(9,10-ethanoanthracen-9(10H)-yl)propyl)-*O*-benzoyl-*N*-methylhydroxylamine (**2ab**) (119.2 mg, 0.30 mmol, 1.5 equiv), the title compound was prepared following the general procedure **A** using NiBr$_2$·DME (6.2 mg, 10 mol%), **(*S*,*S*)-L12** (15.4 mg, 15 mol%), LiOH (9.6 mg, 0.40 mmol, 2.0 equiv), KI (33.2 mg, 0.20 mmol, 1.0 equiv), Me(EtO)$_2$SiH (80 μL, 0.50 mmol, 2.5 equiv), Anhydrous *tert*-butyl alcohol (1.0 mL, 0.20 M). The reaction mixture was stirred for 12 h at 30 °C. The crude material was purified by flash column chromatography (petroleum ether/EtOAc = 3:1) to provide the title compound as a yellow oil in 56% yield (62.3 mg).

^{1}H NMR (400 MHz, CDCl$_3$) δ 7.7 (d, *J* = 7.6 Hz, 2H), 7.4–7.3 (m, 1H), 7.3–7.3 (m, 3H), 7.3–7.2 (m, 6H), 7.2–7.1 (m, 3H), 7.1–6.9 (m, 6H), 4.3–4.2 (m, 1H), 3.9–3.8 (m, 1H), 3.3–3.2 (m, 1H), 3.0–2.9 (m, 1H), 2.8–2.7 (m, 3H), 2.7–2.6 (m, 1H), 2.5–2.4 (m, 1H), 2.4 (s, 3H), 2.0–1.9 (m, 3H), 1.9–1.8 (m, 1H), 1.8–1.8 (m, 2H), 1.7–1.6 (m, 2H), 1.6–1.5 (m, 2H), 1.1 (d, *J* = 6.7 Hz, 3H). **^{13}C NMR** (101 MHz, CDCl$_3$) δ 167.2, 145.3, 145.0, 142.3, 134.7, 131.2, 128.5, 128.4, 126.8, 125.9, 125.3, 125.3, 123.4, 123.4, 121.2, 67.0, 56.2, 44.8, 44.5, 38.0, 37.6, 36.7, 33.5, 31.6, 29.8, 28.7, 27.7, 24.4, 16.8. **HRMS** (ESI) m/z calculated for $C_{39}H_{44}N_2O$ $[M+H]^+$ 557.3527 found: 557.3498. **Optical rotation**: $[\alpha]^{20}_D$ = -9.17 (c = 1.0 g/L, CHCl$_3$). The absolute configuration was assigned by analogy to that of **4b**. **HPLC condition**: Chiral column OD-H, n-hexane/i-PrOH = 95:5, flow rate = 1 mL/min, wavelength = 254 nm, tR = 36.02 min for major isomer, tR = 28.39 min for minor isomer.

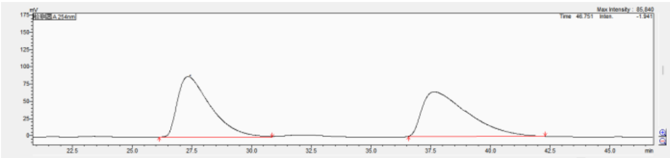

结果视图 - 峰表

峰号	保留时间	面积	高度	标记	浓度	浓度单位	化合物ID号	化合物名	面积%
1	27.340	8415598	87596		49.495				49.495
2	37.655	8587439	64580	M	50.505				50.505
总计		17003038	152177		100.000				100.000

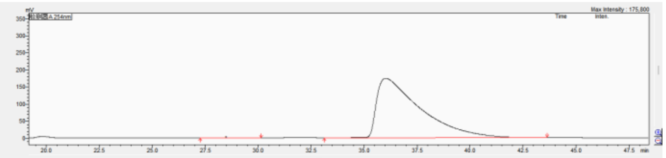

结果视图 - 峰表

峰号	保留时间	面积	高度	标记	浓度	浓度单位	化合物ID号	化合物名	面积%
1	28.389	16507	232	M	0.066				0.066
2	36.021	24867997	174284	M	99.934				99.934
总计		24884503	174516		100.000				100.000

N-((2R,3R)-2-((3-(10,11-dihydro-5H-dibenzo[a,d][7]annulen-5-ylidene)propyl)(methyl)amino)-3-methyl-5-phenylpentyl)benzamide (5f)

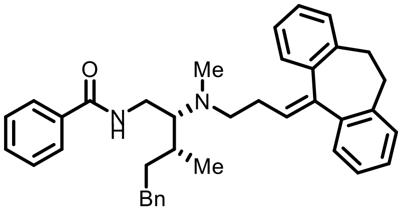

From (*E*)-*N*-(3-methyl-5-phenylpent-2-en-1-yl)benzamide (**1a**) (55.8 mg, 0.20 mmol, 1.0 equiv) and *O*-benzoyl-*N*-(3-(10,11-dihydro-5H-dibenzo[a,d][7]annulen-5-ylidene)propyl)-*N*-methylhydroxylamine (**2ac**) (115.0 mg, 0.30 mmol, 1.5 equiv), the title compound was prepared following the general procedure **A** using $NiBr_2$·DME (6.2 mg, 10 mol%), **(*S*,*S*)-L12** (15.4 mg, 15 mol%), LiOH (9.6 mg, 0.40 mmol, 2.0 equiv), KI (33.2 mg, 0.20 mmol, 1.0 equiv), $Me(EtO)_2SiH$ (80 µL, 0.50 mmol, 2.5 equiv), Anhydrous *tert*-butyl alcohol (1.0 mL, 0.20 M). The reaction mixture was stirred for 12 h at 30 °C. The crude material was purified by flash column chromatography (petroleum ether/EtOAc = 3:1) to provide the title compound as a yellow oil in 52% yield (56.4 mg).

^{1}H NMR (400 MHz, $CDCl_3$) δ 7.62 (s, 2H), 7.43–7.39 (m, 1H), 7.31–7.23 (m, 5H), 7.20–7.08 (m, 9H), 7.01–6.97 (m, 1H), 6.92 (s, 1H), 5.96–5.84 (m, 1H), 3.73 (s, 1H), 3.41–3.28 (m, 1H), 3.16 (q, *J* = 16.5 Hz, 2H), 2.93 (s, 1H), 2.77–2.67 (m, 2H), 2.68–2.49 (m, 4H), 2.39–2.27 (m, 2H), 2.26–2.13 (m, 3H), 1.87–1.79 (m, 1H), 1.70 (s, 1H), 1.55–1.44 (m, 1H), 1.00 (d, *J* = 6.8 Hz, 3H). **^{13}C NMR** (101 MHz, $CDCl_3$) δ 167.1, 144.1, 142.3, 140.1, 139.3, 137.0, 134.6, 131.2, 130.3, 128.7, 128.5, 128.4, 128.1, 127.6, 127.2, 126.8, 126.1, 125.9, 125.8, 65.3, 53.9, 37.6, 36.3, 33.8, 33.4, 31.9, 30.8, 29.8, 28.9, 16.6. **HRMS** (ESI) m/z calculated for $C_{38}H_{42}N_2O$ $[M+H]^+$ 543.3370 found: 543.3370. **Optical rotation**: $[\alpha]^{20}_D$ = -82.6 (c = 1.0 g/L, $CHCl_3$). The absolute configuration was assigned by analogy to that of **4b**. **HPLC condition**: Chiral column OD-H, n-hexane/i-PrOH = 95:5, flow rate = 1 mL/min, wavelength = 254 nm, tR = 35.61 min for major isomer, tR = 28.41 min for minor isomer.

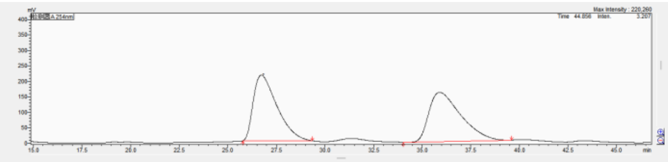

峰号	保留时间	面积	高度	标记	浓度	浓度单位	化合物ID号	化合物名	面积%
1	26.723	18036834	212390	M	49.932				49.932
2	35.899	18085852	158917	M	50.068				50.068
总计		36122686	371307		100.000				100.000

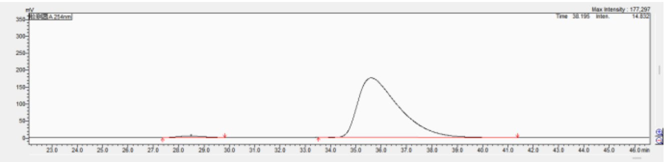

峰号	保留时间	面积	高度	标记	浓度	浓度单位	化合物ID号	化合物名	面积%
1	28.412	343192	4668	M	1.703				1.703
2	35.605	19807207	175803	M	98.297				98.297
总计		20150398	180471		100.000				100.000

8. Gram-Scale Reaction, Product Derivatizations and Synthetic Potentials

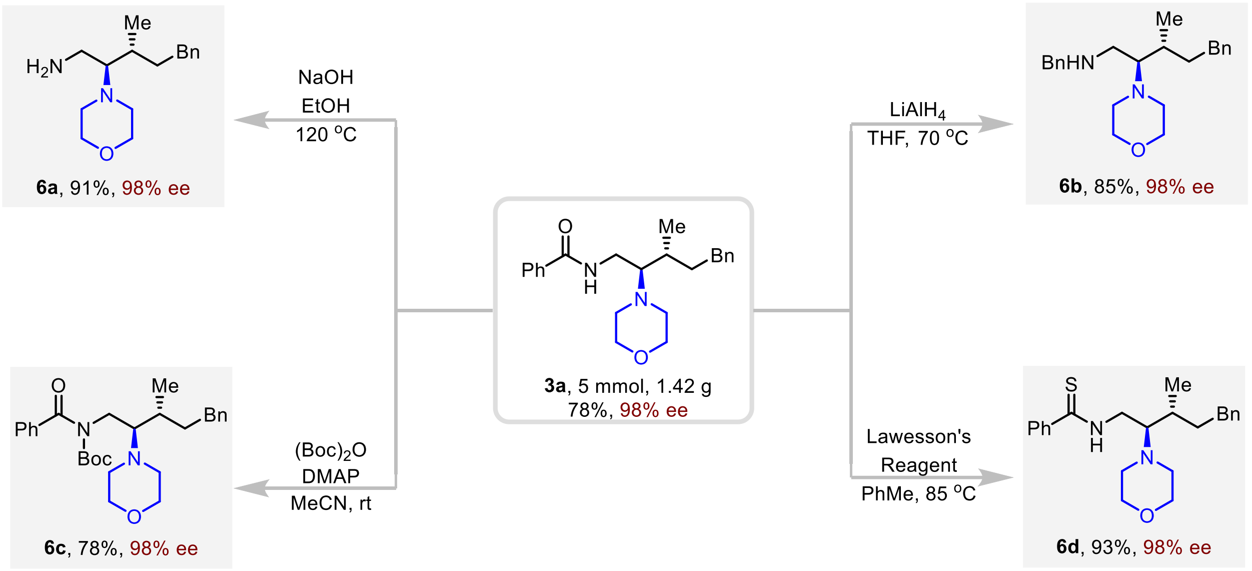

8.1 Gram-scale reaction and further derivatization

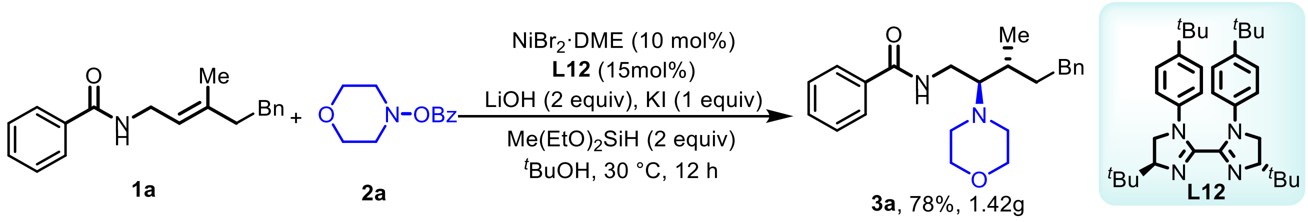

In an Ar-filled glove box, to an oven-dried 4 mL screw-cap vial equipped with a magnetic stir bar was added $NiBr_2$·DME (0.5 mmol, 10 mol%), ***(S,S)*-L12** (0.75 mmol, 15 mol%), LiOH (10.0 mmol, 2.0 equiv), KI (5.0 mmol, 1.0 equiv), Olefin feedstock (5.0 mmol, 1.0 equiv), hydroxylamine ester (7.5 mmol, 1.5 equiv) and Anhydrous *tert*-butyl alcohol (25 mL, 0.20 M). The mixture was stirred for 10 min at room temperature, at which time $Me(EtO)_2SiH$ (12.5 mmol, 2.5 equiv) were added to the resulting mixture . The reaction mixture was stirred for 12 h at 30 °C. The crude material was purified by flash column chromatography (petroleum ether/EtOAc = 3:1) to provide the title compound as a yellow oil in 78% yield (1.42 g).

(2*R*,3*R*)-3-methyl-2-morpholino-5-phenylpentan-1-amine (6a)

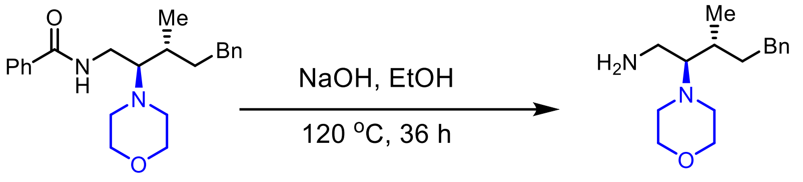

3a **6a**, 91%, 98% ee

In an oven-dried 15 mL sealed tube equipped with a Teflon-coated stir bar, **3a** (73.2 mg, 0.20 mmol), NaOH (120 mg, 3.0 mmol, 15 equiv), and anhydrous EtOH (1.5 mL) were combined. The vessel was sealed under N_2, immersed in a preheated oil bath at 120 °C, and stirred vigorously for 36 h. After cooling to room temperature, the reaction mixture was diluted with EtOAc (5 mL) and washed with brine (3 × 5 mL). The combined organic extracts were dried over anhydrous Na_2SO_4, filtered through a short pad of Celite®, and concentrated under reduced pressure to afford the product **6a** as a pale yellow oil (47.7 mg, 88% yield).[6]

^{1}H NMR (400 MHz, Chloroform-*d*) δ 7.34–7.28 (m, 2H), 7.25–7.18 (m, 3H), 3.74–3.61 (m, 4H), 2.80–2.67 (m, 5H), 2.65–2.50 (m, 3H), 2.37–2.28 (m, 1H), 1.89–1.72 (m, 5H), 1.61–1.45 (m, 1H), 0.97 (d, *J* = 6.7 Hz, 3H). **^{13}C NMR** (101 MHz, Chloroform-*d*) δ 142.6, 128.4, 128.4, 125.8, 70.9, 67.8, 49.8, 38.9, 37.5, 33.4, 31.1, 16.8. **HRMS (ESI)** m/z calculated for $C_{16}H_{26}N_2O$ $[M+H]^+$ 263.2118, found 263.2118. **Optical rotation**: $[\alpha]^{20}_D$ = 4.67 (c = 1.0 g/L, $CHCl_3$). **HPLC condition**: HPLC data were tested by converting **6a** to **3a**. Chiral column AD-H, *n*-hexane/*i*-PrOH = 95:5, flow rate = 1.0 mL/min, wavelength =254 nm, tR = 41.1 min for major isomer, tR = 36.7 min for minor isomer. The absolute configuration was assigned by analog to that of **4b**.

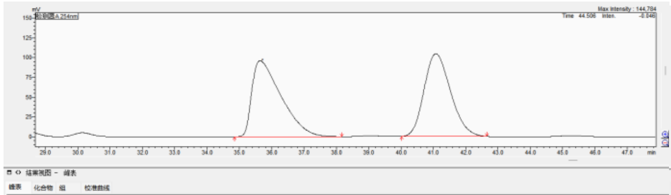

结果视图 - 峰表

峰号	保留时间	面积	高度	标记	浓度	浓度单位	化合物ID号	化合物名	面积%
1	35.640	6045308	96260	M	50.358				50.358
2	41.076	5959338	104530	M	49.642				49.642
总计		12004646	200790		100.000				100.000

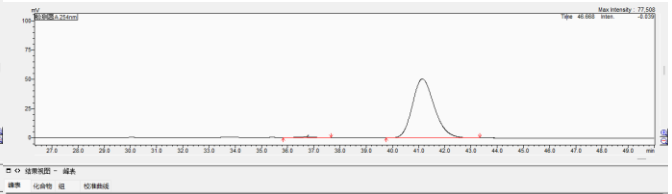

结果视图 - 峰表

峰号	保留时间	面积	高度	标记	浓度	浓度单位	化合物ID号	化合物名	面积%
1	36.689	22517	600	M	0.764				0.764
2	41.145	2922859	50279		99.236				99.236
总计		2945376	50878		100.000				100.000

(2*R*,3*R*)-*N*-benzyl-3-methyl-2-morpholino-5-phenylpentan-1-amine (6b)

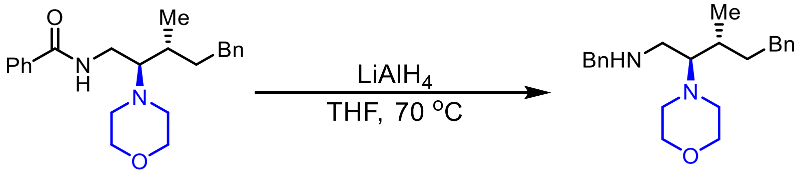

3a

6b, 85%, 98% ee

A solution of **3a** (73.2 mg, 0.2 mmol) in anhydrous THF (4.0 mL) was added to a flame-dried 20 mL round-bottom flask under N_2. The mixture was cooled to 0 °C, and $LiAlH_4$ (1.0 M in THF, 1.0 mL, 1.0 mmol, 5 equiv) was added dropwise via syringe over 5 min. The reaction was warmed to room temperature over 15 min, then heated at 70 °C with vigorous stirring for 12 h. After cooling to 0 °C, the reaction was carefully quenched by dropwise addition of MeOH (1.0 mL). Volatiles were removed *in vacuo*, and the residue was treated with 2 N HCl (4.0 mL) and stirred at 75 °C for 1 h. The mixture was cooled to room temperature, basified to pH 9-10 with saturated K_2CO_3 solution (10 mL), and extracted with EtOAc (3 × 25 mL). The combined organic layers were dried over anhydrous $MgSO_4$, filtered through Celite®, and concentrated under reduced pressure. Purification by flash chromatography (PE/EtOAc= 3/1) afforded **6b** (47.0 mg, 85% yield) as a colorless oil.[7]

^{1}H NMR (600 MHz, Chloroform-*d*) δ 7.31–7.24 (m, 4H), 7.22–7.17 (m, 3H), 7.12–7.05 (m, 3H), 4.14–3.96 (m, 1H), 3.90 (d, *J* = 13.4 Hz, 1H), 3.72 (d, *J* = 13.3 Hz, 1H), 3.61–3.55 (m, 2H), 3.54–3.46 (m, 2H), 2.61–2.54 (m, 3H), 2.53–2.44 (m, 4H), 2.43–2.35 (m, 2H), 1.75–1.69 (m, 1H), 1.68–1.61 (m, 1H), 1.45–1.38 (m, 1H), 0.81 (d, *J* = 6.8 Hz, 3H). **^{13}C NMR** (151 MHz, Chloroform-*d*) δ 142.3, 138.5, 128.6, 128.5, 128.4, 128.4, 127.5, 125.8, 67.7, 67.0, 53.3, 49.5, 45.2, 37.4, 33.3, 30.9, 16.7. **HRMS (ESI)** m/z calculated for $C_{23}H_{32}N_2O$ $[M+H]^+$ 353.2588, found 353.2589. **Optical rotation**: $[\alpha]^{20}_D$ = 21.3 (c = 1.0 g/L, $CHCl_3$). **HPLC condition**: Testing HPLC data after protecting **6b** with Boc (t-Butyloxy carbonyl). Chiral column AD-H, *n*-hexane/*i*-PrOH = 95:5, flow rate = 1.0 mL/min, wavelength =254 nm, tR = 6.3 min for major isomer, tR = 5.5 min for minor isomer. The absolute configuration was assigned by analog to that of **4b**.

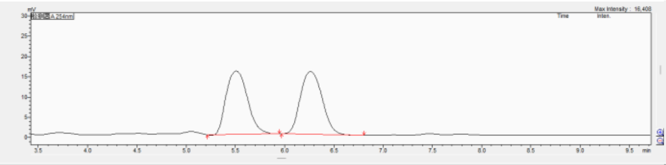

峰号	保留时间	面积	高度	标记	浓度	浓度单位	化合物ID号	化合物名	面积%
1	5.508	235438	15619	M	49.846				49.846
2	6.263	236894	15441	M	50.154				50.154
总计		472332	31060		100.000				100.000

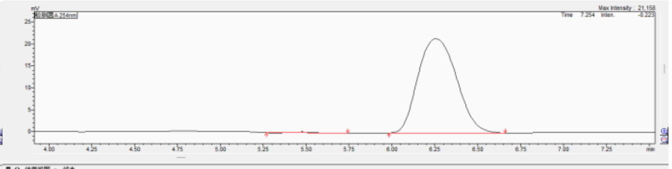

峰号	保留时间	面积	高度	标记	浓度	浓度单位	化合物ID号	化合物名	面积%
1	5.465	857	69	M	0.254				0.254
2	6.256	336566	21479	M	99.746				99.746
总计		337423	21548		100.000				100.000

tert-butyl benzoyl((2_R_,3_R_)-3-methyl-2-morpholino-5-phenylpentyl)carbamate (6c)

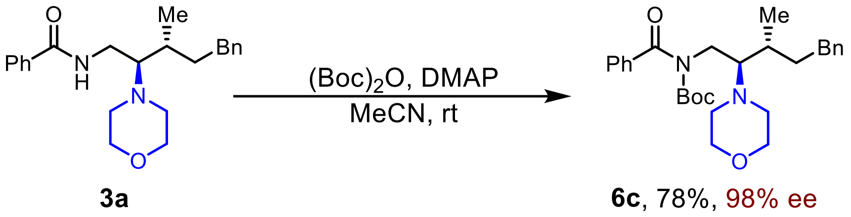

3a (73.2 mg, 0.2 mmol) and DMAP (2.4 mg, 0.02 mmol) in MeCN (2 mL) was added dropwise to a solution of di-*tert*-butyl dicarbonate (Boc_2O, 91.9 µL, 0.4 mmol) in MeCN at 0 °C. The reaction mixture was warmed to room temperature and stirred overnight. The reaction was then quenched by the addition of water (5 mL). The layers were separated, and the aqueous layer was extracted with ethyl acetate (3 × 5 mL). The combined organic extracts were dried over anhydrous Na_2SO_4. Concentration in vacuo followed by purification by flash column chromatography on silica gel (PE/EtOAc= 20/1) afforded the corresponding product **6c** as a colorless liquid (72.7 mg, 78% yield).[8]

^{1}H NMR (400 MHz, Chloroform-*d*) δ 7.64–7.60 (m, 2H), 7.53–7.49 (m, 1H), 7.45–7.39 (m, 2H), 7.34–7.29 (m, 2H), 7.27–7.20 (m, 3H), 4.25–4.12 (m, 1H), 3.70–3.59 (m, 1H), 3.49–3.34 (m, 4H), 2.91–2.81 (m, 3H), 2.77–2.67 (m, 2H), 2.49–2.39 (m, 2H), 1.99–1.91 (m, 1H), 1.89–1.81 (m, 1H), 1.71–1.62 (m, 1H), 1.14 (s, 9H), 1.06 (d, J = 6.8 Hz, 3H). **^{13}C NMR** (101 MHz, Chloroform-*d*) δ 173.5, 153.7, 142.4, 138.2, 131.2, 128.5, 128.4, 128.0, 125.8, 82.5, 67.5, 67.3, 49.6, 43.0, 38.2, 33.6, 30.0, 27.5, 16.8. **HRMS (ESI)** m/z calculated for $C_{28}H_{38}N_2O_4$ $[M+H]^+$ 467.2905, found 467.2905. **Optical rotation**: $[\alpha]^{20}_D$ = -54.5 (c = 1.0 g/L, $CHCl_3$). **HPLC condition**: Chiral column IC, *n*-hexane/*i*-PrOH = 95:5, flow rate = 1.0 mL/min, wavelength =254 nm, tR = 11.8 min for major isomer, tR = 12.9 min for minor isomer. The absolute configuration was assigned by analog to that of **4b**.

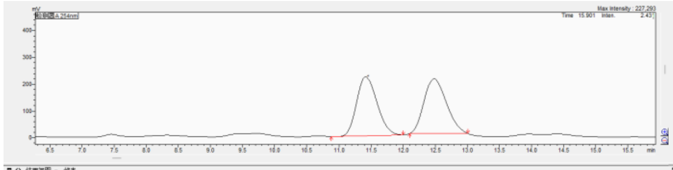

峰号	保留时间	面积	高度	标记	浓度	浓度单位	化合物ID号	化合物名	面积%
1	11.419	4832255	219605	M	49.236				49.236
2	12.481	4982200	204112	M	50.764				50.764
总计		9814455	423717		100.000				100.000

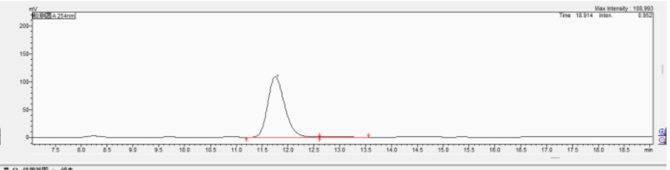

峰号	保留时间	面积	高度	标记	浓度	浓度单位	化合物ID号	化合物名	面积%
1	11.754	2458090	108291	M	99.214				99.214
2	12.905	19478	628	V M	0.786				0.786
总计		2477569	108919		100.000				100.000

N-((2R,3R)-3-methyl-2-morpholino-5-phenylpentyl)benzothioamide (6d)

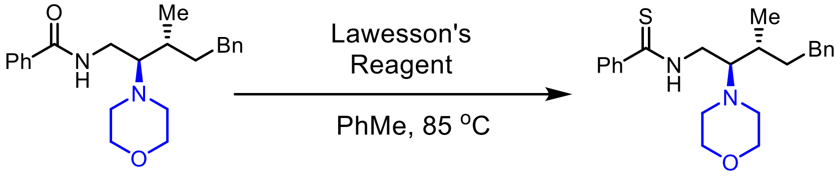

3a **6d**, 93%, 98% ee

3a (73.2 mg, 0.2 mmol) was dissolved in toluene (2 mL) and treated with Lawesson's reagent (2,4-bis(4-methoxyphenyl)1,3,2,4-dithiadiphosphetane-2,4-dithione] (161.8 mg, 0.4 mmol, 2.0 eq.) undermagnetic stirring.The resulting solution was heated to 85 °C and stirred at that temperature for 2 hours. GC-MS indicated clean conversion to the product. The reaction was cooled, diluted with EtOAc (25 mL) and washed with saturated NH_4Cl aqueous solution and brine (25 mL). Theorganic layer was driedover anhydrous $MgSO_4$, filtered and concentrated in vacuo. The product was then chromatized with a neutral alumina column (PE/EtOAc= 20/1) and afforded the corresponding product **6d** as a colorless liquid (71.1 mg, 93% yield).[9]

^{1}H NMR (400 MHz, Chloroform-d) δ 8.61 (s, 1H), 7.88–7.79 (m, 2H), 7.56–7.50 (m, 1H), 7.49–7.43 (m, 2H), 7.38–7.32 (m, 2H), 7.28–7.21 (m, 3H), 4.16–3.97 (m, 1H), 3.80–3.66 (m, 4H), 3.65–3.56 (m, 1H), 2.87–2.72 (m, 4H), 2.71–2.57 (m, 3H), 2.12–1.98 (m, 1H), 1.91–1.78 (m, 1H), 1.75–1.61 (m, 1H), 1.10 (d, J = 6.9 Hz, 3H). **^{13}C NMR** (101 MHz, Chloroform-d) δ 197.4, 141.9, 141.5, 131.1, 128.6, 128.5, 128.4, 126.6, 126.0, 67.7, 66.1, 49.5, 43.6, 37.6, 33.4, 31.0, 16.4. **HRMS (ESI)** m/z calculated for $C_{23}H_{30}N_2OS$ $[M+H]^+$ 383.2152, found 383.2152. **Optical rotation**: $[\alpha]^{20}_D$ = 63.4 (c = 1.0 g/L, $CHCl_3$). **HPLC condition**: Chiral column AD-H, n-hexane/i-PrOH = 95:5, flow rate = 1.0 mL/min, wavelength =254 nm, tR = 31.2 min for major isomer, tR = 24.2 min for minor isomer. The absolute configuration was assigned by analog to that of **4b**.

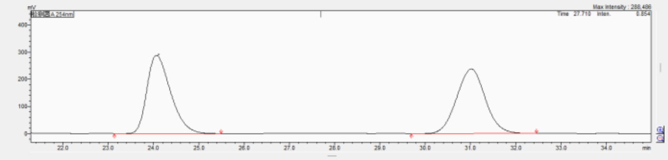

峰号	保留时间	面积	高度	标记	浓度	浓度单位	化合物ID号	化合物名	面积%
1	24.065	10484625	288176	M	50.167				50.167
2	31.018	10414763	237626	M	49.833				49.833
总计		20899387	525803		100.000				100.000

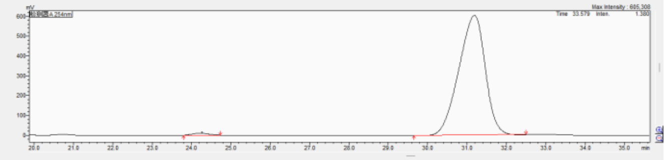

峰号	保留时间	面积	高度	标记	浓度	浓度单位	化合物ID号	化合物名	面积%
1	24.213	287512	9823	M	1.009				1.009
2	31.186	28193810	604175	M	98.991				98.991
总计		28481322	613999		100.000				100.000

8.2 Construction of drug analogues featuring N-(2-ethylamino)amide motifs

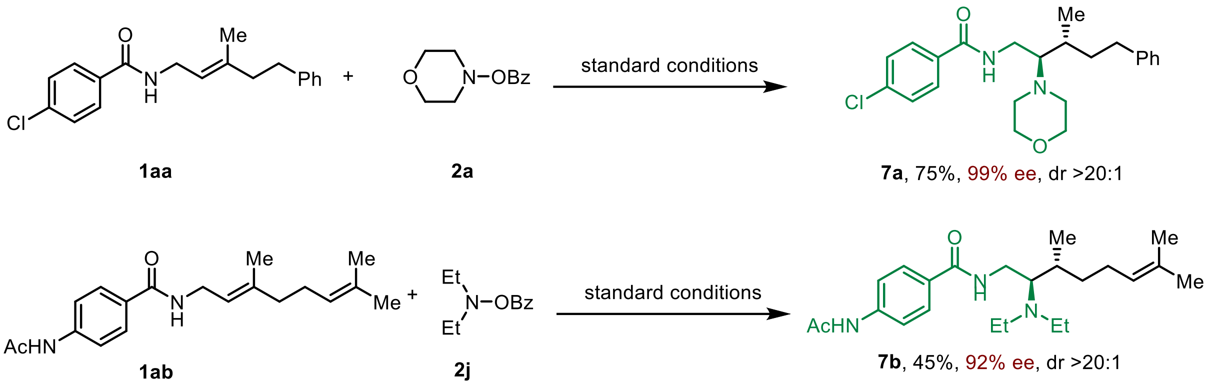

4-Chloro-*N*-((2*R*,3*R*)-3-methyl-2-morpholino-5-phenylpentyl)benzamide (7a)

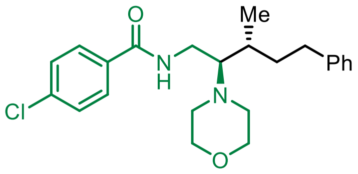

From (*E*)-4-chloro-*N*-(3-methyl-5-phenylpent-2-en-1-yl)benzamide (**1aa**) (62.6 mg, 0.20 mmol, 1.0 equiv) and morpholino benzoate (**2a**) (62.1 mg, 0.30 mmol, 1.5 equiv), the title compound was prepared following the general procedure **A** using $NiBr_2$·DME (6.2 mg, 10 mol%), **(*S*,*S*)-L12** (15.4 mg, 15 mol%), LiOH (9.6 mg, 0.40 mmol, 2.0 equiv), KI (33.2 mg, 0.20 mmol, 1.0 equiv), $Me(EtO)_2SiH$ (80 μL, 0.50 mmol, 2.5 equiv), Anhydrous *tert*-butyl alcohol (1.0 mL, 0.20 M). The reaction mixture was stirred for 12 h at 30 °C. The crude material was purified by flash column chromatography (petroleum ether/EtOAc = 3:1) to provide the title compound as a yellow oil in 75% yield (60.0 mg).

^{1}H NMR (400 MHz, $CDCl_3$) δ 7.69 (d, *J* = 8.2 Hz, 2H), 7.44–7.38 (m, 2H), 7.30–7.25 (m, 2H), 7.21–7.15 (m, 3H), 6.92 (s, 1H), 3.75–3.61 (m, 5H), 3.32–3.21 (m, 1H), 2.78–2.65 (m, 3H), 2.64–2.50 (m, 4H), 1.97–1.85 (m, 1H), 1.84–1.71 (m, 1H), 1.65–1.53 (m, 1H), 1.01 (d, *J* = 6.8 Hz, 3H). **^{13}C NMR** (101 MHz, $CDCl_3$) δ 166.0, 142.0, 137.7, 133.1, 128.9, 128.5, 128.4, 128.3, 125.9, 67.9, 67.0, 49.6, 37.4, 36.9, 33.4, 31.0, 16.5. **HRMS** (ESI) m/z calculated for $C_{23}H_{29}ClN_2O_2$ $[M+H]^+$ 401.1991 found: 401.1991. **Optical rotation**: $[\alpha]^{20}_D$ = -97.9 (c = 1.0 g/L, $CHCl_3$). The absolute configuration was assigned by analogy to that of **4b**. **HPLC condition**: Chiral column AD-H, n-hexane/i-PrOH = 95:5, flow rate = 1 mL/min, wavelength = 254 nm, tR = 37.5 min for major isomer, tR = 47.8 min for minor isomer.

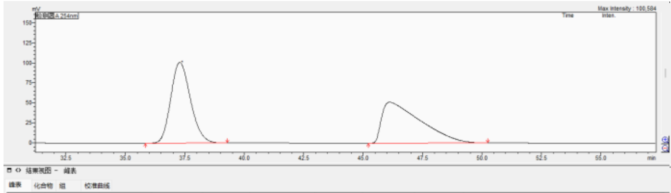

峰号	保留时间	面积	高度	标记	浓度	浓度单位	化合物ID号	化合物名	面积%
1	37.284	5836929	100829		50.174				50.174
2	46.117	5796348	51078		49.826				49.826
总计		11633277	151907		100.000				100.000

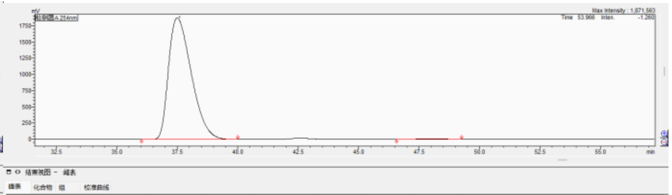

峰号	保留时间	面积	高度	标记	浓度	浓度单位	化合物ID号	化合物名	面积%
1	37.501	122958960	1872272	M	99.405				99.405
2	47.835	736073	11989	M	0.595				0.595
总计		123695032	1884260		100.000				100.000

4-Acetamido-*N*-((2*R*,3*R*)-2-(diethylamino)-3,7-dimethyloct-6-en-1-yl)benzamide (7b)

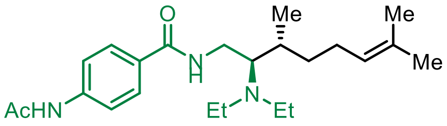

From (*E*)-4-acetamido-*N*-(3,7-dimethylocta-2,6-dien-1-yl)benzamide (**1ab**) (62.8 mg, 0.20 mmol, 1.0 equiv) and *O*-benzoyl-*N*,*N*-diethylhydroxylamine (**2j**) (57.9 mg, 0.30 mmol, 1.5 equiv), the title compound was prepared following the general procedure **A** using $NiBr_2$·DME (6.2 mg, 10 mol%), **(*S*,*S*)-L12** (15.4 mg, 15 mol%), LiOH (9.6 mg, 0.40 mmol, 2.0 equiv), KI (33.2 mg, 0.20 mmol, 1.0 equiv), $Me(EtO)_2SiH$ (80 µL, 0.50 mmol, 2.5 equiv), Anhydrous *tert*-butyl alcohol (1.0 mL, 0.20 M). The reaction mixture was stirred for 12 h at 30 °C. The crude material was purified by flash column chromatography (petroleum ether/EtOAc = 1:1) to provide the title compound as a yellow oil in 45% yield (34.9 mg).

^{1}H NMR (600 MHz, $CDCl_3$) δ 8.82–8.68 (m, 1H), 7.70 (d, *J* = 8.2 Hz, 2H), 7.64 (d, *J* = 8.4 Hz, 2H), 7.41–7.28 (m, 1H), 5.09–5.03 (m, 1H), 3.75–3.68 (m, 1H), 3.04–2.93 (m, 1H), 2.82–2.73 (m, 2H), 2.70–2.62 (m, 1H), 2.61–2.53 (m, 2H), 2.17 (s, 3H), 2.06–1.97 (m, 1H), 1.96–1.87 (m, 1H), 1.83–1.74 (m, 1H), 1.66 (s, 3H), 1.58 (s, 3H), 1.53–1.43 (m, 1H), 1.21–1.13 (m, 1H), 1.11–1.01 (m, 6H), 0.90 (d, *J* = 6.7 Hz, 3H). **^{13}C NMR** (151 MHz, $CDCl_3$) δ 169.2, 166.8, 141.4, 131.8, 127.7, 124.8, 124.2, 119.3, 63.7, 44.4, 38.3, 35.5, 32.6, 31.4, 29.7, 25.7, 25.5, 24.6, 17.7, 16.5. **HRMS** (ESI) m/z calculated for $C_{23}H_{37}N_3O_2$ $[M+H]^+$ 388.2959 found: 388.2959. **Optical rotation**: $[\alpha]^{20}_D$ = -102 (c = 1.0 g/L, $CHCl_3$). The absolute configuration was assigned by analogy to that of **4b**. **HPLC condition**: Chiral column AD-H, n-hexane/i-PrOH = 95:5, flow rate = 1 mL/min, wavelength = 254 nm, tR = 23.1 min for major isomer, tR = 19.4 min for minor isomer.

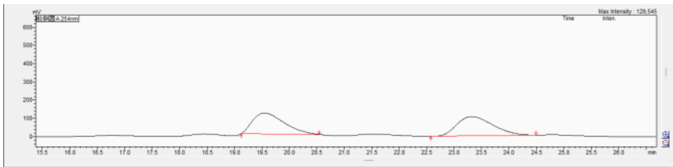

结果视图 - 峰表

峰号	保留时间	面积	高度	标记	浓度	浓度单位	化合物ID号	化合物名	面积%
1	19.546	4628863	115231	M	49.017				49.017
2	23.322	4814491	105655	M	50.983				50.983
总计		9443354	220886		100.000				100.000

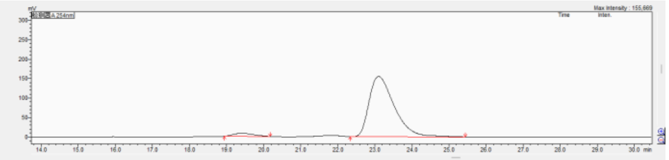

结果视图 - 峰表

峰号	保留时间	面积	高度	标记	浓度	浓度单位	化合物ID号	化合物名	面积%
1	19.407	293968	7686	M	3.869				3.869
2	23.101	7303455	155181	M	96.131				96.131
总计		7597422	162867		100.000				100.000

9. Mechanism experiment

9.1 Control experiment with terminal or 1,1-disubstituted alkenes

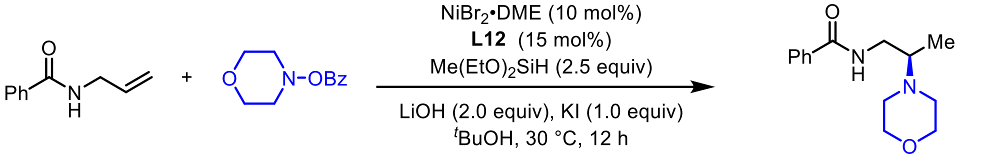

8a **2a** **9a**, 89%, 84% ee, rr = 89:11

(*R*)-*N*-(2-morpholinopropyl)benzamide (9a)

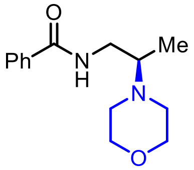

From *N*-allylbenzamide (**8a**) (32.2 mg, 0.20 mmol, 1.0 equiv) and morpholino benzoate (**2a**) (62.1 mg, 0.30 mmol, 1.5 equiv), the title compound was prepared following the general procedure **A** using $NiBr_2$·DME (6.2 mg, 10 mol%), **(*S*,*S*)-L12** (15.4 mg, 15 mol%), LiOH (9.6 mg, 0.40 mmol, 2.0 equiv), KI (33.2 mg, 0.20 mmol, 1.0 equiv), $Me(EtO)_2SiH$ (80 µL, 0.50 mmol, 2.5 equiv), Anhydrous *tert*-butyl alcohol (1.0 mL, 0.20 M). The reaction mixture was stirred for 12 h at 30 °C. The crude material was purified by flash column chromatography (petroleum ether/EtOAc = 2:1) to provide the title compound as a white solid in 89% yield (44.2 mg).

^{1}H NMR (400 MHz, $CDCl_3$) δ 7.81–7.71 (m, 2H), 7.51–7.47 (m, 1H), 7.46–7.39 (m, 2H), 7.05 (s, 1H), 3.77–3.66 (m, 4H), 3.66–3.57 (m, 1H), 3.20–3.09 (m, 1H), 2.88–2.74 (m, 1H), 2.69–2.59 (m, 2H), 2.50–2.40 (m, 2H), 1.04 (d, J = 6.6 Hz, 3H). **^{13}C NMR** (101 MHz, $CDCl_3$) δ 167.2, 134.7, 131.4, 128.6, 126.9, 67.5, 58.1, 48.3, 41.6, 11.4. **Optical rotation**: $[\alpha]^{25}_D$ = +12.3 (c = 1.0 g/L, $CHCl_3$). **HPLC condition**: Chiral column OD-H, *n*-hexane/*i*-PrOH = 90:10, flow rate = 1.0 mL/min, wavelength =254 nm, tR = 12.1 min for major isomer, tR = 14.2 min for minor isomer. The absolute configuration was assigned by analog to that of **4b**.

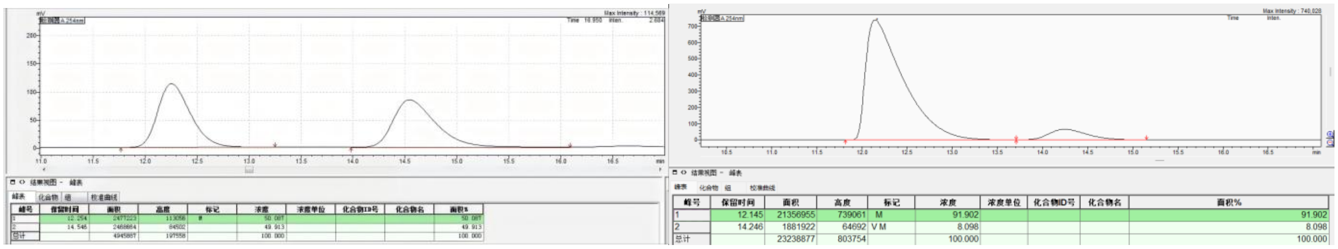

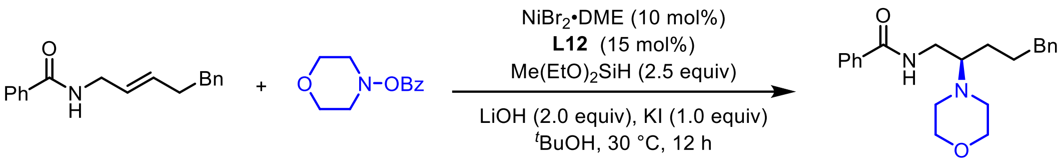

8b **2a** **9b**, 93%, 84% ee, rr > 99:1

(*R*)-*N*-(2-morpholino-5-phenylpentyl)benzamide (9b)

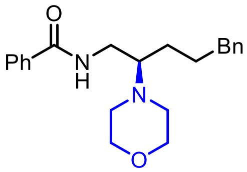

From (*E*)-*N*-(5-phenylpent-2-en-1-yl)benzamide (**8b**) (53.0 mg, 0.20 mmol, 1.0 equiv) and morpholino benzoate (**2a**) (62.1 mg, 0.30 mmol, 1.5 equiv), the title compound was prepared following the general procedure **A** using $NiBr_2$·DME (6.2 mg, 10 mol%), **(*S*,*S*)-L12** (15.4 mg, 15 mol%), LiOH (9.6 mg, 0.40 mmol, 2.0 equiv), KI (33.2 mg, 0.20 mmol, 1.0 equiv), $Me(EtO)_2SiH$ (80 μL, 0.50 mmol, 2.5 equiv), Anhydrous *tert*-butyl alcohol (1.0 mL, 0.20 M). The reaction mixture was stirred for 12 h at 30 °C. The crude material was purified by flash column chromatography (petroleum ether/EtOAc = 3:1) to provide the title compound as a white solid in 93% yield (65.5 mg).

^{1}H NMR (400 MHz, $CDCl_3$) δ 7.83–7.79 (m, 2H), 7.57–7.52 (m, 1H), 7.51–7.44 (m, 2H), 7.36–7.27 (m, 2H), 7.25–7.18 (m, 3H), 7.12 (d, *J* = 6.4 Hz, 1H), 3.85–3.62 (m, 5H), 3.20–3.01 (m, 1H), 2.76–2.60 (m, 5H), 2.56–2.46 (m, 2H), 1.83–1.72 (m, 2H), 1.70–1.60 (m, 1H), 1.37–1.30 (m, 1H). **^{13}C NMR** (101 MHz, $CDCl_3$) δ 167.1, 141.8, 134.7, 131.4, 128.7, 128.4, 128.4, 126.9, 125.9, 67.7, 63.1, 48.5, 39.5, 36.0, 28.8, 26.1. **HRMS** (ESI) m/z calculated for $C_{22}H_{28}N_2O_2$ $[M+Na]^+$ 375.2043 found: 375.2043. **Optical rotation**: $[\alpha]^{25}_D$ = -25.1 (c = 1.0 g/L, $CHCl_3$). **HPLC condition**: Chiral column AD-H, *n*-hexane/*i*-PrOH = 90:10, flow rate = 1.0 mL/min, wavelength =254 nm, tR = 18.9 min for major isomer, tR = 17.3 min for minor isomer. The absolute configuration was assigned by analog to that of **4b**.

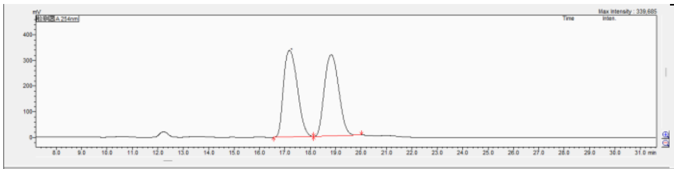

结果视图 - 峰表

峰号	保留时间	面积	高度	标记	浓度	浓度单位	化合物ID号	化合物名	面积%
1	17.183	12751054	337853	M	50.582				50.582
2	18.826	12457857	316779	M	49.418				49.418
总计		25208911	654632		100.000				100.000

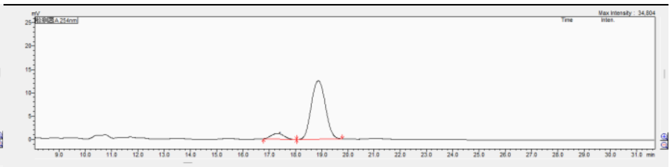

结果视图 - 峰表

峰号	保留时间	面积	高度	标记	浓度	浓度单位	化合物ID号	化合物名	面积%
1	17.321	40869	1190	M	8.019				8.019
2	18.876	468757	12547	M	91.981				91.981
总计		509626	13737		100.000				100.000

9.2 Deuterium labeling experiment

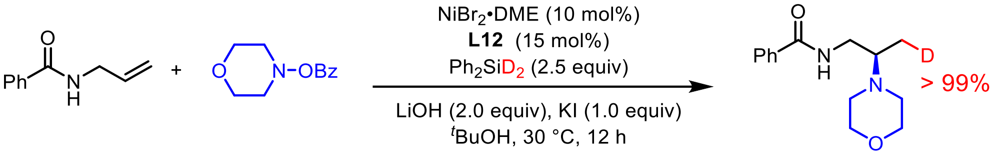

8a **2a** **9a-*d1***, 92%, rr = 92:8

(*R*)-*N*-(2-morpholinopropyl-3-d)benzamide (9a-*d1*)

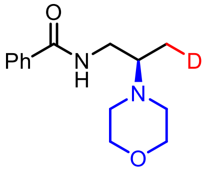

From *N*-allylbenzamide (**8a**) (32.2 mg, 0.20 mmol, 1.0 equiv) and morpholino benzoate (**2a**) (62.1 mg, 0.30 mmol, 1.5 equiv), the title compound was prepared following the general procedure **A** using $NiBr_2$·DME (6.2 mg, 10 mol%), **(*S*,*S*)-L12** (15.4 mg, 15 mol%), LiOH (9.6 mg, 0.40 mmol, 2.0 equiv), KI (33.2 mg, 0.20 mmol, 1.0 equiv), Ph_2SiD_2 (93.0 mg, 0.50 mmol, 2.5 equiv), Anhydrous *tert*-butyl alcohol (1.0 mL, 0.20 M). The reaction mixture was stirred for 12 h at 30 °C. The crude material was purified by flash column chromatography (petroleum ether/EtOAc = 1:1) to provide the title compound as a white solid in 92% yield (45.8 mg).

^{1}H NMR (400 MHz, $CDCl_3$) δ 7.80–7.74 (m, 2H), 7.52–7.45 (m, 1H), 7.43–7.37 (m, 2H), 7.27–7.21 (m, 1H), 3.84–3.68 (m, 4H), 3.67–3.56 (m, 1H), 3.32–3.20 (m, 1H), 2.95–2.80 (m, 1H), 2.76–2.64 (m, 2H), 2.58–2.42 (m, 2H), 1.07 (d, J = 6.2 Hz, 2H). **HRMS** (ESI) m/z calculated for $C_{14}H_{19}DN_2O_2$ $[M+H]^+$ 250.1661 found: 250.1661.

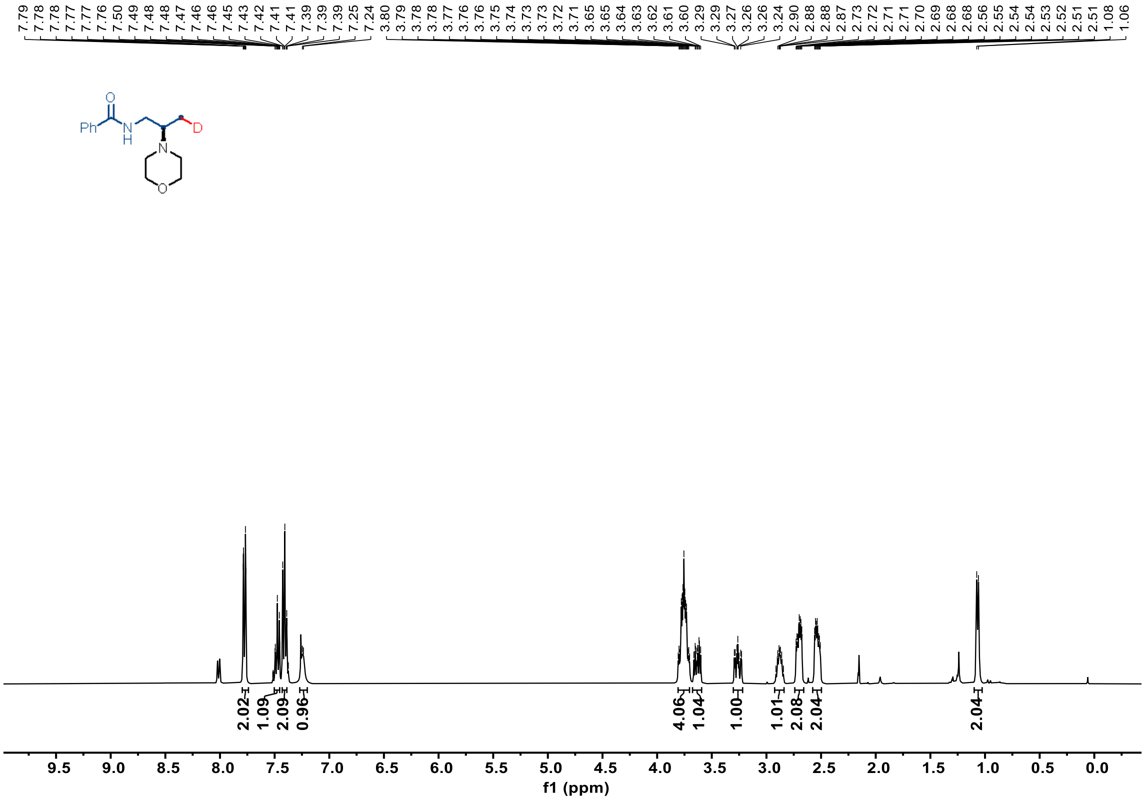

^{1}**H NMR** (400 MHz, CDCl$_3$) spectra of **9a-*d1***

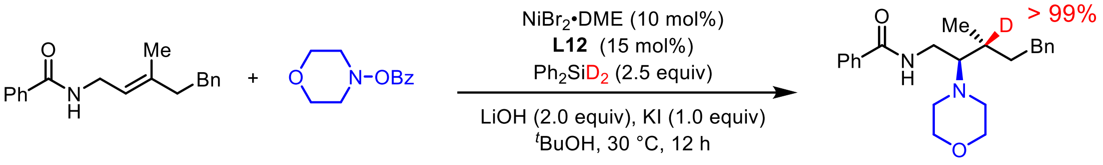

1a **2a** **3a-*d1***, 22%, 96% ee, dr > 20:1

N-((2*R*,3*R*)-3-methyl-2-morpholino-5-phenylpentyl-3-d)benzamide (3a-*d1*)

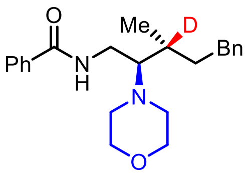

From (*E*)-*N*-(3-methyl-5-phenylpent-2-en-1-yl)benzamide (**1a**) (55.8 mg, 0.20 mmol, 1.0 equiv) and morpholino benzoate (**2a**) (62.1 mg, 0.30 mmol, 1.5 equiv), the title compound was prepared following the general procedure **A** using $NiBr_2$·DME (6.2 mg, 10 mol%), **(*S*,*S*)-L12** (15.4 mg, 15 mol%), LiOH (9.6 mg, 0.40 mmol, 2.0 equiv), KI (33.2 mg, 0.20 mmol, 1.0 equiv), Ph_2SiD_2 (93.0 mg, 0.50 mmol, 2.5 equiv), Anhydrous *tert*-butyl alcohol (1.0 mL, 0.20 M). The reaction mixture was stirred for 12 h at 30 °C. The crude material was purified by flash column chromatography (petroleum ether/EtOAc = 1:1) to provide the title compound as a white solid in 22% yield (16.2 mg).

^{1}H NMR (400 MHz, $CDCl_3$) δ 7.79–7.74 (m, 2H), 7.53–7.49 (m, 1H), 7.48–7.43 (m, 2H), 7.31–7.26 (m, 2H), 7.21–7.16 (m, 3H), 6.91 (s, 1H), 3.77–3.62 (m, 5H), 3.35–3.24 (m, 1H), 2.79–2.67 (m, 3H), 2.65–2.51 (m, 4H), 1.85–1.71 (m, 1H), 1.65–1.54 (m, 1H), 1.02 (s, 3H). **HPLC condition**: Chiral column AD-H, *n*-hexane/*i*-PrOH = 95:5, flow rate = 1.0 mL/min, wavelength =254 nm, tR = 41.7 min for major isomer, tR = 37.4 min for minor isomer. The absolute configuration was assigned by analog to that of **4b**.

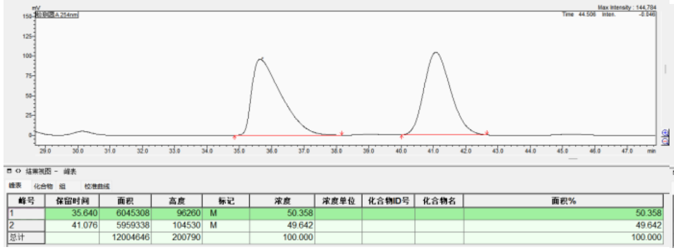

峰号	保留时间	面积	高度	标记	浓度	浓度单位	化合物ID号	化合物名	面积%
1	35.640	6045308	96260	M	50.358				50.358
2	41.076	5959338	104530	M	49.642				49.642
总计		12004646	200790		100.000				100.000

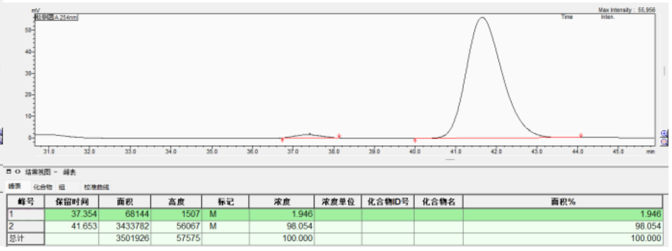

峰号	保留时间	面积	高度	标记	浓度	浓度单位	化合物ID号	化合物名	面积%
1	37.354	68144	1507	M	1.946				1.946
2	41.653	3433782	56067	M	98.054				98.054
总计		3501926	57575		100.000				100.000

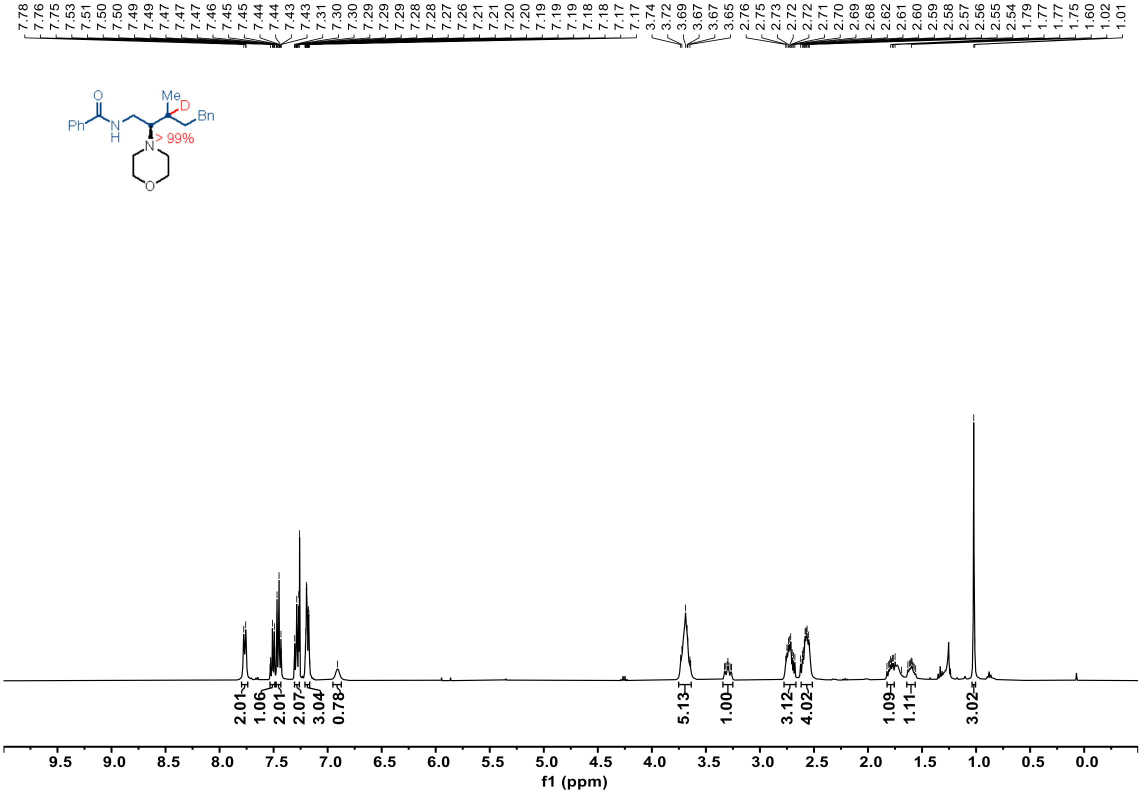

^{1}H NMR (400 MHz, $CDCl_3$) spectra of **3a-*d1***

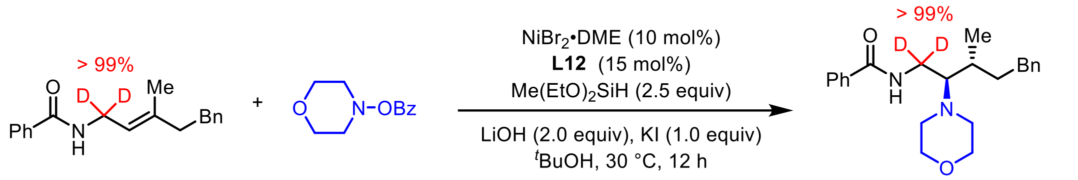

1a-*d2* **2a** **3a-*d2***, 83%, 96% ee, dr > 20:1

N-((2R,3R)-3-methyl-2-morpholino-5-phenylpentyl-1,1-d2)benzamide (3a-*d2*)

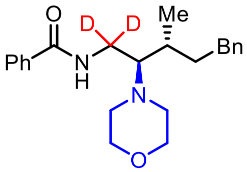

From (*E*)-*N*-(3-methyl-5-phenylpent-2-en-1-yl-1,1-d2)benzamide (**1a-*d2***) (56.2 mg, 0.20 mmol, 1.0 equiv) and morpholino benzoate (**2a**) (62.1 mg, 0.30 mmol, 1.5 equiv), the title compound was prepared following the general procedure **A** using $NiBr_2$·DME (6.2 mg, 10 mol%), **(*S*,*S*)-L12** (15.4 mg, 15 mol%), LiOH (9.6 mg, 0.40 mmol, 2.0 equiv), KI (33.2 mg, 0.20 mmol, 1.0 equiv), Me(EtO)$_2$SiH (80 μL, 0.50 mmol, 2.5 equiv), Anhydrous *tert*-butyl alcohol (1.0 mL, 0.20 M). The reaction mixture was stirred for 12 h at 30 °C. The crude material was purified by flash column chromatography (petroleum ether/EtOAc = 3:1) to provide the title compound as a white solid in 83% yield (61.3 mg).

^{1}H NMR (400 MHz, $CDCl_3$) δ 7.75–7.69 (m, 2H), 7.49–7.44 (m, 1H), 7.43–7.38 (m, 2H), 7.28–7.19 (m, 2H), 7.18–7.10 (m, 3H), 6.87 (s, 1H), 3.71–3.57 (m, 4H), 2.75–2.62 (m, 3H), 2.58–2.46 (m, 4H), 1.92–1.83 (m, 1H), 1.79–1.68 (m, 1H), 1.62–1.48 (m, 1H), 0.98 (d, J = 6.9 Hz, 3H). **HPLC condition**: Chiral column AD-H, *n*-hexane/*i*-PrOH = 95:5, flow rate = 1.0 mL/min, wavelength =254 nm, tR = 41.1 min for major isomer, tR = 37.0 min for minor isomer. The absolute configuration was assigned by analog to that of **4b**.

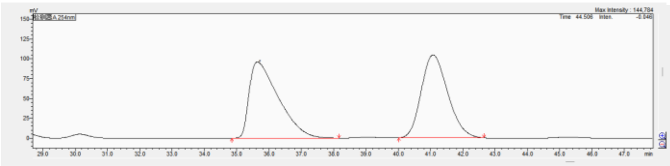

结果视图 - 峰表

峰号	保留时间	面积	高度	标记	浓度	浓度单位	化合物ID号	化合物名	面积%
1	35.640	6045308	96260	M	50.358				50.358
2	41.076	5959338	104530	M	49.642				49.642
总计		12004646	200790		100.000				100.000

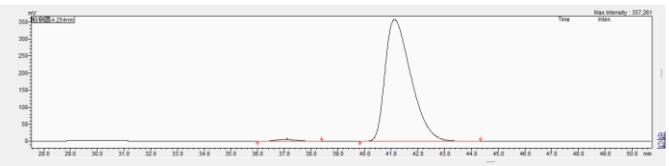

结果视图 - 峰表

峰号	保留时间	面积	高度	标记	浓度	浓度单位	化合物ID号	化合物名	面积%
1	37.018	266356	5063		1.103				1.103
2	41.120	23880740	356844		98.897				98.897
总计		24147096	361907		100.000				100.000

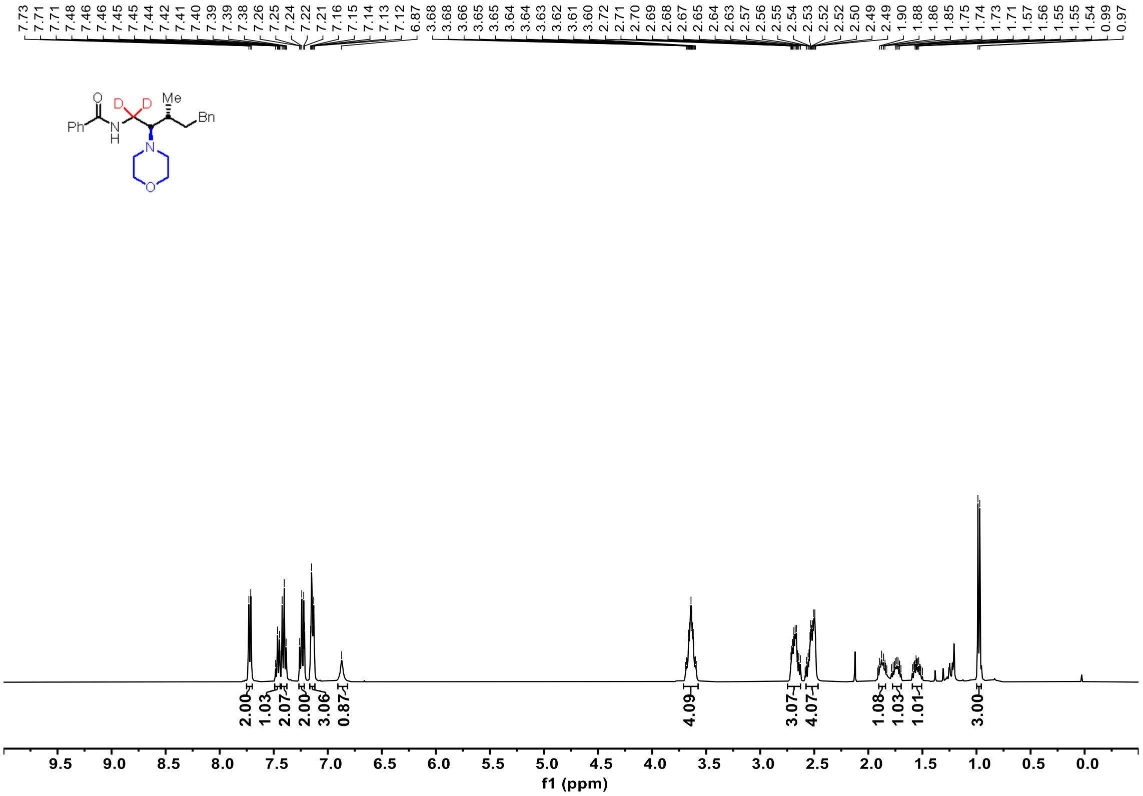

^{1}H NMR (400 MHz, $CDCl_3$) spectra of **3a-*d2***

9.3 Radical trapping experiments

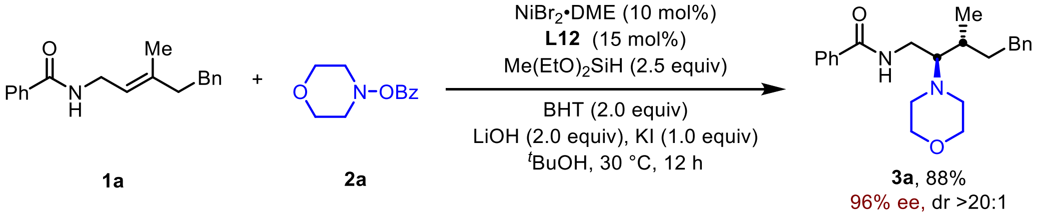

In an Ar-filled glovebox, a 4 mL screw-cap equipped with a magnetic stir bar was added $NiBr_2$•DME (6.2 mg, 0.02 mmol, 10 mol%), **(*S*,*S*)-L12** (15.4 mg, 15 mol%), (*E*)-*N*-(3-methyl-5-phenylpent-2-en-1-yl)benzamide (**1a**) (55.8 mg, 0.20 mmol, 1.0 equiv), morpholino benzoate (**2a**) (62.1 mg, 0.30 mmol, 1.5 equiv), Butylated hydroxytoluene (BHT) (88.1 mg, 0.40 mmol, 2.0 equiv), LiOH (9.6 mg, 0.40 mmol, 2.0 equiv), KI (33.2 mg, 0.20 mmol, 1.0 equiv), $Me(EtO)_2SiH$ (80 μL, 0.50 mmol, 2.5 equiv), Anhydrous *tert*-butyl alcohol (1.0 mL, 0.20 M). The reaction mixture was stirred for 12 h at 30 °C. The crude material was purified by flash column chromatography (petroleum ether/EtOAc = 3:1) to provide the title compound as a white solid in 88% yield (64.4 mg). **HPLC condition**: Chiral column AD-H, *n*-hexane/*i*-

PrOH = 95:5, flow rate = 1.0 mL/min, wavelength =254 nm, tR = 41.9 min for major isomer, tR = 37.0 min for minor isomer. The absolute configuration was assigned by analog to that of **4b**.

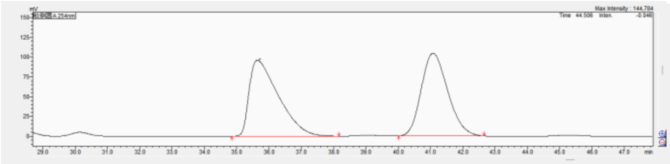

峰号	保留时间	面积	高度	标记	浓度	浓度单位	化合物ID号	化合物名	面积%
1	35.640	6045308	96260	M	50.358				50.358
2	41.076	5959338	104530	M	49.642				49.642
总计		12004646	200790		100.000				100.000

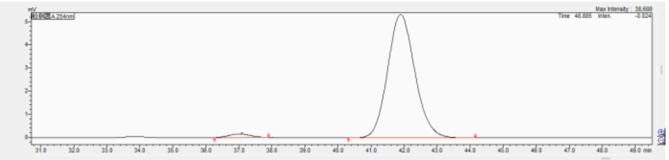

峰号	保留时间	面积	高度	标记	浓度	浓度单位	化合物ID号	化合物名	面积%
1	37.020	6938	153	M	2.215				2.215
2	41.895	306234	5339	M	97.785				97.785
总计		313172	5492		100.000				100.000

9.4 Non-linear effect study

We investigated the dependence of product enantiomeric excess (e.e.) on catalyst e.e. by preparing catalyst systems with nominal e.e. values of 100%, 80%, 60%, 40%, 20%, and 0% through mixing enantiomerically pure ligand **L12** with racemic **L12**, with the actual catalyst e.e. for each system confirmed by chiral HPLC analysis. In an argon-filled glovebox, the following reagents were added sequentially to each of six dried 4-mL vials equipped with magnetic stir bars: $NiBr_2$•DME (6.2 mg, 0.02 mmol, 10 mol%), **L12** (15.4 mg, 15 mol%), (*E*)-*N*-(3-methyl-5-phenylpent-2-en-1-yl)benzamide (**1a**) (55.8 mg, 0.20 mmol, 1.0 equiv), morpholino benzoate (**2a**) (62.1 mg, 0.30 mmol, 1.5 equiv), LiOH (9.6 mg, 0.40 mmol, 2.0 equiv), KI (33.2 mg, 0.20 mmol, 1.0 equiv), $Me(EtO)_2SiH$ (80 μL, 0.50 mmol, 2.5 equiv), Anhydrous *tert*-butyl alcohol (1.0 mL, 0.20 M). The reaction mixture was stirred for 12 h at 30 °C. Upon completion, the vials were removed from the glovebox; each reaction mixture was filtered through a short pad of silica gel, and the filter cake was washed thoroughly with EtOAc. The resulted filtrate was then concentrated under reduced pressure to give the crude product. The e.e. of **3a** was determined by HPLC after purification by preparative TLC.

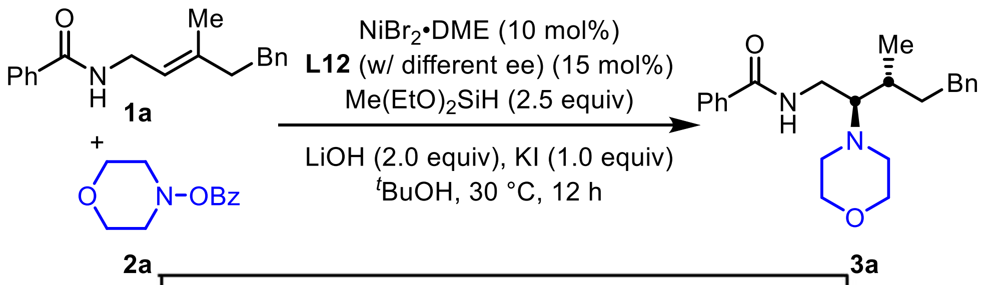

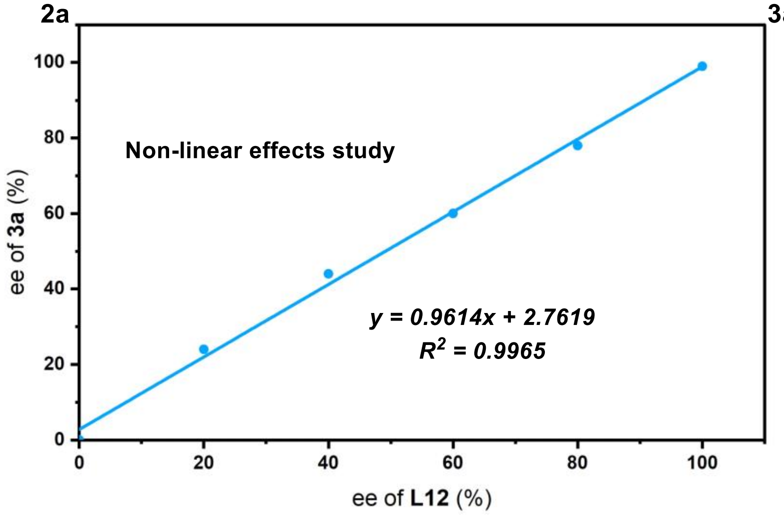

Supplementary Fig. 4. Non-linear effect study

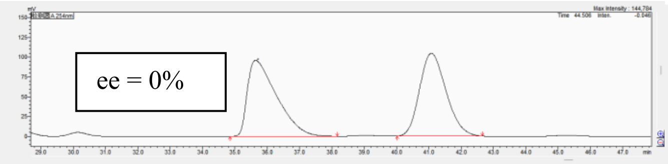

峰号	保留时间	面积	高度	标记	浓度	浓度单位	化合物ID号	化合物名	面积%
1	35.640	6045308	96260	M	50.358				50.358
2	41.076	5959338	104530	M	49.642				49.642
总计		12004646	200790		100.000				100.000

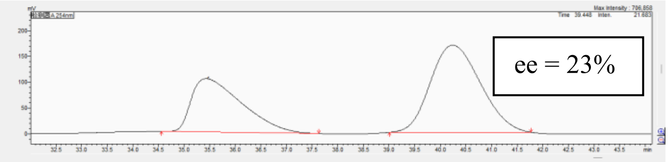

峰号	保留时间	面积	高度	标记	浓度	浓度单位	化合物ID号	化合物名	面积%
1	35.429	7113054	103697	M	38.291				38.291
2	40.242	11463477	168869	M	61.709				61.709
总计		18576531	272567		100.000				100.000

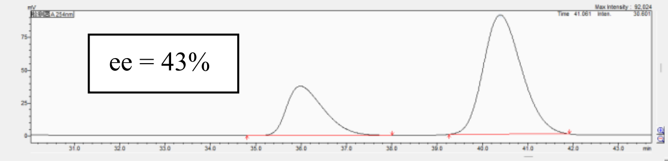

峰号	保留时间	面积	高度	标记	浓度	浓度单位	化合物ID号	化合物名	面积%
1	35.983	2112857	37474	M	28.361				28.361
2	40.393	5336902	90560	M	71.639				71.639
总计		7449759	128035		100.000				100.000

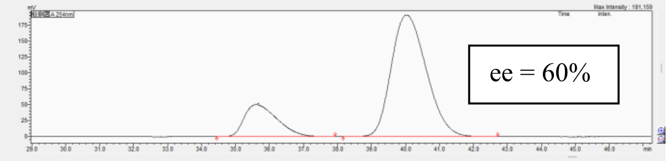

峰号	保留时间	面积	高度	标记	浓度	浓度单位	化合物ID号	化合物名	面积%
1	35.608	3392144	50346		19.824				19.824
2	40.033	13719445	191506		80.176				80.176
总计		17111589	241852		100.000				100.000

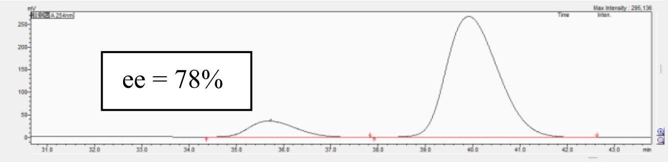

峰号	保留时间	面积	高度	标记	浓度	浓度单位	化合物ID号	化合物名	面积%
1	35.685	2460231	35619		10.837				10.837
2	39.917	20241240	267636		89.163				89.163
总计		22701471	303256		100.000				100.000

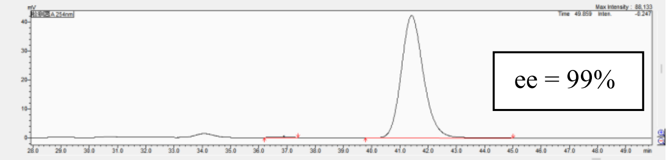

峰号	保留时间	面积	高度	标记	浓度	浓度单位	化合物ID号	化合物名	面积%
1	36.826	7854	198	M	0.339				0.339
2	41.418	2309995	42327	M	99.661				99.661
总计		2317849	42525		100.000				100.000

9.5 Preparation of the nickel complex L12•NiBr2 and its reactivity

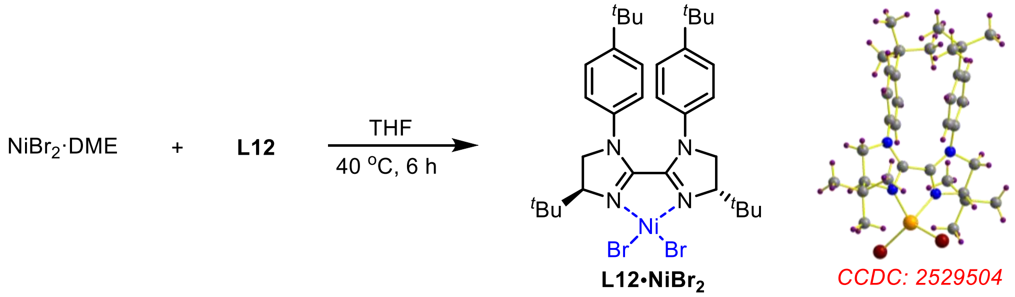

In an argon-filled glovebox, $NiBr_2$•DME (155.0 mg, 0.5 mmol, 1.0 equiv), **L12** (257 mg, 0.5 mmol, 1.0 equiv), THF (5 mL) were added to a 10 mL Schlenk flask. The reaction mixture was stirred at 40 ºC for 6 h. the reaction mixture concentrated in vacuo to give deep purper solid.

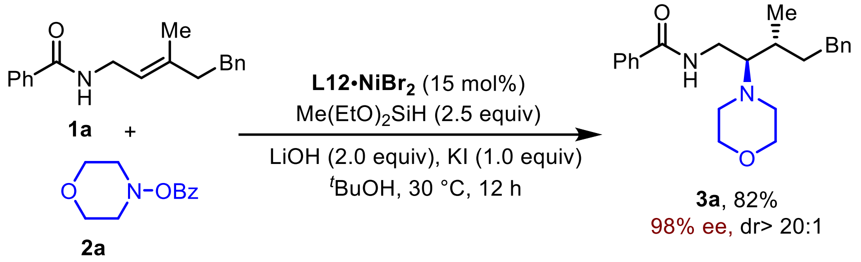

In an argon-filled glovebox, **L12•NiBr2** (21.9 mg, 0.03 mmol, 15 mol%), (*E*)-*N*-(3-methyl-5-phenylpent-2-en-1-yl)benzamide (**1a**) (55.8 mg, 0.20 mmol, 1.0 equiv), morpholino benzoate (**2a**) (62.1 mg, 0.30 mmol, 1.5 equiv), LiOH (9.6 mg, 0.40 mmol, 2.0 equiv), KI (33.2 mg, 0.20 mmol, 1.0 equiv), $Me(EtO)_2SiH$ (80 µL, 0.50 mmol, 2.5 equiv), Anhydrous *tert*-butyl alcohol (1.0 mL, 0.20 M). The reaction mixture was stirred for 12 h at 30 °C. After the reaction time, the vessel was allowed to silica gel column chromatography. The crude product was purified by column chromatography on silica gel with a mixture of ethyl acetate and petroleum ether as eluent. The conditions for flash chromatography and data for characterization of the products are listed below.

9.6 HR-MS (ESI) analysis of the reaction mixture.

In an argon-filled glovebox, $NiBr_2$•DME (6.2 mg, 0.02 mmol, 10 mol%), **L12** (15.4 mg, 15 mol%), (*E*)-*N*-(3-methyl-5-phenylpent-2-en-1-yl)benzamide (**1a**) (55.8 mg, 0.20 mmol, 1.0 equiv), morpholino benzoate (**2a**) (62.1 mg, 0.30 mmol, 1.5 equiv), LiOH (9.6 mg, 0.40 mmol, 2.0 equiv), KI (33.2 mg, 0.20 mmol, 1.0 equiv), $Me(EtO)_2SiH$ (80 µL, 0.50 mmol, 2.5 equiv), Anhydrous *tert*-butyl alcohol (1.0 mL,

0.20 M). The reaction mixture was stirred for 12 h at 30 °C. After completion, the reaction mixture was sampled for HRMS-ESI analysis.

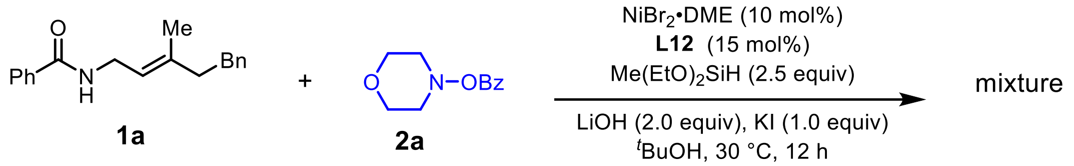

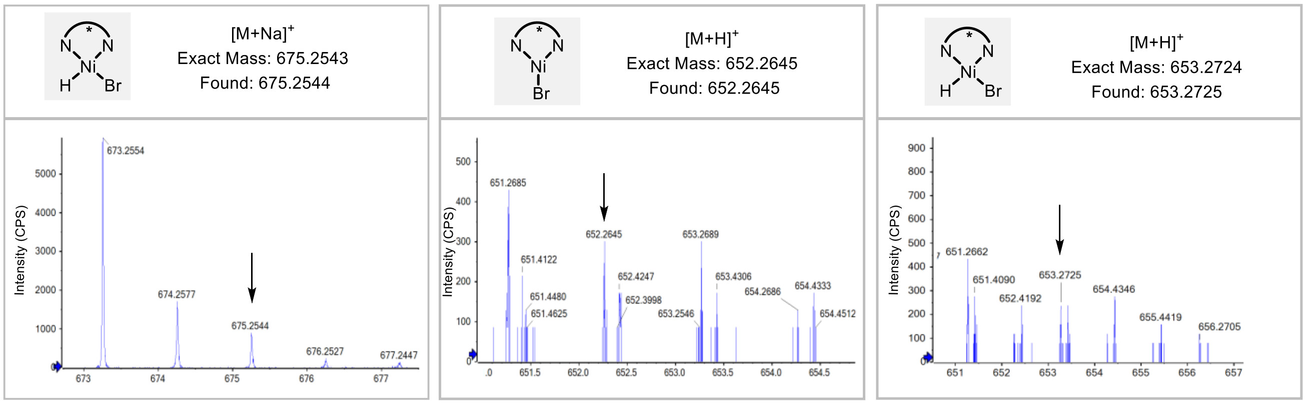

Supplementary Fig. 5. HR-MS spectra of reaction mixture

10.X-ray crystallographic data

Single crystals for X-ray studies were grown by slow evaporation of a solution of compound **4b** in a mixture of petroleum ether and tetrahydrofuran at room temperature. X-Ray structural analysis of single crystal **4b** was obtained to confirm the absolute configuration. The X-ray data of **4b** is deposited in the Cambridge Crystallographic Data Centre with a number of **CCDC 2403087**.

Crystal Data for C29H34N2O2 (M = 442.58 g/mol): orthorhombic, space group P21 (4), a = 8.7469(5) Å, b = 5.5489(3) Å, c = 25.1685(14) Å, V = 1219.35(11) Å3, Z = 2, T = 293(2) K, μ (Cu K α) = 0.588 mm-1, Dcalc = 1.203 g/cm3, 8194 reflections measured (7.04° ⩽ 2Θ ⩽ 134.14°), 4240 unique (Rint = 0.0442, Rsigma = 0.0433) which were used in all calculations. The final R1 was 0.0528 (I > 2 σ (I)) and wR2 was 0.1564 (all data).

Supplementary Table 6. Crystal data and structure refinement for **4b.**

Identification code	a250225a
Empirical formula	$C_{29}H_{34}N_2O_2$
Formula weight	442.58
Temperature/K	293(2)
Crystal system	monoclinic
Space group	$P2_1$ (4)
a/Å	8.7469(5)
b/Å	5.5489(3)
c/Å	25.1685(14)
α/°	90
β/°	93.461(5)
γ/°	90
Volume/Å^3	1219.35(11)
Z	2
ρcalcg/cm^3	1.205
μ/mm-1	0.588
F(000)	476
Crystal size/mm^3	0.12×0.13×0.25

Radiation	Cu K_α (λ=1.54184 Å)
2Θ range for data collection/	7.04 to 134.14 (0.84 Å)
Index ranges	$-10 \le h \le 10$, $-6 \le k \le 6$, $-30 \le l \le 25$
Reflections collected	8194
Independent reflections	4240 [R_{int} = 0.0442, R_{sigma} = 0.0433
Data/restraints/parameters	4240/1/294
Goodness-of-fit on F^2	1.030
Final R indexes [I>=2σ (I)]	R_1 = 0.0528, wR_2 = 0.1416
Final R indexes [all data]	R_1 = 0.0692, wR_2 = 0.1564
Largest diff. peak/hole / e Å-3	0.15/-0.14
Flack parameter	-0.1(3)

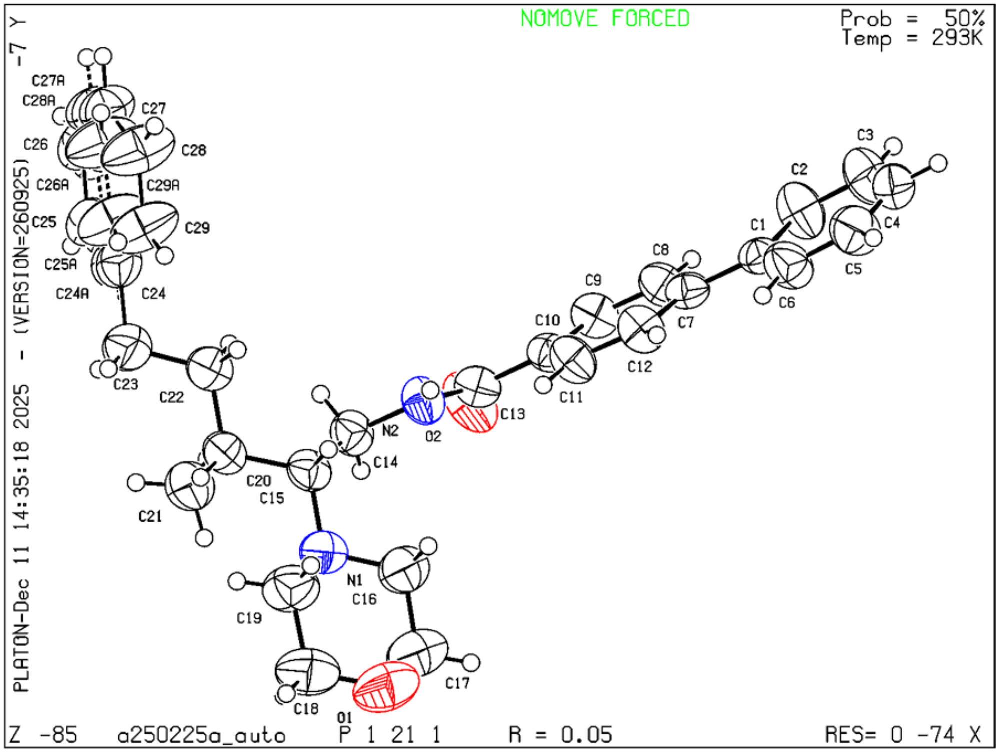

Supplementary Fig. 6. X-ray structure of compound **4b** (The thermal ellipsoid was drawn at the 50% probability level). **CCDC: 2403087**

Single crystals for X-ray studies were grown by slow evaporation of a solution of compound **L12·NiBr2** in a mixture of petroleum ether and tetrahydrofuran at room temperature. X-Ray structural analysis of single crystal **L12·NiBr2** was obtained to confirm the absolute configuration. The X-ray data of **L12·NiBr2** is deposited in the Cambridge Crystallographic Data Centre with a number of **CCDC 2529504**.

Crystal Data for $C_{40}H_{62}Br_2N_4NiO_{1.50}$ (M = 841.46 g/mol): orthorhombic, space group $P6_1$ (169), a = 22.2320(12) Å, b = 22.2320(12) Å, c = 16.5775(6) Å, V = 7095.9(8) Å3, Z = 6, T = 293(2) K, μ(Cu Kα) = 2.803 mm^{-1}, D_{calc} = 1.181 g/cm^3, 36723 reflections measured (4.59° ≤ 2Θ ≤ 146.99°), 9341 unique (R_{int} = 0.0557, R_{sigma} = 0.0395) which were used in all calculations. The final R_1 was 0.0623 (I > 2σ(I)) and wR_2 was 0.2272 (all data).

Supplementary Table 7. Crystal data and structure refinement for **L12·NiBr2.**

Identification code	a241104a
Empirical formula	$C_{40}H_{62}Br_2N_4NiO_{1.50}$
Formula weight	841.46
Temperature/K	293(2)
Crystal system	hexagonal
Space group	$P6_1$ (169)
a/Å	22.2320(12)
b/Å	22.2320(12)
c/Å	16.5775(6)
α/°	90
β/°	90
γ/°	120
Volume/Å^3	7095.9(8)
Z	6
ρcalcg/cm^3	1.181
μ/mm-1	2.803
F(000)	2640
Crystal size/mm^3	0.1×0.12×0.25

Radiation	Cu K_{α} (λ=1.54184 Å)
2Θ range for data collection/	4.59 to 146.99 (0.80 Å)
Index ranges	$-26 \leq h \leq 26$, $-27 \leq k \leq 26$, $-20 \leq l \leq 20$
Reflections collected	36723
Independent reflections	9341 [R_{int} = 0.0557, R_{sigma} = 0.0395
Data/restraints/parameters	9341/210/388
Goodness-of-fit on F^2	1.068
Final R indexes [I>=2σ (I)]	R_1 = 0.0623, wR_2 = 0.1862
Final R indexes [all data]	R_1 = 0.1022, wR_2 = 0.2272
Largest diff. peak/hole / e Å-3	0.56/-0.53
Flack parameter	0.001(14)

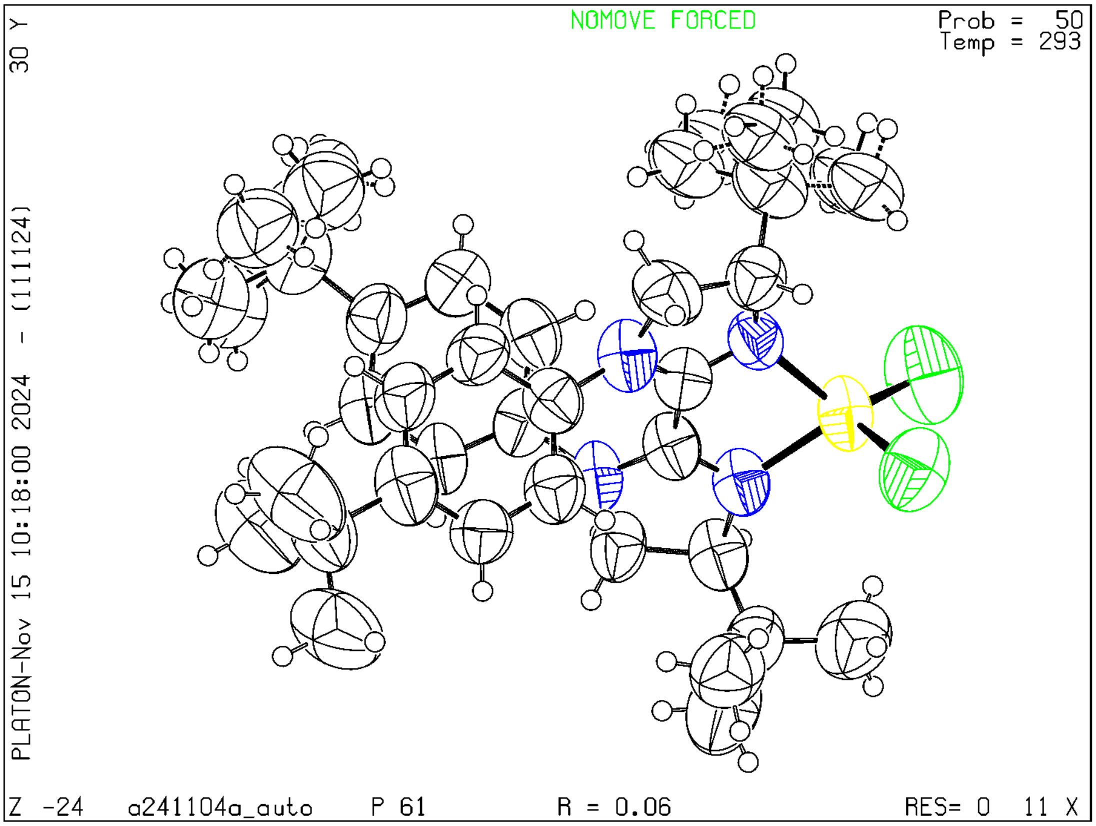

Supplementary Fig. 7. X-ray structure of compound **L12·NiBr₂** (The thermal ellipsoid was drawn at the 50% probability level). **CCDC: 2529504**

11. NMR spectra

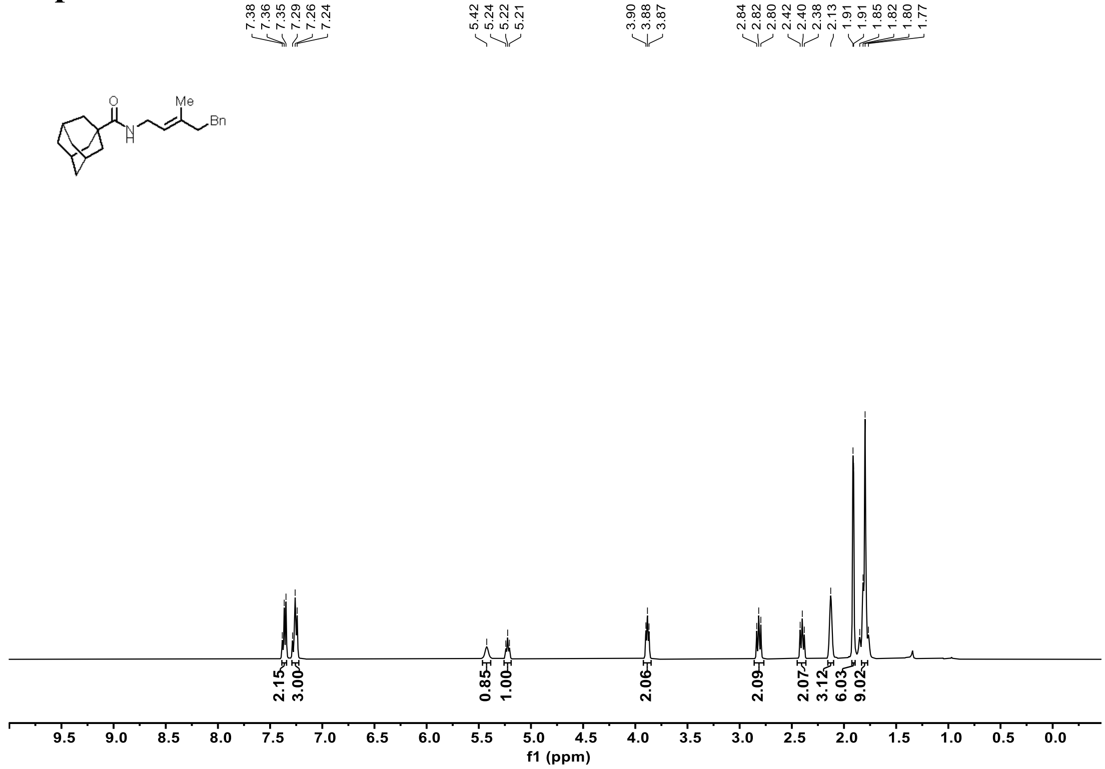

^{1}H NMR (400 MHz, $CDCl_3$) spectra of **1i**

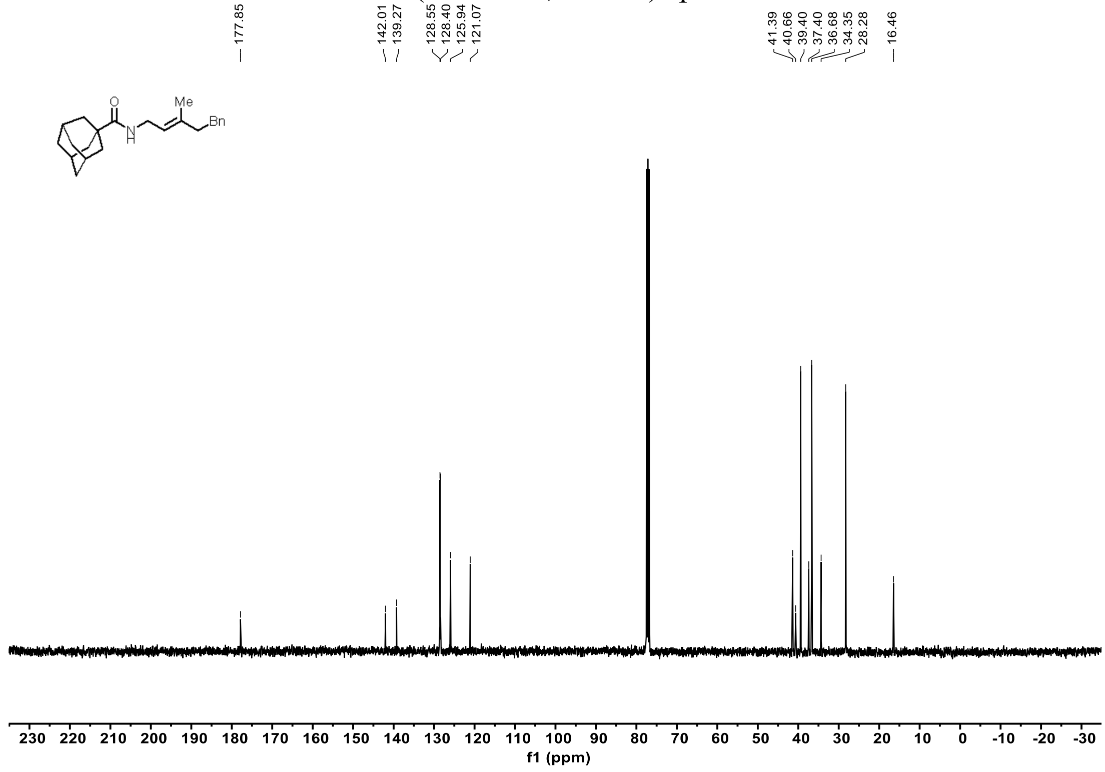

^{13}C NMR (101 MHz, $CDCl_3$) spectra of **1i**

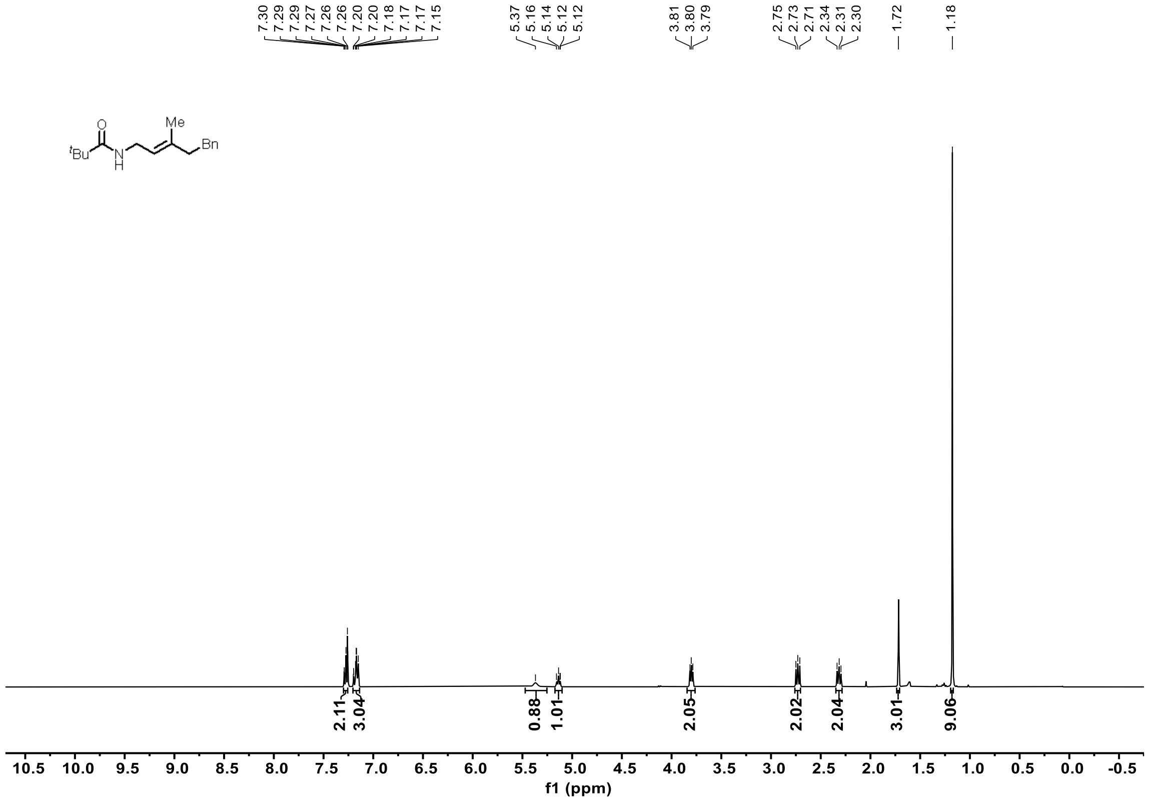

^{1}H NMR (400 MHz, $CDCl_3$) spectra of **1h**

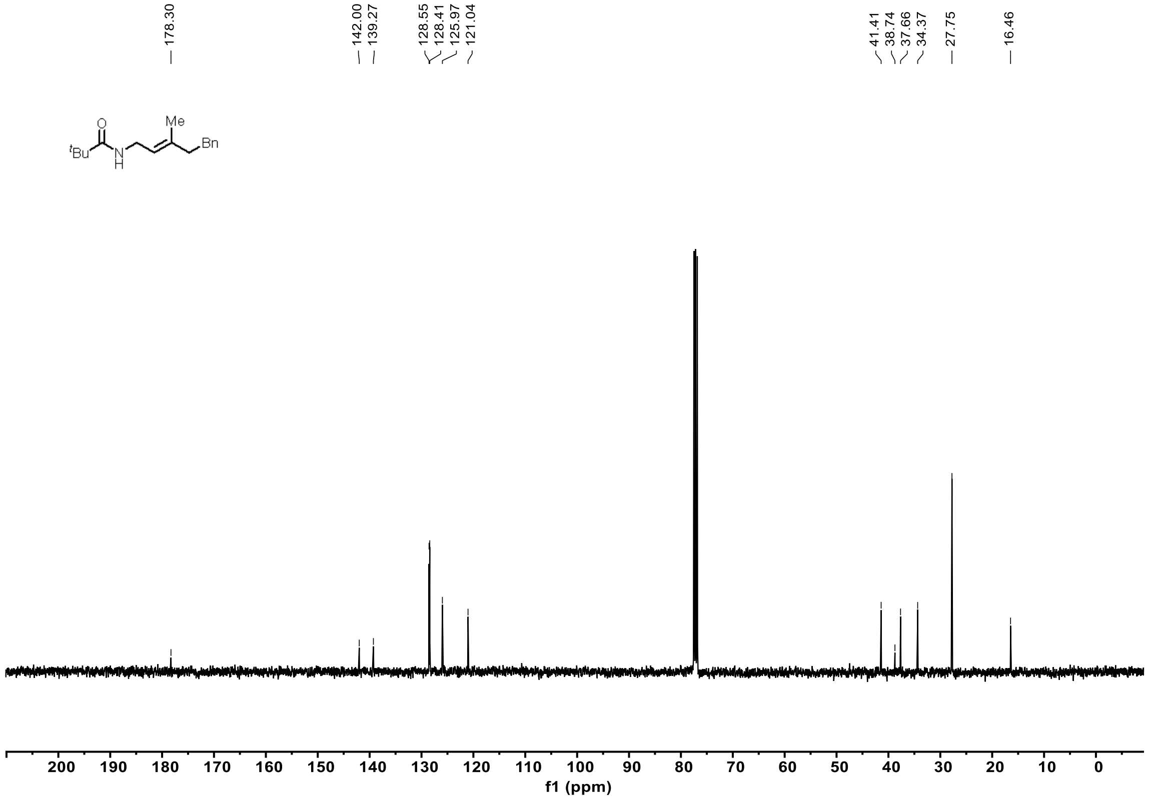

^{13}C NMR (101 MHz, $CDCl_3$) spectra of **1h**

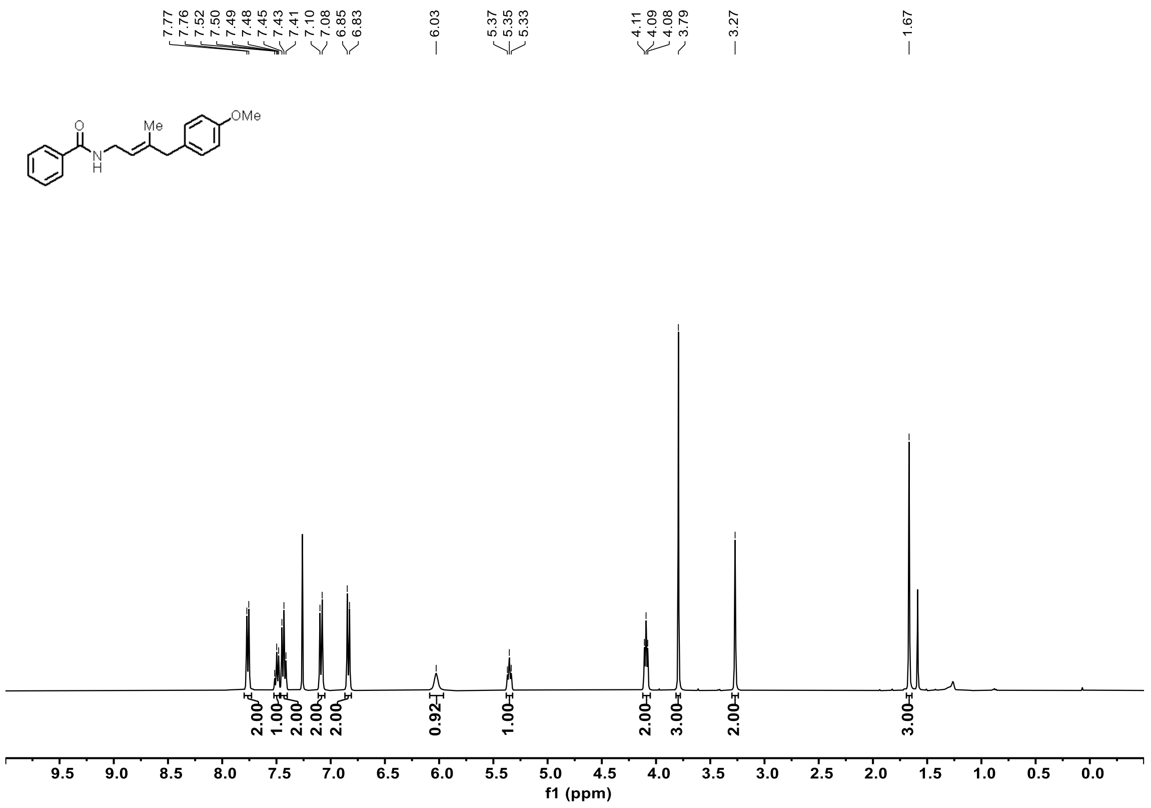

^{1}H NMR (400 MHz, $CDCl_3$) spectra of **1j**

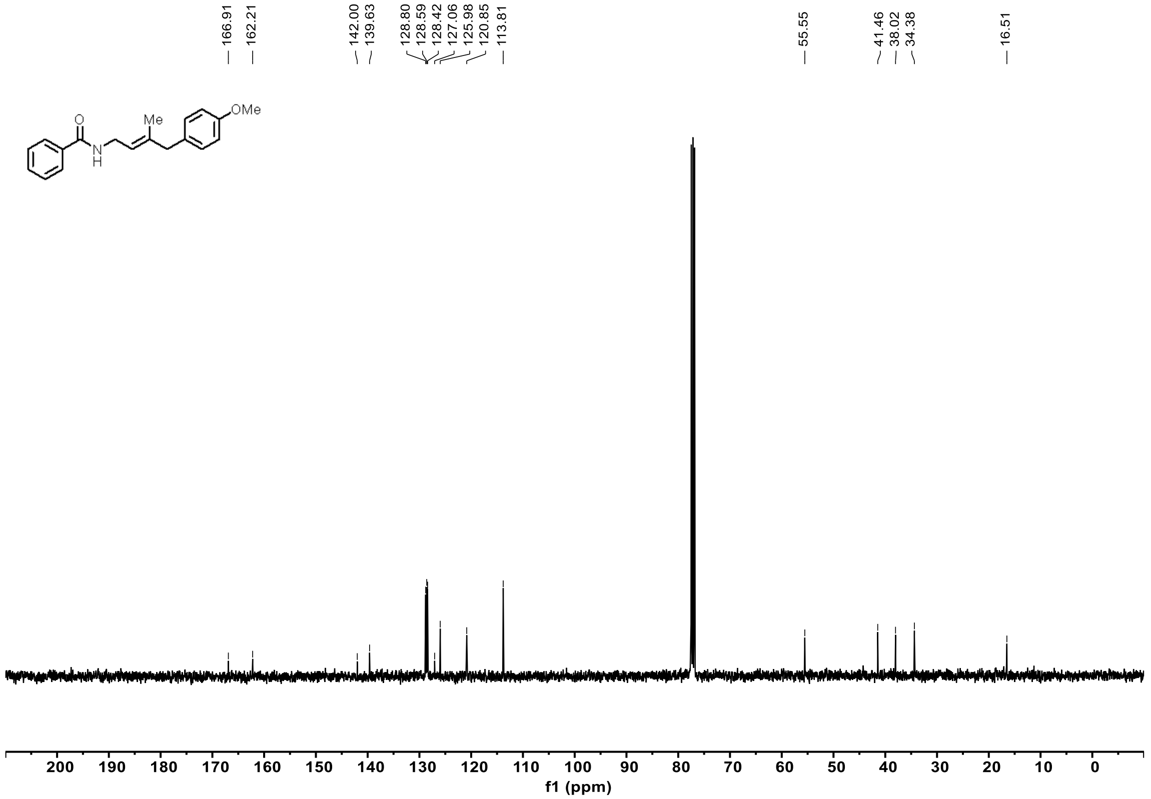

^{13}C NMR (101 MHz, $CDCl_3$) spectra of **1j**

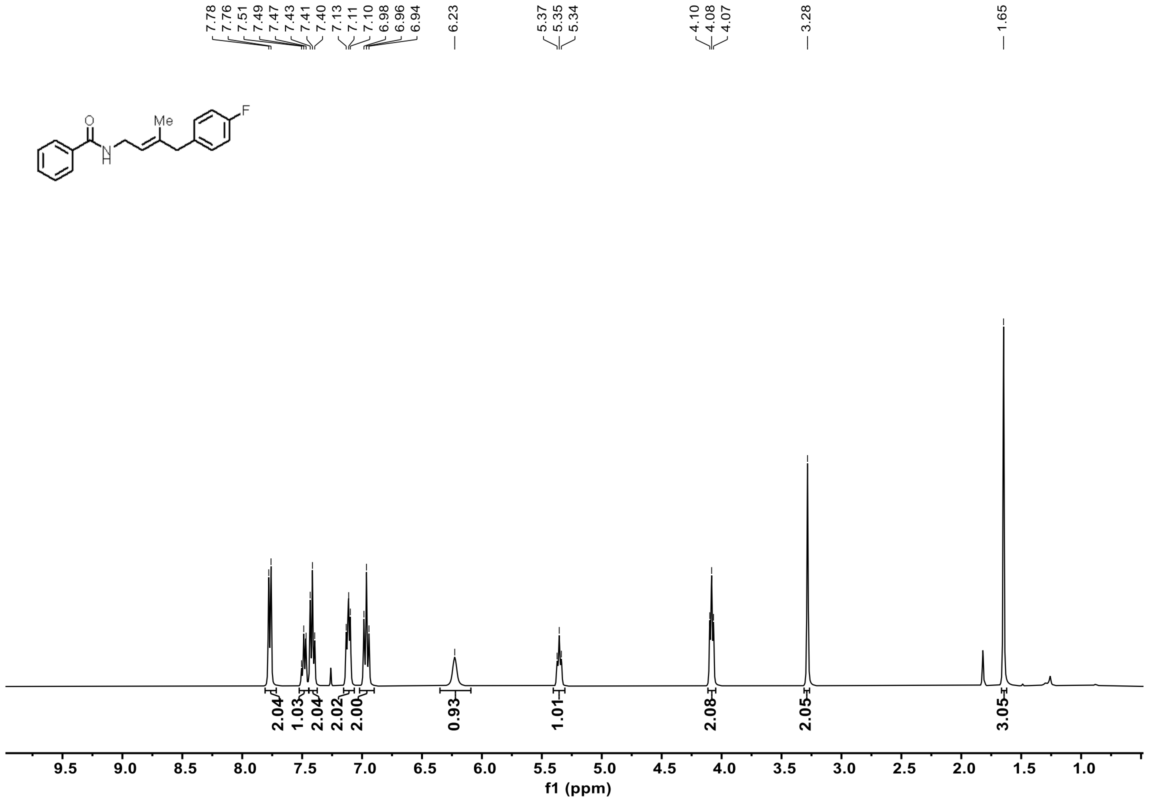

^{1}**H NMR** (400 MHz, $CDCl_3$) spectra of **1k**

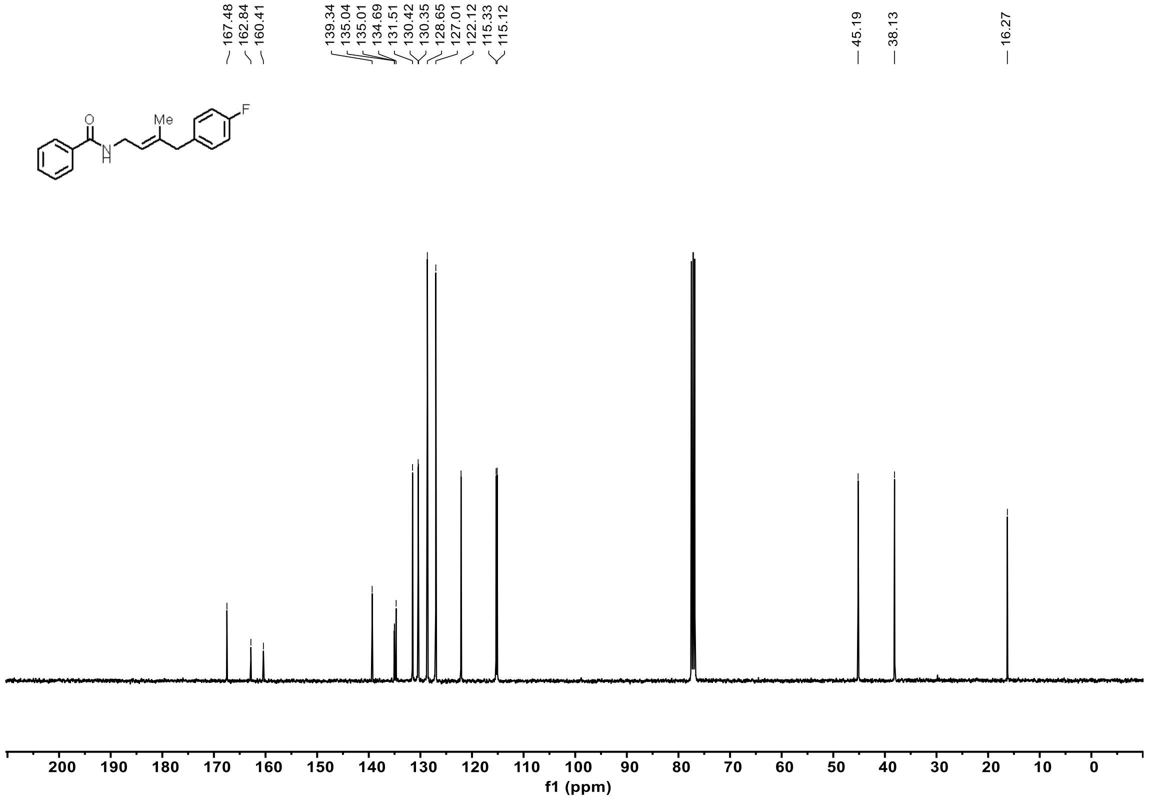

^{13}**C NMR** (101 MHz, $CDCl_3$) spectra of **1k**

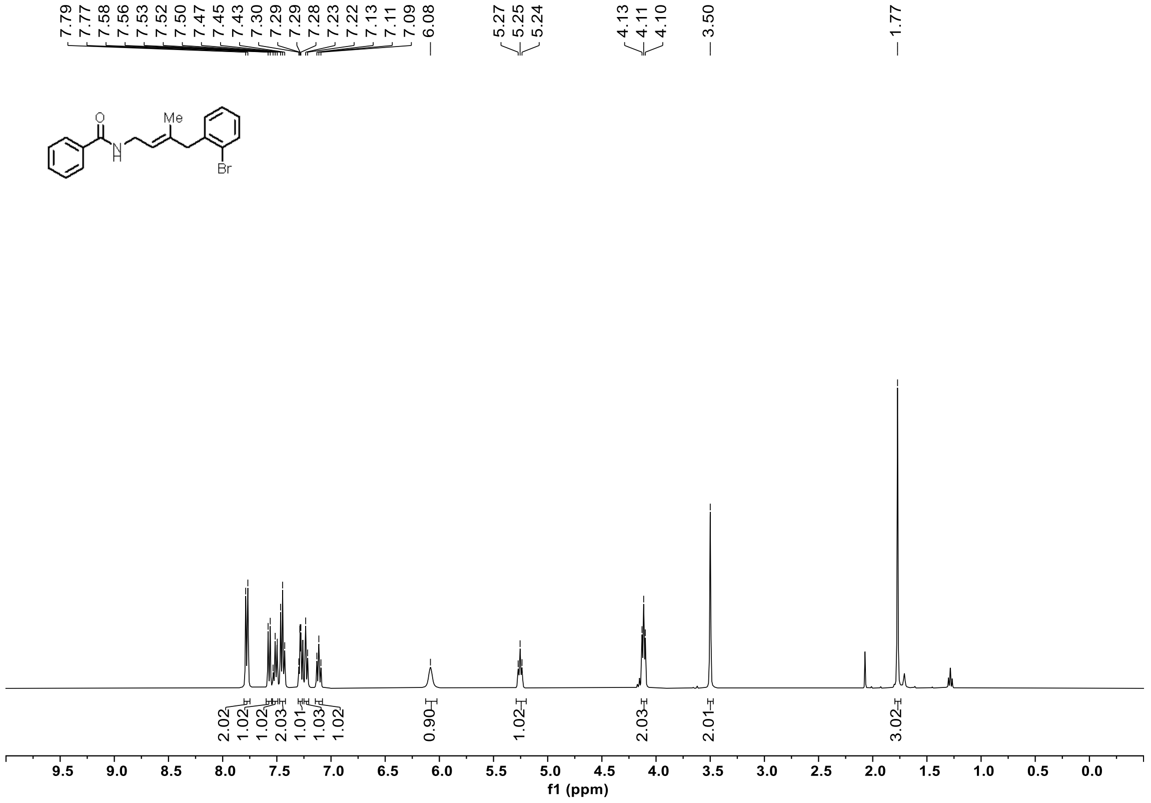

^{1}**H NMR** (400 MHz, $CDCl_3$) spectra of **1l**

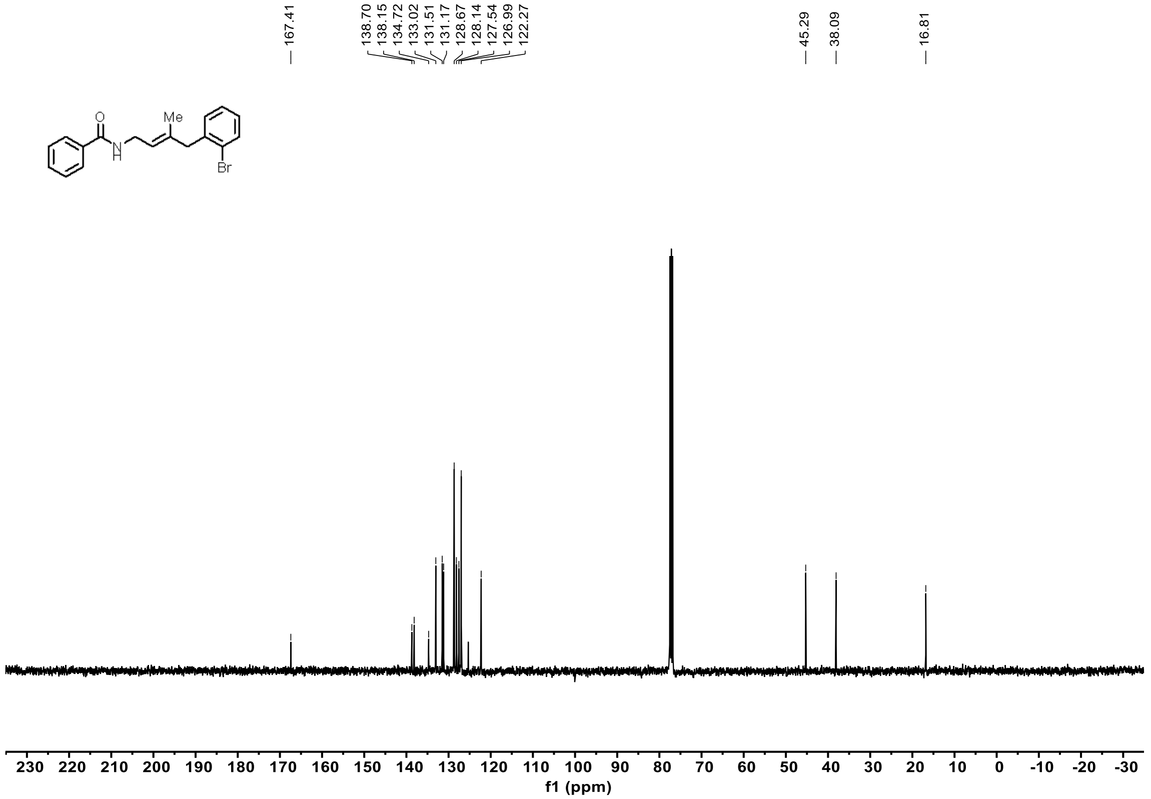

^{13}**C NMR** (101 MHz, $CDCl_3$) spectra of **1l**

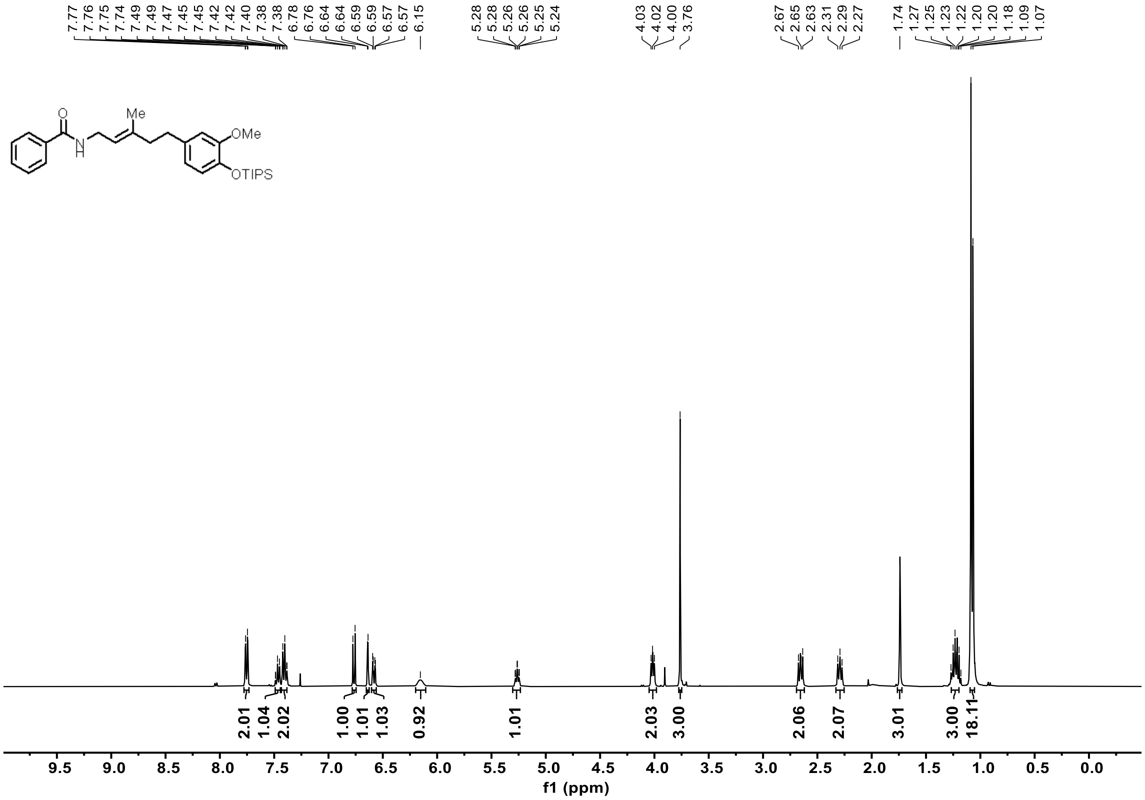

^{1}H NMR (400 MHz, $CDCl_3$) spectra of **1n**

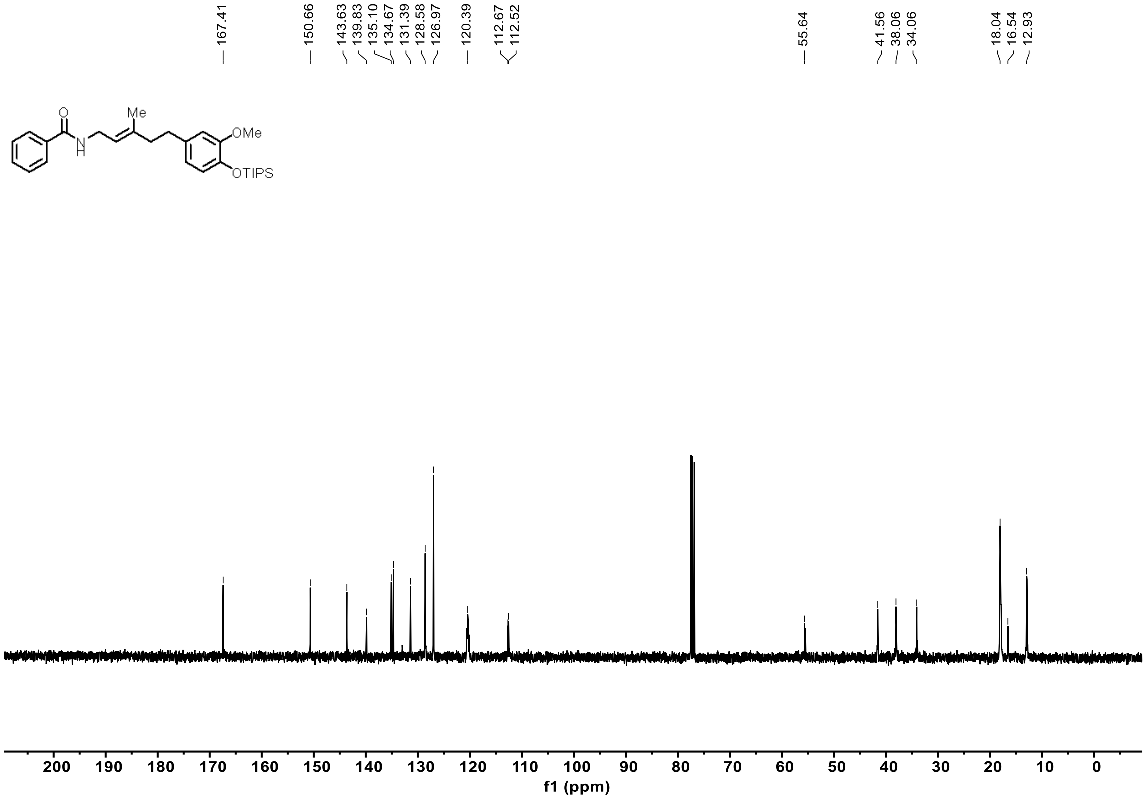

^{13}C NMR (101 MHz, $CDCl_3$) spectra of **1n**

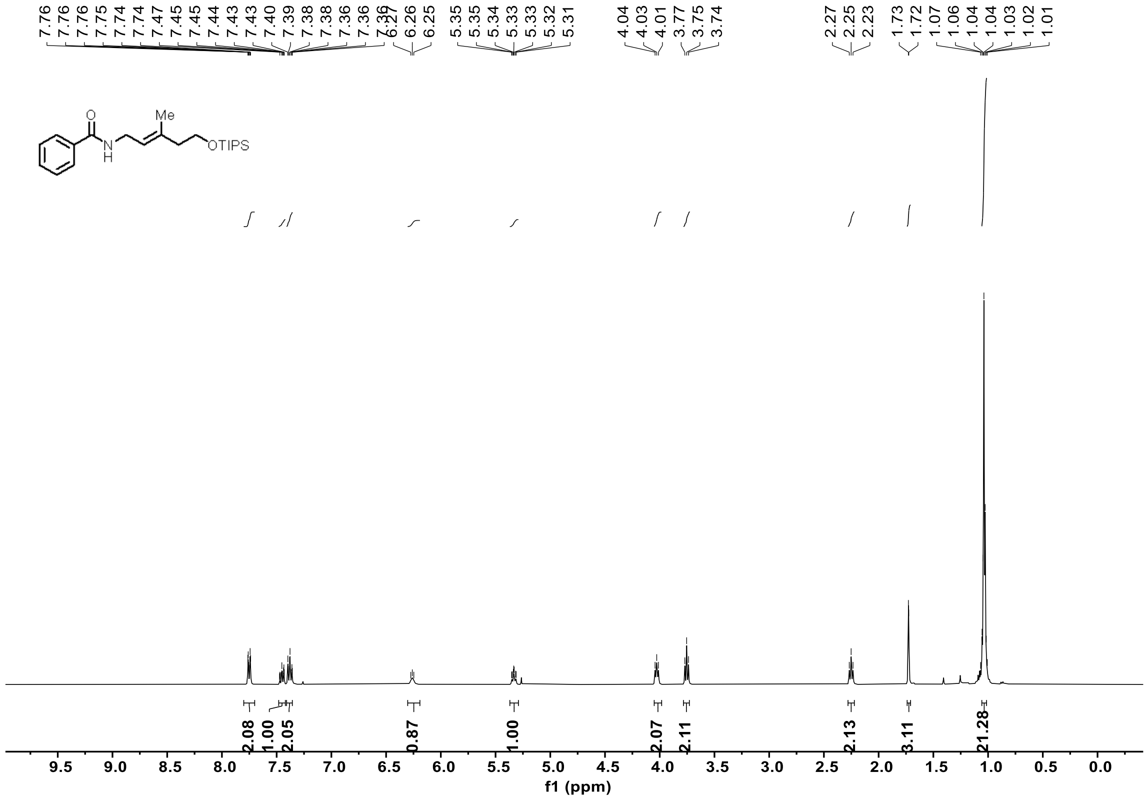

^{1}H NMR (400 MHz, $CDCl_3$) spectra of **1t**

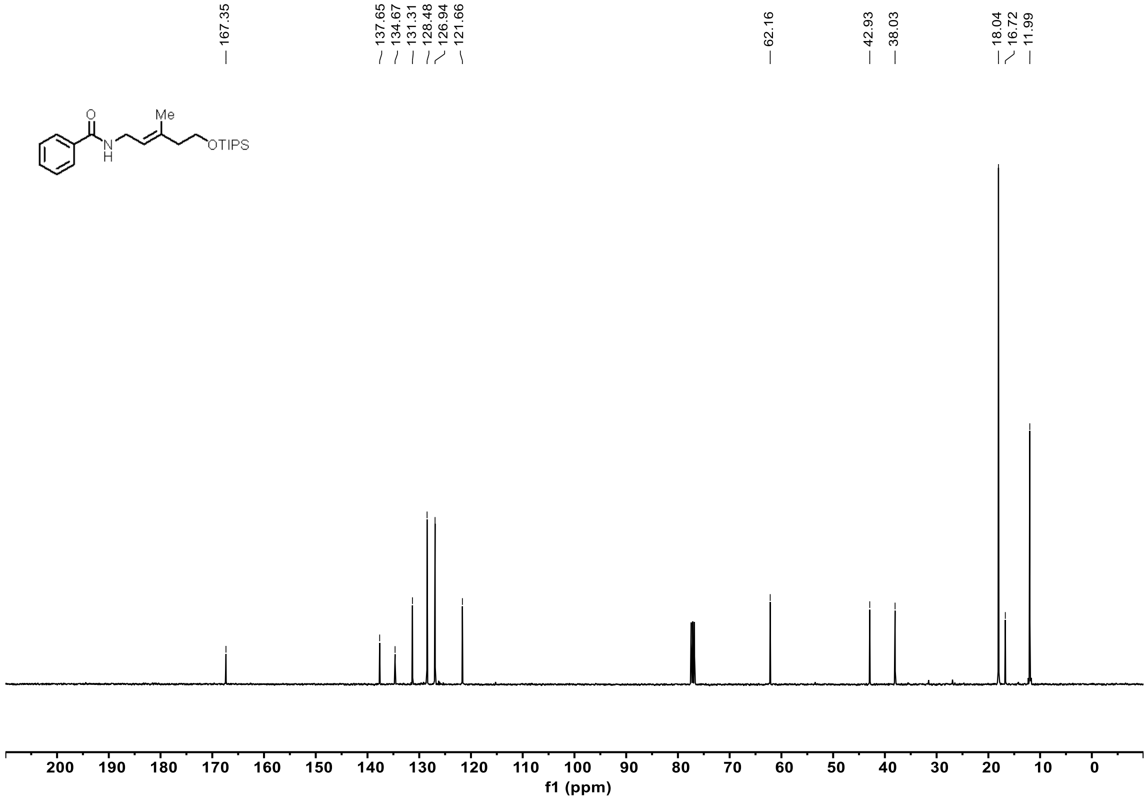

^{13}C NMR (101 MHz, $CDCl_3$) spectra of **1t**

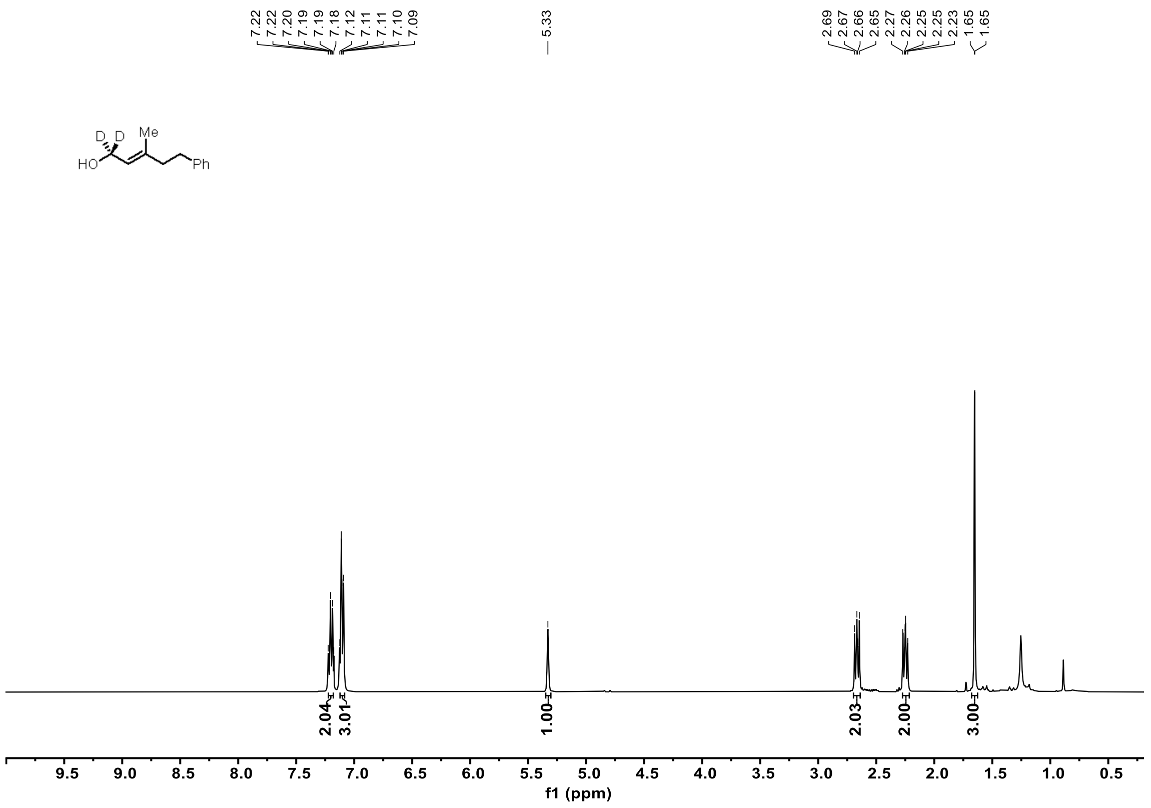

^{1}H NMR (400 MHz, CDCl$_3$) spectra of **sub-1**

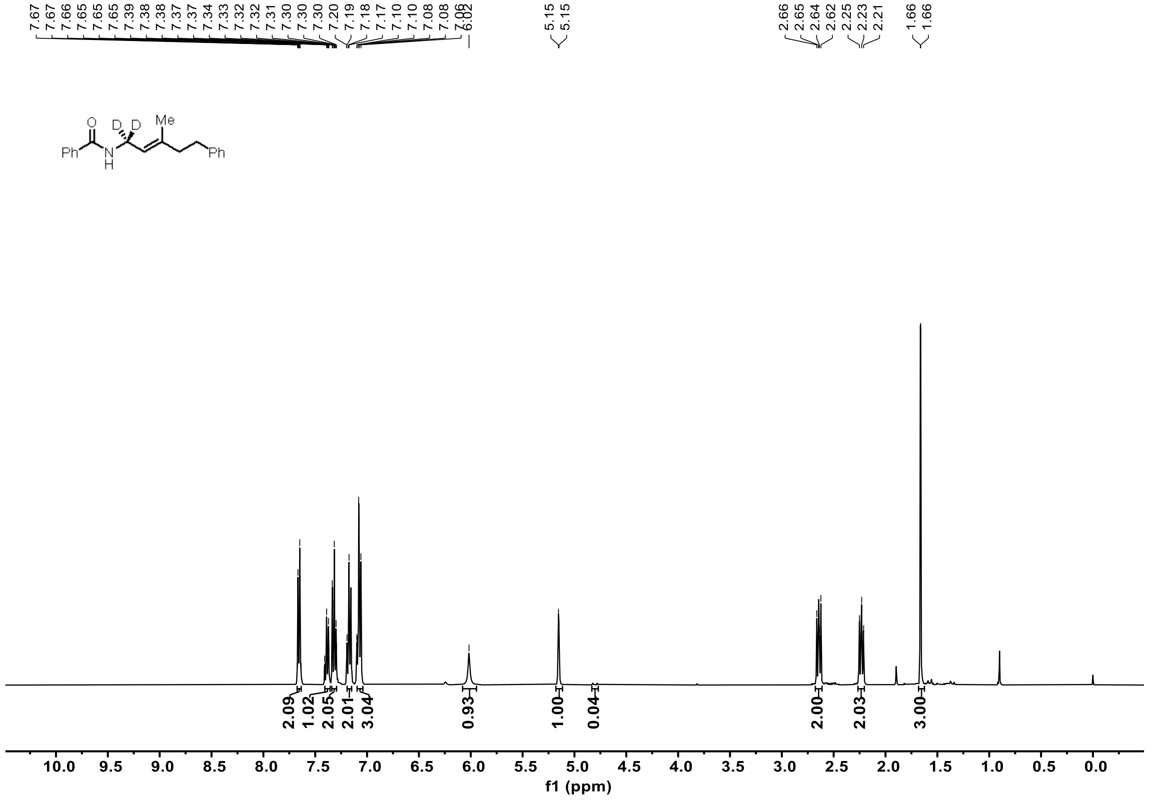

^{1}H NMR (400 MHz, CDCl$_3$) spectra of **1a-*d***

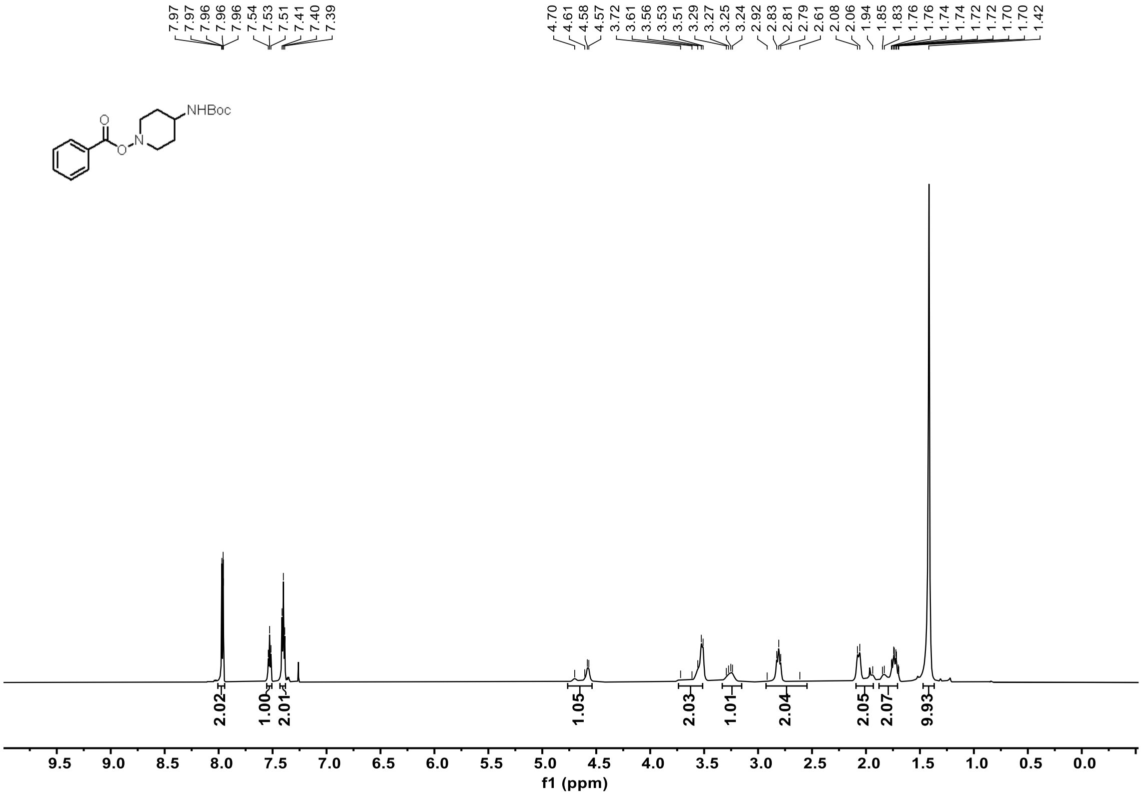

^{1}H NMR (600 MHz, $CDCl_3$) spectra of **2e**

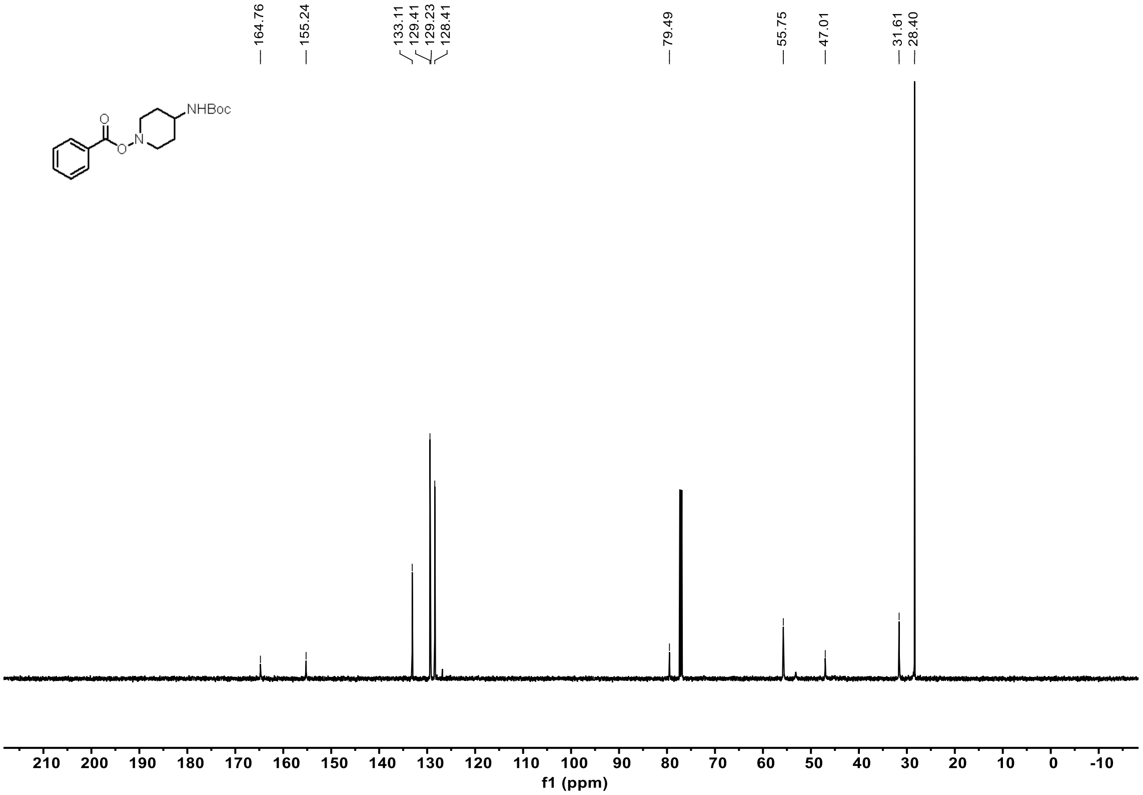

^{13}C NMR (151 MHz, $CDCl_3$) spectra of **2e**

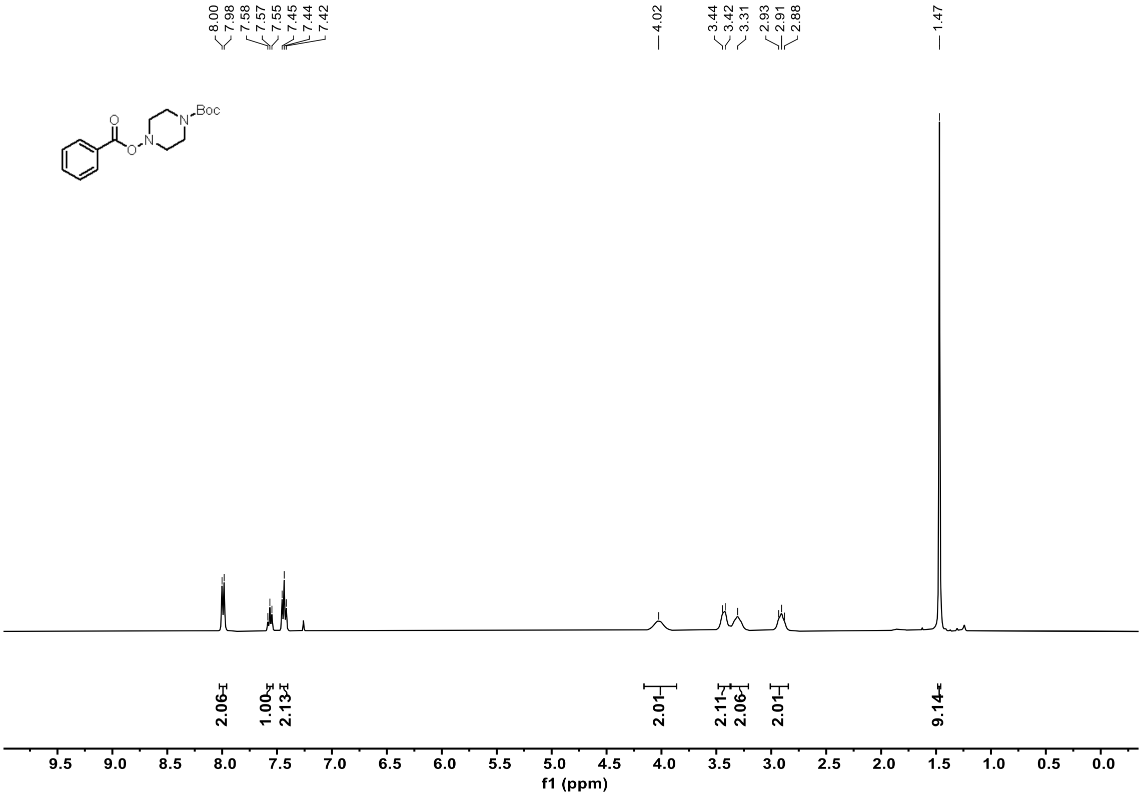

^{1}H NMR (400 MHz, $CDCl_3$) spectra of **2f**

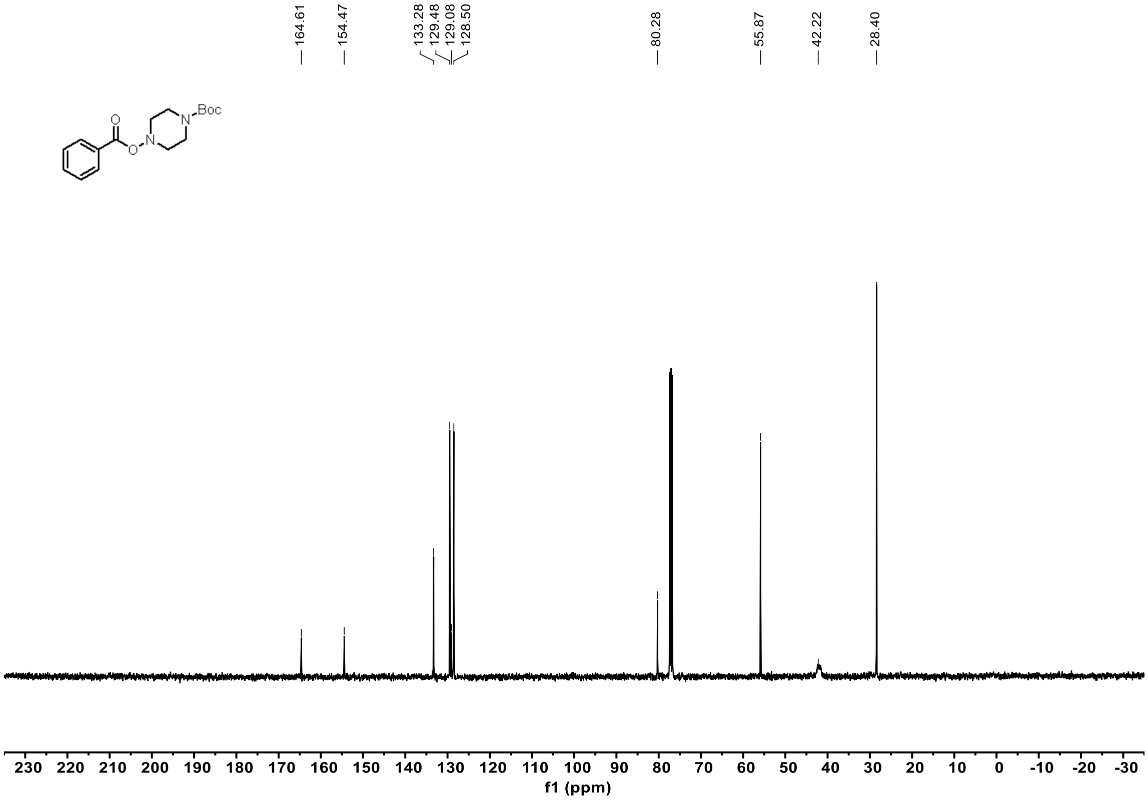

^{13}C NMR (101 MHz, $CDCl_3$) spectra of **2f**

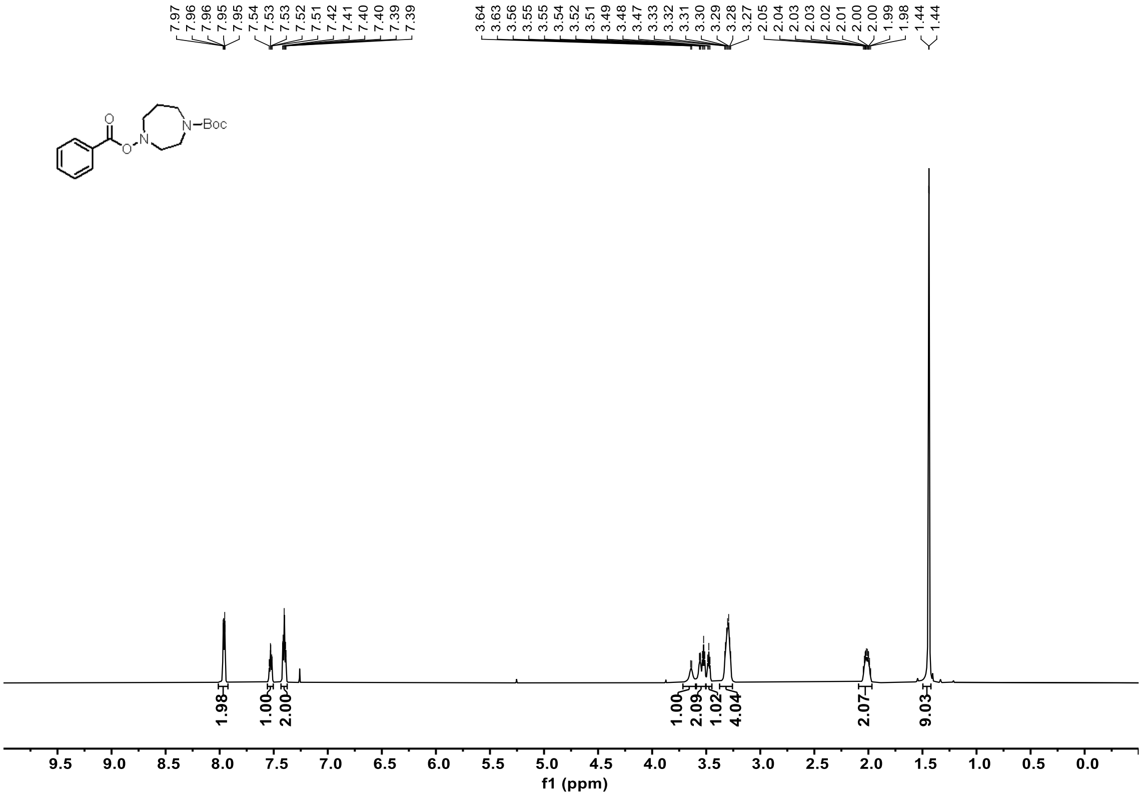

^{1}H NMR (600 MHz, $CDCl_3$) spectra of **2g**

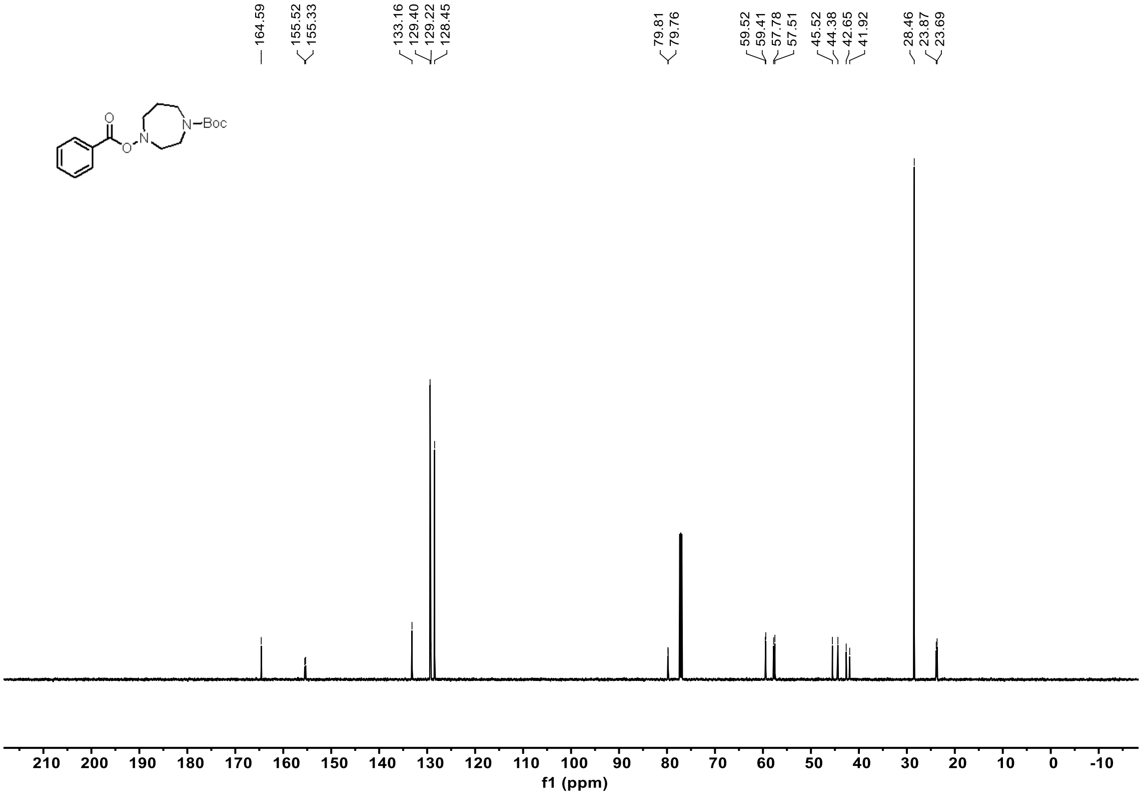

^{13}C NMR (151 MHz, $CDCl_3$) spectra of **2g**

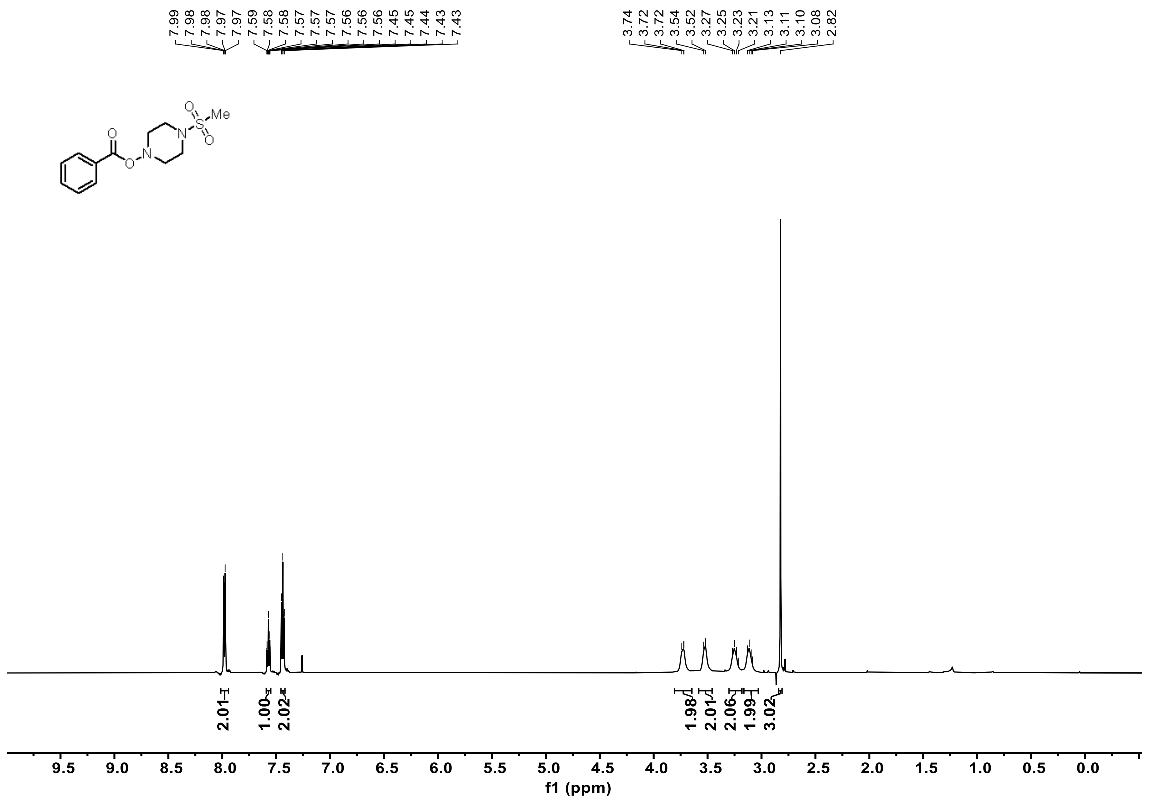

^{1}H NMR (600 MHz, $CDCl_3$) spectra of **2h**

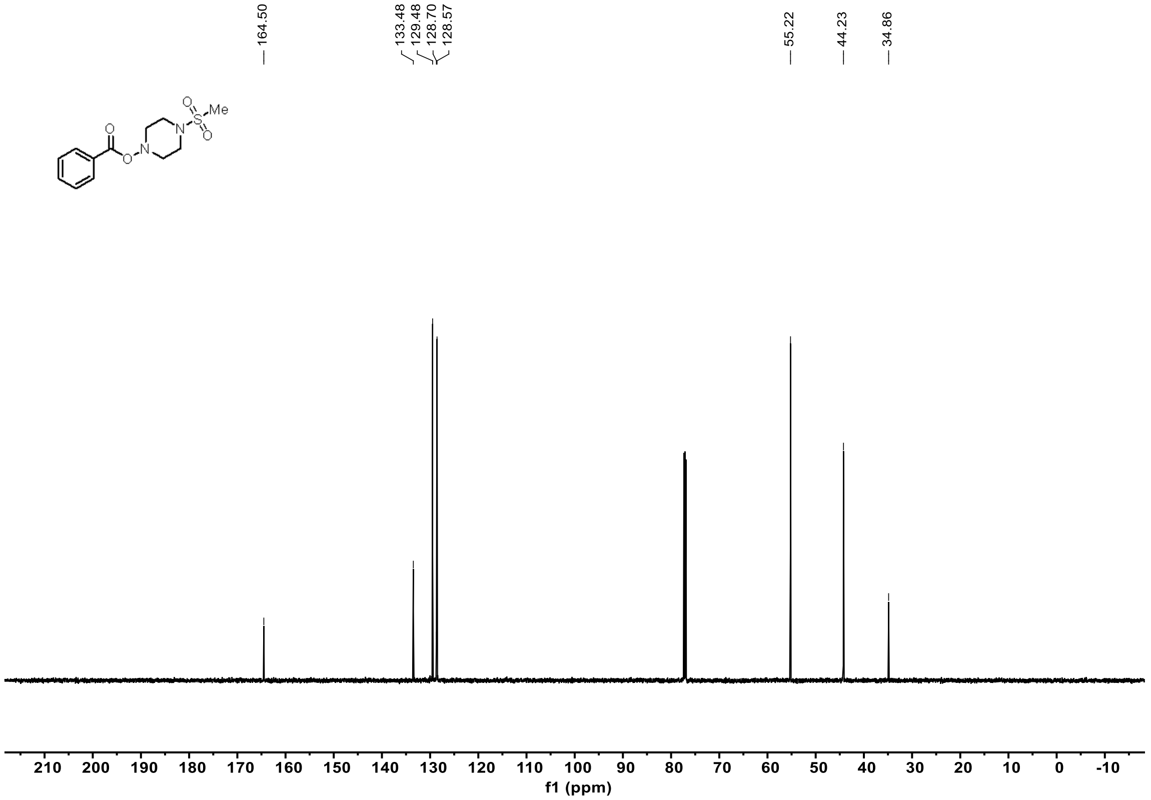

^{13}C NMR (151 MHz, $CDCl_3$) spectra of **2h**

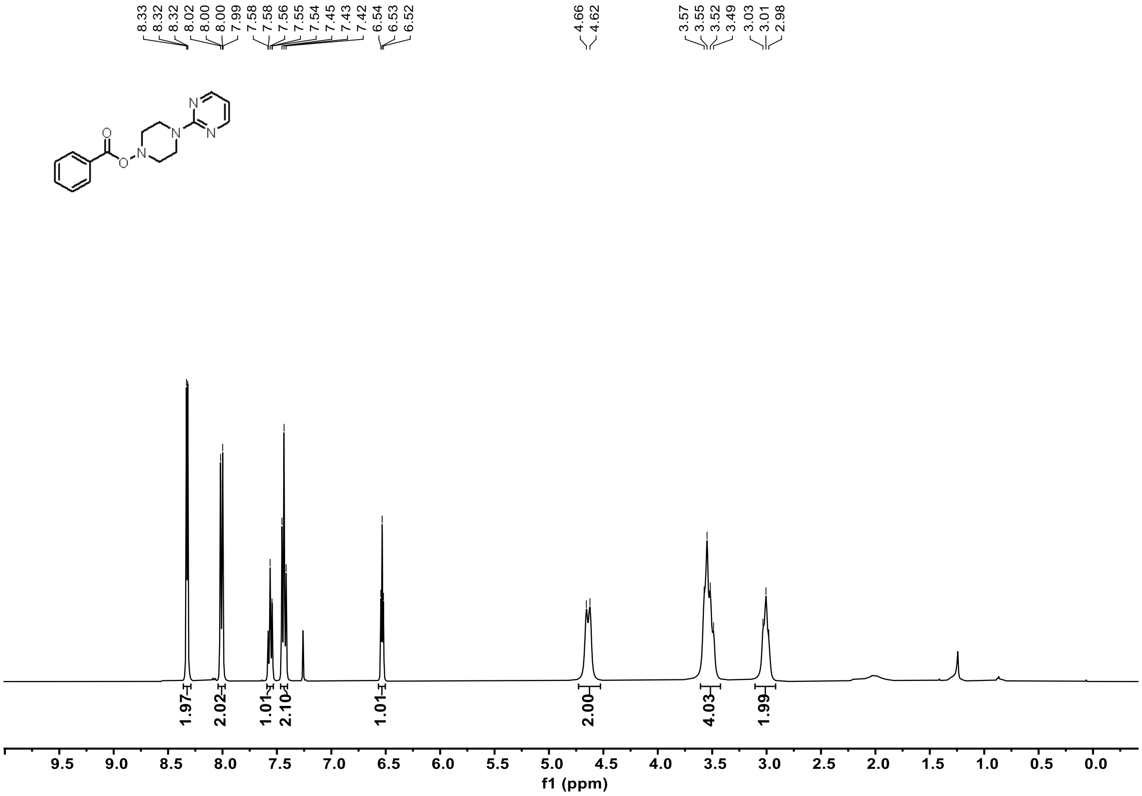

^{1}**H NMR** (400 MHz, $CDCl_3$) spectra of **2i**

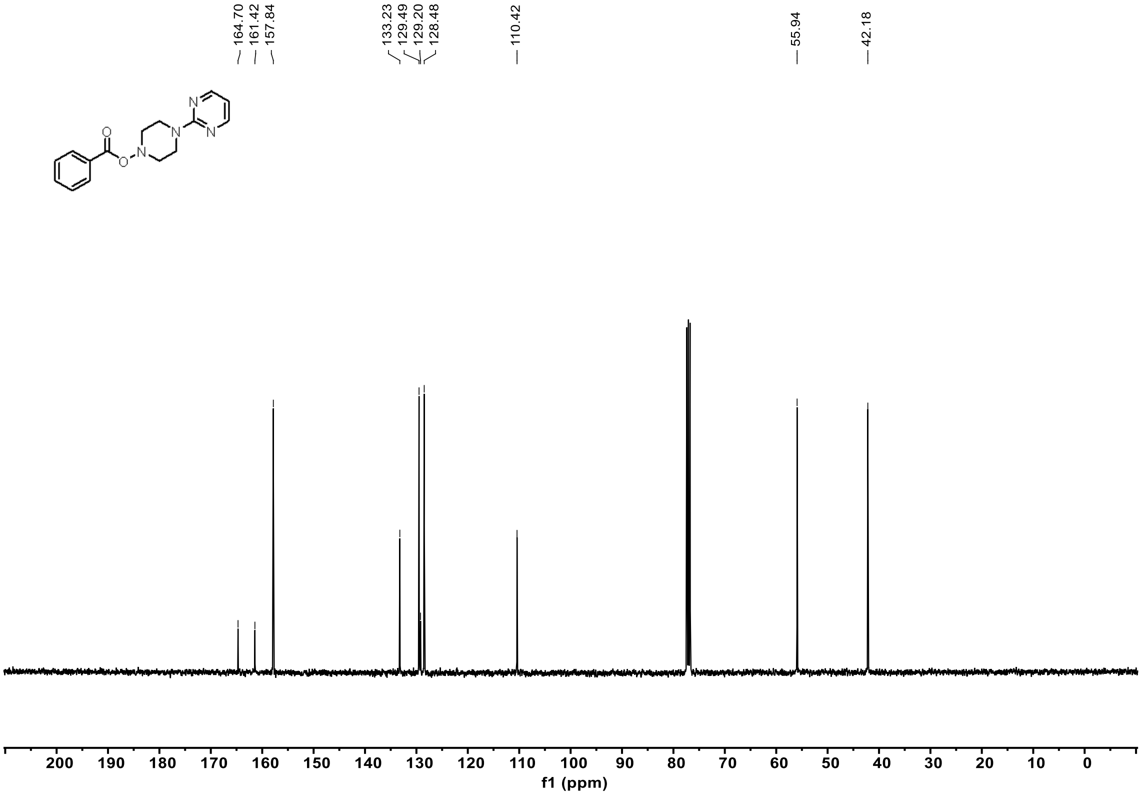

^{13}**C NMR** (101 MHz, $CDCl_3$) spectra of **2i**

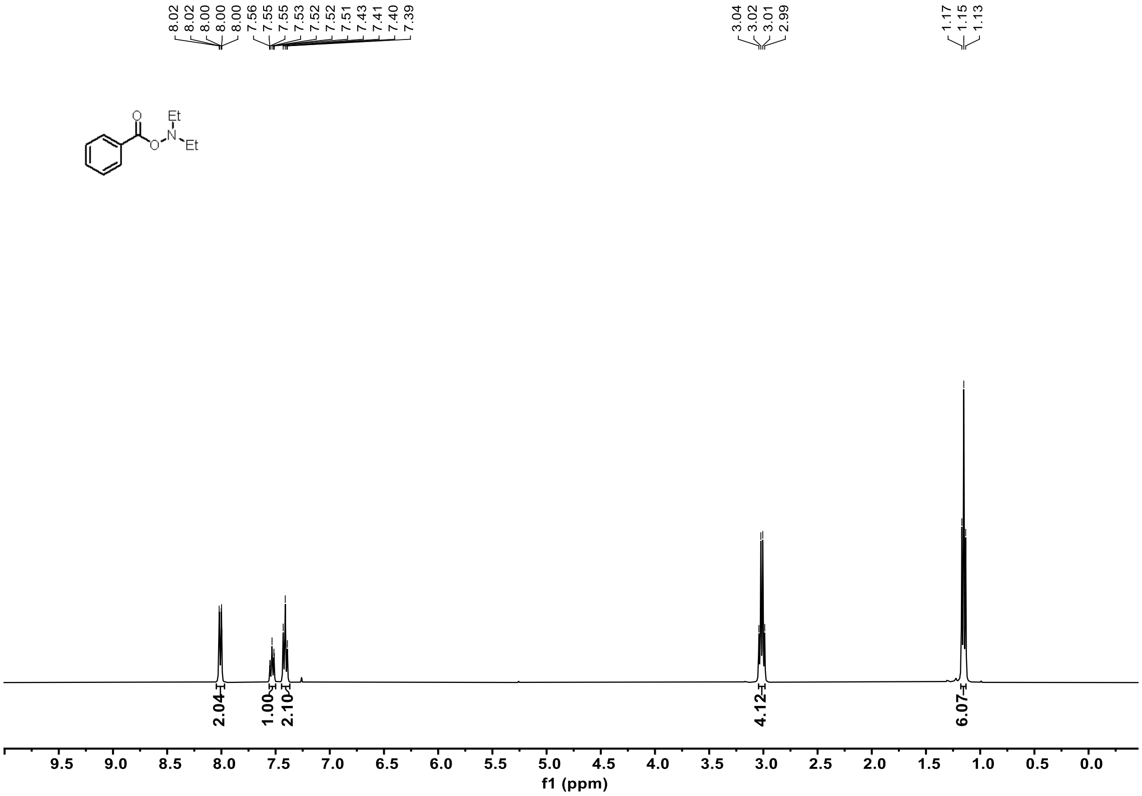

^{1}**H NMR** (400 MHz, $CDCl_3$) spectra of **2j**

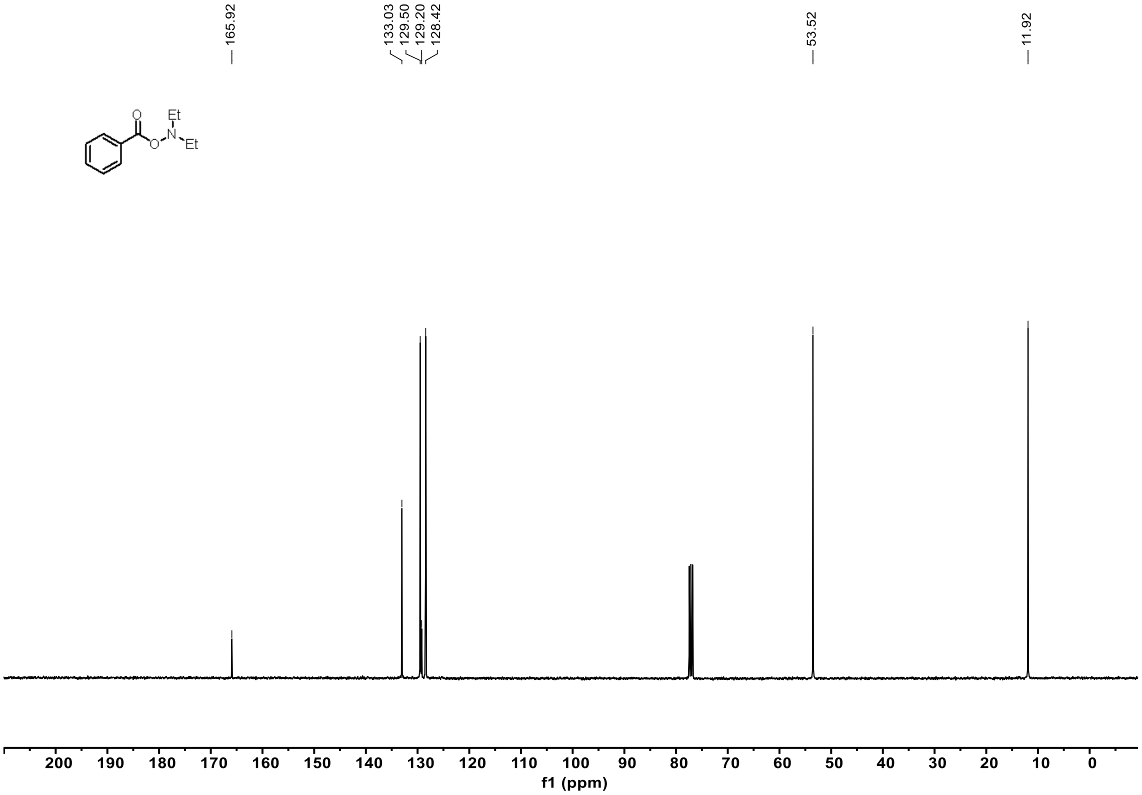

^{13}**C NMR** (101 MHz, $CDCl_3$) spectra of **2j**

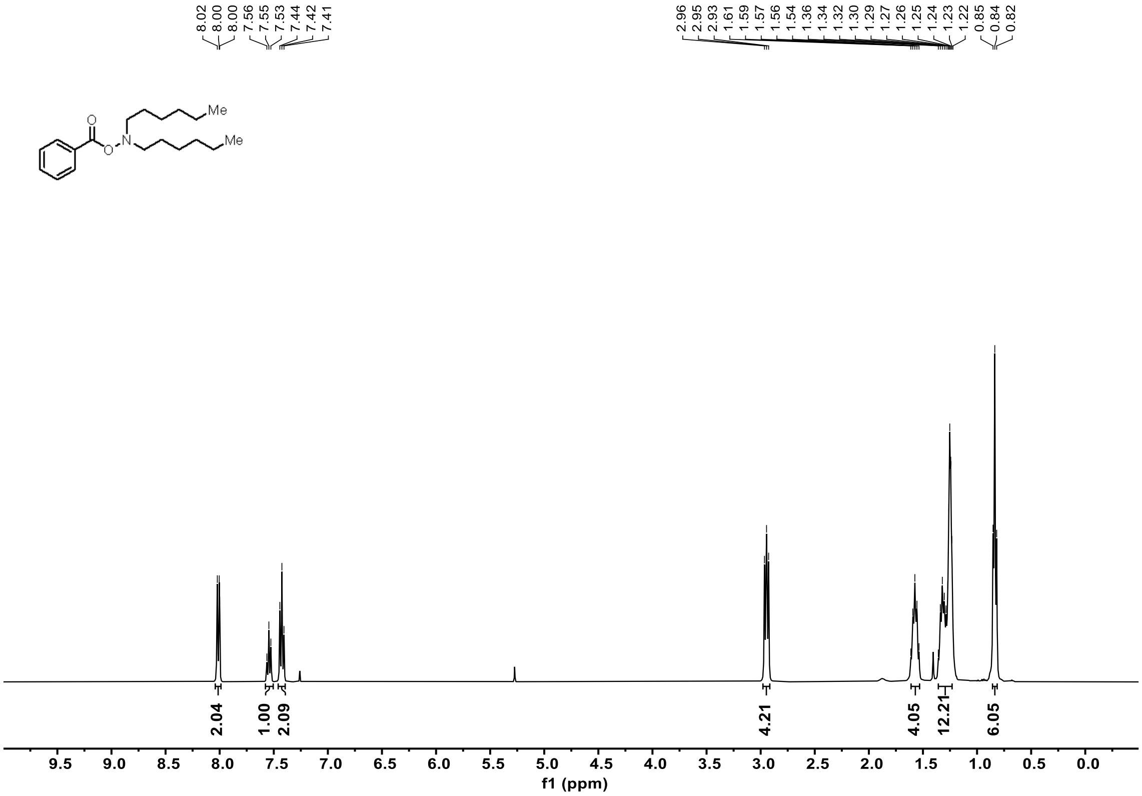

^{1}H NMR (400 MHz, $CDCl_3$) spectra of **2k**

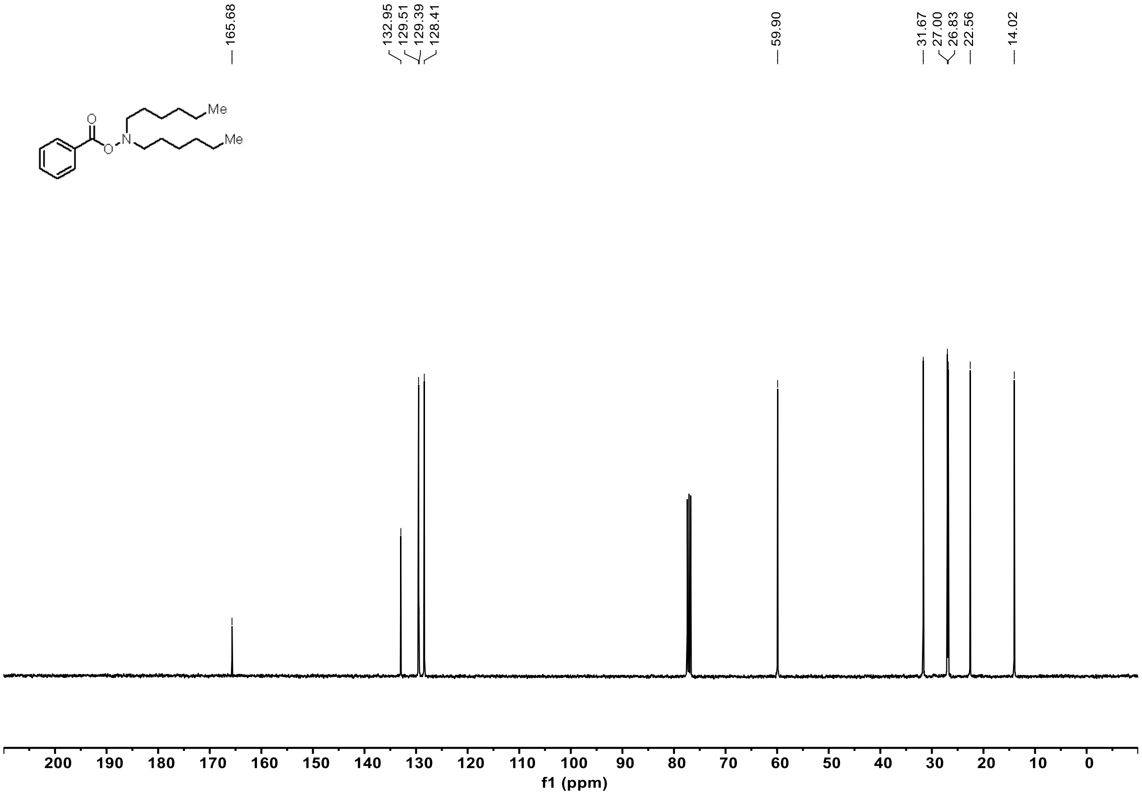

^{13}C NMR (101 MHz, $CDCl_3$) spectra of **2k**

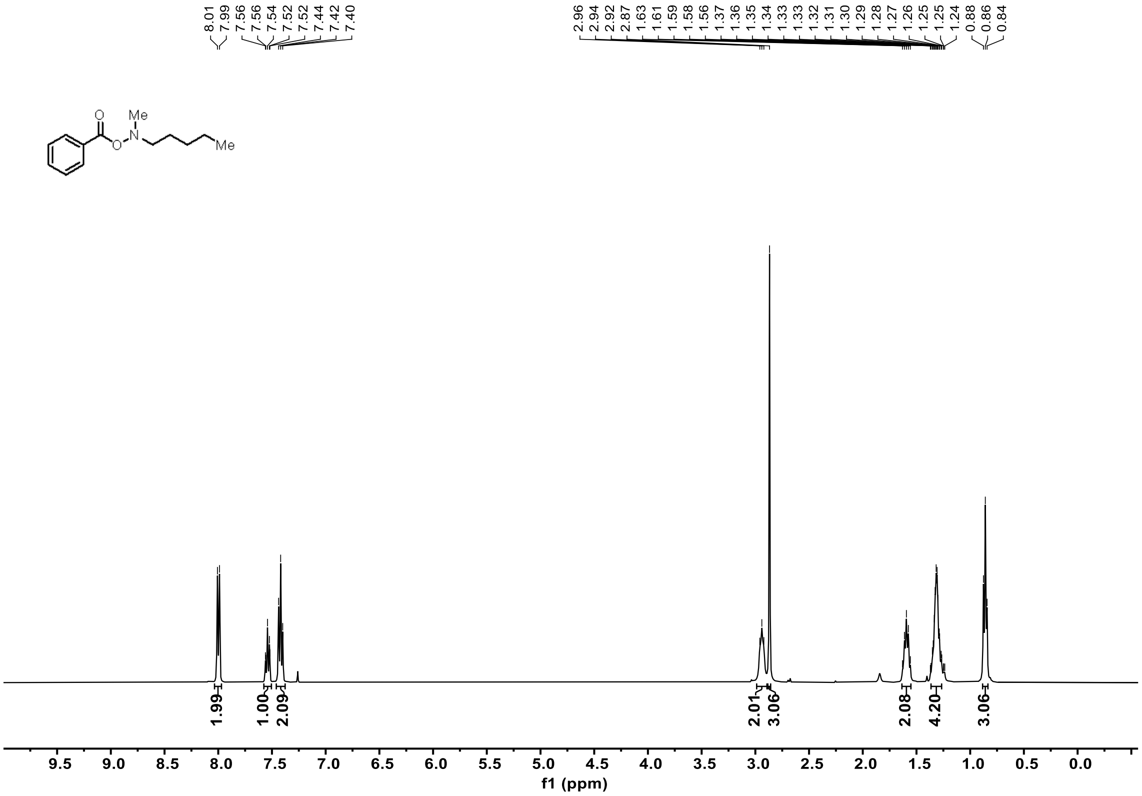

^{1}H NMR (400 MHz, $CDCl_3$) spectra of **2l**

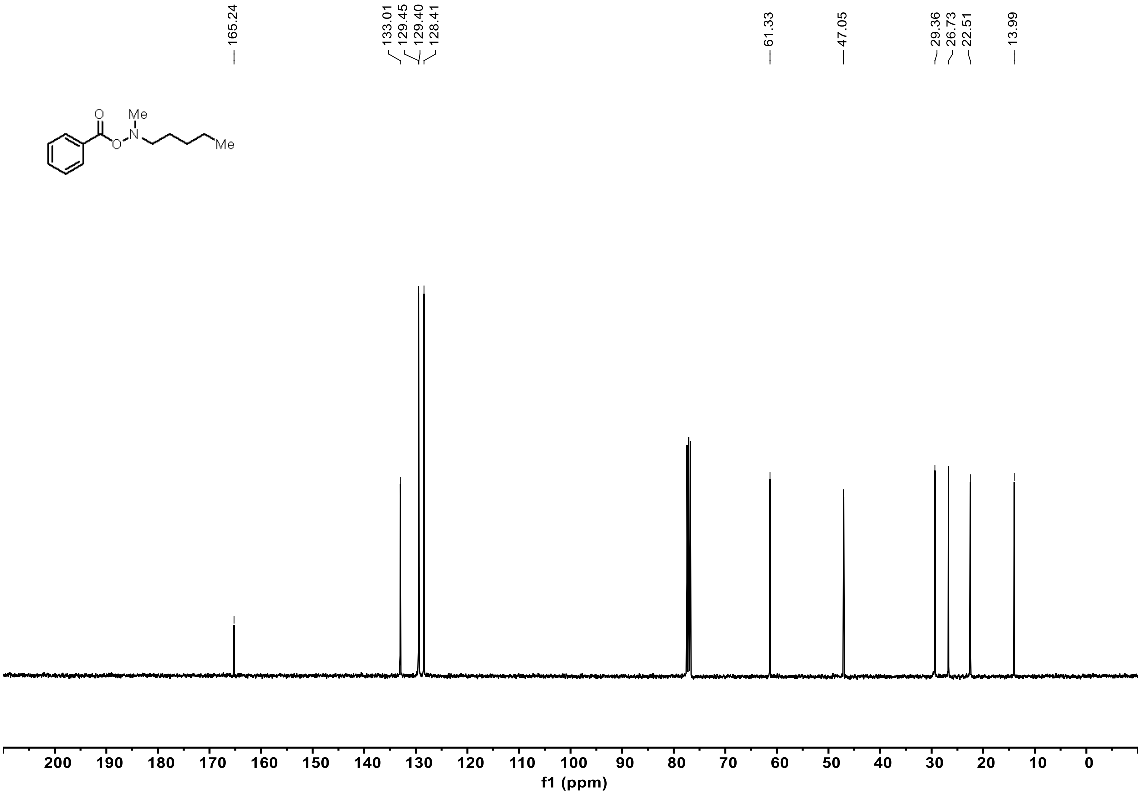

^{13}C NMR (101 MHz, $CDCl_3$) spectra of **2l**

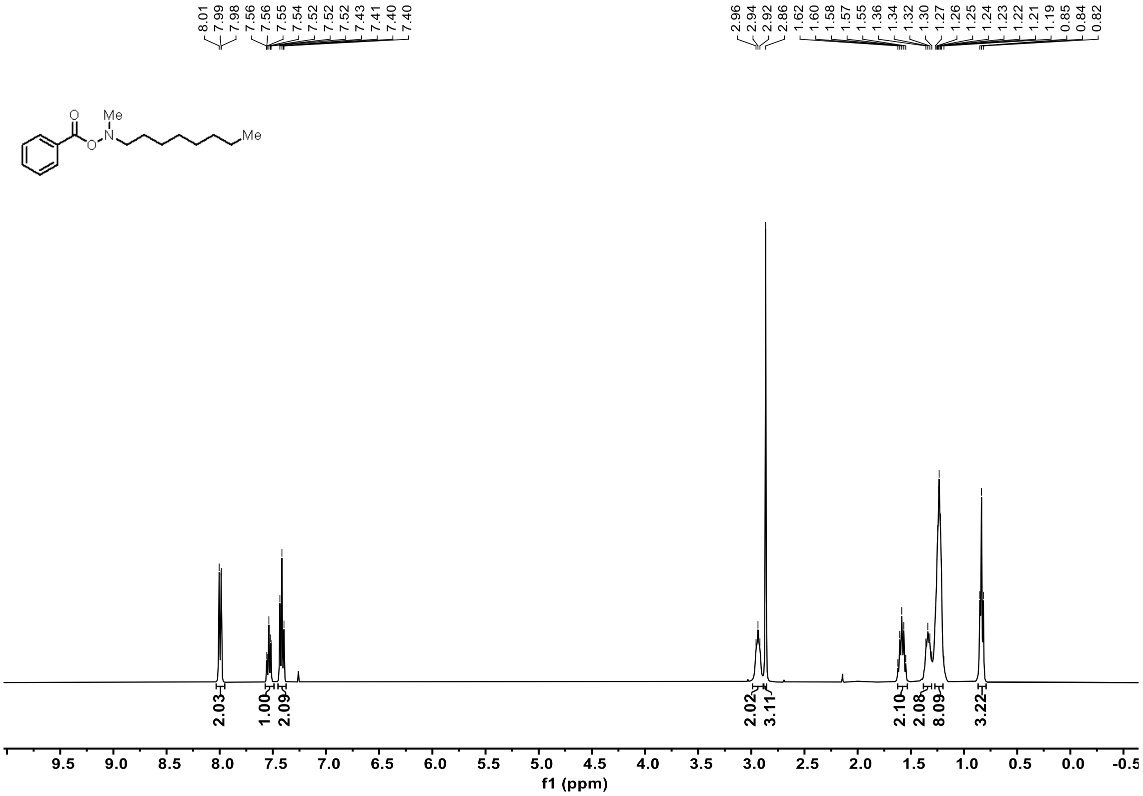

^{1}H NMR (400 MHz, $CDCl_3$) spectra of **2m**

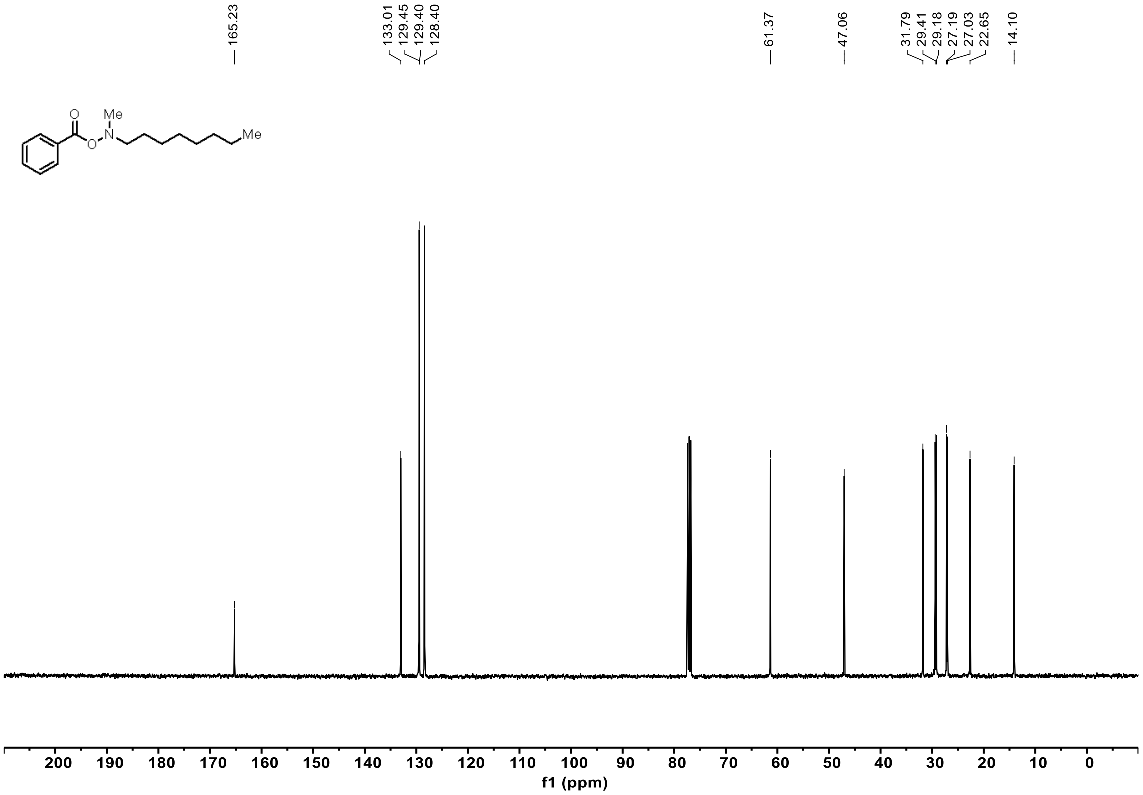

^{13}C NMR (101 MHz, $CDCl_3$) spectra of **2m**

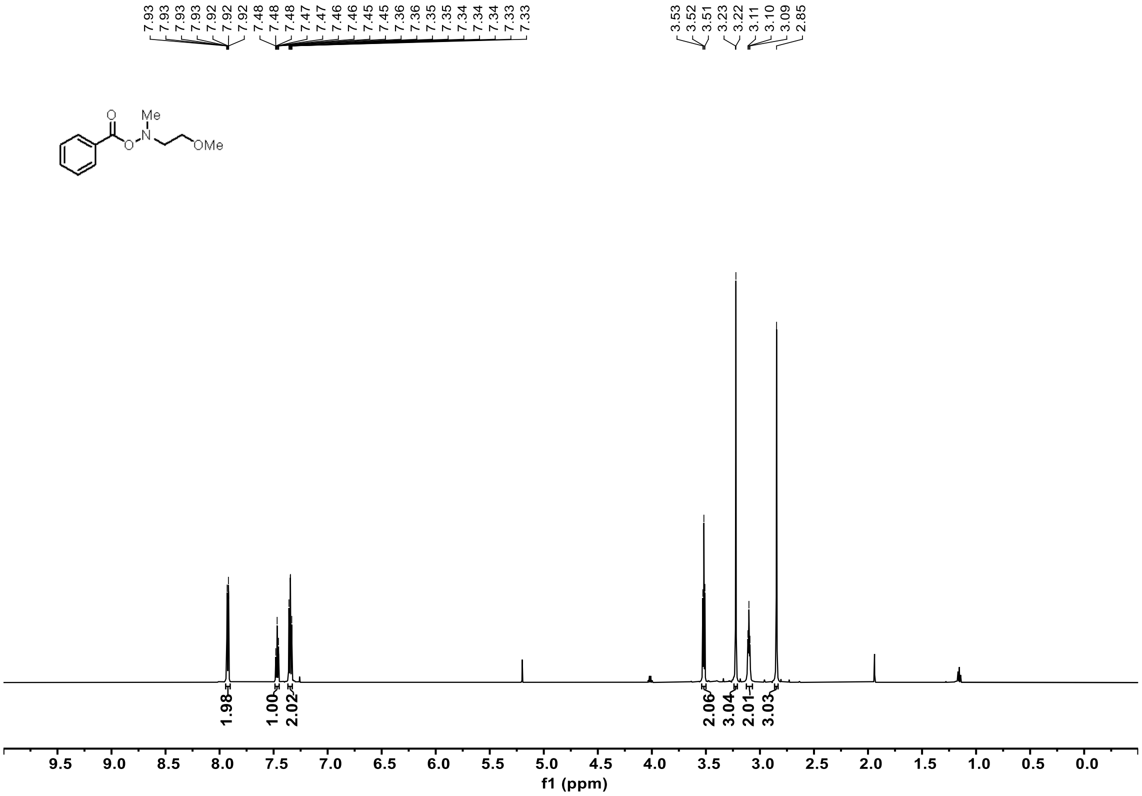

^{1}H NMR (600 MHz, $CDCl_3$) spectra of **2n**

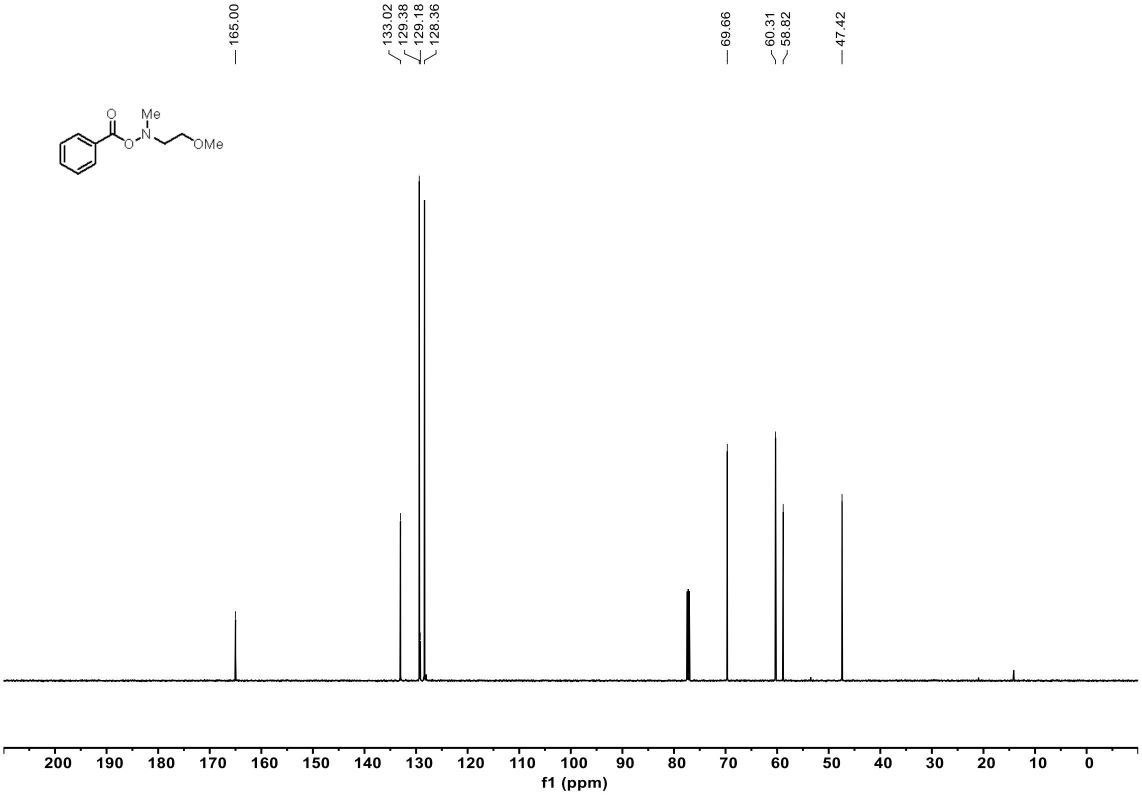

^{13}C NMR (151 MHz, $CDCl_3$) spectra of **2n**

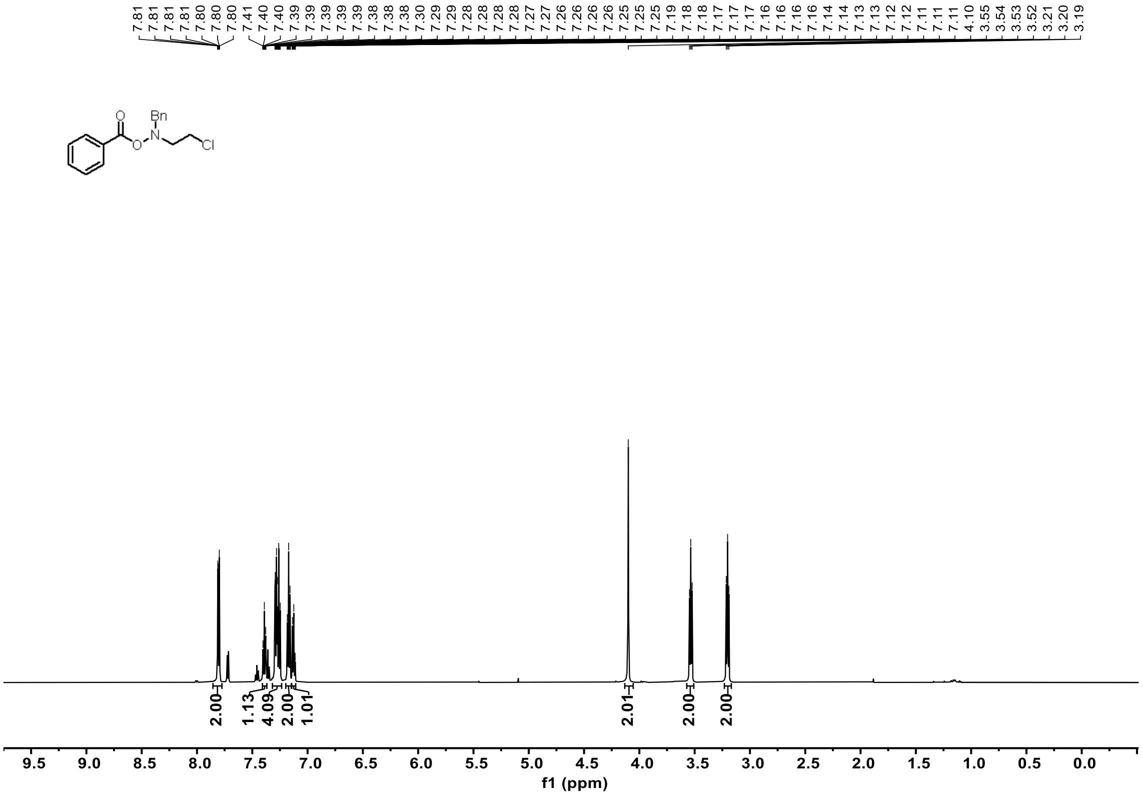

^{1}H NMR (600 MHz, $CDCl_3$) spectra of **2p**

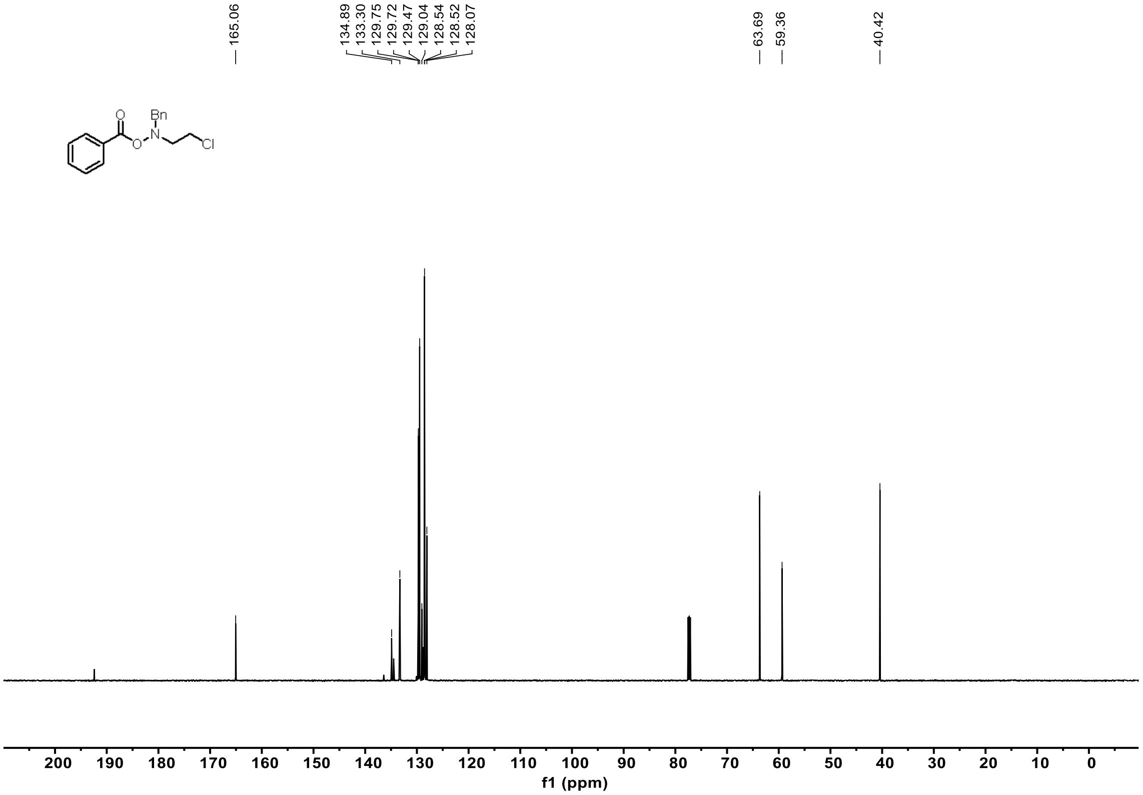

^{13}C NMR (151 MHz, $CDCl_3$) spectra of **2p**

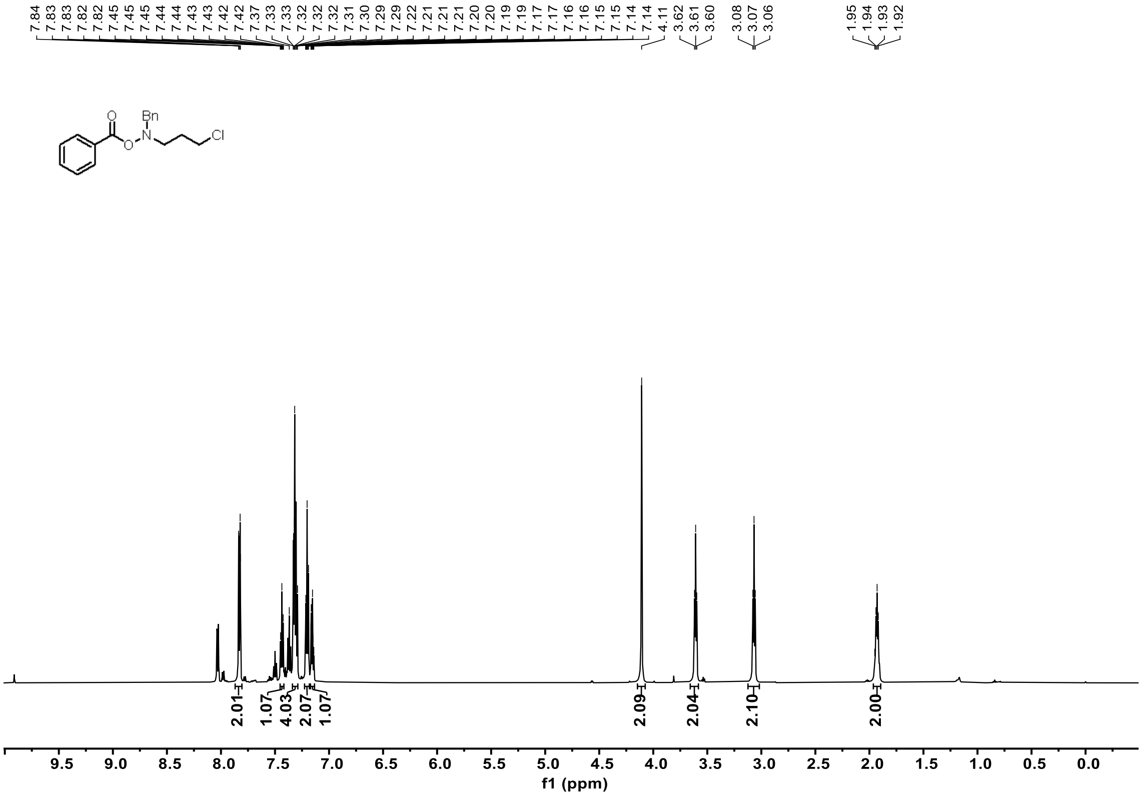

^{1}H NMR (600 MHz, $CDCl_3$) spectra of **2q**

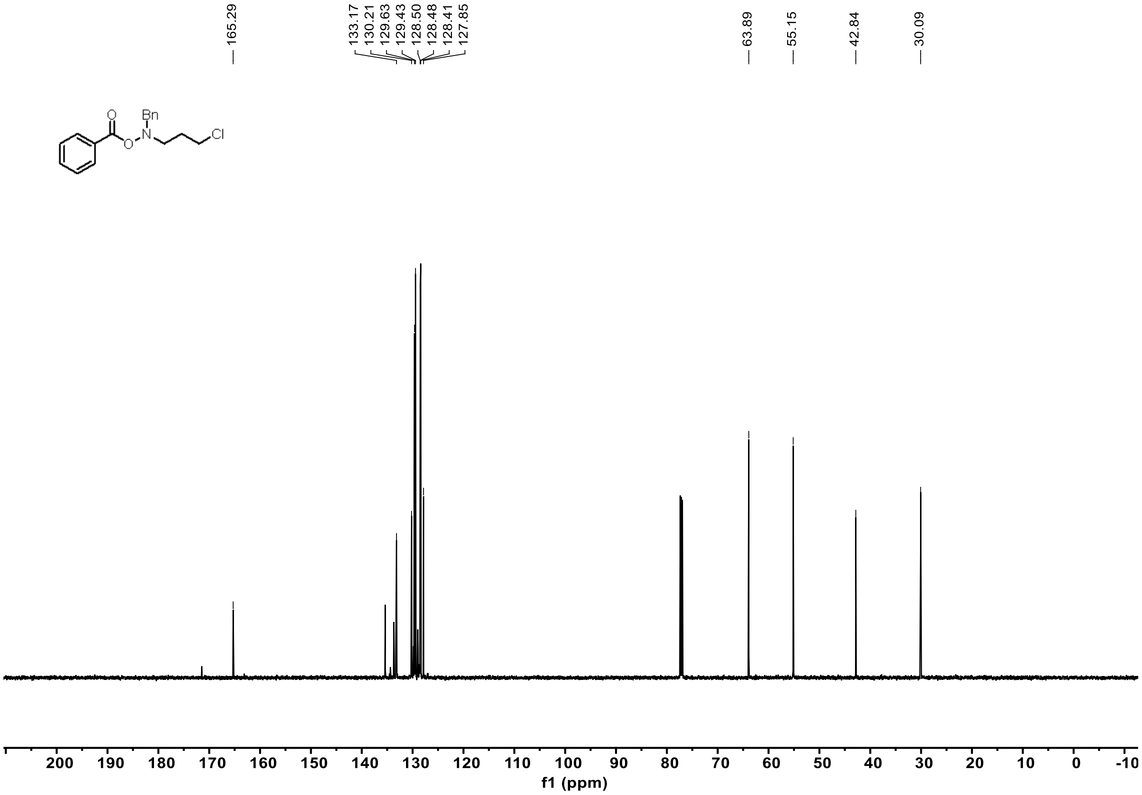

^{13}C NMR (151 MHz, $CDCl_3$) spectra of **2q**

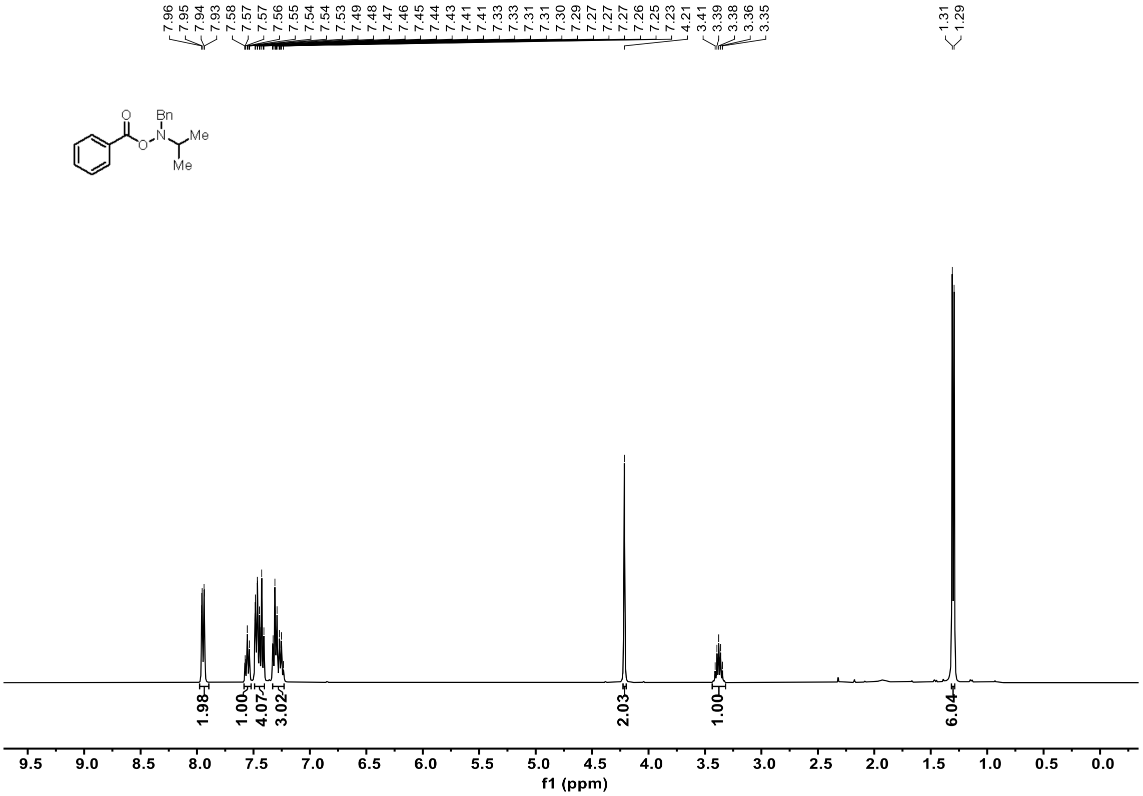

^{1}H NMR (400 MHz, $CDCl_3$) spectra of **2r**

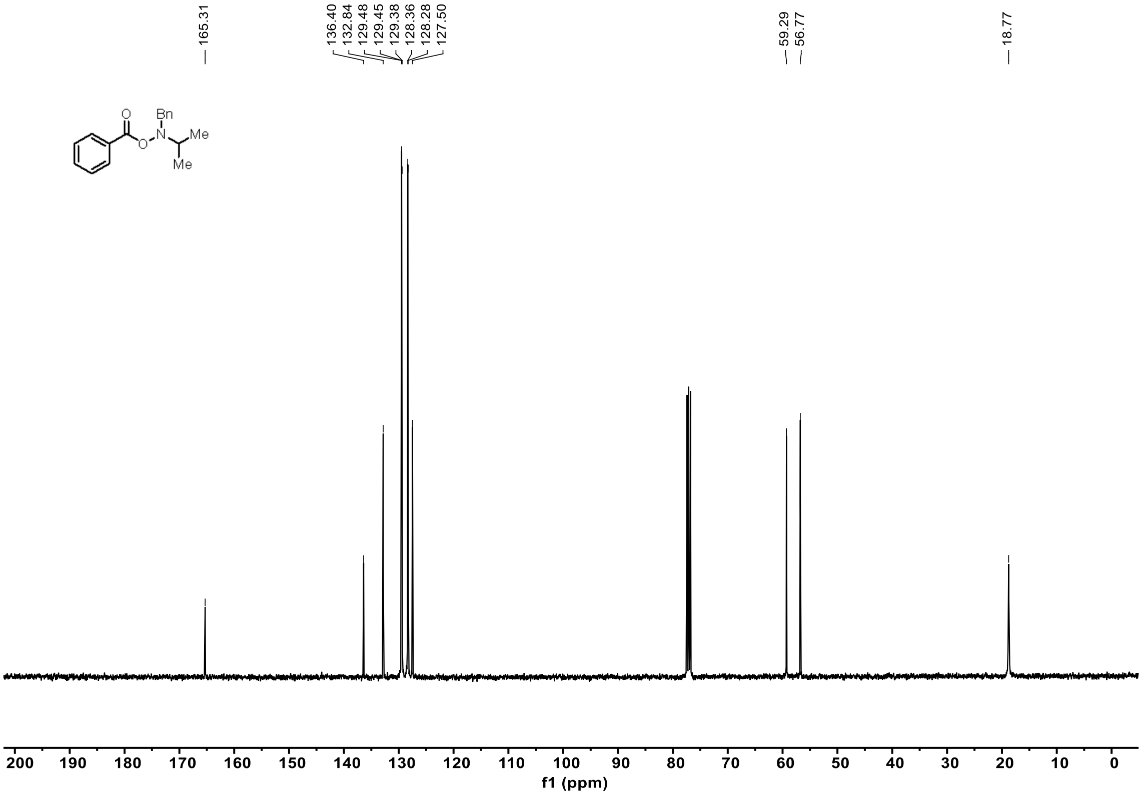

^{13}C NMR (101 MHz, $CDCl_3$) spectra of **2r**

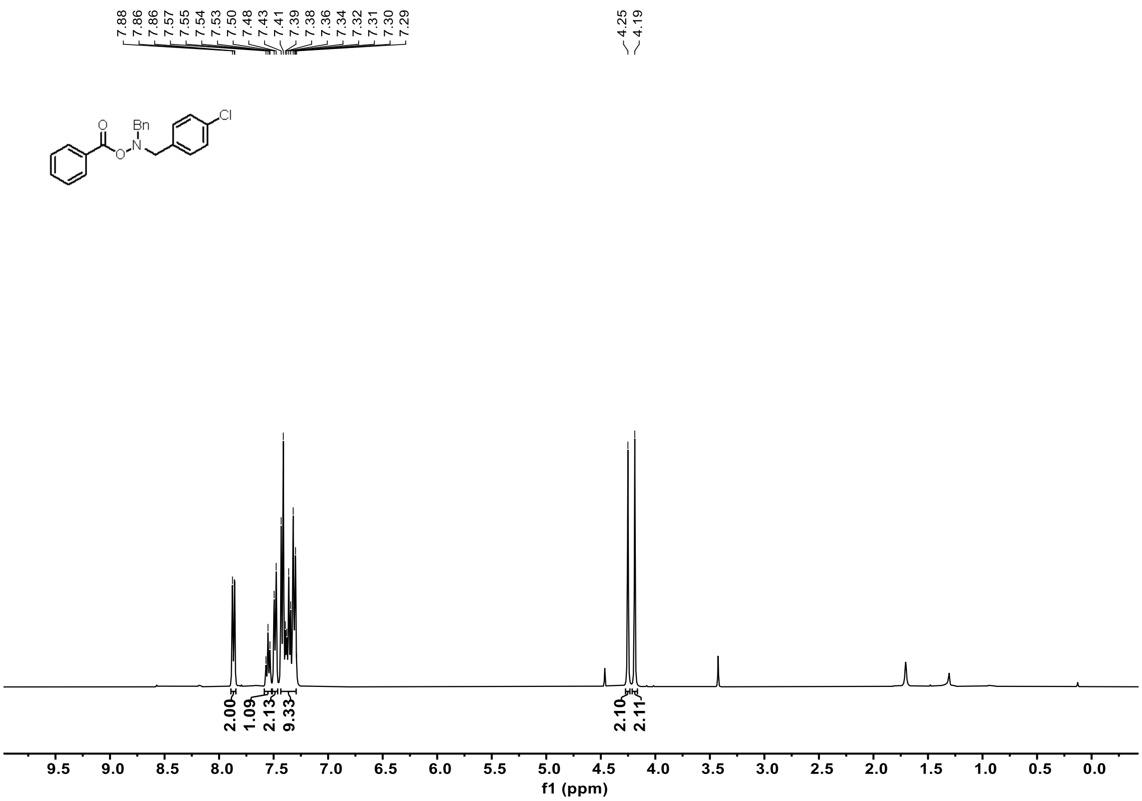

^{1}H NMR (400 MHz, $CDCl_3$) spectra of **2u**

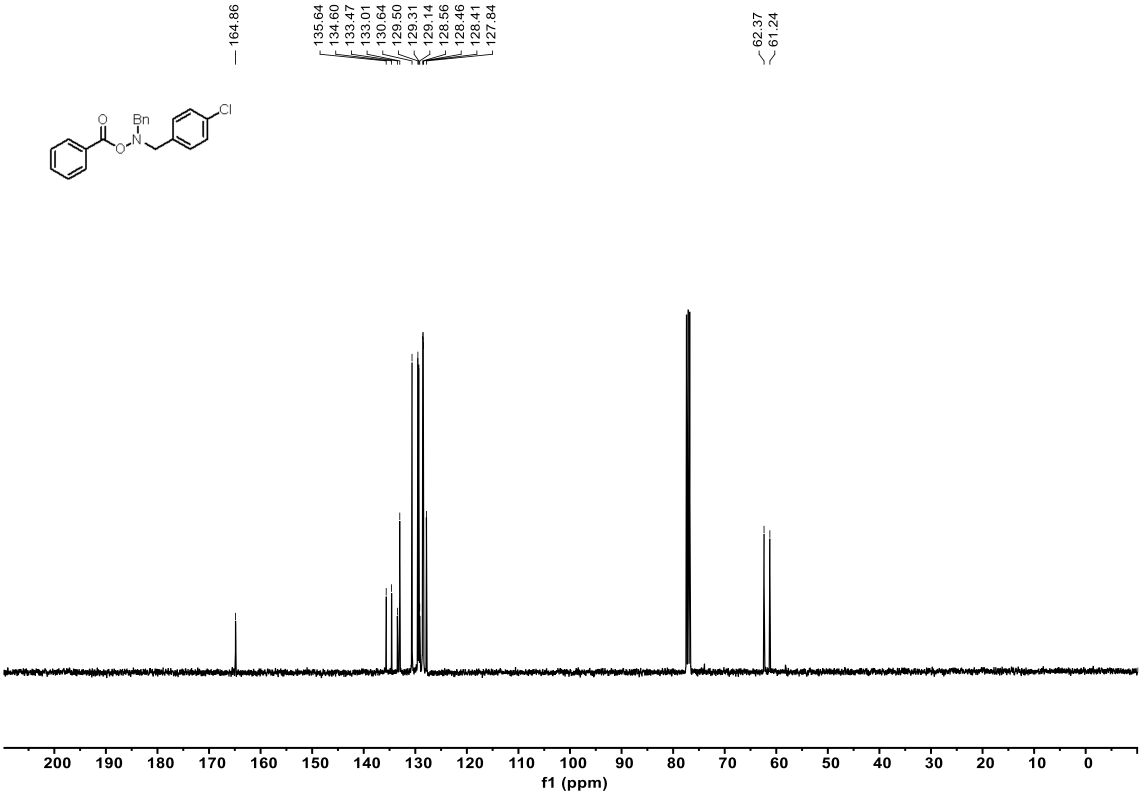

^{13}C NMR (101 MHz, $CDCl_3$) spectra of **2u**

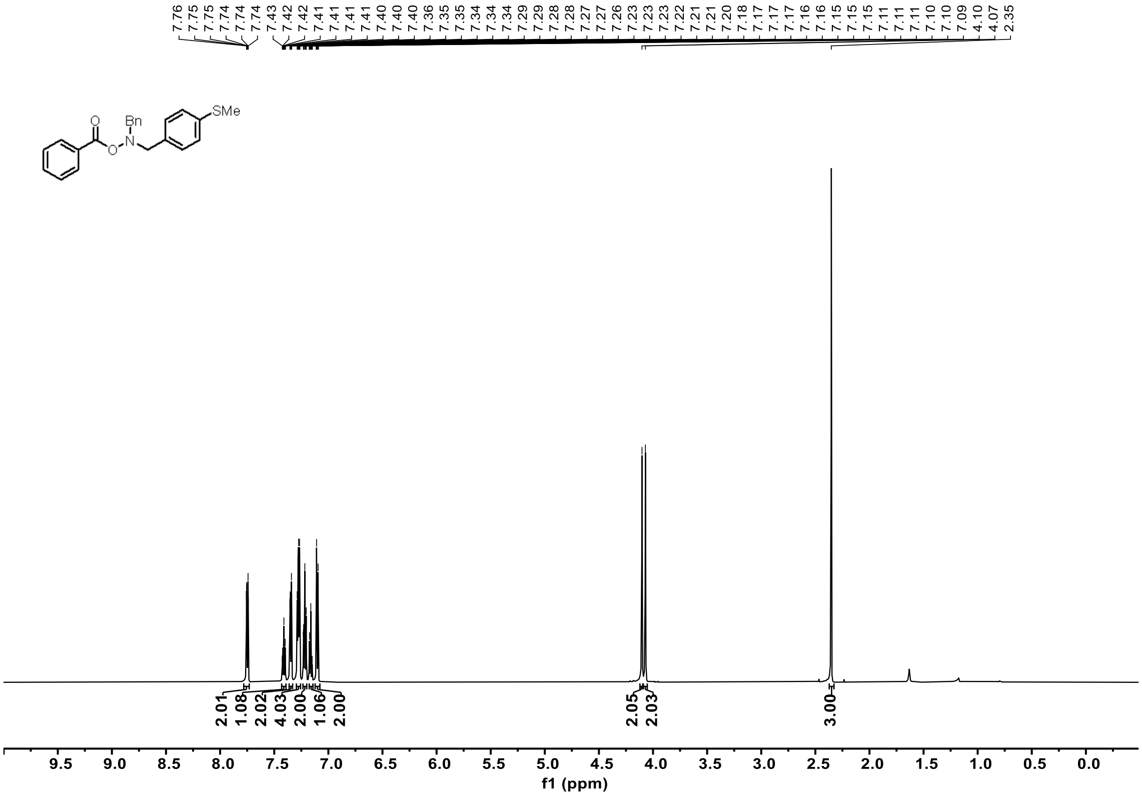

^{1}H NMR (600 MHz, $CDCl_3$) spectra of **2v**

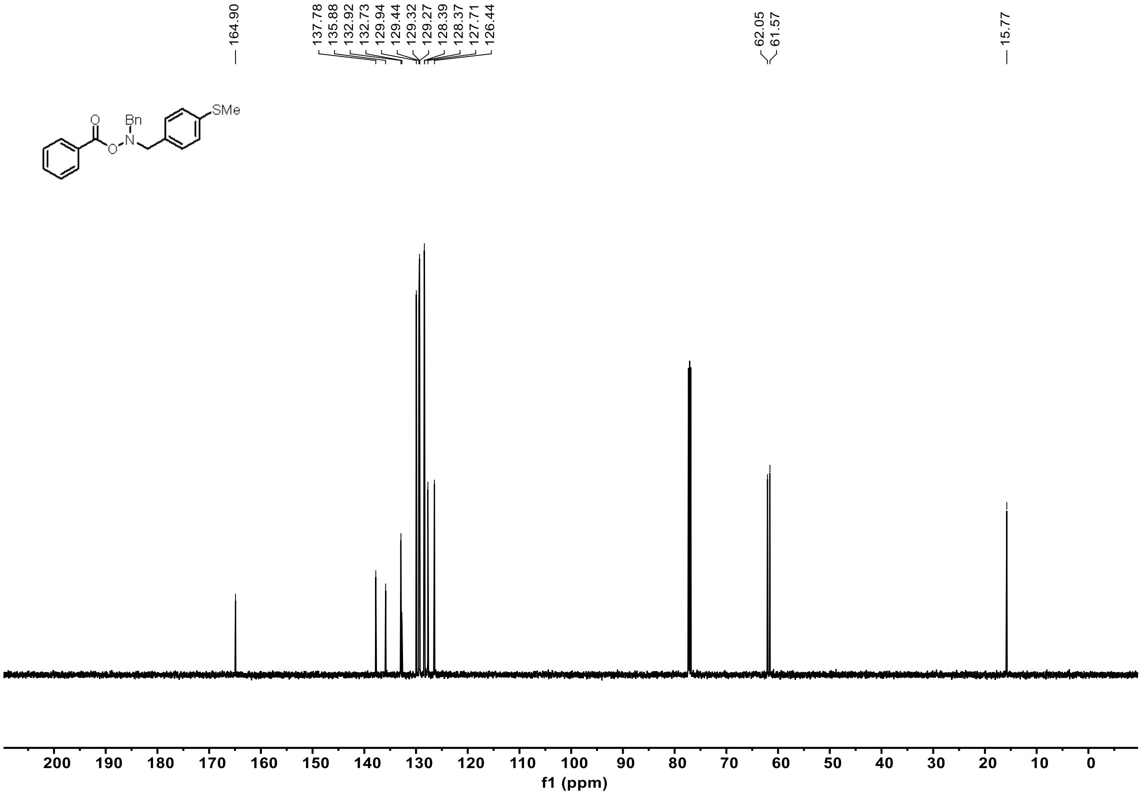

^{13}C NMR (151 MHz, $CDCl_3$) spectra of **2v**

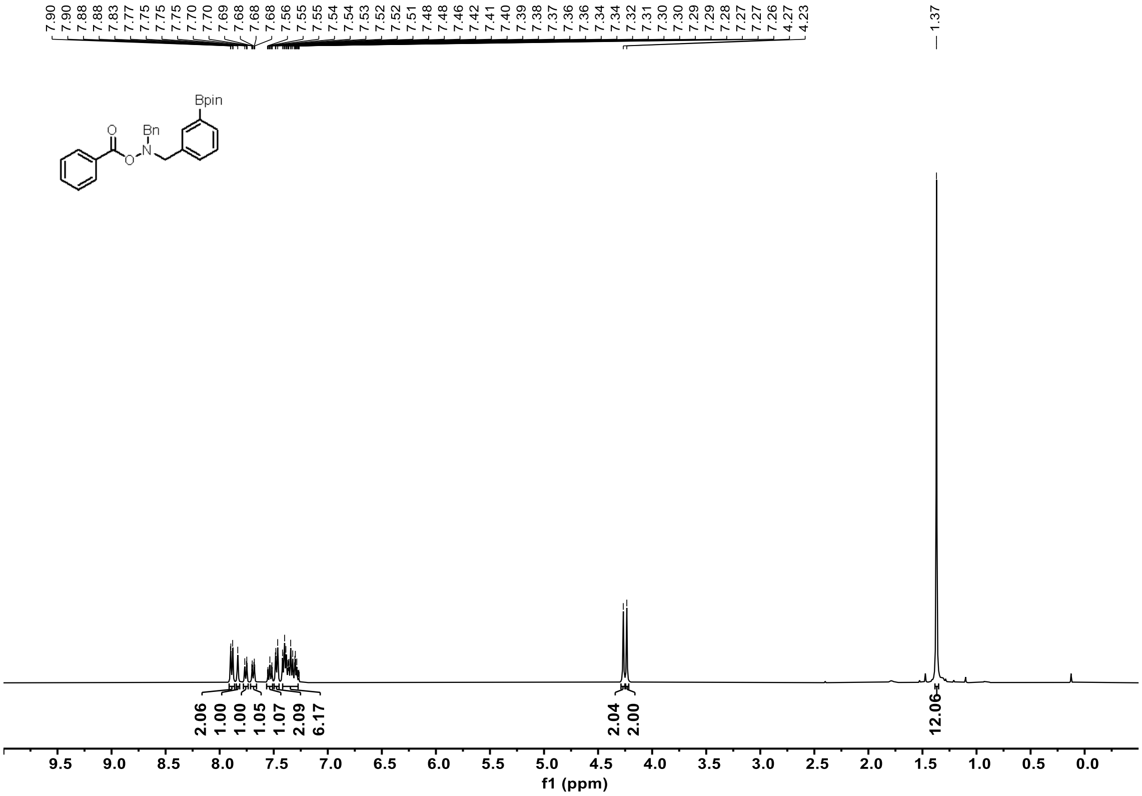

^{1}H NMR (400 MHz, $CDCl_3$) spectra of **2x**

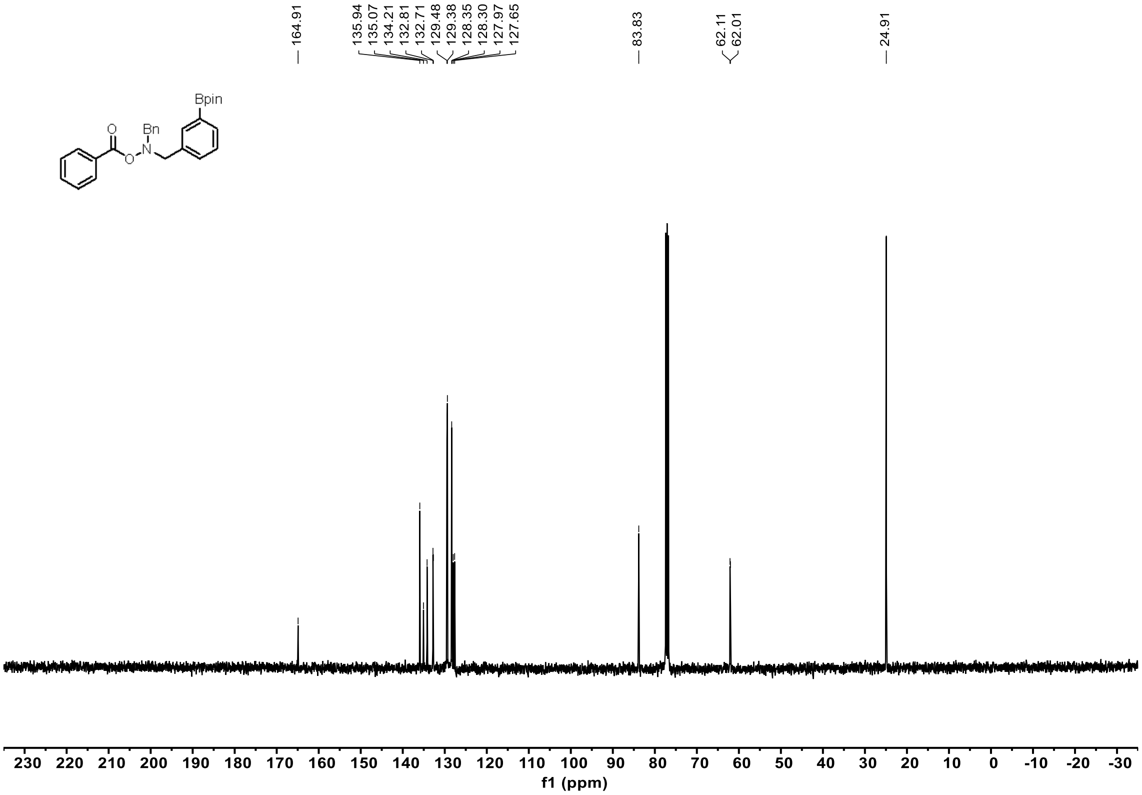

^{13}C NMR (101 MHz, $CDCl_3$) spectra of **2x**

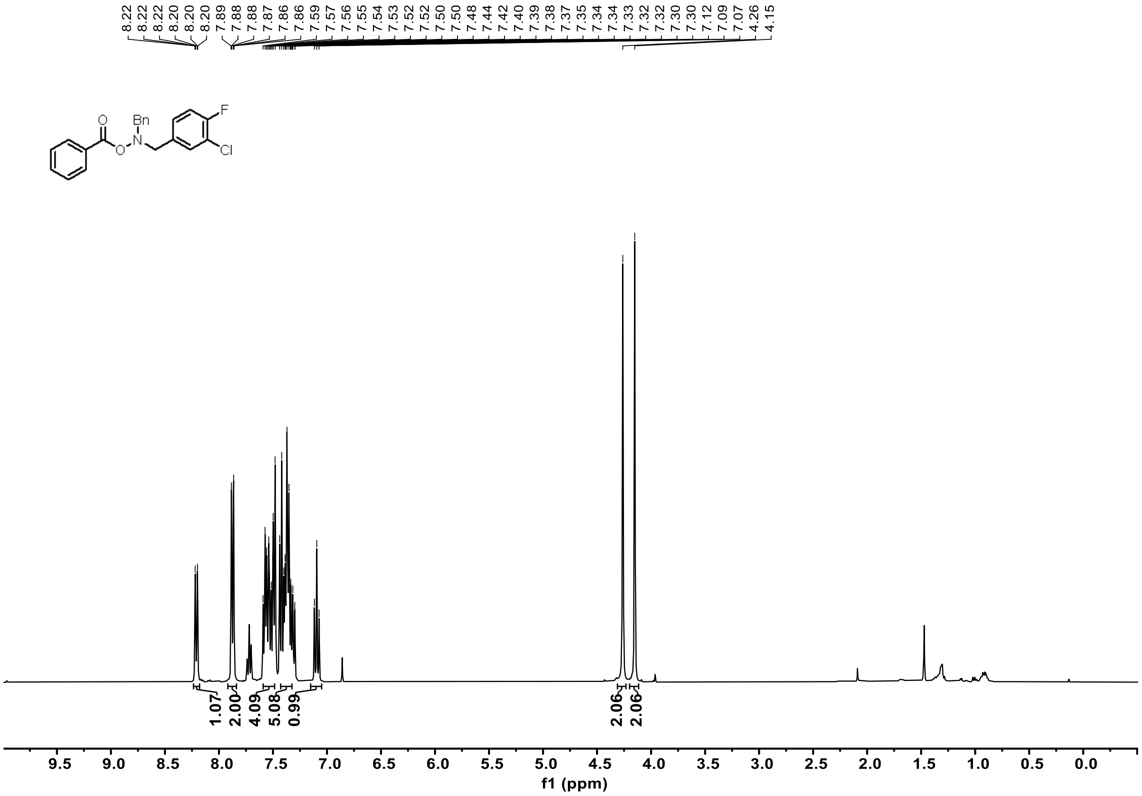

^{1}H NMR (400 MHz, $CDCl_3$) spectra of **2y**

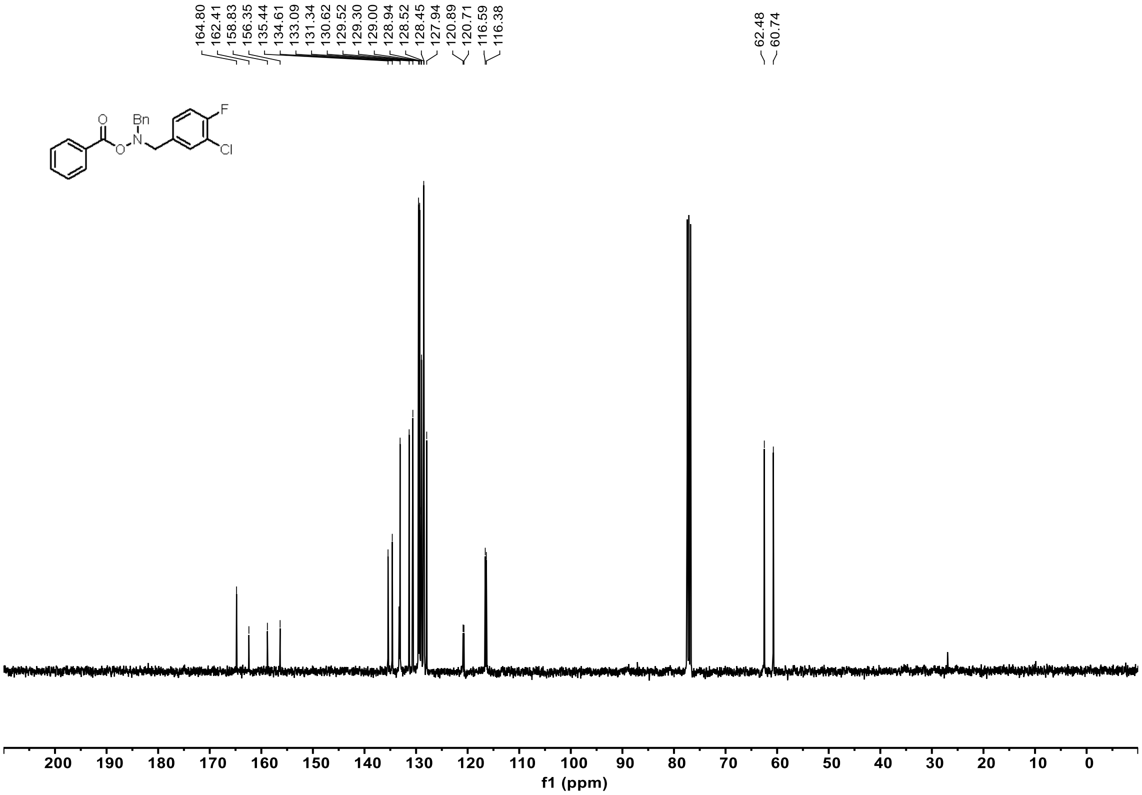

^{13}C NMR (101 MHz, $CDCl_3$) spectra of **2y**

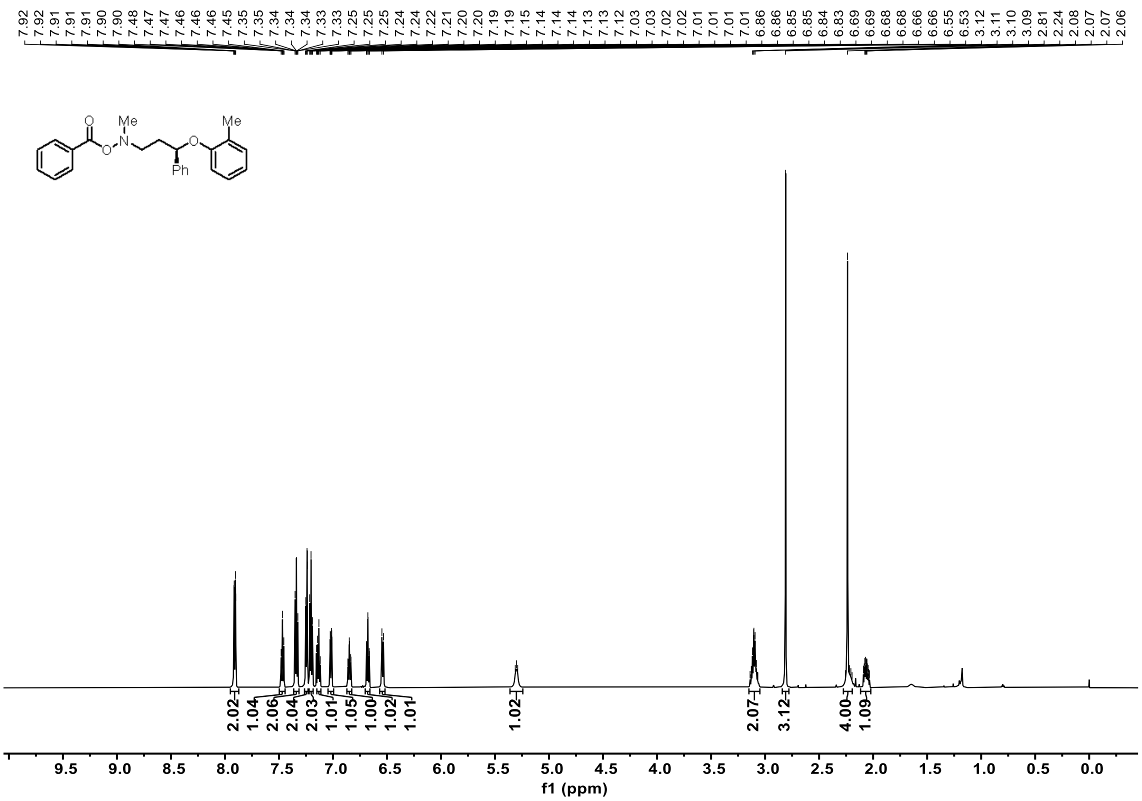

^{1}H NMR (600 MHz, $CDCl_3$) spectra of **2z**

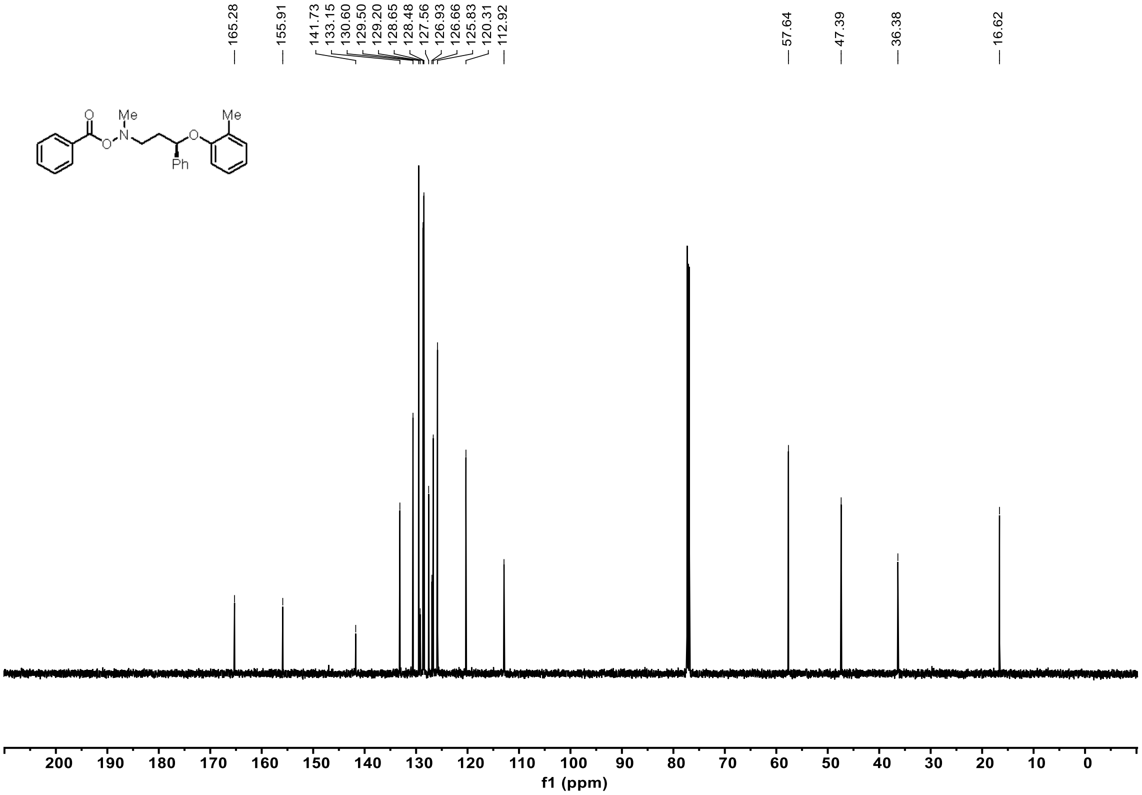

^{13}C NMR (151 MHz, $CDCl_3$) spectra of **2z**

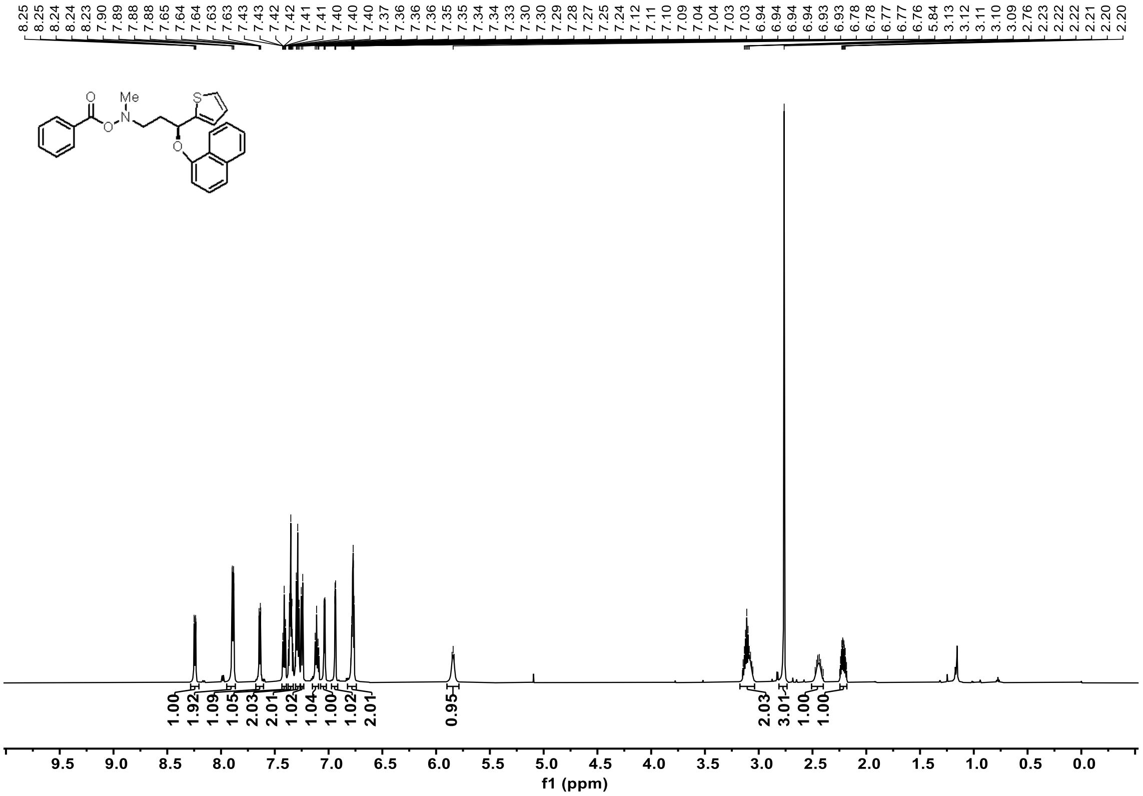

^{1}H NMR (600 MHz, $CDCl_3$) spectra of **2aa**

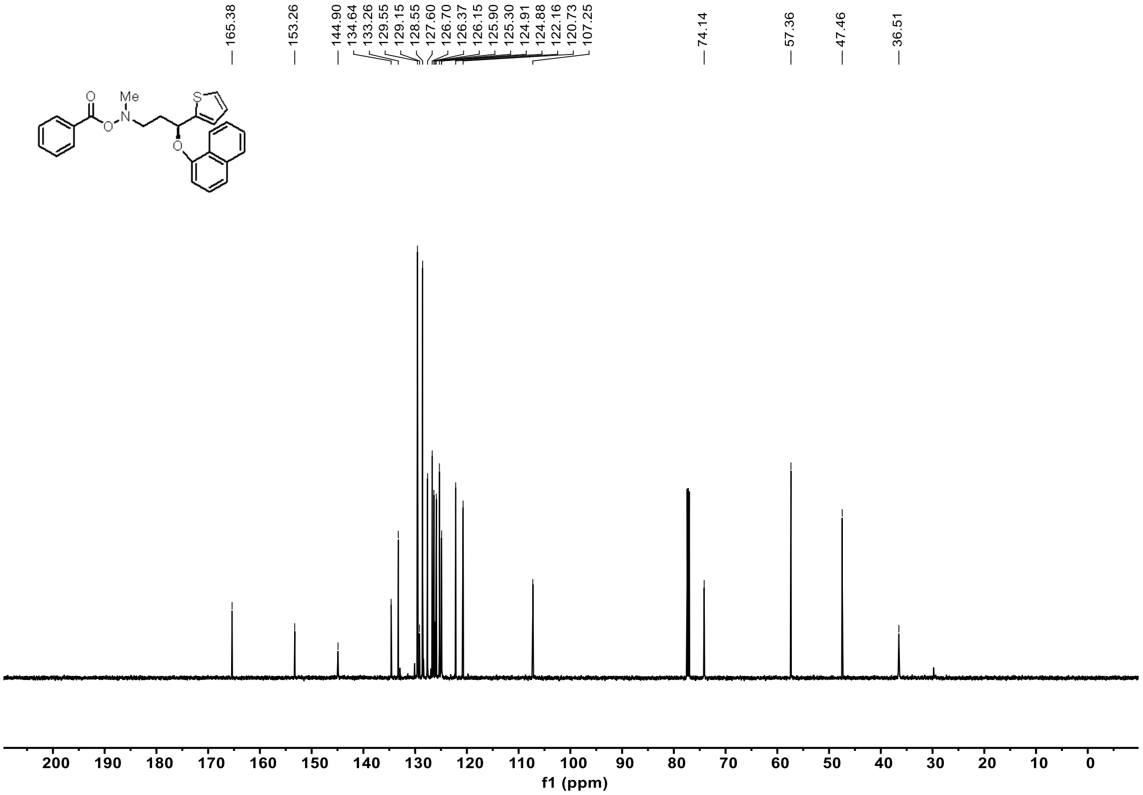

^{13}C NMR (151 MHz, $CDCl_3$) spectra of **2aa**

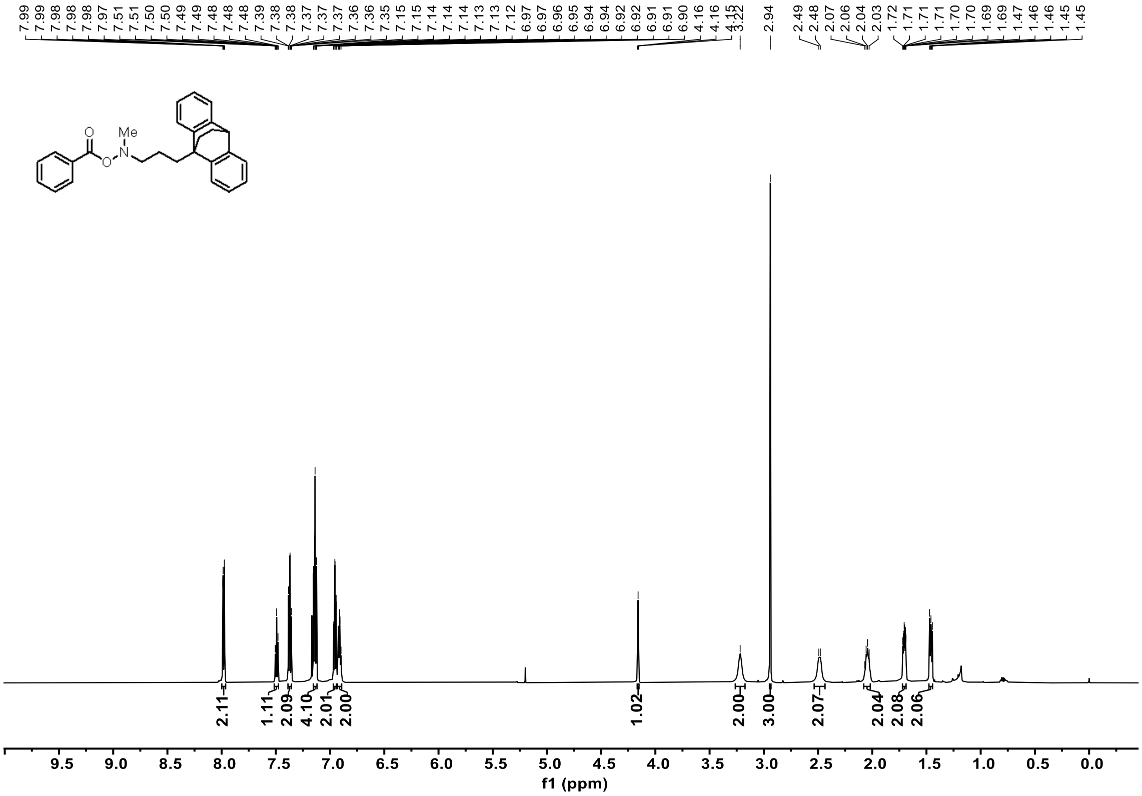

^{1}H NMR (600 MHz, $CDCl_3$) spectra of **2ab**

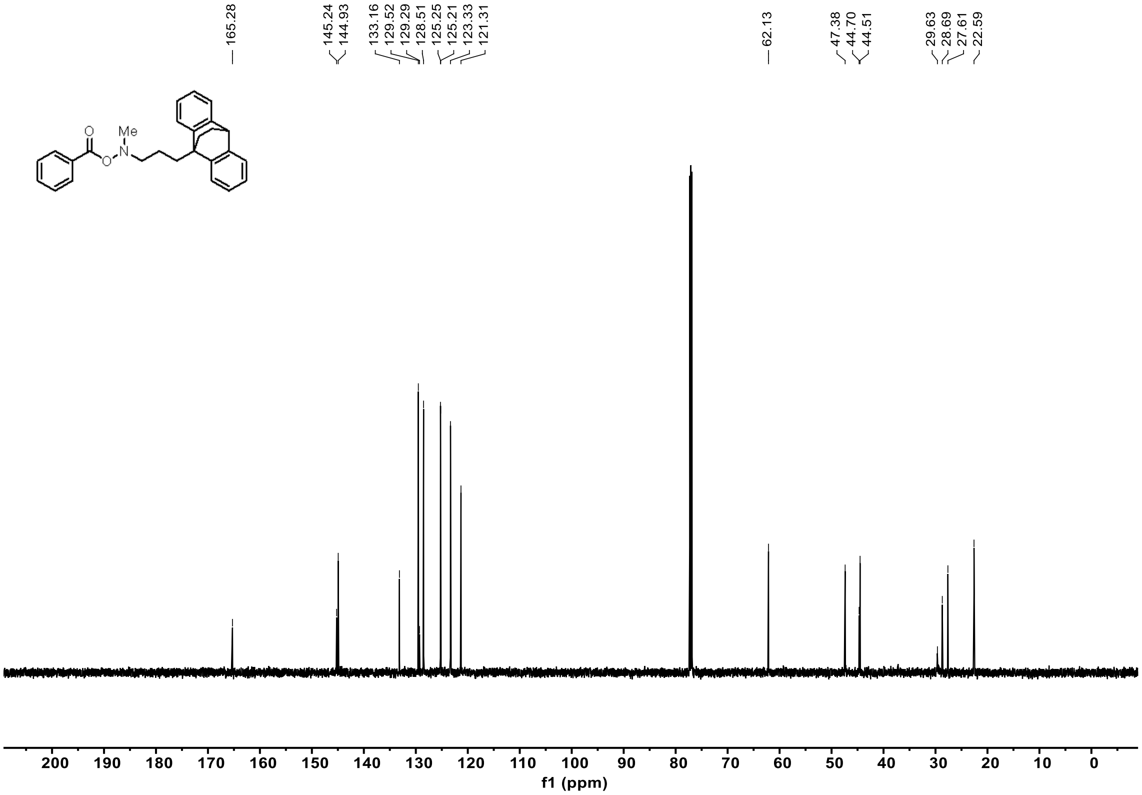

^{13}C NMR (151 MHz, $CDCl_3$) spectra of **2ab**

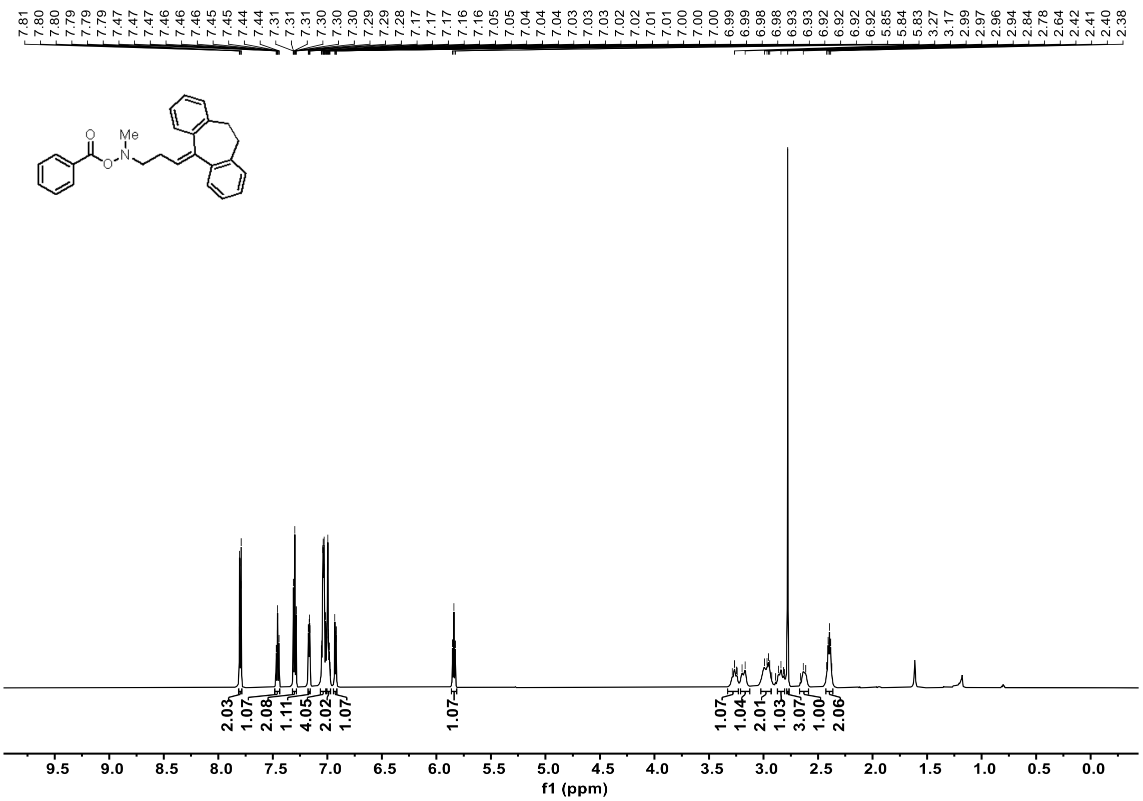

^{1}H NMR (600 MHz, $CDCl_3$) spectra of **2ac**

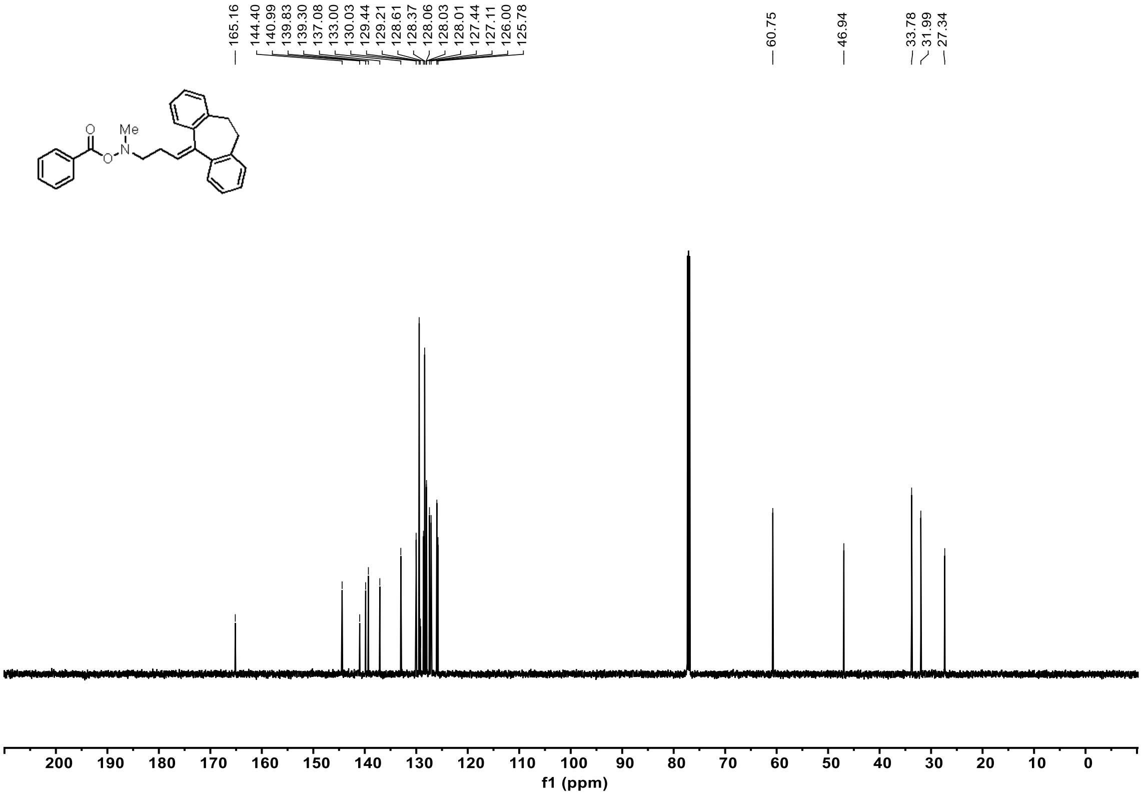

^{13}C NMR (151 MHz, $CDCl_3$) spectra of **2ac**

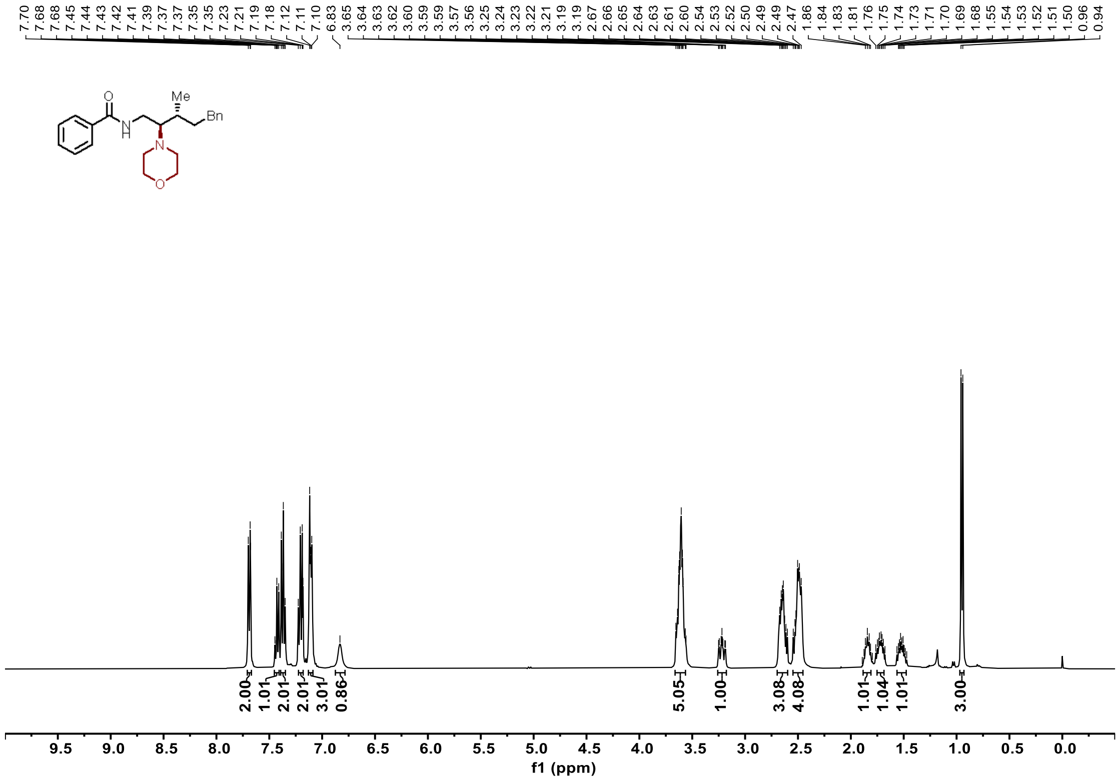

^{1}H NMR (400 MHz, $CDCl_3$) spectra of **3a**

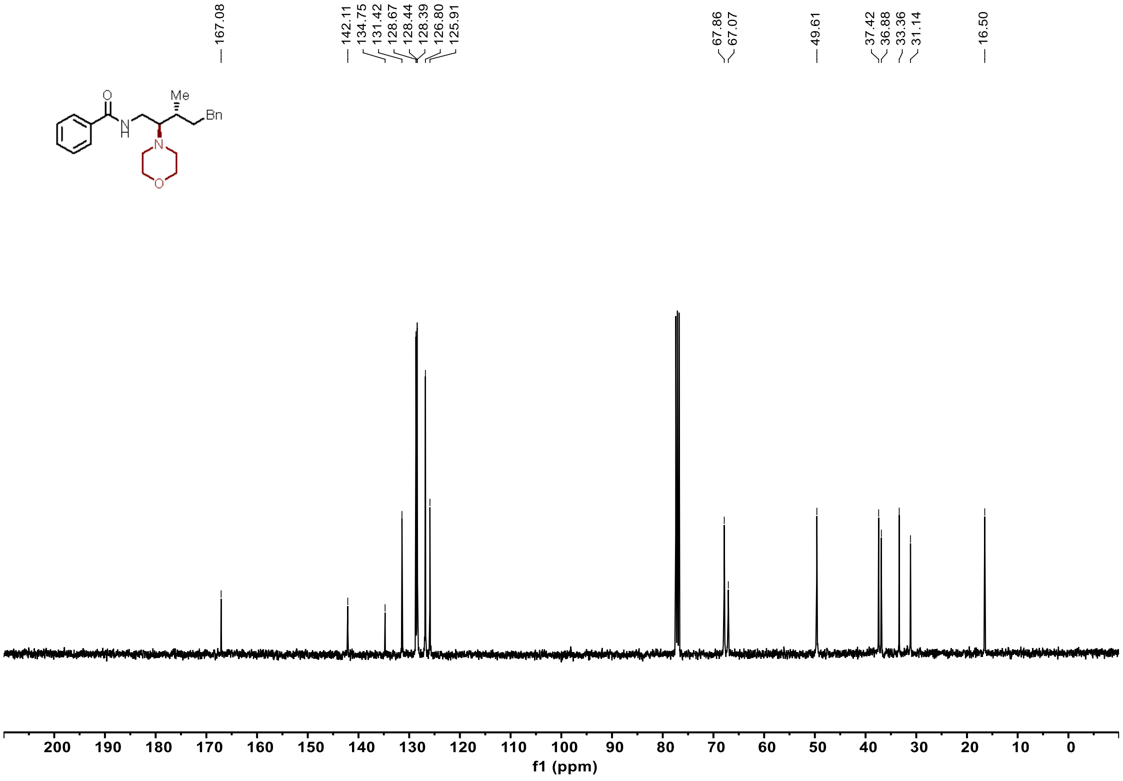

^{13}C NMR (101 MHz, $CDCl_3$) spectra of **3a**

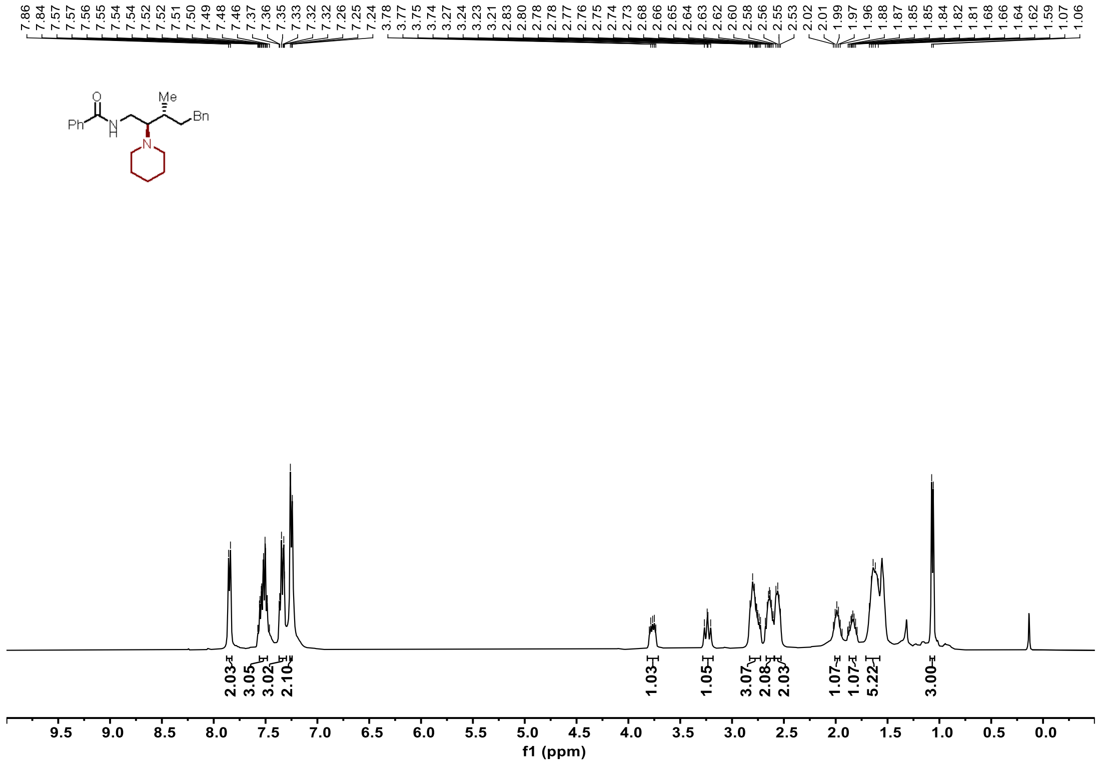

^{1}H NMR (400 MHz, $CDCl_3$) spectra of **3b**

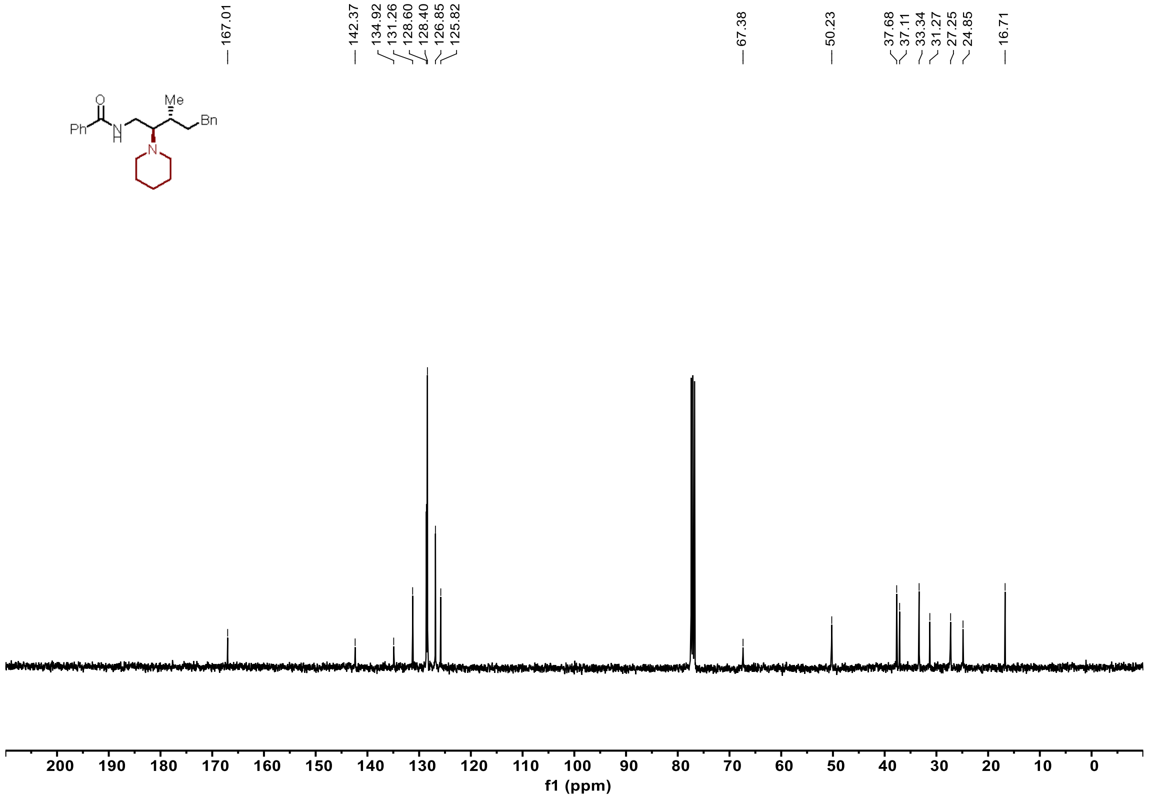

^{13}C NMR (101 MHz, $CDCl_3$) spectra of **3b**

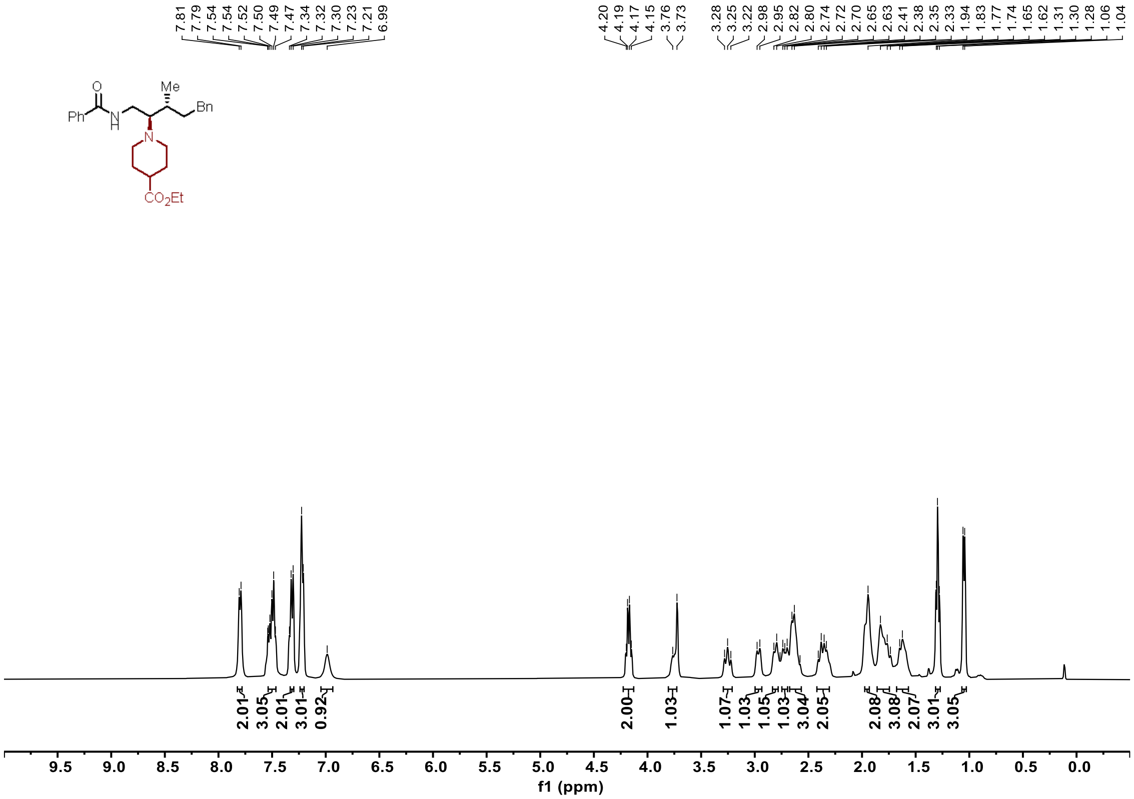

^{1}H NMR (400 MHz, CDCl$_3$) spectra of **3c**

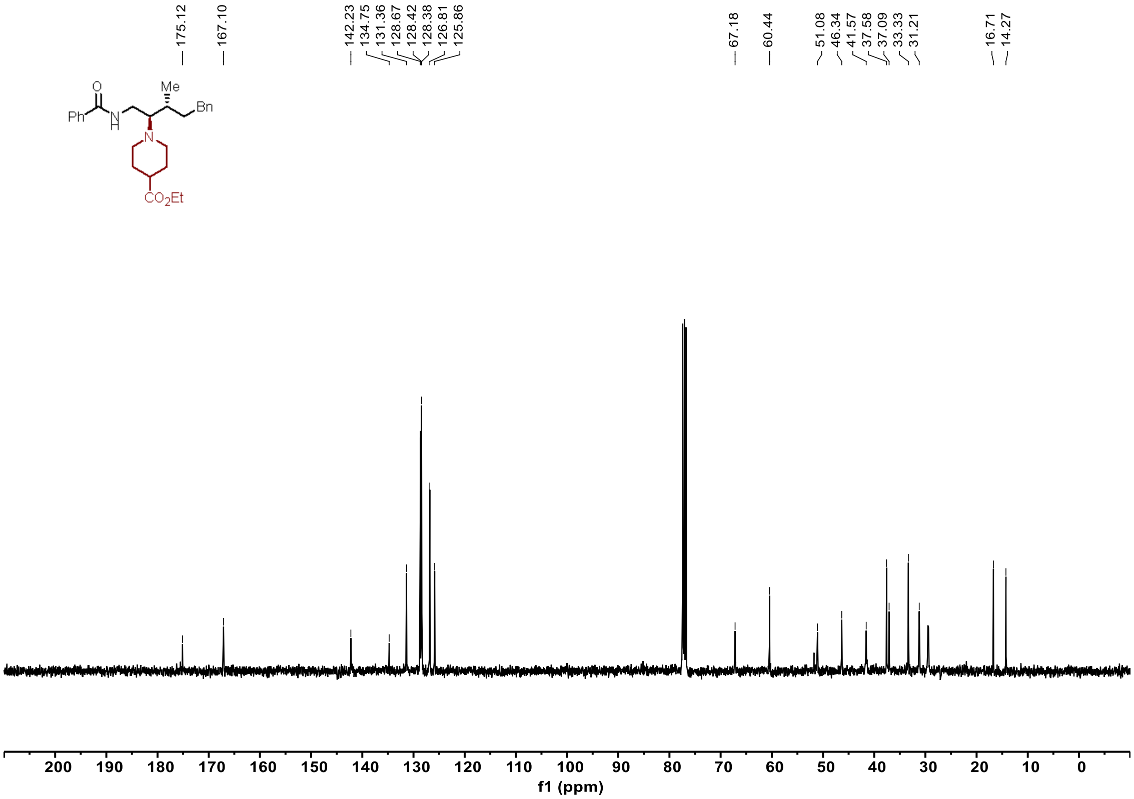

^{13}C NMR (101 MHz, CDCl$_3$) spectra of **3c**

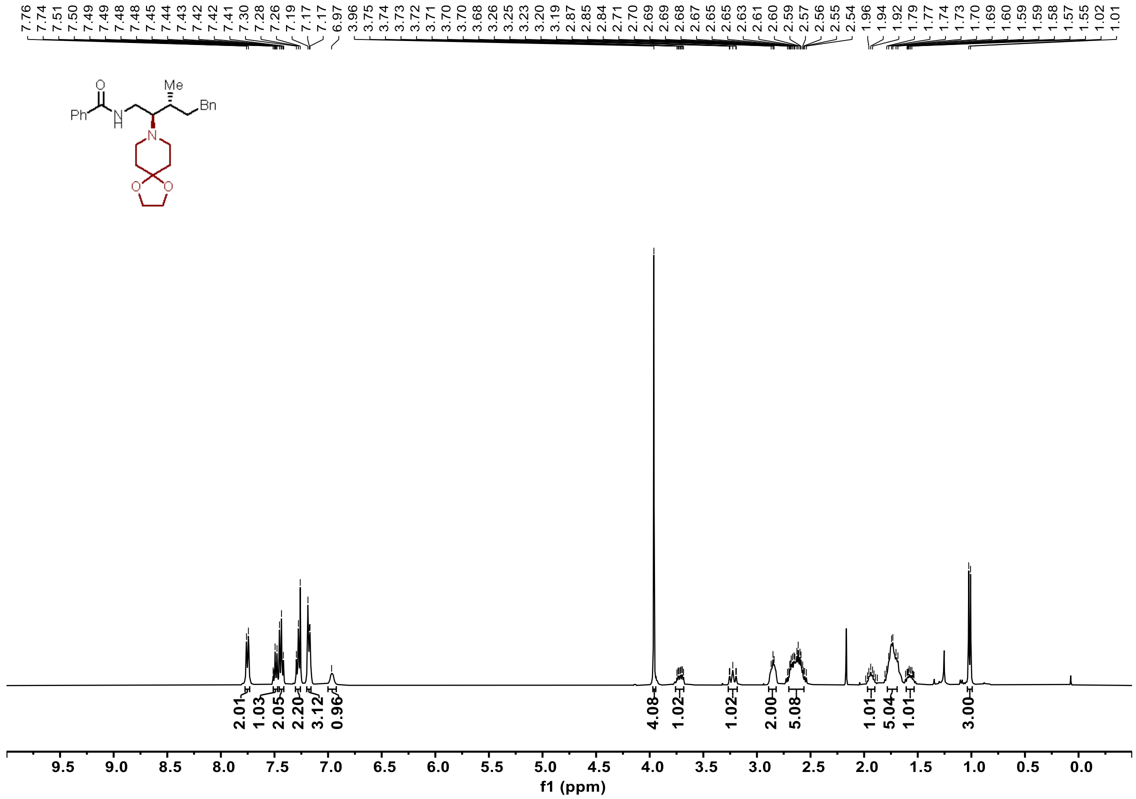

^{1}H NMR (400 MHz, $CDCl_3$) spectra of **3d**

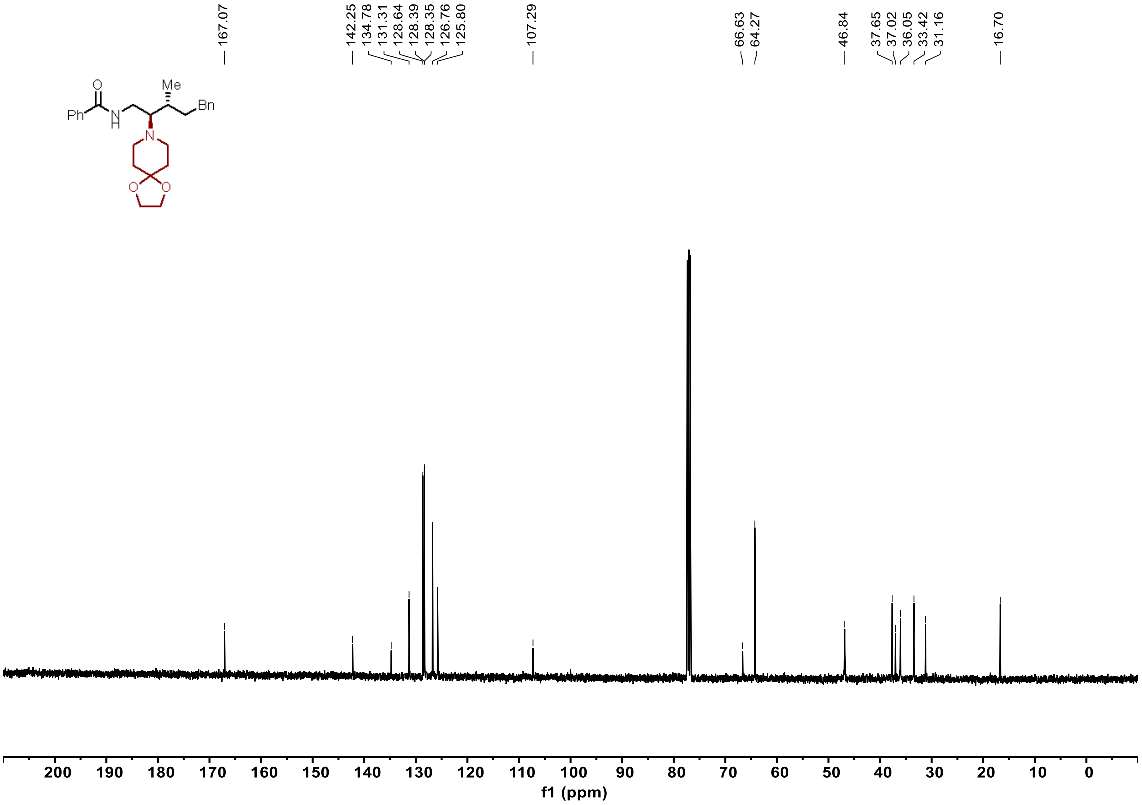

^{13}C NMR (101 MHz, $CDCl_3$) spectra of **3d**

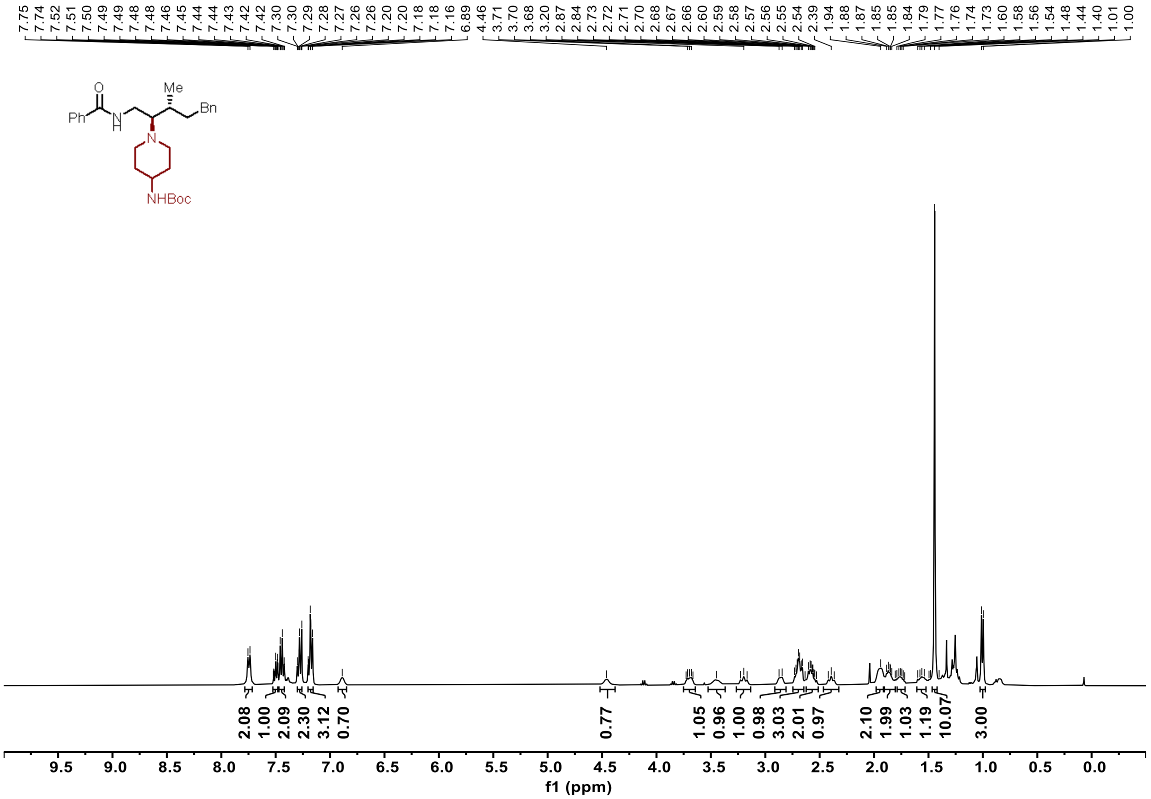

^{1}H NMR (400 MHz, $CDCl_3$) spectra of **3e**

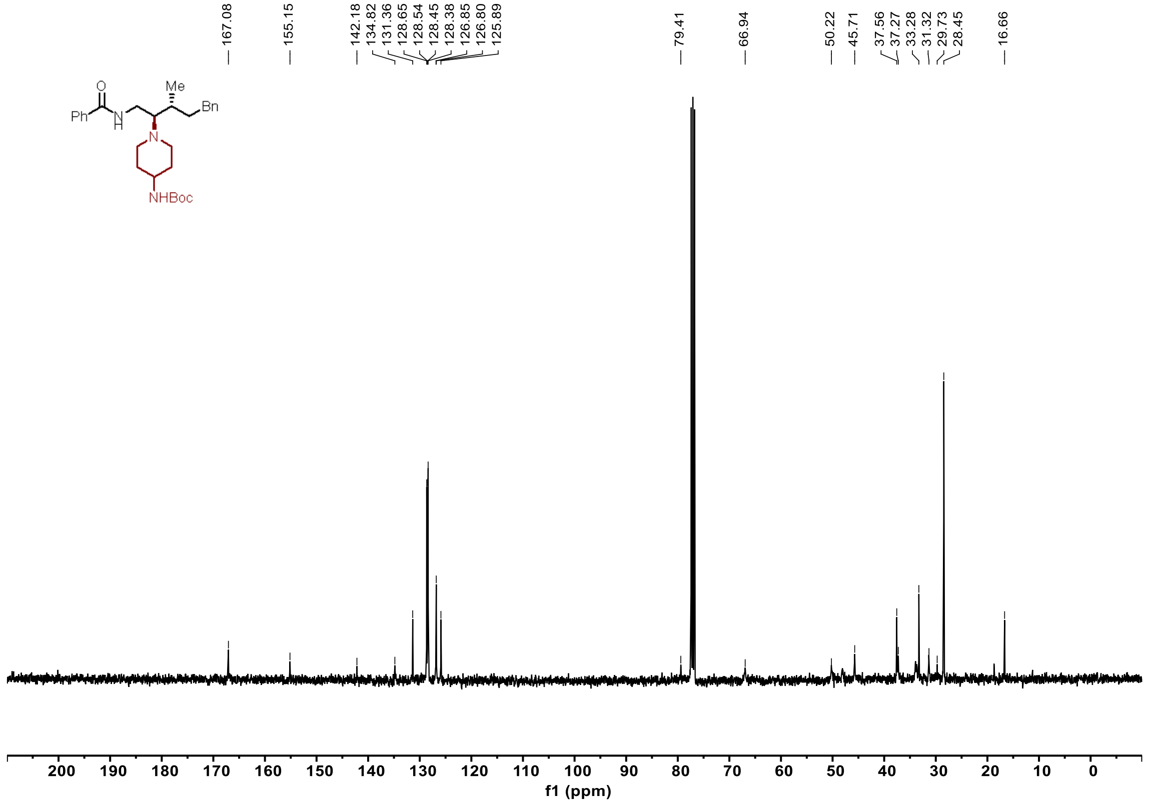

^{13}C NMR (101 MHz, $CDCl_3$) spectra of **3e**

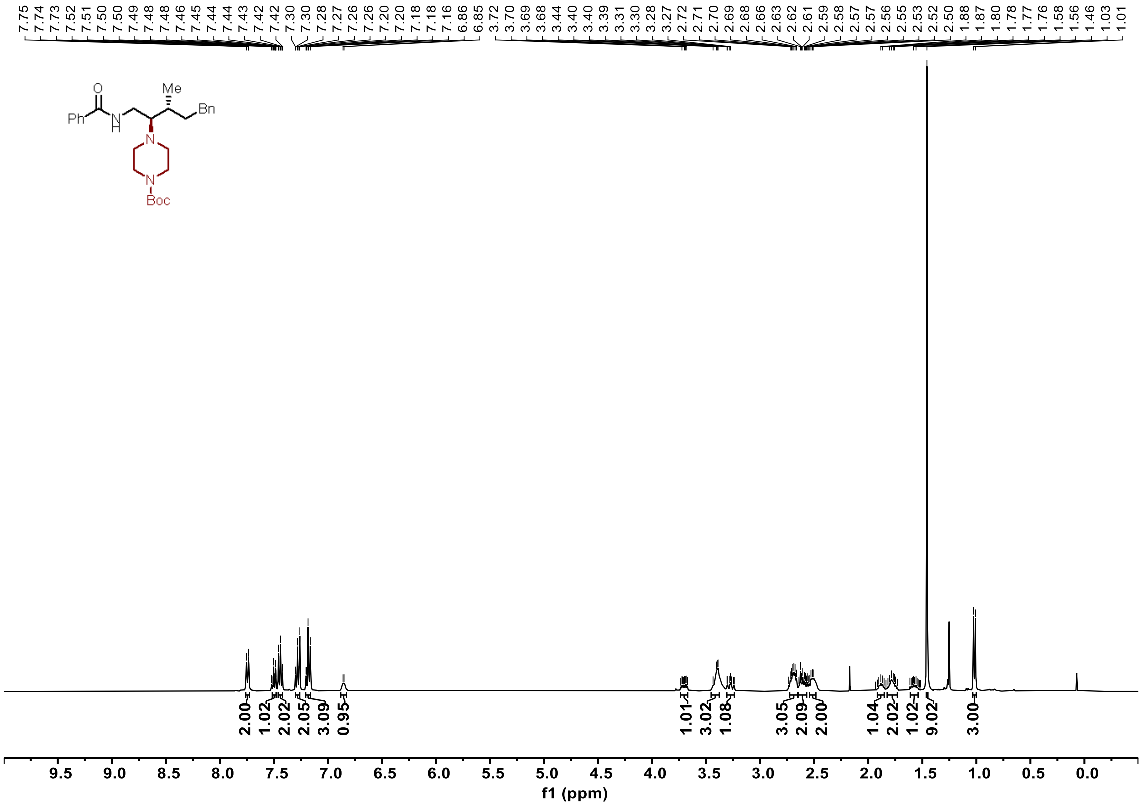

^{1}**H NMR** (400 MHz, $CDCl_3$) spectra of **3f**

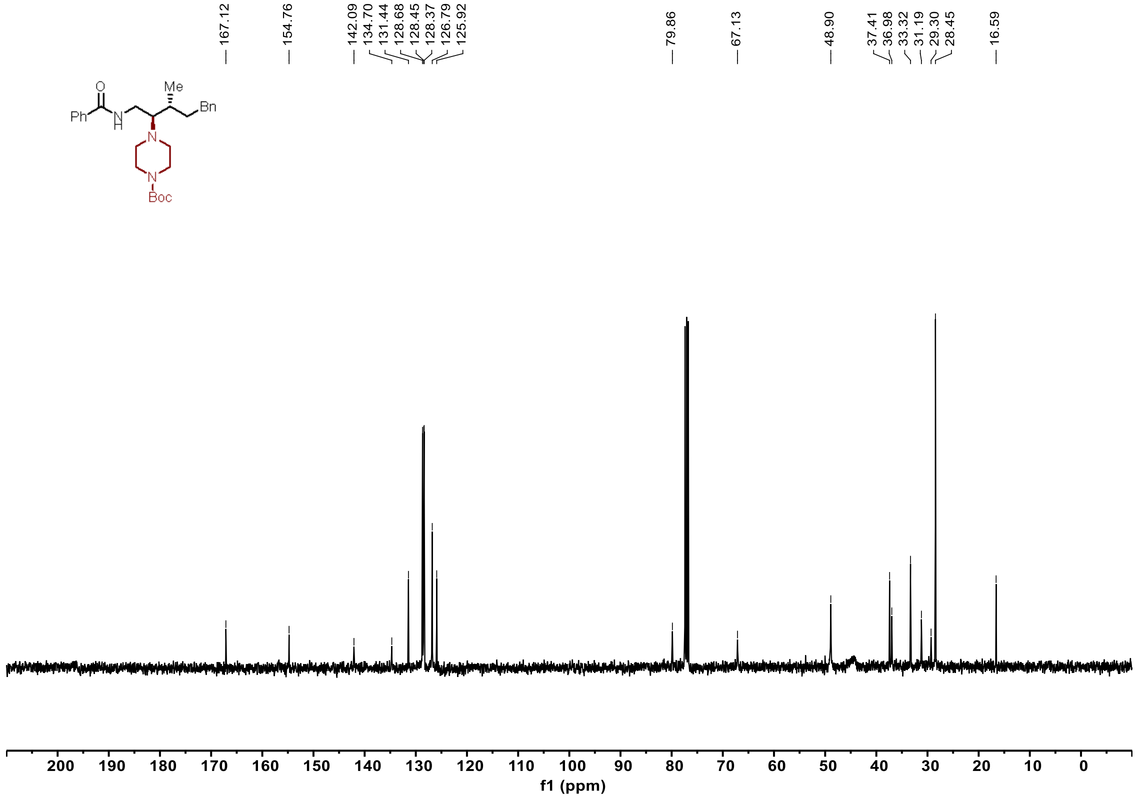

^{13}**C NMR** (101 MHz, $CDCl_3$) spectra of **3f**

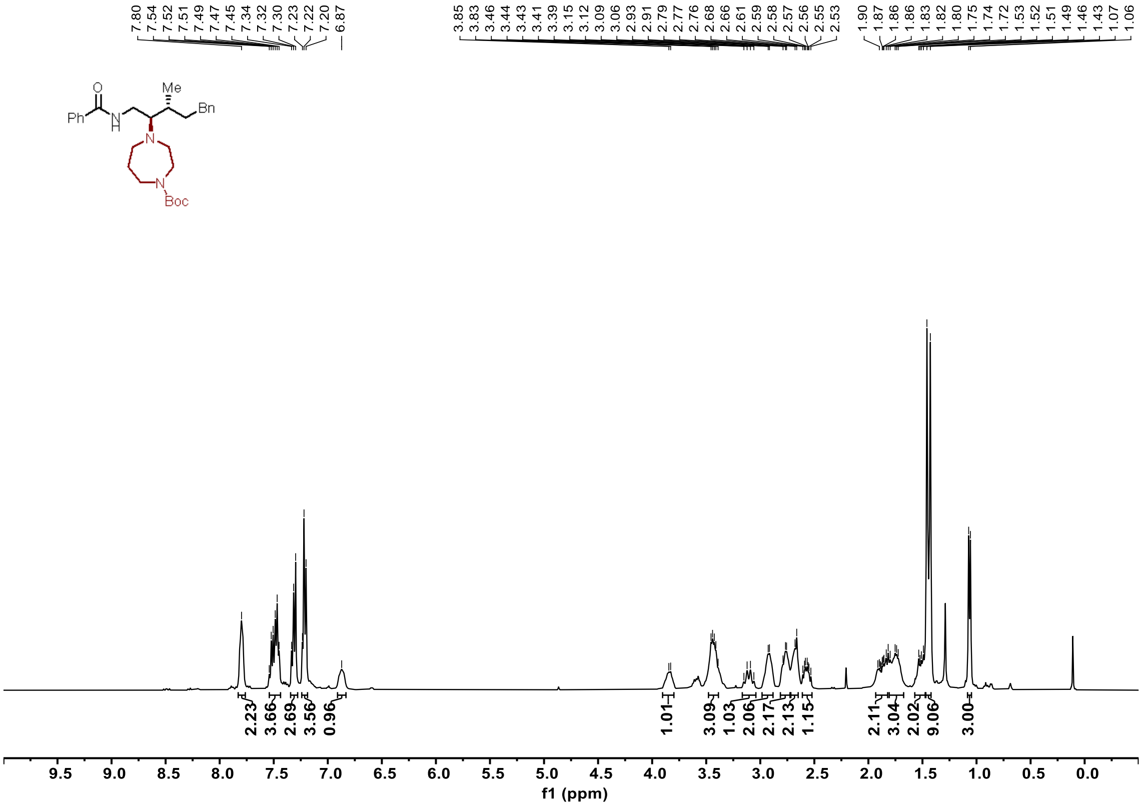

^{1}**H NMR** (400 MHz, $CDCl_3$) spectra of **3g**

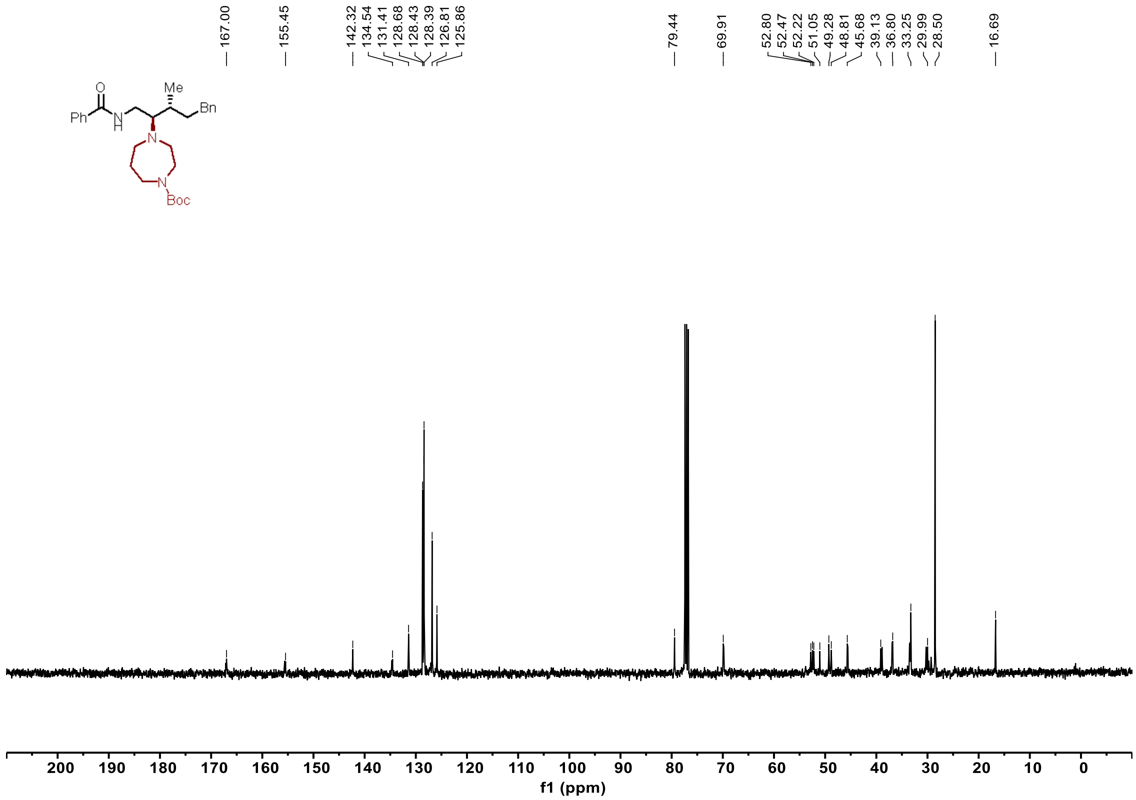

^{13}**C NMR** (101 MHz, $CDCl_3$) spectra of **3g**

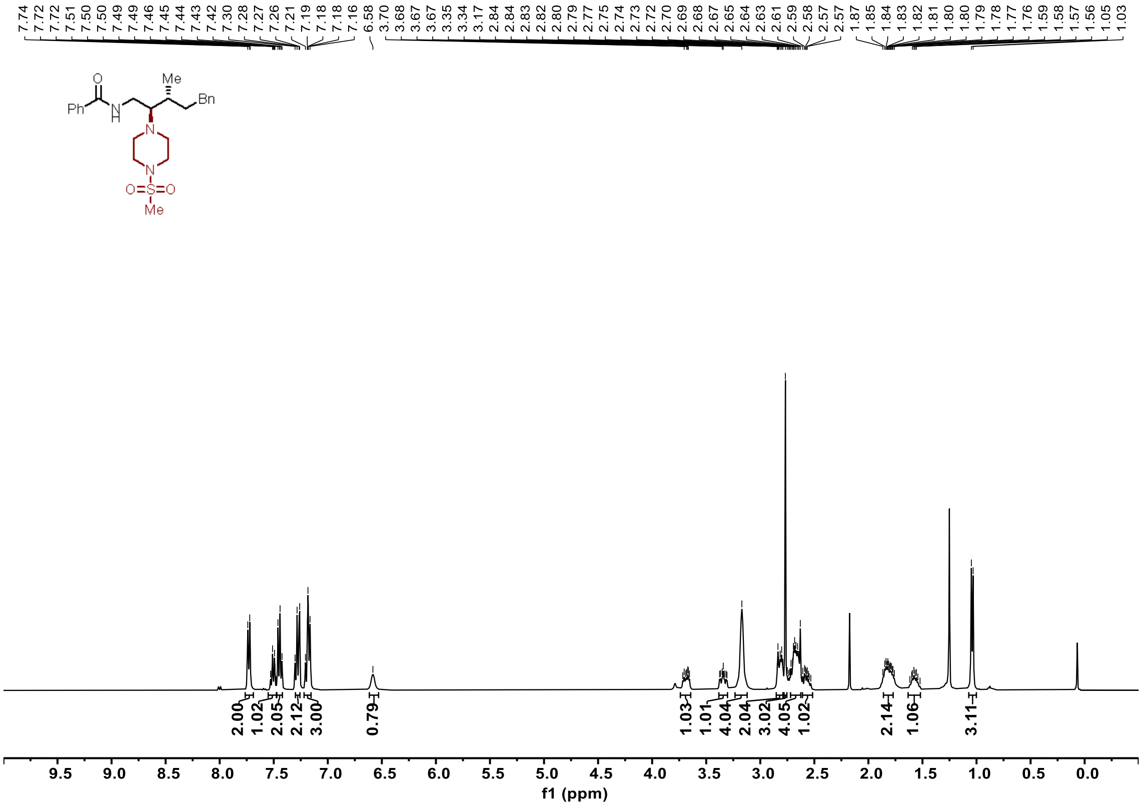

^{1}H NMR (400 MHz, $CDCl_3$) spectra of **3h**

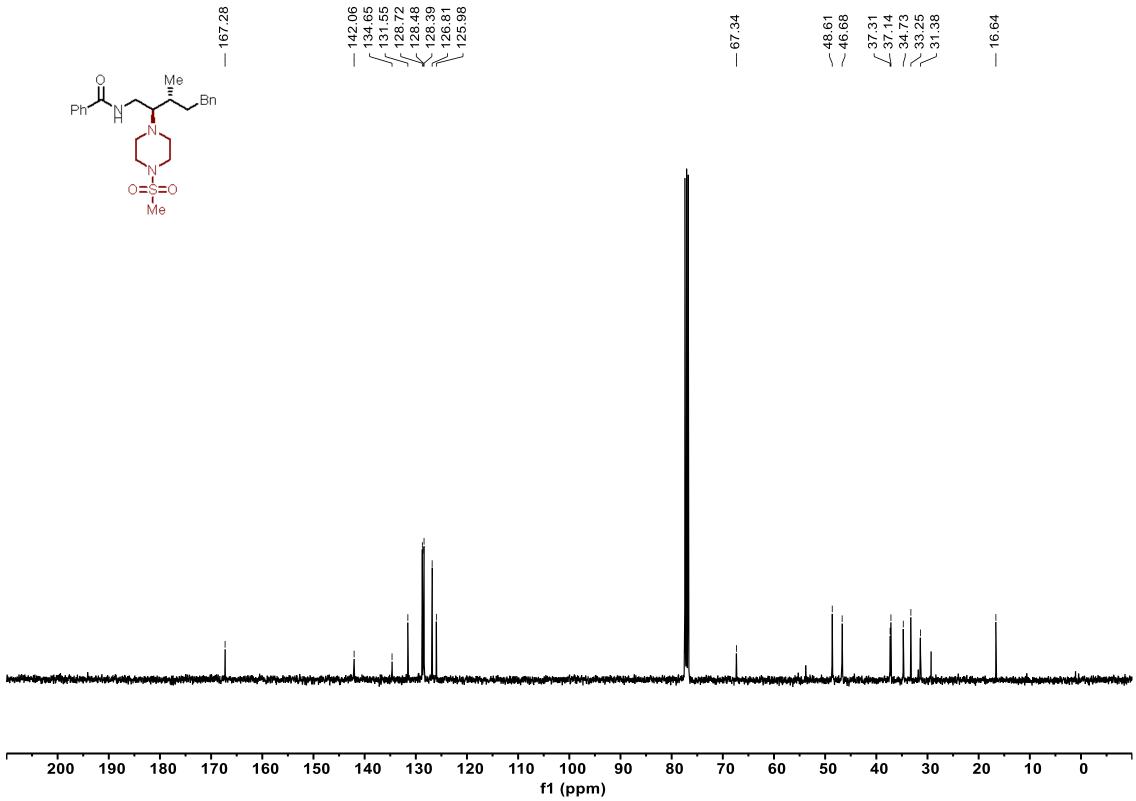

^{13}C NMR (101 MHz, $CDCl_3$) spectra of **3h**

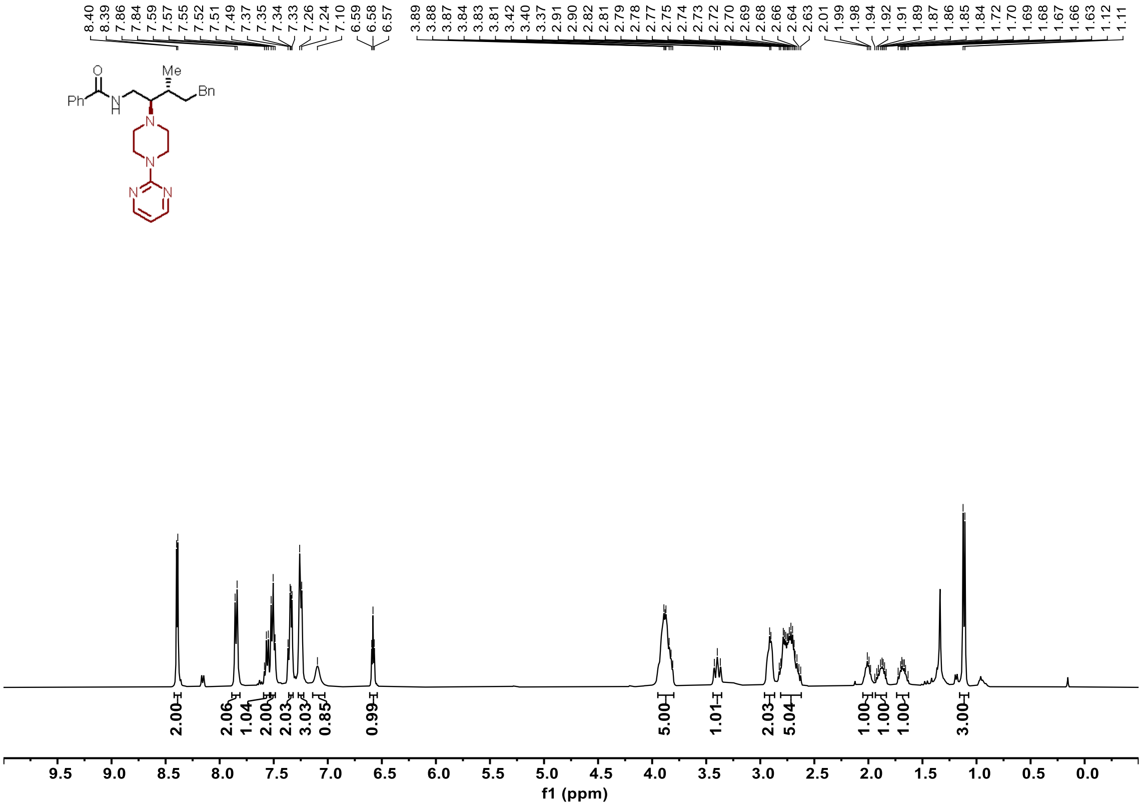

^{1}H NMR (400 MHz, $CDCl_3$) spectra of **3i**

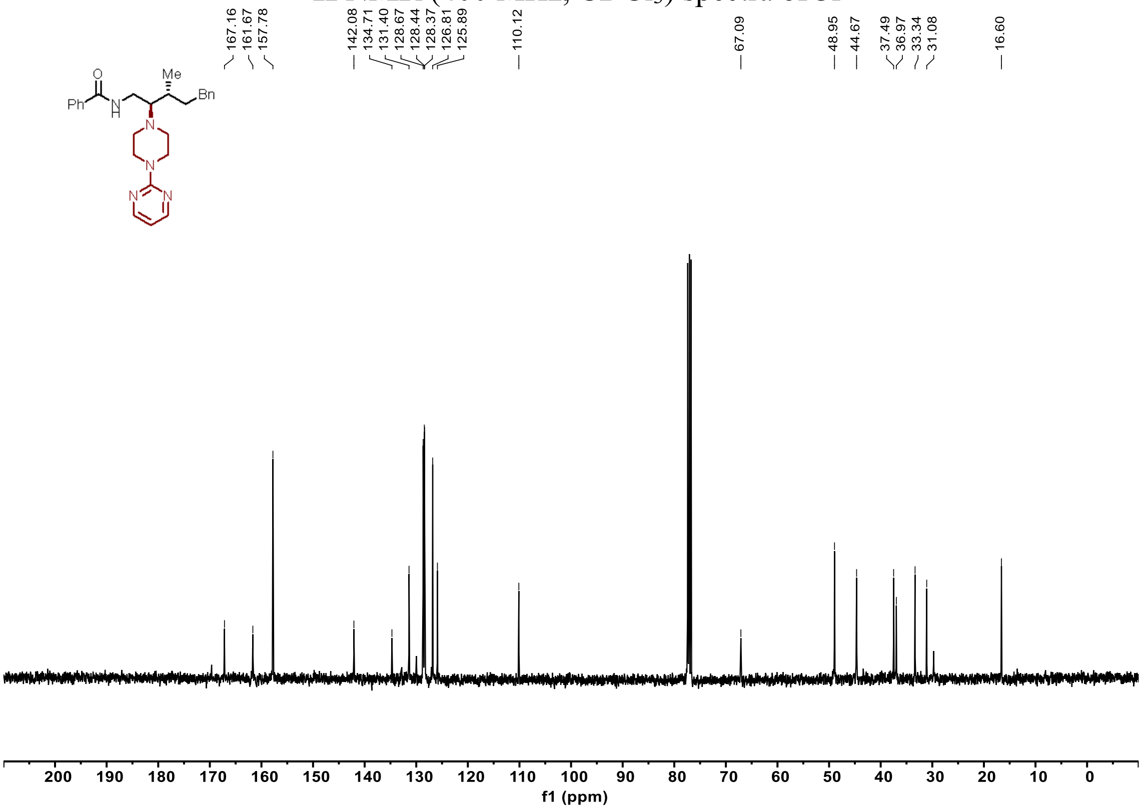

^{13}C NMR (101 MHz, $CDCl_3$) spectra of **3i**

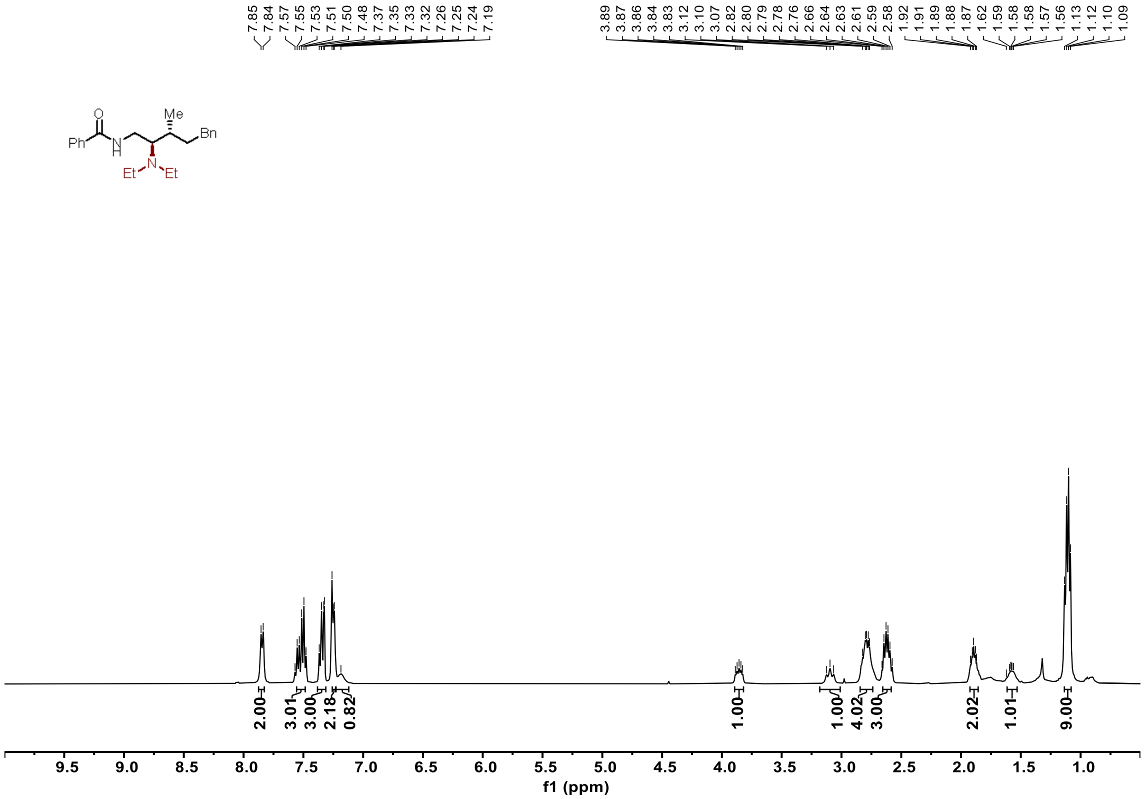

^{1}**H NMR** (400 MHz, $CDCl_3$) spectra of **3j**

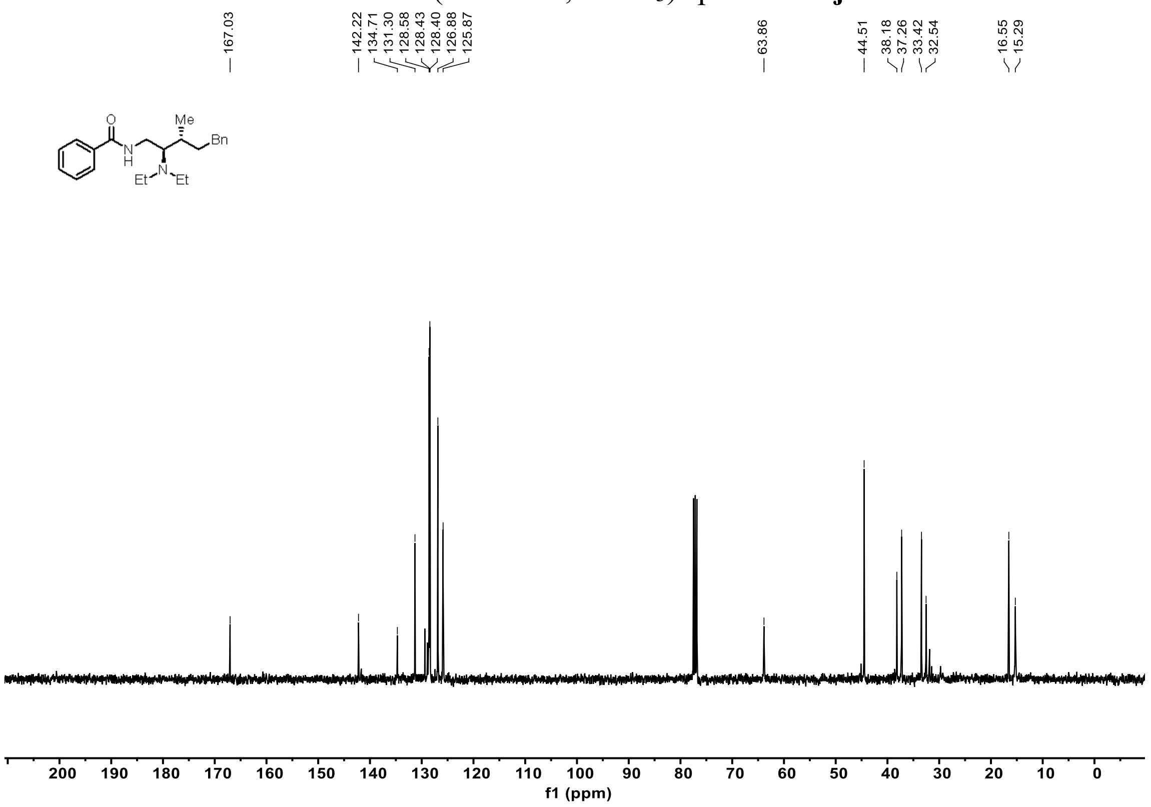

^{13}**C NMR** (101 MHz, $CDCl_3$) spectra of **3j**

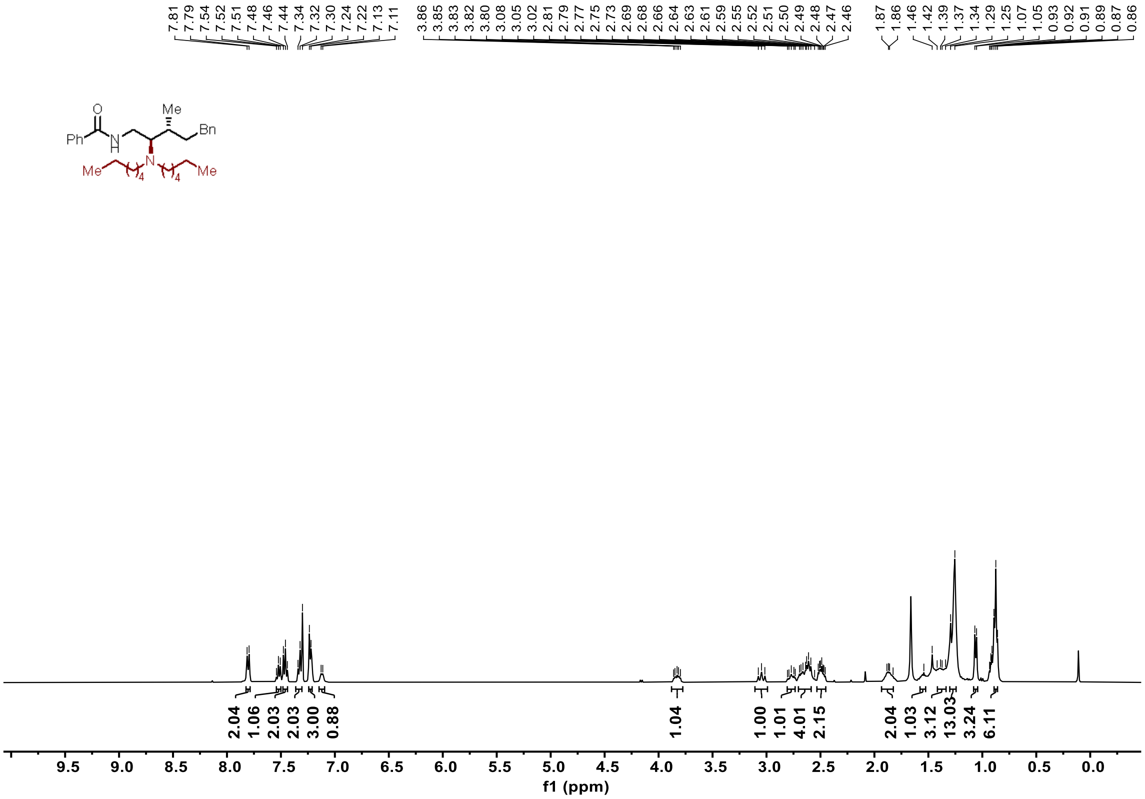

^{1}H NMR (400 MHz, $CDCl_3$) spectra of **3k**

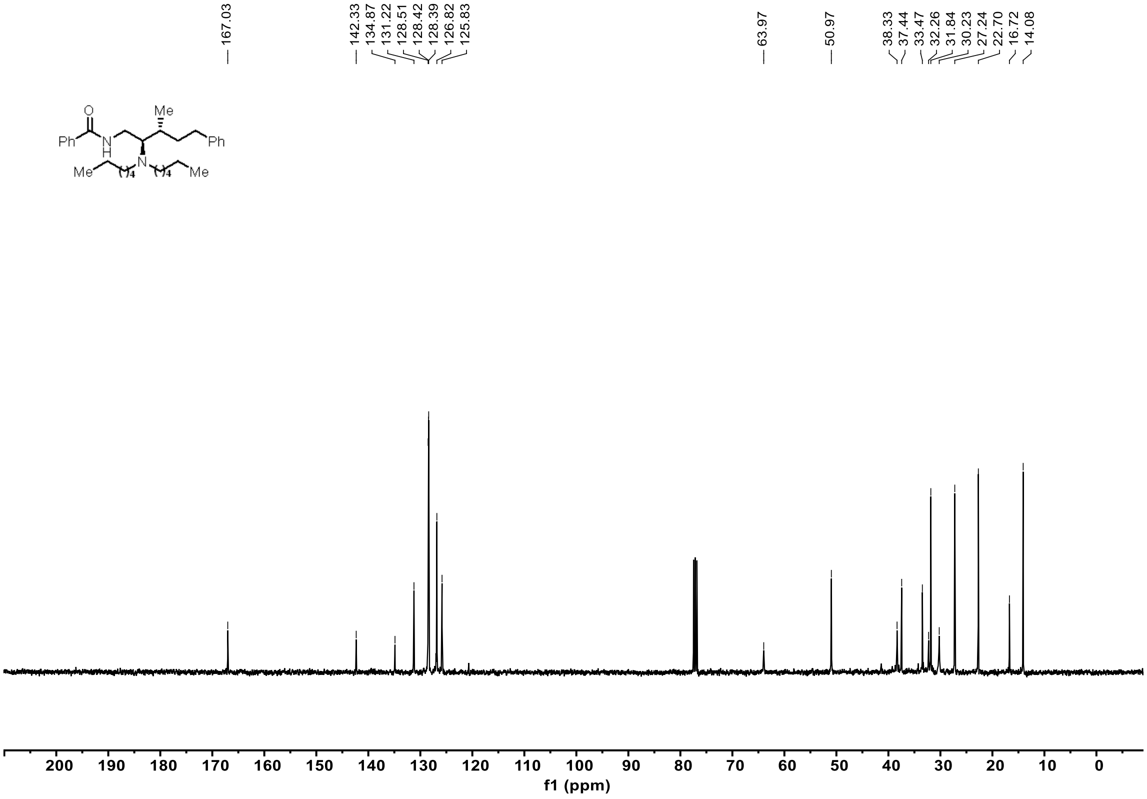

^{13}C NMR (101 MHz, $CDCl_3$) spectra of **3k**

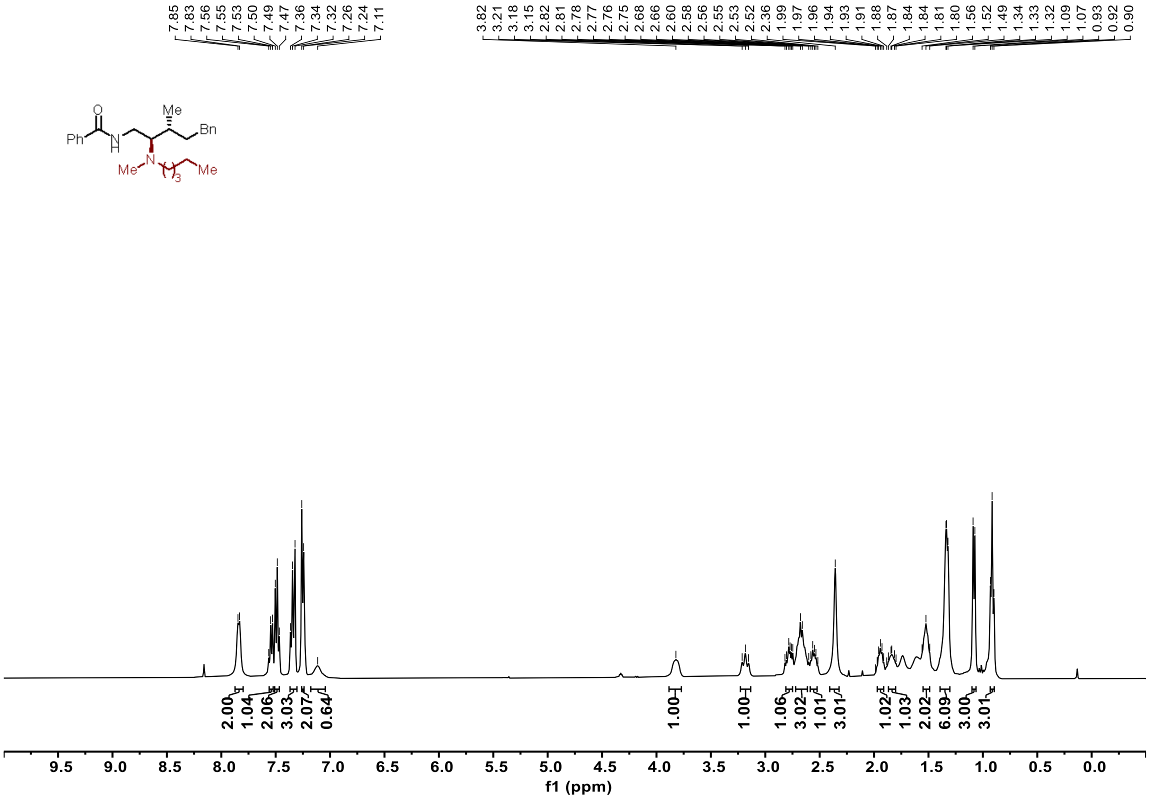

^{1}H NMR (400 MHz, $CDCl_3$) spectra of **3l**

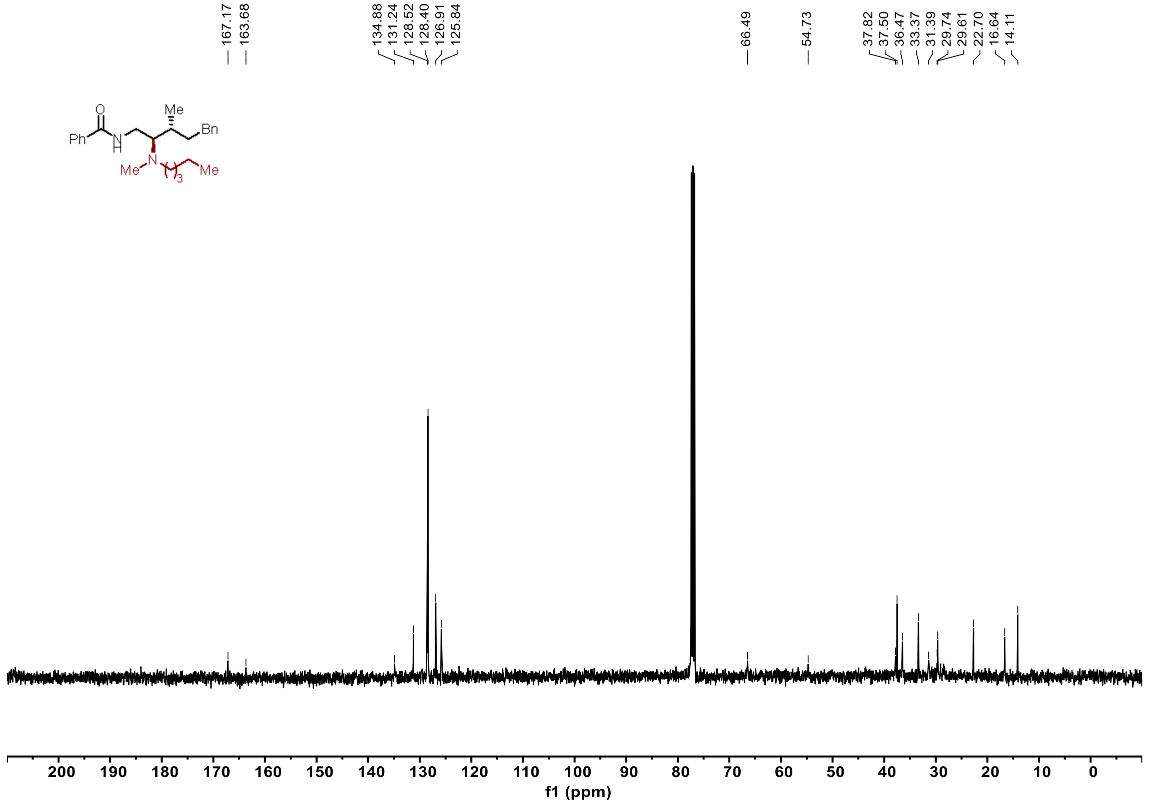

^{13}C NMR (101 MHz, $CDCl_3$) spectra of **3l**

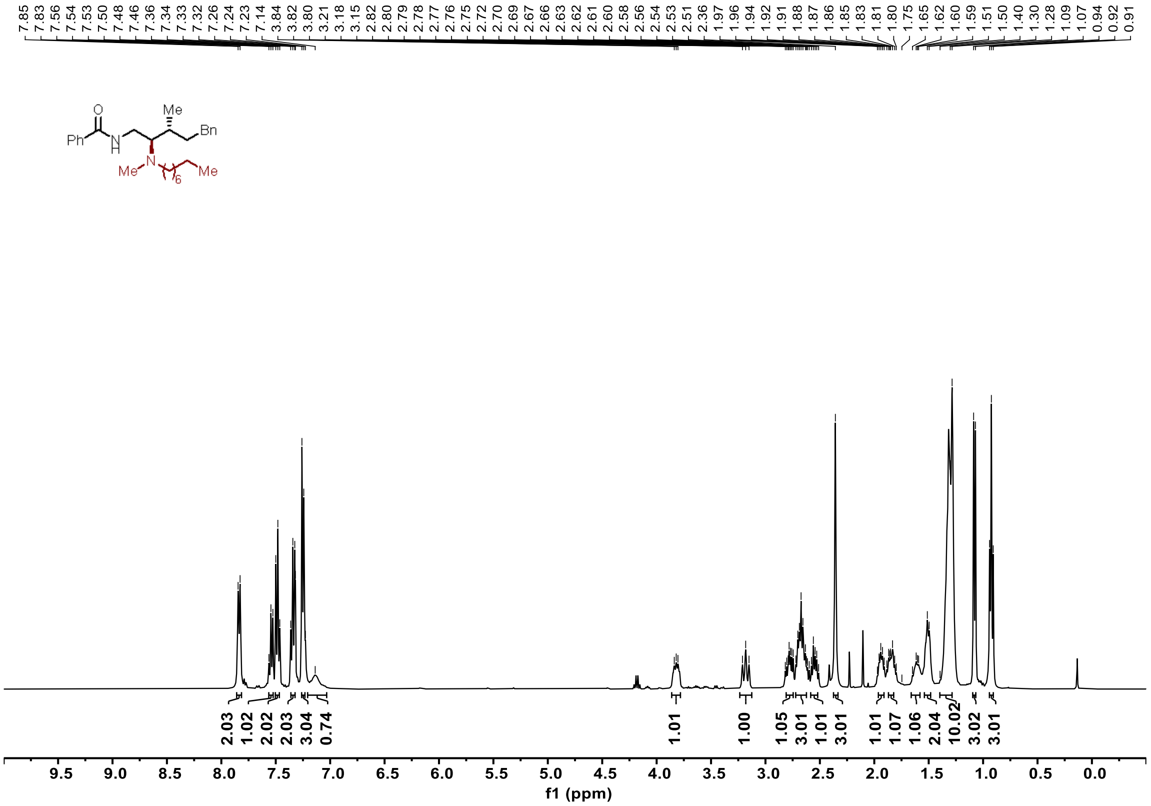

^{1}H NMR (400 MHz, $CDCl_3$) spectra of **3m**

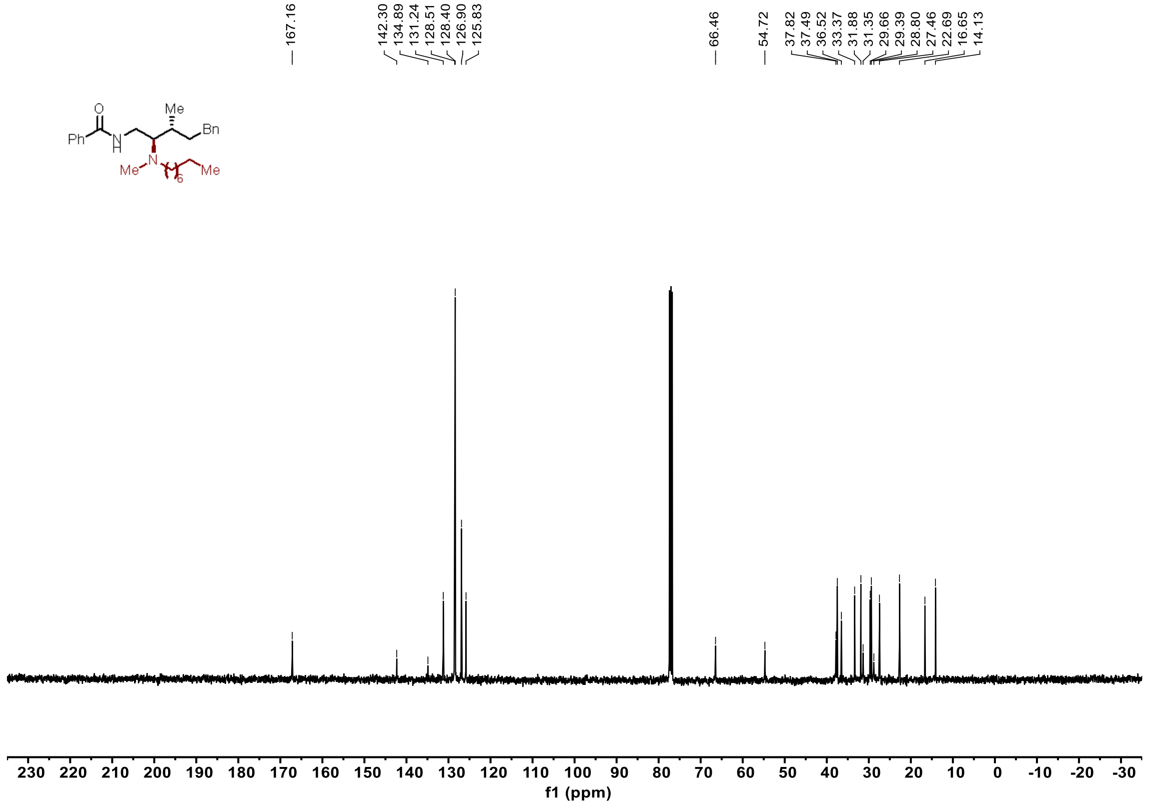

^{13}C NMR (101 MHz, $CDCl_3$) spectra of **3m**

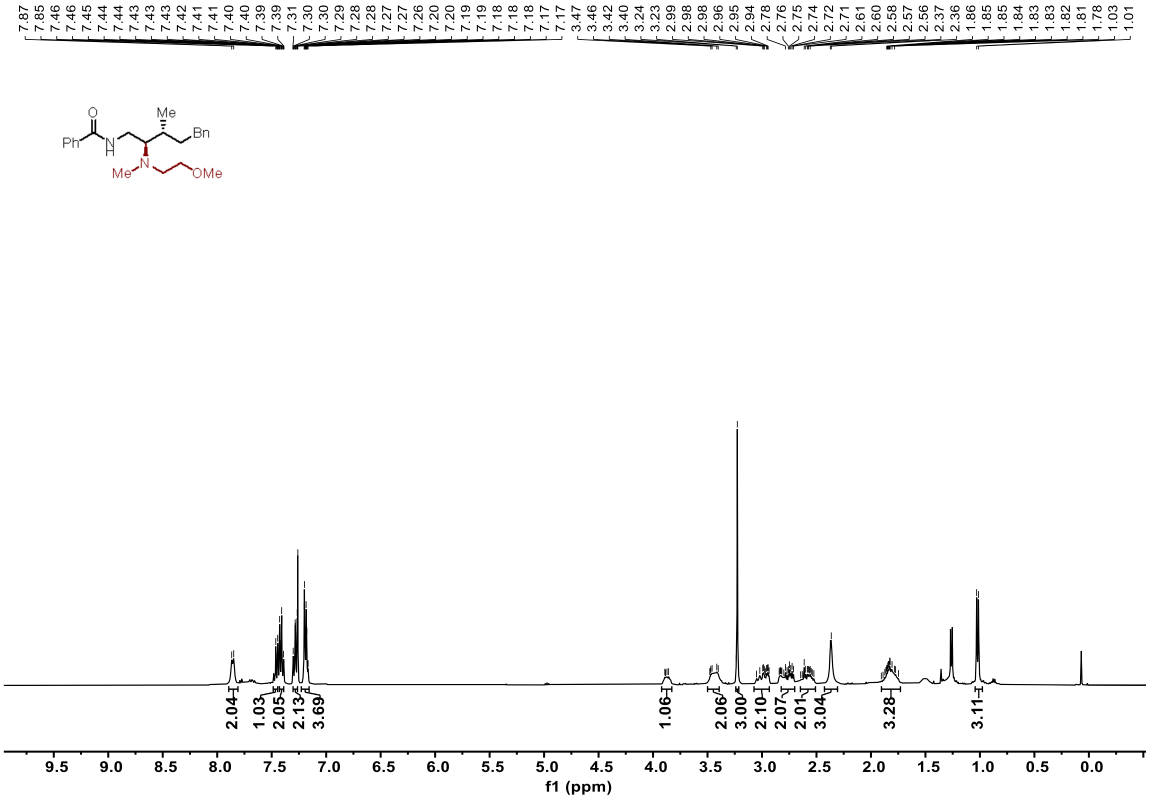

^{1}**H NMR** (400 MHz, CDCl$_3$) spectra of **3n**

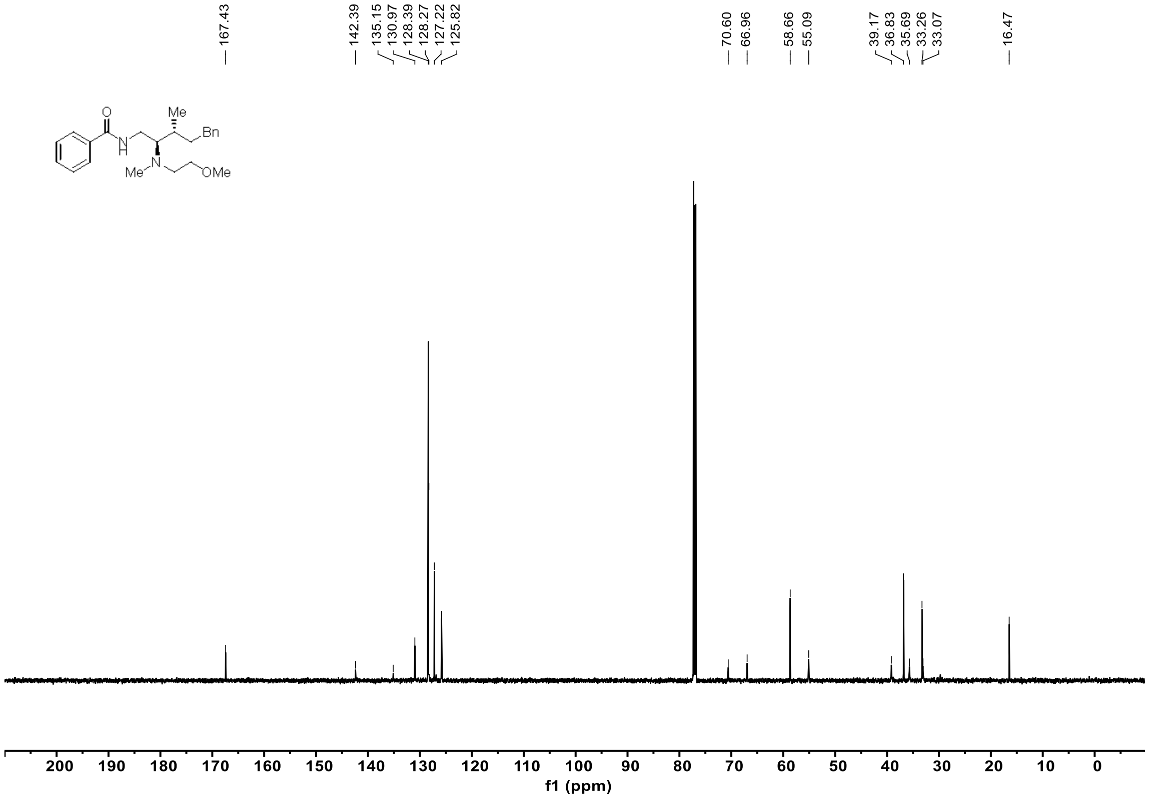

^{13}**C NMR** (101 MHz, CDCl$_3$) spectra of **3n**

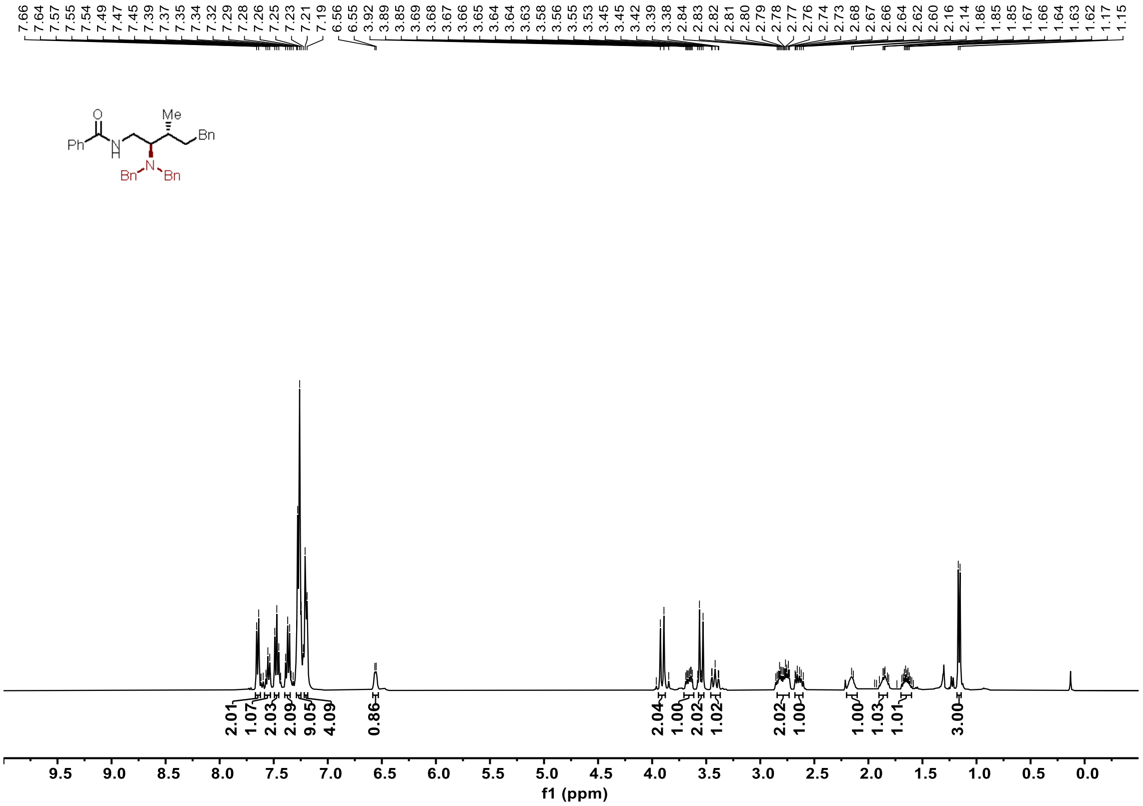

^{1}**H NMR** (400 MHz, $CDCl_3$) spectra of **3o**

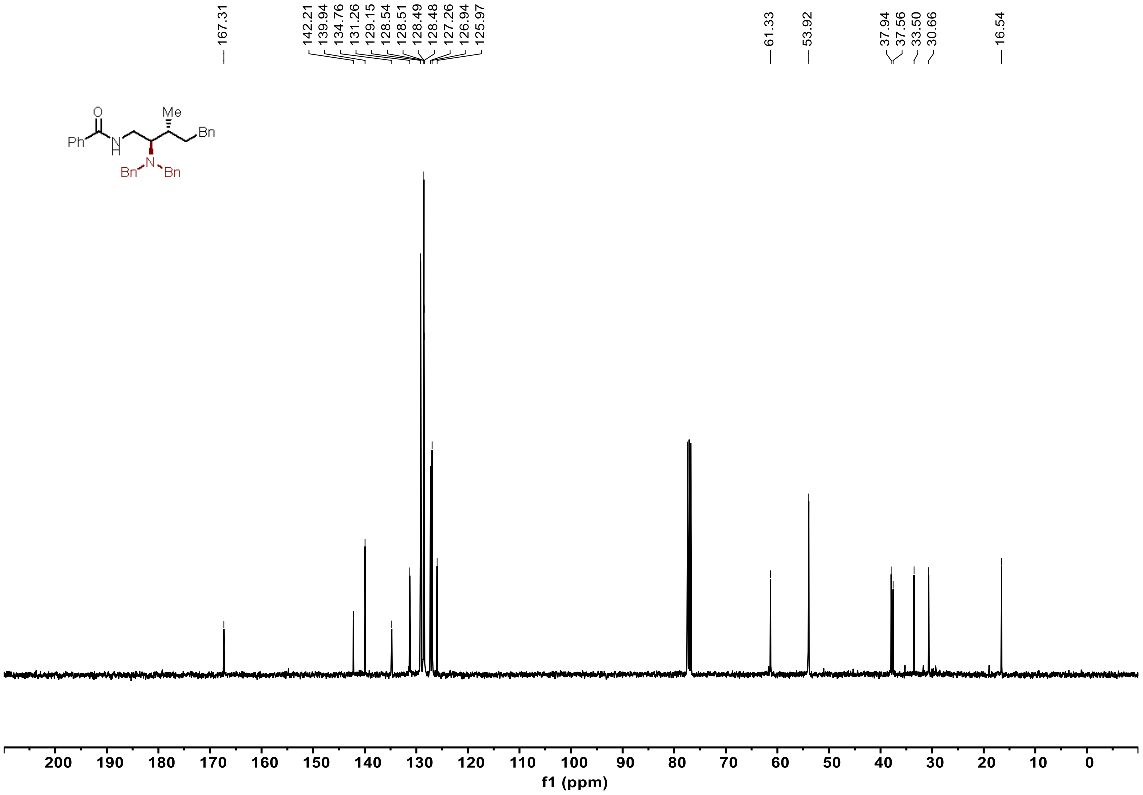

^{13}**C NMR** (101 MHz, $CDCl_3$) spectra of **3o**

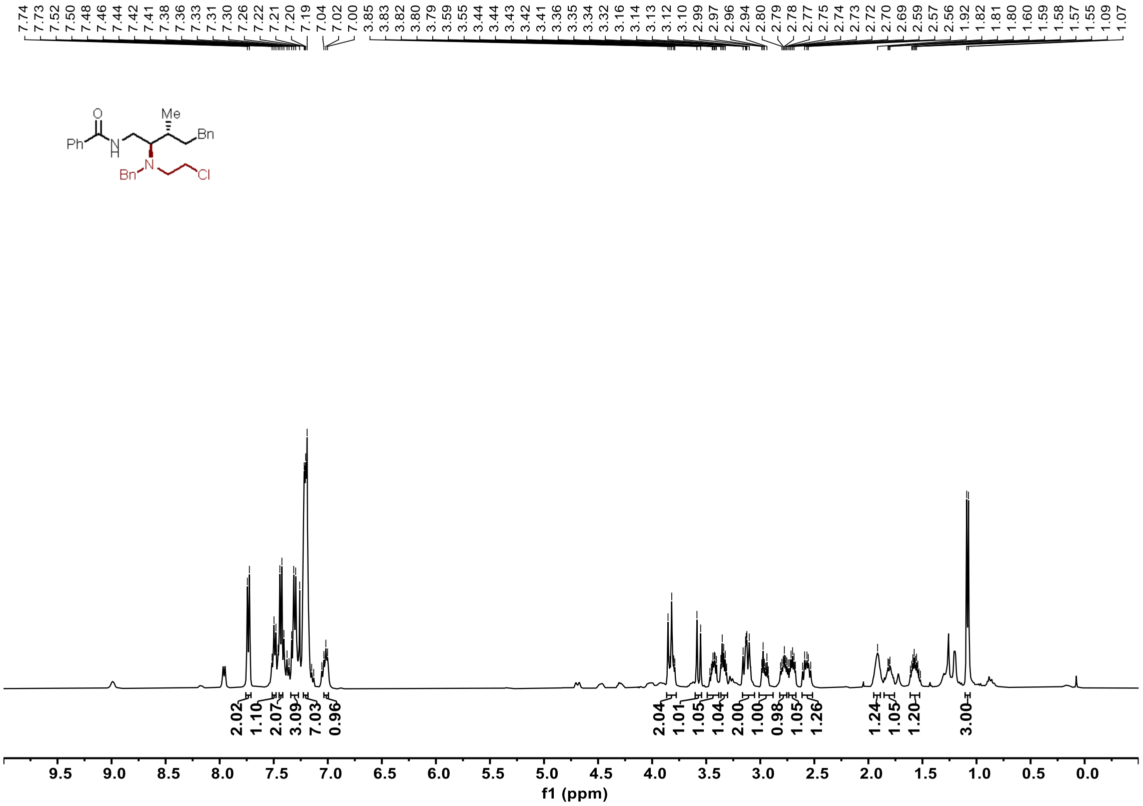

^{1}H NMR (400 MHz, $CDCl_3$) spectra of **3p**

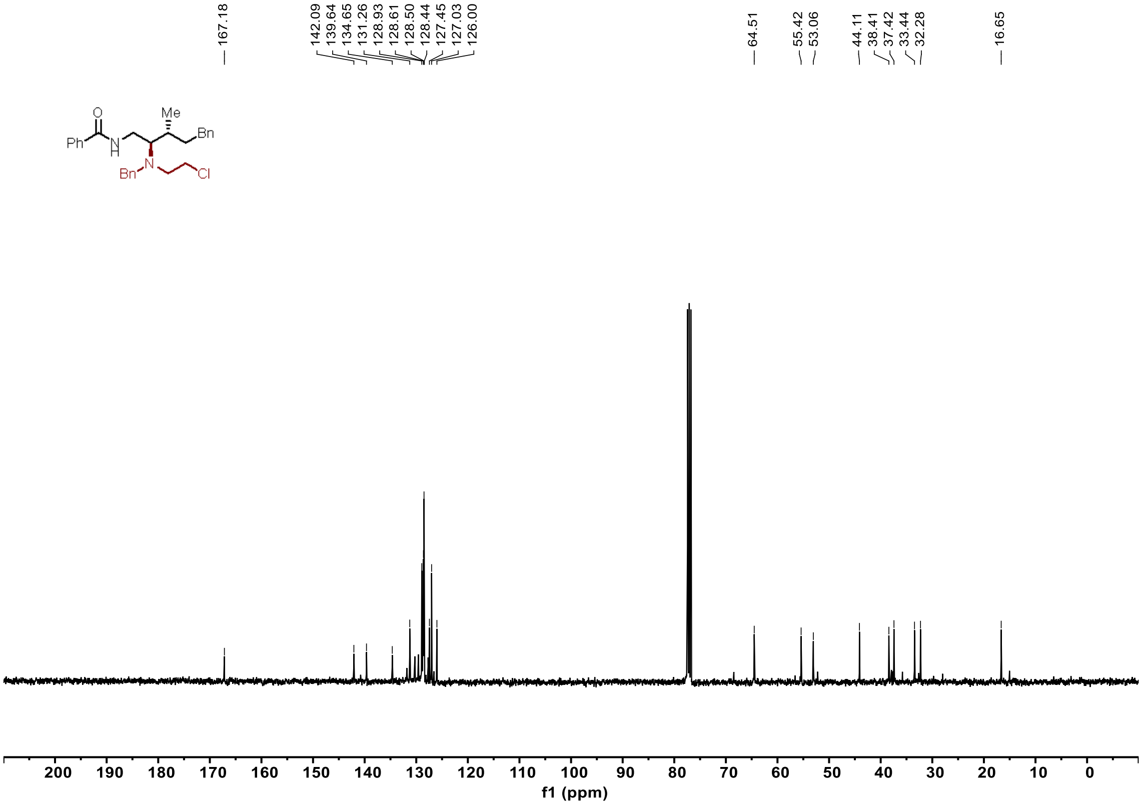

^{13}C NMR (101 MHz, $CDCl_3$) spectra of **3p**

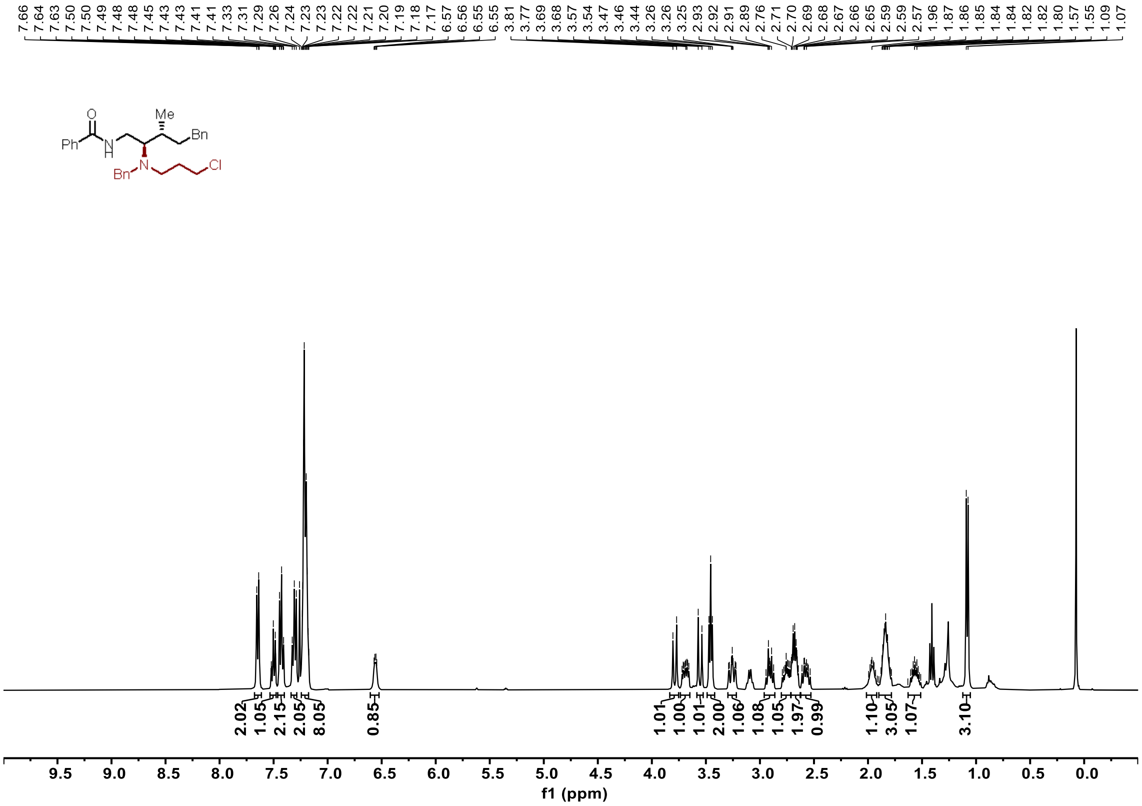

^{1}H NMR (400 MHz, $CDCl_3$) spectra of **3q**

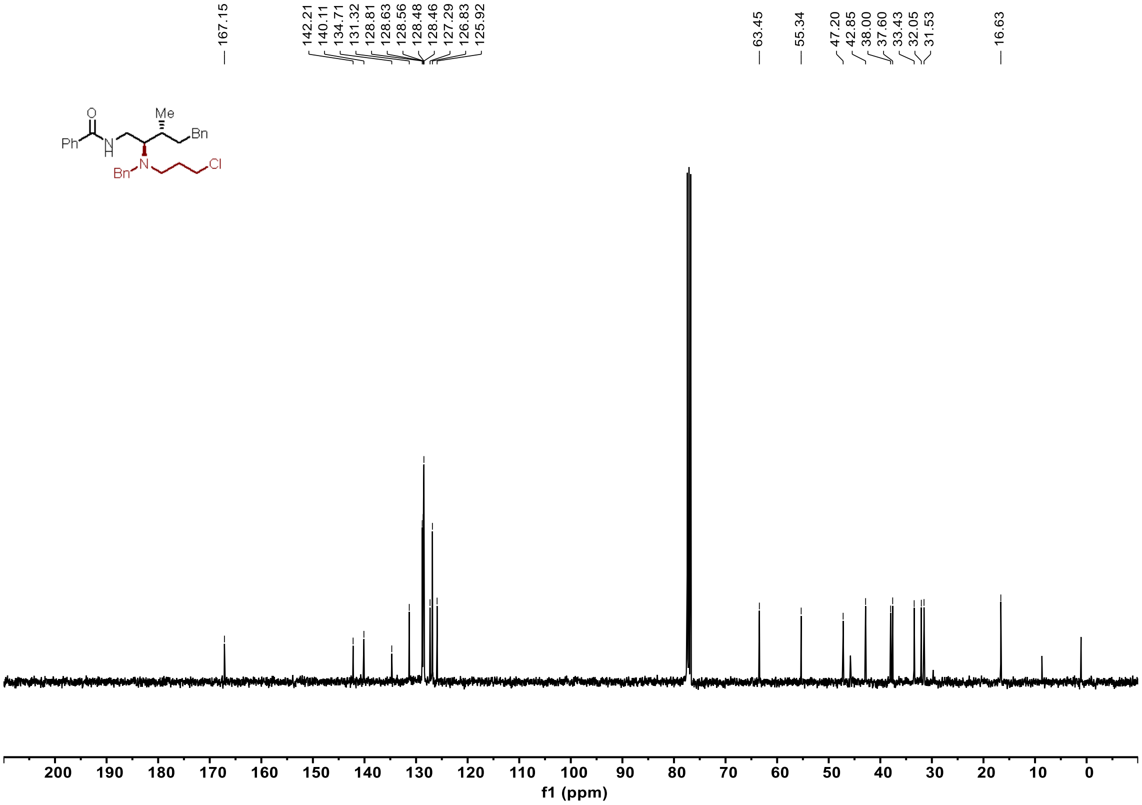

^{13}C NMR (101 MHz, $CDCl_3$) spectra of **3q**

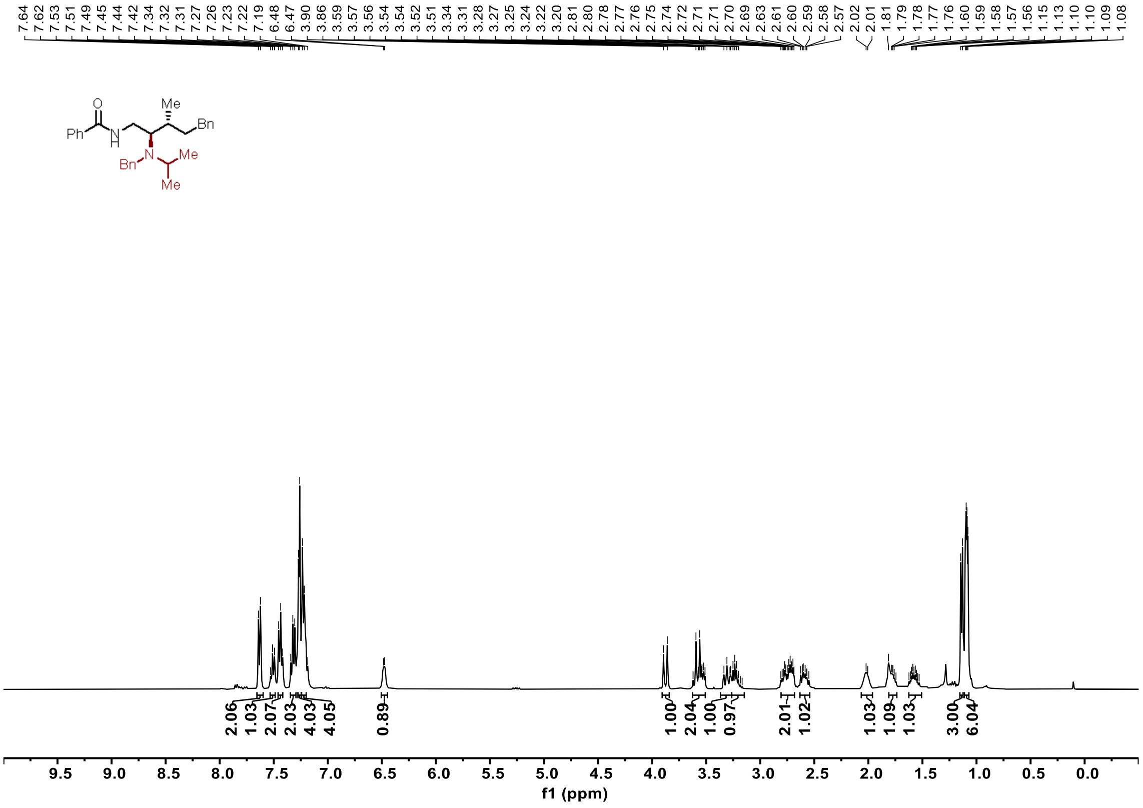

^{1}H NMR (400 MHz, $CDCl_3$) spectra of **3r**

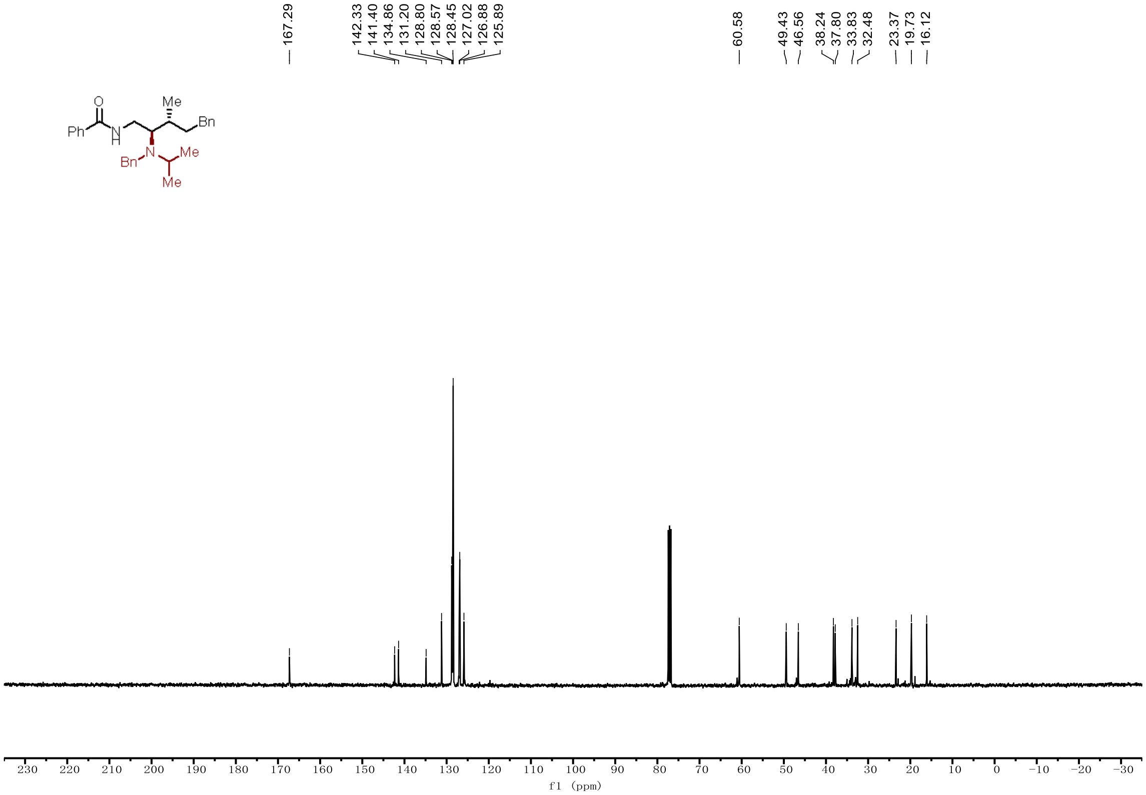

^{13}C NMR (101 MHz, $CDCl_3$) spectra of **3r**

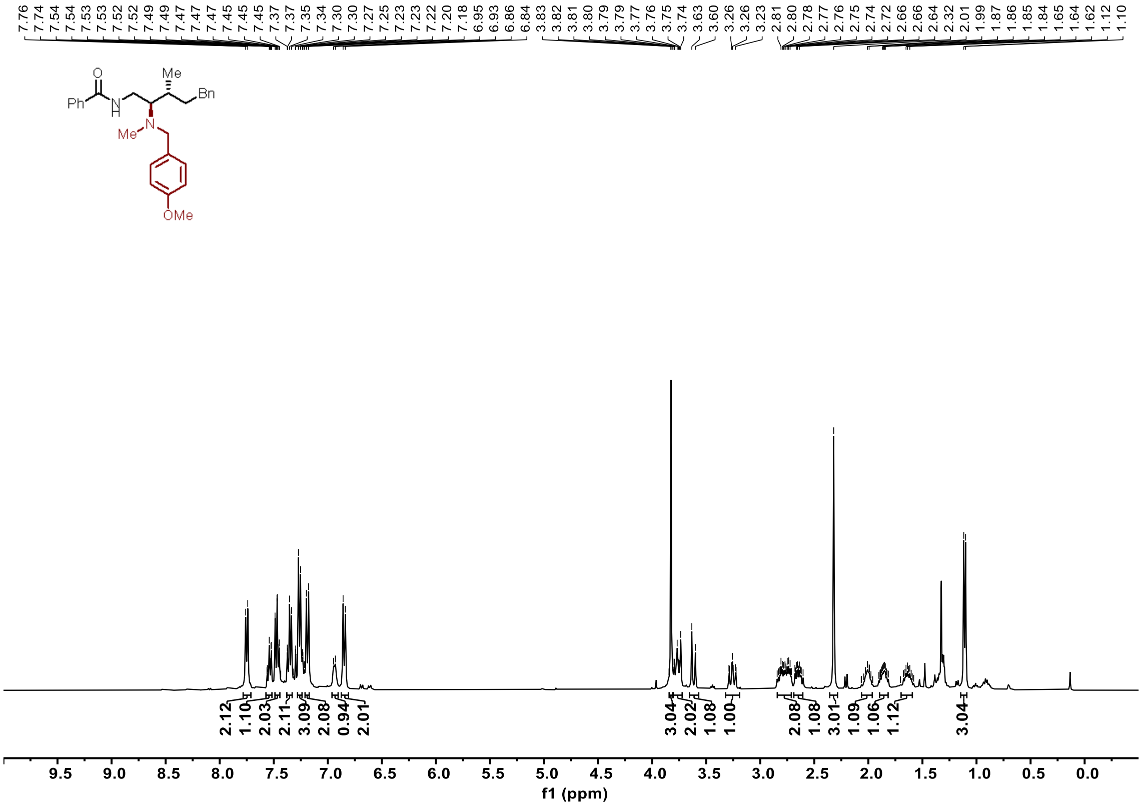

¹H NMR (400 MHz, $CDCl_3$) spectra of **3s**

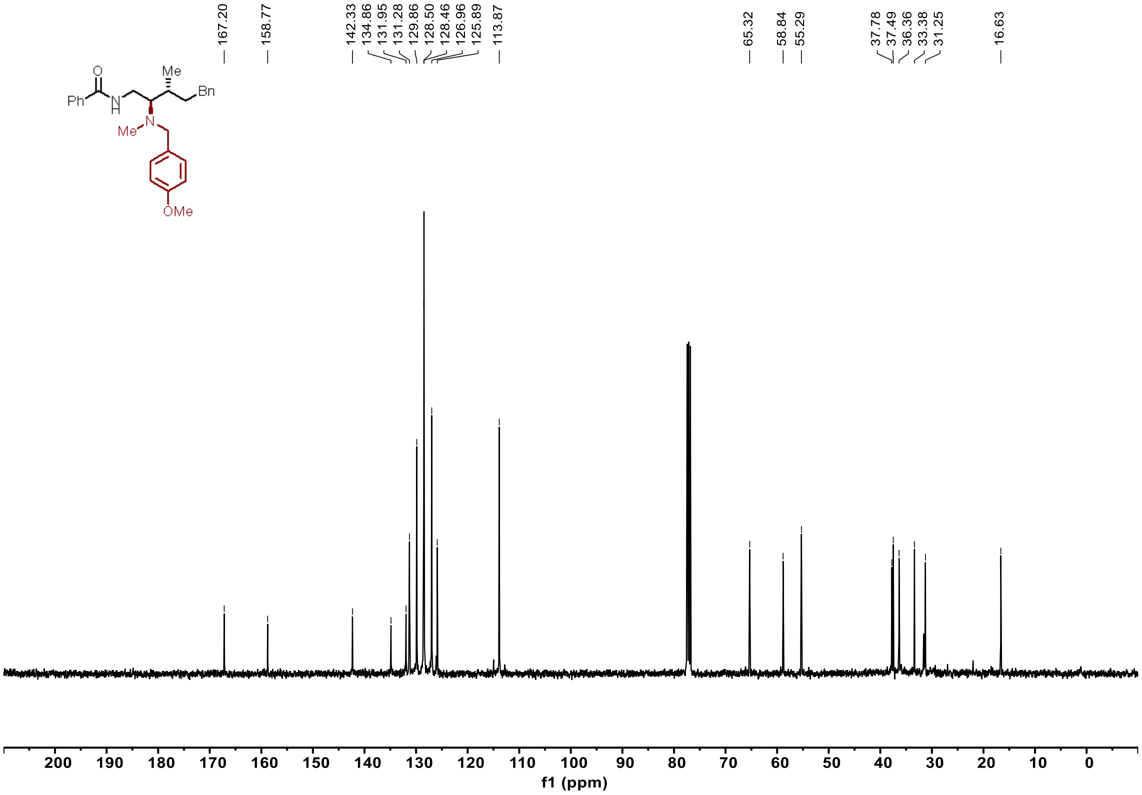

¹³C NMR (101 MHz, $CDCl_3$) spectra of **3s**

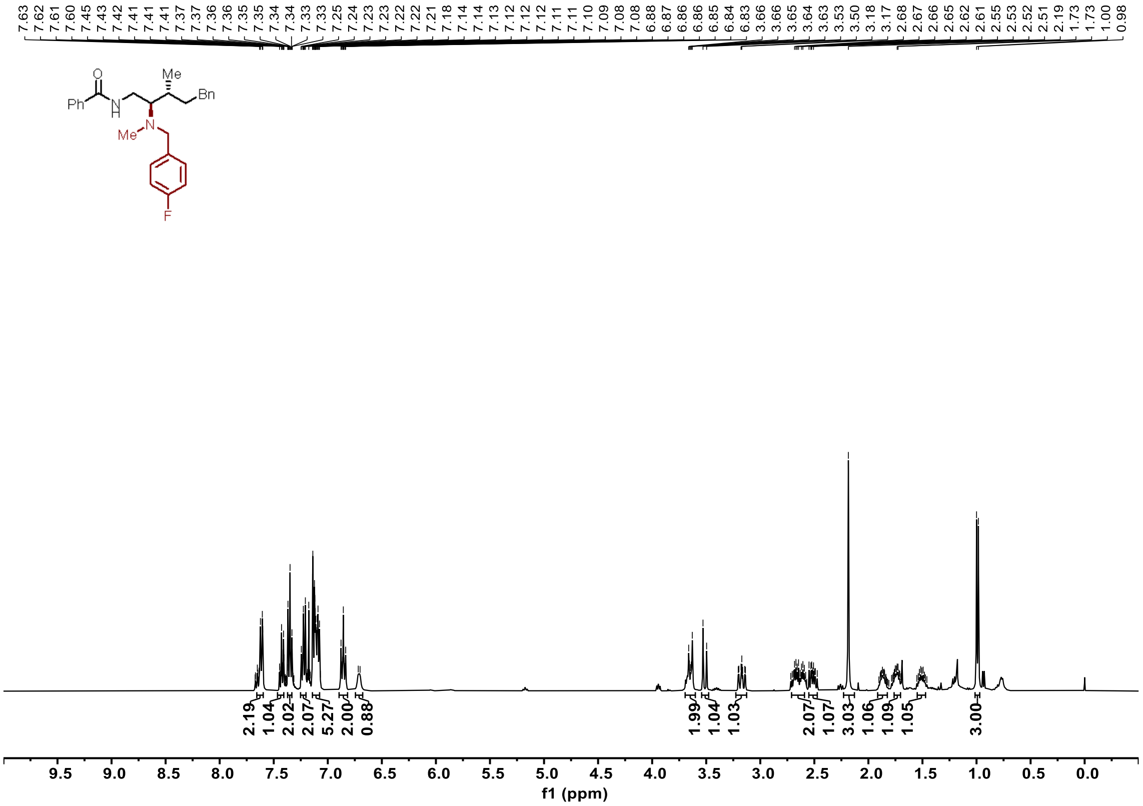

^{1}H NMR (400 MHz, $CDCl_3$) spectra of **3t**

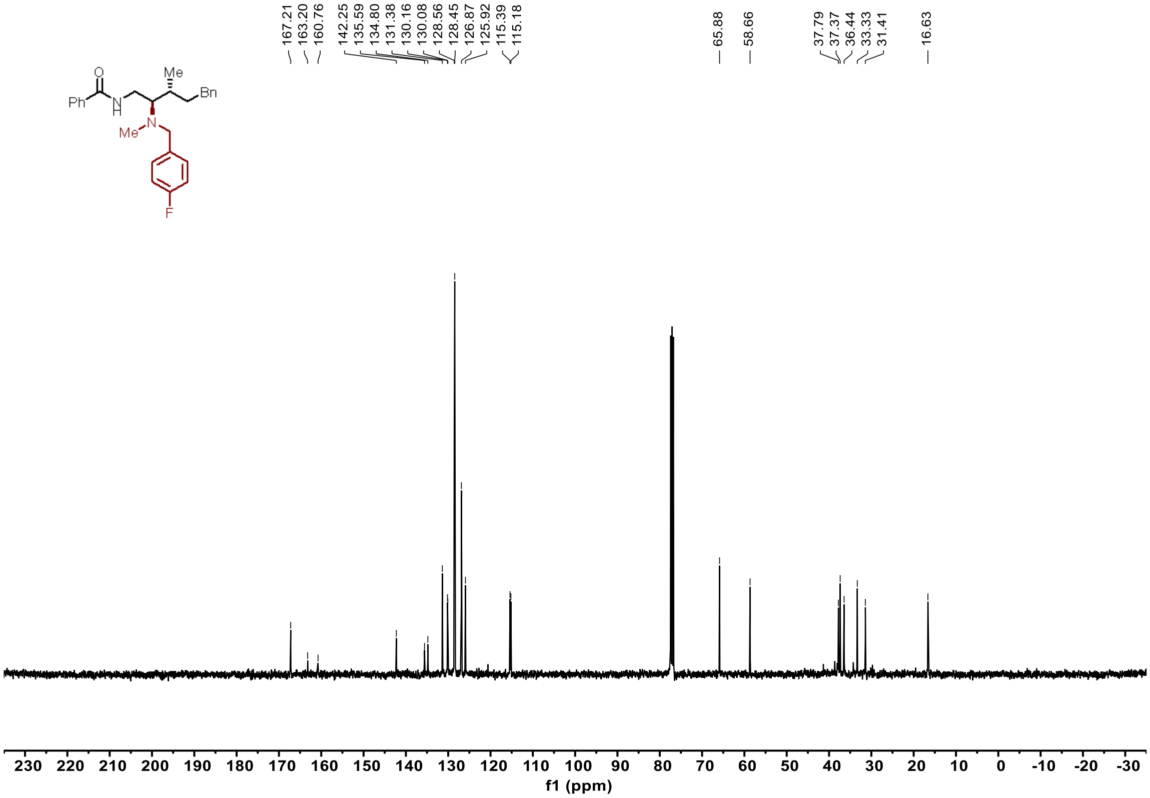

^{13}C NMR (101 MHz, $CDCl_3$) spectra of **3t**

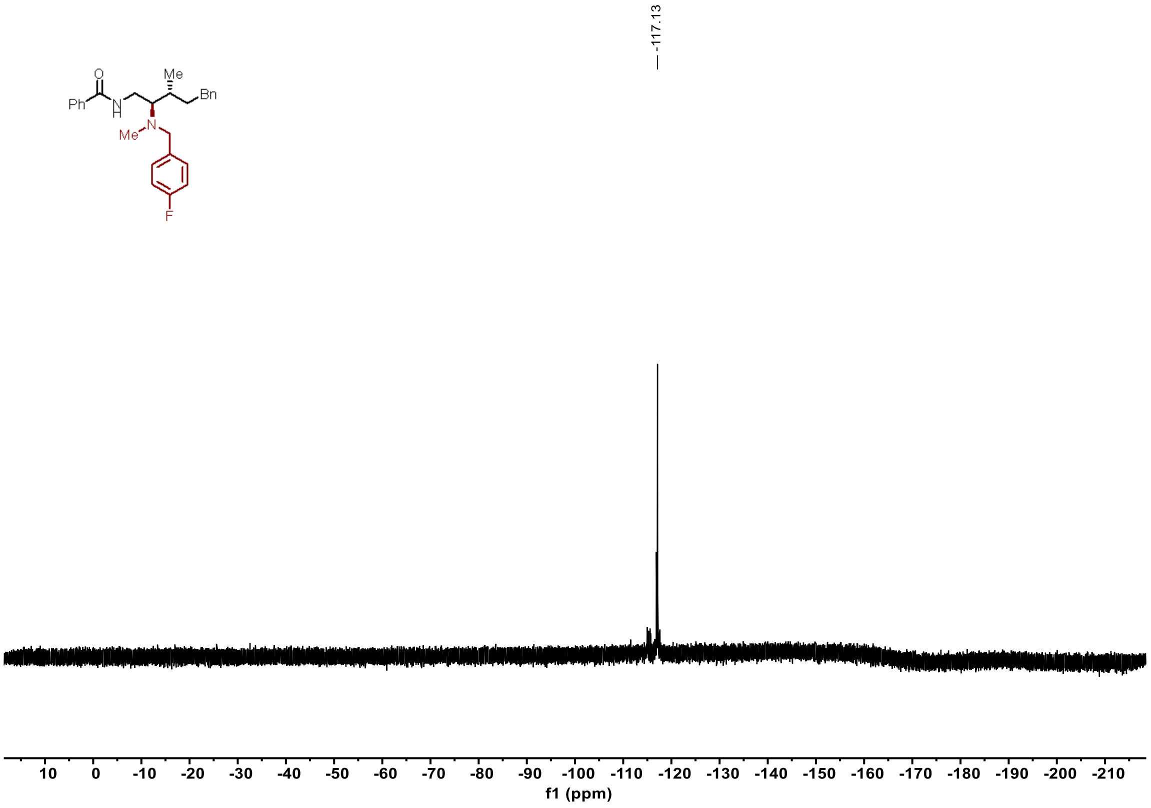

^{19}**F NMR** (376 MHz, CDCl$_3$) spectra of **3t**

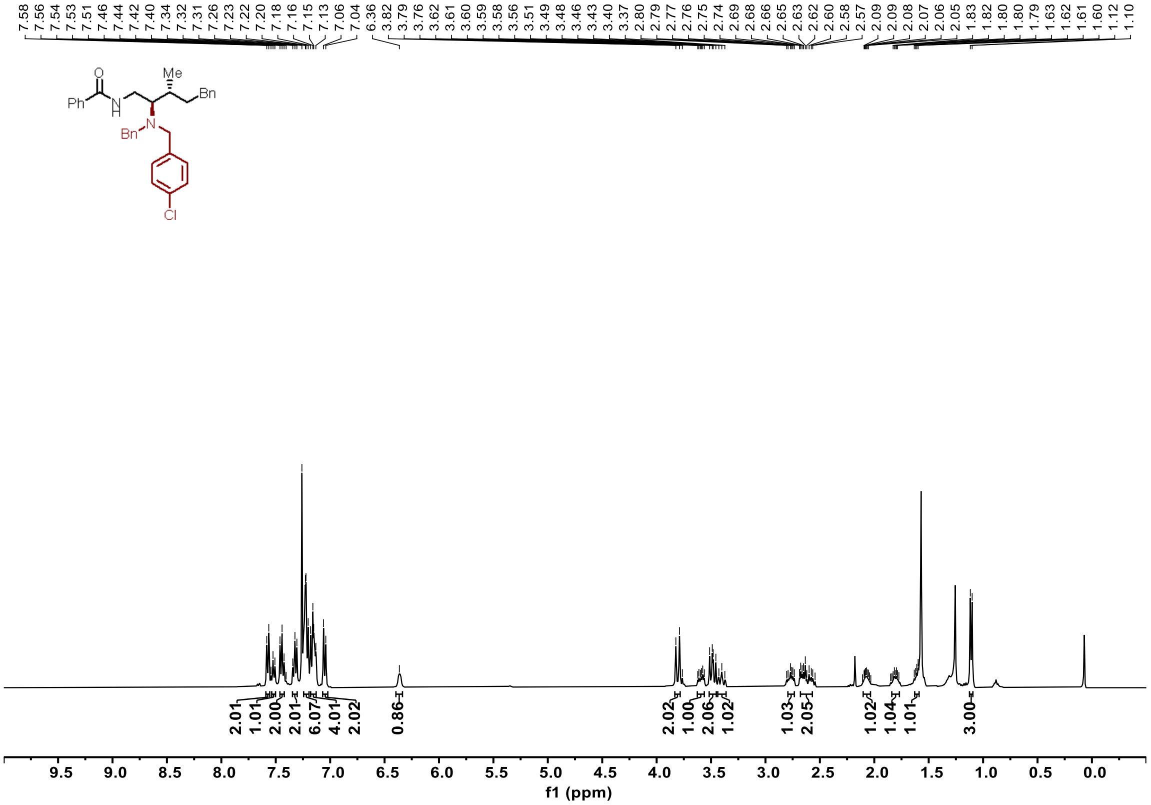

^{1}**H NMR** (400 MHz, CDCl$_3$) spectra of **3u**

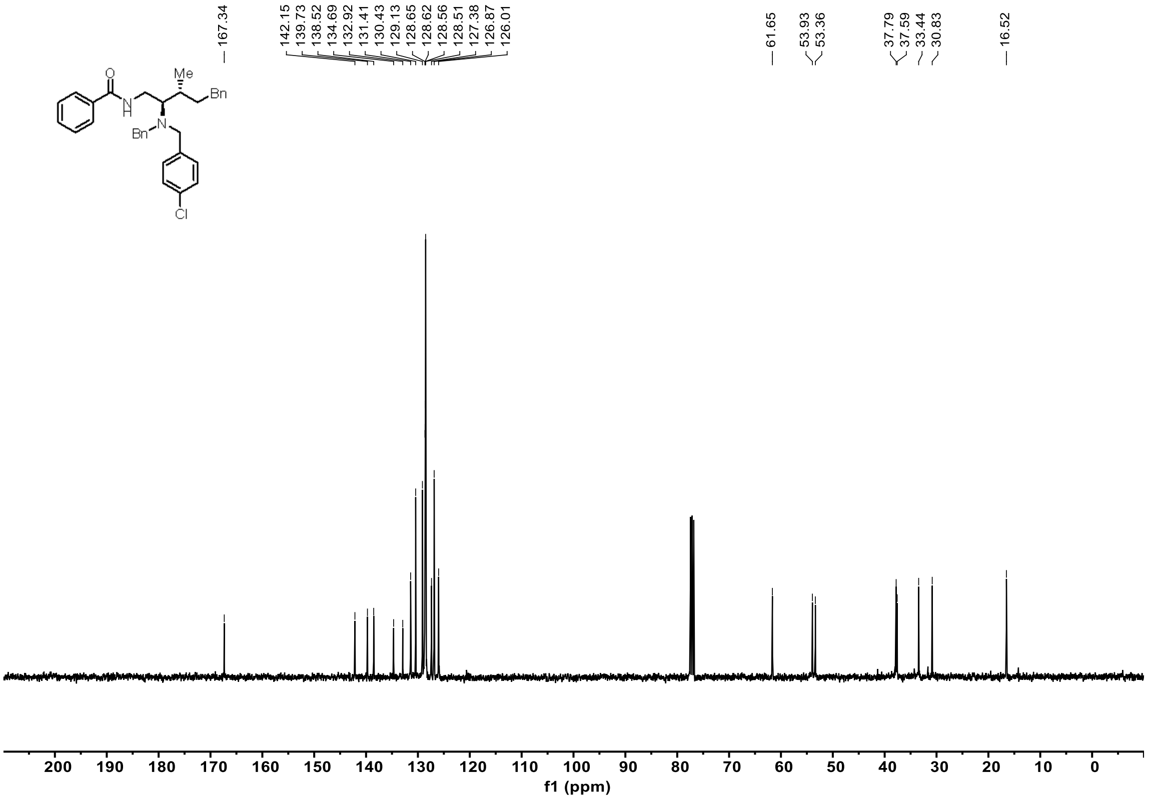

^{13}C NMR (101 MHz, $CDCl_3$) spectra of **3u**

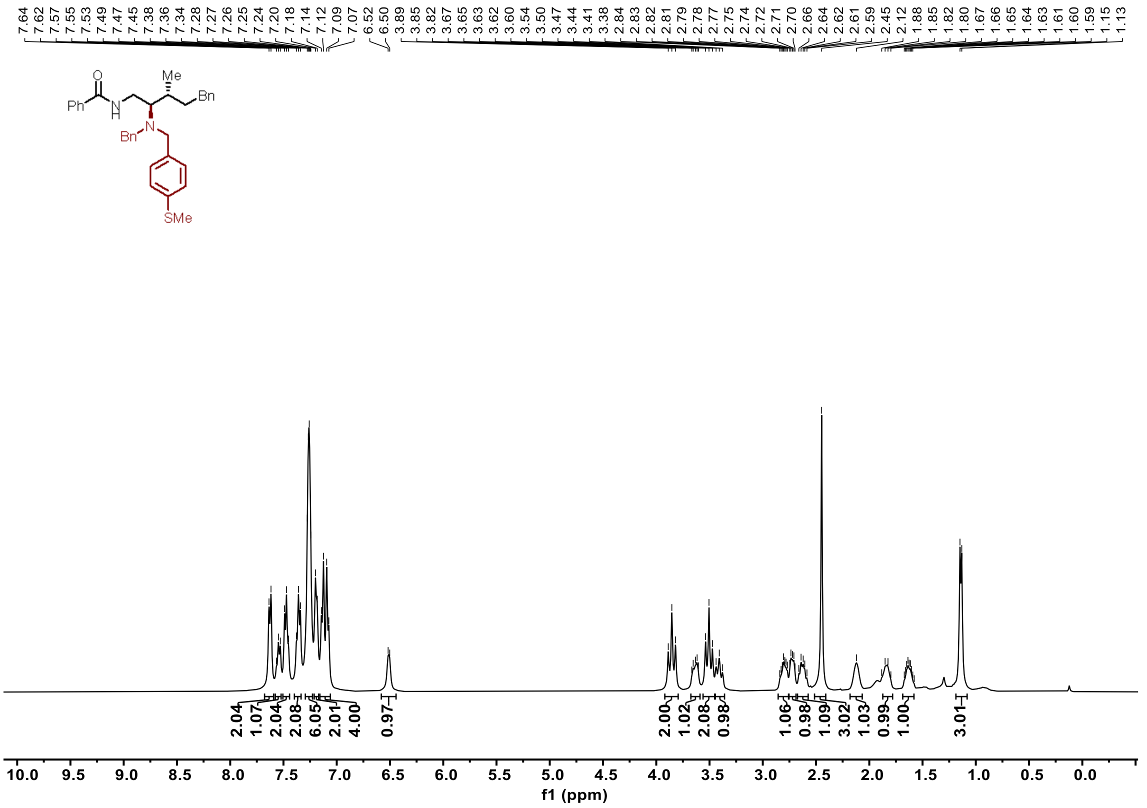

^{1}H NMR (400 MHz, $CDCl_3$) spectra of **3v**

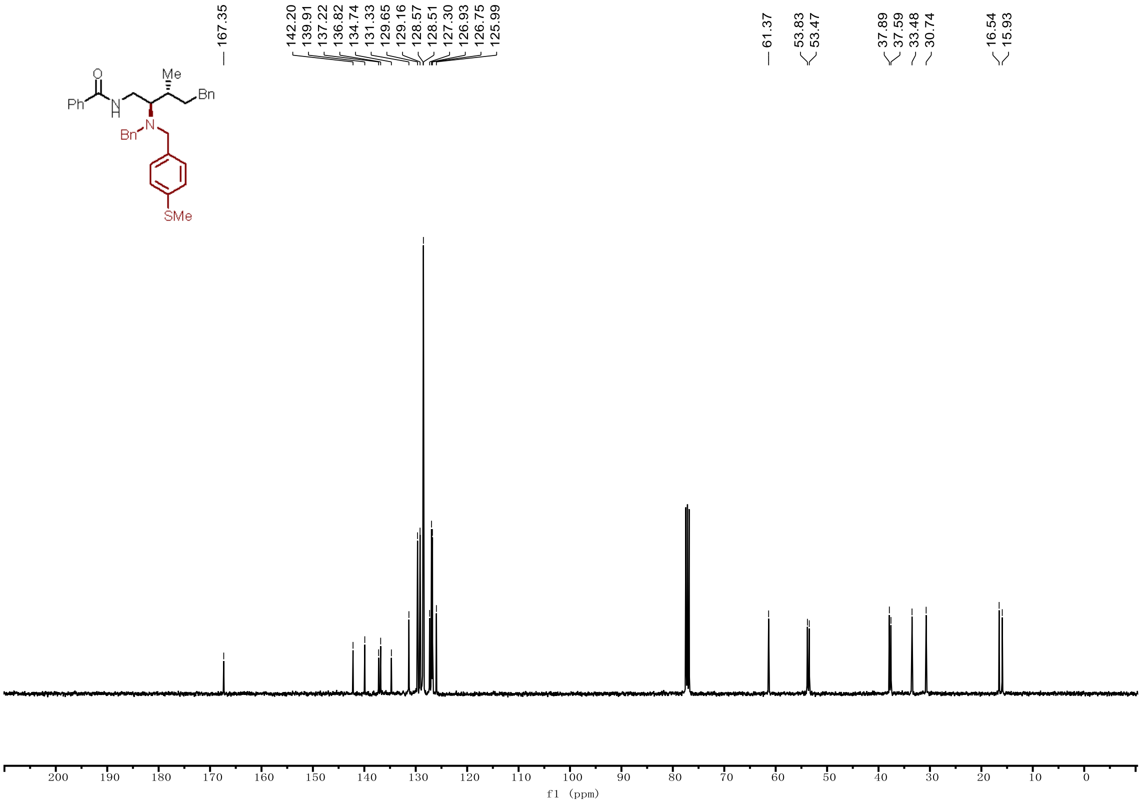

^{13}**C NMR** (101 MHz, CDCl$_3$) spectra of **3v**

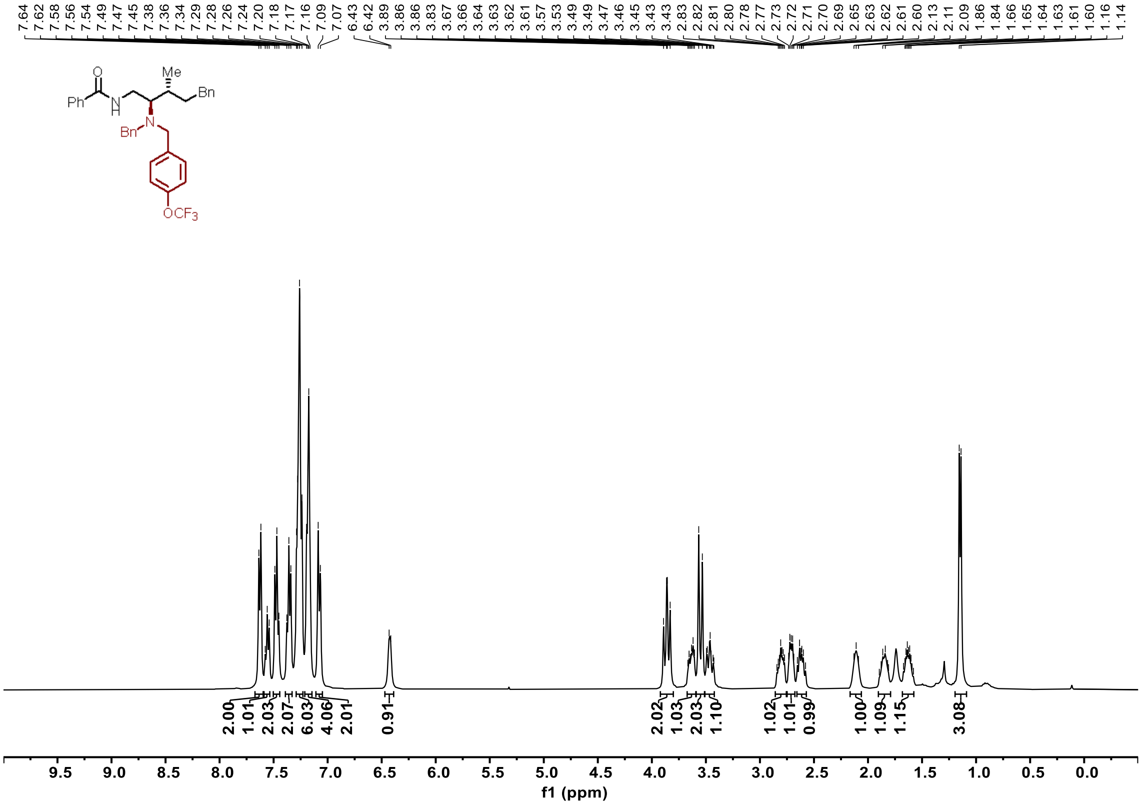

^{1}**H NMR** (400 MHz, CDCl$_3$) spectra of **3w**

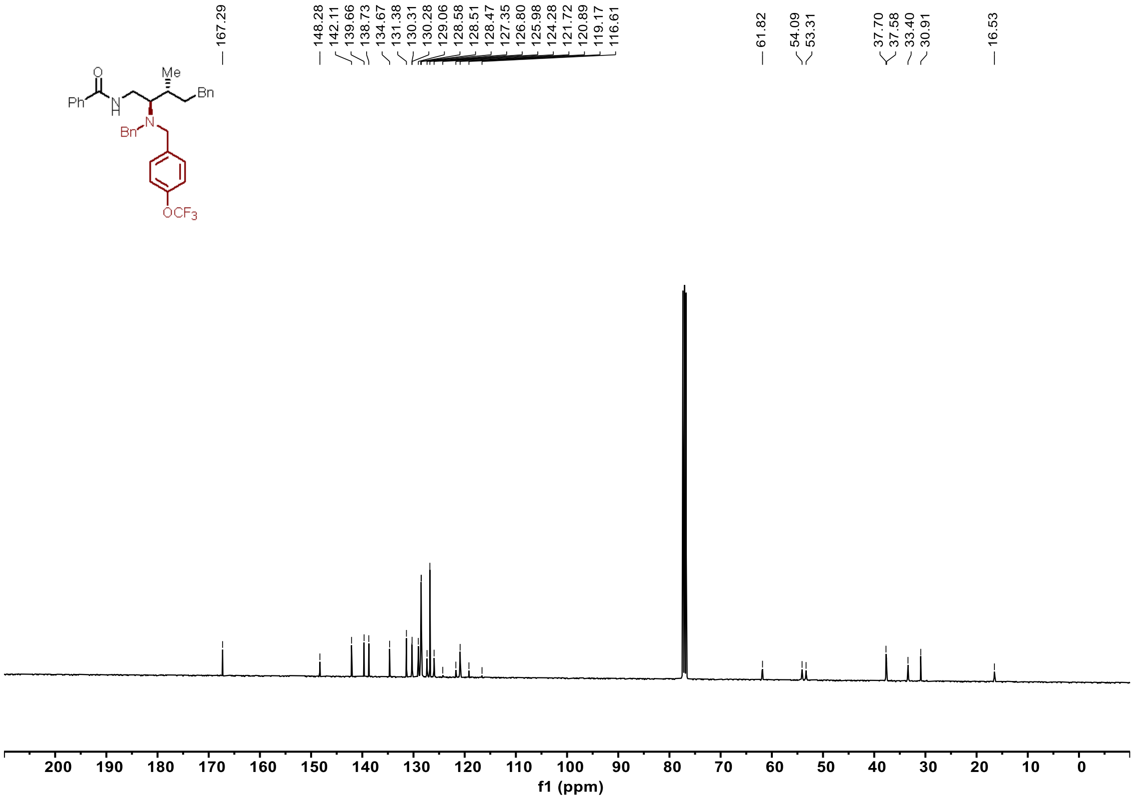

^{13}C NMR (101 MHz, $CDCl_3$) spectra of **3w**

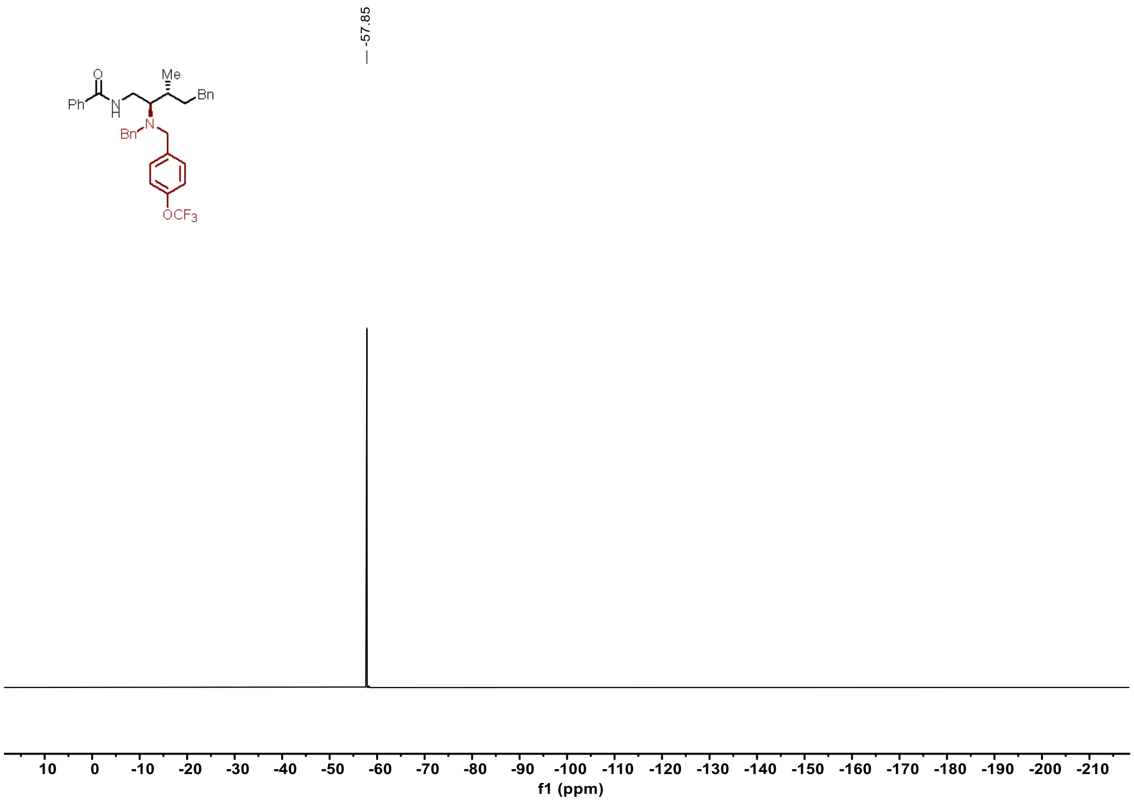

^{19}F NMR (376 MHz, $CDCl_3$) spectra of **3w**

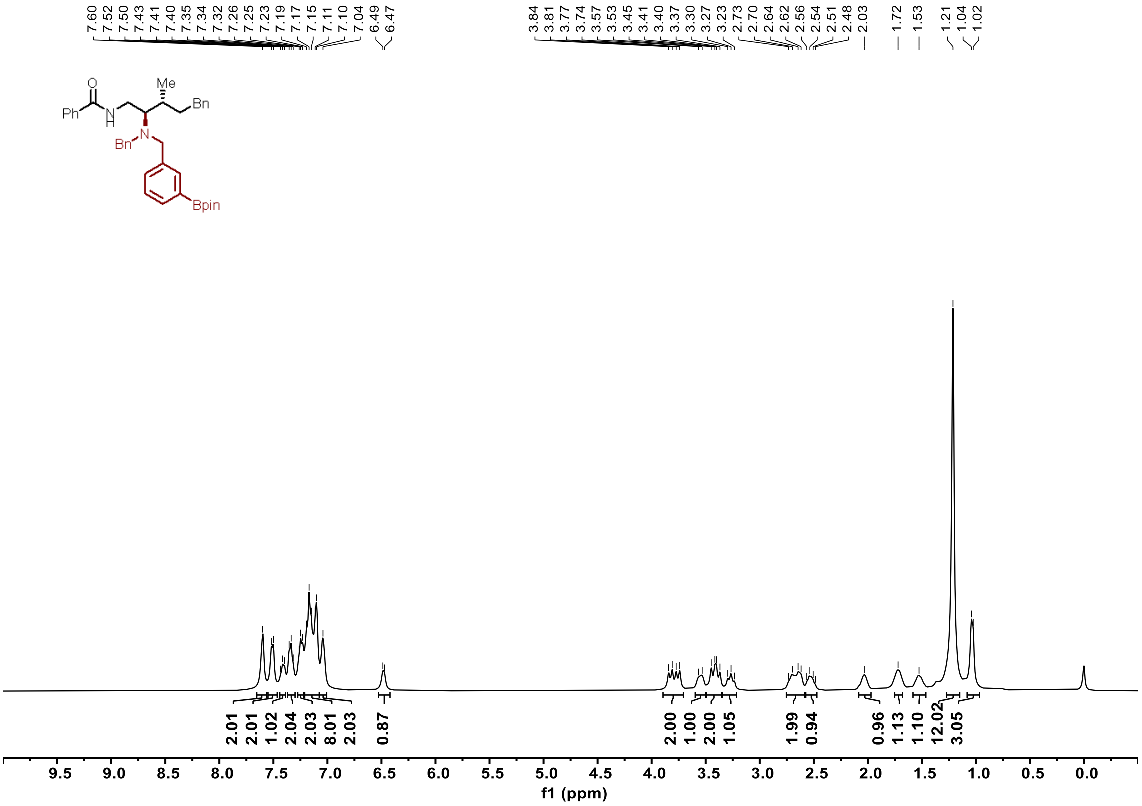

^{1}H NMR (400 MHz, $CDCl_3$) spectra of **3x**

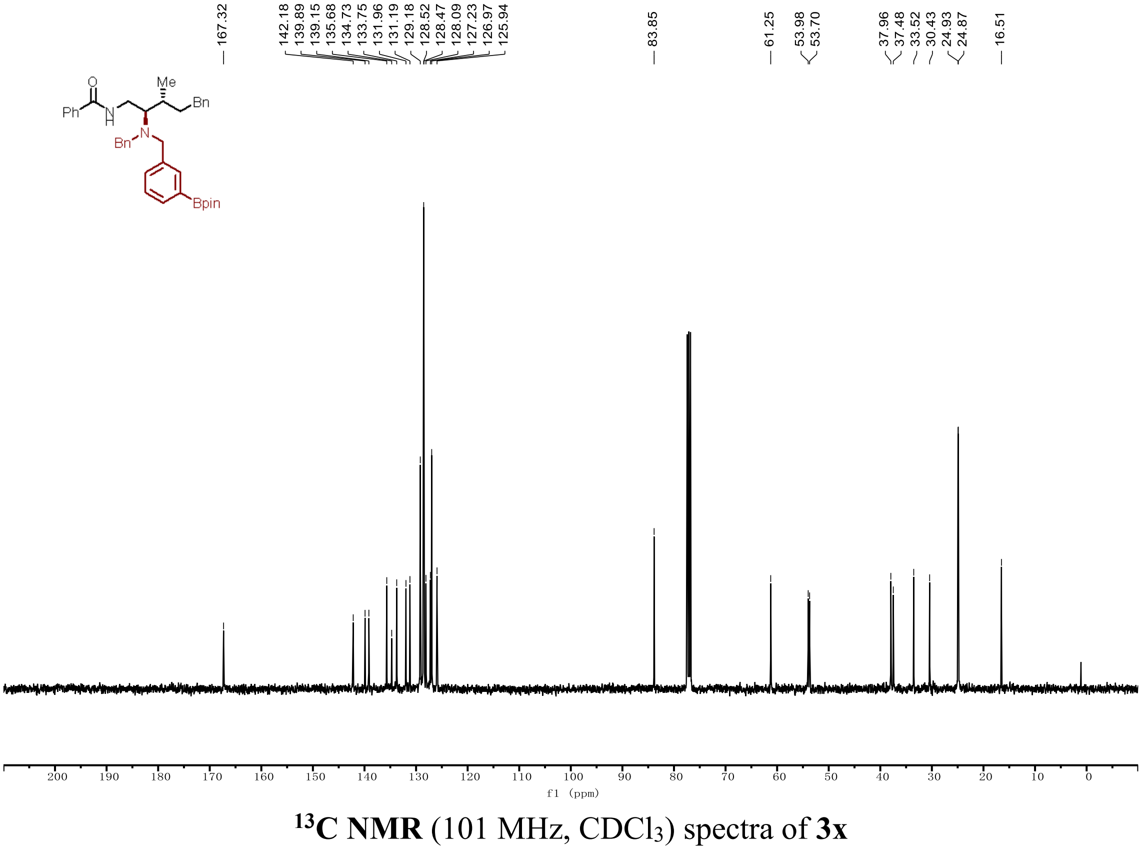

^{13}C NMR (101 MHz, $CDCl_3$) spectra of **3x**

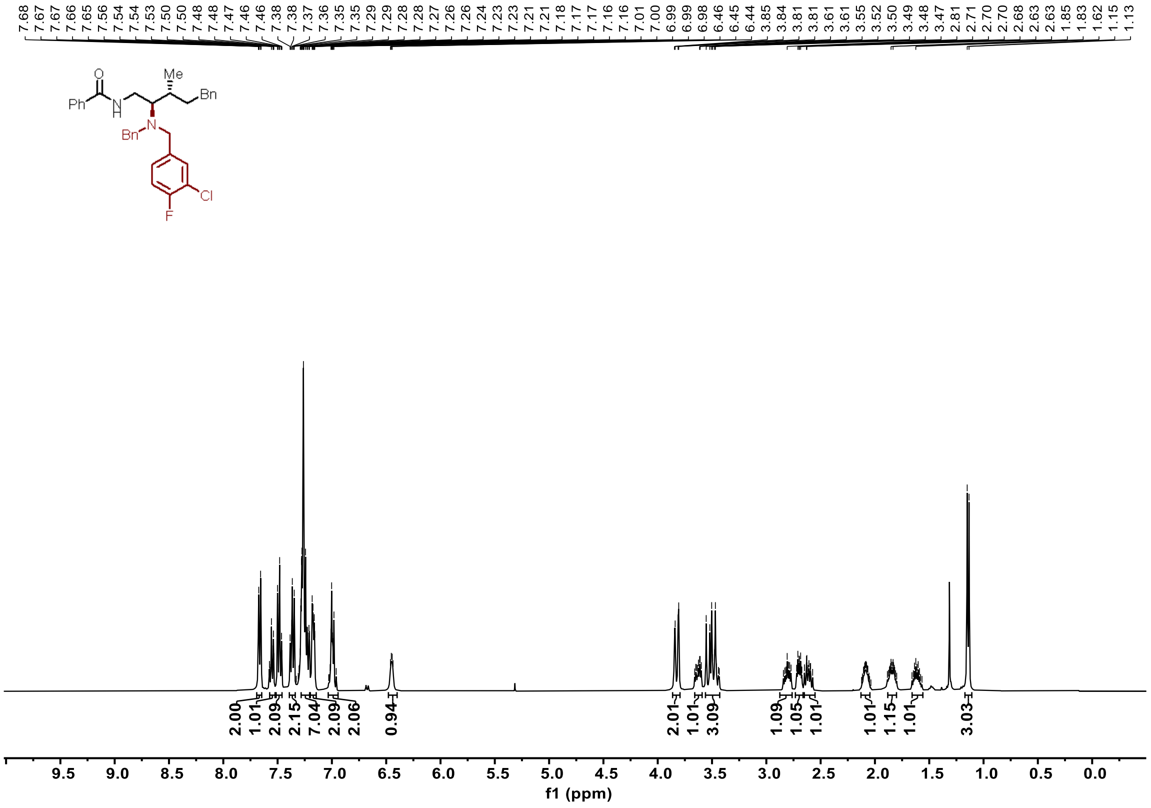

^{1}**H NMR** (400 MHz, $CDCl_3$) spectra of **3y**

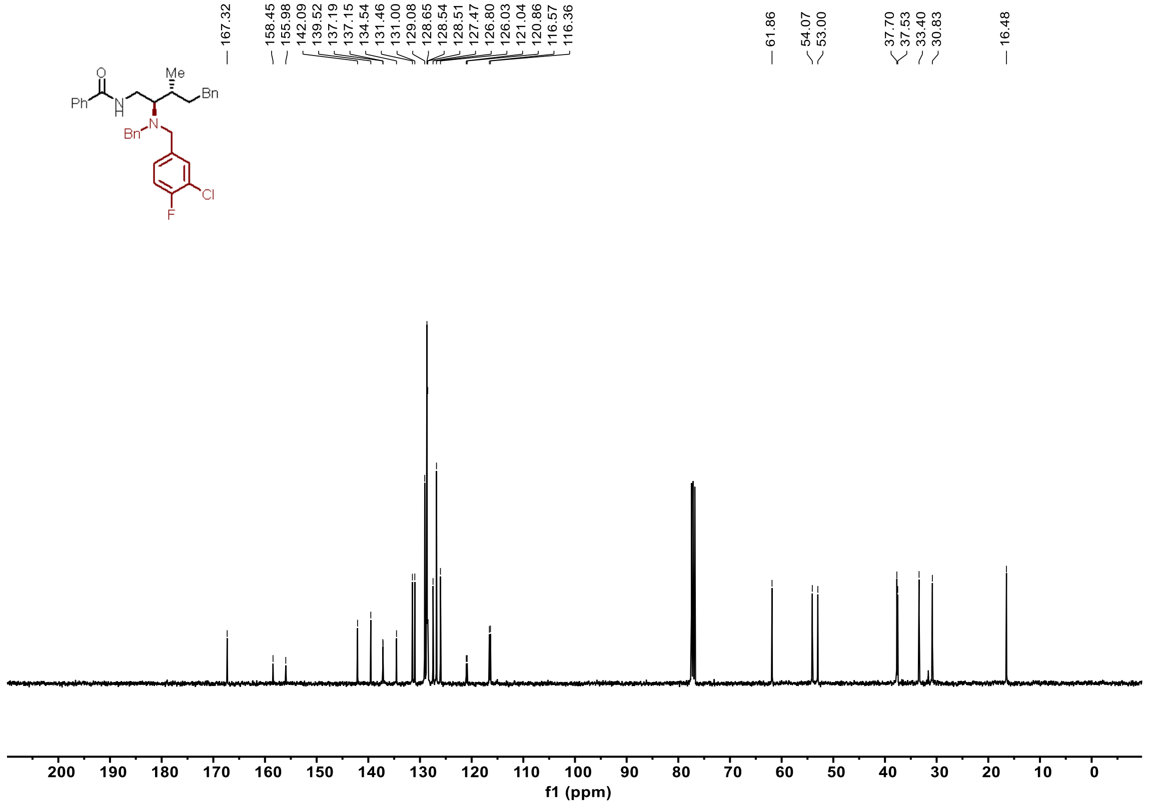

^{13}**C NMR** (101 MHz, $CDCl_3$) spectra of **3y**

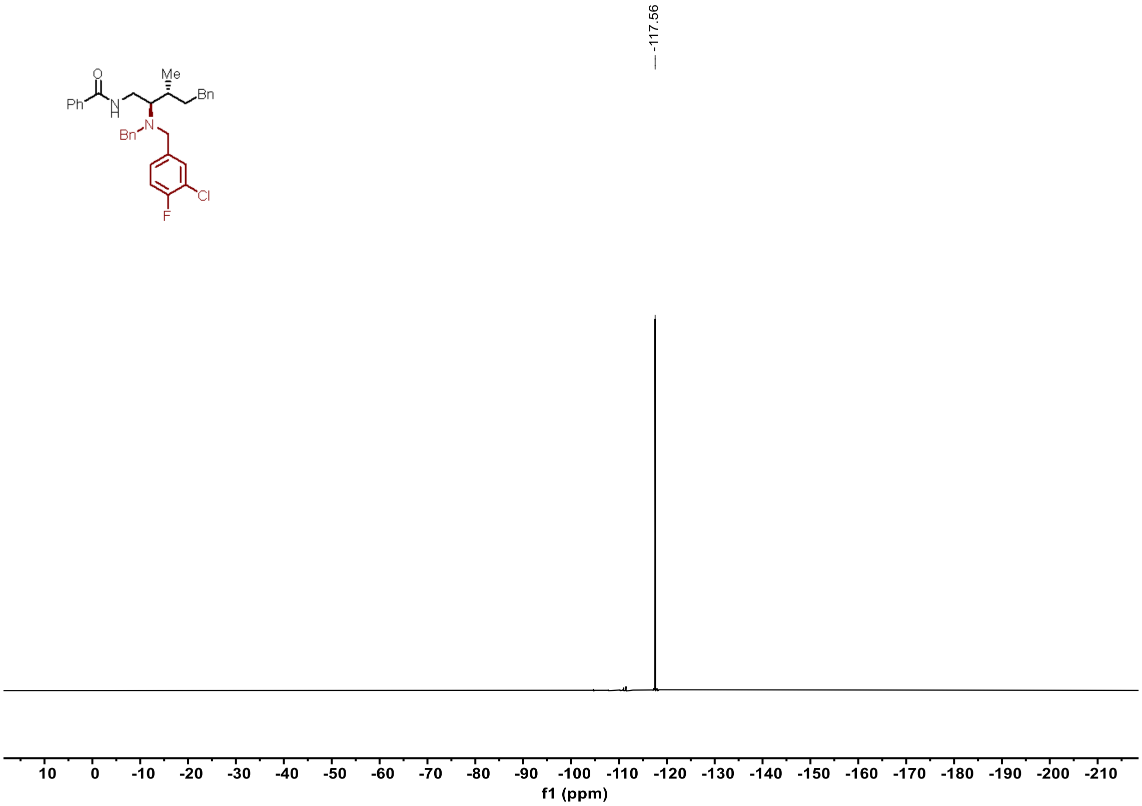

^{19}F NMR (376 MHz, $CDCl_3$) spectra of **3y**

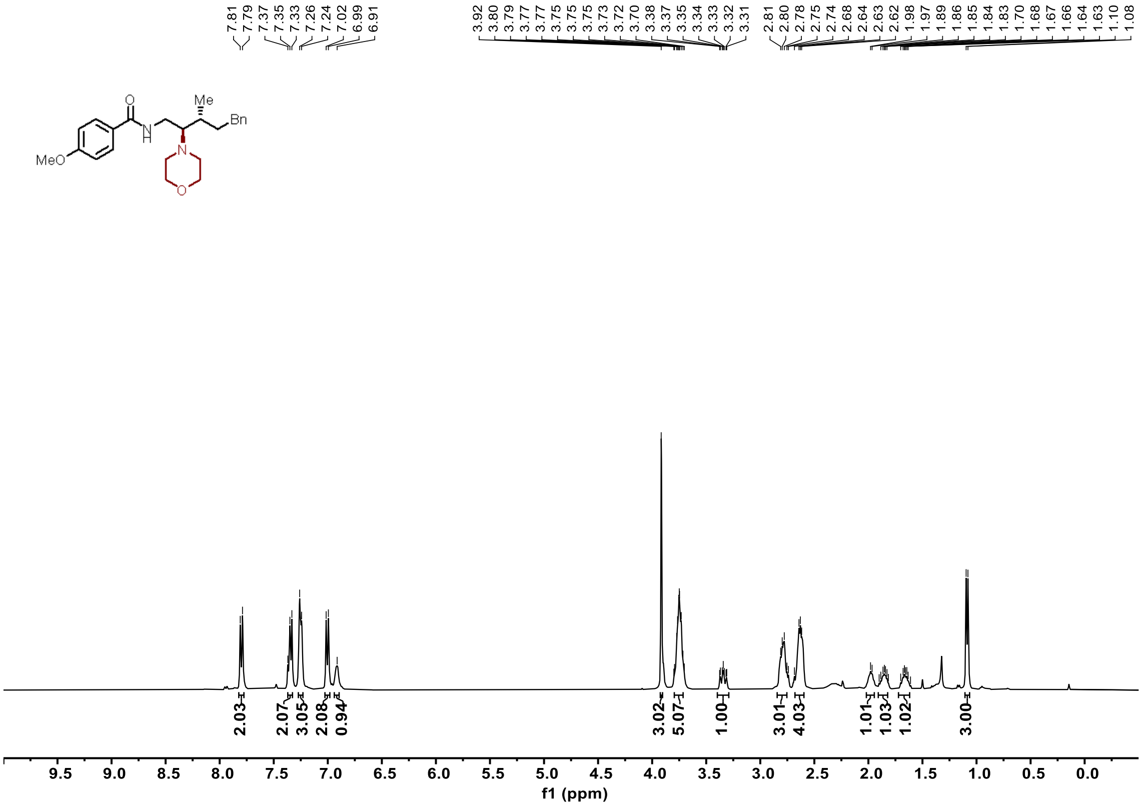

^{1}**H NMR** (400 MHz, CDCl$_3$) spectra of **4a**

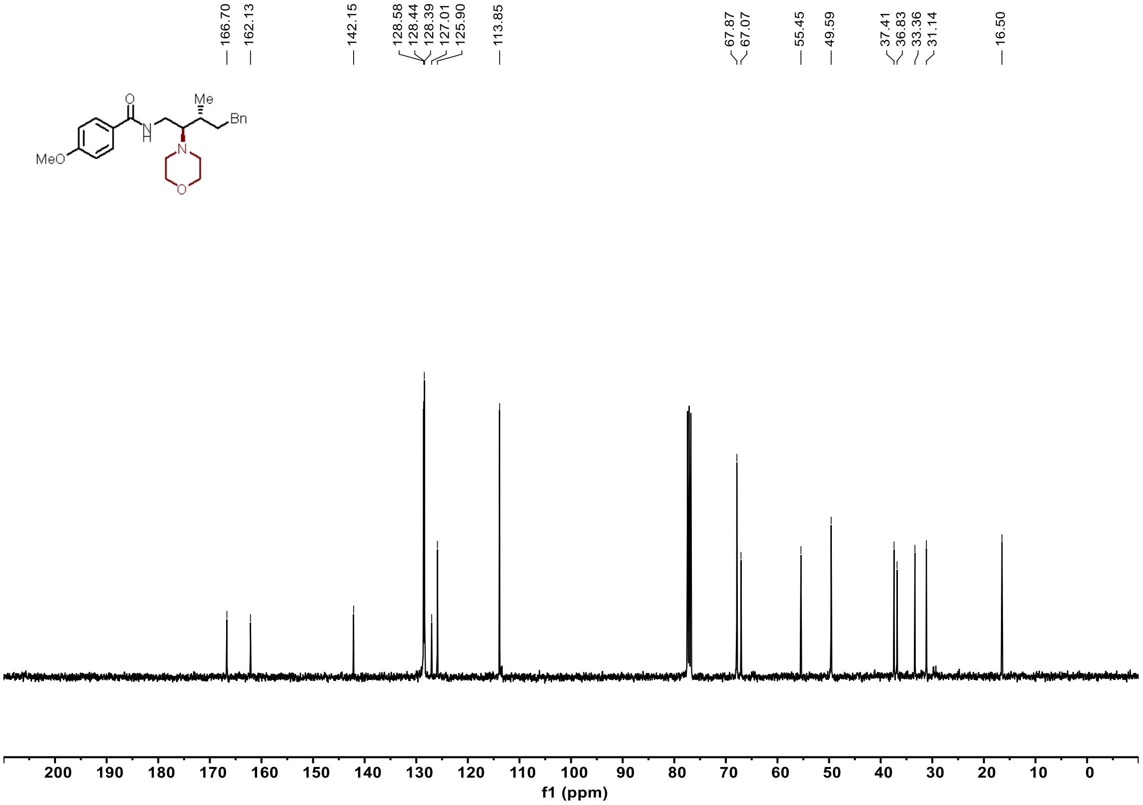

^{13}**C NMR** (101 MHz, CDCl$_3$) spectra of **4a**

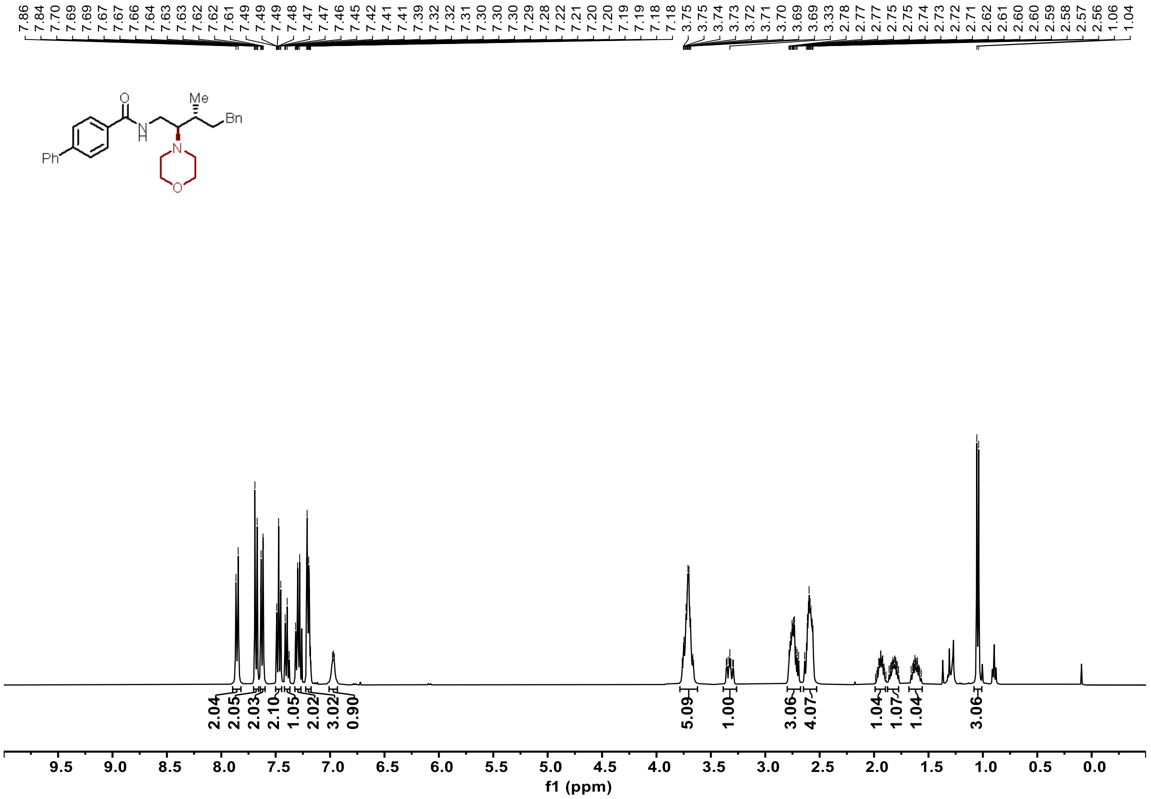

^{1}H NMR (400 MHz, $CDCl_3$) spectra of **4b**

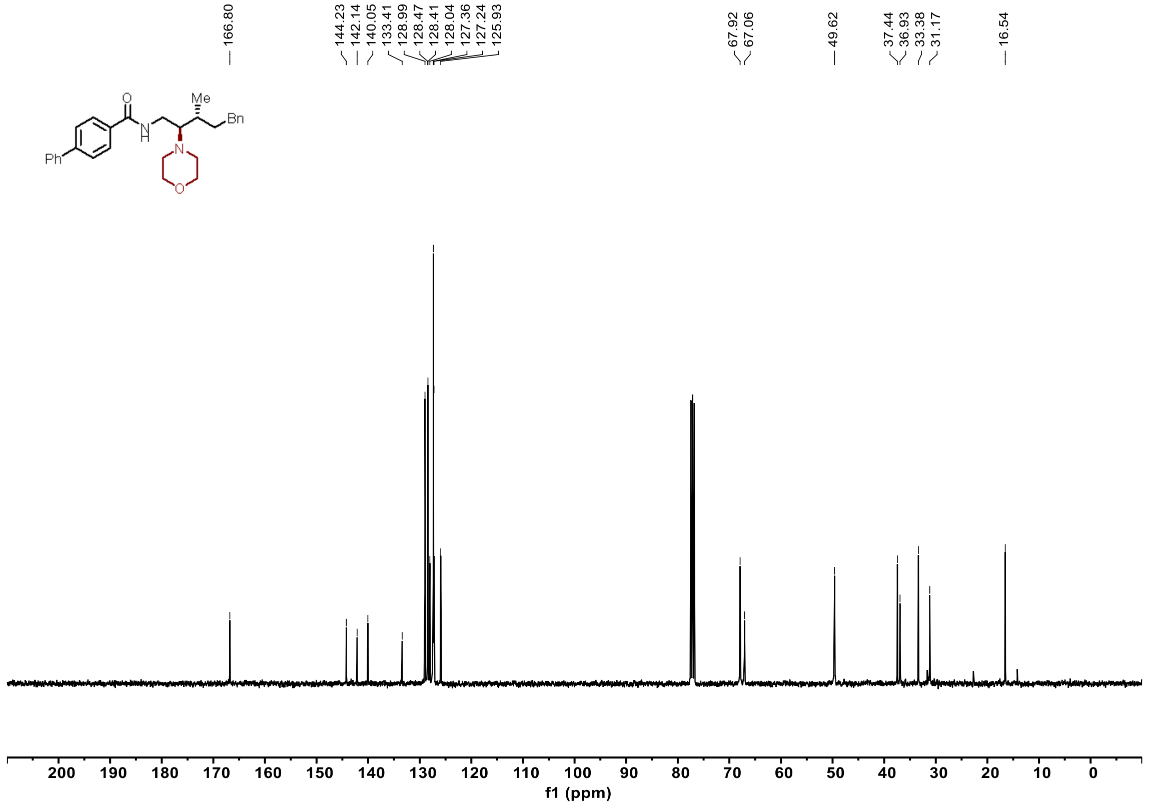

^{13}C NMR (101 MHz, $CDCl_3$) spectra of **4b**

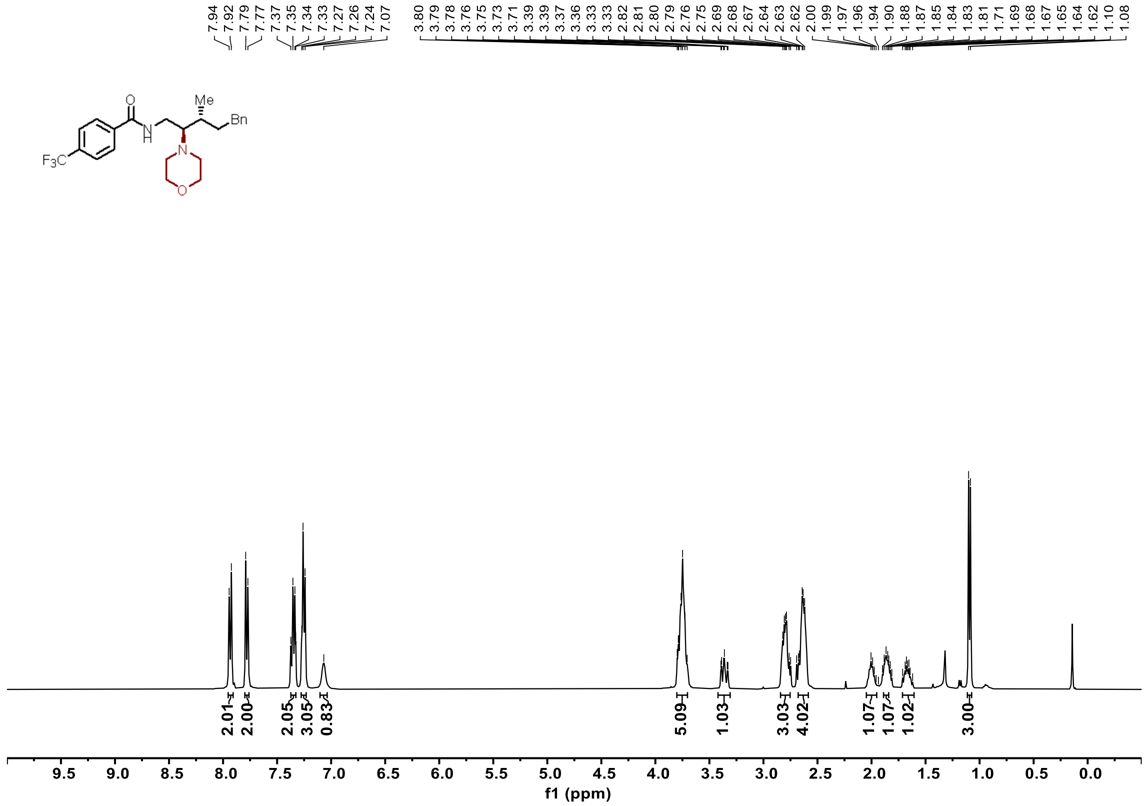

^{1}H NMR (400 MHz, $CDCl_3$) spectra of **4c**

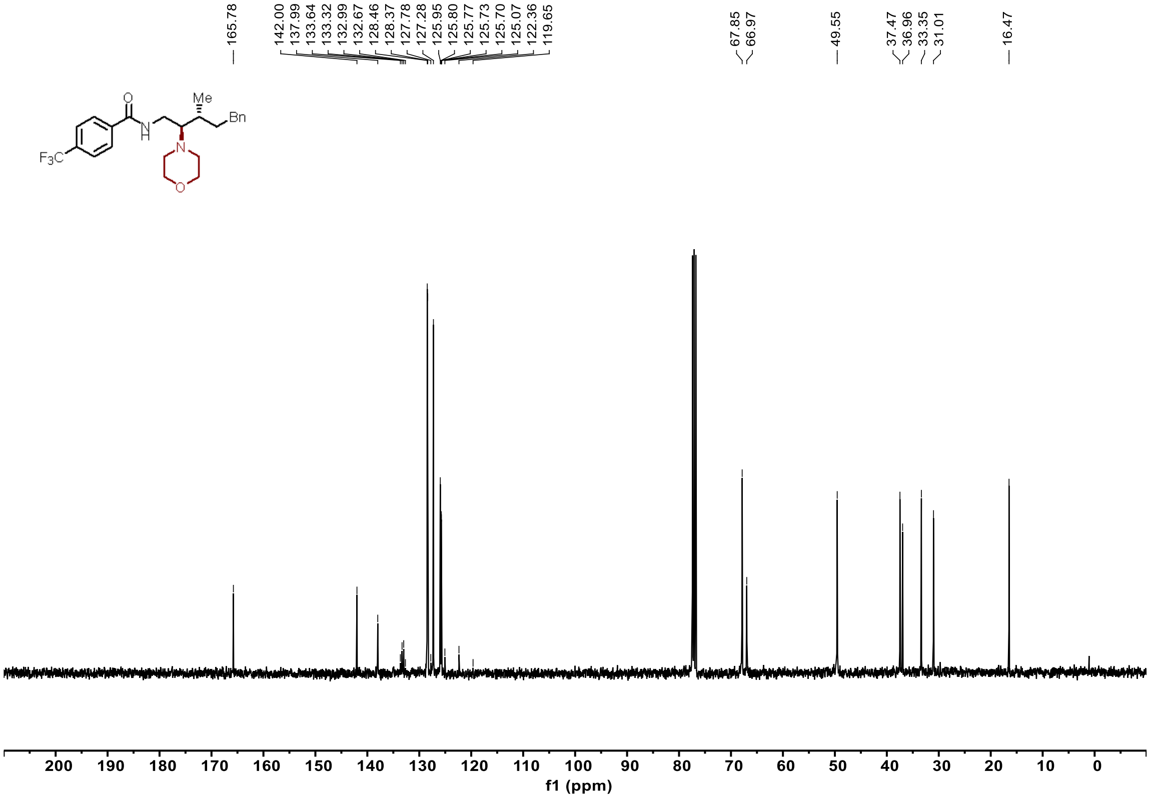

^{13}C NMR (101 MHz, $CDCl_3$) spectra of **4c**

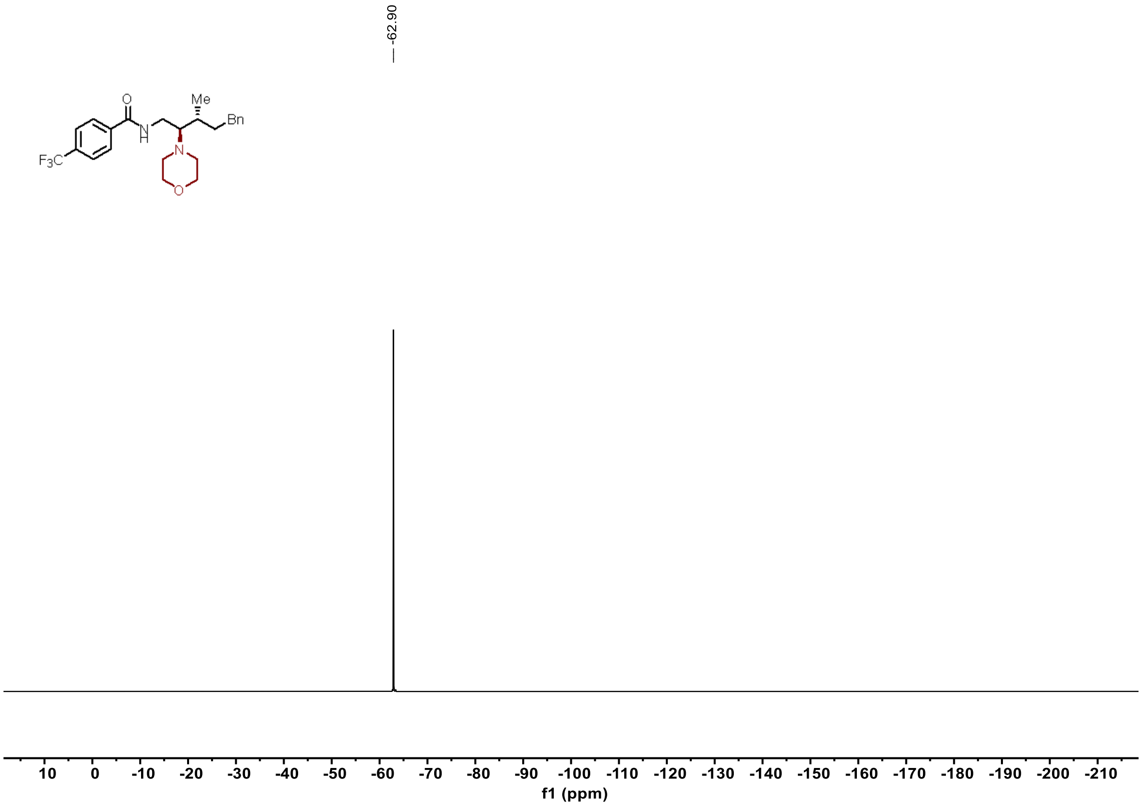

^{19}F NMR (376 MHz, $CDCl_3$) spectra of **4c**

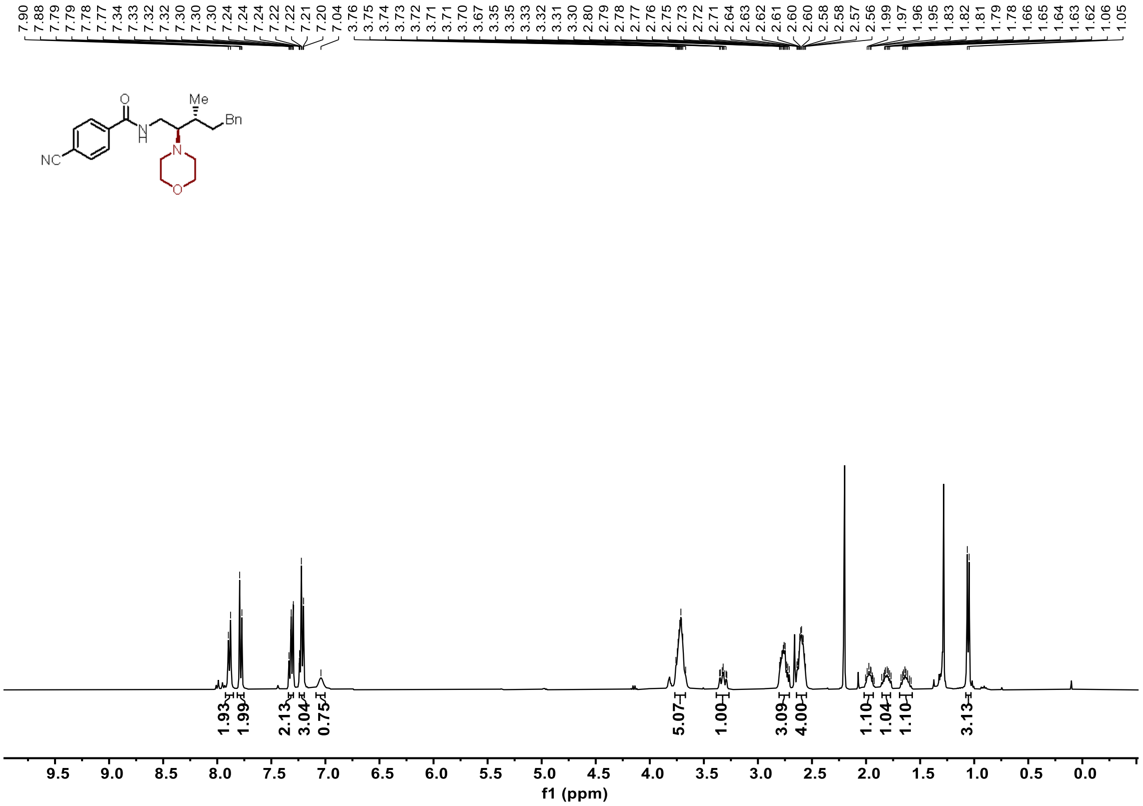

^{1}H NMR (400 MHz, $CDCl_3$) spectra of **4d**

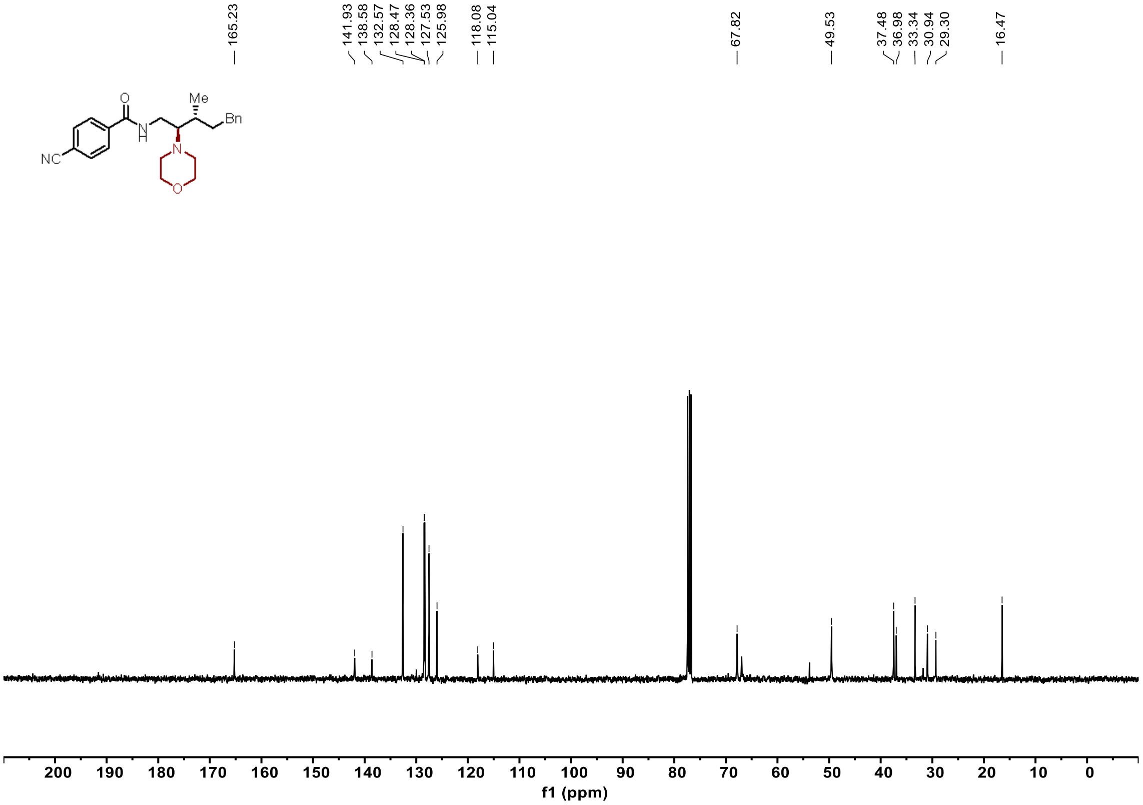

^{13}C NMR (101 MHz, $CDCl_3$) spectra of **4d**

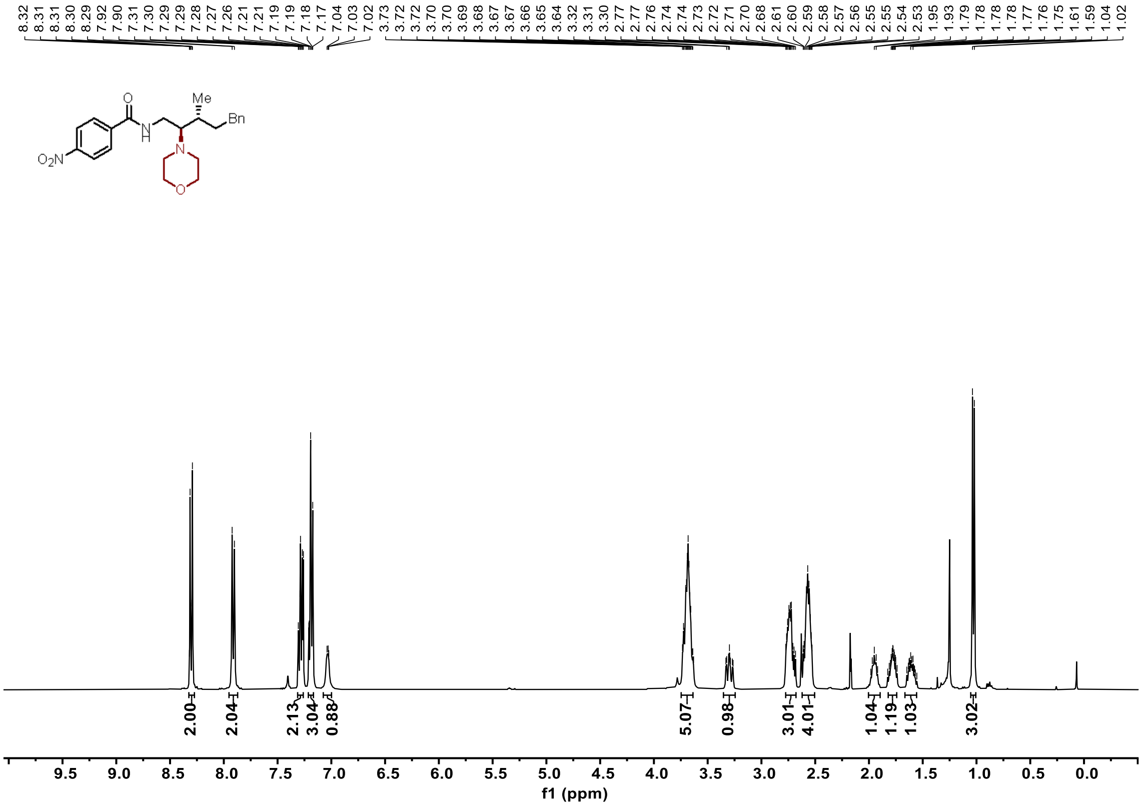

^{1}H NMR (400 MHz, $CDCl_3$) spectra of **4e**

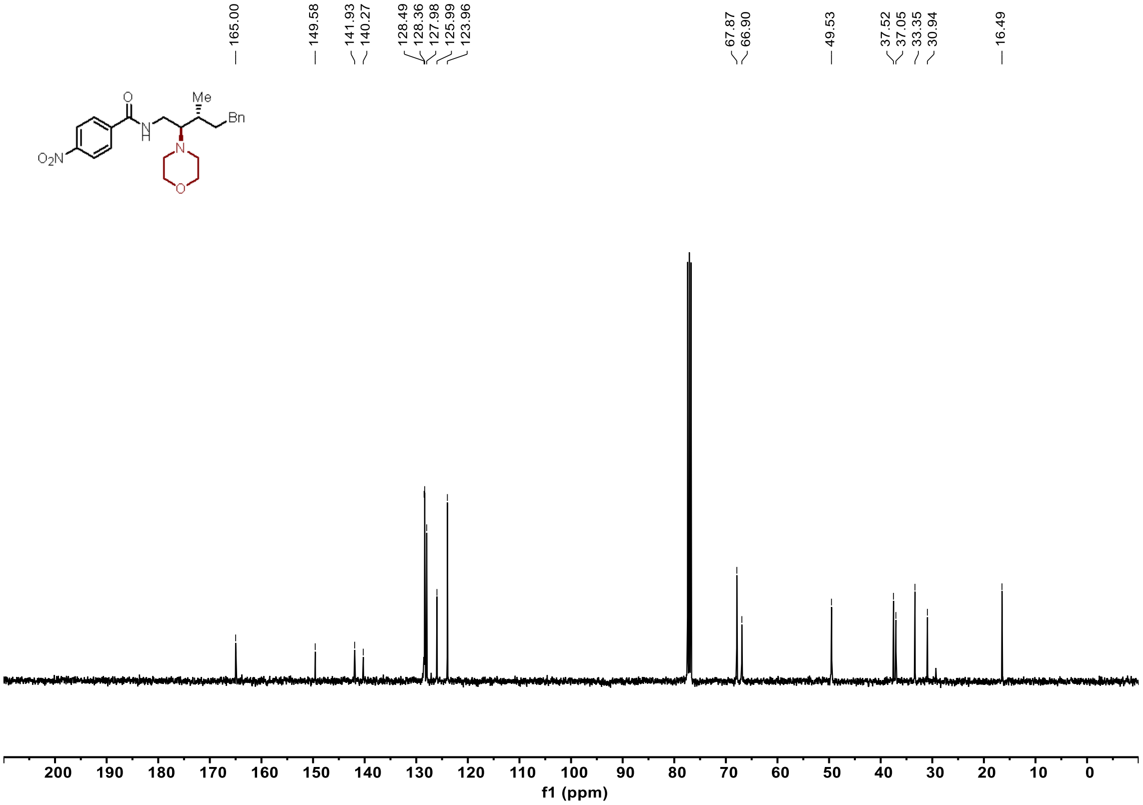

^{13}C NMR (101 MHz, $CDCl_3$) spectra of **4e**

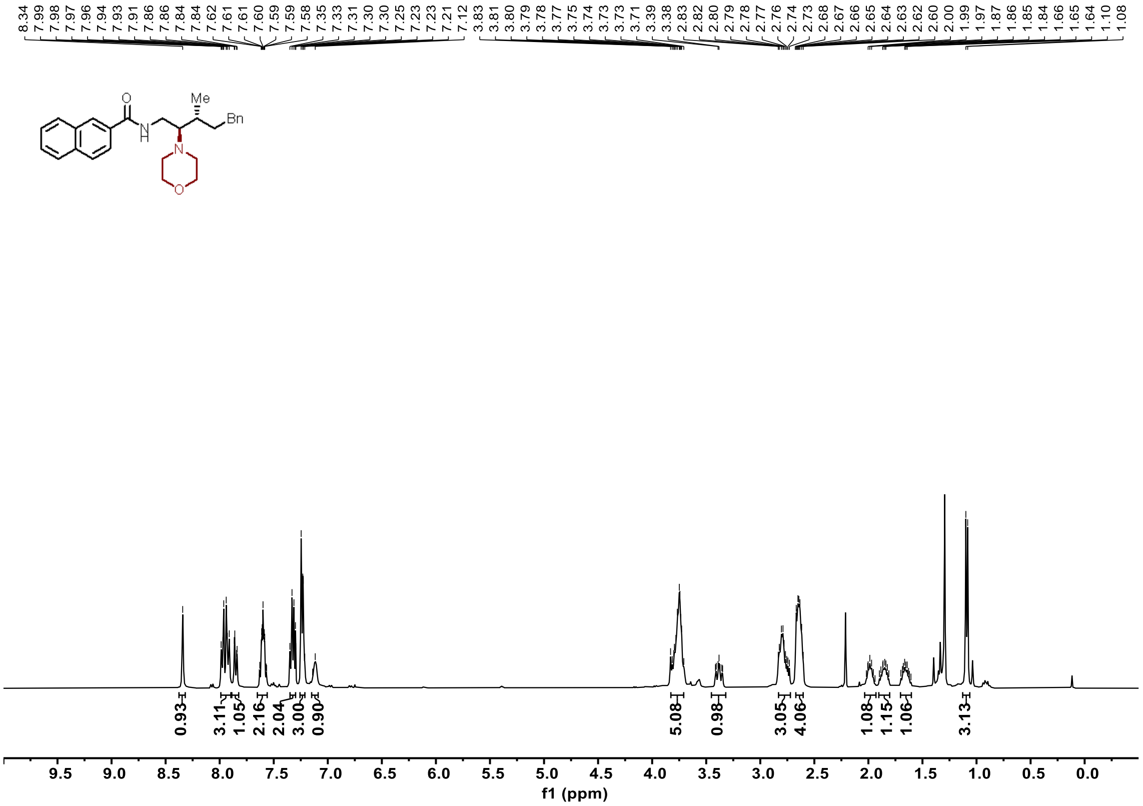

^{1}H NMR (400 MHz, $CDCl_3$) spectra of **4f**

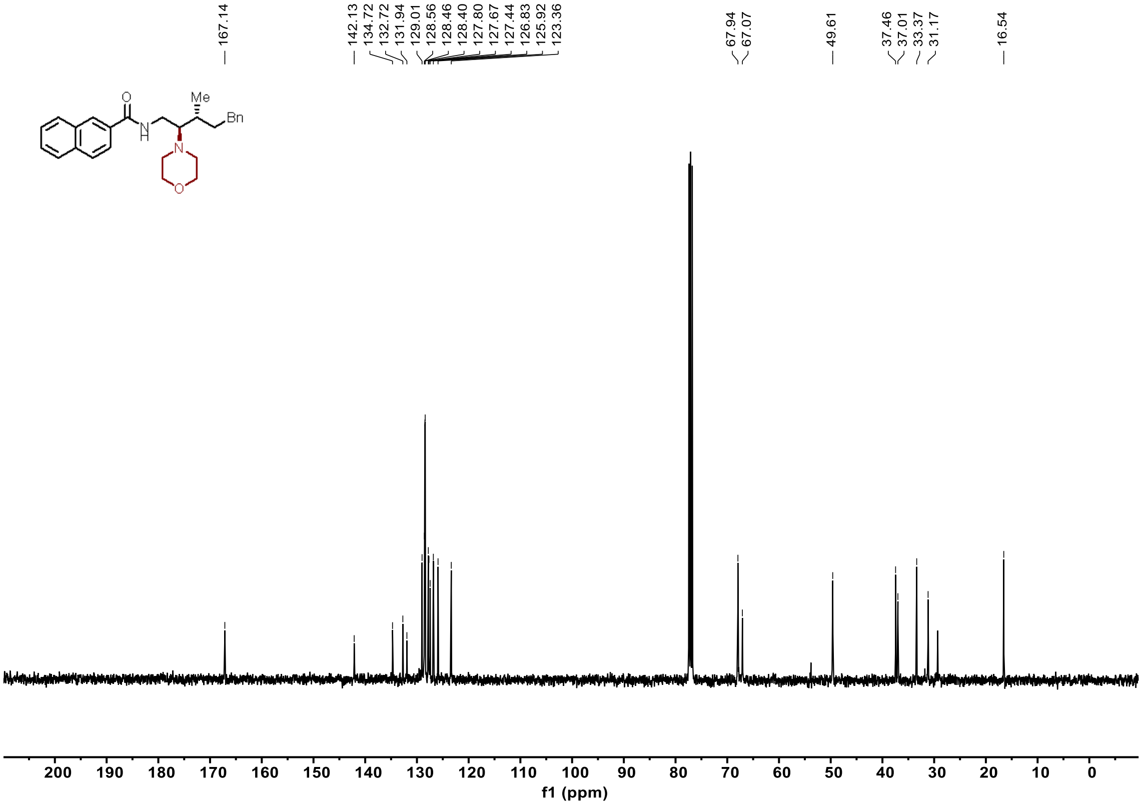

^{13}C NMR (101 MHz, $CDCl_3$) spectra of **4f**

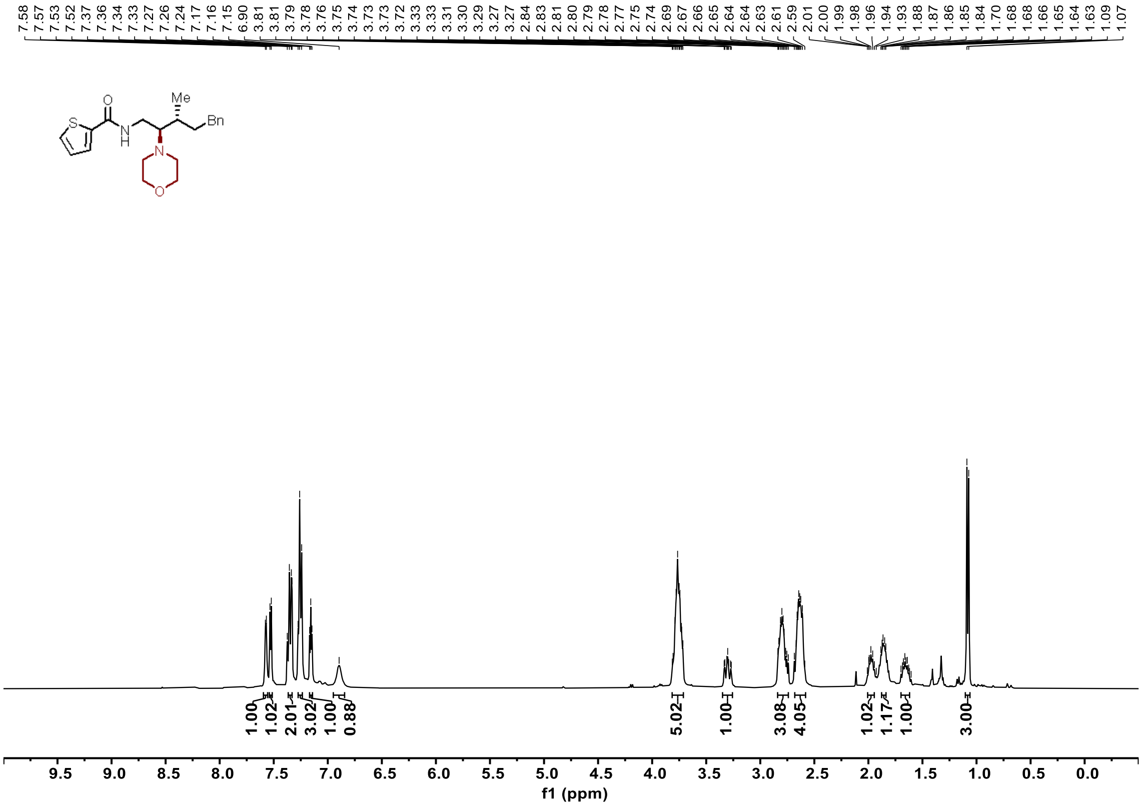

^{1}H NMR (400 MHz, $CDCl_3$) spectra of **4g**

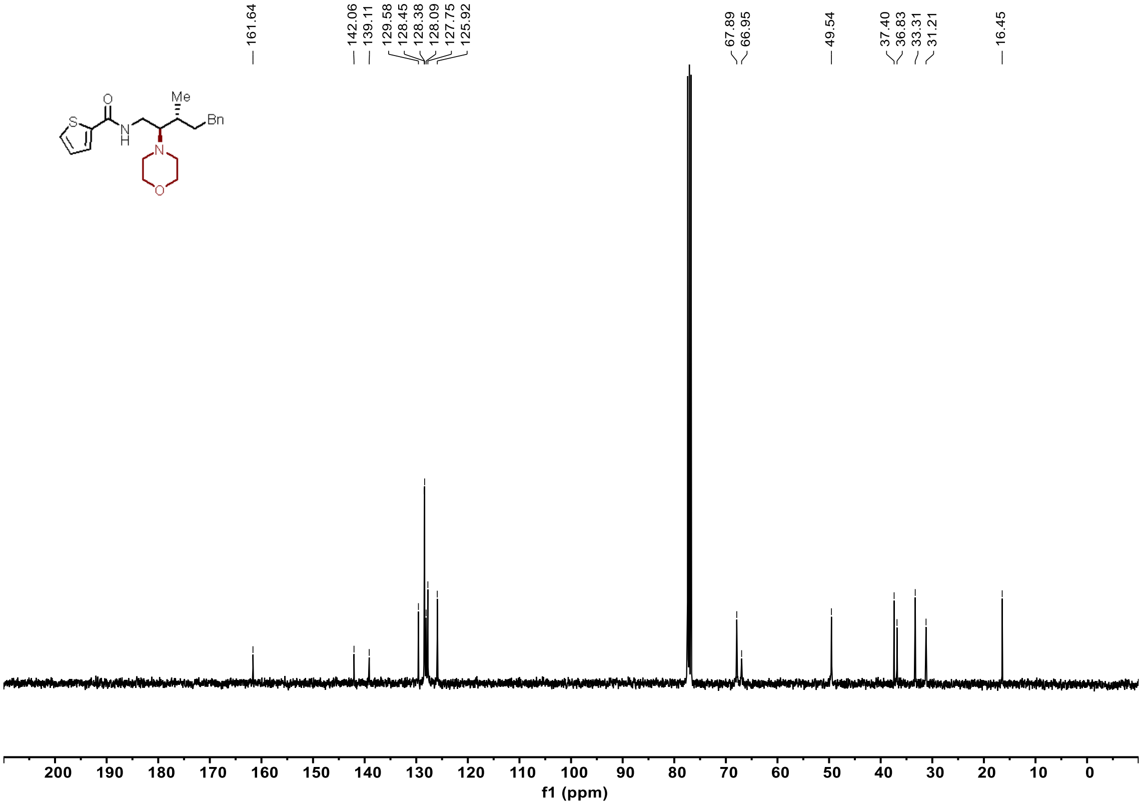

^{13}C NMR (101 MHz, $CDCl_3$) spectra of **4g**

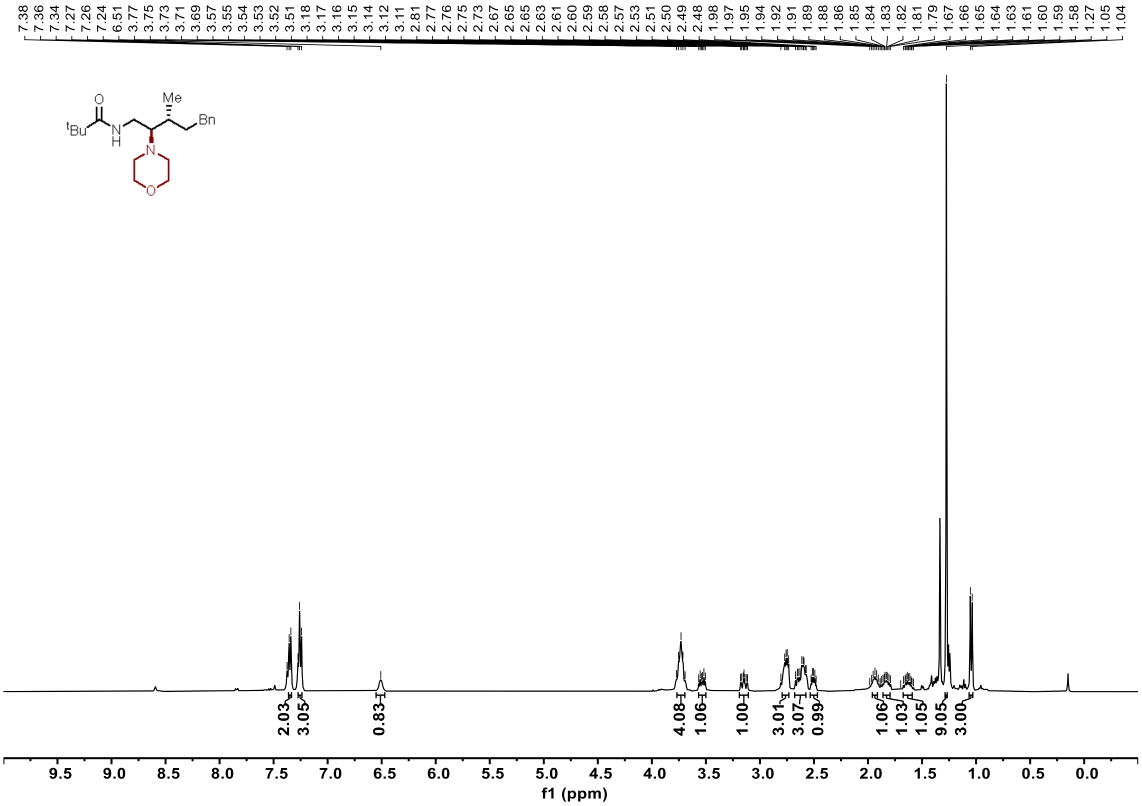

^{1}H NMR (400 MHz, $CDCl_3$) spectra of **4h**

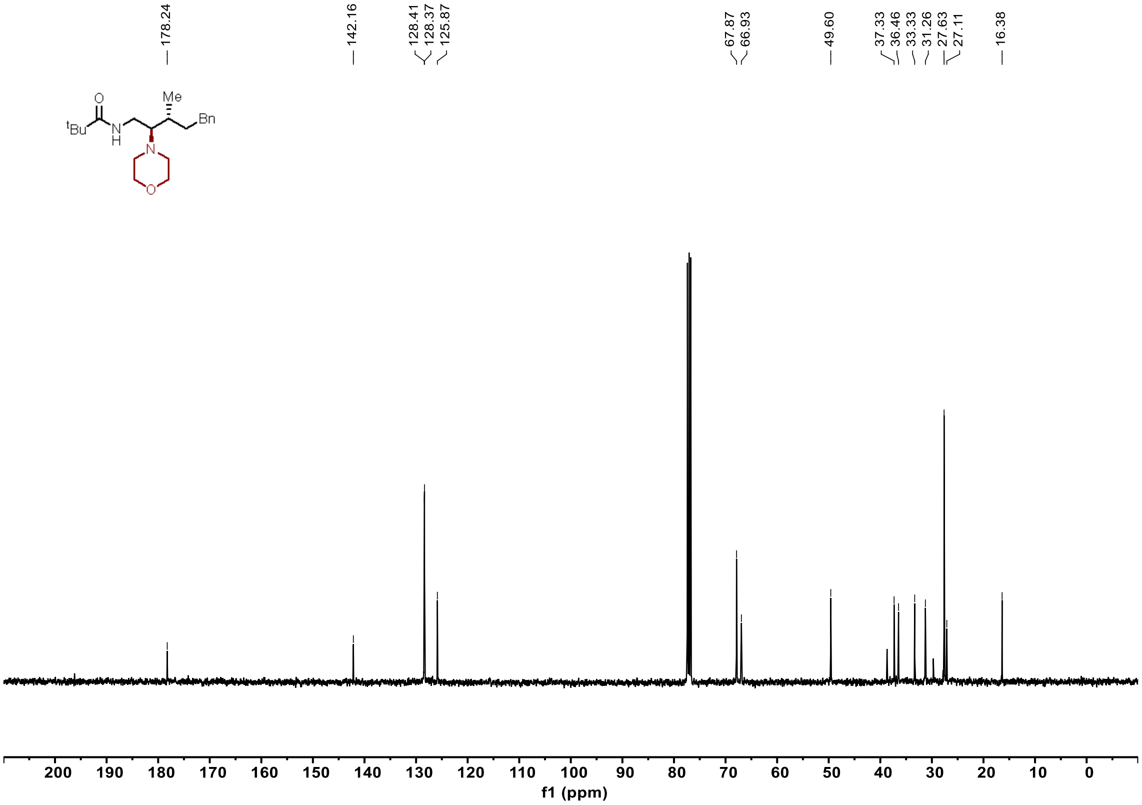

^{13}C NMR (101 MHz, $CDCl_3$) spectra of **4h**

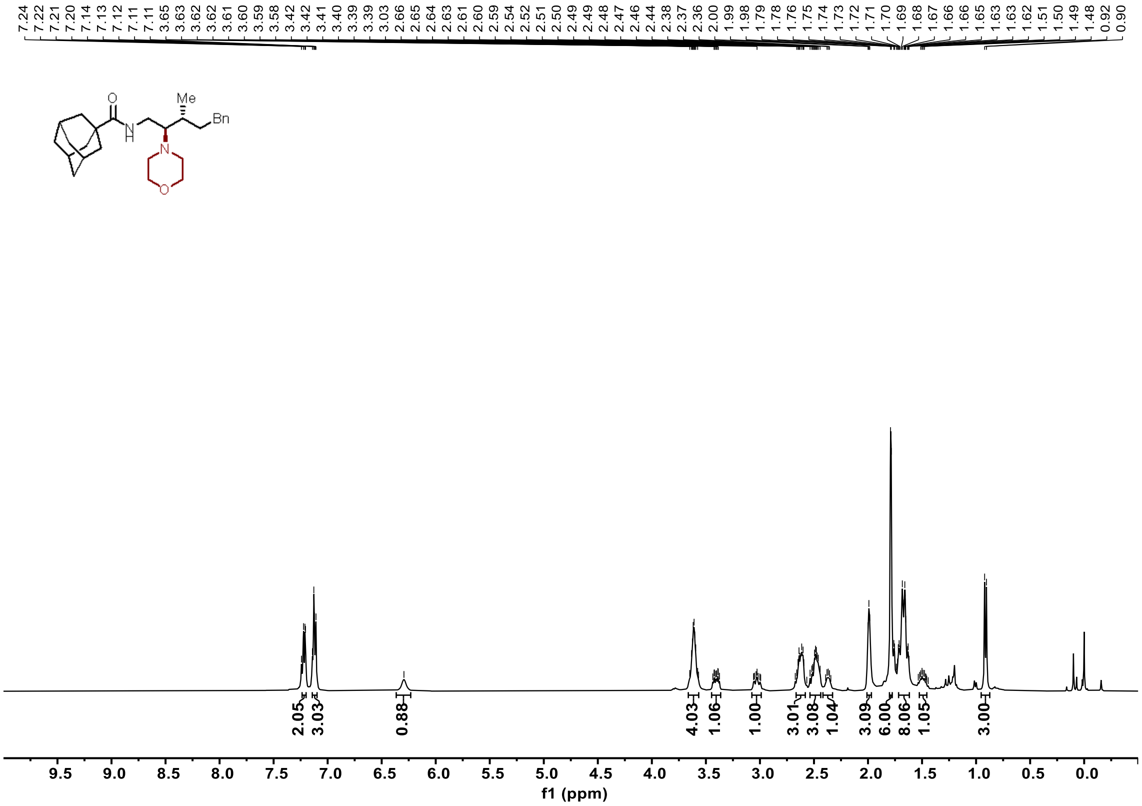

^{1}H NMR (400 MHz, $CDCl_3$) spectra of **4i**

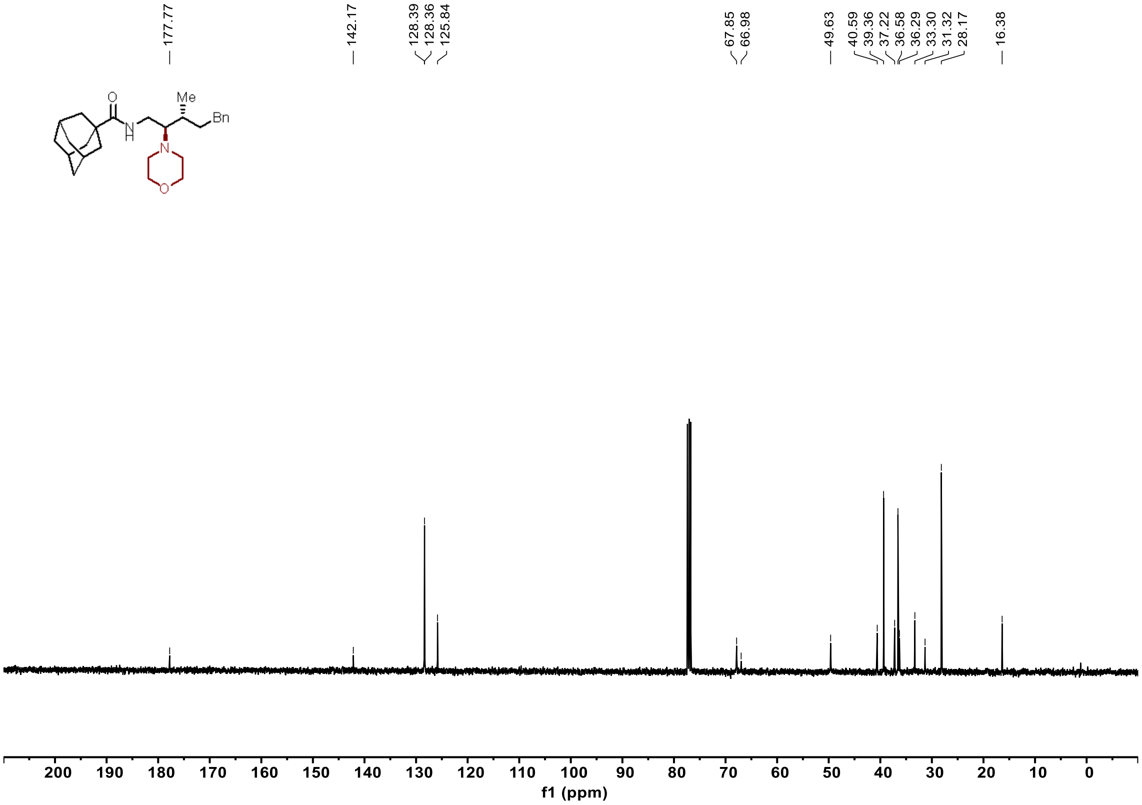

^{13}C NMR (101 MHz, $CDCl_3$) spectra of **4i**

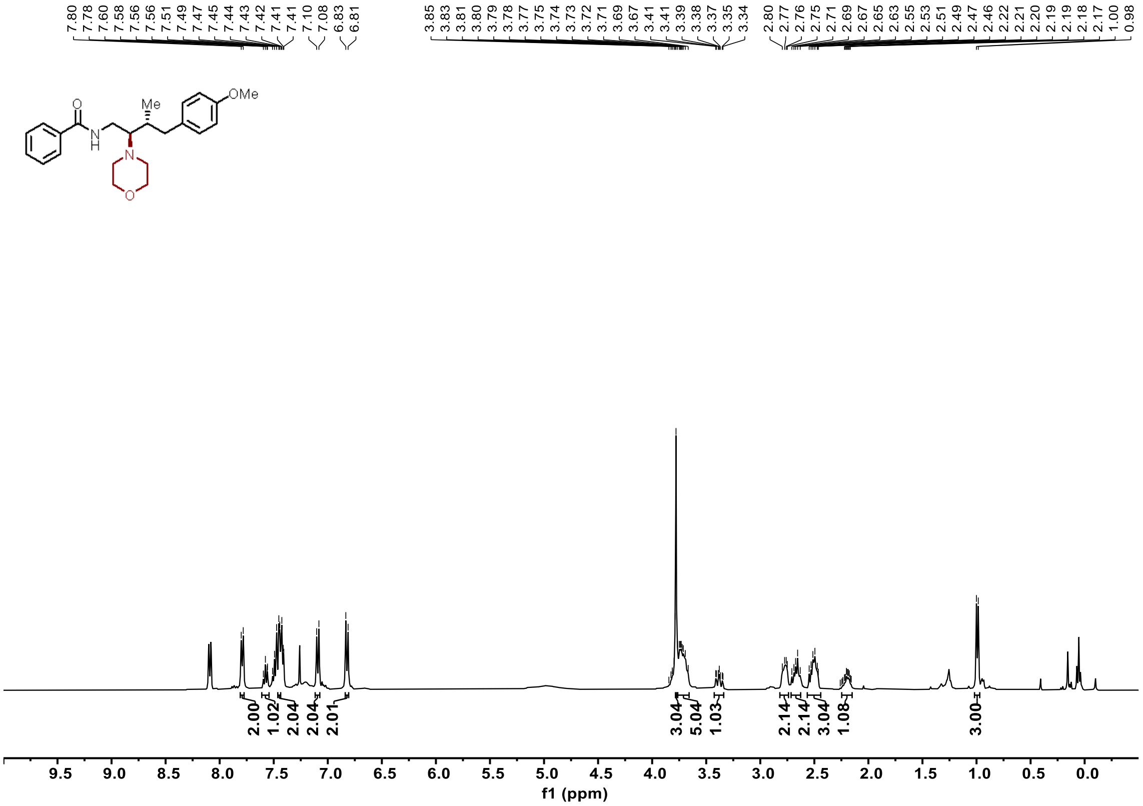

^{1}H NMR (400 MHz, $CDCl_3$) spectra of **4j**

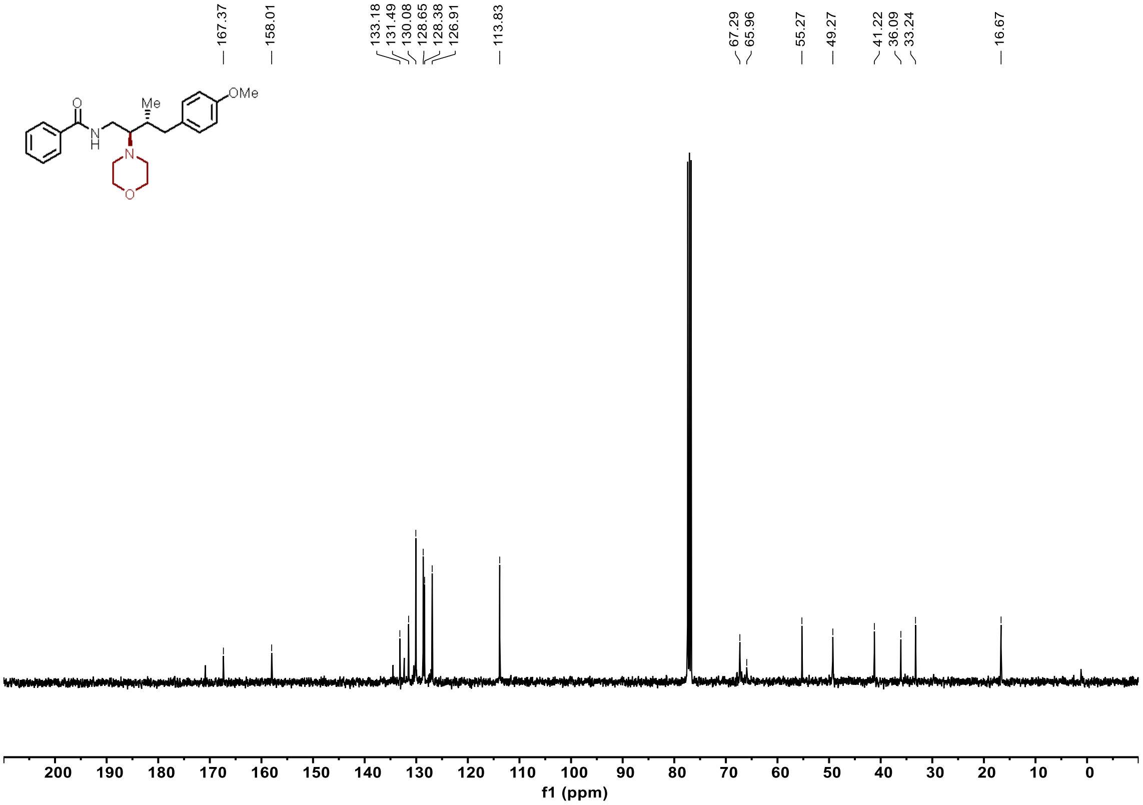

^{13}C NMR (101 MHz, $CDCl_3$) spectra of **4j**

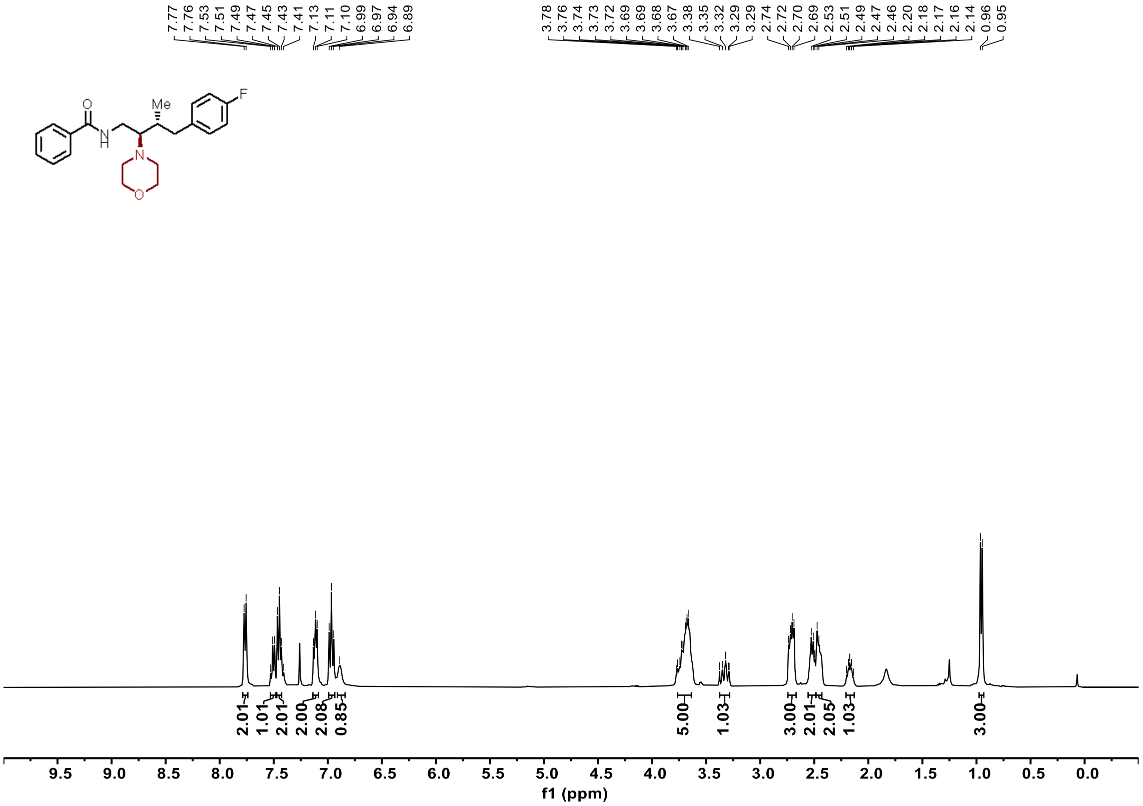

^{1}H NMR (400 MHz, $CDCl_3$) spectra of **4k**

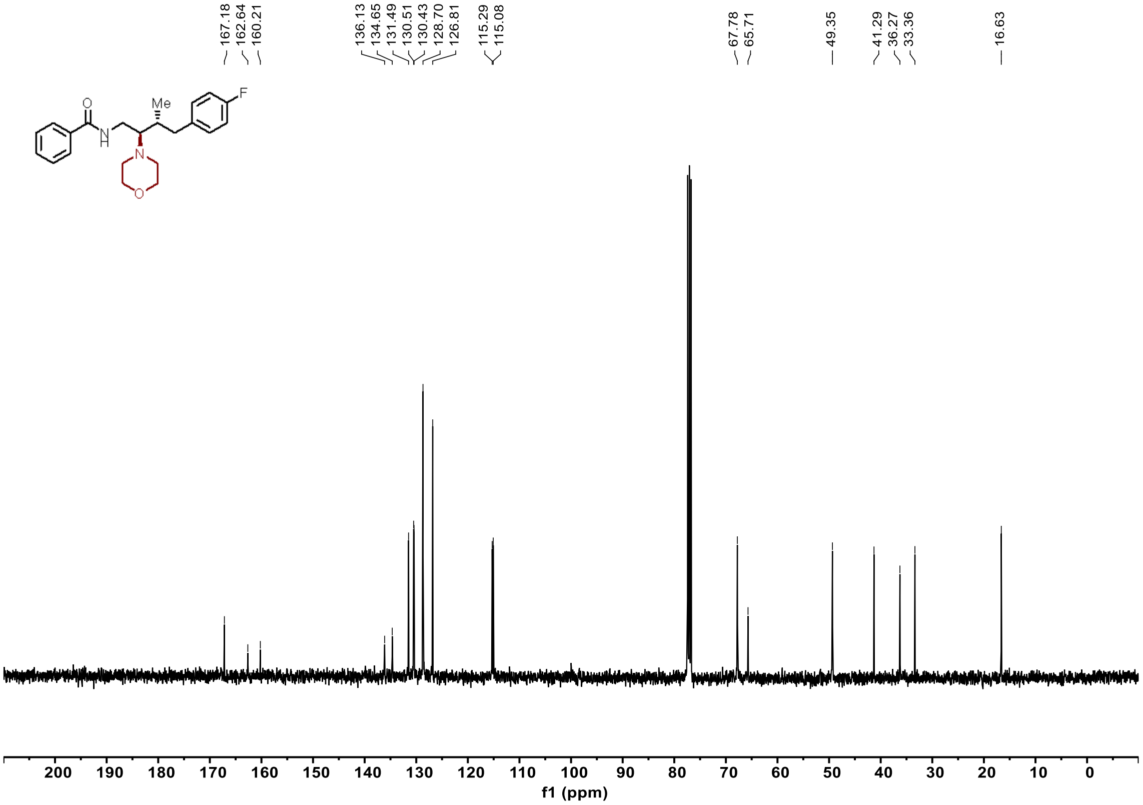

^{13}C NMR (101 MHz, $CDCl_3$) spectra of **4k**

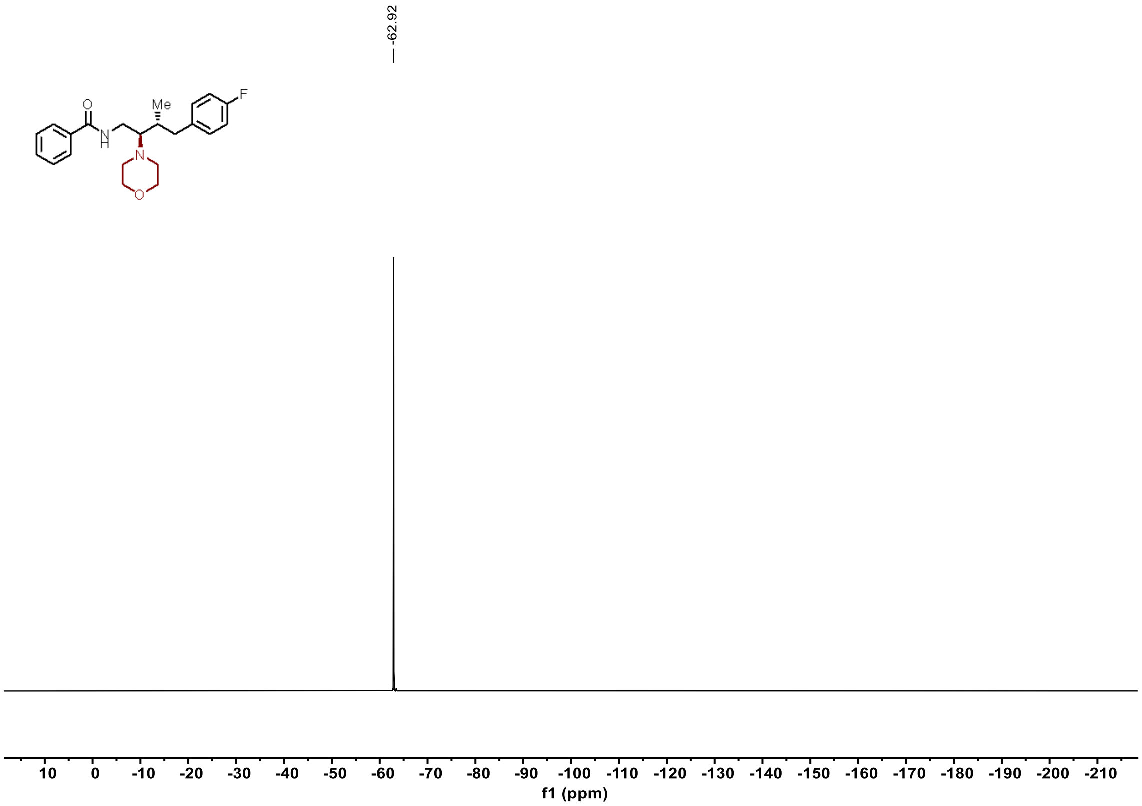

^{19}F NMR (376 MHz, $CDCl_3$) spectra of **4k**

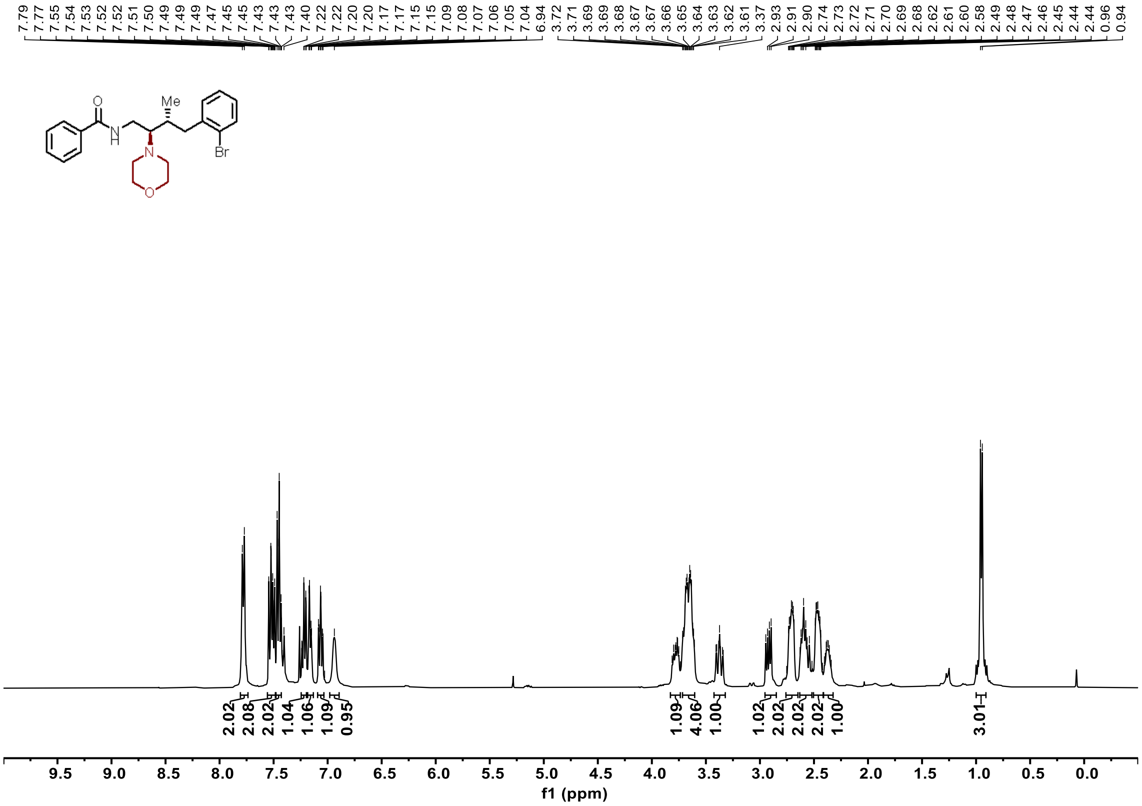

^{1}H NMR (400 MHz, $CDCl_3$) spectra of **4l**

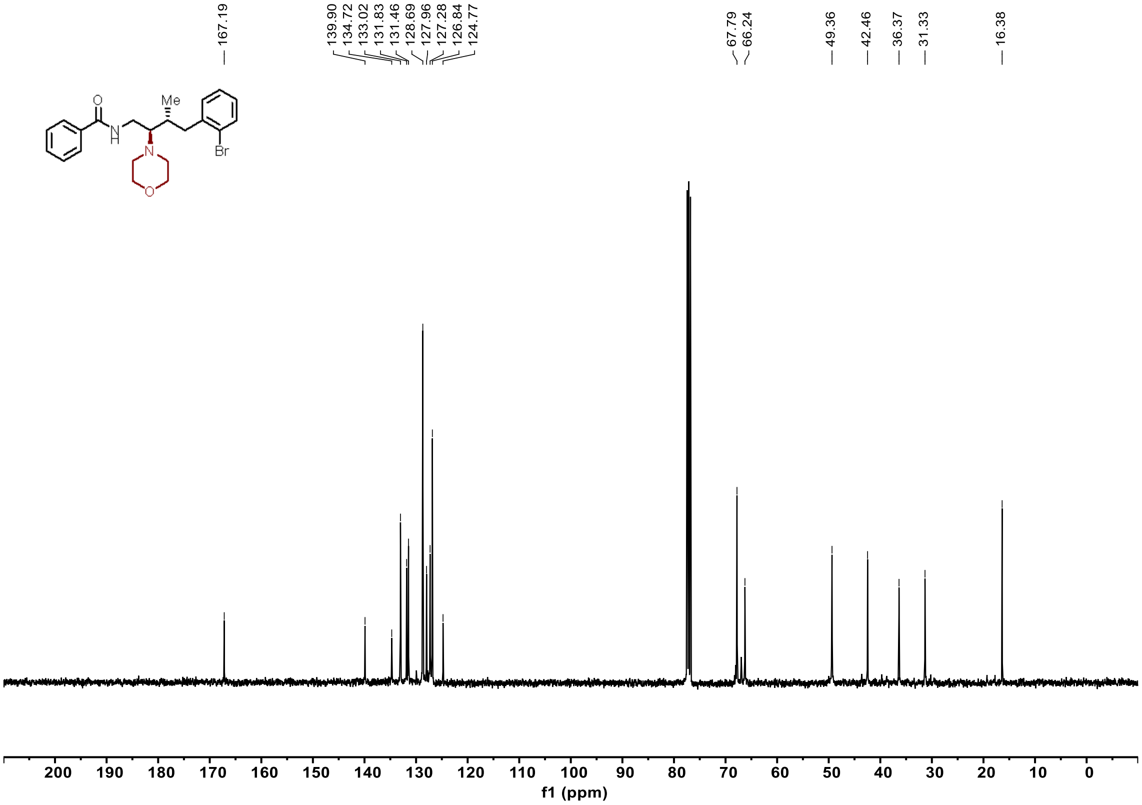

^{13}C NMR (101 MHz, $CDCl_3$) spectra of **4l**

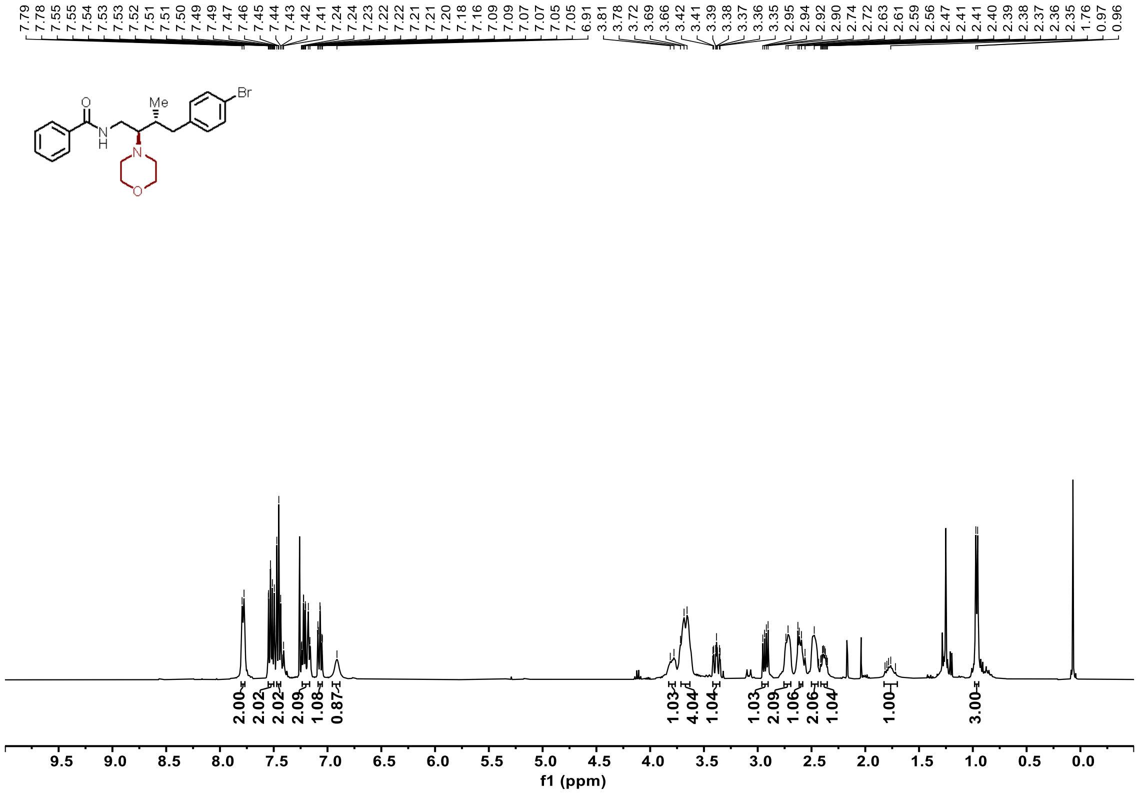

^{1}H NMR (400 MHz, $CDCl_3$) spectra of **4m**

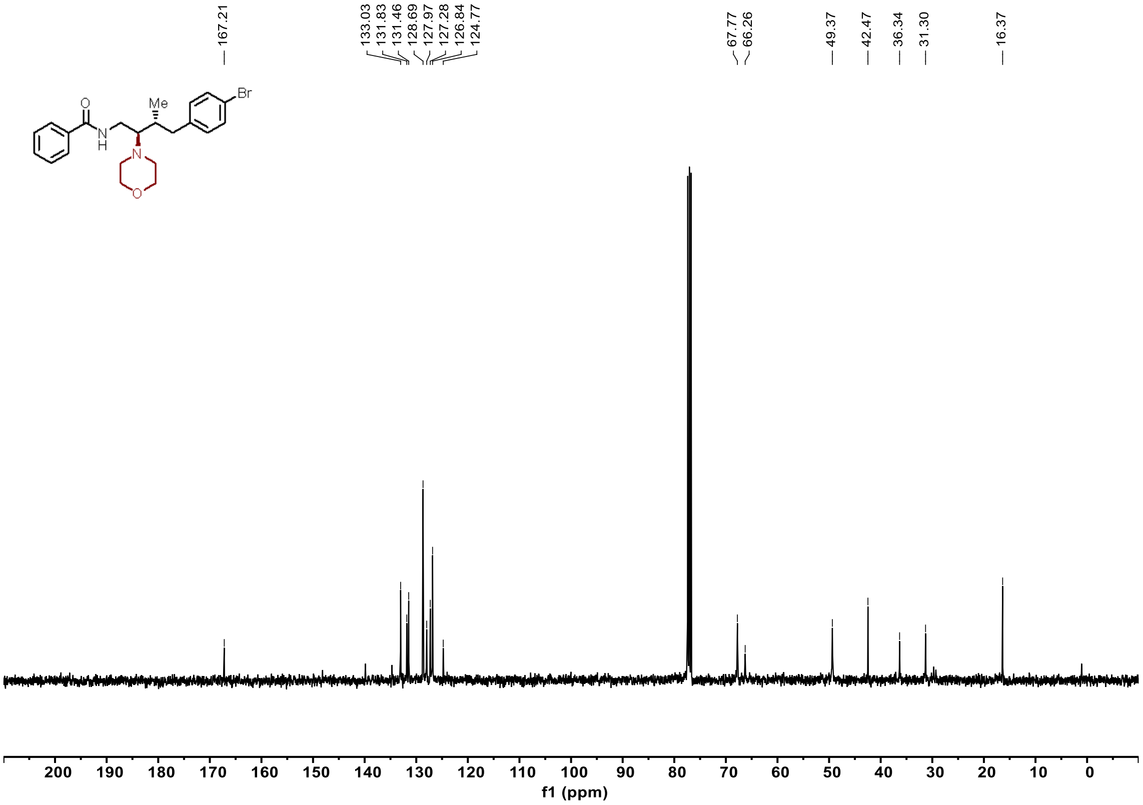

^{13}C NMR (101 MHz, $CDCl_3$) spectra of **4m**

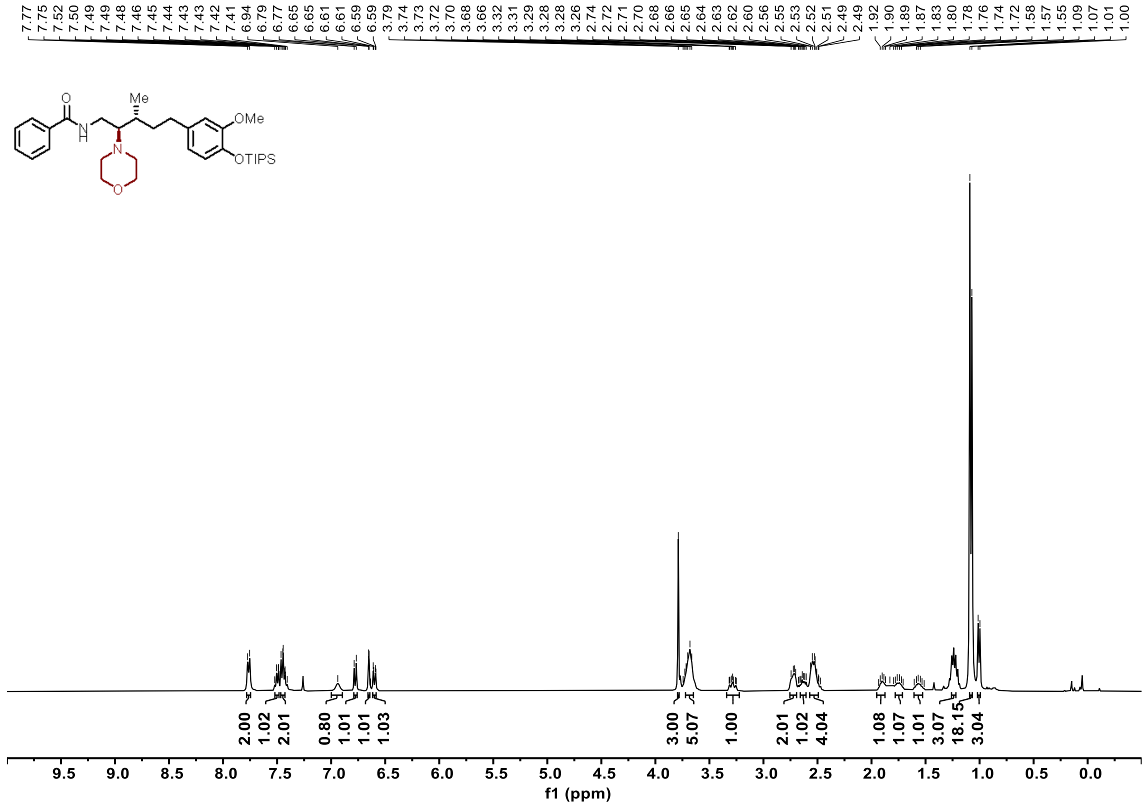

^{1}H NMR (400 MHz, $CDCl_3$) spectra of **4n**

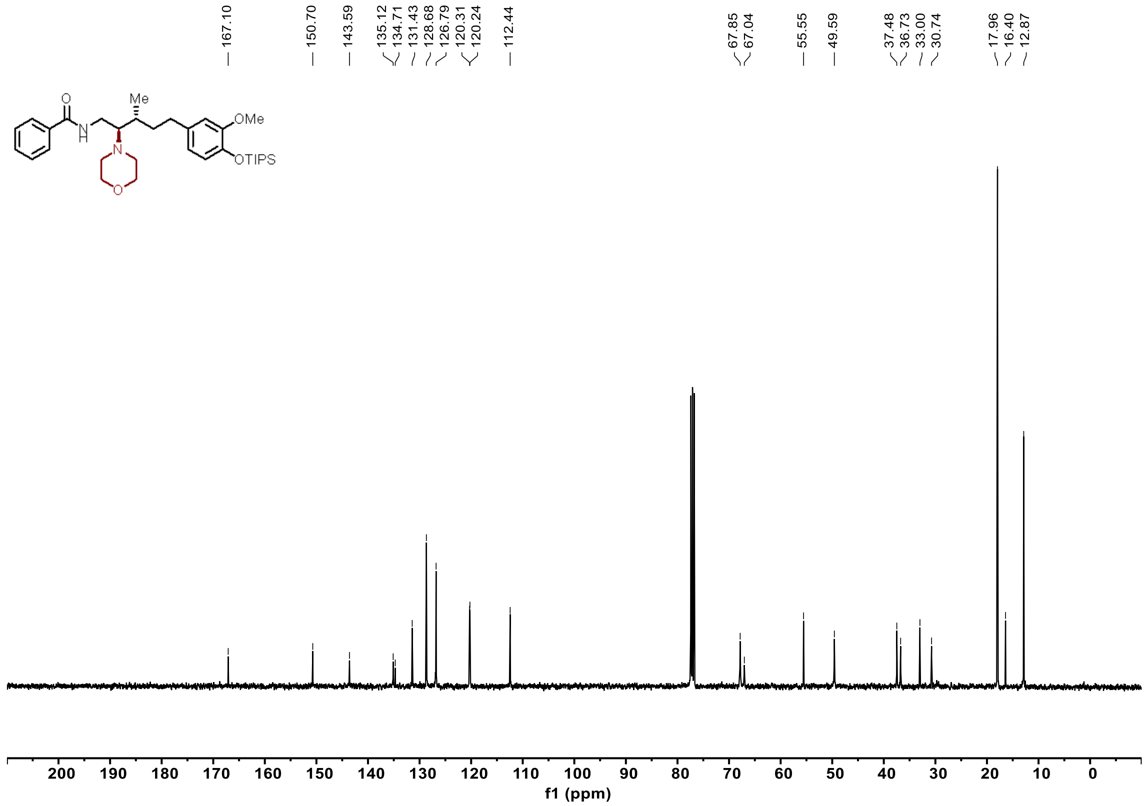

^{13}C NMR (101 MHz, $CDCl_3$) spectra of **4n**

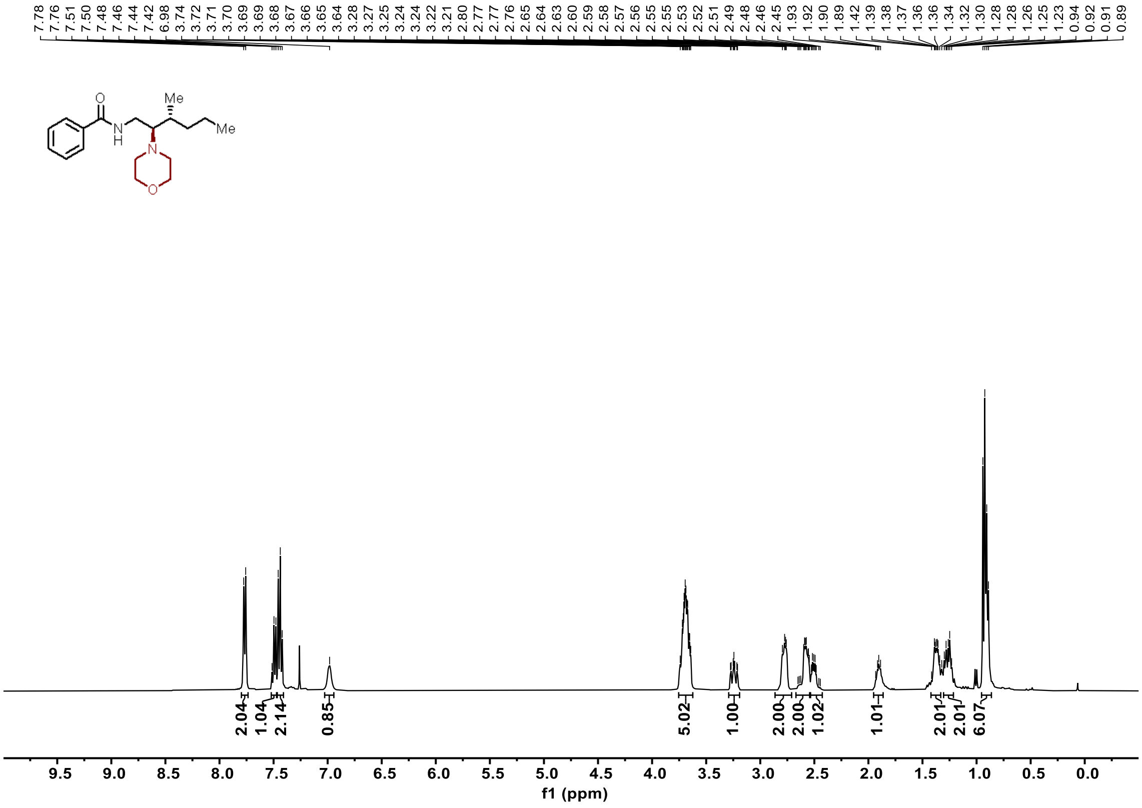

^{1}H NMR (400 MHz, $CDCl_3$) spectra of **4o**

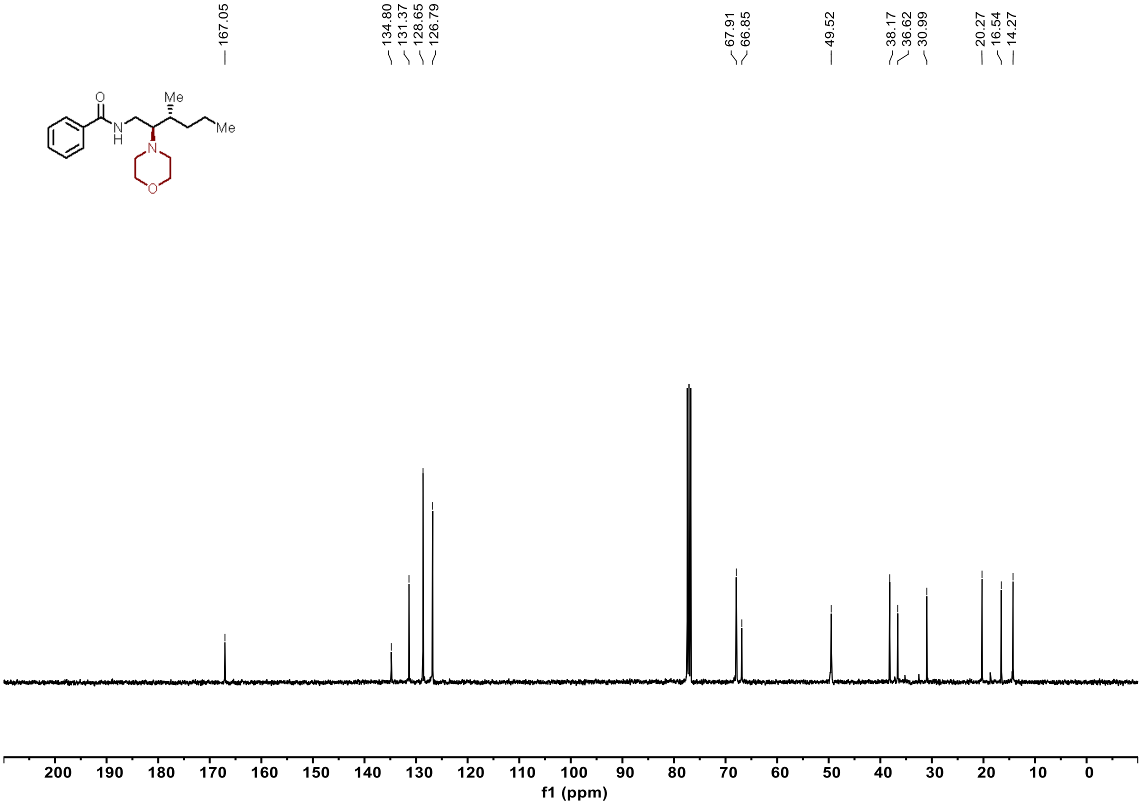

^{13}C NMR (101 MHz, $CDCl_3$) spectra of **4o**

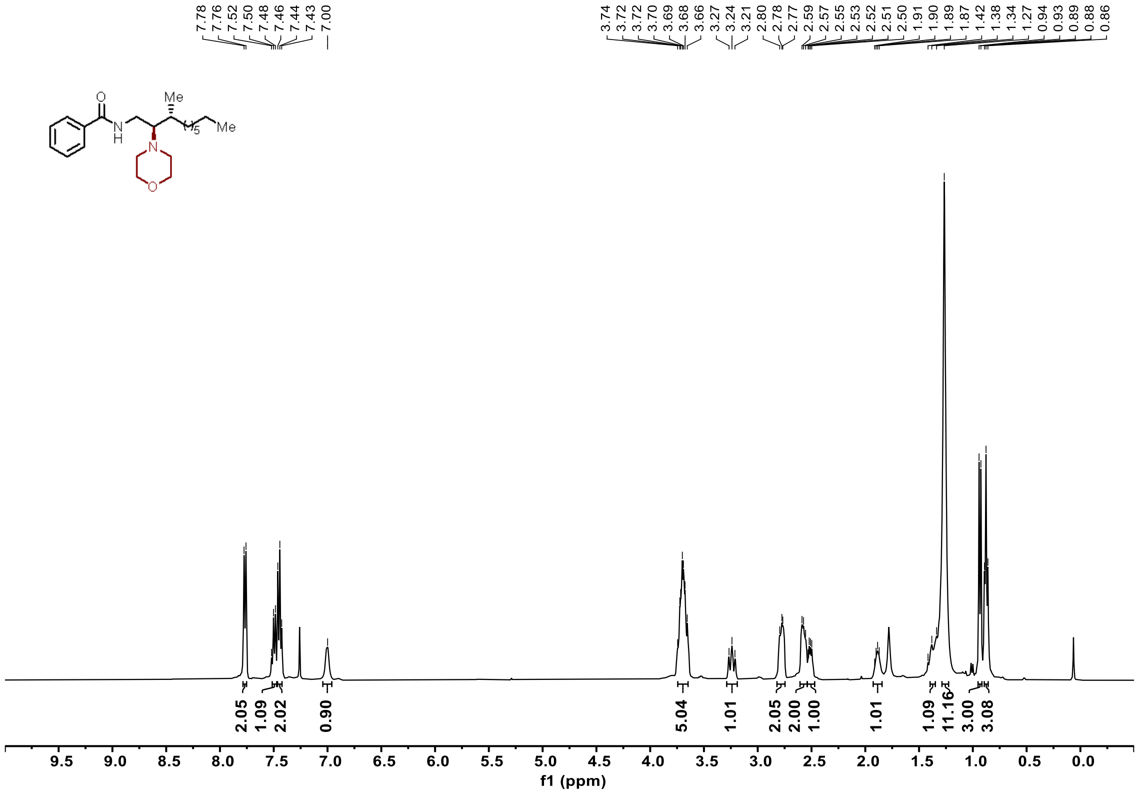

^{1}H NMR (400 MHz, $CDCl_3$) spectra of **4p**

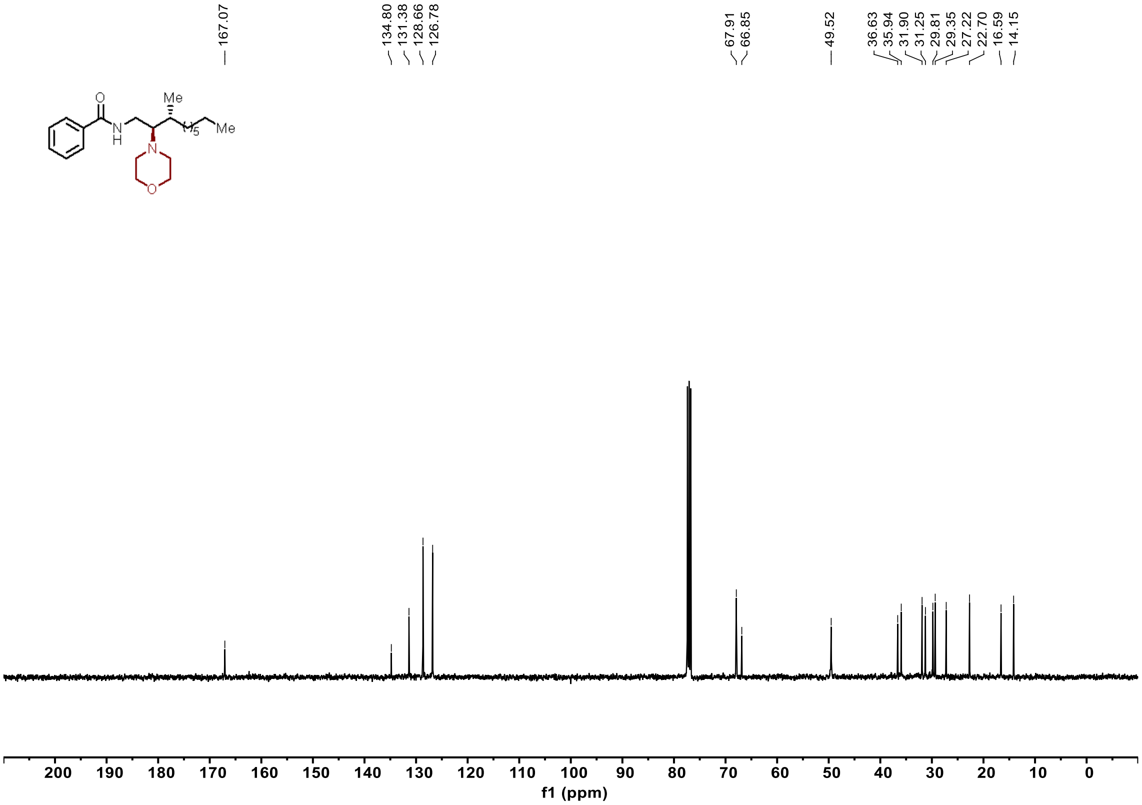

^{13}C NMR (101 MHz, $CDCl_3$) spectra of **4p**

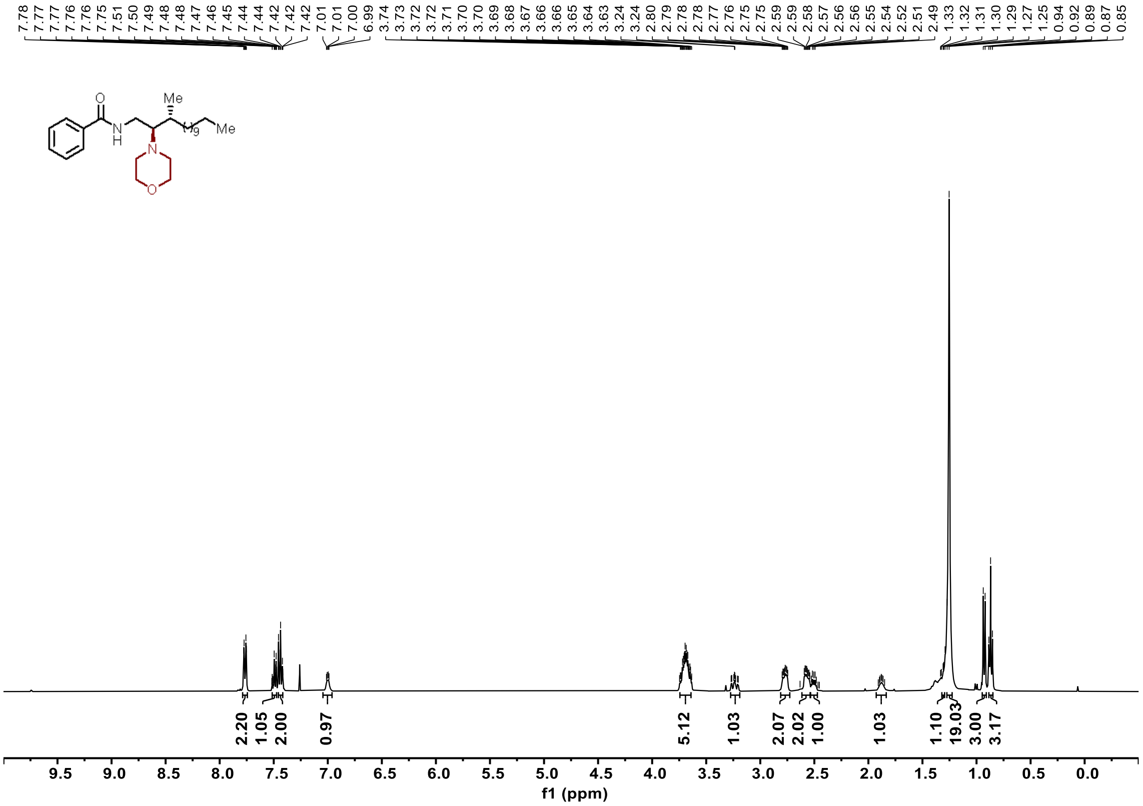

^{1}H NMR (400 MHz, $CDCl_3$) spectra of **4q**

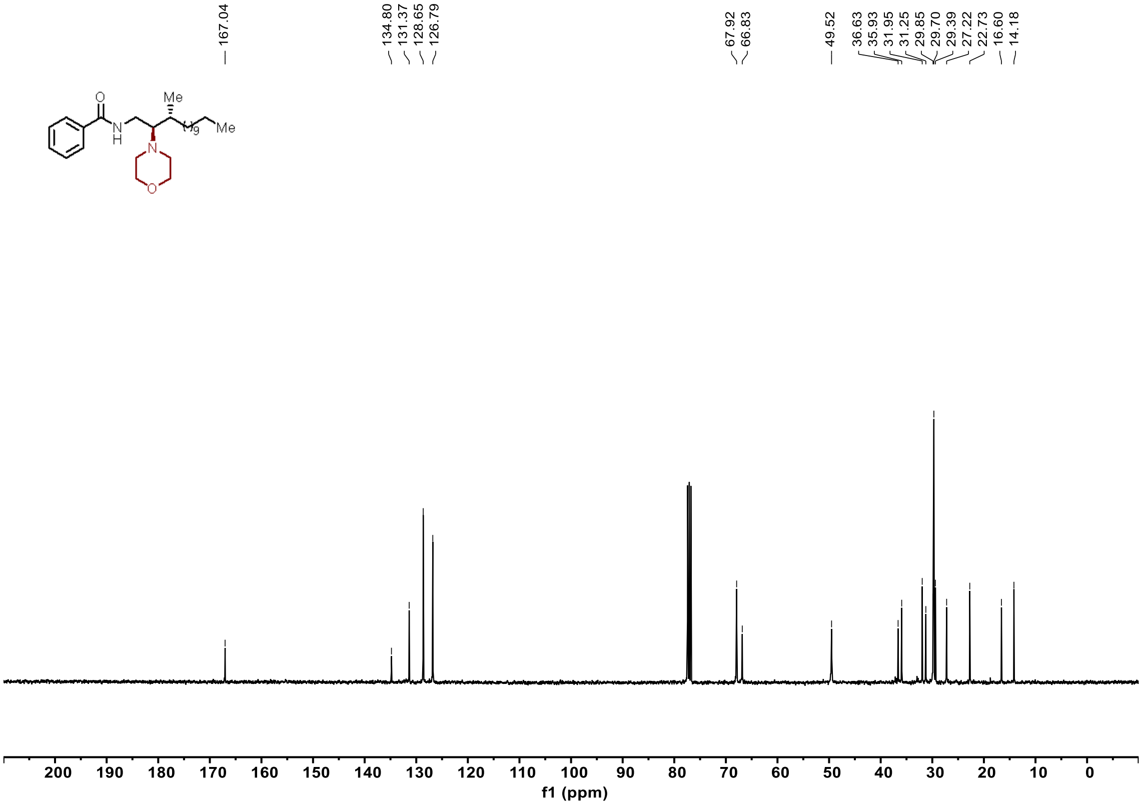

^{13}C NMR (101 MHz, $CDCl_3$) spectra of **4q**

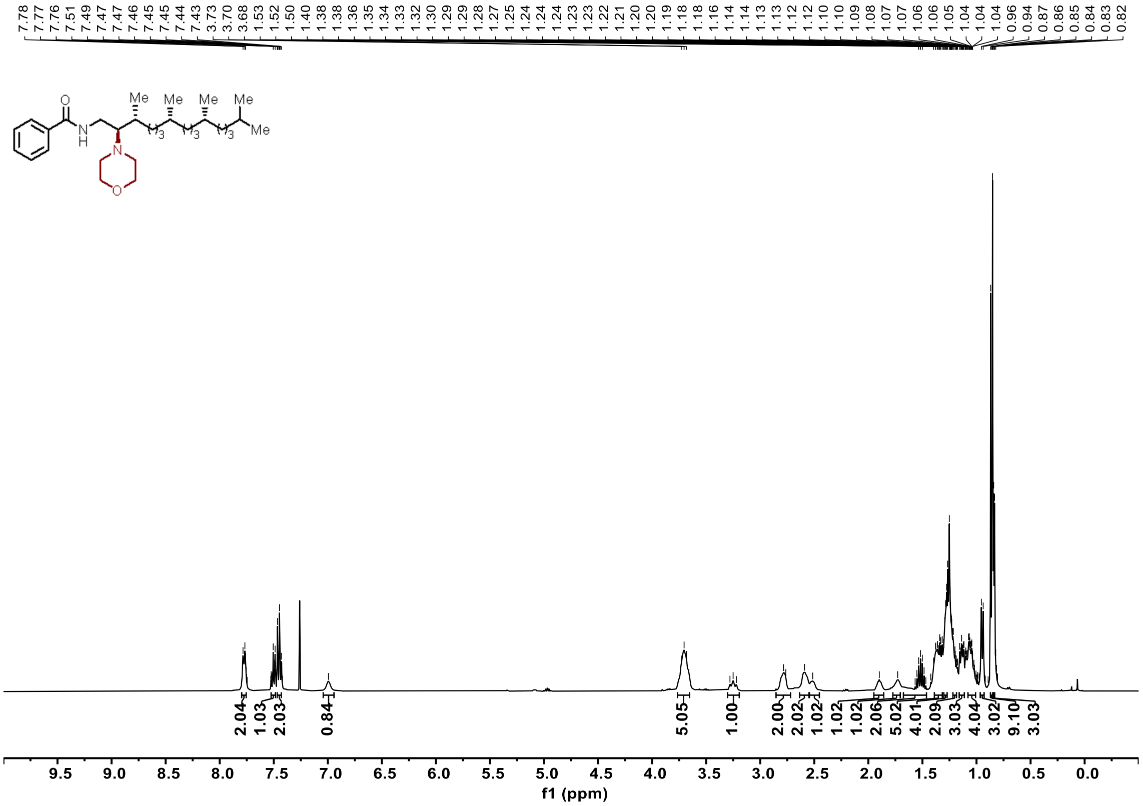

^{1}H NMR (400 MHz, $CDCl_3$) spectra of **4r**

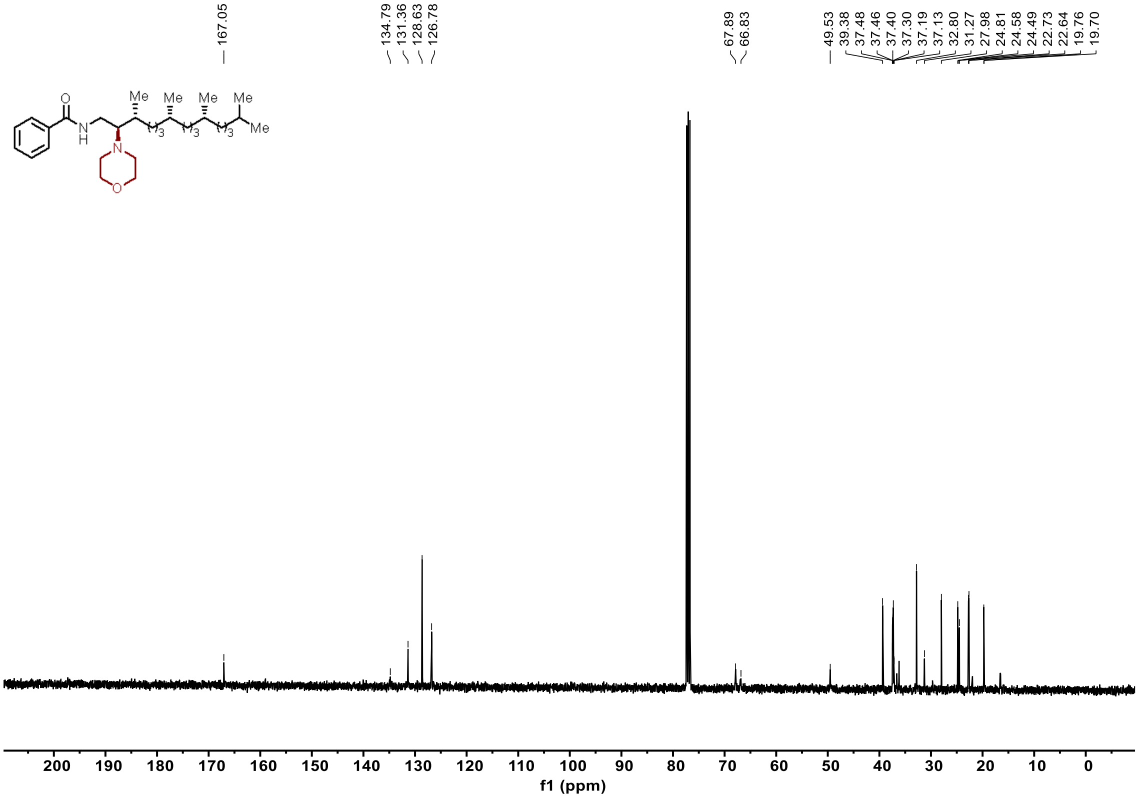

^{13}C NMR (101 MHz, $CDCl_3$) spectra of **4r**

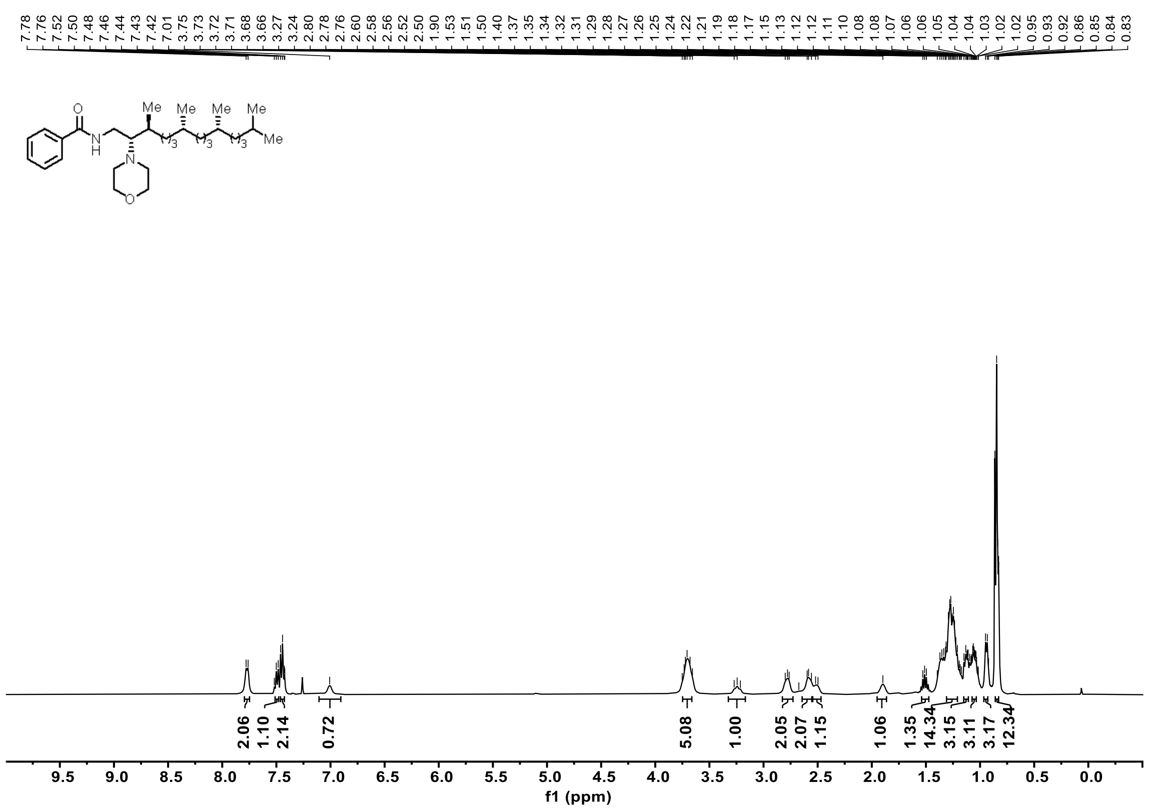

¹H NMR (400 MHz, $CDCl_3$) spectra of **4ak**

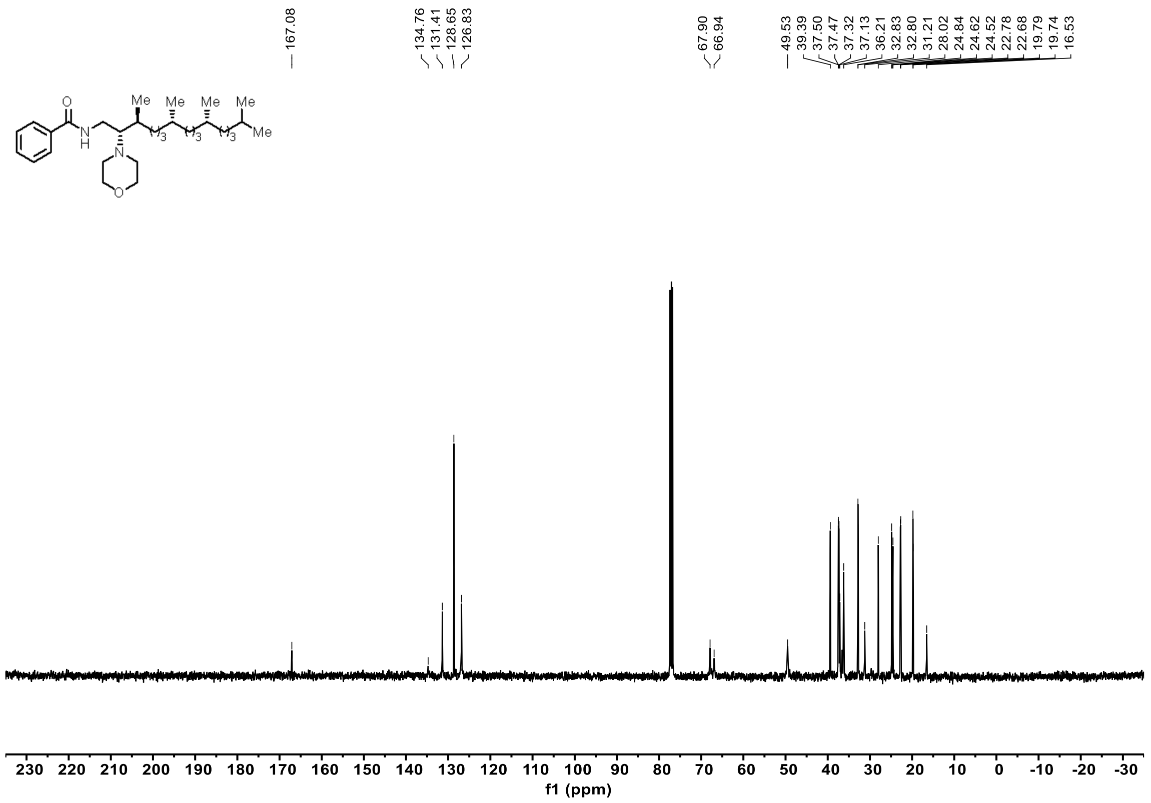

¹³C NMR (101 MHz, $CDCl_3$) spectra of **4ak**

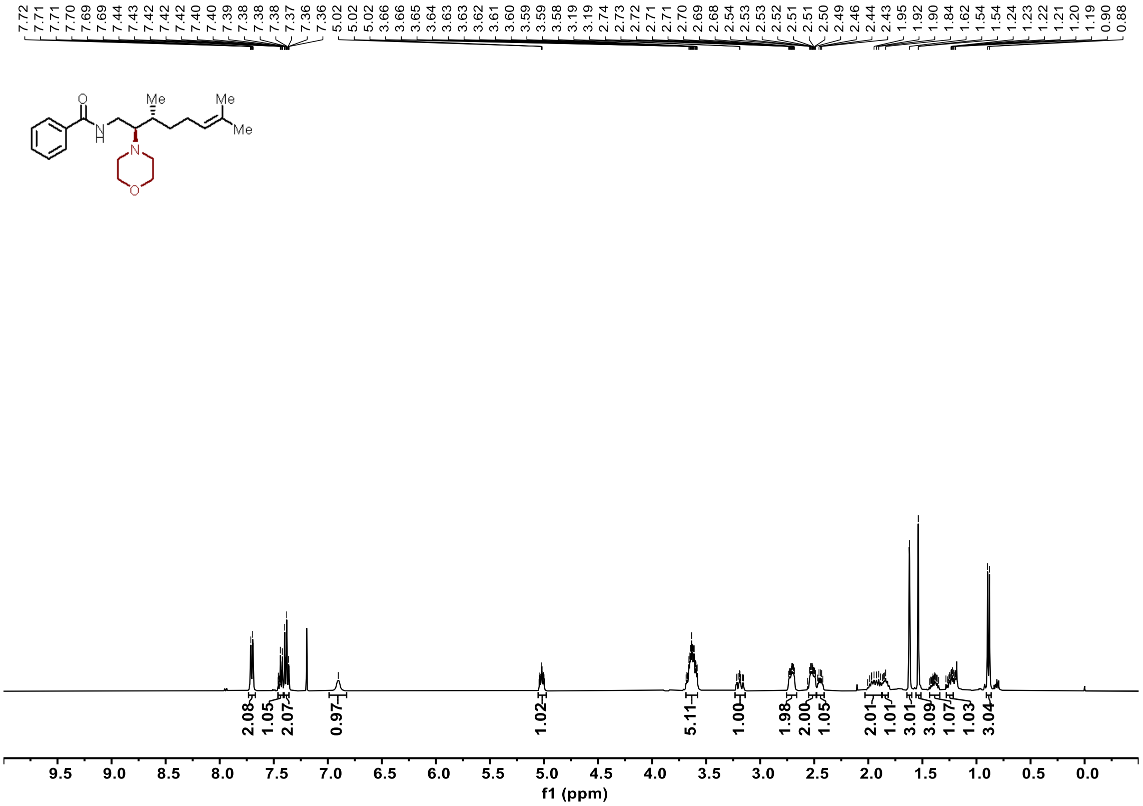

^{1}H NMR (400 MHz, $CDCl_3$) spectra of **4s**

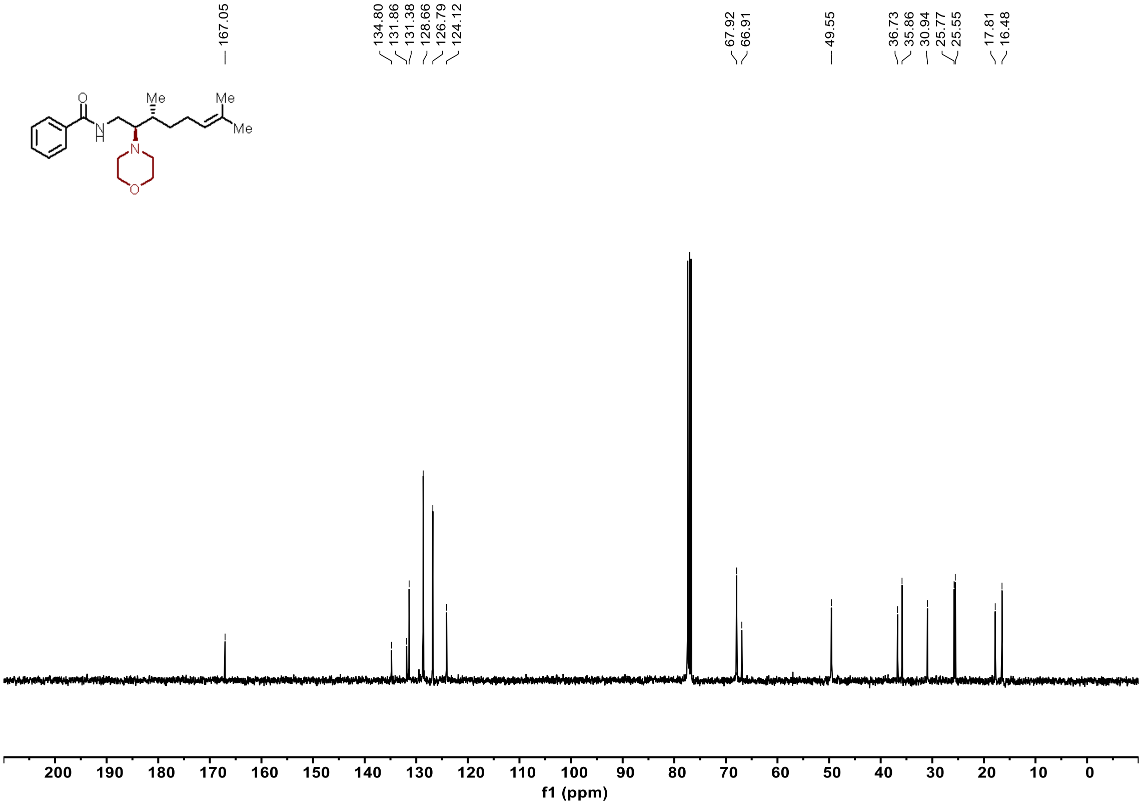

^{13}C NMR (101 MHz, $CDCl_3$) spectra of **4s**

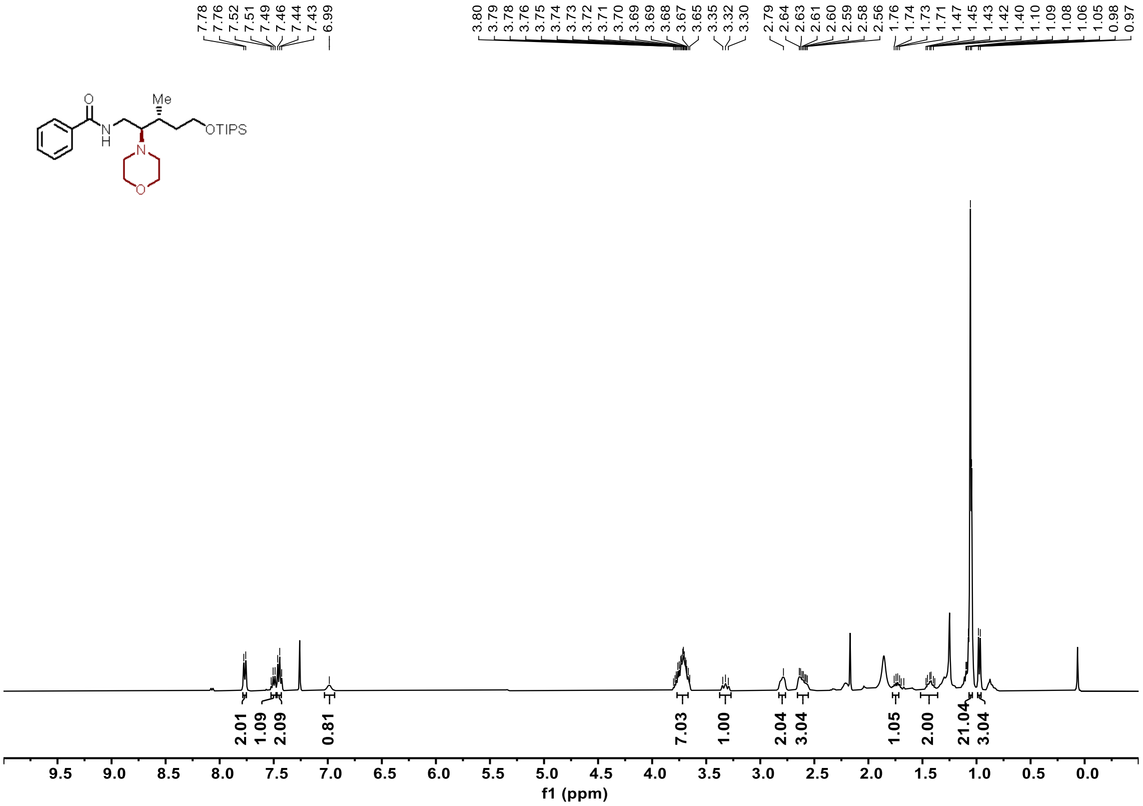

^{1}H NMR (400 MHz, $CDCl_3$) spectra of **4t**

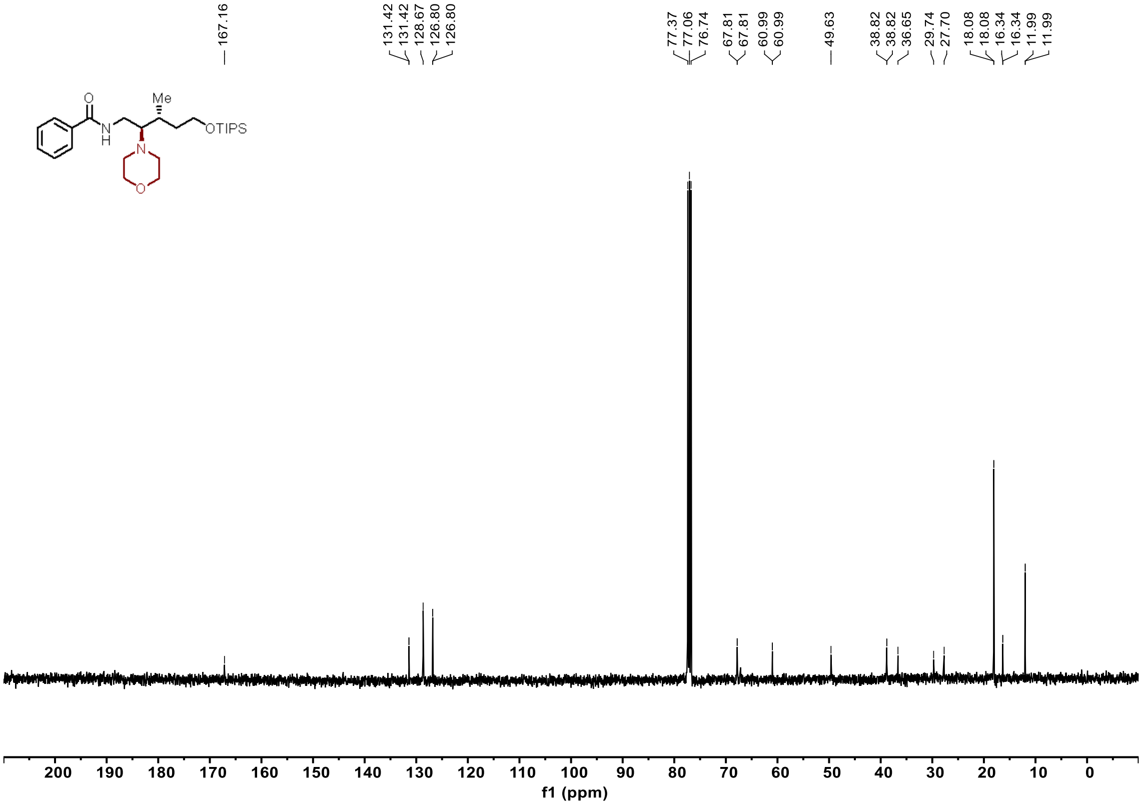

^{13}C NMR (101 MHz, $CDCl_3$) spectra of **4t**

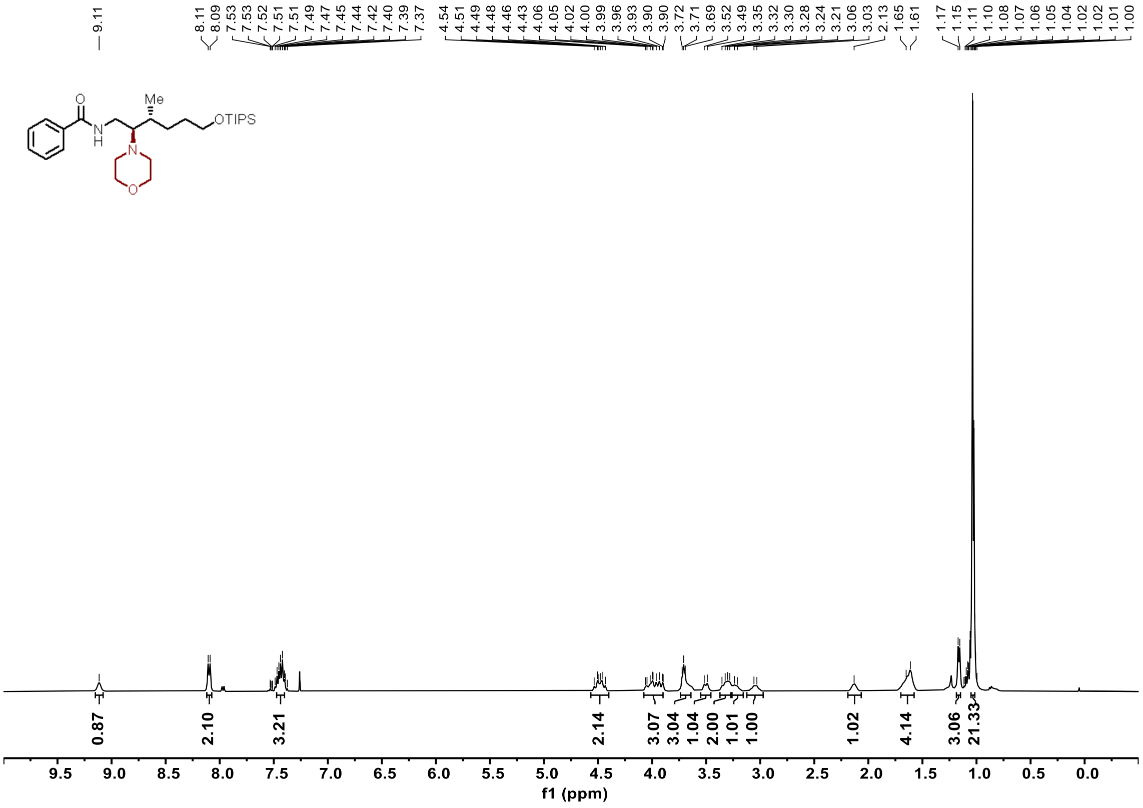

^{1}**H NMR** (400 MHz, $CDCl_3$) spectra of **4u**

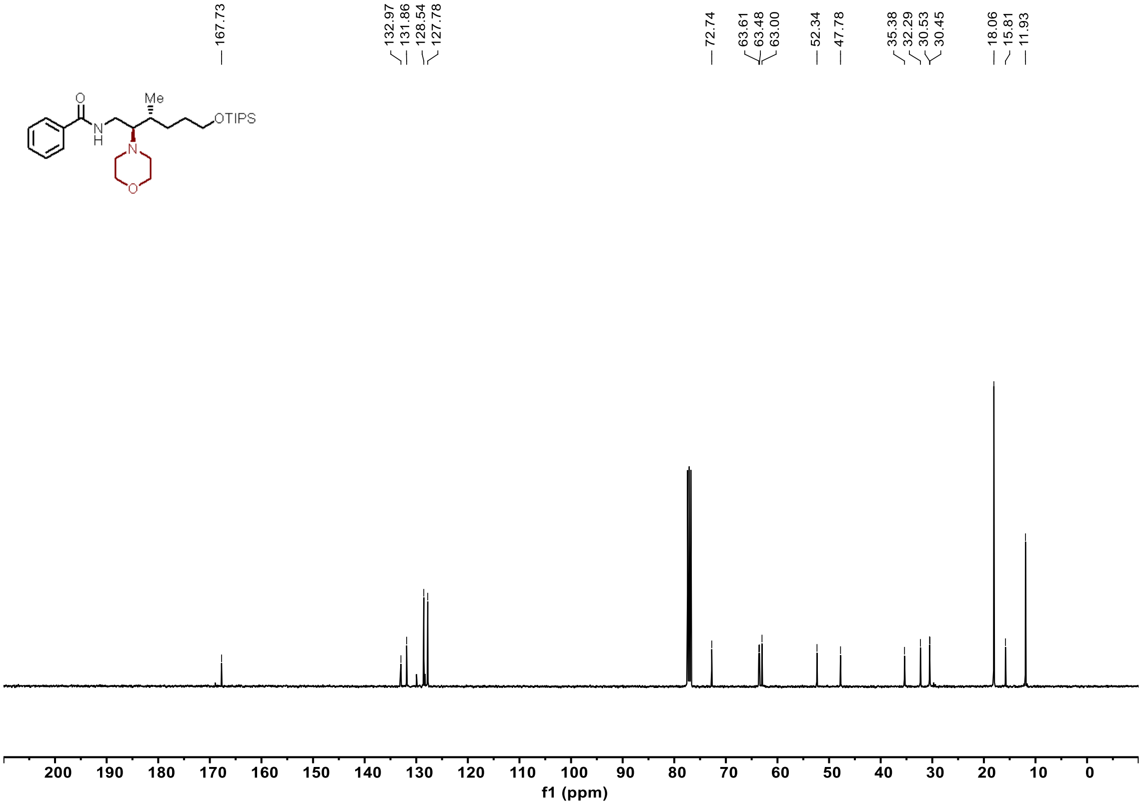

^{13}**C NMR** (101 MHz, $CDCl_3$) spectra of **4u**

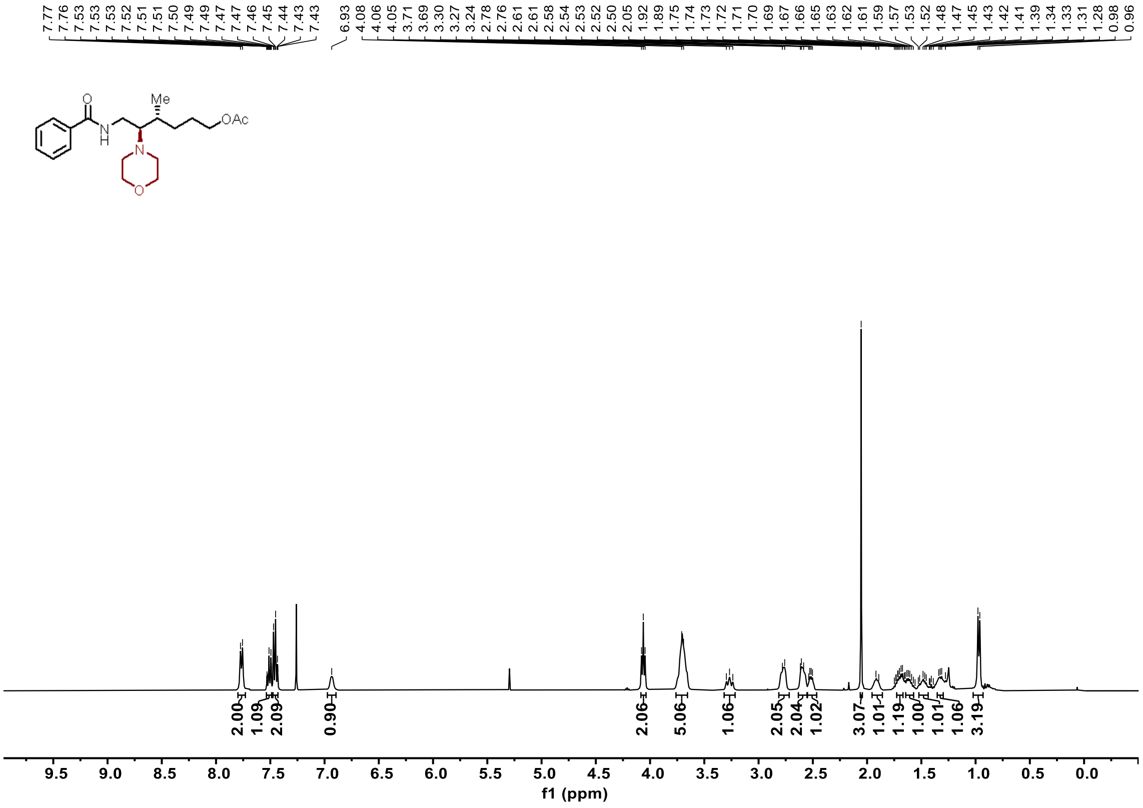

^{1}H NMR (400 MHz, $CDCl_3$) spectra of **4v**

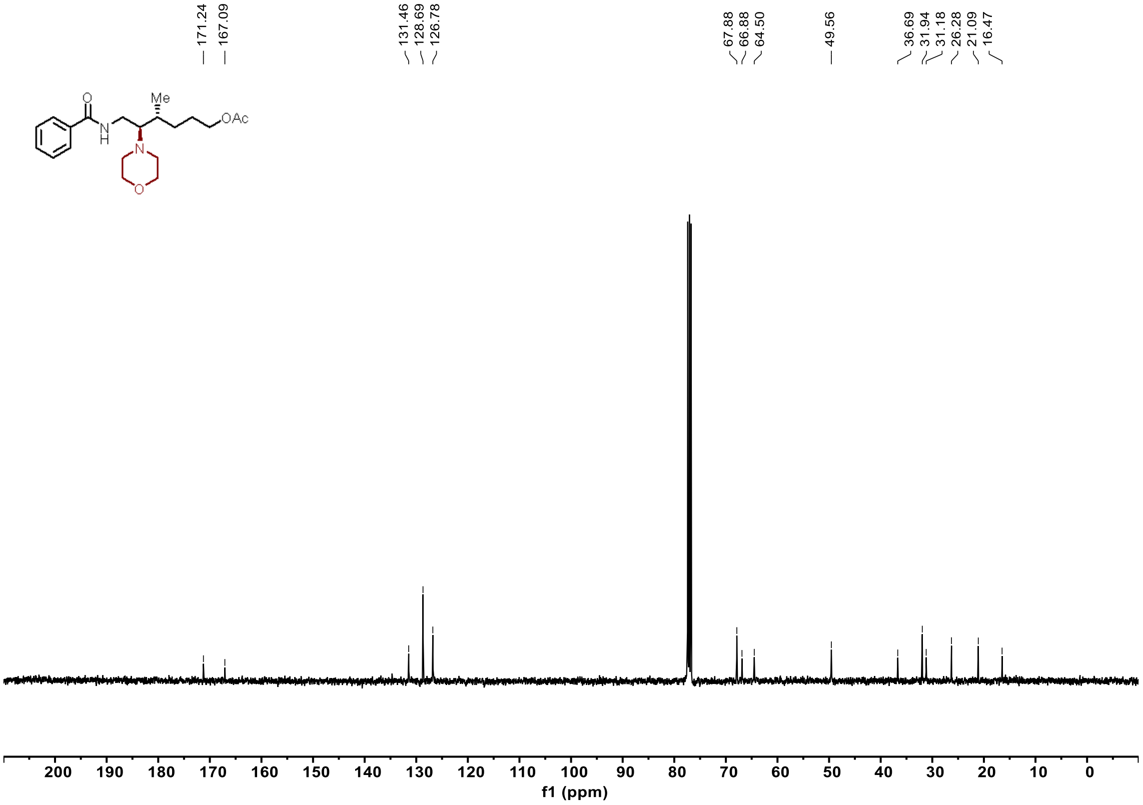

^{13}C NMR (101 MHz, $CDCl_3$) spectra of **4v**

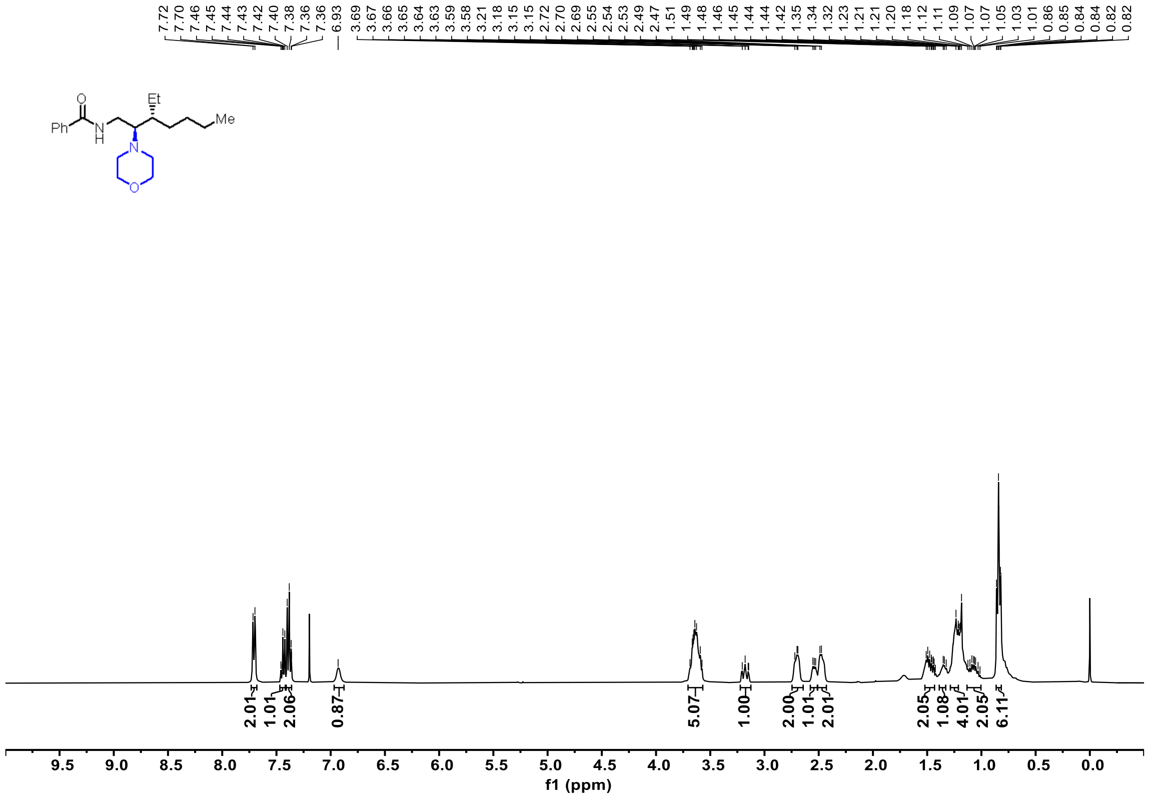

^{1}H NMR (400 MHz, $CDCl_3$) spectra of **4w**

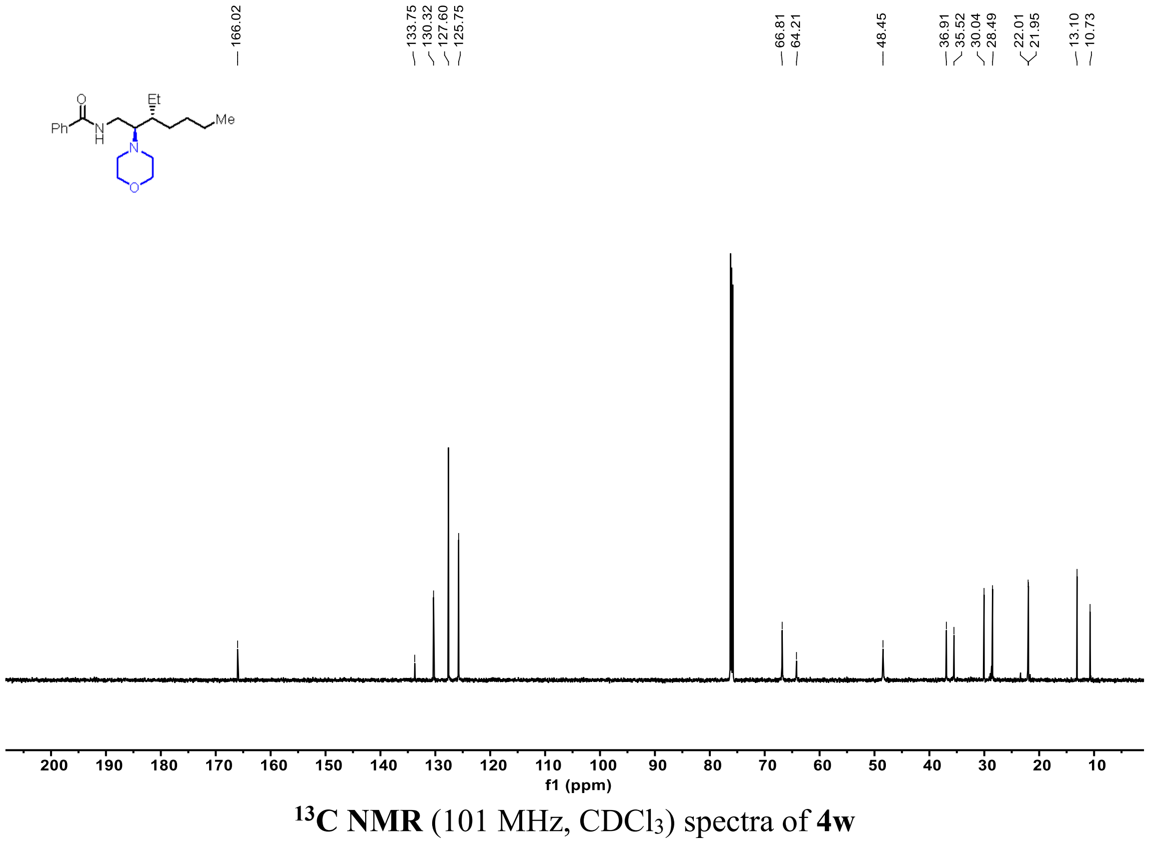

^{13}C NMR (101 MHz, $CDCl_3$) spectra of **4w**

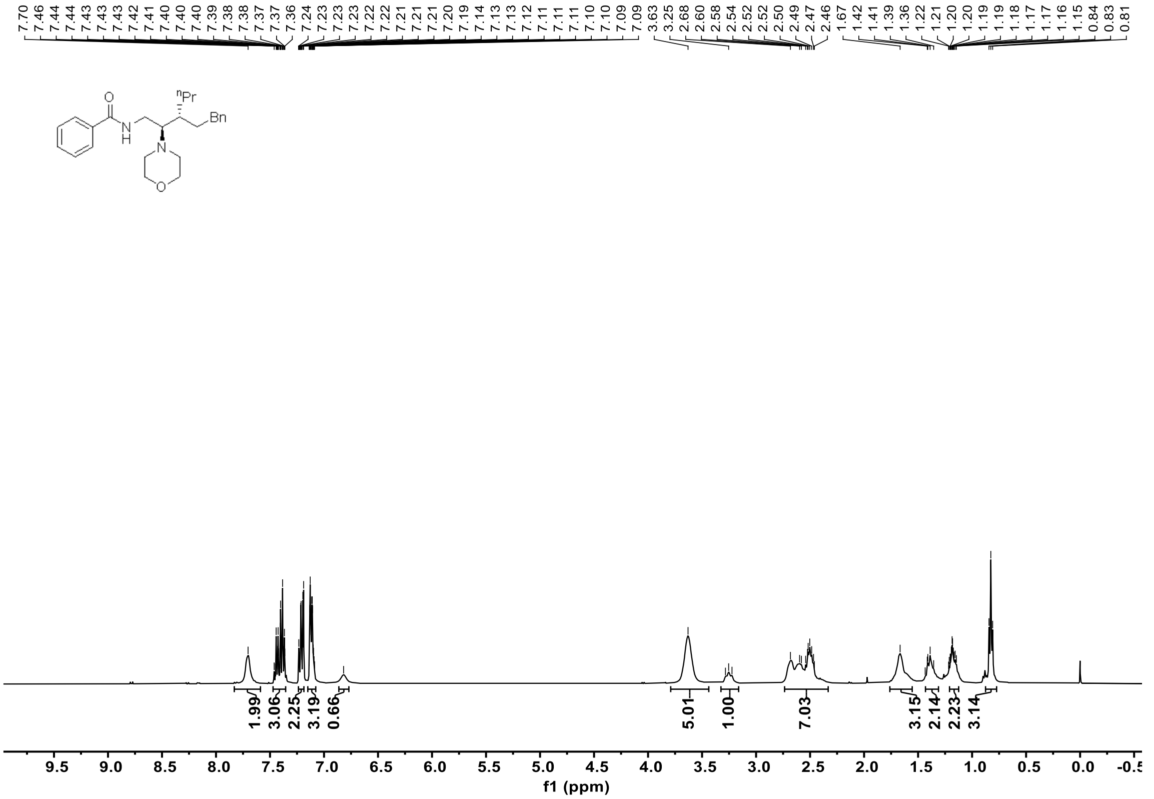

^{1}H NMR (400 MHz, $CDCl_3$) spectra of **4x**

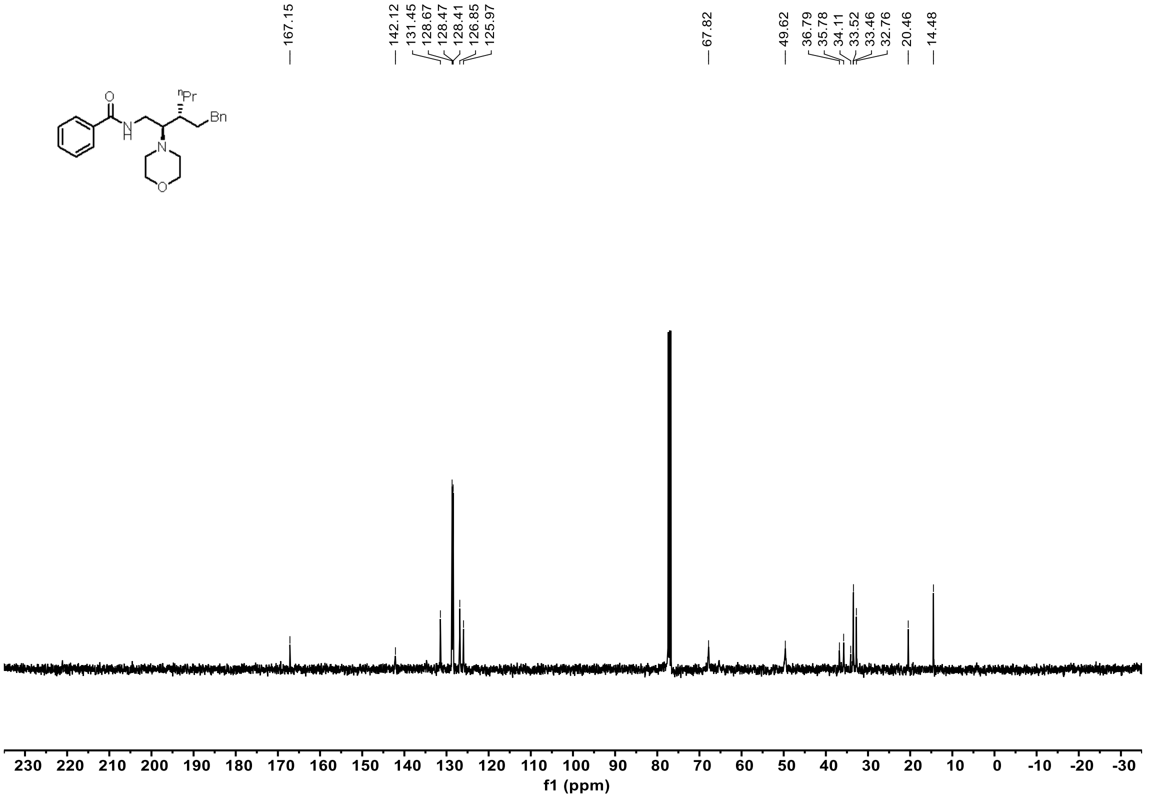

^{13}C NMR (101 MHz, $CDCl_3$) spectra of **4x**

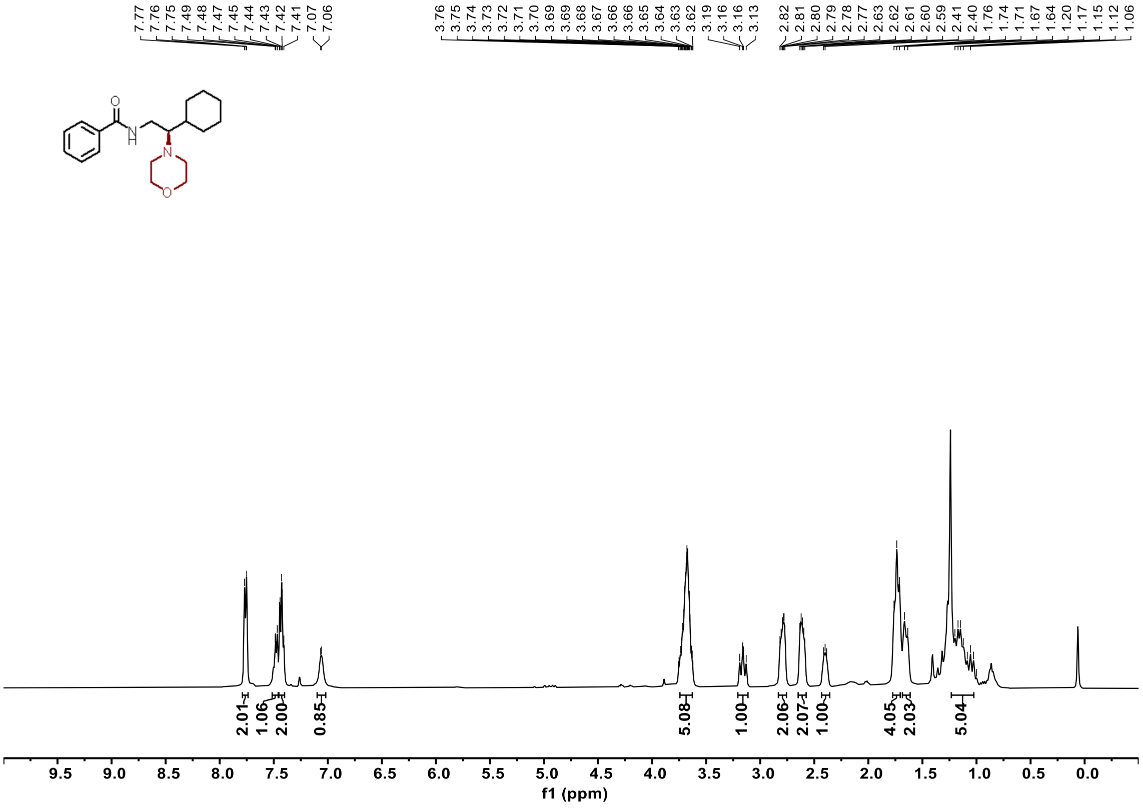

^{1}H NMR (400 MHz, $CDCl_3$) spectra of **4y**

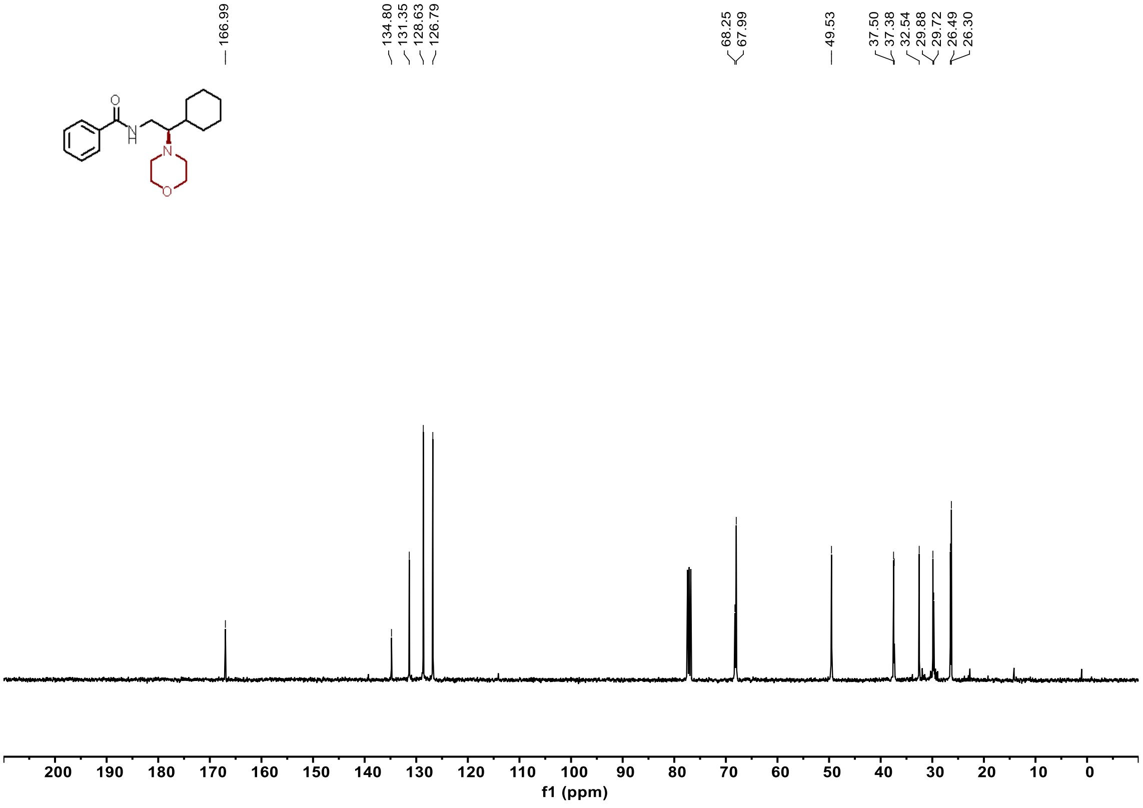

^{13}C NMR (101 MHz, $CDCl_3$) spectra of **4y**

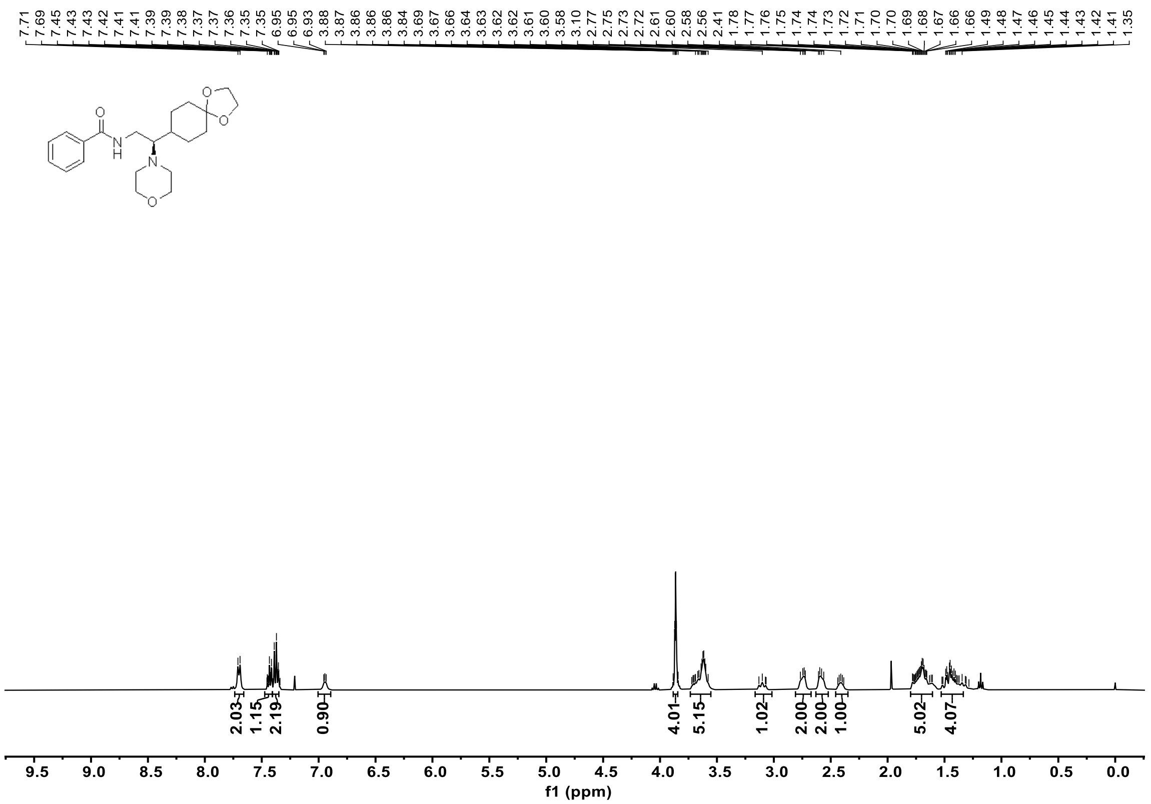

^{1}H NMR (400 MHz, $CDCl_3$) spectra of **4z**

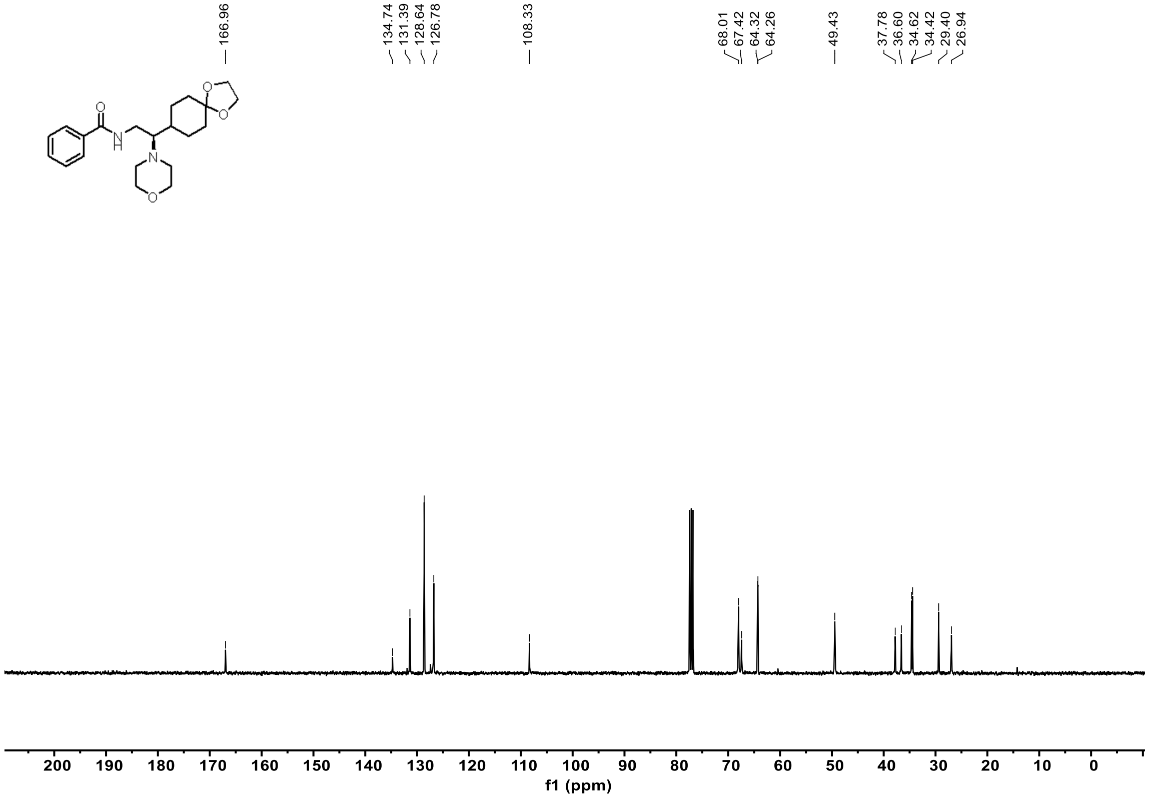

^{13}C NMR (101 MHz, $CDCl_3$) spectra of **4z**

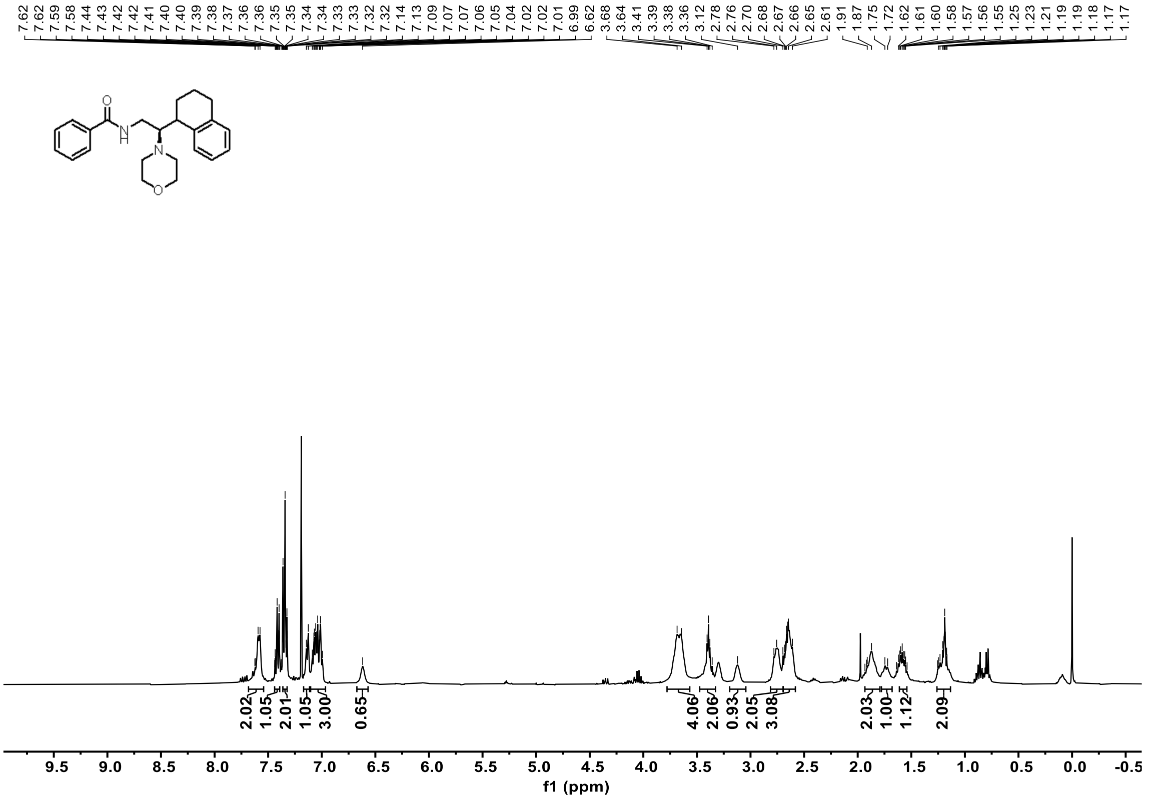

^{1}H NMR (400 MHz, $CDCl_3$) spectra of **4aa**

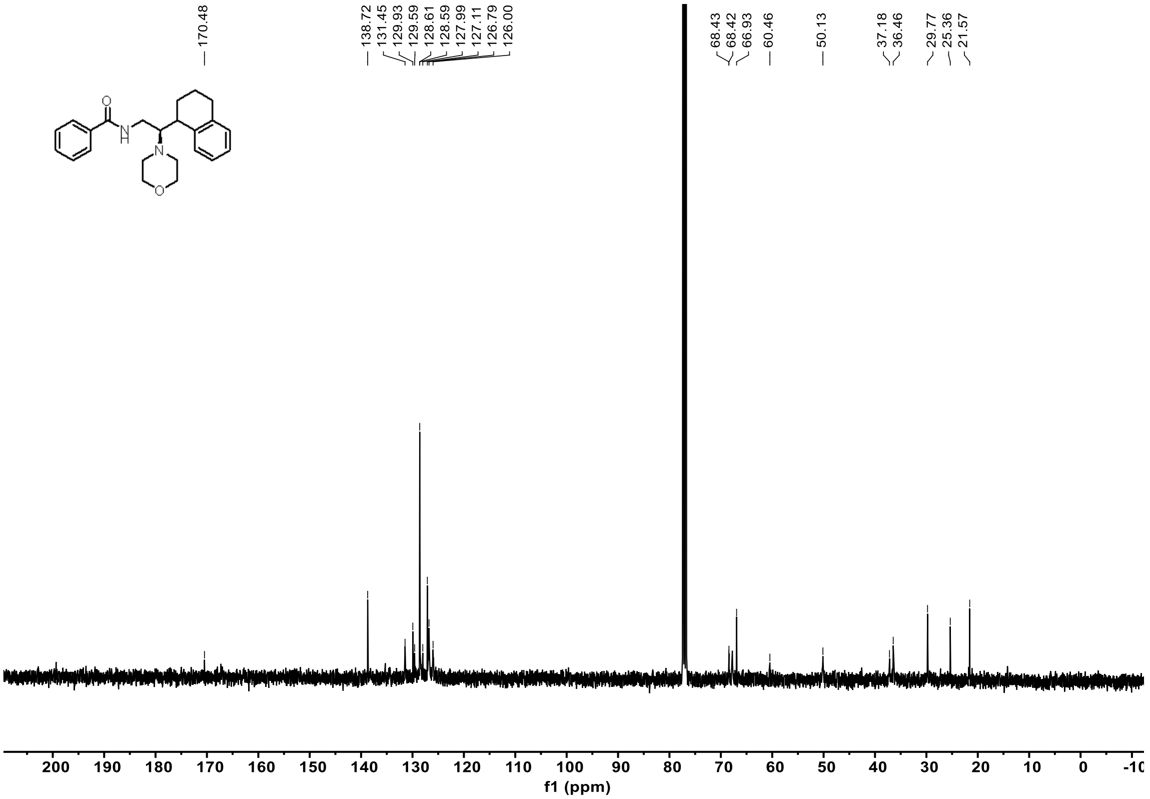

^{13}C NMR (101 MHz, $CDCl_3$) spectra of **4aa**

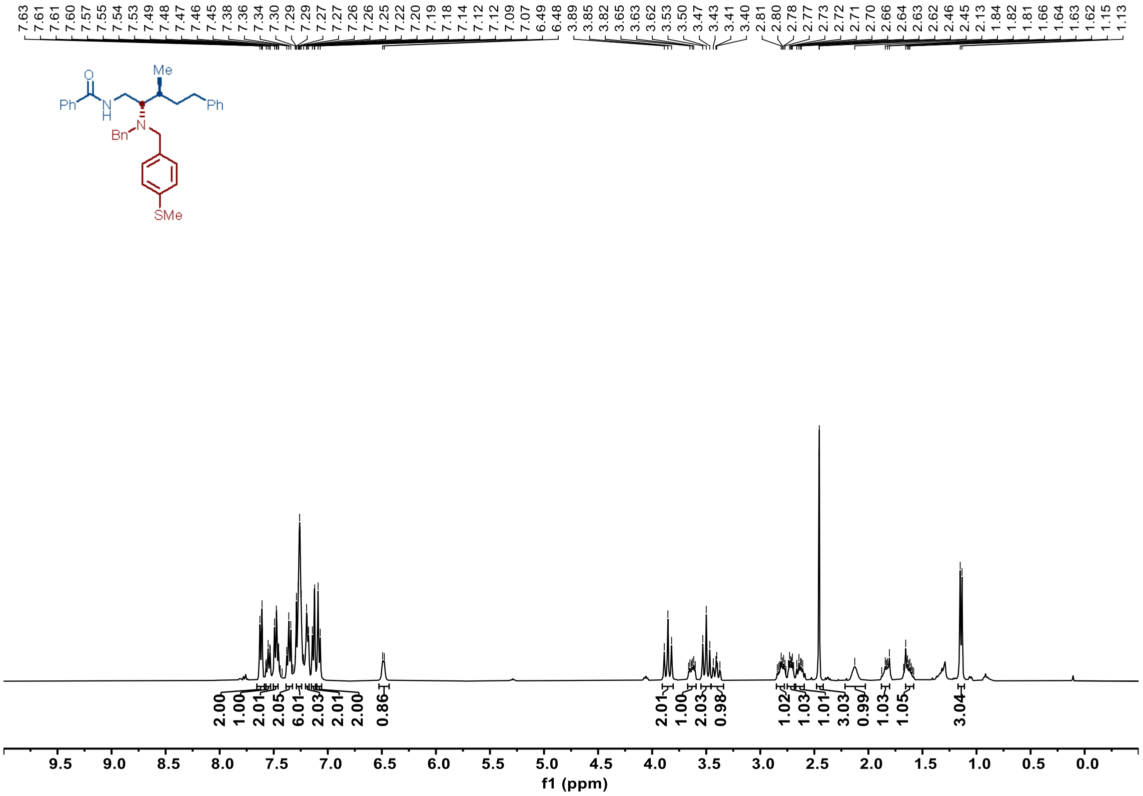

^{1}H NMR (400 MHz, $CDCl_3$) spectra of **(S,S)-3v**

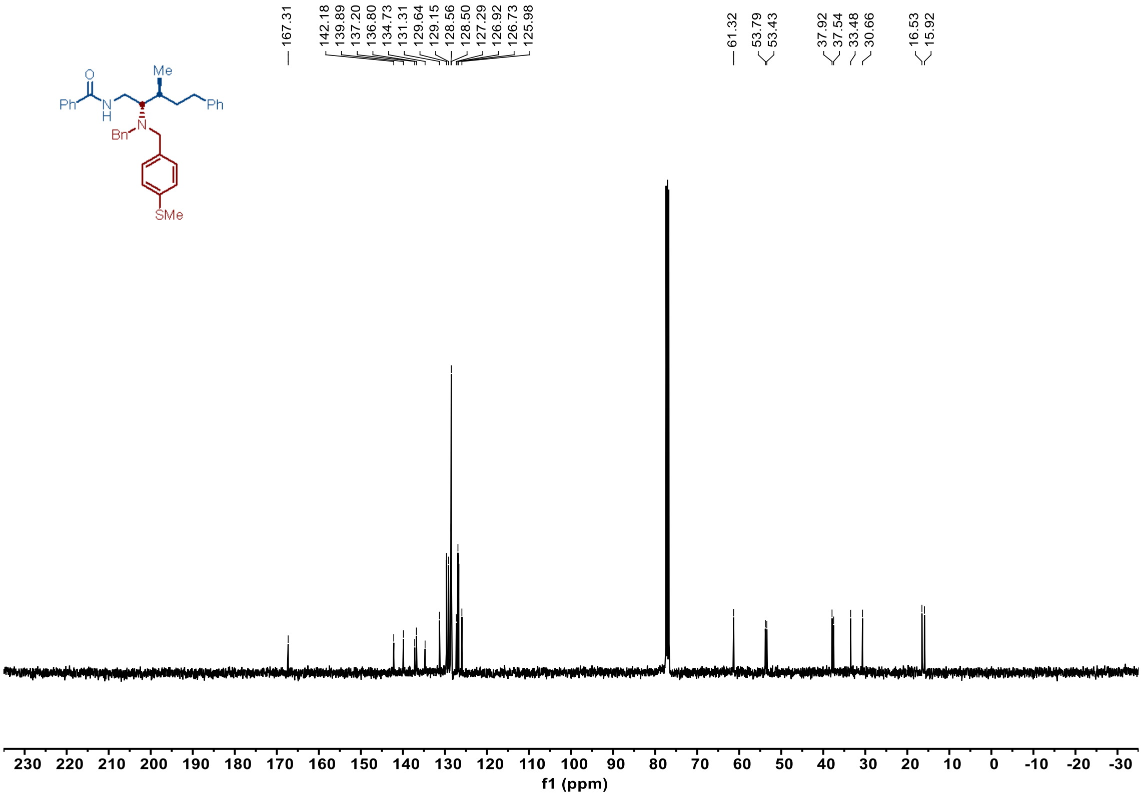

^{13}C NMR (101 MHz, $CDCl_3$) spectra of **(S,S)-3v**

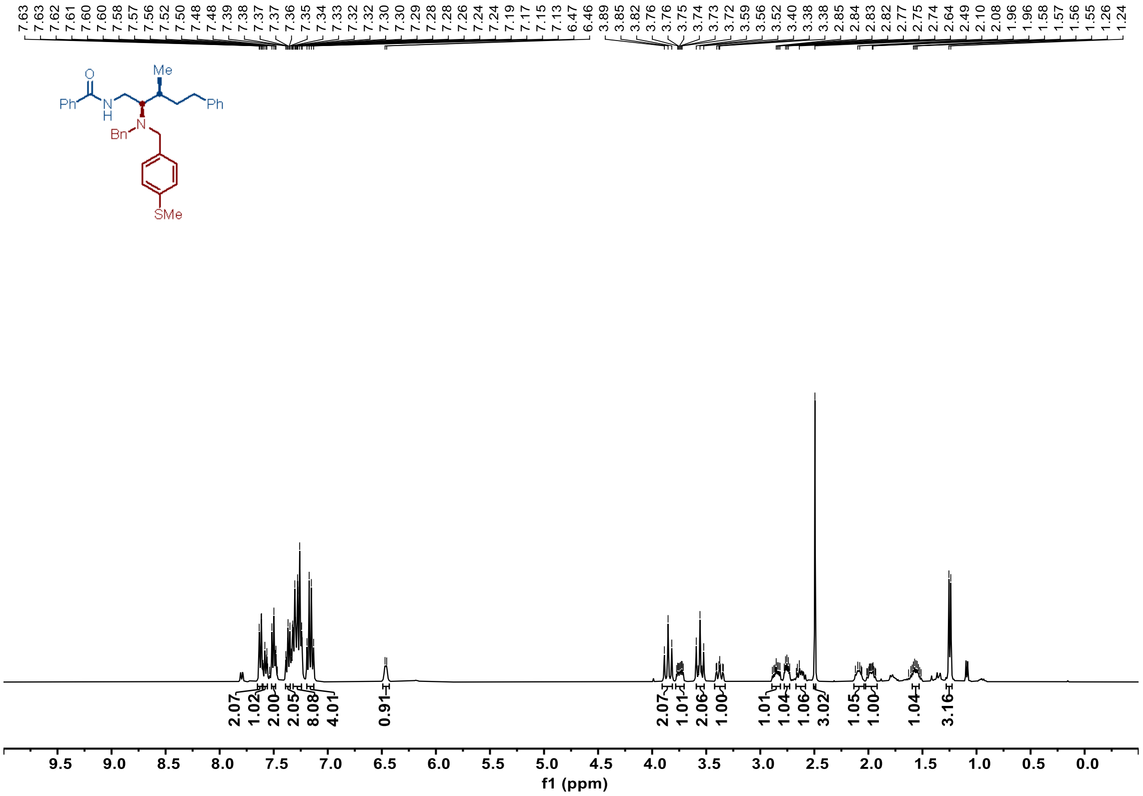

^{1}H NMR (400 MHz, $CDCl_3$) spectra of **(R,S)-3v**

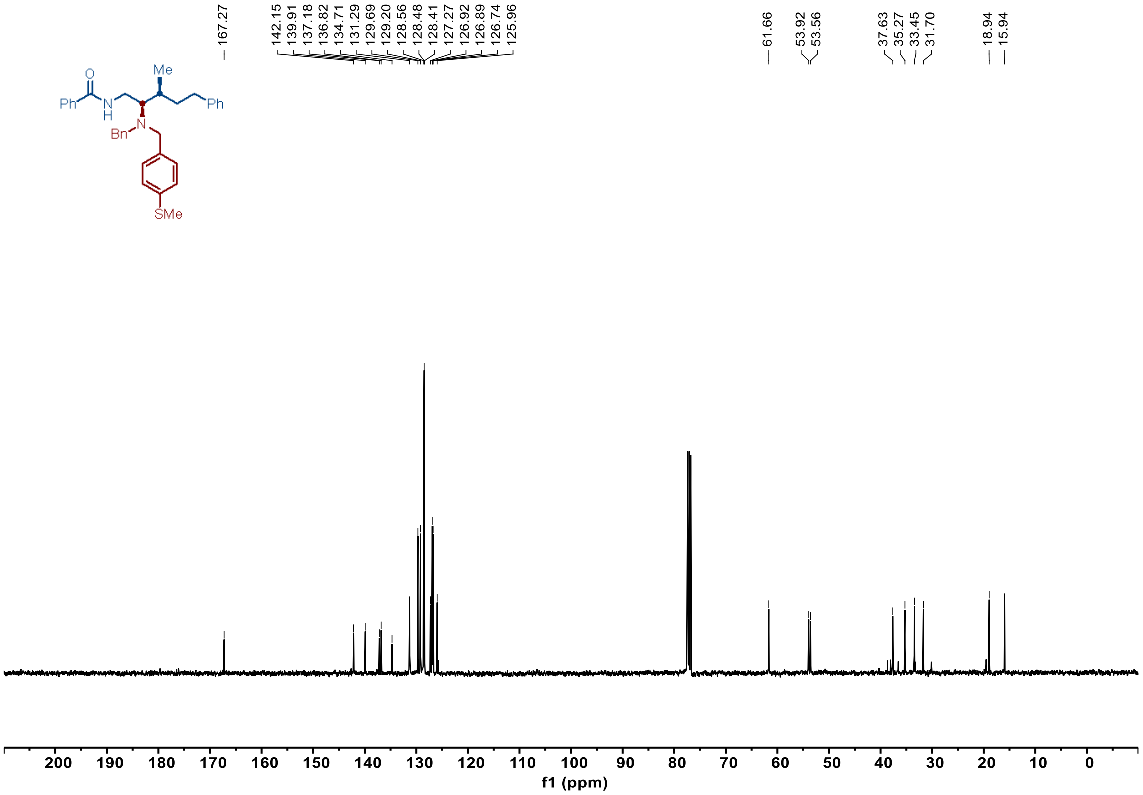

^{13}C NMR (101 MHz, $CDCl_3$) spectra of **(R,S)-3v**

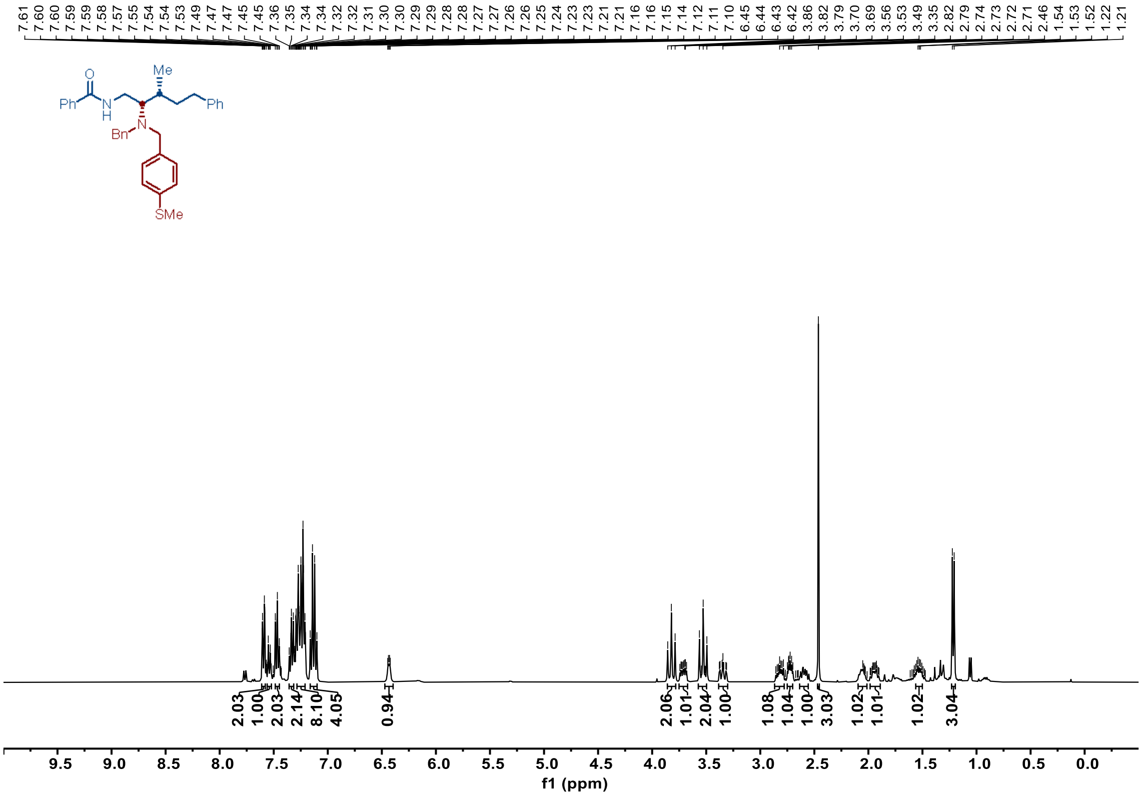

^{1}H NMR (400 MHz, $CDCl_3$) spectra of **(S,R)-3v**

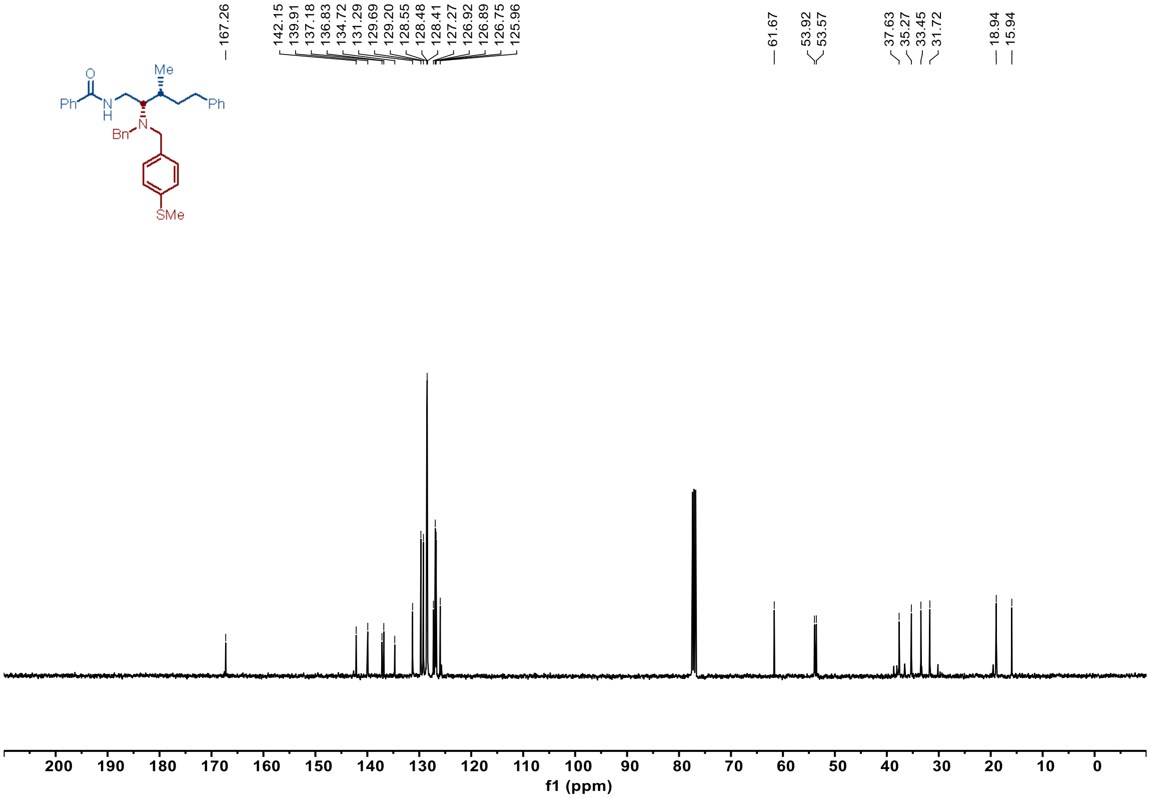

^{13}C NMR (101 MHz, $CDCl_3$) spectra of **(S,R)-3v**

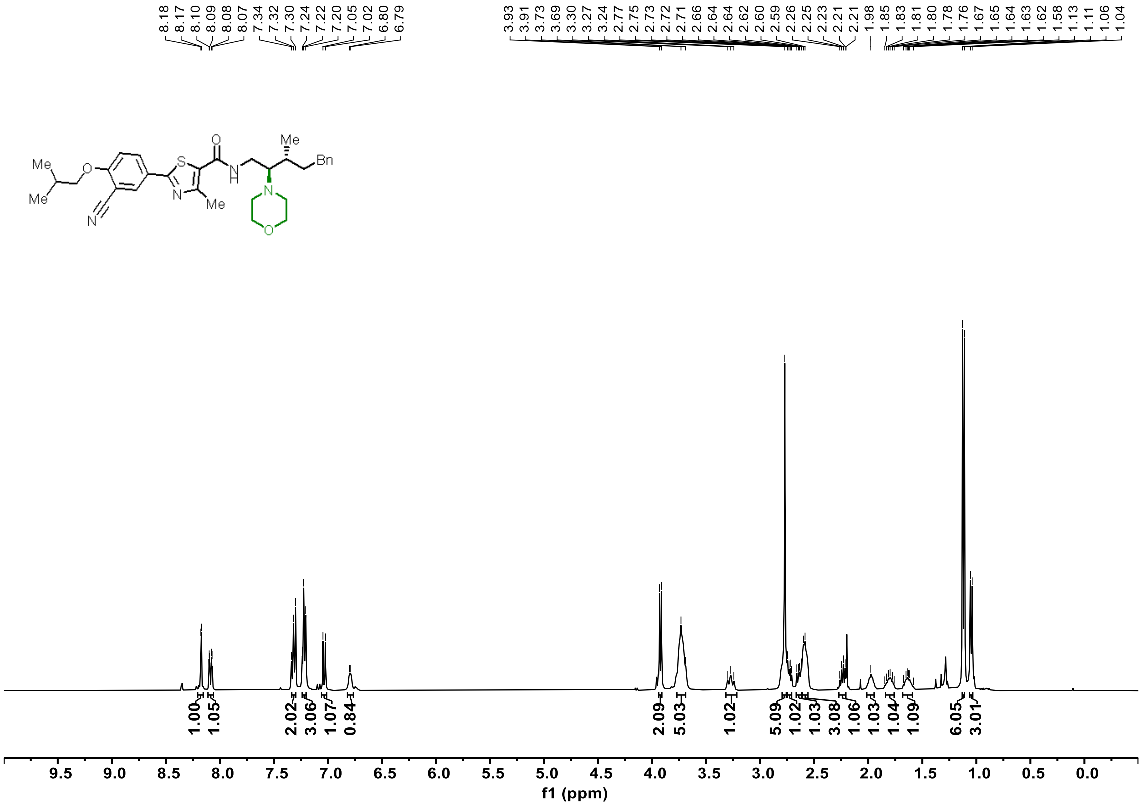

^{1}H NMR (400 MHz, $CDCl_3$) spectra of **5a**

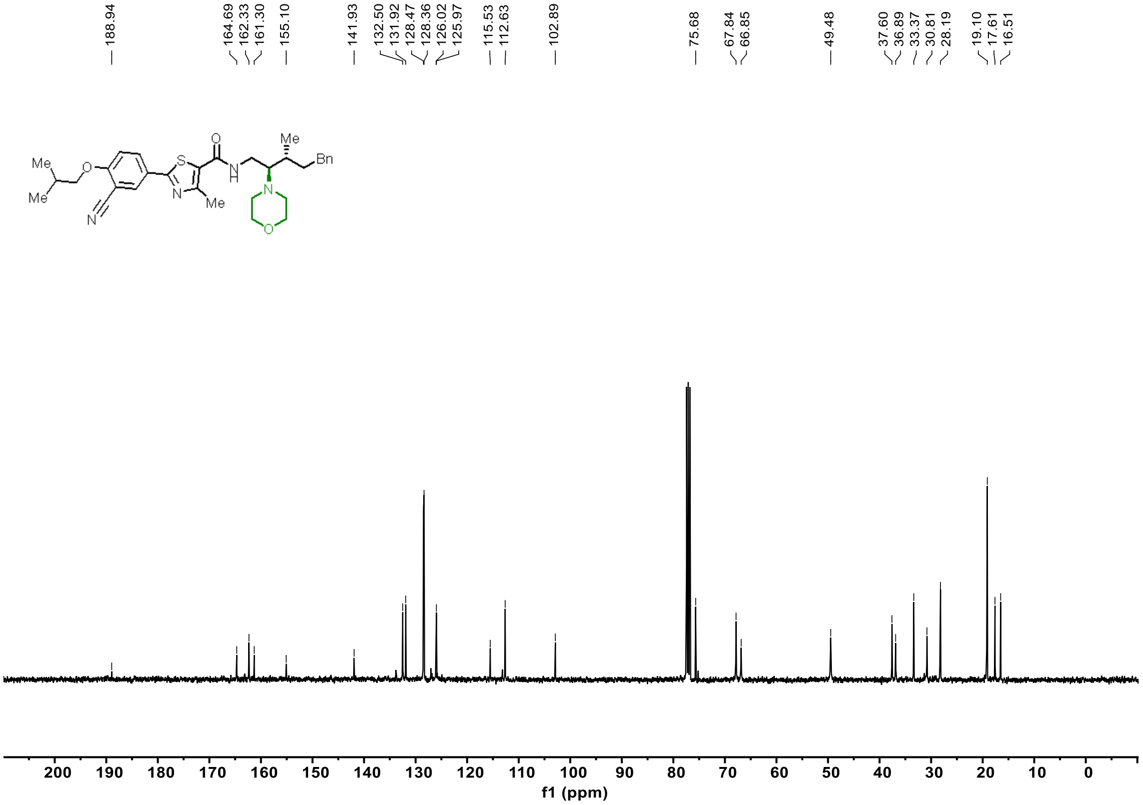

^{13}C NMR (101 MHz, $CDCl_3$) spectra of **5a**

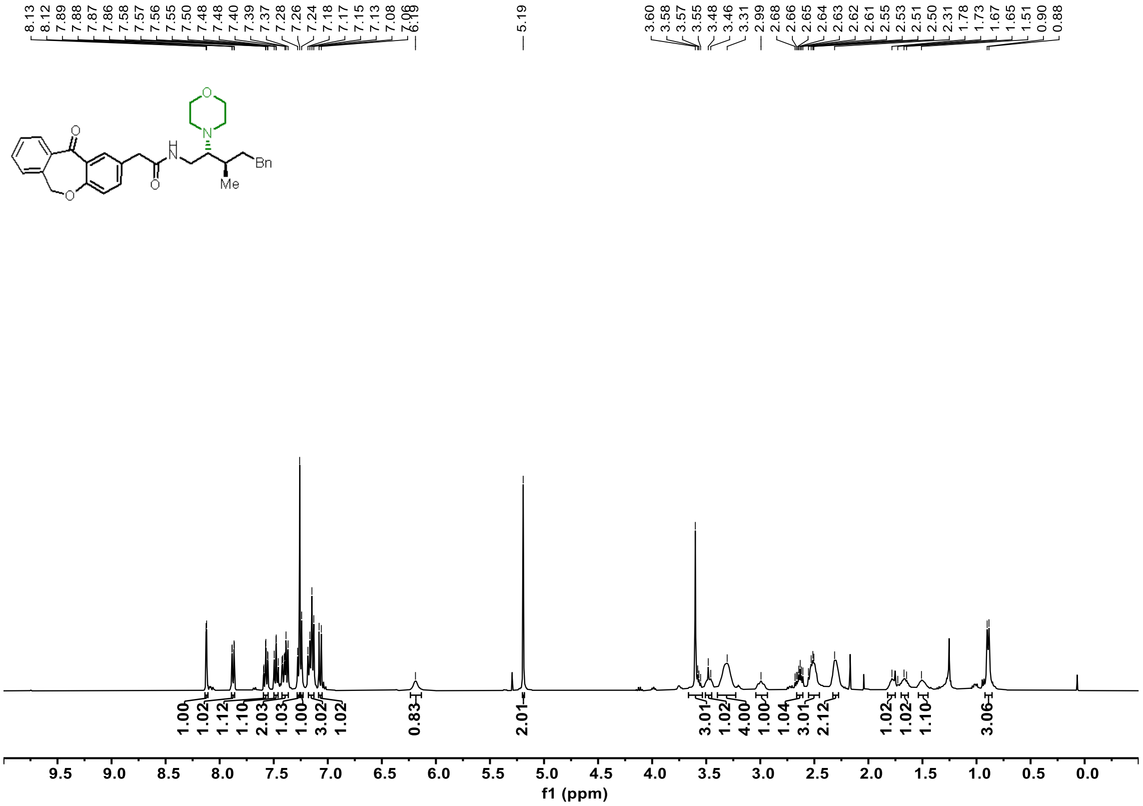

^{1}H NMR (400 MHz, $CDCl_3$) spectra of **5b**

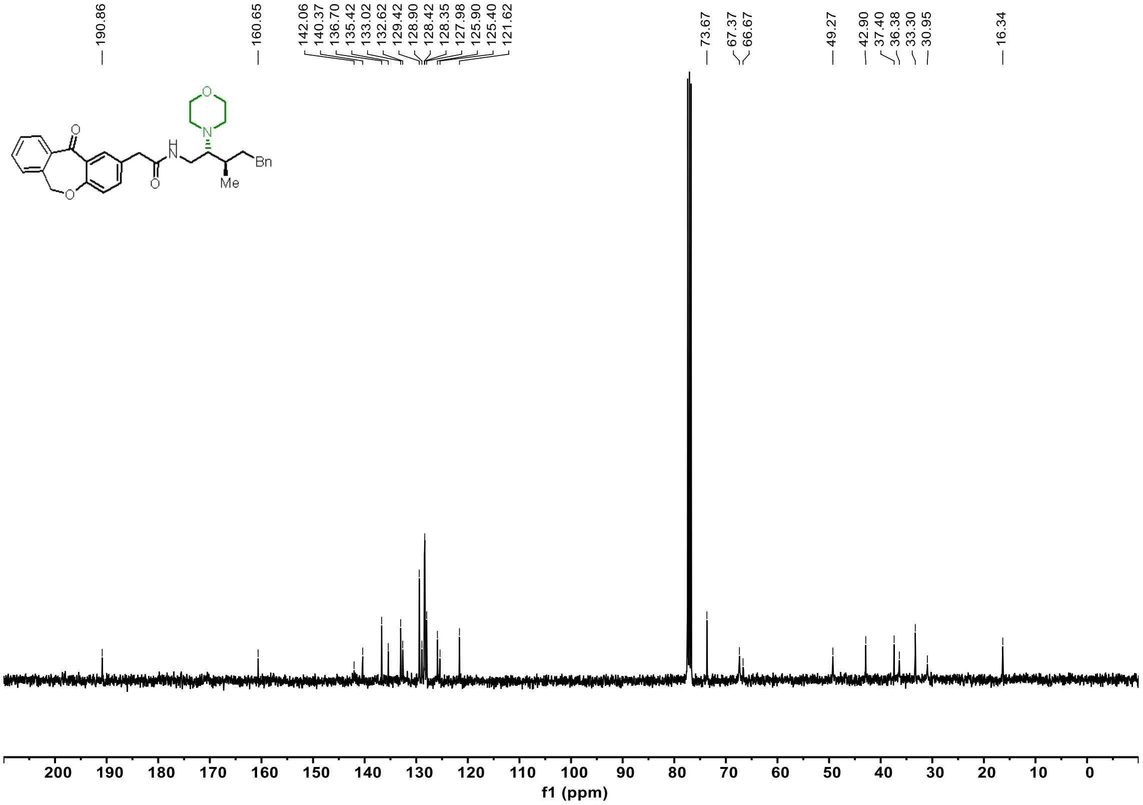

^{13}C NMR (101 MHz, $CDCl_3$) spectra of **5b**

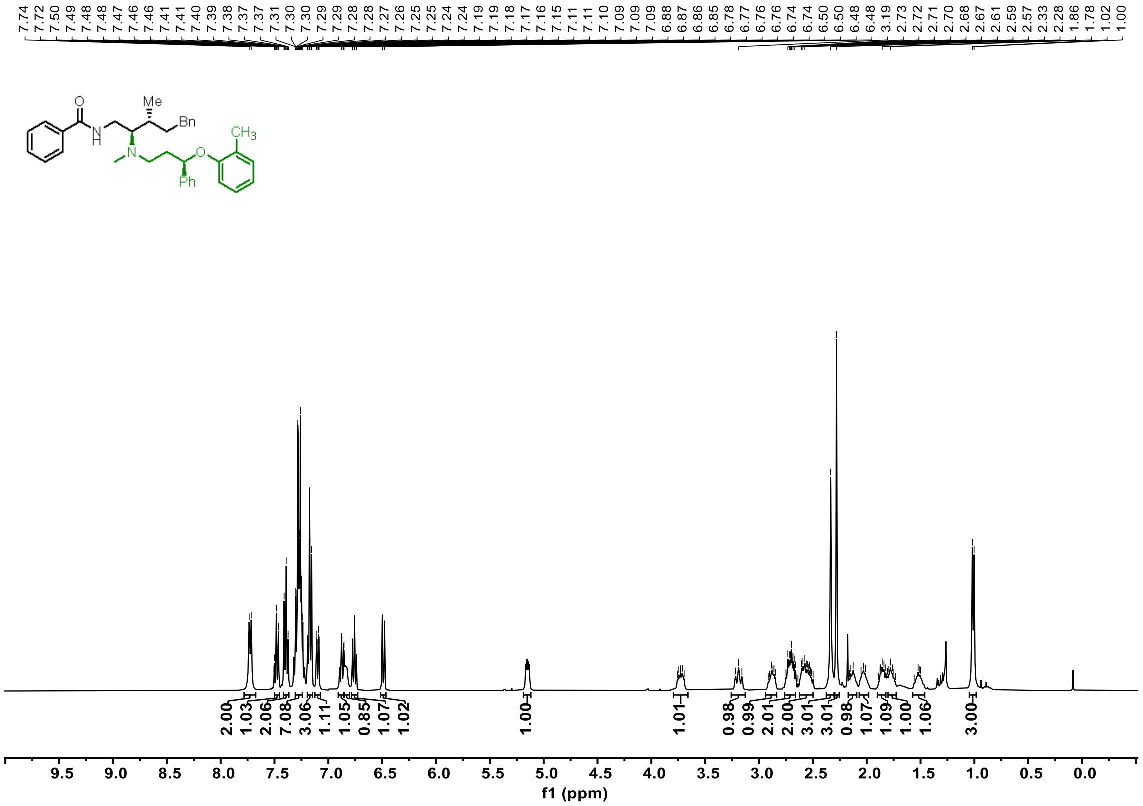

^{1}H NMR (400 MHz, $CDCl_3$) spectra of **5c**

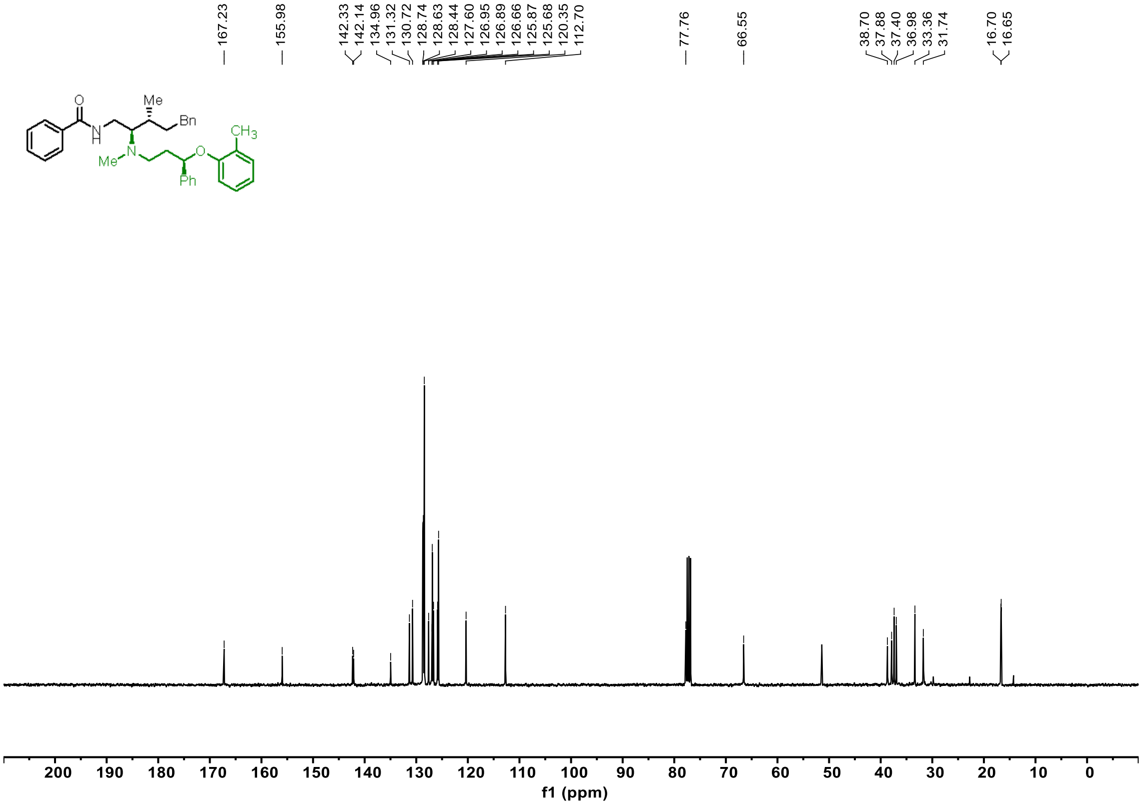

^{13}C NMR (101 MHz, $CDCl_3$) spectra of **5c**

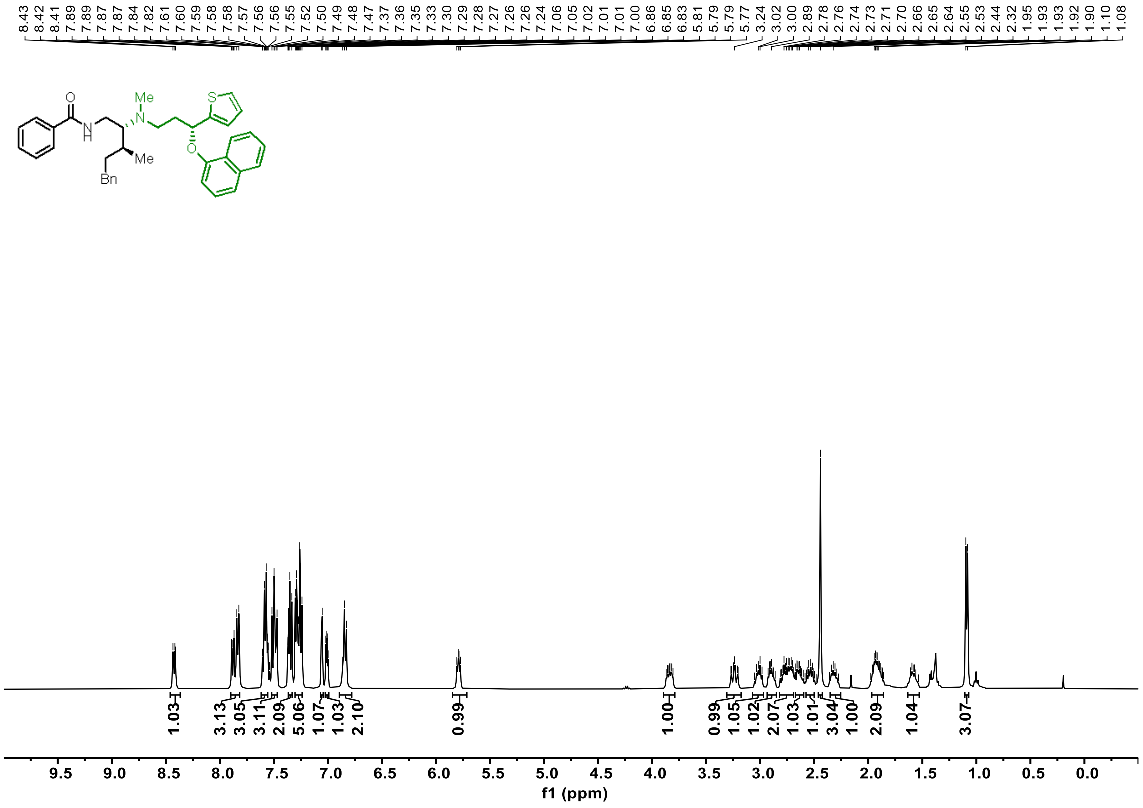

^{1}H NMR (400 MHz, $CDCl_3$) spectra of **5d**

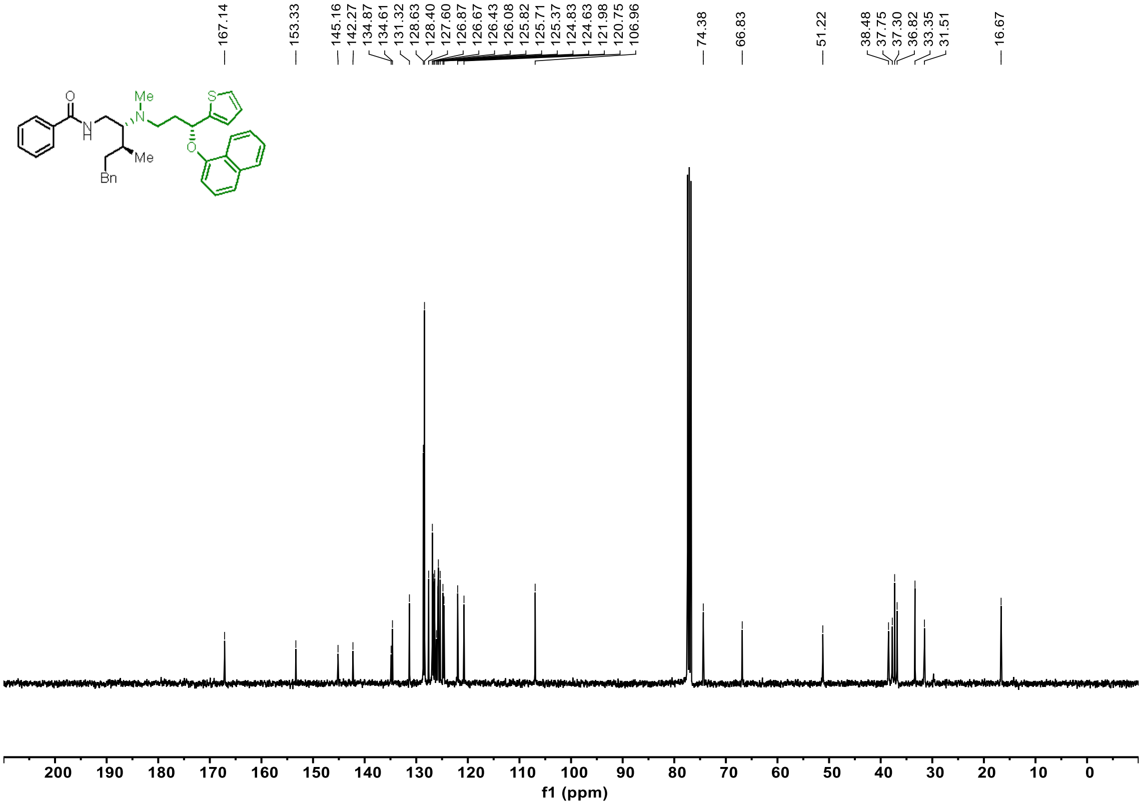

^{13}C NMR (101 MHz, $CDCl_3$) spectra of **5d**

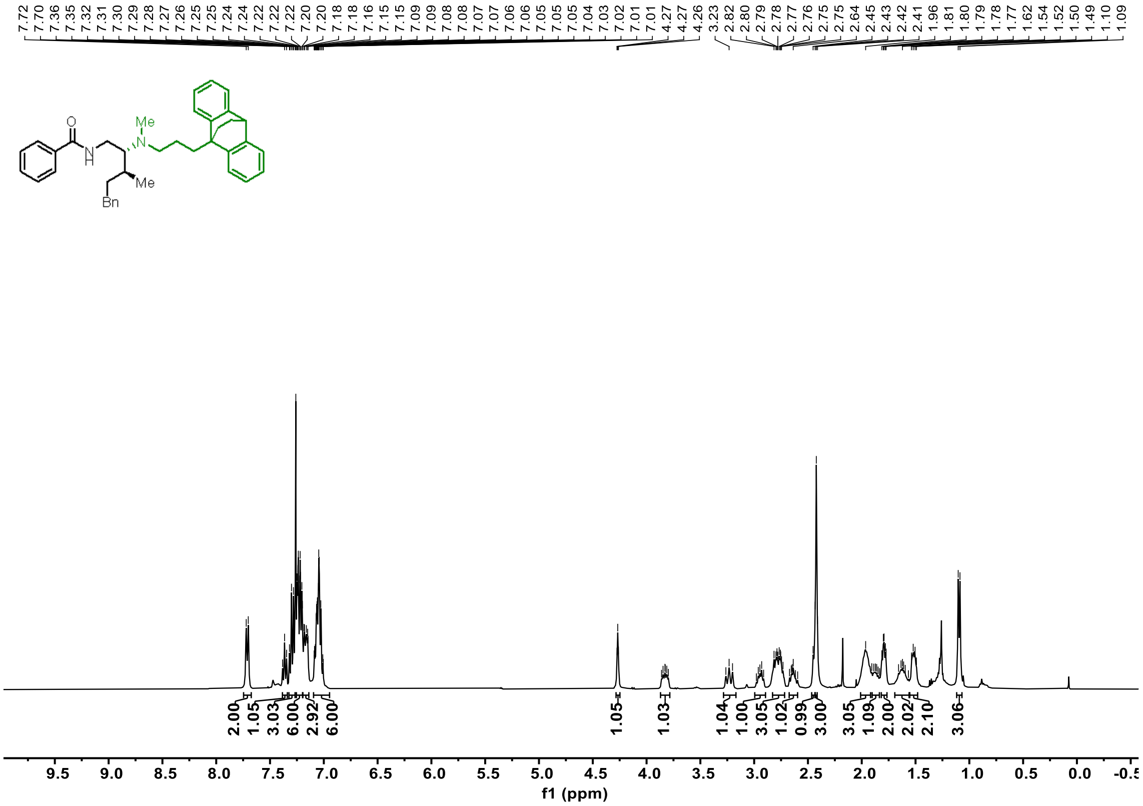

^{1}H NMR (400 MHz, $CDCl_3$) spectra of **5e**

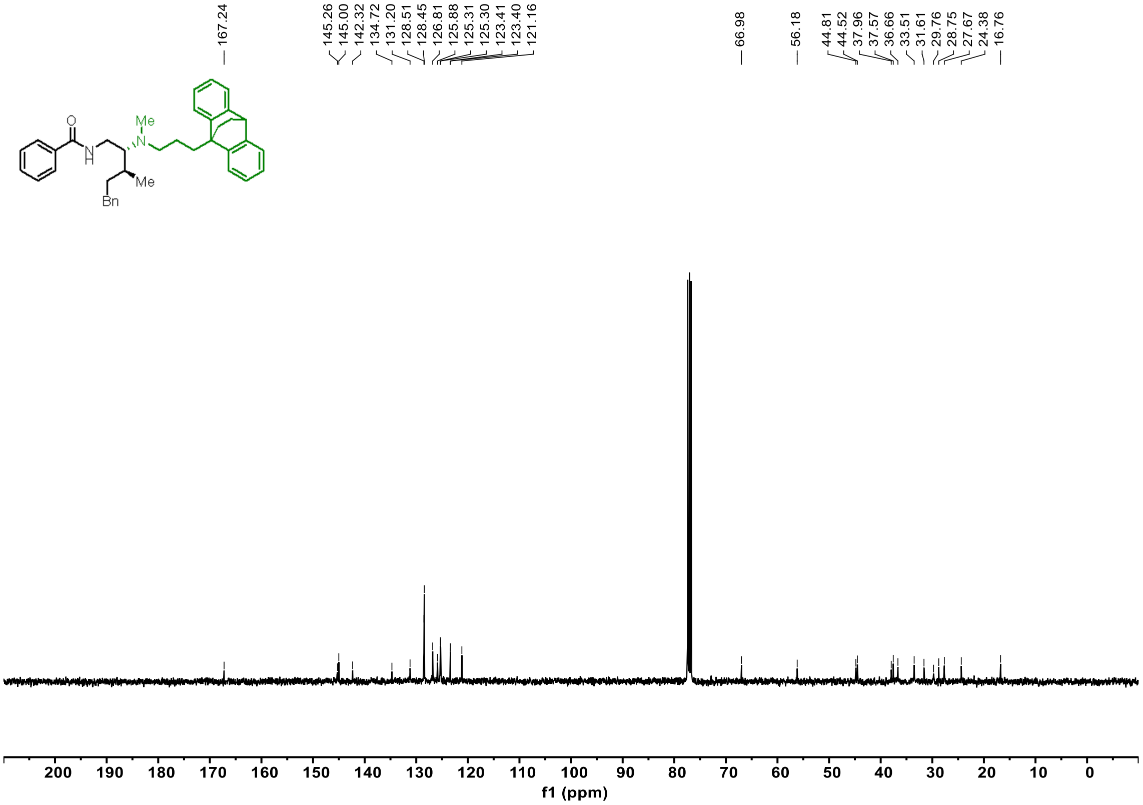

^{13}C NMR (101 MHz, $CDCl_3$) spectra of **5e**

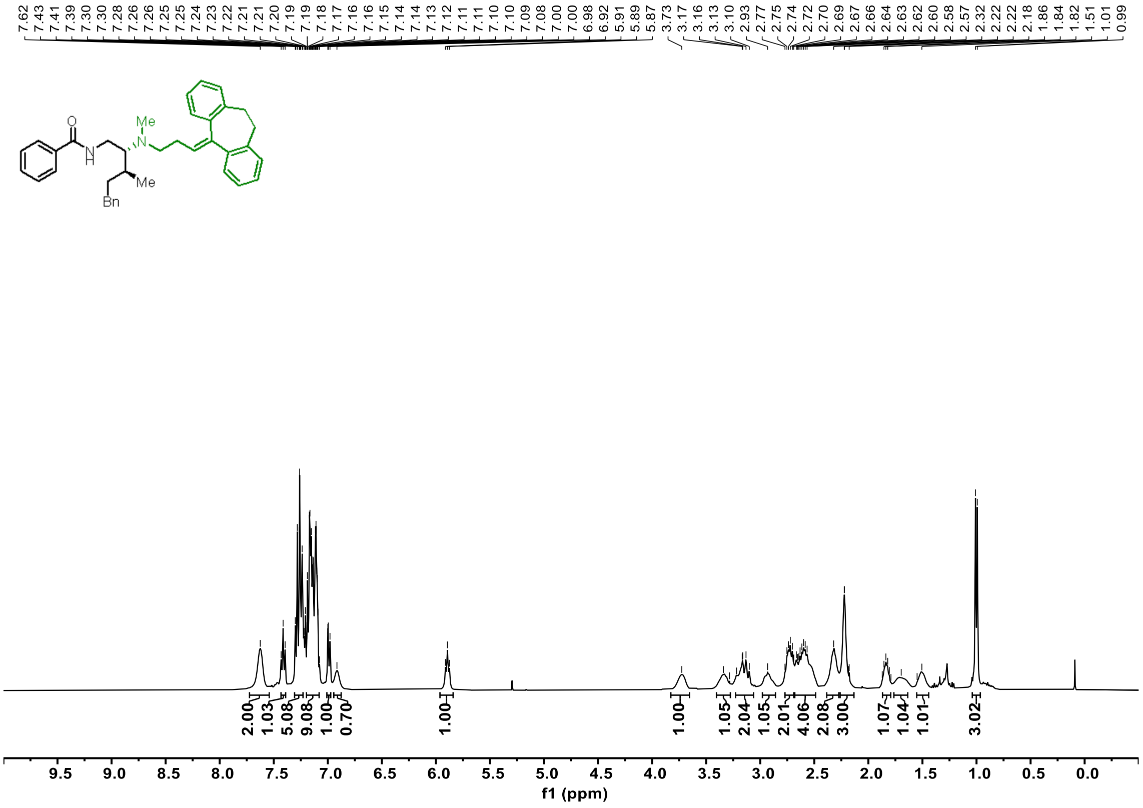

^{1}H NMR (400 MHz, $CDCl_3$) spectra of **5f**

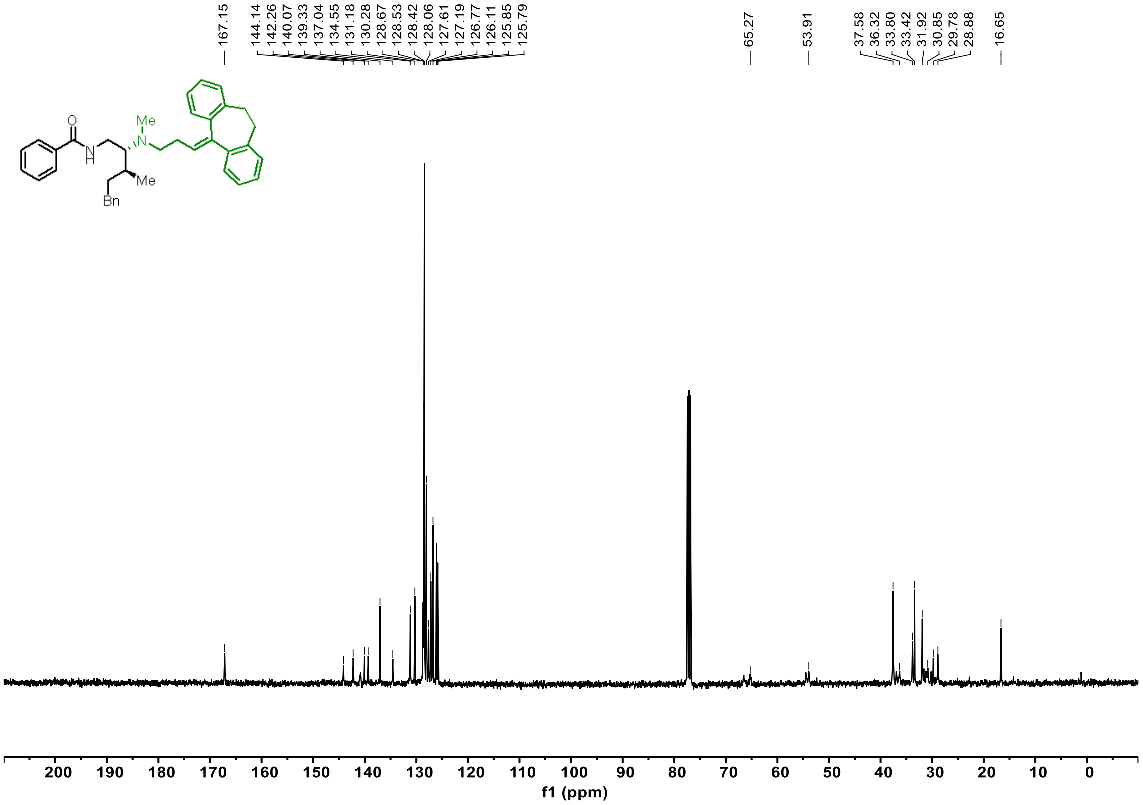

^{13}C NMR (101 MHz, $CDCl_3$) spectra of **5f**

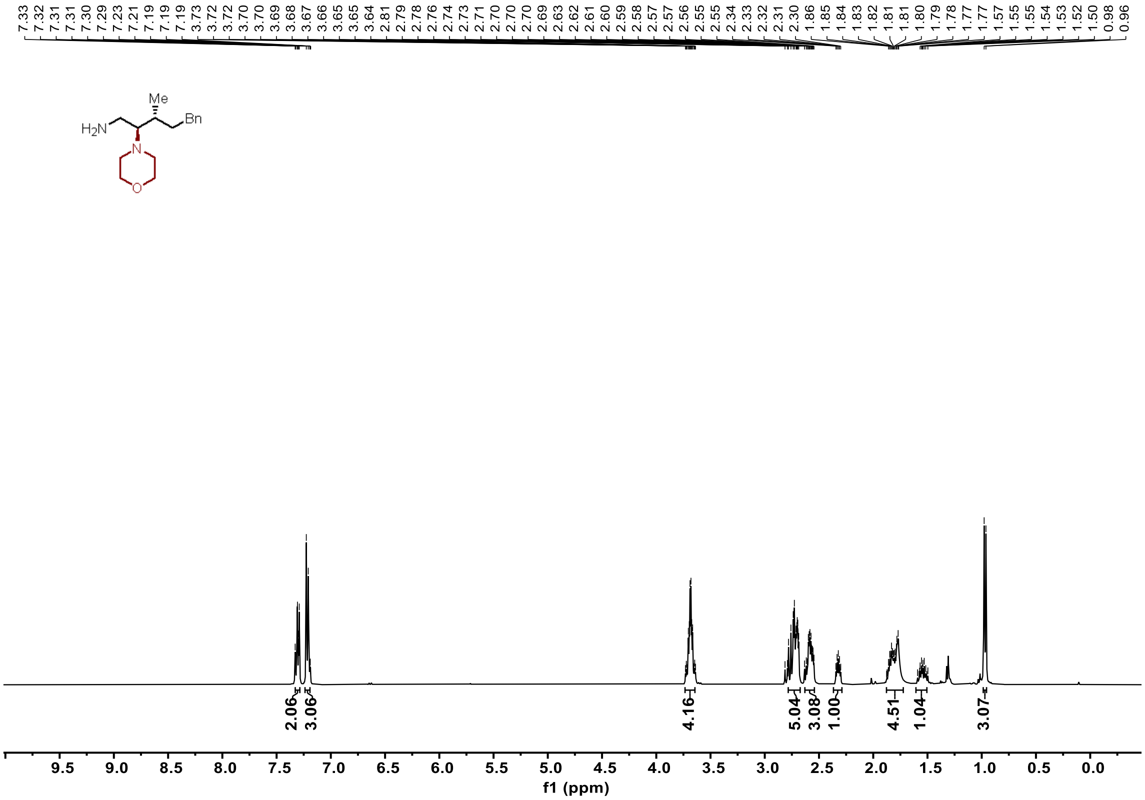

^{1}H NMR (400 MHz, $CDCl_3$) spectra of **6a**

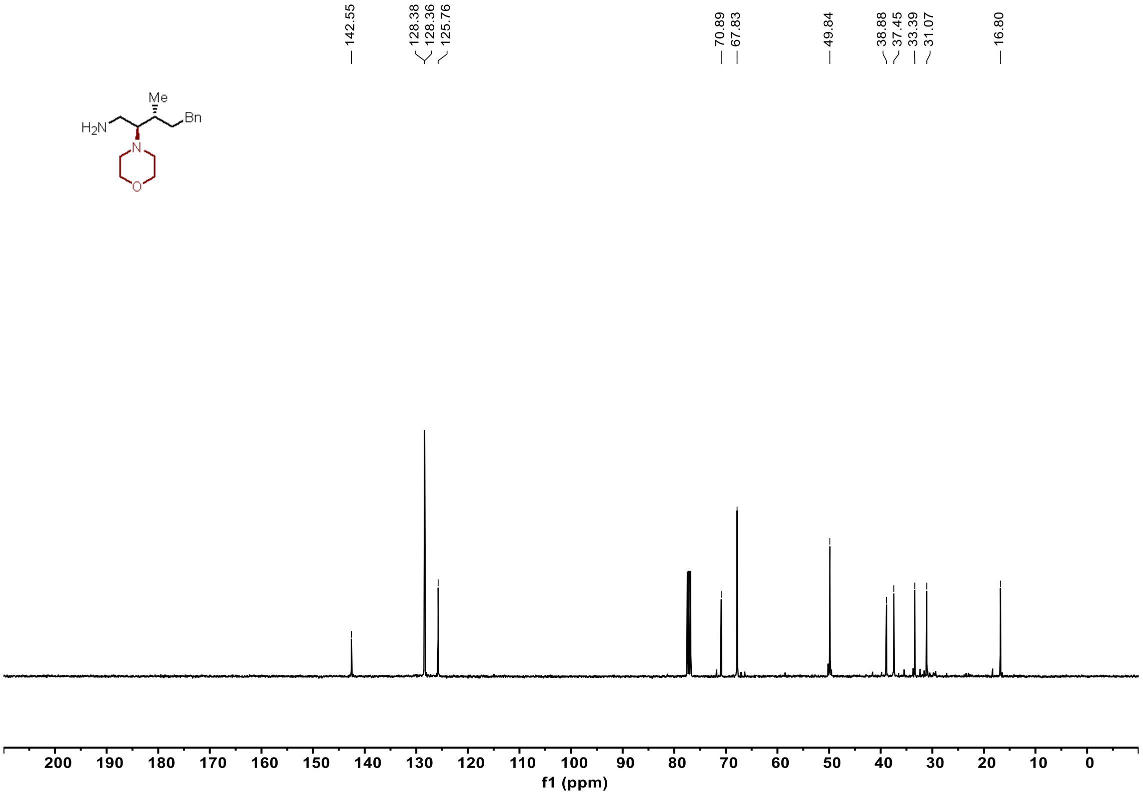

^{13}C NMR (101 MHz, $CDCl_3$) spectra of **6a**

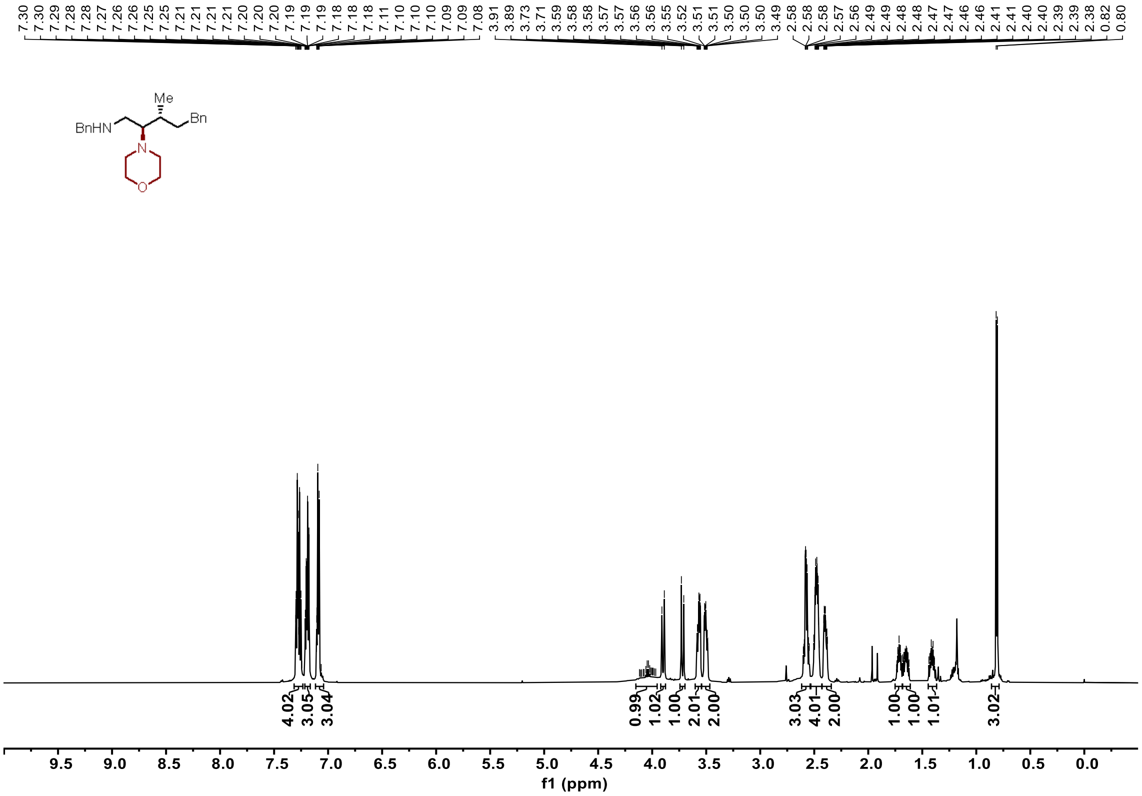

^{1}H NMR (400 MHz, $CDCl_3$) spectra of **6b**

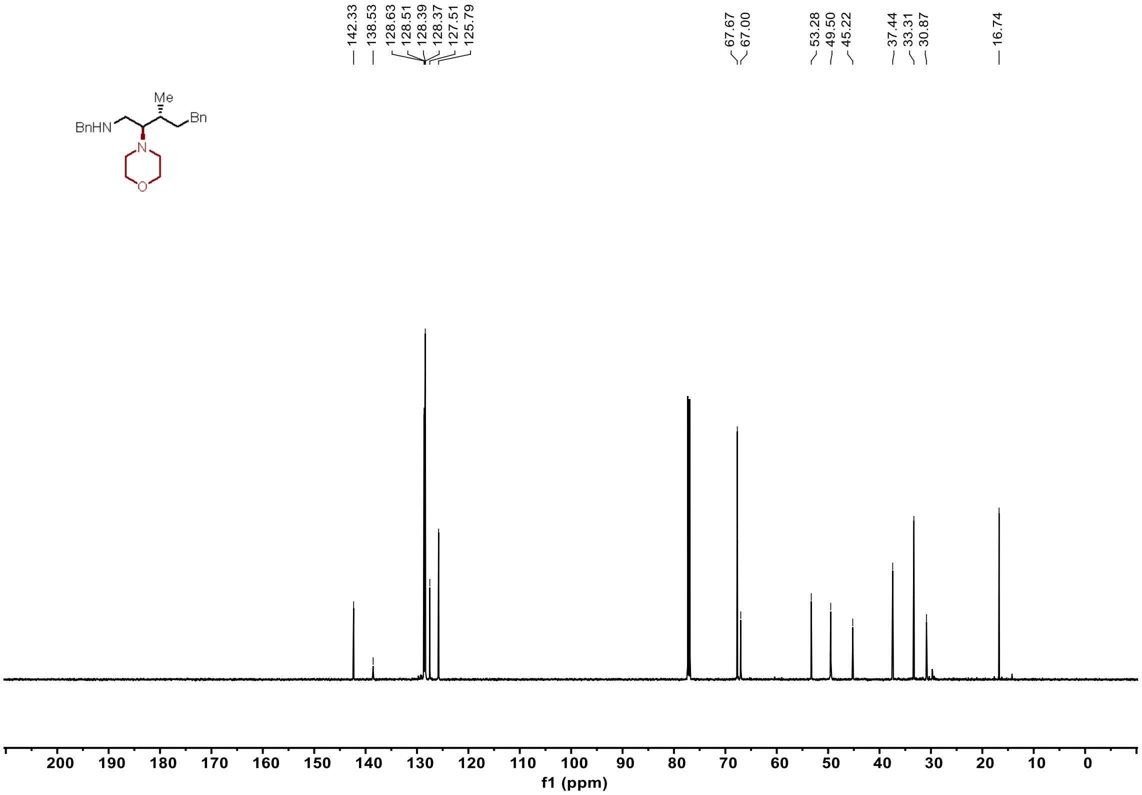

^{13}C NMR (101 MHz, $CDCl_3$) spectra of **6b**

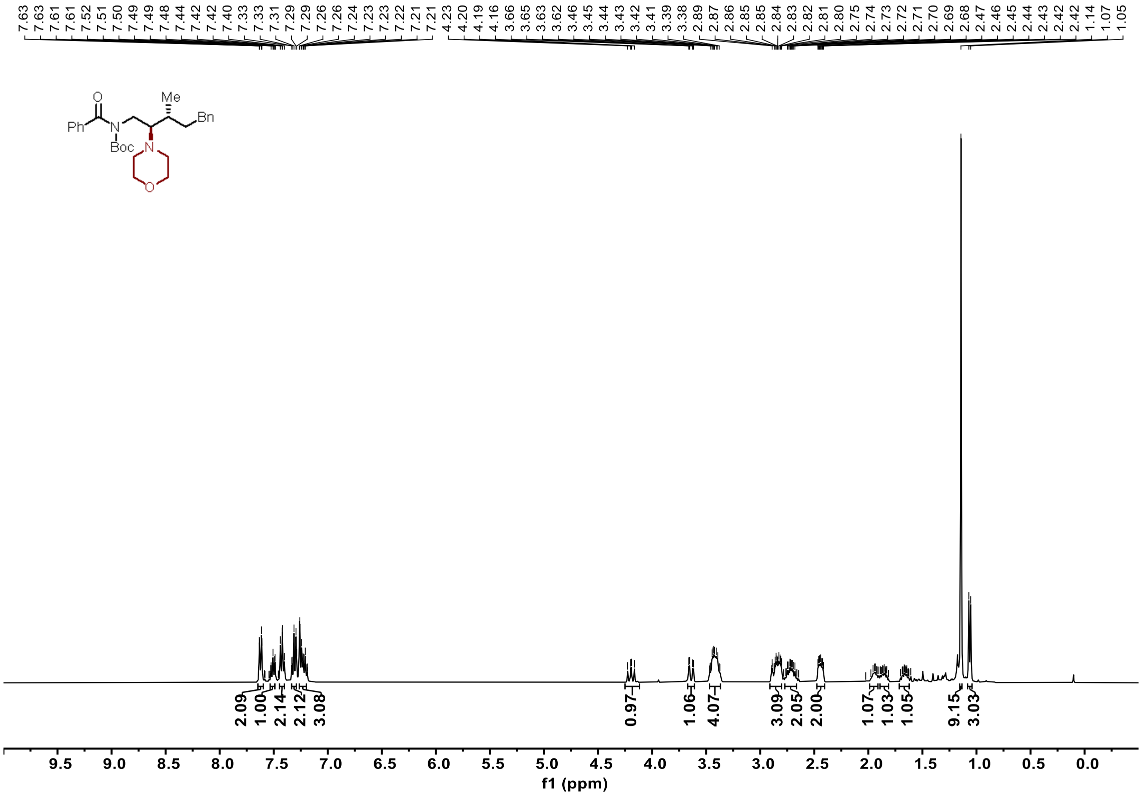

^{1}H NMR (400 MHz, $CDCl_3$) spectra of **6c**

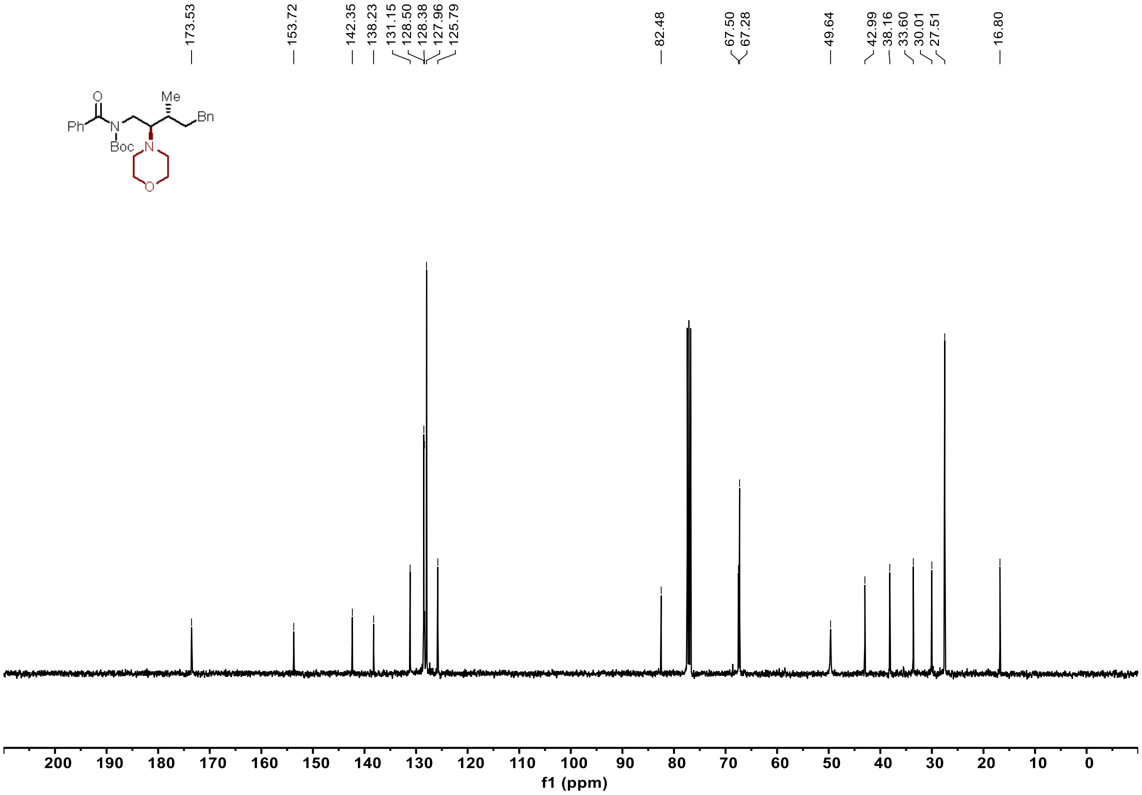

^{13}C NMR (101 MHz, $CDCl_3$) spectra of **6c**

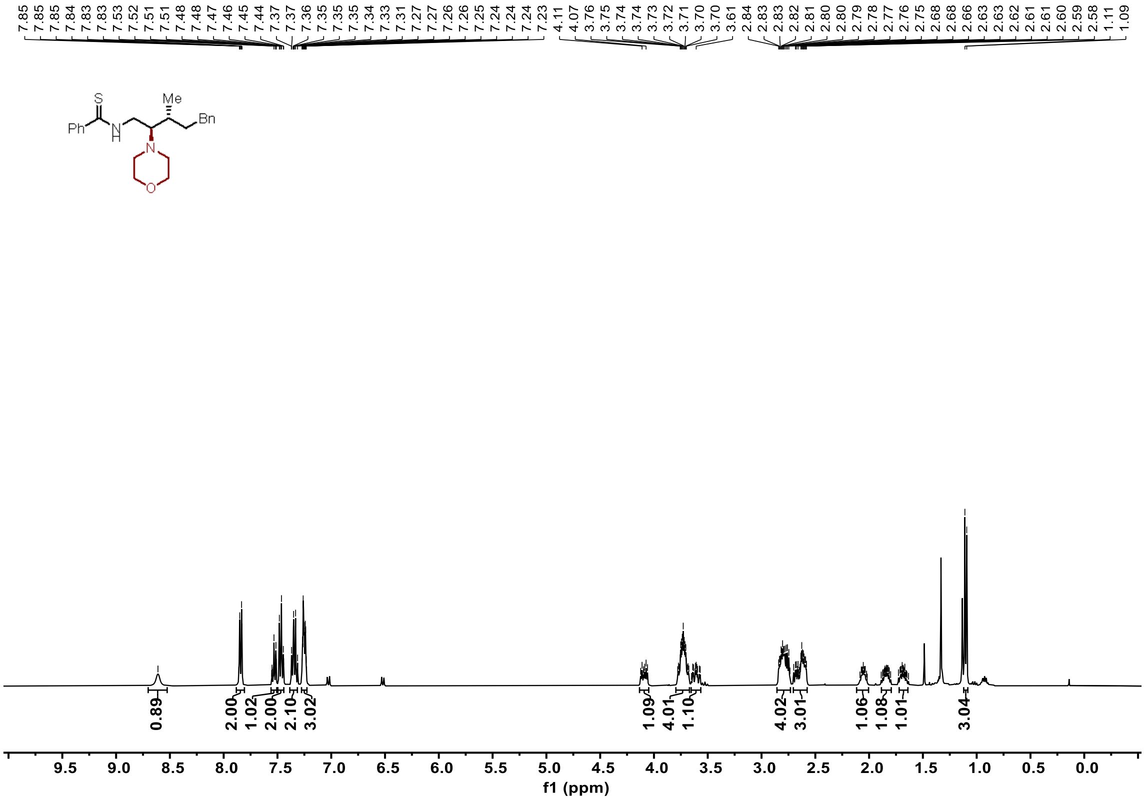

^{1}**H NMR** (400 MHz, CDCl$_3$) spectra of **6d**

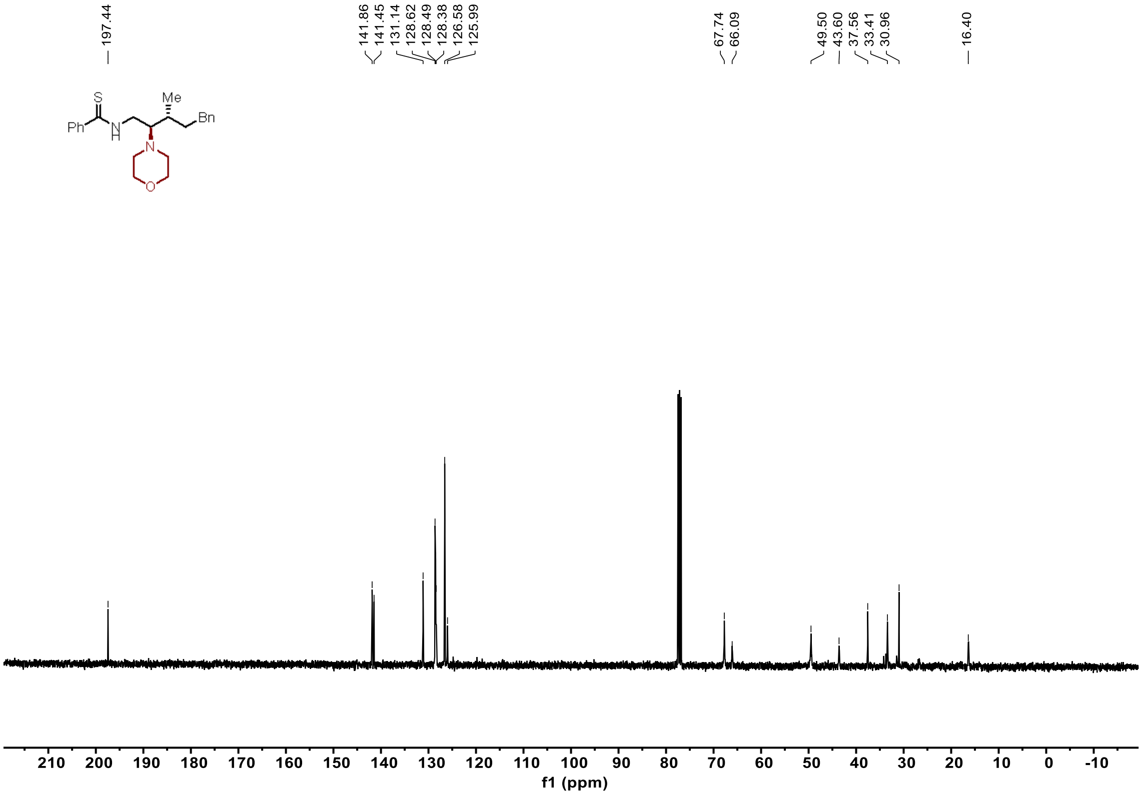

^{13}**C NMR** (101 MHz, CDCl$_3$) spectra of **6d**

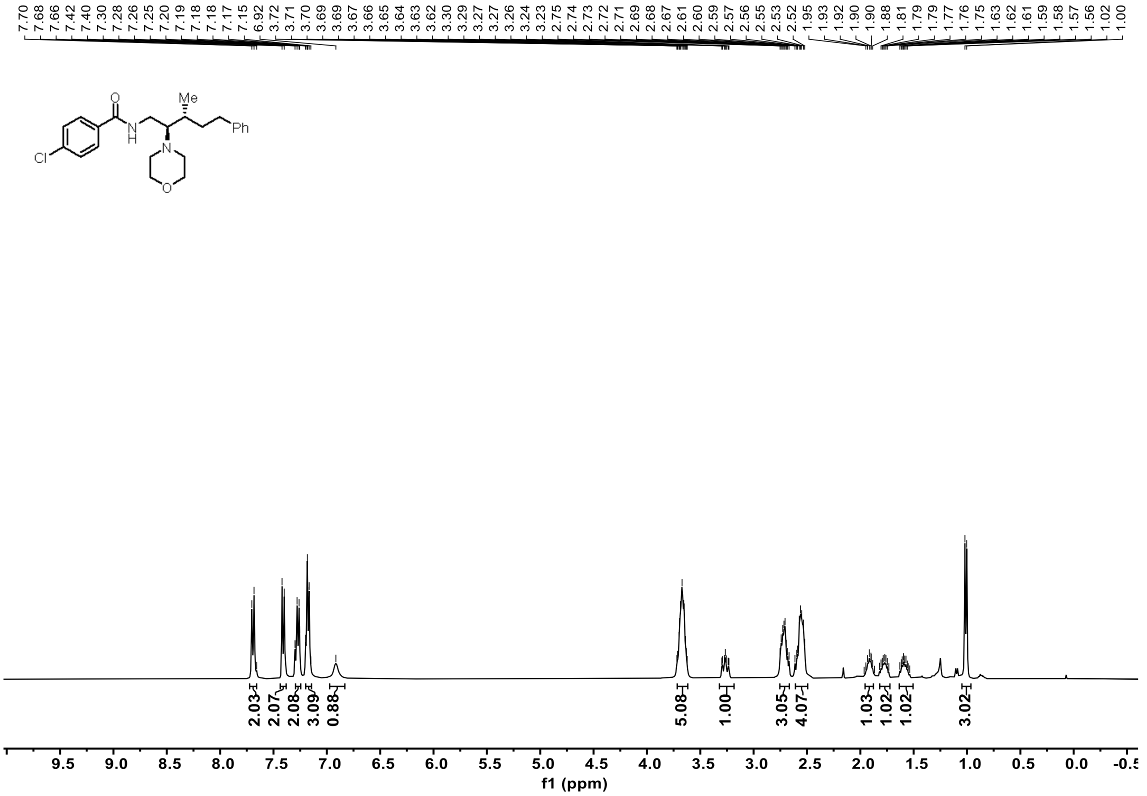

^{1}H NMR (400 MHz, $CDCl_3$) spectra of **7a**

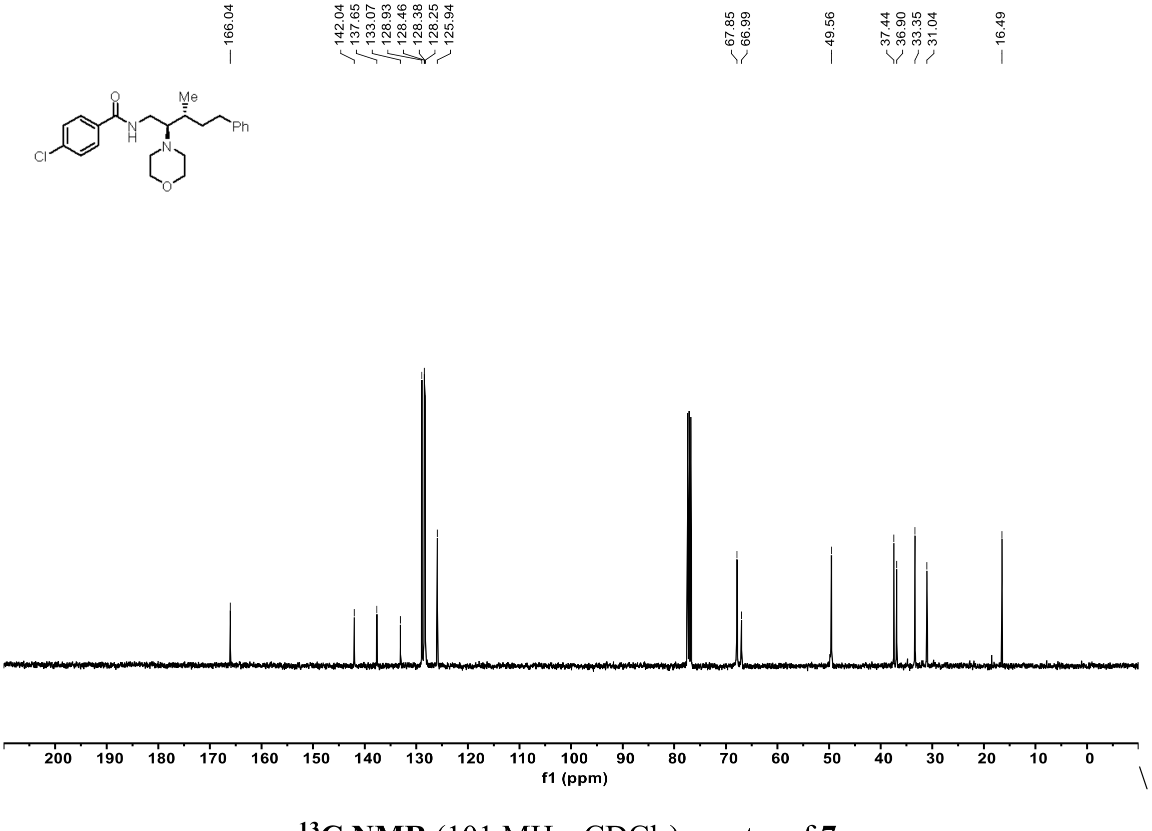

^{13}C NMR (101 MHz, $CDCl_3$) spectra of **7a**

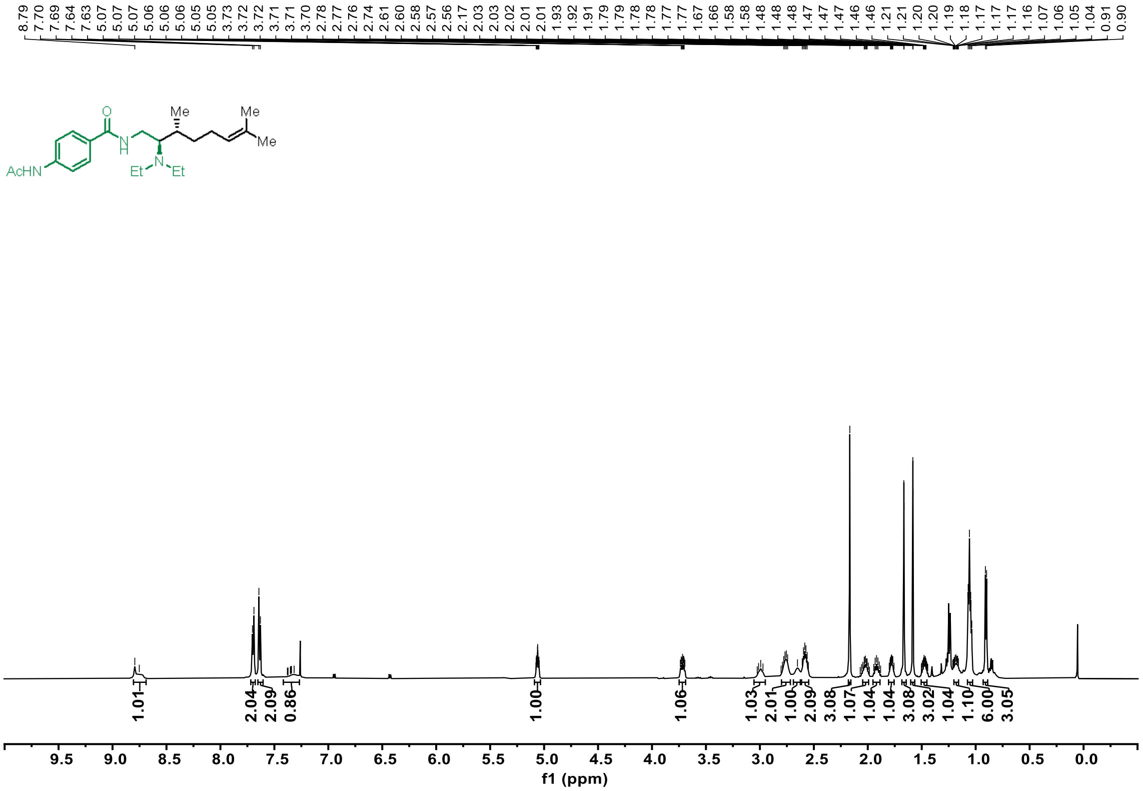

^{1}H NMR (400 MHz, $CDCl_3$) spectra of **7b**

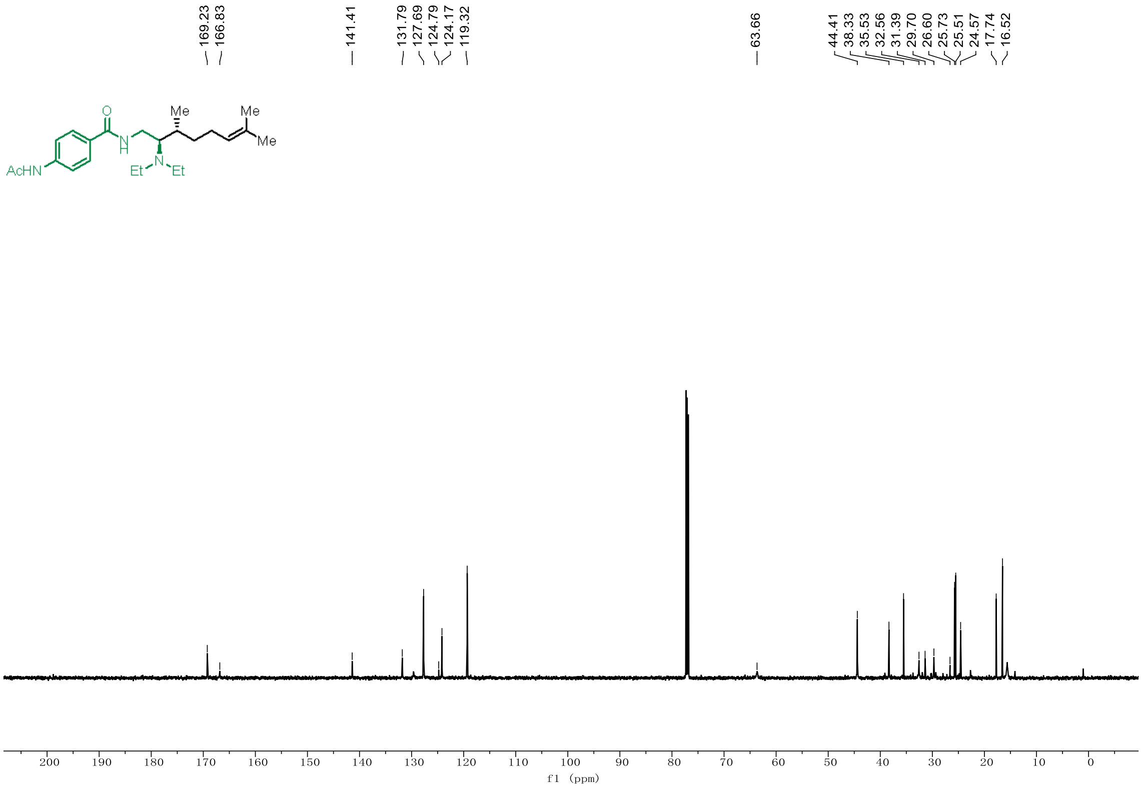

^{13}C NMR (101 MHz, $CDCl_3$) spectra of **7b**

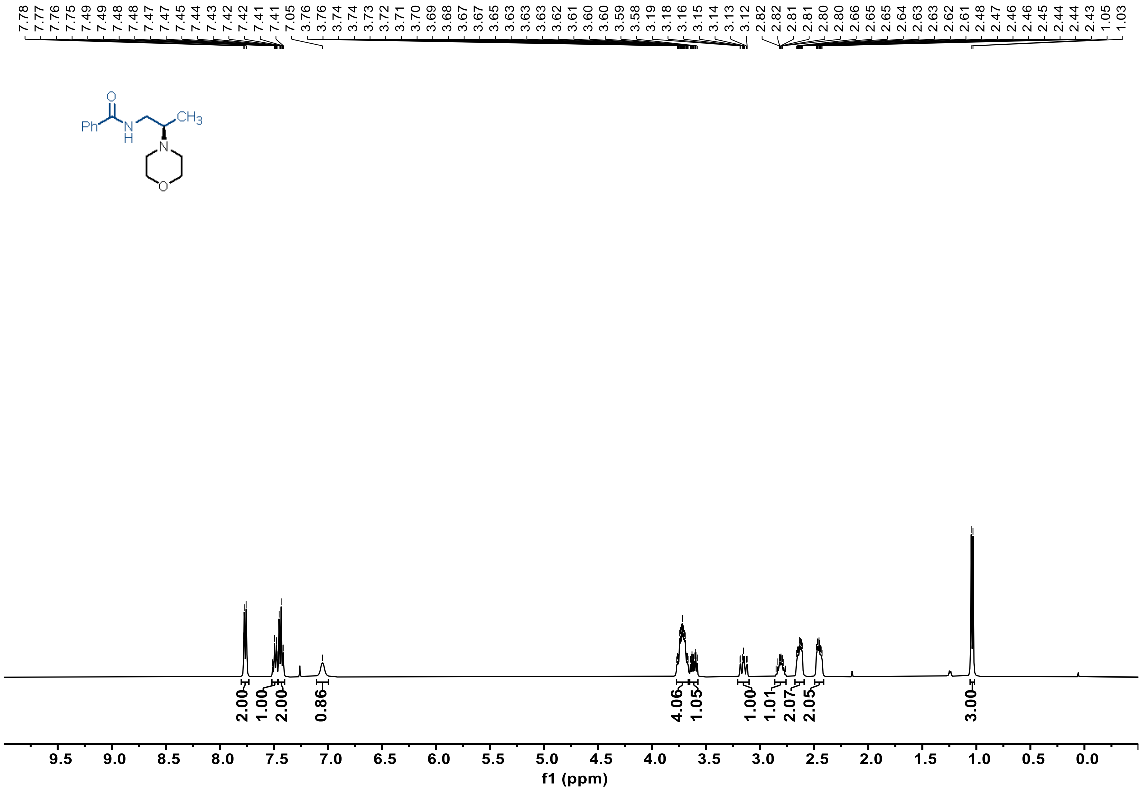

^{1}H NMR (400 MHz, $CDCl_3$) spectra of **9a**

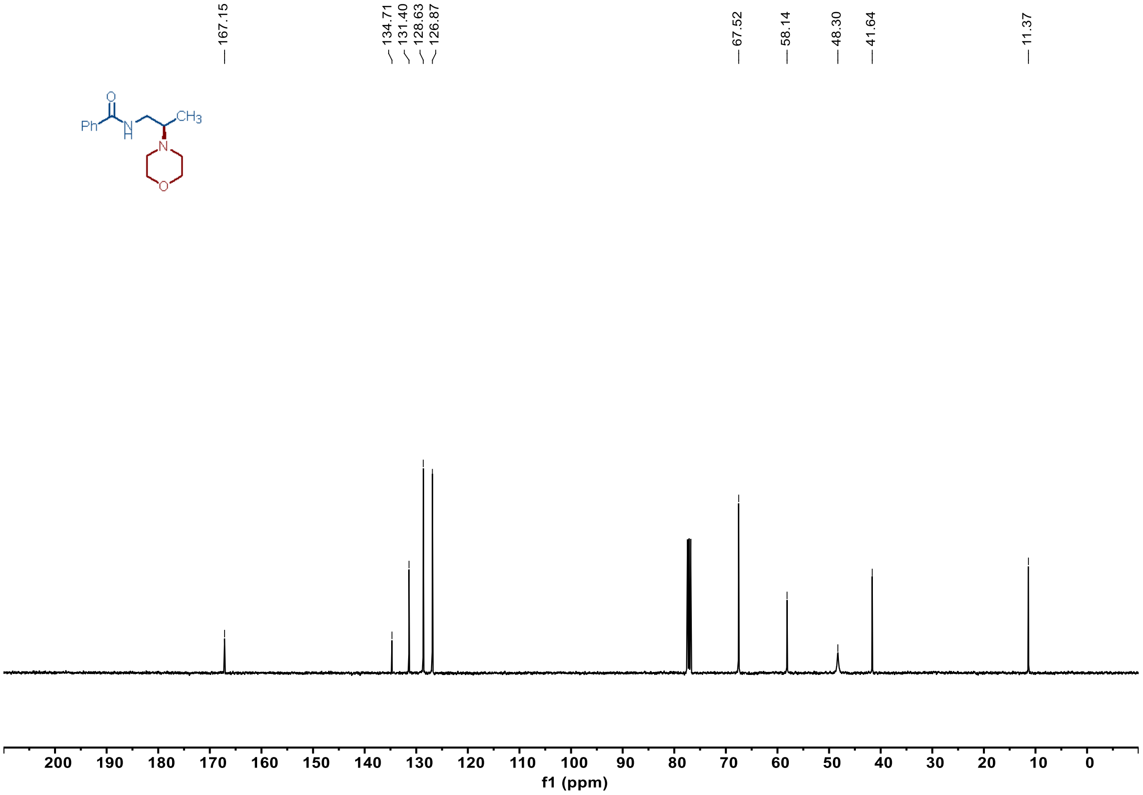

^{13}C NMR (101 MHz, $CDCl_3$) spectra of **9a**

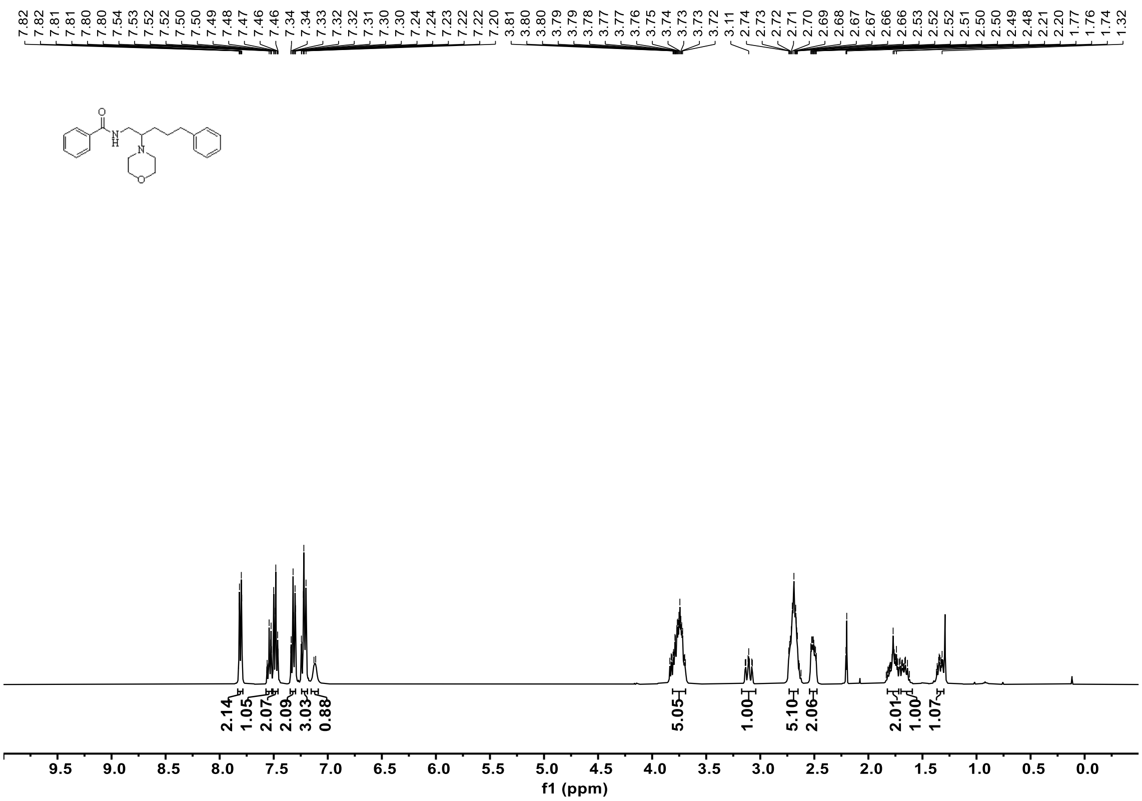

^{1}H NMR (400 MHz, $CDCl_3$) spectra of **9b**

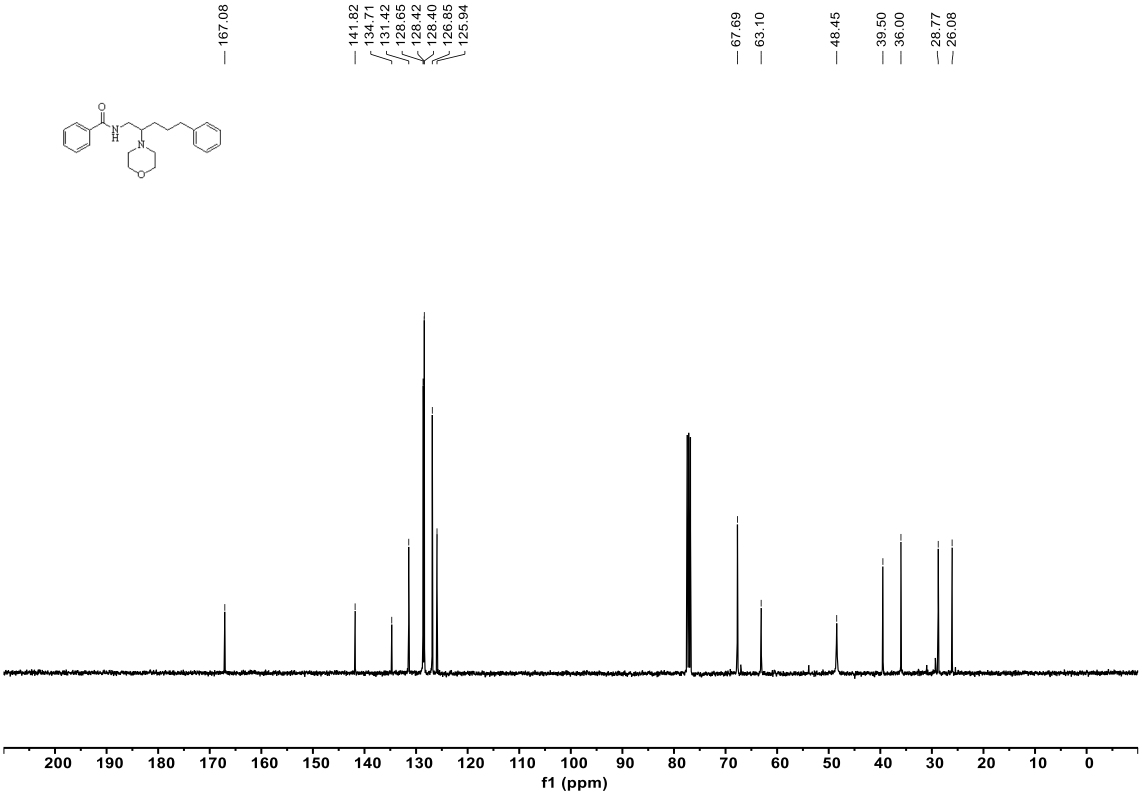

^{13}C NMR (101 MHz, $CDCl_3$) spectra of **9b**

12. Supplementary references

1. Zhao, W., Chen, K. Z., Li, A. Z. & Li, B. J. Remote Stereocenter through Amide-Directed, Rhodium-Catalyzed Enantioselective Hydroboration of Unactivated Internal Alkenes. *J. Am. Chem. Soc.* **144**, 13071-13078 (2022).

2. Zhang, S. et al. An Isopentenol Utilization Pathway-Based "Deuterium-Scanning" Method for Mechanistic Investigations of Terpene Cyclases. *ACS Catal.* **14**, 17598-17608 (2024).

3. Xie L. P., Liang J. M., Bai H. H., Liu X. Y., Meng X., Xu Y-Q, Cao Z-Y., Wang C. Ligand-Controlled NiH-Catalyzed Regiodivergent and Enantioselective Hydroamination of Alkenyl Amides. A*CS Catal.* **13**, 10041. (2023).

4. Habtemariam et al. Control of aminophosphine chelate ring-opening in Pt(II) and Pd(II) complexes: potential dual-mode anticancer agents. *J. Chem. Soc., Dalton Trans*. **8**, 1306-1318 (2001).

5. Wang Q., Tao X. Z., Ni S. Y., Pan Y., Wang Y. Nickel-Catalyzed Amination of Alkyl Electrophiles. *Org. Lett*. **25**, 5822−5826 (2023).

6. Lu, L., Chen, S., Kong, W., Gao, B., Li, Y., Zhu, L. & Yin, G. Enantioselective Synthesis of β-Aminoboronic Acids via Borylalkylation of Enamides. *J. Am. Chem. Soc.* **146**, 16639–16647 (2024).

7. Schönbauer, David., Sambiagio, Carlo., Noël, Timothy., Schnürch, Michael. Photocatalytic deaminative benzylation and alkylation of tetrahydroisoquinolines with N-alkylpyrydinium salts. *Beilstein J. Org. Chem*. **16**, 809–817 (2020).

8. Verho, Oscar., Pourghasemi Lati., Monireh; Oschmann, Michael. A Two-Step Procedure for the Overall Transamidation of 8-Aminoquinoline Amides Proceeding via the Intermediate N -Acyl-Boc-Carbamates. *Org. Chem*. **83**, 4464–4476 (2018).

9. Zhao, Q., Li, G. C., Nareddy, P. Jordan, F., Lalancette, R., Szostak, R., Szostak, M. Structures of the Most Twisted Thioamide and Selenoamide: Effect of Higher Chalcogens of Twisted Amides on N−C(X) Resonance. *Angew. Chem. Int. Ed*. **61**, e202207346 (2022).
